# Evolution based on domain combinations: the case of glutaredoxins

**DOI:** 10.1186/1471-2148-9-66

**Published:** 2009-03-25

**Authors:** Rui Alves, Ester Vilaprinyo, Albert Sorribas, Enrique Herrero

**Affiliations:** 1 Departament de Ciències Mèdiques Bàsiques, Universitat de Lleida, IRBLleida, Montserrat Roig 2, 25008, Lleida, Spain; 2 Institut d'Investigació Biomédica de Bellvitge (IDIBELL), Hospitalet de Llobregat, Spain

## Abstract

**Background:**

Protein domains represent the basic units in the evolution of proteins. Domain duplication and shuffling by recombination and fusion, followed by divergence are the most common mechanisms in this process. Such domain fusion and recombination events are predicted to occur only once for a given multidomain architecture. However, other scenarios may be relevant in the evolution of specific proteins, such as convergent evolution of multidomain architectures. With this in mind, we study glutaredoxin (GRX) domains, because these domains of approximately one hundred amino acids are widespread in archaea, bacteria and eukaryotes and participate in fusion proteins. GRXs are responsible for the reduction of protein disulfides or glutathione-protein mixed disulfides and are involved in cellular redox regulation, although their specific roles and targets are often unclear.

**Results:**

In this work we analyze the distribution and evolution of GRX proteins in archaea, bacteria and eukaryotes. We study over one thousand GRX proteins, each containing at least one GRX domain, from hundreds of different organisms and trace the origin and evolution of the GRX domain within the tree of life.

**Conclusion:**

Our results suggest that single domain GRX proteins of the CGFS and CPYC classes have, each, evolved through duplication and divergence from one initial gene that was present in the last common ancestor of all organisms. Remarkably, we identify a case of convergent evolution in domain architecture that involves the GRX domain. Two independent recombination events of a TRX domain to a GRX domain are likely to have occurred, which is an exception to the dominant mechanism of domain architecture evolution.

## Background

Domain duplication and shuffling by recombination and fusion, followed by divergence are the more frequent mechanisms for the evolution of proteins [1]. It has been estimated that such recombination and fusion events are likely to occur only once for a given multidomain architecture and that, after such an event, the fusion protein is duplicated and/or diverges over time [1]. In addition, statistical analysis of known multidomain proteins has shown that a) there is a strong bias for individual domains involved in recombination and fusion events to be short [2], and b) some specific sets of recombined domains (supra domains) participate in further recombination and fusion events [3]. This provides a model for the dominant mode of domain architecture evolution in proteins that is very much consensual. Recent work has further estimated that between 88% and 95% of all multidomain architectures have evolved through such mechanisms [4-6]. The remaining architectures are likely to have evolved through convergent evolution [6]. A recent theory proposes that, during major evolutionary transitions, evolution is biphasic, further complicating the model of protein evolution [7]. According to this view, in an initial post-transition phase, large scale horizontal gene transfer (HGT) would occur. This would be followed by a second phase where the more common mechanisms for protein evolution become dominant. Given this background, it is of interest to analyze the evolution of a specific type of protein domain in order to assess the importance of the evolutionary mechanisms described above in the evolution of that domain.

A protein domain of small size that is known to participate in the architecture of multidomain proteins and is widespread over the many branches of the evolutionary tree would be an appropriate choice to study. The glutaredoxin (GRX) domain meets all these conditions. It has approximately one hundred amino acids, it is a part of several multidomain architectures and it is present in archaea, bacteria and eukaryotes. GRXs are thiol oxidoreductases responsible for the reduction of protein disulfides or glutathione-protein mixed disulfides, which employ reduced glutathione (GSH) as hydrogen donor [8]. Together with thioredoxins (TRXs) and other proteins, GRXs are grouped in the thioredoxin fold superfamily, because they share a common structural fold consisting of a four or five-stranded *β*-sheet flanked by several *α*-helices on either side of the *β*-sheet [9]. Functionally, TRXs are also disulfide oxireductases. In contrast with GRXs, oxidized TRXs are reduced by thioredoxins reductases at the expense of NADPH [8]. Alternatively, in plant chloroplasts, TRXs are reduced directly by the ferredoxin/ferredoxin reductase system that is coupled to photosynthesis [10]. Three GRX families have been defined based on the sequence of the putative active sites. Classical dithiol GRXs (CPYC class) with a C [P/S] [Y/F]C active site sequence are widespread in archaea, bacteria and eukaryotes. On the other hand, monothiol GRXs (CGFS class) contain a CGFS-like active site sequence, and they have currently been reported to exist in bacteria and eukaryotes [11]. Finally, land plants contain (in addition to GRXs species of the above classes) a third class of GRXs (CCMC class), with the sequence CC [M/L] [C/S] in the putative active sites [12,13]. This division in three groups is further complicated by the recent characterization in *Saccharomyces cerevisiae *of three GRXs (Grx6, Grx7 and Grx8) with CSYS, CPYS and CPDC active site motifs [14-16].

Multidomain proteins that contain GRX domains have also been reported in a variety of different organisms [12,13,17-19]. The most studied GRX fusion proteins in eukaryotes are TRX-GRX fusions, containing CGFS class GRXs modules. In these proteins, one to three GRX modules are linked to an N-terminal TRX-like module which does not conserve the WC [G/P]PC active site of functional TRXs. *S. cerevisiae *Grx3 and Grx4 [11] and human GLRX3 (PICOT) [20] are examples of these multidomain GRXs.

Most GRXs are likely to participate in a diversity of processes that require redox-type regulation, although their specific roles and targets in those processes are often unclear. Examples of processes in which CPYC class GRXs are involved in include activation of ribonucleotide reductase, or 3'-phosphoadenylylsulfate reductase, reduction of ascorbate, regulation of the DNA binding activity of nuclear factors, or protection against heavy metals (reviewed in [21]). Examples of processes in which CGFS class GRXs are involved include iron sulfur cluster biogenesis (Grx5), and regulation of cellular iron homeostasis (Grx3 and Grx4) in *S. cerevisiae *[11,18,22], cytochrome-c biogenesis in bacteria [23,24], PROKAR-(lipid membrane) proteins in archaea [25], and regulation of signal transduction pathways in response to external signals (the human PICOT) [20].

In this work we analyze the distribution and evolution of GRX domains in archaea, bacteria and eukaryotes. We study over one thousand proteins, containing at least one GRX domain, from hundreds of different organisms and trace the origin and evolution of the GRX domain within the tree of life. Our phylogenetic analysis suggests that single domain GRX proteins of the CGFS class have evolved through duplication and divergence from one initial gene that was present in the last common ancestor (LCA) of all organisms. The same appears to hold true for single domain GRX proteins of the CPYC class. We predict sets of residues that are likely to be important for protein function in the CGFS and CPYC classes of GRXs. Remarkably, we identify a case of convergent evolution in domain architecture, where two independent recombination events of a TRX domain to a GRX domain are likely to have occurred. We also identify domain combinations that, in the context of GRX domain evolution, appear to function as the supra-domains proposed by Vogel *et al*. [3].

## Results

We have made a systematic identification of GRX domains from the UNIPROT database (information summarized in Table 1). Over 75% of all GRX domains are found in single domain proteins (Table 1). The other 25% are found as a part of a variety of multidomain proteins. For example, GRXs have combined with pyridine nucleotide-disulphide oxidoreductases class-II domains in thioredoxin-glutathione-reductase proteins, or with peptide methionine sulfoxide reductase domains, among others. An interesting case is that of the triple fusion between the GRX domain and frataxin and rhodanese domains. In this case, the frataxin-rhodanese cassette appears to act as a supra domain of recombination [3] in the context of GRX domain evolution, because proteins where the GRX domain recombined exclusively either with the frataxin domain or with the rhodanese domain are not found. In addition to these and other well characterized protein domains, GRXs are also associated with other types of less well characterized protein domains (for example DUF296 domains). Many of these domains are recurrently found in different proteins but have yet to be assigned a specific function. Table 2 details the major domain types found to combine with the different GRX classes. [Additional Files 1, 2 and 3 contain the sequences and alignments for the GRX domains that have been identified in multidomain proteins from the UNIPROT database.] Because of the large number of sequences being analyzed we divided the sequences into smaller sets for a more detailed and accurate analysis. We also analyzed the full set, with results that are similar to the ones described below (data not shown).

**Table 1:** Number of proteins used in this study*.

	GRX Classes					
	
	Archaea		Bacteria		Eukaryotes	
	
	CGFS	CPYC	CGFS	CPYC	CGFS	CPYC
Single domain proteins	5	92	452	543	31	186**

Multidomain proteins	0	28	16	223	94	47

Total number of domains***	5	120	468	766	125	233

* Alignments are provided as supplementary files 1 to 6, where UNIPROT IDs and web links are also included for each protein. Many of the proteins that were found are predicted by homology annotation of fully sequenced genomes. **Out of these, 34 sequences belong to the CCMC class. ***Grand total of 1717 GRX domains.

**Table 2:** Type of major domains associated with GRXs in multidomain proteins.

	GRX Classes					
	
Domain type	Archaea		Bacteria		Eukaryotes	
	
	CGFS	CPYC	CGFS	CPYC	CGFS	CPYC
TRX		X		X	X	
Frataxin- Rhodanese			X			
DUF296				X		
Methionine sulfoxide reductase				X		
Pyridine nucleotide disulfide oxidoreductase		X		X		X
Peroxiredoxin				X		

### GRXs from single domain proteins

**Figure 1:**
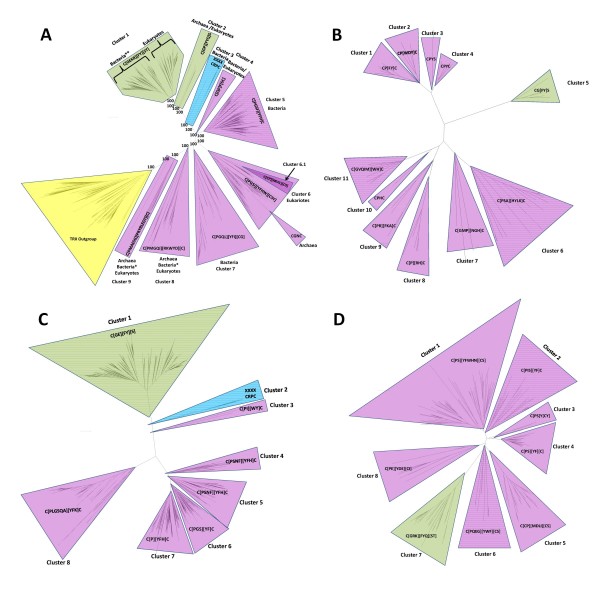
**Condensed phylogenetic tree for single domain GRXs from UNIPROT database**. **Panel A**: Global tree. The outgroup on the lower left of the tree is composed of TRX single domain proteins (in yellow). All major divisions between clusters have bootstrap values of 100%, indicated by the "100" label. The CGFS class of GRXs is depicted on the upper branchs [in green]. Sequences are more homogeneous on this class. The mid-lower branches depict CPYC class GRXs [in mauve]. There is a wider variability in the sequences of this class. The clusters identified with the tag "Bacteria*" include almost all non-proteobacteria GRX domains. These include domains from *Actinobacteria, Deinnococcus, Planktomycetes*, Green sulfur bacteria, Green non sulfur bacteria, *Thermotoga, Aquifaceae*, and *Flavobacteria*. The cluster identified with the tag "Bacteria**" include mostly proteobacteria GRX domains. GRX domains from *Cyanobacteria *and *Spirochaetes *are also present in this cluster. **Panel B**: Condensed phylogenetic tree of single domain GRXs in archaea. **Panel C**: Phylogenetic tree of GRX proteins for bacteria. CGFS class GRXs [Cluster 1] have less variability in their sequence than CPYC class GRXs [other clusters]. Some GRX-like proteins have lost their active site [XXXX in Cluster 2]. **Panel D**: Phylogenetic tree of GRX proteins for eukaryotes. CGFS GRXs [in green] have less variability in their sequence than CPYC GRXS [in mauve]. Nevertheless, the variability in the sequence of the active site for CGFS GRXs is far greater than that found in bacteria [compare to panel C].

Figure 1A shows a condensed phylogenetic tree of single domain GRX proteins. Sequences of TRX domains were used as outgroup for the tree for control purposes. CPYC class and CGFS class GRXs segregate well in the phylogenetic tree. GRXs of CCMC class cluster together, but also within the CPYC class (cluster 6.1 within cluster 6). The CCMC GRXs have only been identified in higher plants (*Embriophita*) and in single domain proteins. This observation suggests that they may be an offspring or a subclass of the CPYC class of GRXs. We also find that the third position in the putative active centre of CCMC GRXs is occupied by an uncharged amino acid that is not necessarily a methionine residue, but can also be a branched amino acid. It is noteworthy that some GRXs that have, overall, higher sequence similarity to GRXs of the CPYC class contain active sites where the final cysteine residue has been replaced by other residues. This is summarized in Figure 1A, where for example in cluster 6, GRXs with CGFS active sites are present. In addition, there are a few proteins with high similarity to GRXs that have completely lost their active site (cluster 3 in Figure 1A, e-values<10^-5^). The function of these proteins is unknown, and this may represent a situation where the GRX domain is being co-opted for a new function that has yet to be characterized.

Within each class of GRXs, different taxa cluster roughly as previously reported (see for example [26-28]). For example, in cluster 1 of Figure 1A bacterial GRXs of the CGFS class group together, and apart from eukaryotic GRXs of the same class. This is consistent with GRXs being present at the LCA of the three kingdoms and is inconsistent with massive HGT of GRX genes between different kingdoms. In fact, CGFS class and CPYC class GRXs appear to have been both present in the LCA of all branches in the tree, because both classes of GRXs cluster apart and differentiation between archaea, bacteria, and eukaryotes is only observed within the clusters shown in Figure 1A.

A group of proteins that have been annotated as GRX-like proteins in fully sequenced genomes, mostly in archaea and eukaryotes but also in bacteria, appear to be somewhere in between TRXs and CPYC class GRXs in terms of sequence similarity (clusters 8 and 9 in Figure 1A). They have more variability in their sequence than the other clusters from Figure 1A, but they are nevertheless similar to other well characterized GRXs, with e-value ≤ 10^-10^. They all contain a GRX-like putative active site sequence. The bacterial sequences in this cluster come from groups that are not typically considered as having GRXs (see caption for Figure 1A).

A detailed analysis of the data also reveals that CPYC class GRXs have been found in all sequenced archaea genomes. However, within Archaea, we were only able to find CGFS class GRXs in *Halobacteriales*. Five sequences of the CGFS class have been found in this group (see see Supplementary Table 1 in Additional File 4, Cluster 2 in Figure 1A and Cluster 4 in Figure 1B). A more detailed analysis of the DNA sequence for the genes that code for these GRXs reveals the following.

a) The highest homology between the *H. salinarium *CGFS class GRX and other non-archaea GRX is to a *Myxococcus xanthus *GRX [see Supplementary Table 1 in Additional File 4].

b) The codon usage of genes is a characteristic that can often be used to identify HGT. This is so because the evolution of an organism leads to an optimization of the codon usage in genes for the specific physiology of that organism [29]. We calculated the average codon usage in the *Myxococcus xanthus *and in the *H. salinarium *genomes [29]. We also calculated the codon usage for the CGFS class *grx *genes from *H. salinarium *and *M. xanthus*. We found that the codon usage in the CGFS class GRXs of *Halobaterium *is similar to the average codon usage in the *Myxococcus *genome. It is also similar to the codon usage in the *Myxococcus *CGFS class *grx *genes.

c) The borders of the CGFS class *grx *gene in *H. salinarium *are homologous to the flanking regions of the transposon gene XAC3504 from the gamma proteobacteria *Xanthomonas axonopodis*.

d) The flanking regions of the CGFS class *grx *genes in archaea could be degenerated palindromes. If this is so, it may indicate the remains of a degenerated transposon. Transposons are responsible for gene mobility within and between genes through HGT.

Taken together, these observations suggest that CGFS class GRXs in archaea may have been the result of one HGT from some proteobacteria ancestor to the *halobacteriales*.

Unlike in archaea, single domain CGFS class and CPYC class GRXs are both widespread in bacteria, as shown in Figure 1C. Both classes of GRXs appear to have existed as such in the LCA of bacteria, because in general CGFS class GRXs cluster together, as do CPYC class GRXs. Interestingly, some GRXs that are, sequence-wise, clearly included in the CPYC class have evolved CGFC putative active centers (cluster 6 in Figure 1C; also see Additional Files 1, 2, 3, 5, 6, and 7), although no CGFS class GRXs with a second cysteine in the active site where found.

Figure 1D shows a phylogenetic tree based on the sequence alignment of eukaryotic single domain GRXs. CGFS class and CPYC class GRXs are also commonly found in eukaryotes. A fraction of CPYC class GRXs have lost the second cysteine residue of their putative active site (GRXs in clusters 3, 5, 6 and 8).

### Sequence analysis of individual GRX domains from an evolutionary and functional perspective

We compared the variability of the CPYC class alignments to that of the CGFS alignments. This variability can be quantified by calculating both, the average positional entropy of the alignment and the normalized mutual information (NMI) between each pair of positions in the alignment (see Methods for details).

**Figure 2:**
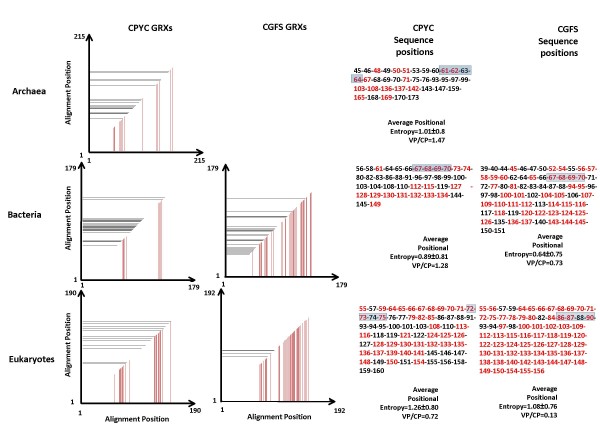
**Functional importance of residues in the different position of the alignment**. Sequences were divided into two classes (CGFS and CPYC). Then, they were further divided into Archaeal, Bacterial and Eukaryotic sequences. Because archaea only have a small number of GRX proteins of the CGFS class, we show the results for archaea GRXs of the CPYC class only. Subsequently, Normalized Mutual Information (NMI) and Position Entropy were calculated for the different alignments. Vertical red lines in the plots of the first and second column indicate positions in the alignment that are highly conserved. Horizontal black lines in the plots of the first and second columns indicate positions in the alignment that have high NMI with at least one other position. Numbers in columns one and two indicate the position in the alignment. First column – CPYC class GRXs. Second column – CGFS class GRXs. Third and fourth columns – Detailed positions in the alignments that are colored, with the consensus residues found in those alignment positions. The putative active centers are shaded in blue. Average entropy per residue is also shown in columns three and four.

The higher the average positional entropy is, the higher the variability per position in the alignment is (see e.g. [30]). Based on this we find that the CPYC class has higher sequence variability than the CGFS class. Although the difference in variability between the two classes may be due to the larger number of sequences that have been identified for the CPYC class, we believe that this is not the case, because the standard deviation of the positional entropy in the two classes is almost the same (approximately 0.8 for the CPYC class and approximately 0.75 for the CGFS class, Figure 2).

The results from NMI profiles support those from the averaged position entropy, although the interpretation of these profiles is more nuanced. On one hand, if two positions in the alignment have a NMI that is very high, one can interpret this result as indicating that any changes in the residue at one of the positions needs to be counterbalanced by a compensatory change in the residue of the other position [30-35]. Thus, the residues in those positions are functionally constrained and functionally important for the protein. On the other hand, positions with residues that are highly conserved will have a NMI with any other position in the alignment that is not significantly different from zero. Therefore, highly conserved positions are also likely to be functionally constrained. All the information regarding conservation and co-variation of residues is summarized in Figure 2. Many of the residues predicted in Figure 2 as being functionally important are known to be involved in the overall function of the GRX domains. For example, residues in the putative active site of both classes of GRXs are marked with a blue transparent rectangle. In the same figure, one can see that CGFS class GRXs have a larger number of positions in the alignment that either have high NMI interaction with other position (black lines and black residues) or are highly conserved (red lines and red residues). If our results are general and our interpretation is correct, GRXs of the CGFS class may require more positions to be constrained, if they are to remain functional.

An alternative explanation for the differences in the variability between CPYC and CGFS GRXs is the following. The larger variability of CPYC GRXs could also be observed if CPYC class GRXs had an earlier origin than CGFS GRXs, because this could have allowed for CPYC GRXs to have evolved for a longer time. Our results suggest that this explanation is unlikely. In Figure 1, the CPYC and CGFS classes of GRXs cluster perfectly apart, and the branching structure of the tree indicates that both classes were already present in the LCA of archaea, bacteria and eukaryotes. This suggests that both proteins may have had, roughly, a similar amount of time to evolve. The DNA sequences of the different domains can be further analyzed in order to assess if the two classes of GRXs have been evolving for significantly different times. It is known that the rate of synonymous mutations in different genes is similar, even when the rate of non-synonymous mutations is quite different [33]. Therefore, by comparing the average percentage of synonymous substitutions per codon between the CGFS class and the CPYC class GRXs in the conserved positions of each multiple alignment, one can obtain additional information regarding whether these proteins have been evolving for approximately the same time. This percentage is similar in both classes of proteins. In addition, we calculated the average ratio of non-synonymous (Ka) over synonymous (Ks) mutations per codon to be <Ka/Ks> = 2.9 for the GRX domains of each of the two classes. This ratio can also be used to estimate the rate of evolution of proteins [36]. Taken together, all these data are consistent with the notion that the difference in the number of highly conserved positions in both classes of GRXs is not due to a difference in the time they had to evolve. The protein and DNA sequence alignments are provided [see Additional Files 1, 2, 3, 5, 6, and 7].

### GRXs domains in multidomain proteins

**Figure 3:**
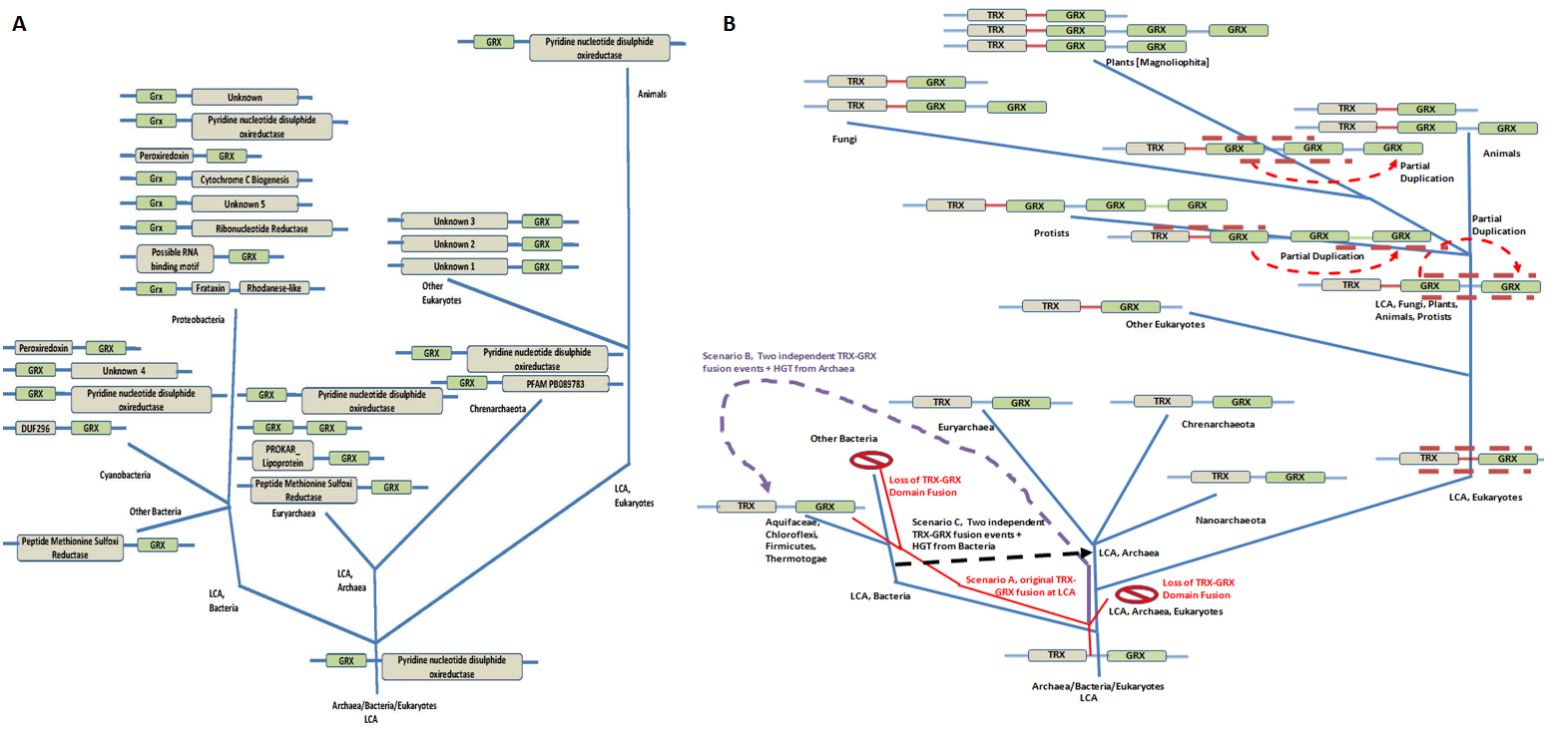
**Schematic representation for the distribution of major groups of multidomain proteins containing GRX domains in the tree of life**. The most ancient fusion appears to be that of GRX domains to pyridine nucleotide disulphide oxireductase domains because it is present in all kingdoms. TRX-GRX fusions may also have been present in the LCA (Latest Common Ancestor) of all three branches. Proteins standing at the end of a node are the ones identified in current organisms. Proteins standing on top of the branches of the tree and surrounded by upper and under lines that are dashed suggest when that type of protein may have first originated. All domains identified as unknown in the figure have not been identified in any of the available domain databases. All domains identified as PFAM in the figure have been identified in PFAM as associated to proteins of unknown function. Panel **A **– Non- TRX-GRX fusion proteins. Panel **B **– TRX-GRX fusion proteins. TRX-GRX fusion proteins exist in all three major branches of the tree of life. Duplication of the GRX domain appears to have occurred early in the eukaryotic life history. Some TRX-GRX proteins appear to have undergone two consecutive partial duplication/recombination events of the GRX domain after the initial TRX-GRX fusion. Distribution of TRX-GRX fusion proteins may result from a) multiple independent deletions of the TRX-GRX fusion in different bacterial branches and in the eukaryotic branch, followed by a new TRX-GRX fusion event in the eukaryotes (Scenario A, red lines), b) Two independent TRX-GRX fusion events, one in archaea and one in eukaryotes followed by horizontal gene transfer from archaea to bacteria (Scenario B, dashed mauve line), or c) TRX-GRX fusion originating in the ancestor of some bacterial lineages, followed by horizontal gene transfer to the ancestor of Archaea and an independent TRX-GRX fusion event in eukaryotes (Scenario C, dashed black line). See Discussion for further details.

**Figure 4:**
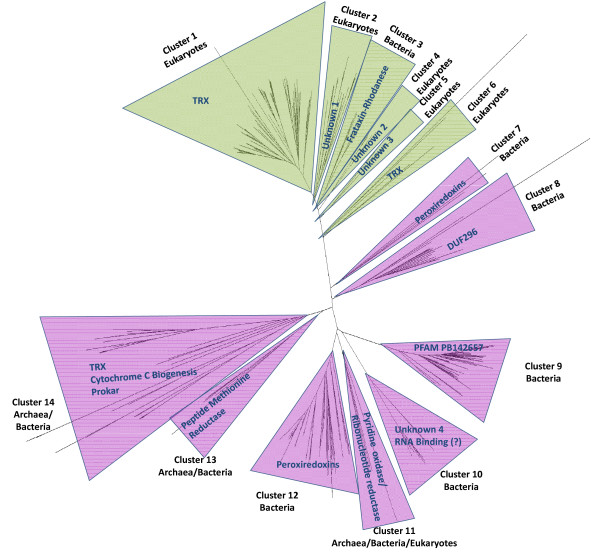
**Phylogenetic tree of GRX domains from multidomain proteins**. CGFS class GRX domains [in green] have less variability in their sequence than CPYC class GRX domains [in mauve]. Note that GRX domains from NrdH-like proteins cluster closer to GRX domains from TRX-GRX fusion proteins, and within the CPYC class. This is consistent with previous results from biochemical assays and sequence analysis that suggest NrdH-like proteins to cluster between TRXs and GRXs [51].

As stated above, and summarized in Table 2 and Figure 3, multidomain proteins that contain GRX domains are widespread. To more accurately analyze these GRX domains we isolated their sequence from the multidomain proteins, as described in the Methods section. The GRX domains were then aligned using MEGA4. The multiple alignment was used to build a phylogenetic tree. A condensed version of this tree is shown in Figure 4. It is striking that, with few exceptions, all GRX domains from proteins containing a specific type of domain combination are, sequence-wise, closely related amongst themselves. They cluster together in the phylogenetic tree and apart from GRX domains extracted from other types of proteins. The simplest interpretation of the data presented in Figure 4 is that a specific type of GRX-containing multidomain proteins is descendent from an original gene fusion event. This event is likely to have occurred before the LCA of all organisms containing the relevant type of multidomain protein (Figure 3). If independent gene fusion events between different GRX domains and a given protein domain occurred at different stages of the evolutionary process, then one would not expect the isolated GRX domains from these fusion proteins to cluster together and apart from all other GRX domains.

Only the GRX-pyridine nucleotide disulphide oxireductase fusion and the TRX-GRX fusion are found in all three branches of the tree of life. All other multidomain proteins that contain a GRX domain are found only in specific branches of that tree (Figure 3A). For example, peroxiredoxin-GRX fusions are found so far only in proteobacteria. TRX-GRX multidomain proteins appear to have a more complex history. In eukaryotes, all GRX domains in TRX-GRX fusion proteins are of the CGFS class. In contrast, they are of the CPYC class in bacteria and archaea. This suggests that two independent recombination events took place to form the original TRX-GRX fusion proteins. In addition, TRX-GRX fusion proteins have undergone further domain shuffling in eukaryotes. An analysis of the phylogenetic tree in Figure 4 suggests a duplication of the GRX domain to have occurred in TRX-GRX domains, forming TRX-GRX-GRX proteins before fungi, plants and animals have separated. A more recent duplication-recombination event of the GRX domain is present in TRX-GRX-GRX-GRX proteins found in plants and in some protists. Based on how the different GRX domains cluster in the phylogenetic tree, we cannot exclude that the duplication events in plants and protists are independent. However, the bootstrap of the tree for the separate clustering of GRX domains from protists and plants is lower than 50% (Figure 4, clusters 1 and 6). This means that only in less than 50% of the trees built using bootstrap do we find separation of the protists cluster and the plant cluster. Such a fact suggests that this final recombination event may have taken place either a) in a common ancestor of plants and protists or b) in one of the branches, followed by HGT to the other branch. Nevertheless, it is of interest that the TRX-GRX multidomain proteins may have gone through several cases of convergent evolution of domain architecture.

In eukaryotes, most GRX domains in multidomain proteins are of the CGFS class (Table 2 and Figure 4). The exceptions are the CPYC class GRX domains recombined with pyrimidine disulphide oxireductase domains. In contrast, in prokaryotes, most GRX domains in multidomain proteins are of the CPYC class (Table 2 and Figure 4). GRX- containing protein architectures that are specific to bacteria are:

a) Proteins with a domain structure of the type peroxiredoxin-GRX, with the GRX domain being of the CPYC class. Peroxiredoxins are thiol dependent peroxidases involved in cell protection against oxidative stress.

b) Proteins with a domain structure DUF296-GRX. DUF296 is a domain of unknown function that contains what appears to be a zinc finger like motif [37]. This suggests that these proteins may be involved in DNA binding, probably acting in regulation of gene expression. The GRX domain is of the CPYC class.

c) Proteins with a domain structure frataxin-rhodanese-GRX. Single domain frataxin proteins are involved in iron storage and metabolism and in iron-sulfur cluster biogenesis [22,38]. The acidic aspartate and glutamate residues that are responsible for iron binding in the frataxin protein are, for the most part, conserved in the fusion proteins, suggesting that these domains may remain at least partially functional. On the other hand, proteins containing rhodanese domains participate in sulfur metabolism [39]. Again, the cysteine and glycine residues of the rhodanese active center are for the most part conserved in the fusion proteins, suggesting that the rhodanese domains may remain at least partially functional. Considering that *S. cerevisiae *Grx5 is implicated in mitochondrial maturation of iron-sulfur clusters [18], this suggests a connection between the complex frataxin-rhodanese-GRX proteins and iron-sulfur (cluster) metabolism.

## Discussion

GRX domains are a part of the thioredoxin-fold superfamily. This superfamily includes domains of several classes, such as TRXs, PRXs (Peroxiredoxins), GSTs (glutathione S-transferase), GRXs, among others. Each of these domain classes is involved in different types of redox reactions that follow dissimilar mechanisms. In this work we analyzed GRX protein domains from a wide number of organisms in the tree of life. We confirm that GRXs are widespread over the three major kingdoms of life, both as individual proteins and as domains of larger proteins. We detect no signal of extensive HGT of GRXs among the different taxa, except for GRXs of the CGFS class between bacteria and *Halobacteriales *(archaea). Because GRXs are present in archaea, bacteria and eukaryotes, our results suggest that both the CPYC class and the CGFS class of GRXs were already present in the LCA. This inference follows from accepting the proposal that bacteria diverged first, followed by the divergence between archaea and eukaryotes [40]. If one accepts this view, it follows that archaea and many bacterial branches have lost GRXs of the CGFS class somewhere in the early stages of their evolution.

Our analysis of the available data leads us to speculate about the origin of GRXs. One should recall that GRXs and TRXs belong to the same protein super family because they have a common structural fold. In addition, when one uses BLAST to search for GRX sequence homologues in the UNIPROT database, we find TRX domains as the closest relatives of GRX domains (data not shown). Taking these two facts together, one could make the case that both types of domains may have originated from the same common ancestor gene. If this is so, then the clusters in Figure 1A may be indicative of how TRXs and GRXs have diverged. Initially, a TRX ancestor may have been duplicated well before the LCA. After divergence, this duplicated TRX ended up becoming the ancestor of the GRX domain proteins. At this stage, further duplication events, either of the GRX domain or of the TRX domain may have led to the formation of the different GRX classes. The branching structure of the tree shown in Figure 1A, together with the active site information that is also shown in that figure, leads us to further speculate that sequences from clusters 8 and 9 may be fossil sequences that are remnants of intermediate steps in the divergence of the original GRX domain towards current day GRXs of the CGFS and CPYC classes. If one accepts this picture, then it follows that the original ancestral of all GRXs is of the dithiolic type (that is with a CXXC active center) and that CGFS class GRXs would be more recent than CPYC class GRXs, even though both may have been present at the LCA of archaea, bacteria and eukaryotes. Consistently with this view, CPYC class GRXs sometimes lose the second cysteine in their active site, while no GRX with two cysteines in its active site was found in clusters with CGFS class GRXs.

If the picture described in the previous paragraph is correct, CGFS-class GRXs would be the more specialized GRXs, which could entail having stronger functional constraints against mutations in their sequence. Our analysis finds that GRXs of the CGFS class indeed have a higher percentage of conserved residues than those of the CPYC class and that the average residue variability, over the stretches of the proteins where residue conservation is low, is similar in the CGFS and CPYC classes. This further supports the notion that the lower sequence diversity in the CGFS class may be due to this class having functional constraints on a larger set of residues than the CPYC class. A deeper understanding of the catalytic mechanisms of the two GRX classes and of the protein dynamics during catalysis would be necessary if one is to confirm this speculation and explain it in a mechanistically rational way. Nevertheless, enzymatic studies already indicate that the mechanism of action of both types of GRXs is different [11,17].

GRX modules as parts of multidomain proteins are widespread. Figure 3 summarizes the broad phylogenetic distribution of these proteins. The only GRX- containing multidomain architecture that is common to the three kingdoms [with the same class of GRX module] is that found in GRX-pyridine nucleotide disulfide oxireductase proteins. This suggests that this type of fusion protein may be ancestral to the divergence between the three kingdoms of life.

The question arises on the biological advantages of such domain fusions. In the case of *S. cerevisiae *Grx3, the TRX domain seems to be required for the nuclear targeting of the molecule [41], without having enzyme activity as redoxin. However, in other cases both domains might retain the original enzyme activities and be involved in different stages of the same biological process. Domain combinations involving redoxin modules could be an evolutionary strategy to incorporate into a single peptide the enzymatic redox activity and the target of such redox control. The case of the fusions between GRX domains of the CGFS class and frataxin-rhodanese domains could be an example of this situation. The participation of CGFS class GRXs in the synthesis of iron-sulfur clusters is well known to occur in eukaryotes [18], although such participation has not been demonstrated in bacteria up to date.

An interesting observation is that while TRX-GRX proteins in eukaryotes have GRX modules of the CGFS class, TRX-GRX proteins in bacteria and archaea have GRX modules of the CPYC class. This is consistent with the fusion between TRX and GRX modules having occurred independently in evolution at least twice. Furthermore, the TRX-GRX fusion proteins in eukaryotes have undergone further evolution, giving rise to proteins where the GRX module has been duplicated once or twice. The observations are consistent with the following scenarios (Figure 3B):

**a) **Scenario A: TRX-GRX fusion, with GRX belonging to the CPYC class, present at the LCA of the three kingdoms. TRX-GRX fusion lost in the eukaryotic branch, with a subsequent, independent, new TRX-GRX fusion event occurring in eukaryotes, this time with a GRX of the CGFS class.

**b) **Scenario B: TRX-GRX fusion, with GRX belonging to the CPYC class, occurring in ancestral archaea, followed by HGT of this protein to the common ancestral of several bacterial lineages. Independent TRX-GRX fusion event occurring in eukaryotes, this time with a GRX of the CGFS class.

**c) **Scenario C: TRX-GRX fusion, with GRX belonging to the CPYC class, occurring in ancestral bacteria, followed by horizontal gene transfer of this protein to the common ancestral of archaea. Independent TRX-GRX fusion event occurring in eukaryotes, this time with a GRX of the CGFS class.

We do not have enough data to distinguish between the three hypotheses. However, Occam's razor suggests that scenario b) may be the most likley, because that is the scenario where a smaller number of events would have had to occur. This would point to the TRX-GRX hybrid proteins as an interesting example of convergent evolution in domain architecture.

## Conclusion

In this study we trace the origin of glutaredoxins to the LCA of archaea, bacteria and eukaryotes. We propose probable patterns of evolution for the different GRX classes and trace the origin and evolution of recombination events between the GRX domain and other protein domains. We find at an interesting case of convergent evolution in the domain architecture of TRX-GRX proteins.

## Methods

### Retrieval and curation of GRX sequences

We used the UNIPROT database [42] to retrieve all sequences analyzed in this study. We downloaded the sequences of all proteins containing a GRX domain in FASTA format [43]. These proteins have been identified using PSI-BLAST [44]. As query sequences for the BLAST search we have used all GRX domain sequences from *S. cerevisiae*, *Escherichia coli *and *Halobacterium salinarium*. All cDNA sequences corresponding to the UNIPROT entries were retrieved from Genebank [45]. Once all GRX domains were identified, we analyzed them in isolation.

Because a large number of very similar sequences were identified, we used the "Decrease Redundancy" program at SWISSPROT [46] to reduce the number of sequences to analyze. We set the algorithm to eliminate all sequences that had more than 90% identity to the sequence that was retained in the set. We then cross-checked the eliminated sequences and re-introduced any sequence from an organism for which no close relative with a similar sequence had been retained. This was done using a local PERL script. A summary statistics of the analyzed sequences is shown in Table 1.

### Sequence alignments and building of phylogenetic trees

MEGA4 [47] was used to build sequence alignments and the phylogenetic trees shown in Figure 1. All trees presented here were built using a minimum evolution model and bootstrapped one thousand times. Dendroscope [48] was used for tree analysis and representation. TRX domain sequences were used for control purposes as an outgroup for the tree building alignments. As a control we have also build trees from the same set of sequence using neighbor joining methods, and maximum likelihood methods. The resulting trees were similar to those shown in the Figures 1 and 4.

### Domain identification in multidomain proteins

Multidomain proteins were identified using the Domain Fishing server [49] and PROSITE [50]. The GRX domains as identified by PROSITE were then manually excised from the longer proteins. Whenever a domain was exclusively identified by the Domain Fishing Server, that domain was then manually excised from the longer proteins.

### Analysis of residue conservation in alignments

Mutual information between different position pairs in an alignment is indicative of how much the residue variation in one position constrains the residue variation in the other position. It was calculated using the formula , where *f(AA [i, m]) *represents the relative frequency of amino acid of type *m *in position *i *of the alignment and *f(AA [i, m], AA [j, k]) *represents the join relative frequency of that amino acid in position *m *and of amino acid of type *k *in position *j *of the alignment. The higher the covariance between any two positions, the higher the Mutual Information between those two positions will be. For representation purposes in Figure 2, we define normalized mutual information as , where *Max*{*MI*(*alignment*)} is the maximum MI between any two positions in the alignment. The higher *NMI *(*i, j*) is, the higher the covariance between positions *i *and *j *will be.

Positional entropy measures how high or low is the variability at a given alignment position. Positional entropy for position *k *in an alignment was calculated using the formula , where *P*_*jk *_is the frequency of amino acid *j *in alignment position *k*.

The ratio Ka/Ks, where Ka stands for non-synonymous substitutions and Ks stands for synonymous in the DNA can be used to compare how fast different proteins are evolving [36]. We use this ratio to compare the rate of evolution between CPYC class GRXs and CGFS class GRXs. All calculations were done using *Mathematica*.

## Abbreviations

GRX: Glutaredoxin; HGT: horizontal gene transfer; LCA: Last common ancestor; MI: Mutual information; NMI: Normalized mutual information; TRX: Thioredoxin.

## Authors' Information

RA and EH conceived and designed the study. RA and EV executed the study. RA, EV, AS and EH carried out data analysis, wrote the manuscript, and approved the final version.

## Supplementary Material

Additional File 1 **GRX sequences from Archaea. UNIPROT links to the GRX sequences from archaea, together with protein and DNA alignments and links to other sequences that have over 90% sequence identity.** Click here for file

Additional File 2 **GRX sequences from bacteria.** UNIPROT links to the GRX sequences from bacteria, together with protein and DNA alignments and links to other sequences that have over 90% sequence identity. Click here for file

Additional File 3 **GRX sequences from eukaryotes.** UNIPROT links to the GRX sequences from eukaryotes, together with protein and DNA alignments and links to other sequences that have over 90% sequence identity. Click here for file

Additional File 4 **Analysis of CGFS-GRXs sequences from archaea.** Contains the supplementary table I, with compiled information about CGFS class GRXs in archaea. Click here for file

Additional File 5 **Sequences of GRX domains from multidomain proteins. **UNIPROT links to the GRX sequences from multidomain proteins, together with protein and DNA alignments and links to other sequences that have over 90% sequence identity. Click here for file

Additional File 6 **Aligned sequences of CPYC class GRX domains from multidomain proteins.** Alignment used to build the phylogenetic trees. Click here for file

Additional File 7 **Aligned sequences of CGFS-class GRX domains from multidomain proteins.** Alignment used to build the phylogenetic trees. Click here for file
