# Supplementary material for: Evolution based on domain combinations: the case of glutaredoxins
Source: BMC Evol Biol. 2009 Mar 25;9:66. doi: 10.1186/1471-2148-9-66 (PMC2679010; doi:10.1186/1471-2148-9-66)
Supplement: Additional File 1 — GRX sequences from Archaea. UNIPROT links to the GRX sequences from archaea, together with protein and DNA alignments and links to other sequences that have over 90% sequence identity. [file 1471-2148-9-66-S1.htm]

Supplementary file 1: Archaea


#### Alignment for Archaea GRXs

|  |  |  |
| --- | --- | --- |
| UNIPROT accession number | UNIPROT 90\% similarity cluster | Aligned Sequence |
| Q8U2V4 | UniRef cluster | ----------------MKLKALFLLLALGSLIVPRIEAEEFESSKVHFYMYGA--YWCPHCRNVKESIKEH-FGEETL---TYYEIEGVE-----HNKNKFEELYLLTGISGIPATAIFYEGEIYAVIEGEFDVSK------TWQIITEAKNQGGVILVTSGKAYILKWDSQEVQKLKEIFLSGKVNETQKKAEEKSICGPASVLLLSMGAIYLRKRNYSTEVSSNV |
| Q58001 | UniRef cluster | ----------------------------------------MVRVMVVIRIFGT--G-CPKCNQTYENVKKA-VEELG------IDAEIVKVT---DVNEIAEW-VFVTPGVAFD------DVIVFEG----KIPSVE----EIKEELKSYLEGK------------------------------------------------------------------------- |
| A0B723 | UniRef cluster | ------------------------------------------MRCLTCRIYTT--PDCPRCEQLKAFLES--KGVS-------FEEVDMTTAEALTELRVNGVFTMSAPVLEMD-GRFYTTGDLFDGDR--LKELK------L------------------------------------------------------------------------------------ |
| A0B7M2 | UniRef cluster | ----------------------------------MGKVHVDGHDAGAVTLYAL--STCMWCKKTRDLLSK--LGVS-------YDYVYVDLTSGAERAELIDELRKFNPDLTFP-TLVANG-RVIIG----FKEKE------IMSALGAL----------------------------------------------------------------------------- |
| A3CX10 | UniRef cluster | -------------------------------------------MAVKVISFYQ--EGCMGCKEQTPILLEA-AADLG------IEIEEIDAVK--NPEYIQKYNLRVTPTTLVLD--GDEIRERMEG---LVHSED------LEAAIRRHLPEPLPR---------------------------------------------------------------------- |
| A3CYC6 | UniRef cluster | ------------------------------------MQRVPGVDRGRVVLYAL--STCGWCARTKELLTD--LGVG-------FSYVYVDLLAGEERDHVVREVERWNPQVSFP-TVVIDEAKVIVG----FREEE------IREAVGARHSE-------------------------------------------------------------------------- |
| A4G0T6 | UniRef cluster | --------------------------------------------MVKVEVFTS--PMCPHCPAAKRVVDEV-AKEIEG-----LEVVHINVM--DHPEKAAELGIMAVPTVAIN------GEIKFVG----APTKD-----ALLAELKK------------------------------------------------------------------------------ |
| A5ULG5 | UniRef cluster | -------------------------------------------MVVKVEVFTS--DSCPHCPAAVNVANEA-KEVLGDA----ADIIVCNIASEENRQKAIGLGIMAVPTIAIN------DEVAFVG----APALD-----ELVNKVKSLI---------------------------------------------------------------------------- |
| A6UQS2 | UniRef cluster | --------------------------------------------MVKVEVFTS--PMCPHCPAAKRVVEEV-AKEVSG-----VEIVLVNVM--DNPEKAAELGIMAVPTIAIN------GEIKFVG----APSKD-----ALRVELTK------------------------------------------------------------------------------ |
| A6UTU5 | UniRef cluster | ---------------------------------------------MLVEVITS--PQCPHCPTAKKVVEEV-VKKVSCDD---IEVKYIDVT--EDPGTVEKYNIMTVPTIVIN------GEIAFLG----APSIE-----QLENYLRENLSR-------------------------------------------------------------------------- |
| A6UUB7 | UniRef cluster | --------------------------------------------MVKIEVFTS--PQCPHCPAAKRAVEEV-LAEIKGD----FEVEHVNVM--EEPKRAADYGIMAVPTIVID------GEVAFMG----APQKE-----QLKEKLLSL----------------------------------------------------------------------------- |
| A6VHP9 | UniRef cluster | --------------------------------------------MVKVEVFTS--PMCPHCPAAKRVVDEV-AKEIDG-----LEIVHINVM--EHPEKAAELGIMAVPTVAIN------GEVKFVG----APTKD-----ALIAELKK------------------------------------------------------------------------------ |
| A7D0E3 | UniRef cluster | ------------------------------------MSETDASETVPVTVYTR--EDCSLCVAARETIEAVAEGLDVT-----IDLDMVDVD---EDPELAEEYGERVPYVLVD------GHPAFKY---EIDERE------LRLKLLAAS---------------------------------------------------------------------------- |
| A7D188 | UniRef cluster | ---------------------------------------------MAIQLYAL--DGCPWCEKVSDALDE--AGVA-------YETEWVD-----ALHSDRSEVKRISGQRGVP---VLIDEERGVT-M--SESAN------ILEYVERSLV--------------------------------------------------------------------------- |
| A7D3I3 | UniRef cluster | --------------------MTFQPETDAEP-DEIVARVEETIADNDVVLFMKGNRLMPQCGYSKRAVELI-GQHV-------EEFETVDVLP--ALPHYREALESHSGWETIP---QTFVNGEFIGGSDVLAELD------ERGELAAELGAE------------------------------------------------------------------------- |
| A7D485 | UniRef cluster | ------------------------------------------MSESPITLYRL--QACPYCERVVRMLNE--LDLE-------YRSRYVE-----PMHSERNVVKRVSGARSVP---AIVDPETGVT-M--SESAN------IVEYLESTYAEG-----GA------------------------------------------------------------------ |
| A7D4N1 | UniRef cluster | ------------------------------------------MVMTDLTLYEL--EGCPYCAKVKAKLAD--LDLE-------YESVMVP-----RSHGKRTEVEEISGQTGVP---VLVDEEHGIDAM--PESDD------IVEYLEETYGDAS------------------------------------------------------------------------ |
| A7I6I9 | UniRef cluster | -----------------------------------MPTHVNGKNRGSVMLYAL--STCQWCHKTKVLLEE--LGIA-------FDYEYVDLLEGGEQDRVMNAVEHWNPSGSFP-TLVIDNKRAVVG----FREHE------IREALAV------------------------------------------------------------------------------ |
| A7I914 | UniRef cluster | -----------------------------------------MPESVPLIVYSL--EMCPHCEMLKKFLKN--KGYA-------YSEKDLSTAEALTELRVNGVFVNEAPVLQRG-EDFYTTDDIFPAGS--LDEKK------LGAILSGA----------------------------------------------------------------------------- |
| A8TDN4 | UniRef cluster | --------------------------------------------MVKVEVFSS--PSCPHCPAAKRVVEQV-VKEMSD-----IEVIHINVM--EHPEKAIELGIMAVPAIAID------GDVVFVG----APAEE-----DFKNKLLEKINAIE------------------------------------------------------------------------ |
| A9A5N6 | UniRef cluster | -------------------MEKIIVFVVPAVNGHLKIIQSNLKMNHKIEILTT--PSCGNCKVVEAMLDE--MNIK-------YTVIDVTE----KPEYLEKYPIFTAPGVVID------DKLVFTG----IPKKH-----ELLEKLSA------------------------------------------------------------------------------ |
| A9A928 | UniRef cluster | --------------------------------------------MVKVEVFTS--PMCPHCPAAKRVVDEV-AKEIDG-----LEVVHINVM--EHPEKAAELGIMAVPTVAIN------GEVKFVG----APTKD-----ALLAELKK------------------------------------------------------------------------------ |
| B0R3E0 | UniRef cluster | --------------------MAFDPLDSSMAQDAVDDIVDAAIENNEVVLFMKGTPAAPQCGFSERAIRLI-SDYR-------PDVHTVDVLQ--STDEFRAALERHSGWETTP---QAFVDGSFVGGSDILAELA------DRGDLADELNADGVDTTADDAALD-ADIDAPF----------------------------------------------------- |
| B0R4N8 | UniRef cluster | -----------------------------------MRVSTVPIGMSQVTFYAR--EDCPYSQKVRSKLDA--LDVA-------YEETLVP-----DAHTDRTTVEDVTGQTGVP---VVIDDHMEPSFL--ADTQE------IITHLETQYA--------------------------------------------------------------------------- |
| B0R5V0 | UniRef cluster | ---------------------------------------------MTLTLYAL--DGCPDCESVIDTLAA--DDID-------HETVHVD-----ARHSARNAVKRASGQRSVP---VLVDDDRGVV-M--ADSQR------IQTYAAVTLA--------------------------------------------------------------------------- |
| B0R733 | UniRef cluster | --------------------------------------------MS-LELYKL--PGCPYCAKVETKLDE--LGLD-------YVEHEVP-----SSHSDRDAVESVSGQTGVP---VLVDPDHDIDGM--PESDD------IVAHLEQHYAE-------------------------------------------------------------------------- |
| B0R7K4 | UniRef cluster | -------------------------------MSAPAAPPRPPHSDAHITLYRL--QACPFCERVVNRLEE--LGLA-------YQSRYVE-----PMHSERDAVKRIVGARTVP---AIVDDETGVA-M--AESAN------IVAYLDATYGGG-------E----------------------------------------------------------------- |
| Q0W0R7 | UniRef cluster | --------------------------------------------MVKISMYTL--STCPFCRKTKKYFRD--RGIQ-------FDYIDYDTADEKEQERIAADMMKHTDHIAFP--FVRIGDTVVIG----FNPDR------YELLLKSEKSPATAS---------------------------------------------------------------------- |
| Q0W1K8 | UniRef cluster | ------------------------------------------MMAAKVRVFSQ--PTCPACNDLKEYLKK--KRVE-------FEDMDIT-----ASKDAFNELTKVYKVRVTP-LLVM-GDKKLIG----FDPAE------VDKLL-------AEN--K------------------------------------------------------------------- |
| Q0W1U0 | UniRef cluster | --------------------------------------------MPDVKIYTQ--PSCGYCNQLKEYLQK--HNIS-------FEDKDIT-----KDRTAMDELIHKYKVRATP-LLVY-GDKTIVG----FNPDE------INKVLGSEAGQQAEAGAK------------------------------------------------------------------- |
| Q0W246 | UniRef cluster | ----------------------------------MSMEHVPGRKNGEVVLYAL--STCVWCGKTRELLTE--LGVE-------YSYVYVDLLSGAEFDRTVAEMARWNKSRSFP-TLVINNSKVIVG----FREDE------IREVLA------------------------------------------------------------------------------- |
| Q0W408 | UniRef cluster | ---------------------------------------------MKLQVYGT--G-CAKCSMLEKAAKDA-VKELG------VSAEVVKVS---DIDEIVEAGILATPGLAVD------GEVKSMG----RVPSGD----EIKKWIKAKM---------------------------------------------------------------------------- |
| Q12U79 | UniRef cluster | --------------------------------------------MSDIIIYTT--ETCPKCVQLKKVLKS--NDVI-------FTEADMSTPESLTELRLNGVFTVTAPVLQID-DDFLTYEELFNSDG--VNLGS------LKSIL-------------------------------------------------------------------------------- |
| Q18E41 | UniRef cluster | -------------------------------MSADS-----TNSDVSITVYRL--EACPFCERVIRLLEE--LELS-------YHSRFVE-----PMHSDRDVVKRISGKRTVP---ALVDTNTGVV-M--SESGN------IVTYLEQTYGSD--STTSMEKPVEGV----------------------------------------------------------- |
| Q18FD8 | UniRef cluster | ---------------------------------------------MTITLYAL--DGCPYCETVHDALTD--AGVE-------YTTIWVD-----ALHSERDEVRRVSGQRGVP---VLVDEARGVT-M--CESTN------IETYVQQTLAGE------------------------------------------------------------------------- |
| Q18FU5 | UniRef cluster | -------------------------------------------MMSNLTLYEL--SGCPYCAKVIDKLDE--LGLE-------YDSVSVP-----RAHSERTEVESISNQTGVP---VLVDEANDVSGM--PESDD------IVAYLEKTYAN-------------------------------------------------------------------------- |
| Q18JL3 | UniRef cluster | --------------------MTFQPGSELSD-EEITTRVNETIADNDIALFMKGNRLMPQCGYSQRAVNLI-SQHV-------EEFETIDVLP--ALDQYRSALEDHSGWETIP---QTYVDGEFIGGSDILAELD------ERGELAEKLGFDD------------------------------------------------------------------------ |
| Q64EA9 | UniRef cluster | ---------------------------------------------MKILLFVS--SQCPHCPAAEAIVNRV-VPDYSG---YGLSLNKIRMKTKAGKKRSSMYHVRAIPTTLILDD-NGHELKRMVG---VMREDT------LRASIEKLLGLRESVLSRIFGRKK------------------------------------------------------------- |
| O28736 | UniRef cluster | --------------------------------------------MAEVLMYGL--STCPHCKRTLEFLKR--EGVD-------FEVIWIDKLEGEERKKVIEKVHSISGSYSVP--VVVKGDKHVLG----YNEEK------LKELIRG------------------------------------------------------------------------------ |
| Q2FQW9 | UniRef cluster | ------------------------------------------------MLYAL--STCIHCKQTRKLLDE--LKVA-------YDYLYVDQLSRSEMDEVLKEMEKVNPRGSFP-TLVINNNKVIVG----SRLDE------IREALLS------------------------------------------------------------------------------ |
| Q3IPI1 | UniRef cluster | ---------------------------------------------MTLTLYRL--EGCPYCEFVVDTLED--LPLD-------FESVWVE-----GLHSKRNEVHEITGQRQVP---ALVDDAHGVS-M--SQSAR------IIEYLETTYGDATSPDEVELDF--------------------------------------------------------------- |
| Q3IRW7 | UniRef cluster | --------------------MTFDPNADAADPENVQERVDTLIEDNEVVLFMKGNELMPQCGYSKKALQLL-TKYR-------DDVVTEDVLD--ALDAYRAALESHSGWETIP---QTFVDGEFIGGSDILEELD------NRGELHDELGVDADDVDADDAGTDDAGAEAPF----------------------------------------------------- |
| Q3IST6 | UniRef cluster | ------------------------------------------MSEPDITLYRL--QACPFCERVVAVLDE--LGLA-------YRSRFVE-----ARHSRRDVVKRLTGARTVP---ALVDDRTGVT-M--SESAN------IVEYLRATYGDG---GGGAE----------------------------------------------------------------- |
| Q3IT18 | UniRef cluster | --------------------------------------------MANITLYEL--AGCPYCKKVIDKLDE--LGLD-------YDSIEVP-----RSHSDRTEVEDVSGQTGVP---VIIDEEHGIEGM--PESDD------IVEYLEETYGS-------------------------------------------------------------------------- |
| Q469U3 | UniRef cluster | --------------------------MDVSTLNKEKGDHISGIDRGKVVMYGL--STCVWCKRTKKLLTD--LGVD-------FDFIYVDRLEGKEENQAVEEVRHFNPSTSFP-TTVINGEKAIVG----FKEKE------IREALGFQG---------------------------------------------------------------------------- |
| Q46BU0 | UniRef cluster | -----------------------MYLQLEKTKNVQNDKTKHEVKKMKIEILGT--G-CPKCKKTKEIIEKV-LKQTG------VEAEVIKVE---DVEKILNYGVMVTPAVAID------GEVKVAG----KVPDEK----DIRKWVTQ------------------------------------------------------------------------------ |
| Q46DN6 | UniRef cluster | --------------------------------------------MAKIIIYTT--ESCPKCNKLKNFLEA--HSVA-------FEVEDMSTPEALTELRFNGVFTVTAPVLQIN-DTFLTHEELFSGGE--VNPEK------IQEVL-------------------------------------------------------------------------------- |
| Q46FD7 | UniRef cluster | ----------------MCSSSTKLQGTKRKKLKETESTIELGERVAKVSMYTI--SNCPVCRKTKEFFRA--RGIP-------FDFIDYDLASESEQNKIAAEMMEGTGNIGFP--FVRIGEVVVIG----FNPER------FKQLLKSEEL--------------------------------------------------------------------------- |
| Q5UZB3 | UniRef cluster | ------------------------------------------MSESDITLYRL--QACPFCERVVRKLNE--YGLD-------YQSRFVE-----PMHADRDVVKRLSGKRTVP---AIVDESTGVT-M--SESAN------IVAYLERTYGEGEETAGGAA----------------------------------------------------------------- |
| Q5V5M0 | UniRef cluster | ---------------------------------------------MSLTLYQL--DGCPYCEKVADRLDE--LGIE-------YDSVWVE-----ALHSKRDEVKRVSGQRGVP---VLVDDDRGVT-M--AESDR------ILELIETTYAPEAQSA--------------------------------------------------------------------- |
| Q8PS17 | UniRef cluster | --------------------------MNLFGQ-KDRGNHVSGVDRGKVIMYGL--STCVWCKKTKKLLTD--LGVD-------FDYVYVDRLEGKEEEEAVEEVRRFNPSVSFP-TTIINDEKAIVG----FKEKE------IRESLGF------------------------------------------------------------------------------ |
| Q8PUT2 | UniRef cluster | ---------------------------------------------MKIEILGT--G-CPKCKKTKETIEKV-LKQTG------VEAEVIKVE---DIEKIMSYGVMVTPAVVID------GEVKLAG----KVPDEK----DVRKWMI------------------------------------------------------------------------------- |
| Q8PX54 | UniRef cluster | --------------------------------------------MAKVIVYTT--ERCPKCNKLKKFLEA--NAVP-------FEVADMSTPEALTELRFNGVFTVTAPVLQINNDTFLTHDELFRGDE--VDSEK------LLGIV-------------------------------------------------------------------------------- |
| Q8TQ93 | UniRef cluster | --------------------------MNIFSKDTDKGKHVSGIDRGKIVMYGL--STCVWCKKTKKLLTD--LGVD-------FEYVFVDLLEEEEKSNAIKQVSRFNPSVSFP-TTILNDEKAIVG----FKEKQ------IREALGF------------------------------------------------------------------------------ |
| Q8U0X4 | UniRef cluster | --------------------MIRRKKMLELMKRMGIIEERKPKKKVIIEVITA--PGCPYCPIAVQMAREI-EKKYPG-----VIVRELSVATPEGRRKAMEHNILGTPTILIN------NRVEFIG----VPRFI-----DFERRVRELLS--------------------------------------------------------------------------- |
| Q9HN01 | UniRef cluster | -------------------------------MSAPAAPPRPPHSDAHITLYRL--QACPFCERVVNRLEE--LGLA-------YQSRYVE-----PMHSERDAVKRIVGARTVP---AIVDDETGVA-M--AESAN------IVAYLDATYGGG-------E----------------------------------------------------------------- |
| Q9HNG5 | UniRef cluster | --------------------------------------------MS-LELYKL--PGCPYCAKVETKLDE--LGLD-------YVEHEVP-----SSHSDRDAVESVSGQTGVP---VLVDPDHDIDGM--PESDD------IVAHLEQHYAE-------------------------------------------------------------------------- |
| Q9HQT6 | UniRef cluster | -----------------------------------MRVSTVPIGMSQVTFYAR--EDCPYSQKVRSKLDA--LDVA-------YEETLVP-----DAHTDRTTVEDVTGQTGVP---VVIDDHMEPSFL--ADTQE------IITHLETQYA--------------------------------------------------------------------------- |
| Q9HS15 | UniRef cluster | -----------------------------MAQDAVDDIVDAAIENNEVVLFMKGTPAAPQCGFSERAIRLI-SDYR-------PDVHTVDVLQ--STDEFRAALERHSGWETTP---QAFVDGSFVGGSDILAELA------DRGDLADELNADGVDTTADDAALD-ADIDAPF----------------------------------------------------- |
| Q5V5M0 | UniRef cluster | ---------------------------------------------MSLTLYQL--DGCPYCEKVADRLDE--LGIE-------YDSVWVE-----ALHSKRDEVKRVSGQRGVP---VLVDDDRGVT-M--AESDR------ILELIETTYAPEAQSA--------------------------------------------------------------------- |
| Q5V2K9 | UniRef cluster | MMGPHSPRRYAFIADAVSGGMAFEP--EELSPEEVTEQVDSVIEDNEVVLFMKGNELMPQCGYSKKALALL-QQHR-------DDIETVDVLK--ATDAYREALERHSGRETIP---QTFVDGEFIGGSDILEQLD------ERGELAQKVGQ-------------------------------------------------------------------------- |
| B0R3E0 | UniRef cluster | --------------------MAFDPLDSSMAQDAVDDIVDAAIENNEVVLFMKGTPAAPQCGFSERAIRLI-SDYR-------PDVHTVDVLQ--STDEFRAALERHSGWETTP---QAFVDGSFVGGSDILAELA------DRGDLADELNADGVDTTADDAALD-ADIDAPF----------------------------------------------------- |
| B0R5V0 | UniRef cluster | ---------------------------------------------MTLTLYAL--DGCPDCESVIDTLAA--DDID-------HETVHVD-----ARHSARNAVKRASGQRSVP---VLVDDDRGVV-M--ADSQR------IQTYAAVTLA--------------------------------------------------------------------------- |
| B0R733 | UniRef cluster | --------------------------------------------MS-LELYKL--PGCPYCAKVETKLDE--LGLD-------YVEHEVP-----SSHSDRDAVESVSGQTGVP---VLVDPDHDIDGM--PESDD------IVAHLEQHYAE-------------------------------------------------------------------------- |
| B0R7K4 | UniRef cluster | -------------------------------MSAPAAPPRPPHSDAHITLYRL--QACPFCERVVNRLEE--LGLA-------YQSRYVE-----PMHSERDAVKRIVGARTVP---AIVDDETGVA-M--AESAN------IVAYLDATYGGG-------E----------------------------------------------------------------- |
| B0R4N8 | UniRef cluster | -----------------------------------MRVSTVPIGMSQVTFYAR--EDCPYSQKVRSKLDA--LDVA-------YEETLVP-----DAHTDRTTVEDVTGQTGVP---VVIDDHMEPSFL--ADTQE------IITHLETQYA--------------------------------------------------------------------------- |
| Q3IT18 | UniRef cluster | --------------------------------------------MANITLYEL--AGCPYCKKVIDKLDE--LGLD-------YDSIEVP-----RSHSDRTEVEDVSGQTGVP---VIIDEEHGIEGM--PESDD------IVEYLEETYGS-------------------------------------------------------------------------- |
| Q3IST6 | UniRef cluster | ------------------------------------------MSEPDITLYRL--QACPFCERVVAVLDE--LGLA-------YRSRFVE-----ARHSRRDVVKRLTGARTVP---ALVDDRTGVT-M--SESAN------IVEYLRATYGDG---GGGAE----------------------------------------------------------------- |
| Q3IRW7 | UniRef cluster | --------------------MTFDPNADAADPENVQERVDTLIEDNEVVLFMKGNELMPQCGYSKKALQLL-TKYR-------DDVVTEDVLD--ALDAYRAALESHSGWETIP---QTFVDGEFIGGSDILEELD------NRGELHDELGVDADDVDADDAGTDDAGAEAPF----------------------------------------------------- |
| Q3IPI1 | UniRef cluster | ---------------------------------------------MTLTLYRL--EGCPYCEFVVDTLED--LPLD-------FESVWVE-----GLHSKRNEVHEITGQRQVP---ALVDDAHGVS-M--SQSAR------IIEYLETTYGDATSPDEVELDF--------------------------------------------------------------- |
| Q18FD8 | UniRef cluster | ---------------------------------------------MTITLYAL--DGCPYCETVHDALTD--AGVE-------YTTIWVD-----ALHSERDEVRRVSGQRGVP---VLVDEARGVT-M--CESTN------IETYVQQTLAGE------------------------------------------------------------------------- |
| Q18E41 | UniRef cluster | -------------------------------MSADS-----TNSDVSITVYRL--EACPFCERVIRLLEE--LELS-------YHSRFVE-----PMHSDRDVVKRISGKRTVP---ALVDTNTGVV-M--SESGN------IVTYLEQTYGSD--STTSMEKPVEGV----------------------------------------------------------- |
| Q18JL3 | UniRef cluster | --------------------MTFQPGSELSD-EEITTRVNETIADNDIALFMKGNRLMPQCGYSQRAVNLI-SQHV-------EEFETIDVLP--ALDQYRSALEDHSGWETIP---QTYVDGEFIGGSDILAELD------ERGELAEKLGFDD------------------------------------------------------------------------ |
| Q18FU5 | UniRef cluster | -------------------------------------------MMSNLTLYEL--SGCPYCAKVIDKLDE--LGLE-------YDSVSVP-----RAHSERTEVESISNQTGVP---VLVDEANDVSGM--PESDD------IVAYLEKTYAN-------------------------------------------------------------------------- |
| Q9V2B3 | UniRef cluster | -----------------------------------EEFFSKMVNPVKLIVFIG-KEHCQYCDQLKQLVQEL-SELTDK-----LSYEIVDFDTPEGKELAEKYRIDRAPATTITQDGKDFG-VRYFG----IPAGH-----EFAAFLEDIVDVSRAETDLMAESKEEVAKI-------------------------------------------------------- |
| Q9V2B3 | UniRef cluster | -----------------------------------KEEVAKIDKNVRILVFVT--PTCPYCPLAVRMAHKF-AIENTKAGKGKILGDMVEAI--EYPEWADQYNVMAVPKIVIQVD--GEDKVQFEG----AYPEK-----MFLEKLLAALS--------------------------------------------------------------------------- |
| Q980T5 | UniRef cluster | -----------------------------------EAIKSKLNGKVKIETVVT--PSCPYCPYAALMAHMV-AFEACRAGKCNVISEVIEAY--ENQDIAEKYQVMSVPAIAINES------IEFIG----VPYEE-----NFINAILEKQ-------KIS------------------------------------------------------------------ |
| Q97CA1 | UniRef cluster | -----------------------------------GEFDKYLKNDVDLVVFTSNDENCRYCKETVQLATEV-SEINPK-----IHLKVYNFD--EDKDQVKAYGVEKYPATIVSKAGVEDGRIVYYG----LPSGY-----EFGSLIEDLKNVS------------------------------------------------------------------------- |
| Q97CA1 | UniRef cluster | -----------------------------------AELISKIDKPITIKVYVT--PTCPYCPRAVGTAHKF-ALMN-----PNIKGEMIEAL--EFENEAEEVGVSSVPHIVIN------NDVTFIG----AYPDD-----QFAEYVMEAYDHQ------------------------------------------------------------------------- |
| Q6L248 | UniRef cluster | -----------------------------------DEFNGKLKEPVDLVVFTSNKSDCKYCKETVQLAEEL-SSINEK-----INLIKYVYE--DNKDAVNDYGVEKYPATIVAKHGQKDGRIVYYG----IPSGY-----EFGSLIEDIENVS------------------------------------------------------------------------- |
| Q6L248 | UniRef cluster | -----------------------------------MELISKVDRPLTIKVYVT--PTCQYCPRAVGTAHKF-ALLN-----KNIKAEMIESL--EFDKEAEEVGVSAVPHVVIN------DDVTFVG----AQPDD-----QFAEFIMEAYNHQD------------------------------------------------------------------------ |
| Q5V2K9 | UniRef cluster | -----------------------------------TEQVDSVIEDNEVVLFMKGNELMPQCGYSKKALALL-QQHR-------DDIETVDVLK--ATDAYREALERHSGRETIP---QTFVDGEFIGGSDILEQLD------ERGELAQKVGQ-------------------------------------------------------------------------- |
| Q5JGG9 | UniRef cluster | ---------------------------------VDVNGTRIYLDQIHFYMYGM--KTCPHCRHMKEWIPEE-FGADSL---TYYELVNDE-----TNSELFGQLAQLTGITGVPAIAITYNGTIQAIFEGEFNVSA------TPEIVATAMNANGVILFIGGEWYLLA----------------------------------------------------------- |
| Q5JE30 | UniRef cluster | -----------------------------------EEFFSKMTNPVKLIVFVG-KEHCQYCDQLKQLVQEL-SELTDK-----LSYEVVDFDTEEGKKLAEQYRIDRAPATTITQDGKDMG-VRYFG----LPAGH-----EFGAFLEDIVDVSNGTTDLAPDTKEAIHGV-------------------------------------------------------- |
| Q5JE30 | UniRef cluster | -----------------------------------KEAIHGVDKDVRILVFVT--PTCPYCPLAVRMAHKF-AIENTLAGKGKILGDMVEAI--EYPEWADQYSVMAVPKIVIQVD--GEDKVQFEG----AYPEK-----MFLEKLLAALE--------------------------------------------------------------------------- |
| Q51760 | UniRef cluster | -----------------------------------EEFFSKMVNPVKLIVFVR-KDHCQYCDQLKQLVQEL-SELTDK-----LSYEIVDFDTPEGKELAKRYRIDRAPATTITQDGKDFG-VRYFG----LPAGH-----EFAAFLEDIVDVSREETNLMDETKQAIRNI-------------------------------------------------------- |
| Q51760 | UniRef cluster | -----------------------------------KQAIRNIDQDVRILVFVT--PTCPYCPLAVRMAHKF-AIENTKAGKGKILGDMVEAI--EYPEWADQYNVMAVPKIVIQVN--GEDRVEFEG----AYPEK-----MFLEKLLSALS--------------------------------------------------------------------------- |
| Q2FT67 | UniRef cluster | ------------------------------------MTDTYLSHMPEVTVYST--QNCPYCRLAKAFLDR--NNIP-------YRSVDVG-----IDRKAAKEMVELSGQYGVP-VIVA-GEEVIVG----FDTDK------LRALFTTGKKPDMFDVIIVGA---------------------------------------------------------------- |
| O57917 | UniRef cluster | -----------------------------------EEFFSKMVNPVKLIVFIG-KEHCQYCDQLKQLVQEL-SELTDK-----LSYEIVDFDTPEGKELAEKYRIDRAPATTITQDGKDFG-VRYFG----IPAGH-----EFAAFLEDIVDVSKGDTDLMQDSKEEVSKI-------------------------------------------------------- |
| O57917 | UniRef cluster | -----------------------------------KEEVSKIDKDVRILIFVT--PTCPYCPLAVRMAHKF-AIENTKAGKGKILGDMVEAI--EYPEWADQYNVMAVPKIVIQVN--GEDKVQFEG----AYPEK-----MFLEKLLSALS--------------------------------------------------------------------------- |
| A8MAS4 | UniRef cluster | ---------------------------------------------MEVEVFVH--PTCSTCHSLIRLLKQW-GFID--------KVKIYDTS--IDPHAALERGVRSVPSIFID------GDLVFAG---VVDFKR------LKSILDTGLHTERVMLSDEELIE-------------------------------------------------------------- |
| A8M8V0 | UniRef cluster | -----------------------------------ASMINSDAKTVEVVTVVT--PSCPYCPYAVLLANMF-AYESK--GK--VRSVVVEAY--EEPDIADMYGVTAVPTVVIRNE-GSTGDVEFVG----VPPEA-----DLLKKVLSYSGFNP------------------------------------------------------------------------ |
| A8AAY4 | UniRef cluster | ----------------------------------KEGLK-KLKGCYYIENVVT--PQCPYCPYAALLINMF-AFEAKKQGNPCVVADTVEAY--ENEDIADKYNVMSVPAIAINGN------VEFVG----VPYED-----DLLAKLFEVEPKGPCEDEVC------------------------------------------------------------------ |
| A7I8I7 | UniRef cluster | ----------------------------------ESFIRKLWGKPRVVRVYAT--PGCSGCRAVKEYLKS--KNVE-------FTEIDIA-----ADERARTLVMEKTGHLGSP--YVQIDDTFIFG----FDRKK------LDQLLQGT----------------------------------------------------------------------------- |
| A7I4E7 | UniRef cluster | -------------------------------------------MMSTVTVYST--KNCPYCRMAKAFLEK--YGVP-------YTAIDVG-----ADTAAAHKMIALSGQRGVP-VITV-DDEVIVG----FDSQR------LNELFGTHAAGETYDVIIVGAGPGG------------------------------------------------------------ |
| A6UTS1 | UniRef cluster | ------------------------------------------MANNLLKLYVN--STCPLCNILKNILEY--NNIK-------FELINIEDIEESDLNEEFKVFNNIGDIKNIAFPSIVLGNEIIVG----YNIEK------LEKLLNKKLLKPPVTEKSFID---------------------------------------------------------------- |
| A3MWU7 | UniRef cluster | --------------------------------------AQNAPKRVYVLTVVT--PSCPYCPYAVLLANMF-AYESK--GK--VVSVVVEAY--ENPDIADMYGVTGVPTVILQAEDAAVGEVEFVG----VPPEH-----ELLAKVKNHMGL-------------------------------------------------------------------------- |
| A3DKZ8 | UniRef cluster | -----------------------------------IEEAKKIDVPLHIKIFVT--PECPYCPLTVDAFNQL-ALIN-----DKILVETIEAI--ELPLEADMYNVAYVPDVIITD----PDKMDEYG----VEPVERINGYMPIEEAINIVKYAAEKLKEMKKQG-------------------------------------------------------------- |
| A1RS60 | UniRef cluster | -------------------------------------------KRVYILTVVT--PSCPYCPYAVLLANMF-AYESK--GK--VVSVVVEAH--ENPDIADMYGVTGVPAVILQAEDVSVGDVEFVG----VPPEH-----ELLARVKNHMGLS------------------------------------------------------------------------- |
| A1RX05 | UniRef cluster | -----------------------------------EILLKYVTKPTRIMIFVT--PTCPYCPIAVRAAHRF-AMVN----KN-IYGDMIEAL--EFSDLADRYGVYAVPKNVIQVN--GEDKNEFEG----AAPDP-----YFVAKILEAYGVEIPRSLQEAIAGI------------------------------------------------------------- |
| A2STK9 | UniRef cluster | -------------------------------------------MLHEVVVYSL--SGCPHCKALKTFLDN--QNIT-------YTNIDVG-----EDEKAAAEMIKISGQRGVP-VTVIDGEKIVIG----DDLKK------VMEYLDAPVAVKKTPDVSADH---------------------------------------------------------------- |
| A3CRV4 | UniRef cluster | -----------------------------------NTVPEIFPFMAGVKVYTT--ENCPYCRMVKAFLRK--HDIE-------HEIVDVG-----KDREAAREMIEISGQRGVP-VTVS-GDEVVVG----FDAKR------LRELFGTALEETVYDAVIVG----------------------------------------------------------------- |
