# Supplementary material for: Evolution based on domain combinations: the case of glutaredoxins
Source: BMC Evol Biol. 2009 Mar 25;9:66. doi: 10.1186/1471-2148-9-66 (PMC2679010; doi:10.1186/1471-2148-9-66)
Supplement: Additional File 2 — GRX sequences from bacteria. UNIPROT links to the GRX sequences from bacteria, together with protein and DNA alignments and links to other sequences that have over 90% sequence identity. [file 1471-2148-9-66-S2.htm]

Supplementary file 2: Bacteria


#### Alignment for Bacteria GRXs

|  |  |  |
| --- | --- | --- |
| UNIPROT accession number | UNIPROT 90\% similarity cluster | Aligned Sequence |
| A5G1X6 | UniRef cluster | --------------------------------MSQATENPAFASIKTAVEENPVMLFMKGTAMFPQCGFSARVVQILTHMGVPFKTAN-----VLEDPELRDGIKQFSNWPTIPQLYVKG-----EFVGGCDIVTEMFQS--GELQTLFDQNGIARTGA-------------------- |
| A9GUN5 | UniRef cluster | -------------------------------------MTETTSKIQEAISAKDVVLFMKGTKEMPQCGFSSRVAGVLNYMGVDFSDVN-----VLSDETIRQGIKEFSDWPTIPQLYVKG-----EFVGGCDIITEMTLS--GELDTLFAENGVEFDKDAADKIREANG---------- |
| Q2RWI6 | UniRef cluster | --------------------------------------MSVQDRIKSEVDGTPVVLFMKGTPAFPQCGFSAAVVQVLNHYGVAFKGIN-----VLEDDEIRQGIKEFANWPTLPQLYVKG-----EFVGGCDIVREMAAD--GELATLFEKSGIPTTAA-------------------- |
| A6GMB7 | UniRef cluster | --------------------------------------MSVRDSIHETVTSHPVVLFMKGTAQFPMCGFSGRAIQILKACEAE--KVVTVN--VLDDPEIRQGIKEYANWPTIPQLYVNG-----EFLGGCDIMTEMYQS--GELKALIAAANASTKSE-------------------- |
| A6E400 | UniRef cluster | -------------------------------------MTDAKTRIEETVKSADVVLYMKGTKDMPQCGFSSRVAGVLNYMGVDFKDVN-----VLDDADIRQGIKDYSDWPTIPQLYVKG-----EFVGGCDIITEMTLS--GELDDLFEKSGVSYDKDAAEKIREANA---------- |
| A3VN36 | UniRef cluster | ------------------------------------MTASAREEIDSAIKSNDIMLFMKGTPQFPQCGFSSAVVQILDYLGADYGSMN-----VLEDQEIRQGIKDYSDWPTIPQLYVKG-----EFVGGCDIIREMFEQ--GELRPFLAEKGLITAEA-------------------- |
| Q5P7G9 | UniRef cluster | --------------------------------------MDTQEVIREQVTTNPVVLYMKGTPQFPQCGFSSTAVQILKNSGVP--KFFSVN--VLENDEIRNGIKQFANWPTIPQLYVNG-----EFVGGCDIMREMYEN--GELQEVLKSAGATQQG--------------------- |
| A9KCM8 | UniRef cluster | --------------------------------------MRVHEEIQQQVTSNPVVLYMKGTPDFPQCGFSGRVVQILRQCKIDFTSFN-----VLESPELRQGIKEFSSWPTIPQLYVKG-----EFIGGCDIVGELFET--GKLQELL------------------------------ |
| A9HJG8 | UniRef cluster | ------------------------------------MADTITQRIQNDIDTNPVMLYMKGTAQFPQCGFSAKVVKILNHLGVPFQAAN-----VLEDPELRQGVKDFTNWPTVPQLYVKG-----EFIGGCDIVSEMFQT--GELEKLFVEKGIVTASA-------------------- |
| Q83DV9 | UniRef cluster | --------------------------------------MRVHEEIQQQVTSNPVVLYMKGTPDFPQCGFSGRVVQILRQCKIDFTSFN-----VLESPELRQGIKEFSSWPTIPQLYIKG-----EFIGGCDIVGELFET--GKLQELL------------------------------ |
| A9ZIW0 | UniRef cluster | --------------------------------------MRVHEEIQQQVTSNPVVLYMKGTPDFPQCGFSGRVVQILRQCKIDFTSFN-----VLESPELRQGIKEFSSWPTIPQLYIKG-----EFIGGCDIVGELFET--GKLQELL------------------------------ |
| A9NC71 | UniRef cluster | --------------------------------------MRVHEEIQQQVTSNPVVLYMKGTPDFPQCGFSGRVVQILRQCKIDFTSFN-----VLESPELRQGIKEFSSWPTIPQLYIKG-----EFIGGCDIVGELFET--GKLQELL------------------------------ |
| A8TMY6 | UniRef cluster | -----------------------------------MLDETLRQRIDQEVGGNDVVLFMKGTPVFPQCGFSATVVQVLSHLGVKFKGIN-----VLEDPAIRDGIKEYSSWPTIPQLYVKG-----EFVGGCDIIREMFET--GELTQMLNTHGVEARPAA------------------- |
| A3W362 | UniRef cluster | -------------------------------------MTDAKTRIEETVKSADVVLYMKGTKDMPQCGFSSRVAGVLNYMGVDFKDVN-----VLADEDVRQGIKDYSDWPTIPQLYVKG-----EFVGGCDIITEMTLS--GELDELFEKSGVSYDKDAAEKIREANA---------- |
| Q28QC4 | UniRef cluster | --------------------------------------MTAETQIKDAITANDVVLFMKGTKEMPQCGFSSRVAGVLNYMGVDFTDVN-----VLADEGMRQGIKEFSDWPTIPQLYVKG-----EFVGGCDIITEMTLS--GELDTLFSENGVTYDKDAADKIREANG---------- |
| Q164U4 | UniRef cluster | -------------------------------------MTETTTRIQETITANDVVLFMKGTKEMPQCGFSSRVAGVLNYMGVDFSDVN-----VLSDESIRQGIKDFSDWPTIPQLYVKG-----EFVGGCDIITEMTLS--GELDTLFAENGISFDKDAADKIREANG---------- |
| B0SSC3 | UniRef cluster | ------------------------------------MEQELKDKIESLIKSEKVFLFMKGTPEMPQCGFSAGVVSTLKQQGISFGSFN-----VLSDMNIREGIKEYTNWPTIPQLYING-----EFVGGHDITVQMAQS--GELKKKIG----------------------------- |
| B0S9Z1 | UniRef cluster | ------------------------------------MEQELKDKIESLIKSEKVFLFMKGTPEMPQCGFSAGVVSTLKQQGISFGSFN-----VLSDMNIREGIKEYTNWPTIPQLYING-----EFVGGHDITVQMAQS--GELKKKIG----------------------------- |
| A3WVM2 | UniRef cluster | --------------------------------------MSIKETIENELKSNDVVLFMKGTPQFPQCGFSGQVVQILDHVGVGYKGLN-----VLESPELRDGIKTYSNWPTIPQLYVKG-----EFVGGCDIVREMFQA--GELQKLFSDKGITFSAPASA----------------- |
| Q5NLB3 | UniRef cluster | ------------------------------------MADTINDRIKEVLEKSPVVLFMKGTPLFPQCGFSNQIVSILNAVGIEYDSVD-----VLQDPEIRQGIKVYSDWPTFPQLYVKG-----ELVGGCDIVTEMYQS--GELAELMQKENIARKN--------------------- |
| Q3ST18 | UniRef cluster | --------------------------------------MSIKETIENELKSNDVVLFMKGTPQFPQCGFSGQVVQILDHVGVGYKGLN-----VLESPEMRDGIKTYSSWPTIPQLYVKG-----EFIGGCDIVREMFQA--GELQKLLSDKGIAFNTPASA----------------- |
| A3U2V5 | UniRef cluster | -------------------------------------MTDVKTSIDETVKANDVVLFMKGTKEMPQCGFSSRVAGVLNYMGVDYSDVN-----VLADEGIRQGIKDYSDWPTIPQLYVKG-----EFVGGCDIVTEMTLS--GELDTLFEANGVTYDKDAAEKIRAANA---------- |
| A9FQL5 | UniRef cluster | -------------------------------------MTDVKTRIDETVKASDVVLFMKGTKDMPQCGFSSRVAGVLNYIGVDYTDVN-----VLADEEIRAGIKDYSDWPTIPQLYVKG-----EFVGGCDIITEMTLS--GELDGMFEQNGVTFDKDAADKIREANG---------- |
| A9F311 | UniRef cluster | -------------------------------------MTDVKTRIDETVKASDVVLFMKGTKDMPQCGFSSRVAGVLNYIGVDYTDVN-----VLADEEIRAGIKDYSDWPTIPQLYVKG-----EFVGGCDIITEMTLS--GELDGMFEQNGVTFDKDAADKIREANG---------- |
| A3SQ81 | UniRef cluster | -------------------------------------MSDAKTRIDETVKSNDVVLYMKGTKEMPQCGFSSRVAGVLNYMGVNYADVN-----VLADEEIRQGIKEYSDWPTIPQLYVKG-----EFVGGCDIITEMTLS--GELDTLFEENGVTYDKDAAEKIREANA---------- |
| Q0C0U3 | UniRef cluster | -----------------------------------MTDQATLDAIEKAVKSNDVVLFMKGTPTFPQCGFSSTVVQILDYLGVEYVATN-----VLEDQNVREGIKQYANWPTIPQLYVKG-----EFVGGCDILKEMFEN--GELRDLMAENGIELAE--------------------- |
| A9GFE2 | UniRef cluster | -------------------------------------MSDIHQAIKDTVESNRVVLFMKGTKTFPQCGFSARAVDILK--KCG-VDFKDVN--VLSDPSLRQGIKDYSQWPTIPQVYVDG-----KFIGGSDILMEMFQS--GDLQKLLSKQD-------------------------- |
| A9FPP7 | UniRef cluster | -------------------------------------MTDINTRIENEIKSQDVVVFMKGTPQFPMCGFSGQVAQILNYLGVDYKGIN-----VLEDMEIREGIKAYSNWPTIPQVYVKG-----EFVGGCDITREMFQS--GELQQLLAERGIAVKTAAA------------------ |
| Q82TH6 | UniRef cluster | --------------------------------------MDVVDSIEKQVSSHPVVLYMKGTPQQPQCGFSANAIRILNACGVE--DFFAVN--VLADPEIRQGIKDYSSWPTIPQLYVNG-----EFIGGSDIMSEMYQN--GELQKLFEK---------------------------- |
| A9IER4 | UniRef cluster | -------------------------------------MSDVQEFIRDTVTQHPVVLFMKGTAQFPQCGFSGRAIQILKDCGVK--KLVTVN--VLEDDEVRQGIKEYSNWPTIPQLYVSG-----EFIGGSDIMTEMNEN--GELKTLLETAGATTA---------------------- |
| A5VFI3 | UniRef cluster | ------------------------------------MSDDVQARIATAVHSADVLLFMKGTPLFPQCGFSSRAIAILDHLGVE---YATVD--VLQDPAIRTGIKEFSDWPTIPQLYVKG-----EFVGGSDIMMEMYEA--GELLDLLDQNGIAHA---------------------- |
| A4BPF0 | UniRef cluster | -------------------------------------MSNVQETIRKQVEDNPVILYMKGTPQFPECGFSMRTVQALDSCGVQYATVN-----VLENEGIRQGVKEFGNWPTIPQLYING-----ELVGGCDIIMELYQS--GELKRQLQEAAGKVE---------------------- |
| Q5LSA0 | UniRef cluster | -------------------------------------MTDAKTRIDETVKANDVVLYMKGTKEMPQCGFSSRVAGVLNYMGVAYADVN-----VLADDEIRQGIKDYSDWPTIPQLYVKG-----EFVGGCDIITEMTLS--GELDTLFEENGVAYDKDAADKIRAANA---------- |
| A7HWA9 | UniRef cluster | -----------------------------------MSENPVFDRIKGEVASQDVVLFMKGTPVFPQCGFSNAVVQVLTYLGVPFKGIN-----VLEDDDIRQGIKEFSEWPTIPQLYVKG-----EFVGGCDIVREMFEQ--GELRDYLAQKGVQTEAA-------------------- |
| Q2ITR1 | UniRef cluster | -------------------MSIRPGRWWSAGRAAKGSAMSIEQFIDNEVKANDVVLFMKGTPQFPQCGFSGQVVQILDHIGVAYKGHN-----VLENAELRDGIKQFSNWPTIPQLYVKG-----EFVGGCDIVREMFQA--GELQKLFTDKGIKAAA--------------------- |
| Q0AHT9 | UniRef cluster | --------------------------------------MDIADSIEQQVSTHPVVLYMKGTPQQPQCGFSANAVQILSACGVE--DFFAVN--VLADPEIRQGIKDYSSWPTIPQLYVNG-----EFVGGSDIMAEMFQN--GELKKLLEK---------------------------- |
| A9E3V6 | UniRef cluster | -------------------------------------MSDAANQIKEQITKNDVVLFMKGTKEMPQCGFSSRVAGVLNYMGVNFADVN-----VLADEGLRQGIKEFSDWPTIPQLYVKG-----EFVGGCDIITEMTLS--GELDTLFAENGVTFDKDAAEKIREANG---------- |
| A3V4A4 | UniRef cluster | ----------------------------MKHDLTKDRHMTAKDQIQETITSNDVVLFMKGTKSMPQCGFSSRVAGVLNFMNVDFADVN-----VLADEEIRQGVKDFSDWPTIPQLYVKG-----EFVGGCDIITEMTLS--GELDTLFAEKGVSFDKDAAEKIREANA---------- |
| Q2K7V2 | UniRef cluster | -------------------------------------MSGIHEFIDNEIKSNDVVLFMKGTPQFPQCGFSGQVVQILDYIGVDYKGIN-----VLADSEIRQGIKDYSNWPTIPQLYIKG-----EFIGGCDIVREMFQA--GELQQHLQENGVTVRGAA------------------- |
| Q1QMT7 | UniRef cluster | -------------------------------------MMSIKDFIETELKSNDVVLFMKGTPQFPQCGFSGQVVQILDHVGVGYKGLN-----VLESPDLRDGIKTYSNWPTIPQLYVKG-----EFVGGCDIVREMFQA--GELQKLLTDKGITLSAPASA----------------- |
| Q1MG20 | UniRef cluster | -------------------------------------MSGIHEFIDNEIKTNDVVLFMKGTPQFPQCGFSGQVVQILDYIGVDYKGVN-----VLADSEIRQGIKEYSNWPTIPQLYVKG-----EFVGGCDIVREMFQA--GELQQHLQENGIAVRAAS------------------- |
| B0J576 | UniRef cluster | -------------------------------------MSGIHEFIGNEIKSNDVVLFMKGTPQFPQCGFSGQVVQILDYIGVDYKSVN-----VLADSEIRQGIKEYSNWPTIPQLYVKG-----EFVGGCDIVREMFQA--GELQQHLQENGIAVRAAS------------------- |
| A6FRG1 | UniRef cluster | -------------------------------------MTDAKNQIEETVKANDVVLYMKGTKDMPQCGFSSRVAGVLNYMGVDFKDVN-----VLADETVRQGIKDYSDWPTIPQLYIKG-----EFVGGCDIITEMTLS--GELDQMFDQHGVSYDKDAADKIREANG---------- |
| A5EMH3 | UniRef cluster | --------------------------------------MSIEQFIDNEVKSNDVVLFMKGTPQFPQCGFSGQVVQILDHVGVGYKGLN-----VLESAELRNGIKTFSNWPTIPQLYVKG-----EFIGGCDIVREMFQS--GELQQLFTDKGIPVGTAASA----------------- |
| A4YRS7 | UniRef cluster | --------------------------------------MSIEQFIDNEVKSNDVVLFMKGTPQFPQCGFSGQVVQILDHVGVGYKGLN-----VLESAELRNGIKTFSNWPTIPQLYVKG-----EFIGGCDIVREMFQS--GELQQLFTDKGIPVGTAASA----------------- |
| A6G1U1 | UniRef cluster | ---------------------------------MSDVETETNTRIRELIDQNRVMLFMKGNKIFPSCGFSAQVVQILKHHGADFQTFN-----VLADPAMRQGIKVFSEWPTIPQLYVDG-----EFVGGCDIVTQLHNS--GELEQVLAPKS-------------------------- |
| A3UDW8 | UniRef cluster | -----------------------------------MSDDATQEAIRKAVTENDVVLFMKGTPTFPQCGFSSVVARVLDHVGVEYAAVN-----VLEDHAVREGIKVYSDWPTIPQLYVKG-----EFVGGCDIIKEMFEA--GELQQLLKEKGLVEA---------------------- |
| A3JSA5 | UniRef cluster | -------------------------------------MTDAATQIKSTVTNNDVVLFMKGTKDAPQCGFSSRVAGVLNYMGVDFADVN-----VLADENIRQGIKEYSDWPTIPQLYIKG-----EFVGGCDIITEMTLS--GELDTMFESNGVGYDKEMADKIREANS---------- |
| Q5PBN7 | UniRef cluster | -----------------------------------MSSDPVADRIRNEIETSDVVLYMKGTADAPQCGFSGVVVEALRNVGVQFRDVD-----VLKDPELREGIKKFADWPTIPQLYVKG-----EFIGGCDIVREMYHS--GELHALLKSKGIIAG---------------------- |
| Q2W5F4 | UniRef cluster | ------------------------------------MNNPVFDRIRQDLSENDVVLYMKGTPMFPQCGFSAAVVQVLTNLGVKFKGID-----ILVDPSLRDGIKQFTNWPTLPQLYVKG-----EFVGGCDIVREMAES--GELKQLMTDKGVATA---------------------- |
| A1B1U9 | UniRef cluster | -------------------------------------MTDARQQIQETIDGDDVVLFMKGTKEMPQCGFSSRVAGVLNYMNVQYRDVN-----VLADDTIRQGIKDFSDWPTIPQLYVKG-----EFVGGCDIVTEMTLS--GELDQLFDKAGVTYDKDAAEKIREANA---------- |
| Q92PH5 | UniRef cluster | -------------------------------------MSGINDFIDNEVKSNDVVLFMKGTPQFPQCGFSGQVVQILDYVGVDYKGIN-----VLADADLRQGIKDYSNWPTIPQLYVKG-----EFVGGCDIVREMFQA--GELQSLLQGQGISVKGAA------------------- |
| Q63QE8 | UniRef cluster | --------------------------------------MDTQQRIKQIVDENPVVLFMKGTAQFPMCGFSGRAVQVLKACGVD--QFKTVN--VLEDEEIRQGIKEFSNWPTIPQLYVKG-----EFVGGSDIMMEMYQS--GELQQLFTA---------------------------- |
| Q62DF5 | UniRef cluster | --------------------------------------MDTQQRIKQIVDENPVVLFMKGTAQFPMCGFSGRAVQVLKACGVD--QFKTVN--VLEDEEIRQGIKEFSNWPTIPQLYVKG-----EFVGGSDIMMEMYQS--GELQQLFTA---------------------------- |
| Q3JN82 | UniRef cluster | --------------------------------------MDTQQRIKQIVDENPVVLFMKGTAQFPMCGFSGRAVQVLKACGVD--QFKTVN--VLEDEEIRQGIKEFSNWPTIPQLYVKG-----EFVGGSDIMMEMYQS--GELQQLFTA---------------------------- |
| Q2JJ78 | UniRef cluster | -----------------------------------MLDPLLEEKIREQIRTHKVLIYMKGTPEMPQCGFSYAAVRVLDSLGFPYTAIN-----VLEDPEIRQGIKEFSNWPTIPQIYIDG-----EFVGGCDIIQEMHAR--NELRPLVEAAFAKATAQS------------------- |
| Q2C9Z9 | UniRef cluster | -----------------------------------MTTATAEDQIRDTVTSNDVVLFMKGTKTMPQCGFSSRVAGVLNYMGIDYADVN-----VLADDAIRQGIKDYSDWPTIPQLYVKG-----EFIGGCDIITEMTLS--GELDQLFEAKGVAFDKDAADKIREANG---------- |
| Q0FK68 | UniRef cluster | -------------------------------------MTDAKTMIDETVKSNTVVLYMKGTKTMPQCGFSSRVAGVLNYMGVDFHDVN-----VLADDAIRQGIKDYSDWPTIPQLYVKG-----EFVGGCDIITEMTLS--GELDQLFEQSGVEYNKDAADKIREANA---------- |
| Q07R65 | UniRef cluster | --------------------------------------MSIEQFIESEVKSNDVVLFMKGTPQFPQCGFSGQVVQILDHVGVAYKGHN-----VLESADLRDGIKAYSNWPTIPQLYVKG-----EFVGGCDIVREMFQA--GELQQLFTDKGITVTASA------------------- |
| B0UM07 | UniRef cluster | -------------------------------------MTDINSRIESEIKSQDVVVFMKGTPQFPMCGFSGQVAQILNYLGVPYKGVN-----VLEDMEIREGIKAYSNWPTIPQVYVKG-----EFVGGCDITREMFQS--GELQQLLSEKGIAVKTAAA------------------ |
| A9K2X0 | UniRef cluster | --------------------------------------MDTQQRIKQIVDENPVVLFMKGTAQFPMCGFSGRAVQVLKACGVD--QFKTVN--VLEDEEIRQGIKEFSNWPTIPQLYVKG-----EFVGGSDIMMEMYQS--GELQQLFTA---------------------------- |
| A8KT37 | UniRef cluster | --------------------------------------MDTQQRIKQIVDENPVVLFMKGTAQFPMCGFSGRAVQVLKACGVD--QFKTVN--VLEDEEIRQGIKEFSNWPTIPQLYVKG-----EFVGGSDIMMEMYQS--GELQQLFTA---------------------------- |
| A8EMH7 | UniRef cluster | --------------------------------------MDTQQRIKQIVDENPVVLFMKGTAQFPMCGFSGRAVQVLKACGVD--QFKTVN--VLEDEEIRQGIKEFSNWPTIPQLYVKG-----EFVGGSDIMMEMYQS--GELQQLFTA---------------------------- |
| A6T357 | UniRef cluster | ------------------------------------MSDDVQSWIKETVSQNPVVLFMKGTAQFPQCGFSGKAIQLLKESGVQ--DLVTVN--VLDDAEVRQGIKDFSQWPTVPQLYVKG-----EFIGGSDIMNEMFES--GELQALLKA---------------------------- |
| A5XWI0 | UniRef cluster | --------------------------------------MDTQQRIKQIVDENPVVLFMKGTAQFPMCGFSGRAVQVLKACGVD--QFKTVN--VLEDEEIRQGIKEFSNWPTIPQLYVKG-----EFVGGSDIMMEMYQS--GELQQLFTA---------------------------- |
| A5TDH7 | UniRef cluster | --------------------------------------MDTQQRIKQIVDENPVVLFMKGTAQFPMCGFSGRAVQVLKACGVD--QFKTVN--VLEDEEIRQGIKEFSNWPTIPQLYVKG-----EFVGGSDIMMEMYQS--GELQQLFTA---------------------------- |
| A5JAJ4 | UniRef cluster | --------------------------------------MDTQQRIKQIVDENPVVLFMKGTAQFPMCGFSGRAVQVLKACGVD--QFKTVN--VLEDEEIRQGIKEFSNWPTIPQLYVKG-----EFVGGSDIMMEMYQS--GELQQLFTA---------------------------- |
| A4LSM3 | UniRef cluster | --------------------------------------MDTQQRIKQIVDENPVVLFMKGTAQFPMCGFSGRAVQVLKACGVD--QFKTVN--VLEDEEIRQGIKEFSNWPTIPQLYVKG-----EFVGGSDIMMEMYQS--GELQQLFTA---------------------------- |
| A4G9E1 | UniRef cluster | ------------------------------------MSDDVQSWIKETVTQNPVVLFMKGTAQFPQCGFSGKAIALLKESGVT--DLVTVN--VLDDAEVRQGIKDFSQWPTVPQLYVKG-----EFIGGSDIMNEMFAS--GELQALLKA---------------------------- |
| A3NZS6 | UniRef cluster | --------------------------------------MDTQQRIKQIVDENPVVLFMKGTAQFPMCGFSGRAVQVLKACGVD--QFKTVN--VLEDEEIRQGIKEFSNWPTIPQLYVKG-----EFVGGSDIMMEMYQS--GELQQLFTA---------------------------- |
| A3NE26 | UniRef cluster | --------------------------------------MDTQQRIKQIVDENPVVLFMKGTAQFPMCGFSGRAVQVLKACGVD--QFKTVN--VLEDEEIRQGIKEFSNWPTIPQLYVKG-----EFVGGSDIMMEMYQS--GELQQLFTA---------------------------- |
| A3MFT6 | UniRef cluster | --------------------------------------MDTQQRIKQIVDENPVVLFMKGTAQFPMCGFSGRAVQVLKACGVD--QFKTVN--VLEDEEIRQGIKEFSNWPTIPQLYVKG-----EFVGGSDIMMEMYQS--GELQQLFTA---------------------------- |
| A2RYL4 | UniRef cluster | --------------------------------------MDTQQRIKQIVDENPVVLFMKGTAQFPMCGFSGRAVQVLKACGVD--QFKTVN--VLEDEEIRQGIKEFSNWPTIPQLYVKG-----EFVGGSDIMMEMYQS--GELQQLFTA---------------------------- |
| A1UWD1 | UniRef cluster | --------------------------------------MDTQQRIKQIVDENPVVLFMKGTAQFPMCGFSGRAVQVLKACGVD--QFKTVN--VLEDEEIRQGIKEFSNWPTIPQLYVKG-----EFVGGSDIMMEMYQS--GELQQLFTA---------------------------- |
| Q5FNK8 | UniRef cluster | ------------------------------------MSETVFQHIQNLIDANPVMLFMKGDKLFPQCGFSARVVQILTHLGVPFETCN-----ILESAELRQGIKDFSQWPTVPQLYIKG-----EFVGGCDIVTDMYQS--GELETLLTEKGITTANA-------------------- |
| Q13A93 | UniRef cluster | -------------------MTVRPGRWWSAGRAAKGSIMSIEQFIDNEVKSNDVVLFMKGTPQFPQCGFSGQVVQILDHVGVAYKGHN-----VLESADLRDGIKAYSNWPTIPQLYVKG-----EFVGGCDIIREMFQA--GELQKLFTDKGIKAAA--------------------- |
| A9W779 | UniRef cluster | -------------------------------------MTDVNSTIKNEIDSQDVVVFMKGTPQFPMCGFSGQVVQILNYLGVPFKGVN-----VLDDMAVREGIKAFSNWPTIPQIYVKG-----EFVGGCDIAREMFQS--GELQQFLSEKGVPVKSAA------------------- |
| A7WCG7 | UniRef cluster | -------------------------------------MTDVNSTIKNEIDSQDVVVFMKGTPQFPMCGFSGQVVQILNYLGVPFKGVN-----VLDDMAVREGIKAFSNWPTIPQIYVKG-----EFVGGCDIAREMFQS--GELQQFLSEKGVPVKSAA------------------- |
| A7IBI9 | UniRef cluster | -------------------------------------MASSAERIQDIISSADVVLFMKGVPAAPQCGFSSAVVQVLAQVGVPYRAVD-----VLQDPFVREGIKAFSNWPTIPQLYIKG-----EFVGGCDIVREMFQA--GELTALLAEKGVAAPVA-------------------- |
| A3K1U9 | UniRef cluster | ----------------------------------MTETTDAKTRIADTVNAHDVVLYMKGTKAMPQCGFSSRVAGVLNYMGVDFEDVN-----VLADDAIRQGIKDYSDWPTIPQLYVKG-----EFVGGCDIITEMTLS--GELDGLFEQNGISFNKDAADKIREANA---------- |
| Q3J271 | UniRef cluster | ------------------------------------MTTTVEEQIRDMVTTNDVVLFMKGTKSMPQCGFSSRVAGVLNYMQVAYKDVN-----VLEDADVRQGIKDFSDWPTIPQLYVKG-----EFVGGCDIITEMTLS--GELDQLFEAKGVSYDKTAADKIREANA---------- |
| Q1LIF0 | UniRef cluster | -----------------------MPAWRRAQPERISIMSDVQQKIDQIVKGSPVVLFMKGTAQFPMCGFSGRAIQILKACGVD--APTTVN--VLEDEGIRQGIKEYANWPTIPQLYVNG-----EFIGGSDIMMEMYQN--GELQSLLKA---------------------------- |
| Q1GHL7 | UniRef cluster | -------------------------------------MTDAKTRIDETVKANDVVLYMKGTKDMPQCGFSSRVAGVLNYIGVDYTDVN-----VLADEDIRQGIKDYSDWPTIPQLYIKG-----EFVGGCDIITEMTLS--GELDGMFEQNGISFDKDAADKIREANA---------- |
| A4ERU9 | UniRef cluster | -------------------------------------MSDANSRIDETVKANDVVLFMKGTKEMPQCGFSSRVAGVLNYIGVEYTDVN-----VLADEEIRQGIKDYSDWPTIPQLYIKG-----EFVGGCDIITEMTLS--GELDTMFDDNGVAFNKEAADKIREANG---------- |
| A3PK42 | UniRef cluster | ------------------------------------MTTTVEEQIRDMVTTNDVVLFMKGTKSMPQCGFSSRVAGVLNYMQVAYKDVN-----VLEDADVRQGIKDFSDWPTIPQLYVKG-----EFVGGCDIITEMTLS--GELDQLFEAKGVSYDKTAADKIREANA---------- |
| Q89IC8 | UniRef cluster | ------------------------------------MDMSIEEFIANEVKSNDVVLFMKGTPQFPQCGFSGQVVQILDHLGVGYKGLN-----VLESAELRNGIKTFSNWPTIPQLYVKG-----EFVGGCDIVREMFQA--GELQQLLSEKGVAVAA--------------------- |
| Q6N9E3 | UniRef cluster | -------------------MTVRPGRWWSAGRAAKGFAMSIEQFIDNEVKANDVVLFMKGTPQFPQCGFSGQVVQILDHIGVPYKGHN-----VLENAELRDGIKVYSNWPTIPQLYVKG-----EFVGGCDIVREMFQA--GELQKLFTDKGIKAAA--------------------- |
| Q218M5 | UniRef cluster | -------------------------MTRRRPVPQKDPIMSIEQFIESEVKSNDVVLFMKGTPQFPQCGFSGQVVQILDHVGVGYKGLN-----VLESADLRDGIKTYSNWPTIPQLYVKG-----EFVGGCDIVREMFQA--GELQQLLADKGITVAASA------------------- |
| B0IQY1 | UniRef cluster | -------------------MTVRPGRWWSAGRAAKGFAMSIEQFIDNEVKANDVVLFMKGTPQFPQCGFSGQVVQILDHIGVPYKGHN-----VLENAELRDGIKVYSNWPTIPQLYVKG-----EFVGGCDIVREMFQA--GELQKLFTDKGIKAAA--------------------- |
| A3XBY4 | UniRef cluster | -------------------------------------MSDAQTRIDETVKANDVVLFMKGNKEMPQCGFSSRVAGVLNYIGVDYTDVN-----VLADEEIRAGIKEYSDWPTIPQLYIKG-----EFVGGCDIITEMTLS--GELDGMFADNEIAFDKEAADKIREANG---------- |
| Q2KZY7 | UniRef cluster | -------------------------------------MSDVQNFIRDTVTQHPVVLFMKGTAQFPQCGFSGRAIQILKACGVK--KLVTVN--VLEDDEVRQGIKTFSNWPTIPQLYVQG-----EFIGGSDIMNEMNES--GELKTLLDQSGATA----------------------- |
| Q2JWT2 | UniRef cluster | -----------------------------------MLDPLLEEKIRNQVRTHKVLIYMKGTPEMPLCGFSYAAVRILDSLGFPYTAIN-----VLEDPELRQGIKEFSNWPTIPQIYIDG-----EFVGGCDILQEMHAR--NELRPLIEAAFAKAGTQS------------------- |
| A9AJ70 | UniRef cluster | --------------------------------------MDTQQRIKQIVDENPVVLFMKGNAQFPMCGFSGRAVQVLKACGVD--QFKTVN--VLEDEEIRQGIKEFSNWPTIPQLYVKG-----EFIGGSDIMMEMYQS--GELQQLFAAA--------------------------- |
| A7IMR7 | UniRef cluster | ----------------------MKHAGPHSADQNGFETMGIREDIDAIVKSGDVVLFMKGTPQFPQCGFSGQVVQILDHVGVPFKGVN-----VLENDAVRQGIKDYANWPTIPQLYIKG-----EFVGGCDIVREMFQA--GELTTFLEEKGVAIRDRAIG----------------- |
| A4EKD9 | UniRef cluster | --------------------------------------MTAQDQIKETVTSNDVVLFMKGTKSMPQCGFSSRVAGVLNFMGVDFNDVN-----VLADDALRQGIKDYSDWPTIPQLYVKG-----EFVGGCDIITEMTLS--GELDTLFAENGVTYDKDAAHKIREANA---------- |
| A3SY71 | UniRef cluster | -------------------------------------MTDAKTQIQETITANHVVLFMKGNKTMPQCGFSSRVAGVLNYMNVDYTDVN-----VLADEELRQGIKDFSDWPTIPQLYVKG-----EFVGGCDIITEMMLS--GELDTLLEDNGVAFDKEAADKIREANG---------- |
| A3SD28 | UniRef cluster | -------------------------------------MTDAKTQIQETITANNVVLFMKGNKTMPQCGFSSRVAGVLNYMNVDYTDVN-----VLADEELRQGIKDFSDWPTIPQLYVKG-----EFVGGCDIITEMMLS--GELDTLLEDNGVAFDKDAADKIREANG---------- |
| A1W4B6 | UniRef cluster | -------------------------------------MSDVQQRIDQLVKTNDILLFMKGNASFPMCGFSGRAVQILKACGVDPKNIATVN--VLDDQEIRQGIKDYSHWPTIPQLYVKG-----EFIGGSDIMMEMYES--GELQQVLGTGDAA------------------------ |
| Q11IV6 | UniRef cluster | -------------------------------------MSGINEFIDNEVKTNDVVLFMKGTPGFPQCGFSGQVVQILDYLGVDYKGIN-----VLTSDELRQGIKEYSSWPTIPQLYVKG-----EFVGGCDIVREMFQA--GEMQSFLEEKGVSVRGAA------------------- |
| Q0EWW1 | UniRef cluster | ------------------------------------MTDSALKQIDQVVKEHDIVLFMKGTPDFPQCGFSQRVAGILNEYELPYAAVN-----VLLSDAVRQGIKEYSDWPTIPQLYVKG-----EFIGGCDIVSEMHAS--GELKELLAHLQKDA----------------------- |
| Q051X1 | UniRef cluster | ------------------------------------MNEEVKQKINDLIGTSKVFLFMKGTPEAPMCGFSAGVSNVLRSLGISFGSFN-----VLSDETMRQGIKDYANWPTIPQLYIDG-----EFIGGHDIVVEMAKS--GDLQKKAGILNS------------------------- |
| Q04RQ0 | UniRef cluster | ------------------------------------MNEEVKQKINDLIGTSKVFLFMKGTPEAPMCGFSAGVSNVLRSLGISFGSFN-----VLSDETMRQGIKDYANWPTIPQLYIDG-----EFIGGHDIVVEMAKS--GDLQKKAGILNS------------------------- |
| A3VDF2 | UniRef cluster | --------------------------------------MTAEDTIKSTVTSNDVVLYMKGTKEMPQCGFSSRVAGVLNFMGVEFTDVN-----VLADDQIRQGIKDYSDWPTIPQLYVKG-----EFVGGCDIITEMTLS--GELDKLFDDEGITYSKKAAEKIREANG---------- |
| A0HFR3 | UniRef cluster | -------------------------------------MSNTQQRIDDLVKNNDILLFMKGNASFPQCGFSGRAIQILKACGVDAKSIATVN--VLEDQEIRQGIKDYSQWPTIPQLYIKG-----EFIGGSDIMMEMYES--GELQQVLAAK--------------------------- |
| Q2SUG0 | UniRef cluster | --------------------------------------MDTQQRIKQIVDENPVVLFMKGTAQFPMCGFSGRAVQVLKACGVD--QFKTVN--VLEDEEIRQGIKAFSNWPTIPQLYVKG-----EFVGGSDIMMEMYQS--GELQQLFTA---------------------------- |
| Q21YZ3 | UniRef cluster | -------------------------------------MSDTQQRIDDLVKNNEVLLFMKGNASFPQCGFSGRAIQILQACGVDPKTLKTVN--VLEDEEIRAGIKDYSNWPTIPQLYIKG-----EFIGGADIMMEMYQN--GELQQALAAKAS------------------------- |
| A6U9M2 | UniRef cluster | -------------------------------------MSGINDFIDAEVKTNDVVLFMKGTPQFPQCGFSGQVVQILDYVGVDYKGIN-----VLADADLRQGIKDYSSWPTIPQLYVKG-----EFVGGCDIVREMFQA--GELQSLLQGQGISVKGAA------------------- |
| Q8F4D2 | UniRef cluster | ------------------------------------MNNDLKQKIDGLIGSNKVFLFMKGTPEAPMCGFSAGVSNVLRSLGIQFGSFN-----VLSDETIRQGIKEYANWPTIPQLYING-----EFIGGHDIVVEMAKT--GDLQKKAGILSAG------------------------ |
| Q72RD7 | UniRef cluster | ------------------------------------MNNDLKQKIDGLIGSNKVFLFMKGTPEAPMCGFSAGVSNVLRSLGIQFGSFN-----VLSDETIRQGIKEYANWPTIPQLYING-----EFIGGHDIVVEMAKT--GDLQKKAGILSAG------------------------ |
| Q5F9P2 | UniRef cluster | -------------------------------------MASIHDQIKEVVTTHRVVLFMKGTKQFPQCGFSSRAVQILN--AAGCTDYVAVN--VLENPEVRQGIKEYSDWPTIPQLYVNG-----EFVGGSDILMEMYEA--GELQELLKA---------------------------- |
| Q3YRC2 | UniRef cluster | ------------------------------------MKNDIMDKIKHDIENNDVVLYMKGDADFPQCGFSSVVVSILKKMKVNFKSIN-----VLEDLELREAIKEFTNWPTIPQLYVKG-----EFIGGCDIVKEMYHS--GELQELFSKNNLISTQ--------------------- |
| Q0AQX3 | UniRef cluster | ------------------------------------MSATAHDSIKSTIDGNEVVLFMKGTPVFPQCGFSSVVARVLDHLQVNFESVN-----VLEDDGIRQGIKEFSNWPTIPQLYVKG-----EFVGGCDIIKEMFET--GELQAYFKDKGVLADA--------------------- |
| A4SV41 | UniRef cluster | --------------------------------------MDTQAQIKEIVTSHPVVLFMKGNAQFPQCGFSGNAINILKASGVE--KLHTVN--VLEDEGIRQGIKQYANWPTIPQLYING-----EFIGGSDIMTEMFQS--GELQKLVKG---------------------------- |
| A1WI58 | UniRef cluster | -------------------------------------MSDTRQRLDALVKSSDILLFMKGNASFPMCGFSGRAIQILKACGVDPKAVVTVN--VLEDEAIRQGIKEYSNWPTIPQLYVKG-----EFIGGSDIMMQMYES--GELRQVLGT---------------------------- |
| Q7CYD6 | UniRef cluster | -------------------------------------MSGIHDIIDSEVKSNDIVLFLKGTPQFPQCGFSGQVVQILDYLGVEYKGVN-----VLADADIRQGIKDYSNWPTIPQLYIKG-----EFVGGCDIVKEMFQS--GELQSHFQEQGISVRGAA------------------- |
| Q0K6F9 | UniRef cluster | ----------------MASHSPQAAPATRHRPTEKQTMSDVQQKIDQIVKSNPVVLFMKGTAQFPMCGFSGRAIQILKACGVD--APTTVN--VLDDEGIRQGIKEYANWPTIPQLYVNG-----EFIGGSDIMMEMYQN--GELQTLLKG---------------------------- |
| A8LLS4 | UniRef cluster | -------------------------------------MSDANERIKETVTKNDVVLFMKGTKTMPQCGFSSRVAGVLNFMGVEYTDVN-----VLADEEIRQGIKDYSDWPTIPQLYVKG-----EFVGGCDIITEMTLS--GELDQMFETNAVSYNKEAADKIREANA---------- |
| A4TUN6 | UniRef cluster | ------------------------------------MSNPVFERIQQDLSENDVVVYMKGTPMFPQCGFSAAVVQVLTALGVKFKGID-----ILVDPSLREGIKQYTNWPTLPQLYVKG-----EFVGGCDIVREMAGN--GELAALLKDKGVPSNA--------------------- |
| A0NP27 | UniRef cluster | --------------------------------------MSIQDWIKNEVDTNDVVLFMKGTPNFPQCGFSGQVVQILDYVGAPYKGIN-----VLEDDDLRQGIKEFTNWPTIPQLYVKG-----EFVGGCDIIREMFQN--QELQGLLTEKGIATTQQTA------------------ |
| Q7NG33 | UniRef cluster | ------------------------------------MSQSTHEKIDSLVKNNKVLIFMKGTPQFPQCGFSAASVQILSSLGHPFEAVN-----VLDDFEIRQGIKDYANWPTIPQVYVDG-----EFVGGCDILIEMHNR--GELKPLLDQAFEGEGAKA------------------- |
| Q46WS6 | UniRef cluster | -------------------------------------MSDVQQKIDQIVKGNPVVLFMKGTAQFPMCGFSGRAIQILKACGVD--SPATVN--VLDDEGIRQGIKEYANWPTIPQLYVNG-----EFIGGSDIMMEMYQN--GELQTLLKG---------------------------- |
| Q2GHG0 | UniRef cluster | ------------------------------------MTNDIMDKIKHDIENNDVVLYMKGDADFPQCGFSSVVVSILKKMNVKFKSIN-----VLESLELREAIKEFTNWPTIPQLYVKG-----EFIGGCDIVKEMYHN--GELQELLSKNNLIVAQ--------------------- |
| Q40IN5 | UniRef cluster | ------------------------------------MTNDIMDKIKHDIENNDVVLYMKGDADFPQCGFSSVVVSILKKMNVKFKSIN-----VLESLELREAIKEFTNWPTIPQLYVKG-----EFIGGCDIVKEMYHN--GELQELLSKNNLIVAQ--------------------- |
| A9D6C1 | UniRef cluster | -------------------------------------MSGINEFIDNEVKTNDVVLFMKGTPQFPQCGFSGQVVQILDYLGVPYKGVN-----VLADDALRNGIKEFSNWPTIPQLYVKG-----EFVGGCDIIREMFQS--AELQQHLTDNGVPVKAA-------------------- |
| A7CHQ1 | UniRef cluster | MAGSKRRCAFLAKIEGFSNVDVSSGTRLPPLTQSSLPMSTTHEKIDQIVKGHPVVLFMKGTAQFPMCGFSGRAIQILKACGVD--RPHTVN--VLEDDEIRQGIKDYANWPTIPQLYING-----EFIGGSDIMMEMYQS--GELQPLLAA---------------------------- |
| A4WQY1 | UniRef cluster | ------------------------------------MTTTVEEQLRDLVTTNDVVLFMKGSKTMPQCGFSSRVAGVLNYMQVAYKDVN-----VLEDADIRQGIKDFSDWPTIPQLYVKG-----EFVGGCDIVTEMTLS--GELDQLFEAKGVTYDKAAADKIREANA---------- |
| Q7WQE7 | UniRef cluster | -------------------------------------MSDVQEFIRETVTQHPVVLFMKGTAQFPQCGFSGRAIQILKGCGVK--KLVTVN--VLEDDEVRQAIKTFSNWPTIPQLYVGG-----EFIGGSDIMSEMNES--GELKQLLDDSGATA----------------------- |
| Q7WCE1 | UniRef cluster | -------------------------------------MSDVQEFIRETVTQHPVVLFMKGTAQFPQCGFSGRAIQILKGCGVK--KLVTVN--VLEDDEVRQAIKTFSNWPTIPQLYVGG-----EFIGGSDIMSEMNES--GELKQLLDDSGATA----------------------- |
| Q7W021 | UniRef cluster | -------------------------------------MSDVQEFIRETVTQHPVVLFMKGTTQFPQCGFSGRAIQILKGCGVK--KLVTVN--VLEDDEVRQAIKTFSNWPTIPQLYVGG-----EFIGGSDIMSEMNES--GELKQLLDDSGATA----------------------- |
| A2WD21 | UniRef cluster | --------------------------------------MDTQQRIKQIVDENPVVLFMKGNAQFPMCGFSGRAVQVLKACGVD--QFKTVN--VLEDEEIRQGIKAFSNWPTIPQLYVKG-----EFIGGSDIMMEMYQS--GELQQLFAAA--------------------------- |
| Q8YGM9 | UniRef cluster | -----------------MGDILPSCAGIWAGILKGNQMTGINDIIDNEVKNNDVVLFMKGTPGFPQCGFSGQVVQILDYLGVNYKGVN-----VLASDELRQGIKEYSSWPTIPQLYVKG-----EFVGGCDIVREMFQS--KELQALFNEKGIATKAA-------------------- |
| Q8G185 | UniRef cluster | -----------------MGDILPSCAGIWAGILKGNQMTGINDIIDNEVKNNDVVLFMKGTPGFPQCGFSGQVVQILDYLGVNYKGVN-----VLASDELRQGIKEYSSWPTIPQLYVKG-----EFVGGCDIVREMFQS--KELQALFNEKGIATKAA-------------------- |
| Q7DDL4 | UniRef cluster | -------------------------------------MASIHDQIKEVVTTHRVVLFMKGTKQFPQCGFSSRAVQILN--AAGCTDYVTVN--VLENPEVRQGIKEYSDWPTIPQLYVNG-----EFVGGSDILMEMYEA--GELQELLKA---------------------------- |
| Q57DS0 | UniRef cluster | -----------------MGDILPSCAGIWAGILKGNQMTGINDIIDNEVKNNDVVLFMKGTPGFPQCGFSGQVVQILDYLGVNYKGVN-----VLASDELRQGIKEYSSWPTIPQLYVKG-----EFVGGCDIVREMFQS--KELKALFNEKGIATKAA-------------------- |
| Q2YNG8 | UniRef cluster | -----------------MGDILPSCAGIWAGILKGNQMTGINDIIDNEVKNNDVVLFMKGTPGFPQCGFSGQVVQILDYLGVNYKGVN-----VLASDELRQGIKEYSSWPTIPQLYVKG-----EFVGGCDIVREMFQS--KELKALFNEKGIATKAA-------------------- |
| B0CLG2 | UniRef cluster | -------------------------------------MTGINDIIDNEVKNNDVVLFMKGTPGFPQCGFSGQVVQILDYLGVNYKGVN-----VLASDELRQGIKEYSSWPTIPQLYVKG-----EFVGGCDIVREMFQS--KELQALFNEKGIATKAA-------------------- |
| A9MAL2 | UniRef cluster | -------------------------------------MTGINDIIDNEVKNNDVVLFMKGTPGFPQCGFSGQVVQILDYLGVNYKGVN-----VLASDELRQGIKEYSSWPTIPQLYVKG-----EFVGGCDIVREMFQS--KELQALFNEKGIATKAA-------------------- |
| A9M3K4 | UniRef cluster | -------------------------------------MASIHDQIKEVVTTHRVVLFMKGTKQFPQCGFSSRAVQILN--AAGCTDYVTVN--VLENPEVRQGIKEYSDWPTIPQLYVNG-----EFVGGSDILMEMYEA--GELQELLKA---------------------------- |
| A8I8B7 | UniRef cluster | --------------MPPQARCWGMGTGPTPANHSGPKTMSIRDFIDNEVKNNDVVVFMKGTPQFPQCGFSGQVVQILDHLGVSYKGIN-----VLSSDELRQGIKDYANWPTIPQIYVKG-----EFVGGCDIVREMFQA--GELVPFFEEKGVALHDRAIG----------------- |
| A5VQ13 | UniRef cluster | -----------------MGDILPSCAGIWAGILKGNQMTGINDIIDNEVKNNDVVLFMKGTPGFPQCGFSGQVVQILDYLGVNYKGVN-----VLASDELRQGIKEYSSWPTIPQLYVKG-----EFVGGCDIVREMFQS--KELQALFNEKGIATKAA-------------------- |
| A3YYM1 | UniRef cluster | ------------------------------------MDSAVKQRIDSLLSSSPIVVFMKGSKLMPQCGFSNNVVQILNSLGLPFETFD-----VLSDQEIRQGIKEYSEWPTIPQVYVNG-----EFIGGSDILIEMYNS--GELRETLTVALAS------------------------ |
| A1KT33 | UniRef cluster | -------------------------------------MASIHDQIKEVVTTHRVVLFMKGTKQFPQCGFSSRAVQILN--AAGCTDYVTVN--VLENPEVRQGIKEYSDWPTIPQLYVNG-----EFVGGSDILMEMYEA--GELQELLKA---------------------------- |
| A1IR23 | UniRef cluster | -------------------------------------MASIHDQIKEVVTTHRVVLFMKGTKQFPQCGFSSRAVQILN--AAGCTDYVTVN--VLENPEVRQGIKEYSDWPTIPQLYVNG-----EFVGGSDILMEMYEA--GELQELLKA---------------------------- |
| Q8XVC7 | UniRef cluster | -------------------------------------MSTTHEKIDQIVKSHPVVLFMKGTAQFPMCGFSGRAVQILKACGVD--QPYTVN--VLEDDEIRQGIKEYANWPTIPQLYVKG-----EFIGGSDIMMEMYQS--GELQPLLAA---------------------------- |
| Q2GLI7 | UniRef cluster | ------------------------------------MDNNLVNRIKHDIENNDVVLYMKGTATAPQCGFSSVVASVLVHLGIAFKDVD-----VLRDPELREGIKEFSNWPTIPQLYVKG-----EFIGGCDIVREMYQS--GELQELLKKKGVITG---------------------- |
| A8IIQ9 | UniRef cluster | --------------------------------------MSTSDRINGLISENDIMVFMKGTPAMPRCGFSGAVVQILNTLGVPYATLD-----VLEDPFVREGIKEFTNWPTIPQVFVKG-----EFIGGCDIVREMAQK--GELQALFSEKGIAPRAA-------------------- |
| A8GUH6 | UniRef cluster | --------------------MVYNVTNKIKHIRFLMLENKSFKFIENEIKNNKVVLFMKGTKEAPMCGFSGKVVMILTKLGVEFRDID-----VLSDPELRESLKVFSDWPTFPQLYING-----ELVGGCDIVTELYSS--GELEKMLK----------------------------- |
| A6X1K1 | UniRef cluster | -------------------------------------MTGINDFIANEVKTNDVVLFMKGTPGFPQCGFSGQVVQILDYLGVEYKGVN-----VLSSDELRQGIKEYSSWPTIPQLYVKG-----EFVGGCDIIREMFQS--SELQALFTDKGIATKAA-------------------- |
| A4JB48 | UniRef cluster | --------------------------------------MDTQQRIKQIVDENQVVLFMKGNAQFPMCGFSGRAVQVLKACGVD--QFKTVN--VLEDDEIRQGIKEFSNWPTIPQLYVKG-----EFIGGSDIMMEMYQS--GELQQLFAAA--------------------------- |
| A3Z821 | UniRef cluster | ------------------------------------MDDQTKARIESLIQSSPIVVFMKGTKLMPQCGFSNNVVQILNSLGLPFETFD-----VLSDMEIRQGIKEFSDWPTIPQVYVKG-----EFIGGSDILIEMYNS--GELKEKLEVALAS------------------------ |
| A1HAF1 | UniRef cluster | -------------------------------------MSTTHEKIDQIVKGHPVVLFMKGTAQFPMCGFSGRAIQILKACGVD--RPHTVN--VLEDDEIRQGIKDYANWPTIPQLYVNG-----EFIGGSDIMMEMYQS--GELQPLLAA---------------------------- |
| Q98NP0 | UniRef cluster | -------------------------------------MSGINDYIDNEVKGNDVVLFMKGTPGFPQCGFSGQVVQILDYIGADYKGVN-----VLDSAELRQGIKDYSNWPTIPQLYVKG-----EFVGGCDIVREMFQA--GELQTFLVEKGVSVKGAA------------------- |
| Q2G5B8 | UniRef cluster | ------------------------------------MSNTTEERISEIVNGNDVVLFMKGTPLFPQCGFSSKAVAILEHLGVE---YATVD--VLQDMEIRQGIKSFSDWPTIPQLYVKG-----EFVGGSDIMMEMFQA--GELQQVMDDAGVKAA---------------------- |
| Q0BIQ2 | UniRef cluster | --------------------------------------MDTQQRIKQIVDENQVVLFMKGNAQFPMCGFSGRAVQVLKACGVD--QFKTVN--VLEDDEIRQGIKEFSNWPTIPQLYVKG-----EFVGGSDIMMEMYQS--GELQQLFAAA--------------------------- |
| A0G990 | UniRef cluster | --------------------------------------MDTQQRIKQIVDENSVVLFMKGNAQFPMCGFSGRAVQILKACGVD--QFKTVN--VLEDDAVRQGIKEFSNWPTIPQLYVNG-----EFVGGSDIMMEMYQS--GELQQLFAAA--------------------------- |
| Q2GEJ8 | UniRef cluster | -------------------------------------MKDIFAKIEGIIRRHDVVLFMKGTSGLPMCGFSGAVVNILKALDVTFYGVN-----VLEDPELREGIKKFADWPTIPQLYVKG-----EFIGGCDIVREMYEN--RELQTLFEDSLAG------------------------ |
| Q1YEA7 | UniRef cluster | -------------------------------------MTAINEWIDNEVKTNDVVVFMKGTPAFPQCGFSGQVVQILDYLGLEYKGVN-----VLSTDDLRQGIKAYSEWPTIPQLYVKG-----EFVGGCDIIREMFQA--GELQTFLVDKGIATTAPAPAAG--------------- |
| Q0VRM8 | UniRef cluster | --------------------------------------MDVIQVIKDQIEKNPVILYMKGSPQFPQCGFSAQVVEAMTAVGKPFAYVN-----ILEAPEIRQNLKEYANWPTYPQLWVGG-----ELVGGCDIIMDMYRS--GQLKEMVDGVAVESDDDA------------------- |
| A1U323 | UniRef cluster | -----------------MYNKRKGRLAAKLTPNQRNEFMDINETIKSQLEENPVILYMKGTPQAPQCGFSARTVQALMACGERFAFVN-----ILDNQELREALKVYSSWPTYPQLYING-----ELVGGCDIVLEMSES--GELAKMVKDAAGQAEA--------------------- |
| A5ENT2 | UniRef cluster | --------------------------------------MSTSERIQKLIDSNDIVLFMKGVPAAPQCGFSAAVAQILAKLDVSYESVN-----VLADPFIREGIKEFSNWPTIPQLYVKG-----EFVGGCDIVREMFQA--GELATLLADKGIKAPAS-------------------- |
| A4GJN1 | UniRef cluster | ------------------------------------MTDNVQNEIKNIVDQNDVVLFMKGTKDQPQCGFSNAVVNTLSFMNVNYKDVN-----ILESDELRQGIKDFTNWPTIPQLYIKG-----EFIGGCDIILDMHKS--GELASVFDTKGITHD---------------------- |
| Q2Y5P5 | UniRef cluster | --------------------------------------MDTQEIIKQQVTTHPVVLYMKGSPAAPQCGFSAHAVKILNACGVD--DLFAVD--VLAEPEFRQGIKDYSNWPTIPQLYVNG-----EFIGGSDIVSEMYQS--GELQKLLESAEAK------------------------ |
| Q6RJL3 | UniRef cluster | -------------------------------------MADIMEQIESAVKKNKVMIFMKGNRSFPQCGFSAATVAIFDQLGVPYETAD-----VLSDPELRDGIKRYSNWPTIPQVYIDG-----KFVGGCDIIRELHET--GELEPLVKAALAESATQ-------------------- |
| Q05Y05 | UniRef cluster | ------------------------------------MDSQTKERIEALIQTSPIFVFMKGTKLMPQCGFSNNVVQILNAMGMAFETFD-----VLSDMEIRQGIKEFSEWPTIPQVYVKG-----EFIGGSDILIEMYNS--GELKEKLEIALAS------------------------ |
| Q39K20 | UniRef cluster | --------------------------------------MDTQQRIKQIVDENQVVLFMKGNAQFPMCGFSGRAIQVLKACGVD--QFKTVN--VLEDDEIRQGIKAFSNWPTIPQLYVKG-----EFIGGSDIMMEMYQS--GELQQLFAAA--------------------------- |
| A9BP62 | UniRef cluster | -------------------------------------MSDTQQRIDQLVKSNNILLFMKGSASFPMCGFSGRAIQILKACGVETKDISTVN--VLEDQEIRQGIKDYSNWPTIPQLYVQG-----EFIGGSDIMMEMYES--GELQQLLAPKA-------------------------- |
| A4YYV0 | UniRef cluster | --------------------------------------MSTSERIQKLIDSNDIVLFMKGVPAAPQCGFSAAVAQILAKLEVPYESVN-----VLADPFIREGIKEFSNWPTIPQLYVKG-----EFVGGCDIVREMFQA--GELATLLADKGIKASAS-------------------- |
| A2SD43 | UniRef cluster | --------------------------------MTQTTSSDVQQRIDQLVKSHRVVLFMKGSAQFPMCGFSGRAVQILKACGVT--DLATVN--VLEDDGIRQGIKDYANWPTIPQLYVNG-----EFVGGSDIMAEMYQS--GELQPLLSTQA-------------------------- |
| GLRX2 | UniRef cluster | -----------------------------------MMENKNLKFIQNAIKKNKVVLFMKGTKEMPACGFSGTVVAILNKLGVEFSDIN-----VLFDTSLREDLKKFSDWPTFPQLYING-----ELVGGCDIVKELYQN--GELEKMLKDETKLIKN--------------------- |
| Q5FG21 | UniRef cluster | ------------------------------------MTNNIMNRIKHDIETNDVVLYMKGDANMPQCGFSSVVVTILKKMNISFKSIN-----VLEDQELREAIKEFTNWPTIPQLYVKG-----EFIGGCDIVKEMYHT--GELQELFVKNNLITAN--------------------- |
| Q2SL65 | UniRef cluster | --------------------------------------MDVIEVIKEQLTSNPIILFMKGTPNAPQCGFSARTVQALMACGEKFAYVN-----ILEHPEIREALKTYSNWPTYPQLYIKG-----ELVGGCDIVAELYES--GELQGMVKGAAVAE----------------------- |
| Q1BSA9 | UniRef cluster | --------------------------------------MDTQQRIKQIVDENQVVLFMKGNAQFPMCGFSGRAVQVLKACGVD--QFKTVN--VLEDDEIRQGIKAFSNWPTIPQLYVKG-----EFIGGSDIMMEMYQS--GELQQLFAAA--------------------------- |
| Q12F88 | UniRef cluster | ----------------------------------MSTTDTTQQRIDQIVKSSDVVLFMKGTAQFPMCGFSGRAIQVLKACGVN--KPVTVN--VLEDEEIRHGIKEYSNWPTIPQLYVKG-----EFIGGSDIMMEMYQN--GELQQVLGTPTA------------------------- |
| Q0G816 | UniRef cluster | -------------------------------------MSSMNEWIDNEVKSNDIVVFMKGTPSFPQCGFSGQVVQIMDYMGVEYKGVN-----VLTSDDLRQGIKAYSDWPTIPQLYVKG-----EFVGGCDIVREMFQA--GELKQFFEDNGIATTAPQPAAG--------------- |
| A2VTB3 | UniRef cluster | --------------------------------------MDTQQRIKQIVDENQVVLFMKGNAQFPMCGFSGRAVQVLKACGVD--QFKTVN--VLEDDEIRQGIKAFSNWPTIPQLYVKG-----EFIGGSDIMMEMYQS--GELQQLFAAA--------------------------- |
| A2BX98 | UniRef cluster | ------------------------------------MENLTKNKIQKLIDSSPVMVFMKGTKLMPQCGFSNNVVQILNSLGVEFDTFD-----VLSDFEIREGIKEYSEWPTIPQVYLKG-----EFLGGSDILIEMYNS--GTLKEKIEIELAS------------------------ |
| A0K435 | UniRef cluster | --------------------------------------MDTQQRIKQIVDENQVVLFMKGNAQFPMCGFSGRAVQVLKACGVD--QFKTVN--VLEDDEIRQGIKAFSNWPTIPQLYVKG-----EFIGGSDIMMEMYQS--GELQQLFAAA--------------------------- |
| Q6FZI7 | UniRef cluster | -------------------------------------MTTVHDFIDNEIKTNDVILFMKGIPEAPQCGFSGQVVQILDYLGLKYKGIN-----ILTSDELRQGIKEYSNWPTIPQLYIKG-----EFIGGCDIVKEMFQS--NELQELLKEKKISFNLL-------------------- |
| Q5HAI8 | UniRef cluster | ------------------------------------MTNNIMDRIKHDIETNDVVLYMKGDANMPQCGFSSVVVTILKKMNISFKSIN-----VLEDQELREAIKEFTNWPTIPQLYVKG-----EFIGGCDIVKEMYHT--GELQELFVKNNLITAN--------------------- |
| Q3AJ22 | UniRef cluster | ------------------------------------MDPSTKARIETLVASSPIFVFMKGSKLMPQCGFSNNVVQILHSLGVTFETFD-----VLSDPEIRQGIKEFSSWPTIPQVYVKG-----EFIGGSDILIEMYNS--GELREKLEIALAS------------------------ |
| Q0FG89 | UniRef cluster | ------------------------------------MSDTAQSTIKETIEKNDVVLFMKGTSSMPQCGFSSRIAGVLNFLNISWLDIN-----VLADENLRQGIKDFSDWPTIPQMYVKG-----EFVGGCDIITDMMLT--GELDKLLETNNIKFDKDAADKVREANS---------- |
| A9IVH8 | UniRef cluster | -------------------------------------MTTVHDFIDNEIKTNDVILFMKGTPEAPQCGFSGQVVQILDYLGLNYKGIN-----ILTSDELRQGIKEYSNWPTIPQLYVKG-----EFIGGCDIVKEMFQN--NELQELLKEKNIPYNQS-------------------- |
| A8EZU5 | UniRef cluster | -----------------------------------MLENKNFEFIKSEVKNNKVVLFMKGTKETPMCGFSAKVVAILNKLDVEFRDIN-----VLADPELREDLKKFSDWPTFPQLYING-----ELVGGCDIVTELHNN--GALEKILKR---------------------------- |
| Q7NS07 | UniRef cluster | --------------------------------------MSIQQDIQQTVADNAVVLFMKGSAQFPQCGFSSRAVQILKACGVDN--FLTVD--VLRDPDIRQGIKDFSNWPTIPQLYVKG-----EFVGGSDIMYEMYQN--GELQDLLKDL--------------------------- |
| Q1GW92 | UniRef cluster | -----------------------------------MSDPATQARISKLVADHPVLLFMKGTPLFPQCGFSSRAIAMLDRLGVE---YETVD--VLQDMEIRQGIKEYSDWPTIPQLYVKG-----EFVGGSDIMMEMWEA--GELHQLMDGIPTRAQ---------------------- |
| Q13TU0 | UniRef cluster | --------------------------------------MDTQQRIKQIVDDNAVVLFMKGTAQFPMCGFSGRAIQILKACGVG--EIKTVN--VLEDDEVRQGIKQFSNWPTIPQLYVKG-----EFIGGSDIMMEMYES--GELQQLFAAA--------------------------- |
| B0T612 | UniRef cluster | --------------------------------MTDVASSPAIDFIAKTVAENPVVLFMKGVPDQPRCGFSSITVQILDHLGVS---FIGVD--VLQDDELRAGVKAFTDWPTIPQLYVKG-----EFIGGSDIVREMFQS--GELKTLLTEQGLIEA---------------------- |
| A5PB60 | UniRef cluster | -------------------------------------MSDVNSRISDIVKANDVVLFMKGTPLFPQCGFSSRAVAILDHCGVA---YDSVD--VLQDMEIRQGIKAYSDWPTIPQLYVKG-----EFLGGSDIMMEMFEA--GELQTLMDEKQVAKAS--------------------- |
| A1VKB4 | UniRef cluster | ----------------------------------MSHTDPTQQRIEQIVKSSDVVLFMKGTAQFPMCGFSGRAIQVLKACGVT--KPATVN--VLEDEGIRNGIKEYSNWPTIPQLYVKG-----EFVGGSDIMMEMYQN--GELQEMLGTPQA------------------------- |
| A0YTM6 | UniRef cluster | ------------------------------------MTPELQARIDNLVNSQKIFVFIKGTKLMPMCGFSNNVVGILNSLGVPFETCD-----VLEDPDIRQGIKEYSSWPTIPQVYING-----EFVGGSDVVIELYQK--GELQQMVEVAFAS------------------------ |
| A9BAZ5 | UniRef cluster | ------------------------------------MDSDTRSKIESLINSNPIMVFMKGTKLMPQCGFSNNVVQILNALGISFETFD-----VLSDMDIREGIKEFSNWPTIPQVYLKG-----EFLGGSDILIEMYNS--GELKEKLEIALAS------------------------ |
| A0L4I7 | UniRef cluster | -------------------------------------MSDIQATIQEHVNSHAVVLYMKGNPSFPQCGFSGAAAKMLAACDLSNDDFLAVD--VLMNPDIREGIKAFANWPTIPQLYIKG-----QFVGGADIMREMYQS--GDLKTQLDAALGR------------------------ |
| Q6G333 | UniRef cluster | -------------------------------------MTTIRDFIDSEIKENDVILFMKGTPDAPQCGFSGQVAHILNYLGVPYKGIN-----ILTSDELRQGIKDYSNWPTIPQLYVKG-----EFIGGCDIIKEMFQN--NELQELLKEKDIPCNKA-------------------- |
| Q4FLJ2 | UniRef cluster | ------------------------------------MDDSTKNLIQGHIETNEVCLFMKGTPDAPQCGFSMAVSNMLKILEVNYKGIN-----VLESQSLREGIKEFSDWPTIPQVYIKG-----EFVGGCDIVKEMYEN--GELKKVLEDKGINFKK--------------------- |
| Q479R9 | UniRef cluster | -------------------------------------MSDVQQRIHATVTGNPVVLYMKGDARFPQCGFSATAVQILKVCGVN--DFVTVN--VLADEEIRNGVKEYANWPTIPQLYIKG-----EFVGGCDIMKEMYQT--GELQQMLEGIAA------------------------- |
| Q1UZJ3 | UniRef cluster | ------------------------------------MDDSTKNLIQGHIETNEVCLFMKGTPDAPQCGFSMAVSNMLKILEVNYKGIN-----VLESQSLREGIKEFSDWPTIPQVYIKG-----EFVGGCDIVKEMYEN--GELKKVLEDKGINFKK--------------------- |
| A6F2A8 | UniRef cluster | --------------------------------------MDINETIKSQLEENAIILYMKGTPQAPQCGFSAKTVQALMACGERFAFVN-----ILDNQELREGLKVYSSWPTYPQLYIKG-----ELVGGCDIILEMSES--GELAEQVKAAAKQAEA--------------------- |
| A0GU79 | UniRef cluster | --------------------------------------MDTQQRIKQIVDENNVVLFMKGTAQFPMCGFSGRAIQILKACGVG--EIKTVN--VLEDDEVRQGIKQFSNWPTIPQLYVKG-----EFVGGSDIMMEMYES--GELQQLFAAA--------------------------- |
| Q0BRI2 | UniRef cluster | -------------------------------------MSDTASRIQADINENPVMLYMKGTAMFPQCGFSARVTQILNHLEVPYKTAN-----VLEDQALREGIKAFSNWPTIPQLYIKG-----EFIGGCDIVMEMFQS--GELQALLSEKGIPHKAAA------------------- |
| A1K445 | UniRef cluster | --------------------------------------MDIKDVIREQVTTHPVALYMKGVPQAPACGFSATAVQILKASGVK--DFFSVN--VLADDAVRQGIKEFSNWPTIPQLYVKG-----EFVGGADIMREMYQS--GEIQQLLKDAGVTA----------------------- |
| Q9A5E5 | UniRef cluster | --------------------------------MTDAATSPALDFIAKTVADHPVVVFMKGVPDQPRCGFSSVVVQILDHLGVE---FVGVD--VLQDEDLRQGVKTFTDWPTIPQLYVKG-----EFIGGSDIVREMFQS--GELKTFLTEQGVIAA---------------------- |
| Q73HQ5 | UniRef cluster | --------------------------------------MSNFEQIKKDITENDVVLYMKGTSDFPQCGFSGLVVSILKKLNVKFKYIN-----VLENDEIRESIKKFSDWPTIPQLYIKE-----EFIGGCDITREMYEK--GELQSLLKEKKIIAE---------------------- |
| Q4EBB3 | UniRef cluster | --------------------------------------MSNFEQIKKDITENDVVLYMKGTSDFPQCGFSGLVVSILKKLNVKFKYIN-----VLENDEIRESIKKFSDWPTIPQLYIKE-----EFIGGCDITREMYEK--GELQSLLKEKKIIAE---------------------- |
| A5GU93 | UniRef cluster | ------------------------------------MDAQLKSRIETLVASSPVFIFMKGSKLMPQCGFSNNVVQIFHSLGVPFETFD-----VLSDMEIRQGIKEFSNWPTIPQVYLNG-----EFLGGSDIMIEMYNS--GELRETVTVALAS------------------------ |
| Q46K48 | UniRef cluster | ------------------------------------MDSNTRSKIESLINSKPIFVFMKGNKLMPQCGFSNNVVQILNSLGMSFETFD-----VLSDMEIREGIKEYSNWPTIPQVYLKG-----EFMGGSDILISMYNS--GELKEKLEIALAS------------------------ |
| Q7P9P9 | UniRef cluster | -----------------------------------MLENKNFKFIENEIKNNKVVLFMKGIKKSPACGFSGTVVAILNKLGVEFRDIN-----VLFDAELREDLKKFSDWPTFPQLYING-----ELVGGCDIVRELYQS--GELEKMLKAYTR------------------------- |
| A6DQ54 | UniRef cluster | -------------------------------------MSDINATIKAELEAHPIKLFMKGVPAMPQCGFSQTVIQILSFYDVEYSSMN-----ILENPEFRQGLKDYFEWPTFPQLVVNG-----ELVGGCDIIMELHEN--GELQEVLDSAKK------------------------- |
| A2C3G8 | UniRef cluster | ------------------------------------MDSNTRSKIESLINSKPIFVFMKGNKLMPQCGFSNNVVQILNSLGMSFETFD-----VLSDMEIREGIKEYSNWPTIPQVYLKG-----EFMGGSDILISMYNS--GELKEKLEIALAS------------------------ |
| A3WHZ3 | UniRef cluster | -------------------------------------MADINTRISTLVGENDVVLFMKGTPLFPQCGFSSRAVAILDHCGVQ---YESVD--VLQDMEVRQGIKTFSDWPTIPQLYVKG-----EFVGGSDIMMEMFEA--GELQQMMDENKVAKIES-------------------- |
| A3IVB0 | UniRef cluster | ------------------------------------MNPETKEKIDQMVNDHKILVFMKGNKLMPQCGFSNNVVQILGTLGVPFETVD-----VLADPEIRQGIKEYSNWPTIPQVYING-----EFVGGSDIMIEMYQN--GELQQMVEVALAS------------------------ |
| Q6MJ95 | UniRef cluster | -------------------------------------MATTHERIDQIVKGNKIVLFMKGTQQFPMCGFSARACAILQDMGVQ---FHDVN--VLDDDEIRQGIKEYGNWPTIPQLYINH-----QLVGGSDIMMEMYQS--GELQELLK----------------------------- |
| Q1H4S1 | UniRef cluster | --------------------------------------MDTQALIKNQVTSNPVVLYMKGSPKFPQCGFSGLAVQILQACGLK--DFVAVD--VLADPAIREGIKTYANWPTIPQLYIKG-----EFIGGADIMRDLYQQ--GELQKLLQEAQA------------------------- |
| Q0AB38 | UniRef cluster | -------------------------------------MDQVQETIAKQVKENPILLYMKGSPQQPQCGFSMRAAQALAGCGKEFAYVD-----VLQDEAIRQGIKEFGNWPTIPQLYLNG-----ELVGGCDIIMEMYES--GDLQKMVDQAAESASN--------------------- |
| A2BRU0 | UniRef cluster | ------------------------------------MDNSTKDKIQKLIDSNPVMVFMKGTKLMPQCGFSNNVVQILNSLGVEFGTFD-----VLSDFAIREGIKEYSDWPTIPQVYLKG-----EFLGGSDILIEMYNS--GSLKEKIEIELAS------------------------ |
| GLRX2 | UniRef cluster | -----------------------------------MLENKNFKFIENEIKNNKVVLFMKGIKKSPACGFSGTVVAILNKLGVEFRDIN-----VLFDAELREDLKKFSDWPTFPQLYING-----ELVGGCDIARELYQS--GELEKMLKAYTR------------------------- |
| Q31AB4 | UniRef cluster | ------------------------------------MDNLTKDKIQKLIDSNPVMVFMKGTKLMPQCGFSNNVVQILNSLGIEFGTFD-----VLSDFEVREGIKKYSDWPTIPQVYLKG-----EFLGGSDILIEMYNS--GALKEKIEIELAS------------------------ |
| B0BV29 | UniRef cluster | -------------------------------MSLKMLENKNFKFIENEIKNNKVVLFMKGIKKSPACGFSGTVVAILNKLGVEFRDIN-----VLFDAELREDLKKFSDWPTFPQLYING-----ELVGGCDIARELYQS--GELEKMLKAYTR------------------------- |
| A8GTJ1 | UniRef cluster | -----------------------------------MLENKNFKFIENEIKNNKVVLFMKGIKKSPACGFSGTVVAILNKLGVEFRDIN-----VLFDAELREDLKKFSDWPTFPQLYING-----ELVGGCDIARELYQS--GELEKMLKAYTR------------------------- |
| A5GLI6 | UniRef cluster | ------------------------------------MDAQTKSRIEALISSSPVFVFMKGTKLMPQCGFSNNVVQILNALGITFETFD-----VLSDMDVRQGIKEFSDWPTIPQVYVKG-----EFMGGSDILIEMYNN--GELKEKLEIALAS------------------------ |
| A4CV24 | UniRef cluster | ------------------------------------MDAQTKTRIETLINSSPVFVFMKGTKLMPQCGFSNNVVQILNALGIGFETFD-----VLTDMEIRQGIKDFSDWPTIPQVYVKG-----EFMGGSDILIEMYNN--GELKDKLEIALAS------------------------ |
| A3PDL6 | UniRef cluster | ------------------------------------MDKLTKDKIQKLIDSNPVMVFMKGTKLMPQCGFSNNVVQILNSLGVEFGTFD-----VLSDFAIREGIKEYSDWPTIPQVYLKG-----EFLGGSDILIEMYNS--GSLKEKIEIELAS------------------------ |
| A3JJ30 | UniRef cluster | --------------------------------------MNVQEKILSQVTADTIVLYMKGKPQSPLCGFSATTVQVLNACGVQ--DFAAVD--VLADPEIRDGIKVYSNWPTIPQLYIKG-----EFVGGADIVREMYEQ--GELQKLLQSALA------------------------- |
| Q7V6Q1 | UniRef cluster | ------------------------------------MDPTTKTRIEALIQSSPIMVFMKGTKLMPQCGFSNNCVQILNSLGMSFETFD-----VLSDMEIRQGIKDYSNWPTIPQVYVKG-----EFIGGSDILIEMYNA--GELAEKLEIALNS------------------------ |
| Q3J8G3 | UniRef cluster | --------------------------------------MDTMEQIKQAVESNSIILFMKGSPQFPQCGFSGRTVQALQACGT-EFAYVD----VLANPEVRALLPQYSNWPTFPQLYING-----ELVGGCDIILELFES--GELQTKVQEVVPHTSEEG------------------- |
| A2C893 | UniRef cluster | ------------------------------------MDPTTKTRIEALIQSSPIMVFMKGTKLMPQCGFSNNCVQILNSLGMSFETFD-----VLSDMEIRQGIKDYSNWPTIPQVYVKG-----EFIGGSDILIEMYNA--GELAEKLEIALSS------------------------ |
| Q8YYP7 | UniRef cluster | ------------------------------------MTQETSEKINNLITQNKIMVFMKGTKLMPQCGFSNNVVQILNTLGVPFETIN-----VLEDQEIRQGIKEYSNWPTIPQVYING-----EFIGGSDILIELYQK--GELQQLVEVALAS------------------------ |
| Q7V0Y3 | UniRef cluster | ------------------------------------MENPTKNKIQNLIDLNPVMVFMKGTKLMPQCGFSNNVVQILNSLGVTFNTFD-----VLSDFEIREGIKEYSEWPTIPQVYLKG-----EFLGGSDILIEMYNA--GTLKEKIEIALAS------------------------ |
| Q7U7S5 | UniRef cluster | ------------------------------------MDDSTRSRIEALISSSTIFVFMKGSKLMPQCGFSNNVVQILHSLGVSFETFD-----VLSDMEIRQGIKDFSSWPTIPQVYVNG-----EFIGGSDILIEMYNA--GELKEKLEIALAS------------------------ |
| Q3MFM1 | UniRef cluster | ------------------------------------MTQETSEKISNLITQNKIMVFMKGTKLMPQCGFSNNVVQILNTLGVPFETIN-----VLEDQEIRQGIKEYSNWPTIPQVYING-----EFIGGSDILIELYQK--GELQQLVEVALAS------------------------ |
| Q3AVH5 | UniRef cluster | ------------------------------------MDASTKSRIEGLIATSPIFVFMKGSKLMPQCGFSNNVVQILHSLGVTFETFD-----VLSDMEIRQGIKEFSSWPTIPQIYVKG-----EFMGGSDILIEMYNS--GELKEKLEIALAS------------------------ |
| Q05ZT4 | UniRef cluster | ------------------------------------MDASTKSRIEGLIATSPIFVFMKGSKLMPQCGFSNNVVQILHSLGVAFETFD-----VLSDMEIRQGIKEFSSWPTIPQIYVKG-----EFMGGSDILIEMYNS--GELKEKLEIALAS------------------------ |
| A3J9V0 | UniRef cluster | --------------------------------------MDMNDTIKSQLEENSVILYMKGSPQAPECGFSAKTVQALMACGEKFAFVN-----ILDNPELREALKVHSSWPTYPQLYISG-----ELVGGCDIIMEMSEN--GELATAVKAACKKTEA--------------------- |
| Q1NC86 | UniRef cluster | ------------------------------------MTDAVQQRIAQIVSGHDVVLFMKGTPLFPQCGFSSRAIAILEHLGVG---YETVD--VLQDQAIRQGIKAYSDWPTIPQLYVKG-----EFVGGSDIMMEMYEA--GELSQLMTDQGVAPAN--------------------- |
| Q0IB15 | UniRef cluster | ------------------------------------MDSKTKERIQTLIQSSPIFVFMKGTKLMPQCGFSNNVVQILNALGMSFETFD-----VLSDMEIRQGIKDYSDWPTIPQVYVKG-----EFMGGSDILIEMYNN--GELKEKLEIELAS------------------------ |
| GLRX2 | UniRef cluster | -----------------------------------MTKNKNLEFIQNAIKKNKVVLFMKGTKEMPACGFSGTVVAILNKLGVEFSDIN-----VLFDTALREDLKKFSDWPTFPQLYING-----VLVGGCDIAKELYQN--GELEKMLKDVVV------------------------- |
| Q2NC43 | UniRef cluster | -------------------------------------MSDTNAKISGIVGENDVVLFMKGTPLFPQCGFSSRAVSILDHCGVK---YESVD--VLQDMEIRQGIKTYSDWPTIPQLFVKG-----EFLGGSDIMMEMFEA--GELQELLDEKDVARAE--------------------- |
| A8GPQ8 | UniRef cluster | -----------------------------------MLENKNFEFIENALKNNKVVLFMKGTQEAPMCGFSAKVVAILNKLGVEFRDIN-----VFVNPEFREDLKKFSDWPTFPQLYIKG-----ALVGGCDIATELYNN--GELEKILRE---------------------------- |
| Q8P7V8 | UniRef cluster | --------------------------------------MPVMERIQAEVEQHPIVLFMKGTPQFPMCGFSSRAVQALVAAGADQLRTVN----VLEEPEIRANLPRYSNWPTFPQLFIHG-----ELIGGCDITMELFEA--GELKRIVSEAYQP------------------------ |
| Q4UW91 | UniRef cluster | --------------------------------------MPVMERIQAEVEQHPIVLFMKGTPQFPMCGFSSRAVQALVAAGADQLRTVN----VLEEPEIRANLPRYSNWPTFPQLFIHG-----ELIGGCDITMELFEA--GELKRIVSEAYQP------------------------ |
| B0RRC6 | UniRef cluster | --------------------------------------MPVMERIQAEVEQHPIVLFMKGTPQFPMCGFSSRAVQALVAAGADQLRTVN----VLEEPEIRANLPRYSNWPTFPQLFIHG-----ELIGGCDITMELFEA--GELKRIVSEAYQP------------------------ |
| A8G5I1 | UniRef cluster | ------------------------------------MDNLTKDKIQKLIDSNPLMVFMKGTKLMPQCGFSNNVVQILNSLGVEFGTFD-----VLSDFAIREGIKEYSDWPTIPQVYLKG-----EFLGGSDILIEMYNA--GSLKEKIEIELAS------------------------ |
| Q8DKI5 | UniRef cluster | ----------------------MQFCNWSTQEEKRTMTPELHAKIDNLVKSNKIIVFMKGSKLMPQCGFSNNAVQILNALGVPYETVD-----VLEDFEIRQGIKEYSNWPTIPQVFING-----EFIGGSDILIELYQS--GELQQLVEVALAS------------------------ |
| Q7VBK7 | UniRef cluster | ------------------------------------MNLETRARIEDLINSHSIMVFMKGTKLMPQCGFSNNVVQILNALGKHFETFD-----VLSDMDIREAIKEYSNWPTIPQVYLKG-----EFLGGSDILIEMYNS--GELLEKLEIALAS------------------------ |
| Q5GRW0 | UniRef cluster | --------------------------------------MSNFEQIKKDITENDVVLYMKGTSDFPQCGFSGLVVSILKKLNVKFKYIN-----VLENDEIRQSIKKFSNWPTIPQLYIKG-----EFIGGCDITREIYEK--GELQSLLKEKKIVE----------------------- |
| A1WUZ9 | UniRef cluster | -----------------------------------MSDESVQQTIARQVSENPILLYMKGSPEQPMCGFSQRAAQALAGCGREFAYVD-----VLQDERIRQGIKDYGDWPTIPQLYIDG-----ELVGGCDIIMEMFDS--GELQQLVDAAGAGASG--------------------- |
| GLRX2 | UniRef cluster | -----------------------------------MTENKNFEFIENEIKNNKVVLFMKGTKEATMCGFSAKVVAILNKLGVEFRDIN-----VFVNPEFREDLKKFSDWPTFPQLYIKG-----ELVGGCDIATELYNN--GELEKMLKG---------------------------- |
| A5CX40 | UniRef cluster | --------------------------------------MDVMDRIQHQVDSAAVVLYMKGTPQFPQCGFSAKAAQTLTSTGVE-FAYVN----IFEDQEVFQSLPTFANWPTFPQIYFNS-----ELVGGGDIIVEMAEM--GTLKDEMKKASEKFDVK-------------------- |
| B0C3N0 | UniRef cluster | ------------------------------------MTPELKERLDSLVQTNKILVFMKGSKLMPQCGFSNNAVQILNSLGVPYETVD-----VLEDYDIRQGIKEYSNWPTIPQVYING-----EFVGGSDVLIELYQQ--GELQQLVEVALAS------------------------ |
| Q4BXJ4 | UniRef cluster | ------------------------------------MTPELKDRIDQLVNNNKILVFMKGAKLMPQCGFSNNVVQVLNSLGVSYETVD-----VLADEEIRQGIKEYSSWPTIPQVYING-----EFIGGADIVYEMYQK--GELQQMIEVALAS------------------------ |
| A5WE22 | UniRef cluster | ---------------------------------MTDQAQDIETLIRNQIKENPVLLYMKGTPQFPQCGFSAKAIEVLTQIGRPFAFVN-----ILENPEIRATLPKIANWPTFPQLWING-----ELMGGSDIILEMYQS--GELKPLVEEHSPAN----------------------- |
| A5EX60 | UniRef cluster | --------------------------------------MDTLERIKQQIASMPVLIYMKGTPDMPMCGFSAKAVQCLKDLETSFAYVN-----VLQDPEIRATLPKYANWPTFPQLWVNQ-----QLIGGCDIIVEMYEN--GELEKLLENVVWPESME-------------------- |
| A5CEX4 | UniRef cluster | ----------------------------MILYIIPMNKDNIFQLIEQQIKSNDIVLYMKGTIHAPKCGFSAIVANIIQELTKDFKNINCTYIDVLCDDDLRQGIKEYTDWPTIPQLYIQE-----EFIGGCDIVKEMYRN--GELKTLLLQKFKDIKDN-------------------- |
| Q1DAS0 | UniRef cluster | ------------------------------------MTPELKARLEQETRSHKIVLFMKGNALFPQCGFSARALQLLQPLG-QVHTVD-----VLADPEIRQGIKDFTNWPTIPQIFING-----QFVGGSDILMELAER--GELADLVAGKSPA------------------------ |
| A1AW51 | UniRef cluster | --------------------------------------MDVMDRIQQQVDSAAIILYMKGTPQFPQCGFSATAARTLASTGVE-FTYVN----IFEDQEVFQSLPVFADWPTFPQIYFNS-----ELVGGGDIIVEMADM--GTLKGEMEKASEKFDTK-------------------- |
| YC64L | UniRef cluster | ------------------------------------MNPETKARIDQLVTANKVMVFMKGTKLMPQCGFSNNVVQILNMLGIPFETLD-----VLADAEIRQGIKEYSNWPTIPQVYVNG-----EFVGGSDIMIELYQN--GELQEMLEVALAS------------------------ |
| A8YCZ6 | UniRef cluster | ------------------------------------MTPETKDRIDQLVQNNKVLVFMKGNKLMPQCGFSNNVIQILNILGVSYETVD-----ILQDQELRQGVKEYSNWPTIPQVYING-----EFIGGSDIMIELYQN--GELQQIVEVALAS------------------------ |
| Q8PJ66 | UniRef cluster | --------------------------------------MPVMERIQAEVEQHPIVLFMKGTPQFPMCEFSSRAVQALVAAGADQLRTVN----VLEEPEVRANLPRYSNWPTFPQLFIHG-----ELIGGCDITLELFEA--GELKRIVSEAYQP------------------------ |
| Q3BRQ6 | UniRef cluster | --------------------------------------MPVMERIQAEVEQHPIVLFMKGTPQFPMCEFSSRAVQALVAAGADQLRTVN----VLEEPEVRANLPRYSNWPTFPQLFIHG-----ELIGGCDITLELFEA--GELKRIVSEAYQP------------------------ |
| A1UT49 | UniRef cluster | -------------------------------------MIPTHDFIDNEIKTNDVILFMKGTPSSPQCGFSGQVVQILDYLGLDYKGID-----VLASCELRQEIKNYSNWPTIPQLYIKG-----EFIGGCDIVKEMFQC--NELQNLLKEKNIPFSAS-------------------- |
| Q606E0 | UniRef cluster | --------------------------------------MDVIENIKRQIADNPVILYMKGTPDFPQCGFSGRAVQILDKCGVEYAFVN-----VFEAPEVRENLKLVSHWPTFPQLFVRG-----ELVGGSDIMMELYES--GELQKLLAGATKAGERQD------------------- |
| Q5N524 | UniRef cluster | -----------------------------------MMTPELQERLTSIINGDKIVVFMKGNKLMPQCGFSNNVVQILNILGVPFTTVD-----VLADYDIRQGIKEFSNWPTIPQVYVNG-----EFIGGSDILIELYQN--GELQQMLEVALAS------------------------ |
| Q31P44 | UniRef cluster | -----------------------------------MMTPELQERLTSIINGDKIVVFMKGNKLMPQCGFSNNVVQILNILGVPFTTVD-----VLADYDIRQGIKEFSNWPTIPQVYVNG-----EFIGGSDILIELYQN--GELQQMLEVALAS------------------------ |
| Q9Z3F7 | UniRef cluster | ------------------------------------MTPELQERLTSIINGDKIVVFMKGNKLMPQCGFSNNVVQILNILGVPFTTVD-----VLADYDIRQGIKEFSNWPTIPQVYVNG-----EFIGGSDILIELYQN--GELQQMLEVALAS------------------------ |
| Q8KLY3 | UniRef cluster | -----------------------------------MQVLDTEARIRQQLAEHPVLLYMKGTPGVPECGFSRAAVEALKNSGRPFATVN-----VLLAPHIREKLPKISQWPTYPQLFLRG-----ELVGGCDIILGLEAD--GSLVARLDAALAAES---------------------- |
| Q4IU14 | UniRef cluster | -----------------------------------MQVQDTEARIRKQIAENPVILYMKGTPAAPECGFSRAAVGALSKAGKPFAYVN-----VLTAPHIREKLPKLFQWPTFPQLFVNG-----ELIGGSDIILEMEAD--GSLKELLEKAVPQA----------------------- |
| Q2BIL4 | UniRef cluster | --------------------------------------MSVIDTIKEQIDNNDILLYMKGTPRFPQCGFSSRASEAVMGCGERFAFVN-----ILENPEIRAELPKYANWPTFPQLWVKG-----ELIGGCDIICEMAAN--GELAELIKEASAGSEEQSAE----------------- |
| A4VJ45 | UniRef cluster | -----------------------------------MQVLDTEARIRQQLAEHPVLLYMKGTPGVPECGFSRAAVEALKNSGRPFATVN-----VLLAPHIREKLPKISQWPTYPQLFLRG-----ELVGGCDIILGLEAD--GSLVARLDAALAAES---------------------- |
| B0JGE8 | UniRef cluster | ------------------------------------MTPETKARIDQLVQNNKVLVFMKGNKLMPQCGFSNNVIQILNILGVSYETVD-----ILQDQELRQGVKEYSNWPTIPQVYING-----QFIGGSDIMIELYQN--GELQQIVEVALAS------------------------ |
| Q60C52 | UniRef cluster | -------------------------------------MSTIEDTIRDQIAKHPVLLYMKGVPEMPQCGFSAKAVSCLQAAGVPFAYVN-----VLAAPSIRETLPSVSQWPTFPQLFVGG-----ELVGGSDIVSELAAS--GELKTLLEAAAAGQAQG-------------------- |
| Q5GXW0 | UniRef cluster | --------------------------------------MLVMERIQAEVEQHPIVLFMKGTPQFPMCEFSSRAVQALVAAGADQLRTIN----VLEEPEVRANLPRYSNWPTFPQLFIHG-----ELIGGCDITLELFEV--GELKRIVSEAYQP------------------------ |
| A3M620 | UniRef cluster | ---------------------------------MTEQARDTEALIRDQIAKHPVLLYMKGTPQFPQCGFSARAVEALSQIGRPFAYVN-----ILENPDIRATLPKIANWPTFPQLWVNG-----ELIGGSDIMLEMFQN--GELKPLIEQYSAAPEA--------------------- |
| Q8D2M0 | UniRef cluster | -------------------------------------MKNSIEKIKEQIKKNKIILYMKGSPENPKCGFSSKVARILCESNFRFAYVD-----VISNPHIREKLPEFSNWPTFPQLWVNG-----SLVGGCDIVTKMHEK--GELVPFINKEINK------------------------ |
| Q3SG04 | UniRef cluster | --------------------------------------MTPLDRIREQVTNNAIVLYMKGTPQFPQCGFSSRAAQVLQACGVKDFLAVN----VLADPEIFENLKYYANWPTFPQLYVKG-----ELIGGSDIMIEMYQK--GEIQKLLEEAQTA------------------------ |
| GLRX4 | UniRef cluster | -------------------------------------MNDIIKKIQNQIQNNPIIIYMKGSPDAPSCGFSAQAVHAISSCGKKFAYID-----VLKNPDIRLELPKYANWPTFPQLWVNG-----ELIGGCNIILELFQK--GELKKTISICDKLNS---------------------- |
| Q4FSB6 | UniRef cluster | ----------------------------MSEQTPNTAANDIEQLIRNQIKDNKVILYMKGSPQFPQCGFSAKAIEVLTQIGRPFAFVN-----ILENPEIRATLPQIANWPTFPQLWIDG-----ELMGGSDIILQMYQS--GELKPLVEANSPAA----------------------- |
| Q1QBM2 | UniRef cluster | ----------------------------MSEQTPNTAANDIEQLIRNQIKDNKVILYMKGSPQFPQCGFSAKAIEVLTQIGRPFAFVN-----ILENPEIRATLPQIANWPTFPQLWIDG-----ELMGGSDIILQMYQS--GELKPLVEANSPAA----------------------- |
| Q2P0Y2 | UniRef cluster | --------------------------------------MLVMERIQAEVEQHPIVLFMKGTPQFPMCEFSSRAVQALVAAGADQLRTIN----VLEEPEVRANLPRYSNWPTFPQLFIHG-----ELISGCDITLELFEV--GELKRIVSEAYQP------------------------ |
| Q118G0 | UniRef cluster | ----------------------------------MTLTPELKAKIDDLVTKNKIMVFMKGNKLMPQCGFSNNVVQILNILGVTYETCD-----VLENQDIRTGIKEYSNWPTIPQVYVDG-----EFLGGSDVMIEMYNNKKEELEQKLAVASAS------------------------ |
| GLRX4 | UniRef cluster | --------------------------------------MSIFQKIKKQIQDNIILIYMKGTPEAPSCGFSAQAVQALSFCGEKFAYVN-----ILENPDIRSELPKYANWPTFPQLWIDG-----ELIGGCSIILEMLEN--GELKKLILKVKKKYQT--------------------- |
| A6W0R2 | UniRef cluster | -------------------------------------MSNTIETIKEQISSNDILLYMKGNPRAPQCGFSSQAVQALMSCGERFAFVN-----ILDNPDIRAELPKFANWPTFPQLWVKG-----ELVGGCDIIVEMAAN--GELQTLIKDAVGSSEE--------------------- |
| A0Z4U1 | UniRef cluster | --------------------------------------MDIMETIKEQVTSNRVILYMKGSPNQPQCGFSARAVQALASVGERFAYVD-----ILSNPEIRANLPIYANWPTFPQLWVDG-----ELIGGCDIIAEMETS--GELAEAIKAGASDQA---------------------- |
| Q6D5W1 | UniRef cluster | ------------------------------------MTTPTIEKIQRQIAENPILLYMKGSPKLPSCGFSAQTVQALSSCGERFAYVD-----ILQNPDIRAELPKYANWPTFPQLWVDG-----ELVGGCDIVVEMFQR--GELQQLIKETADKYKAQQADQE--------------- |
| A3Y9J3 | UniRef cluster | ---------------------------------------------------------MKGTPRMPQCGFSAQTVQALMSCGERFAFVN-----ILENPEIRAELPKYANWPTFPQLWVNG-----ELIGGCDIVVEMANT--GELAPIIKAATGESDDASE------------------ |
| A1FSQ7 | UniRef cluster | -----------------------------------------MQQIQAEVDRYPLVLFMKGTPQYPMCGFSSRAVQALMAAGAVTLRTVN----VLEEPEIRANLPRFSNLPTFPQLFING-----ELIGGCDIVMELFEA--GELKRIVEEATQG------------------------ |
| A0ZBC5 | UniRef cluster | ------------------------------------MTPETKEKIDNLVQQNKIMVFMKGNKLMPQCGFSNNVVQILNTLAVPFETVD-----VLSDAEIRQGIKEYSNWPTIPQVYIDG-----QFVGGSDILIELYQK--GELQQLVEVALAS------------------------ |
| Q1YSW4 | UniRef cluster | --------------------------------------MDIMETIREQVENNAIILYMKGSPNQPQCGFSARTVQALMECGQRFAYVD-----ILSNPEIRANLPAYGNWPTFPQLWIKG-----ELIGGCDIIADMHQK--GELKPIMDEAAPAPEA--------------------- |
| Q1N4F7 | UniRef cluster | -------------------------------------MSDIMDQIKEQIESNDVLLYMKGNPNQPQCGFSARAVQALMECGKRFAYVD-----VLSNPDIRSNLPIYANWPTFPQLWVKG-----ELVGGCDIITEMGAN--GELKQIIDQAVPDEA---------------------- |
| A3QE03 | UniRef cluster | -----------------------------------MDTNETVEKIKQQIAENPIIVYMKGSPKLPSCGFSSQVAQIMINCGAQFAYVD-----ILQHPDIRAELPKYANWPTFPQLWVEG-----ELIGGCDILTEMYQK--GELQTLIADTAAKYADQEGEA---------------- |
| Q7N3V9 | UniRef cluster | -------------------------------------MTTTIEKIERQIKENPILLYMKGSPKLPNCGFSAQAVQALSACGERFAYVD-----ILQNPDIRAELPKYANWPTFPQLWVEG-----ELIGGCDIIIEMYQR--GELQTLIKETADKCRSQGENATE-------------- |
| Q31I31 | UniRef cluster | ---------------------------------MDNQEKETLDRIHEQVTNNPVVIYMKGTPQMPSCGFSSRTAQAMVETGEK-FAFVN----VLADPLIFEYLPKYQDWPTFPQVYIGG-----ELQGGCDITLELAES--GELKTLMAKANEAVSEEA------------------- |
| Q2NT10 | UniRef cluster | -------------------------------------MTTTIEKIQQQIAENPILLYMKGSPKLPGCGFSAQAVQALSACGERFAYVD-----VLTNPDIRAELPKFSNWPTFPQLWVDG-----ELIGGCDIIIEMYQR--GELQPLIKETAVKYNSQTDSAQ--------------- |
| Q083G7 | UniRef cluster | --------------------------------------METVEKIKQQISENPIIVYMKGSPKLPSCGFSSQVAQVMINCGEQFAFVD-----ILQHPDIRAELPKFANWPTFPQLWVEG-----ELIGGCDIITEMFQK--GELQPIIKATADKFRTEDAAE---------------- |
| Q6FCV2 | UniRef cluster | -------------------------MRLVEDLSMTEQARDTEALIRDQIAKHAVLLYMKGTPQFPQCGFSARAVEALSQIGRPFAYVN-----ILENPDIRATLPAIANWPTFPQLWVNG-----ELIGGSDIMLEMFQN--GELKPLVEQYSAAPDA--------------------- |
| Q1QVM8 | UniRef cluster | -------------------------------------MSTTLENIQQQIGENTILLYMKGTPQLPQCGFSAQAVQAVMACGERFAFVN-----ILDNPDIRAELPKYANWPTFPQLWVNG-----ELVGGCDIIVEMYES--GELEKLIKDAAASAETEKE------------------ |
| A0YH26 | UniRef cluster | --------------------------------------MDIMDTIKSQLESNPIILYMKGSPNQPQCGFSARTVQSVMSCGERFAFVD-----ILSNPDVRSNLPIYANWPTFPQLWVGG-----ELIGGCDIVCEMFEK--GELQTLLTETAALSADSKEDVSE-------------- |
| Q5X0Q8 | UniRef cluster | ----------------------------MGFLFYKRCIVDTLEKIKKQIAENAIMLYMKGTPKMPQCGFSARAVQCIEACGVDFAYVD-----ILANPDIRQVLPQFSDWPTFPQLYVKG-----ELIGGSDIIAEMFQQ--GELEPMLRDAVAA------------------------ |
| Q5WSI2 | UniRef cluster | ----------------------------MGFLFYKRCIVDTLEKIKKQIAENAIMLYMKGTPKMPQCGFSARAVQCIEACGVDFAYVD-----ILANPDIRQVLPQFSDWPTFPQLYVKG-----ELIGGSDIIAEMFQQ--GELEPMLRDAVAA------------------------ |
| Q8RNR7 | UniRef cluster | ----------------------------MGFLFYKRCIVDTLEKIKKQIAENAIMLYMKGTPKMPQCGFSARAVQCIEACGVDFAYVD-----ILANPDIRQVLPQFSDWPTFPQLYVKG-----ELIGGSDIIAEMFQQ--GELEPMLRDAVAA------------------------ |
| A8PPX1 | UniRef cluster | ---------------------------------MSMTQLTTFEIIKQQIERYPLILYMKGTPEKPCCGFSARVVDILKACRVKFAHVN-----ILEQSDIRRDLPQYSNWPTFPQLYYKG-----ELLGGCDIVEQLYIT--GQLEKIIGKLDFN------------------------ |
| A5IIG8 | UniRef cluster | --------------------------------------MDTLEKIKKQIAENAIMLYMKGTPKMPQCGFSARAVQCIEACGVDFAYVD-----ILANPDIRQVLPQFSDWPTFPQLYVKG-----ELIGGSDIIAEMFQQ--GELEPMLRDAVAA------------------------ |
| GLRX4 | UniRef cluster | -------------------------------------MSTTIEKIQRQIAENPILLYMKGSPKLPSCGFSAQAVQALAACGERFAYVD-----ILQNPDIRAELPKYANWPTFPQLWVDG-----ELVGGCDIVIEMYQR--GELQQLIKETAAKYKSEEPDAE--------------- |
| GLRX4 | UniRef cluster | -------------------------------------MSTTIEKIQRQIAENPILLYMKGSPKLPSCGFSAQAVQALAACGERFAYVD-----ILQNPDIRAELPKYANWPTFPQLWVDG-----ELVGGCDIVIEMYQR--GELQQLIKETAAKYKSEEPDAE--------------- |
| GLRX4 | UniRef cluster | -------------------------------------MSTTIEKIQRQIAENPILLYMKGSPKLPSCGFSAQAVQALAACGERFAYVD-----ILQNPDIRAELPKYANWPTFPQLWVDG-----ELVGGCDIVIEMYQR--GELQQLIKETAAKYKSEEPDAE--------------- |
| GLRX4 | UniRef cluster | -------------------------------------MSTTIEKIQRQIAENPILLYMKGSPKLPSCGFSAQAVQALAACGERFAYVD-----ILQNPDIRAELPKYANWPTFPQLWVDG-----ELVGGCDIVIEMYQR--GELQQLIKETAAKYKSEEPDAE--------------- |
| Q8XGR7 | UniRef cluster | -------------------------------------MSTTIEKIQRQIAENPILLYMKGSPKLPSCGFSAQAVQALSACGERFAYVD-----ILQNPDIRAELPKYANWPTFPQLWVDG-----ELVGGCDIVIEMYQR--GELQQLIKETAAKYKTQEPDAE--------------- |
| Q7CQK9 | UniRef cluster | -------------------------------------MSTTIEKIQRQIAENPILLYMKGSPKLPSCGFSAQAVQALSACGERFAYVD-----ILQNPDIRAELPKYANWPTFPQLWVDG-----ELVGGCDIVIEMYQR--GELQQLIKETAAKYKTQEPDAE--------------- |
| Q5PH12 | UniRef cluster | -------------------------------------MSTTIEKIQRQIAENPILLYMKGSPKLPSCGFSAQAVQALSACGERFAYVD-----ILQNPDIRAELPKYANWPTFPQLWVDG-----ELVGGCDIVIEMYQR--GELQQLIKETAAKYKTQEPDAE--------------- |
| Q57PK3 | UniRef cluster | -------------------------------------MSTTIEKIQRQIAENPILLYMKGSPKLPSCGFSAQAVQALSACGERFAYVD-----ILQNPDIRAELPKYANWPTFPQLWVDG-----ELVGGCDIVIEMYQR--GELQQLIKETAAKYKTQEPDAE--------------- |
| Q32FB7 | UniRef cluster | -------------------------------------MSTTIEKIQRQIAENPILLYMKGSPKLPSCGFSAQAVQALAACGERFAYVD-----ILQNPDIRAELPKYANWPTFPQLWVDG-----ELVGGCDIVIEMYQR--GELQQLIKETAAKYKSEEPDAE--------------- |
| Q321B1 | UniRef cluster | -------------------------------------MSTTIEKIQRQIAENPILLYMKGSPKLPSCGFSAQAVQALAACGERFAYVD-----ILQNPDIRAELPKYANWPTFPQLWVDG-----ELVGGCDIVIEMYQR--GELQQLIKETAAKYKSEEPDAE--------------- |
| A1ABJ6 | UniRef cluster | -------------------------------------MSTTIEKIQRQIAENPILLYMKGSPKLPSCGFSAQAVQALAACGERFAYVD-----ILQNPDIRAELPKYANWPTFPQLWVDG-----ELVGGCDIVIEMYQR--GELQQLIKETAAKYKSEEPDAE--------------- |
| Q1RBE1 | UniRef cluster | -------------------------------------MSTTIEKIQRQIAENPILLYMKGSPKLPSCGFSAQAVQALAACGERFAYVD-----ILQNPDIRAELPKYANWPTFPQLWVDG-----ELVGGCDIVIEMYQR--GELQQLIKETAAKYKSEEPDAE--------------- |
| Q0THH5 | UniRef cluster | -------------------------------------MSTTIEKIQRQIAENPILLYMKGSPKLPSCGFSAQAVQALAACGERFAYVD-----ILQNPDIRAELPKYANWPTFPQLWVDG-----ELVGGCDIVIEMYQR--GELQQLIKETAAKYKSEEPDAE--------------- |
| Q0T4B8 | UniRef cluster | -------------------------------------MSTTIEKIQRQIAENPILLYMKGSPKLPSCGFSAQAVQALAACGERFAYVD-----ILQNPDIRAELPKYANWPTFPQLWVDG-----ELVGGCDIVIEMYQR--GELQQLIKETAAKYKSEEPDAE--------------- |
| A9N0X7 | UniRef cluster | -------------------------------------MSTTIEKIQRQIAENPILLYMKGSPKLPSCGFSAQAVQALSACGERFAYVD-----ILQNPDIRAELPKYANWPTFPQLWVDG-----ELVGGCDIVIEMYQR--GELQQLIKETAAKYKTQEPDAE--------------- |
| A9MEI7 | UniRef cluster | -------------------------------------MSTTIEKIQRQIAENPILLYMKGSPKLPSCGFSAQAVQALSACGERFAYVD-----ILQNPDIRAELPKYANWPTFPQLWVDG-----ELVGGCDIVIEMYQR--GELQQLIKETAAKYKTQEPDAG--------------- |
| A9DKN9 | UniRef cluster | -----------------------------------METNETVEKIKAQLAENPIIVYMKGSPKLPSCGFSSQVAEIMINCNAKFAFVD-----ILQNPDIRSELPKYANWPTFPQLWIEG-----ELIGGCDILTEMYQK--GELQTLISETEAKHASEDEKNA--------------- |
| A8GDV9 | UniRef cluster | -------------------------------------MTTTIEKIQHQITENPILLYMKGSPKLPNCGFSAQAVQALSACGERFAYVD-----ILQNPDIRSELPKYANWPTFPQLWVDG-----ELVGGCDIIMEMYQR--GELQQLIKETAEKYKQQEDQQP--------------- |
| A8A0J9 | UniRef cluster | -------------------------------------MSTTIEKIQRQIAENPILLYMKGSPKLPSCGFSAQAVQALAACGERFAYVD-----ILQNPDIRAELPKYANWPTFPQLWVDG-----ELVGGCDIVIEMYQR--GELQQLIKETAAKYKSEEPDAE--------------- |
| A7ZMB8 | UniRef cluster | -------------------------------------MSTTIEKIQRQIAENPILLYMKGSPKLPSCGFSAQAVQALAACGERFAYVD-----ILQNPDIRAELPKYANWPTFPQLWVDG-----ELVGGCDIVIEMYQR--GELQQLIKETAAKYKSEEPDAE--------------- |
| Q12NQ2 | UniRef cluster | --------------------------------------METVEKIKQQISENPIIVYMKGSPKLPSCGFSSQVAQVMINCGEQFAFVD-----ILQHPDIRAELPKYANWPTFPQLWIEG-----ELIGGCDIITDMFQK--GELQPMIKATADKFRTEAE------------------ |
| A8FW30 | UniRef cluster | -----------------------------------METNETVAKIKQQIAENPIIVYMKGSPKLPSCGFSSQVAQIMINCNAQFAFVD-----ILQNPDIRSELPKYANWPTFPQLWVEG-----ELIGGCDILTEMYQK--GELQTLITETAAKYPSEEQA----------------- |
| A8AH34 | UniRef cluster | -------------------------------------MSTTIEKIQRQIAENPILLYMKGSPKLPSCGFSAQAVQALSACGERFAYVD-----ILQNPDIRAELPKYANWPTFPQLWVDG-----ELVGGCDILIEMYQR--GELQQLINETAAKYKTEEPDAE--------------- |
| A4VNU2 | UniRef cluster | --------------------------------------MDIIETIKEQIANNPVLLYMKGSPNAPQCGFSARATQAVMGCGEKFAYVD-----ILQNPEIRANLPIYANWPTFPQLWVNG-----ELVGGSDIILEMFEK--GELQTLIKSAVGKTEA--------------------- |
| Q9PA51 | UniRef cluster | --------------------------------------MLVMERIQAEIEEHPLVLFMKGTLEFPMCGYSSRATQALLAAGARHLHIVN----VLAEAEIRANLPRFSNWPTFPQLFIHG-----ELIGGCEIILELFES--GDLKRIVSEAD-------------------------- |
| Q3RFY2 | UniRef cluster | --------------------------------------MLVMERIQAEIEEHPLVLFMKGTLEFPMCGYSSRATQALLAAGARHLHIVN----VLAEAEIRANLPRFSNWPTFPQLFIHG-----ELIGGCEIILELFES--GDLKRILSEAD-------------------------- |
| Q3R551 | UniRef cluster | --------------------------------------MLVMERIQAEIEEHPLVLFMKGTLEFPMCGYSSRATQALLAAGARHLHIVN----VLAEAEIRANLPRFSNWPTFPQLFIHG-----ELIGGCEIILELFES--GDLKRILSEAD-------------------------- |
| Q15W17 | UniRef cluster | --------------------------------------METVEKIQQQISENPILLYMKGSPKLPNCGFSAQASQALMSCGEEFAYVD-----ILQNPDIRSELPKYADWPTFPQLWIDG-----ELIGGCDIIMEMVQQ--GELQTIVKESAEKRADK-------------------- |
| B0U604 | UniRef cluster | --------------------------------------MLVMERIQAEIEEHPLVLFMKGTLEFPMCGYSSRATQALLAAGARHLHIVN----VLAEAEIRANLPRFSNWPTFPQLFIHG-----ELIGGCEIILELFES--GDLKRILSEAD-------------------------- |
| A7MFC7 | UniRef cluster | -------------------------------------MSTTLEKIQRQIAENPILLYMKGSPKLPSCGFSAQAVQALSACGERFAYVD-----ILQNPDIRAELPKYANWPTFPQLWVDG-----ELVGGCDIVIEMYQR--GELQTLIKETAAKYNSATPDAE--------------- |
| A4A9N0 | UniRef cluster | --------------------------------------MDIMDQIKEQVEQNQVLLYMKGSPNQPQCGFSARVVQALMACGQRFAYVD-----ILSNPEIRANLPTYANWPTFPQLWVKG-----ELIGGCDIVTEMHEK--GELEPLVREASEAA----------------------- |
| A1S6W8 | UniRef cluster | --------------------------------------METVERIKQQIAENPIIVYMKGSPKLPSCGFSARVAEMMINIGEQFAYVD-----ILQHPDIRAELPKYANWPTFPQLWVEG-----ELIGGCDILTEMFQK--GELQPLIKEVAAKYKTEE------------------- |
| Q9HY77 | UniRef cluster | --------------------------------------MDIIETIKEQIANNPILLYMKGSPNAPQCGFSSRAAQVLMACGEKFAYVD-----ILQNPEIRANLPKYANWPTFPQLWVNG-----ELVGGSDILAEMFEK--GELQTLVKDAAAKANA--------------------- |
| Q6LP20 | UniRef cluster | --------------------------------------METIDKIKQQISENPILLYMKGSPKLPSCGFSSQTSQALMSCGEKFAYVD-----ILQNPDIRAELPVYAQWPTFPQLWIEG-----ELIGGCDIVLEMFQK--GELQPLIKEAAVRRDGEAAAE---------------- |
| Q1Z7T7 | UniRef cluster | --------------------------------------METIDKIKQQISENPILLYMKGSPKLPSCGFSSQTSQALMSCGEKFAYVD-----ILQNPDIRAELPVYAQWPTFPQLWIEG-----ELIGGCDIVLEMFQK--GELQPLIKEAAVRRDGEAAAE---------------- |
| Q02R04 | UniRef cluster | --------------------------------------MDIIETIKEQIANNPILLYMKGSPNAPQCGFSSRAAQVLMACGEKFAYVD-----ILQNPEIRANLPKYANWPTFPQLWVNG-----ELVGGSDILAEMFEK--GELQTLVKDAAAKANA--------------------- |
| A9KY85 | UniRef cluster | --------------------------------------METVEKIKQQITENPIIVYMKGSPKLPSCGFSSQVAQIMINCGEQFAFVD-----ILQHPDIRAELPKFANWPTFPQLWVEG-----ELIGGCDIVVDMFQK--GELQPLIKAAAAKIKTEE------------------- |
| A6V1R3 | UniRef cluster | --------------------------------------MDIIETIKEQIANNPILLYMKGSPNAPQCGFSSRAAQVLMACGEKFAYVD-----ILQNPEIRANLPKYANWPTFPQLWVNG-----ELVGGSDILAEMFEK--GELQTLVKDAAAKANA--------------------- |
| A3LDV3 | UniRef cluster | --------------------------------------MDIIETIKEQIANNPILLYMKGSPNAPQCGFSSRAAQVLMACGEKFAYVD-----ILQNPEIRANLPKYANWPTFPQLWVNG-----ELVGGSDILAEMFEK--GELQTLVKDAAAKANA--------------------- |
| A3KXG8 | UniRef cluster | --------------------------------------MDIIETIKEQIANNPILLYMKGSPNAPQCGFSSRAAQVLMACGEKFAYVD-----ILQNPEIRANLPKYANWPTFPQLWVNG-----ELVGGSDILAEMFEK--GELQTLVKDAAAKANA--------------------- |
| Q8D879 | UniRef cluster | --------------------------------------METIDKIKQQIAENPILLYMKGSPKLPSCGFSSQAAQALMACGEKFAYVD-----ILQNPDIRAELPKYAEWPTFPQLWVEG-----ELIGGCDIVIEMFQK--GELQPLIKEAAARAAGSDAE----------------- |
| Q7MM94 | UniRef cluster | -------------------------MRKICSHSVNEEAMETIDKIKQQIAENPILLYMKGSPKLPSCGFSSQAAQALMACGEKFAYVD-----ILQNPDIRAELPKYAEWPTFPQLWVEG-----ELIGGCDIVIEMFQK--GELQPLIKEAAARAAGSDAE----------------- |
| Q5NIK0 | UniRef cluster | --------------------------------MYTPEEQKVVERIEKQLKENDIILYMKGSPNLPQCGFSAHAATAIRSCGKPFAFVN-----ILENPDIRAILPKYADWPTFPQLWVKG-----ELIGGCDIIMEMNES--GELKKLIDSVK-------------------------- |
| Q3Z209 | UniRef cluster | -------------------------------------MSTTIEKIQRQIAENPILLYMKGSPKLPSCGFSAQAVQALAACGERFAYVD-----ILQNPDIRAELPKYANWPTFPQLWVDG-----KLVGGCDIVIEMYQR--GELQQLIKETAAKYKSEEPDAE--------------- |
| Q2A1I5 | UniRef cluster | --------------------------------MYTPEEQKVVERIEKQLKENDIILYMKGSPNLPQCGFSAHAATAIRSCGKPFAFVN-----ILENPDIRAILPKYADWPTFPQLWVKG-----ELIGGCDIIMEMNES--GELKKLIDSVK-------------------------- |
| Q14K03 | UniRef cluster | --------------------------------MYTPEEQKVVERIEKQLKENDIILYMKGSPNLPQCGFSAHAATAIRSCGKPFAFVN-----ILENPDIRAILPKYADWPTFPQLWVKG-----ELIGGCDIIMEMNES--GELKKLIDSVK-------------------------- |
| Q0BK87 | UniRef cluster | --------------------------------MYTPEEQKVVERIEKQLKENDIILYMKGSPNLPQCGFSAHAATAIRSCGKPFAFVN-----ILENPDIRAILPKYADWPTFPQLWVKG-----ELIGGCDIIMEMNES--GELKKLIDSVK-------------------------- |
| A8H3Z9 | UniRef cluster | --------------------------------------METVEKIKQQIAENPIIVYMKGSPKLPSCGFSSQVAQIMINCEAQFAFVD-----ILQHPDIRAELPKYANWPTFPQLWVEG-----ELIGGCDILTEMYQK--GELQTLIKETAAKYKTEDDQG---------------- |
| A7YVC9 | UniRef cluster | --------------------------------MYTPEEQKVVERIEKQLKENDIILYMKGSPNLPQCGFSAHAATAIRSCGKPFAFVN-----ILENPDIRAILPKYADWPTFPQLWVKG-----ELIGGCDIIMEMNES--GELKKLIDSVK-------------------------- |
| A7NEH1 | UniRef cluster | --------------------------------MYTPEEQKVVERIEKQLKENDIILYMKGSPNLPQCGFSAHAATAIRSCGKPFAFVN-----ILENPDIRAILPKYADWPTFPQLWVKG-----ELIGGCDIIMEMNES--GELKKLIDSVK-------------------------- |
| A7JNU5 | UniRef cluster | --------------------------------MYTPEEQKVVERIEKQLKENDIILYMKGSPNLPQCGFSAHAATAIRSCGKPFAFVN-----ILENPDIRAILPKYADWPTFPQLWVKG-----ELIGGCDIIMEMNES--GELKKLIDSVK-------------------------- |
| A7JJN7 | UniRef cluster | --------------------------------MYTPEEQKVVERIEKQLKENDIILYMKGSPNLPQCGFSAHAATAIRSCGKPFAFVN-----ILENPDIRAILPKYADWPTFPQLWVKG-----ELIGGCDIIMEMNES--GELKKLIDSVK-------------------------- |
| A7JAU0 | UniRef cluster | --------------------------------MYTPEEQKVVERIEKQLKENDIILYMKGSPNLPQCGFSAHAATAIRSCGKPFAFVN-----ILENPDIRAILPKYADWPTFPQLWVKG-----ELIGGCDIIMEMNES--GELKKLIDSVK-------------------------- |
| A6WM80 | UniRef cluster | --------------------------------------METVEKIKQQITENPIIVYMKGSPKLPSCGFSSQVAQIMINCGEQFAFVD-----ILQHPDIRAELPKFANWPTFPQLWIEG-----ELIGGCDIVVDMFQK--GELQPLIKAAAAKIKTEE------------------- |
| A4KT54 | UniRef cluster | --------------------------------MYTPEEQKVVERIEKQLKENDIILYMKGSPNLPQCGFSAHAATAIRSCGKPFAFVN-----ILENPDIRAILPKYADWPTFPQLWVKG-----ELIGGCDIIMEMNES--GELKKLIDSVK-------------------------- |
| A4IW27 | UniRef cluster | --------------------------------MYTPEEQKVVERIEKQLKENDIILYMKGSPNLPQCGFSAHAATAIRSCGKPFAFVN-----ILENPDIRAILPKYADWPTFPQLWVKG-----ELIGGCDIIMEMNES--GELKKLIDSVK-------------------------- |
| A3D3H7 | UniRef cluster | --------------------------------------METVEKIKQQITENPIIVYMKGSPKLPSCGFSSQVAQIMINCGEQFAFVD-----ILQHPDIRAELPKFANWPTFPQLWIEG-----ELIGGCDIVVDMFQK--GELQPLIKAAAAKIKTEE------------------- |
| A0Q8D6 | UniRef cluster | --------------------------------MYTPEEQKVVERIEKQLKENDIILYMKGSPNLPQCGFSAHAATAIRSCGKPFAFVN-----ILENPDIRAILPKYADWPTFPQLWVKG-----ELIGGCDIIMEMNES--GELKKLIDSVK-------------------------- |
| GLRXA | UniRef cluster | ------------------------------------------------------MLYMKGTPKMPQCGFSARAVQCIEACGVDFAYVD-----ILANPDIRQVLPQFSDWPTFPQLYVKG-----ELIGGSDIIAEMFQQ--GELEPMLRDAVAA------------------------ |
| Q21HY1 | UniRef cluster | --------------------------------------MDIIETIKKQIEENSIILYMKGSPNAPQCGFSAKASQAVMACGQRFAYVD-----ILSNPDIRANLPKYANWPTFPQLWVNG-----ELVGGCDIIVQMHES--GELKPIIDAAAPKEEASE------------------- |
| Q057V2 | UniRef cluster | -------------------------------------MIKTIKKIEKQLQNNNIVLYMKGSPEHPHCGFSAQAVQALSSCTSNFFYVD-----VLKNPDIRSVLPQYSKWPTFPQLWINK-----KLIGGCDIILEKFYN--KELLDLIKKK--------------------------- |
| Q0I4B2 | UniRef cluster | --------------------------------------METLDKIKKQISENPILIYMKGSPKFPSCGFSARAVEVLINCNVPFGYVD-----ILQHPDIRAALPNYANWPTFPQLWVEG-----ELIGGCDIMLEMFQQ--GELQSLLSEVAARYPQ--------------------- |
| B0UUN9 | UniRef cluster | --------------------------------------METLDKIKKQISENPILIYMKGSPKFPSCGFSARAVEVLINCNVPFGYVD-----ILQHPDIRAALPNYANWPTFPQLWVEG-----ELIGGCDIMLEMFQQ--GELQPLLSEVAARYPQ--------------------- |
| B0TWT0 | UniRef cluster | --------------------------------MYTQEEQKVVDRIEKQLKENDIILYMKGSPNLPQCGFSAHAASAIRACGKPFAFVN-----ILENPDIRAILPKYADWPTFPQLWVKG-----ELIGGCDIIMEMNES--GELKELIDSVK-------------------------- |
| B0TVK9 | UniRef cluster | --------------------------------------METVEKIKQQIAENPIILYMKGSPKLPSCGFSSQVAQIMINCDEQFAFVD-----ILQHPDIRAELPKYANWPTFPQLWVEG-----ELIGGCDILTEMFQK--GELQTLIKETGAKYKTEDDQA---------------- |
| A6TA02 | UniRef cluster | -------------------------------------MSSTLEKIQRQIAENPILLYMKGSPKLPSCGFSAQAVQALSACGERFAYVD-----ILQNPDIRAELPKYANWPTFPQLWVDG-----ELVGGCDIVIEMYQR--GELQQLIKETAAKYHTDEPKAE--------------- |
| A4W9U2 | UniRef cluster | -------------------------------------MTTTIEKIQRQVAENPILLYMKGSPKLPSCGFSAQAVQALSACGERFAYVD-----ILQNPDIRAEMPKYANWPTFPQLWVDG-----ELVGGCDILIEMYQR--GELQQLIKETAAKYKTEEPDAE--------------- |
| Q5QWM7 | UniRef cluster | --------------------------------------METVDRIKQQIEENPILLYMKGSPKLPSCGFSSQASQALMGCGQAFAYVD-----ILQNPDIRAELPKYANWPTFPQLWVEG-----ELVGGCDIIMEMFQN--GELQELVSEVAERHPEPSAE----------------- |
| Q0HW62 | UniRef cluster | --------------------------------------METVEKIKQQIAENPIIVYMKGSPKLPSCGFSSQVAQIMINCGEQFAFVD-----ILQHPDIRAELPKYANWPTFPQLWIEG-----ELIGGCDIVVDMYQK--GELQPLIKATADKYKTAE------------------- |
| Q0HJW7 | UniRef cluster | --------------------------------------METVEKIKQQIAENPIIVYMKGSPKLPSCGFSSQVAQIMINCGEQFAFVD-----ILQHPDIRAELPKYANWPTFPQLWIEG-----ELIGGCDIVVDMYQK--GELQPLIKATADKYKTAE------------------- |
| B0QRF6 | UniRef cluster | --------------------------------------METIEKIQKQISENPILLYMKGSPKFPSCGFSARASEAIVNCQVPFGYVD-----VLSNPDIRAELPKYANWPTFPQLWVDG-----ELIGGCDIVLEMFQK--GELQTLLKDTAKKYSLYSV------------------ |
| A7JQ36 | UniRef cluster | --------------------------------------METIDKIKQQISENPILLYMKGSPKFPSCGFSARAVEAVINCQVPFGYVD-----ILTNPDIRAELPKFANWPTFPQLWVEG-----ELVGGCDIVLEMLQK--GELQTLLKETAAKHAA--------------------- |
| A4Y630 | UniRef cluster | --------------------------------------METVEKIKQQIAENPIIVYMKGSPKLPSCGFSSQVAQIMINCGEQFAFVD-----ILQHPDIRAELPKYANWPTFPQLWIEG-----ELIGGCDIVVDMFQK--GELQPLIKATAEKYKTAE------------------- |
| A2V5E8 | UniRef cluster | --------------------------------------METVEKIKQQIAENPIIVYMKGSPKLPSCGFSSQVAQIMINCGEQFAFVD-----ILQHPDIRAELPKYANWPTFPQLWIEG-----ELIGGCDIVVDMFQK--GELQPLIKATAEKYKTAE------------------- |
| A1RKG6 | UniRef cluster | --------------------------------------METVEKIKQQIAENPIIVYMKGSPKLPSCGFSSQVAQIMINCGEQFAFVD-----ILQHPDIRAELPKYANWPTFPQLWIEG-----ELIGGCDIVVDMFQK--GELQPLIKATAEKYKTAE------------------- |
| A0KVQ4 | UniRef cluster | --------------------------------------METVEKIKQQIAENPIIVYMKGSPKLPSCGFSSQVAQIMINCGEQFAFVD-----ILQHPDIRAELPKYANWPTFPQLWIEG-----ELIGGCDIVVDMYQK--GELQPLIKATADKYKTAE------------------- |
| GLRX4 | UniRef cluster | --------------------------------------METIDKIKQQINENPILLYMKGSPKFPSCGFSARAVEAIIQCQVPFGYVD-----ILTNPDIRSELPKFANWPTFPQLWVEG-----ELIGGCDIILEMFQK--GELHTLLKETATKHG---------------------- |
| Q66A35 | UniRef cluster | --------------------------------------MTTIDKIQRQITENPILLYMKGSPKLPNCGFSAQAVQALSACGERFAYVD-----ILQNPDIRAELPKYANWPTFPQLWVDG-----ELVGGCDILMEMYQR--GELQTLLKETADKYRSQEDQPAAE------------- |
| Q8D0M0 | UniRef cluster | ---------------------------------MRKQEMTTIDKIQRQITENPILLYMKGSPKLPNCGFSAQAVQALSACGERFAYVD-----ILQNPDIRAELPKYANWPTFPQLWVDG-----ELVGGCDILMEMYQR--GELQTLLKETADKYRSQEDQPAAE------------- |
| Q2C5R7 | UniRef cluster | --------------------------------------METIDKIKQQIAENPILLYMKGSPKLPSCGFSSQASQALMGCGEKFAYVD-----ILQNPDIRAELPIYAQWPTFPQLWVDG-----ELIGGCDIILEMFQK--GELQPLLKEVAERRDAASAE----------------- |
| Q1LU00 | UniRef cluster | -------------------------------------MINTVSQLQQQIAEYPILLYMKGSPKLPSCGFSAQAVQILSNCSKSFAYIN-----ILTNPYIRTELPKLANWPTYPQLWVDG-----NFIGGCDIVMEMYQH--GELQILIQKTINKYPSHTYNEPIEIS----------- |
| Q1CIL3 | UniRef cluster | --------------------------------------MTTIDKIQRQITENPILLYMKGSPKLPNCGFSAQAVQALSACGERFAYVD-----ILQNPDIRAELPKYANWPTFPQLWVDG-----ELVGGCDILMEMYQR--GELQTLLKETADKYRSQEDQPAAE------------- |
| Q1C777 | UniRef cluster | --------------------------------------MTTIDKIQRQITENPILLYMKGSPKLPNCGFSAQAVQALSACGERFAYVD-----ILQNPDIRAELPKYANWPTFPQLWVDG-----ELVGGCDILMEMYQR--GELQTLLKETADKYRSQEDQPAAE------------- |
| Q0WED9 | UniRef cluster | --------------------------------------MTTIDKIQRQITENPILLYMKGSPKLPNCGFSAQAVQALSACGERFAYVD-----ILQNPDIRAELPKYANWPTFPQLWVDG-----ELVGGCDILMEMYQR--GELQTLLKETADKYRSQEDQPAAE------------- |
| B0HWI2 | UniRef cluster | --------------------------------------MTTIDKIQRQITENPILLYMKGSPKLPNCGFSAQAVQALSACGERFAYVD-----ILQNPDIRAELPKYANWPTFPQLWVDG-----ELVGGCDILMEMYQR--GELQTLLKETADKYRSQEDQPAAE------------- |
| B0HDS5 | UniRef cluster | --------------------------------------MTTIDKIQRQITENPILLYMKGSPKLPNCGFSAQAVQALSACGERFAYVD-----ILQNPDIRAELPKYANWPTFPQLWVDG-----ELVGGCDILMEMYQR--GELQTLLKETADKYRSQEDQPAAE------------- |
| B0H5N4 | UniRef cluster | --------------------------------------MTTIDKIQRQITENPILLYMKGSPKLPNCGFSAQAVQALSACGERFAYVD-----ILQNPDIRAELPKYANWPTFPQLWVDG-----ELVGGCDILMEMYQR--GELQTLLKETADKYRSQEDQPAAE------------- |
| B0GXE3 | UniRef cluster | --------------------------------------MTTIDKIQRQITENPILLYMKGSPKLPNCGFSAQAVQALSACGERFAYVD-----ILQNPDIRAELPKYANWPTFPQLWVDG-----ELVGGCDILMEMYQR--GELQTLLKETADKYRSQEDQPAAE------------- |
| B0GHL1 | UniRef cluster | --------------------------------------MTTIDKIQRQITENPILLYMKGSPKLPNCGFSAQAVQALSACGERFAYVD-----ILQNPDIRAELPKYANWPTFPQLWVDG-----ELVGGCDILMEMYQR--GELQTLLKETADKYRSQEDQPAAE------------- |
| A9ZZX6 | UniRef cluster | --------------------------------------MTTIDKIQRQITENPILLYMKGSPKLPNCGFSAQAVQALSACGERFAYVD-----ILQNPDIRAELPKYANWPTFPQLWVDG-----ELVGGCDILMEMYQR--GELQTLLKETADKYRSQEDQPAAE------------- |
| A9ZAG6 | UniRef cluster | --------------------------------------MTTIDKIQRQITENPILLYMKGSPKLPNCGFSAQAVQALSACGERFAYVD-----ILQNPDIRAELPKYANWPTFPQLWVDG-----ELVGGCDILMEMYQR--GELQTLLKETADKYRSQEDQPAAE------------- |
| A9QZB0 | UniRef cluster | --------------------------------------MTTIDKIQRQITENPILLYMKGSPKLPNCGFSAQAVQALSACGERFAYVD-----ILQNPDIRAELPKYANWPTFPQLWVDG-----ELVGGCDILMEMYQR--GELQTLLKETADKYRSQEDQPAAE------------- |
| A7FHK5 | UniRef cluster | --------------------------------------MTTIDKIQRQITENPILLYMKGSPKLPNCGFSAQAVQALSACGERFAYVD-----ILQNPDIRAELPKYANWPTFPQLWVDG-----ELVGGCDILMEMYQR--GELQTLLKETADKYRSQEDQPAAE------------- |
| A6BQ01 | UniRef cluster | --------------------------------------MTTIDKIQRQITENPILLYMKGSPKLPNCGFSAQAVQALSACGERFAYVD-----ILQNPDIRAELPKYANWPTFPQLWVDG-----ELVGGCDILMEMYQR--GELQTLLKETADKYRSQEDQPAAE------------- |
| A4TIQ7 | UniRef cluster | --------------------------------------MTTIDKIQRQITENPILLYMKGSPKLPNCGFSAQAVQALSACGERFAYVD-----ILQNPDIRAELPKYANWPTFPQLWVDG-----ELVGGCDILMEMYQR--GELQTLLKETADKYRSQEDQPAAE------------- |
| A1JP40 | UniRef cluster | --------------------------------------MTTIDKIQRQIAENPILLYMKGSPKLPNCGFSAQAVQALSACGERFAYVD-----ILQNPDIRAELPKYANWPTFPQLWVDG-----ELVGGCDILMEMYQR--GELQQLLKETADKYRTEKPAAE--------------- |
| Q5E6C9 | UniRef cluster | --------------------------------------METIDKIKQQISENHILLYMKGSPKLPSCGFSSQAAQALMNCGEKFAYVD-----ILQNPDIRAELPAYAQWPTFPQLWVEG-----ELVGGCDIIIEMFQK--GELQPLIKEAAERNAPAAE------------------ |
| Q1ZRZ9 | UniRef cluster | --------------------------------------METIDKIKQQIAENPILLYMKGSPKLPSCGFSSQASQALMGCGEKFAYVD-----ILQNPDIRAELPVYAQWPTFPQLWIEG-----ELIGGCDIILEMFQK--GELQSLIKDAAARRDAASAE----------------- |
| A9I2B5 | UniRef cluster | --------------------------------------METIDKIKQQISENHILLYMKGSPKLPSCGFSSQAAQALMNCGEKFAYVD-----ILQNPDIRAELPAYAQWPTFPQLWVEG-----ELVGGCDIIIEMFQK--GELQPLIKEAAERNAPAAE------------------ |
| Q8ED84 | UniRef cluster | --------------------------------------METVEKIKQQIGENPIIVYMKGSPKLPSCGFSCQVAQIMINCGEQFAFVD-----ILQHPDIRAELPKYANWPTFPQLWIEG-----ELIGGCDIVVDMYQK--GELQPLIKATAEKYKTAE------------------- |
| Q094B7 | UniRef cluster | ---------------------------------------------------------MKGNALFPQCGFSARALQLLQPLG-QVHTVD-----VLADPAIRQGIKDYSNWPTIPQVYING-----EFVGGSDILAEMAER--GELANLVAGTSSGAAQ--------------------- |
| A4SKQ6 | UniRef cluster | --------------------------------------METIEKIKQQLADNPIILYMKGSPKLPSCGFSAQASQALMSCGEPFAYVD-----ILQNPDIRAELPKFANWPTFPQLWVEG-----ELVGGCDILIEMFQA--GELQTLIKETAAKHKQDDVAAE--------------- |
| B0BRN1 | UniRef cluster | -----------------------------------METIDTIEKIKKQIGENPILLYMKGSPKFPSCGFSARAVEAVINCQVPFGYVD-----ILTNPDIRAELPKFANWPTFPQLWVEG-----ELVGGCDIVLEMFQK--GELQTLLKEVAAKHA---------------------- |
| A5L1H2 | UniRef cluster | --------------------------------------METIDKIKQQIEENTILLYMKGSPKLPSCGFSSQASQALMACGEKFAYVD-----ILQNPDIRAELPAYAQWPTFPQLWVEG-----ELIGGCDIILEMFQK--GELQPIIKEAAAKVAGDDAE----------------- |
| A3Y4A7 | UniRef cluster | --------------------------------------METIDKIKQQIEENTILLYMKGSPKLPSCGFSSQASQALMACGEKFAYVD-----ILQNPDIRAELPAYAQWPTFPQLWVEG-----ELIGGCDIILEMFQK--GELQPIVKEAAAKVAGDDAE----------------- |
| A0KI42 | UniRef cluster | --------------------------------------METIEKIKQQLAENPIILYMKGSPKLPSCGFSAQASQALMSCGEPFAYVD-----ILQNPDIRAELPKFANWPTFPQLWVEG-----ELVGGCDIMIEMFQA--GELQTLIKETAAKHKQDDAAAE--------------- |
| Q87A05 | UniRef cluster | --------------------------------------MLVMERIQAEIEEHPLVLFMKGTLEFPMCGYSSRATQALLAAGARHLHIVN----VLAEAEIRANLPRFSNWLTFPQLFIHG-----ELIGGCEIILELFES--GDLKRILSEAD-------------------------- |
| Q47YH1 | UniRef cluster | --------------------------------------MDTIERIKEQISENTILLYMKGSPKLPNCGFSSQASQALISCEEKFAYVD-----ILQNPDIRAELPKYADWPTFPQLWVDG-----ELVGGCDIIMEMFQQ--GELQTLVKAAASKNASAEDSSADA------------- |
| A5UCU2 | UniRef cluster | --------------------------------------METLDKIKKQISENPILIYMKGSPKFPSCGFSARASEALMNCKVPFGYVD-----ILQHPDIRAELPTYANWPTFPQLWVDG-----ELIGGCDIILEMYQA--GELQTLLAEVAAKHV---------------------- |
| A4BI09 | UniRef cluster | -------------------------------------MSDTLENIKEQISGNDILLYMKGNPNQPMCGFSARAVQAVMSCGERFAYVD-----ILQHPDIRAELPKYANWPTFPQLWIKG-----ELIGGCDIITEMFES--GELQEAIKEAAPSA----------------------- |
| A4B3F5 | UniRef cluster | --------------------------------MDNTENQSTLDRIKQQIEENPILLYMKGSPKLPSCGFSSQASQALMSCGEPFAYVD-----ILQNPDIRAELPKYANWPTFPQLWVEG-----ELVGGCDIIIEMFQQ--NELQPLIKETAEKFKEEE------------------- |
| Q9KQF4 | UniRef cluster | --------------------------------------METIDKIKQQIAENPILLYMKGSPKLPSCGFSSQAAQALMACGEKFAYVD-----ILQNPDIRAELPVYAQWPTFPQLWIEG-----ELIGGCDIMLEMFQK--GELQTLVKEAAARSASQE------------------- |
| Q87XL7 | UniRef cluster | --------------------------------------MDIIETIKEQIASNTILLYMKGAPNAPQCGFSAKASQALMACGEKFAYVD-----ILQNPEIRANLPKYANWPTFPQLWVAG-----ELVGGSDIITEMMAD--GSLQTLVKEASAAKAE--------------------- |
| A7MUK1 | UniRef cluster | ---------------------------------MNEEAMETIDKIKQQISENTILLYMKGSPKLPSCGFSSQAAQALMACGEKFAYVD-----ILQNPDIRAELPKYAQWPTFPQLWVEG-----ELIGGCDIILEMFQK--GELQPLIKEAAARVEGDAE------------------ |
| A6XX40 | UniRef cluster | --------------------------------------METIDKIKQQIAENPILLYMKGSPKLPSCGFSSQAAQALMACGEKFAYVD-----ILQNPDIRAELPVYAQWPTFPQLWIEG-----ELIGGCDIMLEMFQK--GELQILVKEAAARSASQE------------------- |
| A5F6L2 | UniRef cluster | --------------------------------------METIDKIKQQIAENPILLYMKGSPKLPSCGFSSQAAQALMACGEKFAYVD-----ILQNPDIRAELPVYAQWPTFPQLWIEG-----ELIGGCDIMLEMFQK--GELQTLVKEAAARSASQE------------------- |
| A3GTQ5 | UniRef cluster | --------------------------------------METIDKIKQQIAENPILLYMKGSPKLPSCGFSSQAAQALMACGEKFAYVD-----ILQNPDIRAELPVYAQWPTFPQLWIEG-----ELIGGCDIMLEMFQK--GELQTLVKEAAARSASQE------------------- |
| A2PD62 | UniRef cluster | --------------------------------------METIDKIKQQIAENPILLYMKGSPKLPSCGFSSQAAQALMACGEKFAYVD-----ILQNPDIRAELPVYAQWPTFPQLWIEG-----ELIGGCDIMLEMFQK--GELQTLVKEAAARSASQE------------------- |
| A1FB11 | UniRef cluster | --------------------------------------METIDKIKQQIAENPILLYMKGSPKLPSCGFSSQAAQALMACGEKFAYVD-----ILQNPDIRAELPVYAQWPTFPQLWIEG-----ELIGGCDIMLEMFQK--GELQTLVKEAAARSASQE------------------- |
| GLRX4 | UniRef cluster | --------------------------------------METLDKIKKQISENPILIYMKGSPKFPSCGFSARAVEALMHCKVPFGYVD-----ILQHPDIRAELPAYANWPTFPQLWVDG-----ELVGGCDIILEMFQQ--GELQTLLADVAAKYPQE-------------------- |
| Q87MW4 | UniRef cluster | --------------------------------------METIDKIKQQISENSILLYMKGSPKLPSCGFSSQASQALMACGEKFAYVD-----ILQNPDIRAELPKYAQWPTFPQLWVEG-----ELIGGCDIILEMFQK--GELQPLIKEAAARAEGQAE------------------ |
| Q3IKP3 | UniRef cluster | --------------------------------------METIDKIKQQISENPIILFMKGSPKLPNCGFSSQASQALMSCGEPFAYVD-----ILLNPDIRAELPAYANWPTFPQLWVDG-----ELVGGCDIIIEMFQR--GELQPLITETAAKYKEADAE----------------- |
| A4XXM5 | UniRef cluster | --------------------------------------MDIIETIKEQIANNTVLLYMKGSPNAPQCGFSARAAQVVMGCGEKFAYVD-----ILQNPEIRANLPKYANWPTFPQLWVAG-----ELVGGSDILTEMYEK--GELQTLIKDAVSKAGA--------------------- |
| A0Y092 | UniRef cluster | --------------------------------------METIDKIKQQISENPIILFMKGSPKLPNCGFSSQASQALMSCGEPFAYVD-----ILLNPDIRAELPAYANWPTFPQLWVEG-----ELIGGCDIIIEMFQR--GELQPLITETAAKYKEADAE----------------- |
| Q48F08 | UniRef cluster | --------------------------------------MDIIETIKEQIANNTILLYMKGAPNAPQCGFSAKASQALMACGEKFAYVD-----ILQNPEIRANLPKYANWPTFPQLWVAG-----ELVGGSDIITEMMAD--GSLQTLVKEASAAKAE--------------------- |
| Q4ZPJ4 | UniRef cluster | --------------------------------------MDIIETIKEQIANNTILLYMKGAPNAPQCGFSAKASQALMACGEKFAYVD-----ILQNPEIRANLPKYANWPTFPQLWVAG-----ELVGGSDIITEMMDD--GSLQTLVKEASAAKAE--------------------- |
| A6FIU8 | UniRef cluster | --------------------------------------METLDKIKQQLAENSIILYMKGSPKLPSCGFSSQASQAVINCGEQFAYVD-----ILQNPDIRAELPKYANWPTFPQLWVDG-----ELVGGCDIIMEMFQQ--GELQPLIAAAAEKAKAE-------------------- |
| A8T8W0 | UniRef cluster | --------------------------------------METIDKIKQQISENTILLYMKGSPKLPSCGFSSQASQALMACGEKFAYVD-----ILQNPDIRAELPIYAQWPTFPQLWVEG-----ELIGGCDIILEMFQK--GELQPLIKEAAARVDGDAE------------------ |
| GLRX4 | UniRef cluster | --------------------------------------MNVIEKIERQIKDNIILIYMKGTPQSPSCGFSAQAVQALSICGEKFAYVD-----ILENLDIRKELPRYANWPTFPQLWIKG-----ELIGGCSIILEMLEN--GELKKIISNAVLNSK---------------------- |
| Q88NX2 | UniRef cluster | --------------------------------------MDIIETIKEQIANNTILLYMKGSPNAPQCGFSARASQAVMGCGEKFAYVD-----ILQNPEIRANLPKYANWPTFPQLWVAG-----ELVGGSDIMLEMFEK--GELQTLIKDAAAKAKASEA------------------ |
| Q4J689 | UniRef cluster | --------------------------------------MDIIETIKEQIASNGILLYMKGSPNAPQCGFSARAAQALMACGERFAYVD-----ILQNPEIRANLPKYANWPTFPQLWVKG-----ELIGGSDIILELFEK--GELQQIVRGAADKADA--------------------- |
| A4NAA0 | UniRef cluster | --------------------------------------METLDKIKKQISENSILIYMKGSPKFPSCGFSARASEALMNCKVPFGYVD-----ILQHPDIRAELPTYANWPTFPQLWVEG-----ELIGGCDIILEMYQA--GELQTLLAEVAAKHA---------------------- |
| A1SXG5 | UniRef cluster | --------------------------------------METLDKIKSQISENTVLLYMKGSPKLPSCGFSSQASQALMQCGHPFAYVD-----ILQNPDIRAELPKYANWPTFPQLWIAG-----ELVGGCDIIMEMSQQ--GELKPIVEQAVAATATAESE----------------- |
| GLRX4 | UniRef cluster | --------------------------------------METLDKIKKQISENPILIYMKGSPKLPSCGFSARASEALMHCKVPFGYVD-----ILQHPDIRAELPTYANWPTFPQLWVEG-----ELIGGCDIILEMYQA--GELQTLLAEVAAKHA---------------------- |
| Q1IE10 | UniRef cluster | --------------------------------------MDIIETIKEQIANNTILLYMKGSPNAPQCGFSAKAAQAVMGCGEKFAYVD-----ILQNPEIRANLPKYANWPTFPQLWVAG-----ELVGGSDIMSEMFAN--GELQTLIKEAAAKAKASEA------------------ |
| A5UIT4 | UniRef cluster | --------------------------------------METLDKIKKQISENPILIYMKGSPKLPSCGFSARASEALMHCKVPFGYVD-----ILQHPDIRAELPTYANWPTFPQLWVEG-----ELIGGCDIILEMYQA--GELQTLLAEVAAKHA---------------------- |
| A4NY98 | UniRef cluster | --------------------------------------METLDKIKKQISENPILIYMKGSPKLPSCGFSARASEALMHCKVPFGYVD-----ILQHPDIRAELPTYANWPTFPQLWVEG-----ELIGGCDIILEMYQA--GELQTLLAEVAAKHA---------------------- |
| A4NSJ1 | UniRef cluster | --------------------------------------METLDKIKKQISENPILIYMKGSPKLPSCGFSARASEALMHCKVPFGYVD-----ILQHPDIRAELPTYANWPTFPQLWVEG-----ELIGGCDIILEMYQA--GELQTLLAEVAAKHA---------------------- |
| A4NLC0 | UniRef cluster | --------------------------------------METLDKIKKQISENPILIYMKGSPKLPSCGFSARASEALMHCKVPFGYVD-----ILQHPDIRAELPTYANWPTFPQLWVEG-----ELIGGCDIILEMYQA--GELQTLLAEVAAKHA---------------------- |
| A4NFZ0 | UniRef cluster | --------------------------------------METLDKIKKQISENPILIYMKGSPKLPSCGFSARASEALMHCKVPFGYVD-----ILQHPDIRAELPTYANWPTFPQLWVEG-----ELIGGCDIILEMYQA--GELQTLLAEVVAKHA---------------------- |
| A4N4Z8 | UniRef cluster | --------------------------------------METLDKIKKQISENPILIYMKGSPKLPSCGFSARASEALMHCKVPFGYVD-----ILQHPDIRAELPTYANWPTFPQLWVEG-----ELIGGCDIILEMYQA--GELQTLLAEVAAKHA---------------------- |
| Q7VR56 | UniRef cluster | ------------------------------------MSNNTVEKIKKQIQANPILLYMKGTPDSAKCGFSAKSAQILHLYVKSFYYID-----VLIDTDIRSVLPTFSNWPTFPQLWLEG-----KLIGGCDIIMDMHQN--GTLKQAIDPIKLKYNLN-------------------- |
| Q4K742 | UniRef cluster | --------------------------------------MDIIETIKEQIANNTILLYMKGSPNAPQCGFSAKAAQAVMGCGEKFAYVD-----ILQNPEIRANLPKYANWPTFPQLWVAG-----ELVGGSDIMAEMFAN--GELQSLIKAAAEKAAAKTEA----------------- |
| Q492U1 | UniRef cluster | -------------------------------------MNNAIEKIKNQIKENPIVLYMKGTPSAPKCGFSSKAAQILSTYTQSFFYID-----VLIHVDVRNALPIFSNWPTFPQLWIEG-----KLIGGSDIILNMSHS--GTLKTLIDQVKLRHNLN-------------------- |
| Q3K7J3 | UniRef cluster | --------------------------------------MDIIETIKEQIANNTILLYMKGSPNAPQCGFSAKAAQAVMGCGEKFAYVD-----ILQNPEIRANLPKYANWPTFPQLWVAG-----ELVGGSDIMAEMFAN--GELQTLVKEASAKAAAAKSQA---------------- |
| A6VN68 | UniRef cluster | --------------------------------------METLDKIKKQIAENPILIYMKGSPKLPACGFSARAVEALMNCQVPFGYVD-----ILQHADIRAELPKFANWPTFPQLWVEG-----ELVGGCDIVLEMFQV--GELQTLLKEVAERHSA--------------------- |
| GLRX4 | UniRef cluster | --------------------------------------METLDKIKKQISENPILIYMKGSPKLPSCGFPARASEALMHCKVPFGYVD-----ILQHPDIRAELPTYANWPTFPQLWVEG-----ELIGGCDIILEMYQA--GELQTLLAEVAAKHA---------------------- |
| Q65T91 | UniRef cluster | --------------------------------------METLDKIKKQIAENPILIYMKGSPKLPACGFSARAVEALINCQVPFGYVD-----ILQHADVRAELPKYANWPTFPQLWVEG-----ELIGGCDILLEMYQA--GELQTLLKEVAERHKEQV------------------- |
| B0KUF4 | UniRef cluster | --------------------------------------MDIIDTIKEQIANNTILLYMKGSPNAPQCGFSAKASQAVMGCGEKFAYVD-----ILQNPEIRANLPKYANWPTFPQLWVAG-----ELVGGSDIMLEMFEK--GELQTLIKEAAAKAKAAEA------------------ |
| A4C5N6 | UniRef cluster | --------------------------------------METIDKIKQQISENSILLYMKGSPKLPNCGFSSQASQALMSCGEPFAYVD-----ILLNPDIRAELPHYANWPTFPQLWIEG-----ELIGGCDIIIEMFQR--GELQPLIAEAAARNKPAEEAAE--------------- |
| A3MYE5 | UniRef cluster | -----------------------------------METIDTIEKIKKQIGENPILLYMKGSPKFPSCGFSARAGEAVINCQVPFGYVD-----ILTNPDIRAEL--FANWPTFPQLWVEG-----ELVGGCDIVLEMFQK--GELQTLLKEVAAKHA---------------------- |
| A4MVH5 | UniRef cluster | ---------------------------------------------------------MKGSPKLPSCGFSARASEALMHCKVPFGYVD-----ILQHPDIRAELPTYANWPTFPQLWVEG-----ELIGGCDIILEMYQA--GELQTLLAEVAAKHA---------------------- |
| A9CFB6 | UniRef cluster | ---------------------------------------MTLSCVLPHVCEALNNMAPVTIYTRDFCGYCARAKALLDMK-GVDYAEYNATTTPEYRQEMIEKS---GGTTFPQIFINGQ------HVGGCDDLHALERA--GKLDAMLAG---------------------------- |
| A8MJH2 | UniRef cluster | -------------------------------------------------------MKNITIYTKNYCPYCKKAVSLLSSK-GVDFKEVDVTHDSKAFEDVMAKTG---WDTVPQVFVDEE------FLGGCDDIHALDRQ--GILDKKLGLK--------------------------- |
| Q16D29 | UniRef cluster | -------------------------------------------------------MKNVEIYTSPLCGFCHAAKRLLSQK-GIDFAEVDVLADPDRKPEMVQRAK--GSRTVPQIFVGDV------HVGGCDDLYALERA--GKLDQLLAA---------------------------- |
| Q5P9T8 | UniRef cluster | -------------------------------------------------------MREVLIYTKVPCPYCTRAKALFNKKNVPFKEIDITDN---PEAMREMVERS-GRRTVPQIFIDGK------SIGGCDDLYALYES--GELEL-------------------------------- |
| Q2GFD6 | UniRef cluster | -------------------------------------------------------MIKVKIYTKDFCPYCTKAKALFNKKNIPFEEIDITGN---NSLLEQMIQQSNGMKTVPQIFINDQ------HIGGCDDLYKLYES--GKLEL-------------------------------- |
| Q40IH3 | UniRef cluster | -------------------------------------------------------MIKVKIYTKDFCPYCTKAKALFNKKNIPFEEIDITGN---NSLLEQMIQQSNGMKTVPQIFINDQ------HIGGCDDLYKLYES--GKLEL-------------------------------- |
| Q3ICP5 | UniRef cluster | -------------------------------------------------------MTKIEIYSKSYCPYCKRAKATLTRLGLDFEEFEITDS----EKLTKEMQQRSGRKTVPQIFINSQ------HIGGGDDFHHALNS--GSLADLIGDHV-------------------------- |
| Q5WUE2 | UniRef cluster | -------------------------------------------------------MNEVILYTTGYCPYCIKAKELLDRK-KVIYTEIRVDLKPELREEMIQKSG---RRTVPQIFINGQ------AIGGCDDLYALEAQ--GTLNELLKK---------------------------- |
| Q5ZT58 | UniRef cluster | -------------------------------------------------------MNEVILYTTGYCPYCIKAKELLDRK-KVIYTEIRVDLQPELREEMIQKSG---RRTVPQIFINGQ------AIGGCDDLYALEAQ--GTLNELLKK---------------------------- |
| Q5X2Y2 | UniRef cluster | -------------------------------------------------------MNEVILYTTGYCPYCIKAKELLDRK-KVIYTEIRVDLQPELREEMIQKSG---RRTVPQIFINGQ------AIGGCDDLYALEAQ--GTLNELLKK---------------------------- |
| Q2NQW4 | UniRef cluster | -------------------------------------------------------MANIKIYTKATCPYCHRAKALITRK-GVPFQEIPIDGDVDLREEMIKRSG---RTTVPQIFIDGK------HVGGCDDLHALDAR--GGLDPLLK----------------------------- |
| Q0BUK7 | UniRef cluster | ---------------------------------------------------------MIEIYTQPYCPYCSRALALLERK-QVPFKEIQALPGSPARAEARQRSGG--RTSVPQIFIGGR------HIGGCDDMMALEAA--GELDPLLQAA--------------------------- |
| A5IEA5 | UniRef cluster | -------------------------------------------------------MNEVILYTTGYCPYCIKAKELLDRK-KVIYTEIRVDLQPELREEMIQKSG---RRTVPQIFINGQ------AIGGCDDLYALEAQ--GTLNELLKK---------------------------- |
| Q3YQX0 | UniRef cluster | -------------------------------------------------------MTKVIIYTKDPCPYCTKAKALFNKKNILFKEIDVTNN---STLLEEMIQKSNGMRTLPQIFINDQ------HIGGCDDLYRLYES--GKLEL-------------------------------- |
| A3SWN2 | UniRef cluster | ------------------------------------------------------MTAKVTLYTKDYCPHCKAAKALLKSK-GIIFENYEVSTDPLLRAEMIARSG--GRRTVPQIFIGEF------HVGGNSDLVALNAA--GNLDPLLGLGEPA------------------------ |
| Q8D579 | UniRef cluster | -------------------------------------------------------MPKIEIYTKSYCPHCKAAKQTLASMGLVYREIEVSDD----QALFNEMLNRSQRRTVPQIFVGDV------HVGGNQDLITAIRK--GRFEKILRSQAIRH----------------------- |
| Q7MC26 | UniRef cluster | -------------------------------------------------------MPKIEIYTKSYCPHCKAAKQTLASMGLVYREIEVSDD----QALFNEMLNRSQRRTVPQIFVGDV------HVGGNQDLITAIRK--GRFEKILRSQAIRH----------------------- |
| Q2L0A8 | UniRef cluster | -------------------------------------------------------MQKVVMYSKDYCPYCARAEALLRQRGVTEIEKIQIDRDPAQRDVMIERT---GRRTVPQIYIGDT------HVGGCDDLQALDRS--GGLLPLLNG---------------------------- |
| A9FG74 | UniRef cluster | ---------------------------------------------------MPMQAAEVTIYVTDYCPYCAMAKRLLTQKQARFTEINVENRDD-LRAWLVKASG---QRTVPQIFINGA------SIGGFSDLSALDKE--GGLDPRLGEAPSADAPPMPR----------------- |
| Q7WQN4 | UniRef cluster | --------------------------------------------MPAIPSPPGATMQKVVMYSKDYCPYCARAQALLKQRGVADLEIIRIDQDPSQRDIMIERT---GRRTVPQIFIGET------HVGGSDDLQALDRS--GGLLPLLNGG--------------------------- |
| Q7W1Q8 | UniRef cluster | --------------------------------------------MPAIPSPPGATMQKVVMYSKDYCPYCARAQALLKQRGVADLEIIRIDQDPSQRDIMIERT---GRRTVPQIFIGET------HVGGSDDLQALDRS--GGLLPLLNGG--------------------------- |
| Q7VS45 | UniRef cluster | -------------------------------------------------------MQKVVMYSKDYCPYCARAQALLKQRGVADLEIIRIDQDPSQRDIMIERT---GRRTVPQIFIGET------HVGGSDDLQALDRS--GGLLPLLNGG--------------------------- |
| A8EXS7 | UniRef cluster | --------------------------------------------------MNKTILHTIIIYTLASCPYCIKAKALLDKKEVVYEEIEVSNFTQEEKEKLIKKAGG--SRTVPQIFINNI------HIGGNDDLQKLNEE--GRLDKLLEGQPKKTPPAAASV---------------- |
| A1B579 | UniRef cluster | ------------------------------------------------------MMAKIEIYTTPTCPYCIAAKSLLQKK-GITYEETDVSCDPQLRIAMTQRAG--GRRTVPQIFIDGQ------HVGGSDDLHALEHR--GKLDGLLGLTA-------------------------- |
| Q2GIU9 | UniRef cluster | -------------------------------------------------------MRDVVIYTKVPCPYCTRAKALFNKKSIPFKEIDITND---PAAQLEMVERS-GRKTVPQIFIDGE------SIGGCDDLYELYES--GKLEL-------------------------------- |
| A3JMC3 | UniRef cluster | -------------------------------------------------------MQQVEIYTTQLCGFCHRAKGLLKSK-GVSFTEYDVSRDAAKRQEMMQRAK--GGRTVPQIFIGGK------HVGGSDELAALERG--GKLDKILKG---------------------------- |
| Q5LWM3 | UniRef cluster | -------------------------------------------------------MKPVEIYTSPLCGYCHAAKRLLDQK-GIAFTEIDVLTNPKRKPEMIQRAG--GRRTVPQIFIDGQ------HVGGCDDLYALEQD--GKLDPMLASR--------------------------- |
| A9IFJ3 | UniRef cluster | -------------------------------------------------------MDKVVMYSKDYCPYCARAQALLKQRGVTDLEIIRIDQDPAQRDIMIERT---GRRTVPQIFIGER------HIGGCDDLMALDRA--GGLAPLLNG---------------------------- |
| Q5FGI2 | UniRef cluster | ---------------------------MTFIKFLYLLSSITMISTNTWLNIIGYAVTKVIIYTKDFCSYCTKAKALFNRKNIPFEEINITGN---STLKDEMIQKSNGMKTLPQIFINDV------HIGGCDDLYRLYES--GQLKL-------------------------------- |
| Q3KJH7 | UniRef cluster | -------------------------------------------------------MSDVIVYSSDYCPYCSRAKYLLANK-GVAFEEIKVDGKPQVRAAMAQKAG---RTSVPQIWIGDT------HVGGCDDLYALERA--GKLDALLKA---------------------------- |
| Q2GE95 | UniRef cluster | ------------------------------------------------------MNHKVVIYVKEFCPYCSRAKELLDRKGVLYTVVDITND---PDLAVVMMERSGGRKTVPQVFINDV------CVGGFDDLNSLNES--GKLNELLFLNNQ------------------------- |
| A5CD64 | UniRef cluster | -------------MKRILIIVILPIILSYFIIEKTGILEYMKKSDLQDEDFMIYDDDNIYIYTKPTCPYCLNAKSLLNQKSVSFKEIDISNN--QQLHEKLKQATS--QTTVPYIFIYGQ------FIGGYMQLQDLDNT--DKLDELLAQK--------------------------- |
| Q1NCC7 | UniRef cluster | -------------------------------------------------------MAKVEIYTKAWCGYCARAKALLGDK-GVAFDEYDISMGGPTRDEMLKRAP--GQTTVPQIFIDGQ------HIGGSDDLAALNRA--GKLDAMLGQ---------------------------- |
| Q88CX6 | UniRef cluster | -------------------------------------------------------MKPVIVYSSDYCPYCMRAKYLLESK-GVAFEEIKVDGKPQVRAEMSQKAG---RTSVPQIWIGST------HVGGCDDLYALERA--GKLDALLAA---------------------------- |
| Q1IG60 | UniRef cluster | -------------------------------------------------------MKPVIVYSSDYCPYCMRAKYLLESK-GVAFEEIKVDGKPQVRAEMSQKAG---RTSVPQIWIGST------HVGGCDDLYALERA--GKLDALLAA---------------------------- |
| B0KN09 | UniRef cluster | -------------------------------------------------------MKPVIVYSSDYCPYCMRAKYLLESK-GVAFEEVKVDGKPQVRAEMSQKAG---RTSVPQIWIGST------HVGGCDDLYALERA--GKLDALLAA---------------------------- |
| A6G1Q6 | UniRef cluster | ---------------------------------------------MGTFPLPDAIDKDVVIYLTPWCPYCMAARRLLDTR-KVSYEVVDVTGNAAARTWMRQNTG---QSTVPQIFIKGE------SIGGFDELSTLDQR--GGLREMLA----------------------------- |
| A5WA87 | UniRef cluster | -------------------------------------------------------MKPVIVYSSDYCPYCMRAKYLLESK-GVAFEEIKVDGKPQVRAEMSQKAG---RTSVPQIWIGST------HVGGCDDLYALERA--GKLDALLAA---------------------------- |
| A5FYM2 | UniRef cluster | -------------------------------------------------------MPTVEIYTQAFCPYCSRAVRLMREK-GVPFTEIDAPRGSAARREAIERSGG--STTVPQIFIDGQ------SIGGCDELLELERT--GRLDPLLAA---------------------------- |
| A0YA28 | UniRef cluster | -------------------------------------------------------MADVQIYTTRFCPFCIRAKQLLDKK-NVPYNEISVDGRPELRSEMNSRSG---RHTVPQIWIGDQ------HIGGNDELWALDRN--GNLDDLLGA---------------------------- |
| Q5HA67 | UniRef cluster | -----------------------------------------MISTNTWLNIIGYAVTKVIIYTKDFCSYCTKAKALFNRKNIPFEEINITGN---STLKDEMIQKSNGMKTLPQIFINDV------HIGGCDDLYRLYES--GQLKL-------------------------------- |
| A9HJ33 | UniRef cluster | ---------------------------------------------MITGRYSRIRMPRIEIYTQPGCPYCVRALRLLEQK-GTAFTEIRALHGTAERAEARERSGG--RTTVPQIFIDGR------HIGGCDDIMALDRA--GKLDPLLHAA--------------------------- |
| A6FDX8 | UniRef cluster | ------------------------------------------------MAPEYKVQESITVFTKPGCPFCAKAKQTLIDQGLNYEEVVLGKDATTVSLRAVTGQS--------TVPQIFIGG---KHIGGSEELETHFA---------------------------------------- |
| Q0EVS6 | UniRef cluster | -------------------------------------------------------MNKVEVYSGDFCPYCVRAKSLLKKK-GVDFTEYNVQKESDKRIEMLDRSN--GARTIPQIFINDR------HVGGCDELYALEKR--GELDSWLNA---------------------------- |
| A9M8L2 | UniRef cluster | -------------------------------------------------------MVDVIIYTRPGCPYCARAKALLARK-GAEFNEIDASATPELRAEMQERS---GRNTFPQIFIGSV------HVGGCDDLYALEDE--GKLESLLKTGKLI------------------------ |
| Q8YJA2 | UniRef cluster | ---------------------------------------------------MEFLMVDVIIYTRPGCPYCARAKALLARK-GAEFNEIDASATPELRAEMQERS---GRNTFPQIFIGSV------HVGGCDDLYALEDE--GKLDSLLKTGKLI------------------------ |
| Q8FYJ6 | UniRef cluster | -------------------------------------------------------MVDVIIYTRPGCPYCARAKALLARK-GAEFNEIDASATPELRAEMQERS---GRNTFPQIFIGSV------HVGGCDDLYALEDE--GKLDSLLKTGKLI------------------------ |
| Q6G5J5 | UniRef cluster | -------------------------------------------------------MKEIILYTRPNCPYCKRARDLLDKK-GVKYTDIDASTS--LRQEMVQRAN--GRNTFPQIFIGDY------HVGGCDDLYALENK--GKLDSLLQDVH-------------------------- |
| Q57B16 | UniRef cluster | -------------------------------------------------------MVDVIIYTRPGCPYCARAKALLARK-GAEFNEIDASATPELRAEMQERS---GRNTFPQIFIGSV------HVGGCDDLYALEDE--GKLDSLLKTGKLI------------------------ |
| Q2YLN2 | UniRef cluster | -------------------------------------------------------MVDVIIYTRPGCPYCARAKALLARK-GAEFNEIDASATPELRAEMQERS---GRNTFPQIFIGSV------HVGGCDDLYALEDE--GKLDSLLKTGKLI------------------------ |
| Q1D2S0 | UniRef cluster | -------------------------------------------------------MKPVKIFTTTYCGFCVRAKDLLKRKGVDFEEVDVTGDDD-LRAKLVEMSG--GQRTVPQIFIGDT------HVGGYSDLSRLDTE--GRLEPMLQA---------------------------- |
| Q090J7 | UniRef cluster | -------------------------------------------------------MKPVKIYTTTYCGFCVRAKDLLKRKGVNYEELDVTGNDE-MRARLVEMSG--GQRTVPQIFIGDT------HVGGYTDLAQLDRD--GQLEPMLQG---------------------------- |
| B0CID0 | UniRef cluster | -------------------------------------------------------MVDVIIYTRPGCPYCARAKALLARK-GAEFNEIDASATPELRAEMQERS---GRNTFPQIFIGSV------HVGGCDDLYALEDE--GKLDSLLKTGKLI------------------------ |
| A5VSK7 | UniRef cluster | -------------------------------------------------------MVDVIIYTRPGCPYCARAKALLARK-GAEFNEIDASATPELRAEMQERS---GRNTFPQIFIGSV------HVGGCDDLYALEDE--GKLDSLLKTGKLI------------------------ |
| A0Y781 | UniRef cluster | -------------------------------------------------------MSNVVLYTKAYCPFCQRAMALLKSK-GVEFTNFDIGVQPELRDEMITKAG--GASTVPQIFINDE------HIGGCDDMMAIEAQ--GKLDAKLNA---------------------------- |
| Q2G6S1 | UniRef cluster | ----------------------------------------------------MPNMPKVEMYTKWGCPYCFRAKQLLDGK-GVSYEEIDVTMGGPKKTEMLERAP--GHTTVPSIFIDGL------HVGGSDDLAALNAQ--GKLDMMLGL---------------------------- |
| A4C7A3 | UniRef cluster | ------------------------------------------------------MAATVVIYTKDYCPYCIRAKALLSSK-GVPFTEFDIGKQPELRDEMVAKAN--GGYTVPQIFIGDQ------HIGGCDDMMALDSQ--GKLDTLLK----------------------------- |
| A4A8S4 | UniRef cluster | ------------------------------------------------------------MYTTRFCPYCVAAKRLLDAK-GVSYEDIPVDGDAQLRKKMTELAG---QRTVPQIWIGES------HVGGYTDLAALEQR--GLLDGLLSGGPQS------------------------ |
| Q4ZLR2 | UniRef cluster | -------------------------------------------------------MAQVIVYSSDYCPYCIRAKQLLQSK-SVAFEEIRVDGKPQLRAEMTQKAG---RTSVPQIWIGST------HVGGCDDLFALERA--GKLDALLA----------------------------- |
| Q4KJR6 | UniRef cluster | -------------------------------------------------------MSKVIVYSSDYCPYCSRAKHLLASK-GVAFEEIKVDGKPQVRAEMAQKAG---RTSVPQIWIGAT------HVGGCDDLFALERA--GKLDALLAA---------------------------- |
| Q93TF5 | UniRef cluster | -------------------------------------------------------MSHVVVYSSDYCPYCSRAKFLLQNK-GVAFEEIKVDGKPQLRAQMAQKAG---RTSVPQIWIGST------HVGGCDDLFALERA--GKLDALLKA---------------------------- |
| Q4EAC5 | UniRef cluster | -------------------------------------------------------MKNVVIYVKKGCPYCIRAKDLLDKK-GVKYEEIDVLKNSDLFNDIKSKYN---VRTVPQIFINDK------HIGGCDKLMDLEKE--GKLDDMLNNNDNHTDVTTYTNSNDECGECVIPHDDFM |
| A8VEH5 | UniRef cluster | -----------------------------------------------------MSAPTITVYTKQNCPYCVRAKRLLEKKGVAFEEISVEGKDE-LRTWLAEKSG---QLTVPQIFAGER------SLGGFSDLDALEQR--GELDPILRGE--------------------------- |
| A7HB97 | UniRef cluster | -----------------------------------------------------MNAPKVTVYTKRSCPYCVRAKALLARKGVAFQEIDVEGDDA-LRSWLVERSG---QRTVPQVFVGDR------SLGGFMDVDALDRE--GRLDPILRGEAA------------------------- |
| A5V3R0 | UniRef cluster | ----------------------------------------------------MDRMPKVEIYTKAFCPYCSRAKALLETK-GVGFEEYDISMGGPKRAEMIERAR--GGSTVPQIFIDDR------HIGGCDDMFALDRQ--GKLDPLLAA---------------------------- |
| Q2IJG1 | UniRef cluster | -----------------------------------------------------MSAPKITVYTKQNCPYCVRAKRLLEKKGVAFEEISVEGRDE-LRTWLSEKTG---QLTVPQIFAGER------SLGGFSDLDALEQR--GELDPILRGE--------------------------- |
| A4BE90 | UniRef cluster | ------------------------------------------------------MSADIRMYSSQWCPFCIQAKRLLESK-QVSFKEIIVDGDPTLRAQMMQESG---RHTVPQIWINGE------HIGGCDELYTLERN--QKLDSLLTEA--------------------------- |
| A3SKQ4 | UniRef cluster | -------------------------------------------------------MKQVEIYTSPLCGFCHAAKRLLKEK-NVNFTEIDVLENPNRKPEMIQRAN--GGRTVPQIFVGDT------HVGGCDDLYALERA--GKLDALLAA---------------------------- |
| Q7QC85 | UniRef cluster | -------MNVLTRNLAQTLFKYNGAGLIQAAARSFSAPALDGKEIEKLVSNNKVVVFMKGNPDAPRCGFSNAVVQILRMHSVKYDSHD-----VLQNEALRQGIKDFSNWPTIPQVFING-----EFVGGCDILLQMHQN--GELIDELKKAGIESALAKESEK--------------- |
| Q46HJ3 | UniRef cluster | ----------------------------------------------MNRSIPDSGITTVEIYTWRFCPFCLRAKALLNEK-GVQFTEYSIDGDDGARTKMSERAG--GRRTVPQIFINGK------SIGGCDELYELERN--NELNELIGIRN-------------------------- |
| A2C008 | UniRef cluster | ----------------------------------------------MNRSIPDSGITTVEIYTWRFCPFCLRAKALLNEK-GVQFTEYSIDGDDGARTKMSERAG--GRRTVPQIFINGK------SIGGCDELYELERN--NELNELIGIRN-------------------------- |
| Q83BI8 | UniRef cluster | -------------------------------------------------------MAKIEIYTTARCPYCVRAKALLDRK-GLDYMEIRIDEAPEKRDEMLSRSE--GRRTVPQIFINGR------GIGGFDELWELEQS--KKLDELLKT---------------------------- |
| A9ZJG9 | UniRef cluster | -------------------------------------------------------MAKIEIYTTARCPYCVRAKALLDRK-GLDYMEIRIDEAPEKRDEMLSRSE--GRRTVPQIFINGR------GIGGFDELWELEQS--KKLDELLKT---------------------------- |
| A9N958 | UniRef cluster | -------------------------------------------------------MAKIEIYTTARCPYCVRAKALLDRK-GLDYMEIRIDEAPEKRDEMLSRSE--GRRTVPQIFINGR------GIGGFDELWELEQS--KKLDELLKT---------------------------- |
| A9KF83 | UniRef cluster | -------------------------------------------------------MAKIEIYTTARCPYCVRAKALLDRK-GLDYMEIRIDEAPEKRDEMLSRSE--GRRTVPQIFINGR------GIGGFDELWELEQS--KKLDELLKT---------------------------- |
| Q7NYZ6 | UniRef cluster | -------------------------------------------------------MKPVTMYTTAVCPYCVRAKQLLASKGVGGINEIRIDLDPDARDKMMALT---GRRTVPQIFIGDT------HVGGCDDLVALNQA--GKLDPLLAD---------------------------- |
| Q70P99 | UniRef cluster | ------------------------------MSRRAVVRVLAQCLAWDEVREEAPSMSLVTLYTKKDCPYSRGAKALLNQMGIHYEDIDVTYDKR-RLLEMMERSN--GGISVPQIFIAGH------HIGGFSELTRLQQR--GDLTALLGGQEPAPSPS-------------------- |
| A8PKS1 | UniRef cluster | -------------------------------------------------------MQKVVIYTKPDCPYCADAKELFTKKGV-QFEEIQVDKNP-EKLQEMVKLS--NRRSVPQIFINNK------SIGGFEELSKLATS--GELDTLLKTE--------------------------- |
| Q7V3A4 | UniRef cluster | -------------------------------------------------------MSKVEIYTWRFCPFCIRAKSLLEKK-NITFTEHKIDGDDNARELMMERAN--GKRTVPQIFIDDK------SIGGCDELYELEKE--DKLDLLLN----------------------------- |
| A6GLR6 | UniRef cluster | ---------------------------------------------------MSTIQAPVRMYTSAVCPFCVRAERLLNERGVQNIEKIRVDLDPSQKEKMMAET---GRRTVPQIYIGST------HVGGCDDLFDLDRA--GKLLPLLGQPG-------------------------- |
| A2SDN8 | UniRef cluster | -----------------------------------------------------MSSSTVKMFTTQVCPFCIRAKALLKQRGVEQIEEIRIDLDPAQRDAMMQAT---GRRTVPQIFIGDT------HVGGCDELIALDQR--GGLMPLLQQQSPA------------------------ |
| Q488D7 | UniRef cluster | -------------------------------------------------------MAKIEIYTRPGCGYCTHAKRLLTNK-GLDYVEYDVYENPMYIQELQQRTT---GRTYPQIFIEEL------SVGGFTELLENEQF--NLLKK-------------------------------- |
| Q3SG97 | UniRef cluster | -------------------------------------------------------MAKVVMYCTAVCPYCVAAERLLKSRGVAEIEKIRVDLDPSRMDEMIERSG--GRRTVPQVFIGDT------HVGGFDDTSALDAA--GELLPLLARD--------------------------- |
| Q2Y9Z5 | UniRef cluster | ---------------------------------------------------MSEGSAKVLMYSTGFCPYCVMAERLLRARGVEEIEKIRVDLEPARRAEMMEKT---GRRTVPQIYIGDT------HVGGYDDLARLDRN--EGLAKLLAI---------------------------- |
| A8LNA5 | UniRef cluster | ------------------------------------------------------MTRTITLYTKDYCPYCKAAKALLHRK-GARFTNHEITGKPALRAEMIQRAG--GLRTVPQIFIGGV------HVGGYDALTRLDAR--GELDGLLGLVRTA------------------------ |
| B0BWK1 | UniRef cluster | --------------------------------------------------MNKAILHTIIVYTLASCPYCIKAKALLDEKNVAYEEIELSNFTQEEKEKFIKKSGG--KKTVPQIFIDNM------HVGGCDALFDLEKE--GRLDKLLENQPKTTAPAAGA----------------- |
| A8GR54 | UniRef cluster | --------------------------------------------------MNKAILHTIIVYTLASCPYCIKAKALLDEKNVAYEEIELSNFTQEEKEKFIKKSGG--KKTVPQIFIDNM------HVGGCDALFDLEKE--GRLDKLLENQPKTTAPAAGA----------------- |
| A1TTW3 | UniRef cluster | -------------------------------------------------------MQPVKMYTTAVCPYCIRAKQILKAKGVEQIEEIRVDLDPEARSHMMEVT---GRRTVPQIFIGDT------HVGGHDDLVALDGR--GGLMPLLGA---------------------------- |
| Q8YQH1 | UniRef cluster | ------------------------------------------------------MAATVEIYTWRTCPFCIRAKNLLNNK-GVEFVEYSIDGDEEARDKMAQRAN--GRRSLPQIFINDR------HVGGCDDIHALERQ--GQLDELLASSTSL------------------------ |
| Q3MC29 | UniRef cluster | ------------------------------------------------------MAATVEIYTWRTCPFCIRAKNLLNNK-GVEFVEYSIDGDEEARDKMAQRAN--GRRSLPQIFINDR------HVGGCDDIHALERQ--GQLDELLAI---------------------------- |
| A0ZI20 | UniRef cluster | ------------------------------------------------------MAANVEIYTWSTCPFCIRAKSLLKKK-GVDFTEYCIDGDEAERAKMSERAN--GRRSLPQIFINDY------HVGGCDDMHALESQ--GKLDELLASGA-------------------------- |
| Q0VM77 | UniRef cluster | -------------------------------------------------------MARVELYTTSWCPFCVRAKQLLNSK-NVAFEDTDVDREPQQRAVMMQRGG---GRTVPQIFINDH------AVGGCDELFALERA--GALDALLGADQ-------------------------- |
| A8URC9 | UniRef cluster | ---------------------------------------------------MSAQDRHVILLVSQWCPTCPHADALWRKLQEEYGFKYEVLDIATPEGRYWVNKLMVRSVPSSVVDGKLA------FVGVPEEGEARQVIEQGVQTG-------------------------------- |
| Q6F802 | UniRef cluster | ------------------------------------------------------MAANITIYSTTVCPYCIRAKQLLERKGVKYKEINLSNEAPEVRVELMQRTH---HRTVPQIFINDQ------FIGGFDQLYALERD--GKLDELLA----------------------------- |
| B0V4V3 | UniRef cluster | ------------------------------------------------------MAANVIVYSTSVCPYCVRAKQLLERKGVAYKEVNLSVEAPEVRAELMQRTN---HRTVPQIFINDQ------FIGGFDQLYALERE--GKLDELLA----------------------------- |
| A3M238 | UniRef cluster | ------------------------------------------------------MAANVIVYSTSVCPYCVRAKQLLERKGVAYKEVNLSVEAPEVRAELMQRTN---HRTVPQIFINDQ------FIGGFDQLYALERE--GKLDELLA----------------------------- |
| B0CE55 | UniRef cluster | ------------------------------------------------------MTAKVEIYTWAACPFCIQAKQLLDSK-NINFTEYGIDGDEMARSEMAERAF--GRRSLPQIFINDE------HIGGCDDLYDLEGQ--GKLDPLLQT---------------------------- |
| A0YNQ4 | UniRef cluster | ------------------------------------------------------MAAQVEIYTWSTCPFCLRAKSLLKNK-GVDFTEYVIDGDEEARDKMAKRAN--GGRSVPQIFINDQ------HIGGCDDIHALDAQ--GKLDPLLA----------------------------- |
| GLRX1 | UniRef cluster | --------------------------------------------------------MQTVIFGRSGCPYCVRAKDLAEKLSNERDDFQYQYVDIRAEGITKEDLQQKAGKPVETVPQIFVDQ---QHIGGYTDFAAWVKENLDA----------------------------------- |
| GLRX1 | UniRef cluster | --------------------------------------------------------MQTVIFGRSGCPYCVRAKDLAEKLSNERDDFQYQYVDIRAEGITKEDLQQKAGKPVETVPQIFVDQ---QHIGGYTDFAAWVKENLDA----------------------------------- |
| Q3Z3T5 | UniRef cluster | --------------------------------------------------------MQTVIFGRSGCPYCVRAKDLAEKLSNERDDFQYQYVDIRAEGITKEDLQQKAGKPVETVPQIFVDQ---QHIGGYTDFAAWVKENLDA----------------------------------- |
| Q323Q2 | UniRef cluster | --------------------------------------------------------MQTVIFGRSGCPYCVRAKDLAEKLSNERDDFQYQYVDIRAEGITKEDLQQKAGKPVETVPQIFVDQ---QHIGGYTDFAAWVKENLDA----------------------------------- |
| Q0T8K6 | UniRef cluster | --------------------------------------------------------MQTVIFGRSGCPYCVRAKDLAEKLSNERDDFQYQYVDIRAEGITKEDLQQKAGKPVETVPQIFVDQ---QHIGGYTDFAAWVKENLDA----------------------------------- |
| A7ZJR7 | UniRef cluster | --------------------------------------------------------MQTVIFGRSGCPYCVRAKDLAEKLSNERDDFQYQYVDIRAEGITKEDLQQKAGKPVETVPQIFVDQ---QHIGGYTDFAAWVKENLDA----------------------------------- |
| Q8X6S5 | UniRef cluster | --------------------------------------------------------MQTVIFGRPGCPYCVRAKDLAEKLSNERDDFQYQYVDIRAEGITKEDLQQKAGKPVETVPQIFVDQ---QHIGGYTDFAAWVKENLDA----------------------------------- |
| Q8FJF3 | UniRef cluster | ---------------------------------------------------MRREIMQTVIFGRPGCPYCVRAKDLAEKLSNERDDFQYQYVDIRAEGITKEDLQQKAGKPVETVPQIFVDQ---QHIGGYTDFAAWVKENLDA----------------------------------- |
| Q32IC7 | UniRef cluster | --------------------------------------------------------MQTVIFGRPGCPYCVRAKDLAEKLSNERDDFQYQYVDIRAEGITKEDLQQKAGKPVETVPQIFVDQ---QHIGGYTDFAAWVKENLDA----------------------------------- |
| Q0TJJ9 | UniRef cluster | --------------------------------------------------------MQTVIFGRPGCPYCVRAKDLAEKLSNERDDFQYQYVDIRAEGITKEDLQQKAGKPVETVPQIFVDQ---QHIGGYTDFAAWVKENLDA----------------------------------- |
| A1A988 | UniRef cluster | ---------------------------------------------------MRREIMQTVIFGRPGCPYCVRAKDLAEKLSNERDDFQYQYVDIRAEGITKEDLQQKAGKPVETVPQIFVDQ---QHIGGYTDFAAWAKENLDA----------------------------------- |
| Q1RE76 | UniRef cluster | ---------------------------------------------------MRREIMQTVIFGRPGCPYCVRAKDLAEKLSNERDDFQYQYVDIRAEGITKEDLQQKAGKPVETVPQIFVDQ---QHIGGYTDFAAWAKENLDA----------------------------------- |
| A7ZYF2 | UniRef cluster | --------------------------------------------------------MQTVIFGRPGCPYCVRAKDLAEKLSNEHDDFQYHYVDIRAEGITKEDLQQKAGKPVETVPQIFVDQ---QHIGGYTDFAAWVKENLDA----------------------------------- |
| A8AIR8 | UniRef cluster | -------------------------------------------------MSSRKKTMFTVIFGRPGCPYCVRAKELAEKLSTERDDFNYRYIDIHAEGITKADLEKTVGKPVETVPQIFVDQ---KHIGGYTDFEAWAKENLNLFA--------------------------------- |
| A9MIN3 | UniRef cluster | --------------------------------------------------------MFTVIFGRPGCPYCVRAKELAEKLSNERDDFNYRYIDIHAEGITKADLEKTVGKPVETVPQIFVDQ---KHIGGCTDFEAWAKENLNLFA--------------------------------- |
| GLRX1 | UniRef cluster | --------------------------------------------------------MFTVIFGRPGCPYCVRAKELAEKLSKERDDFNYRYIDIHAEGITKADLEKTVGKPVETVPQIFVDQ---KHIGGCTDFEAWAKENLNLFA--------------------------------- |
| GLRX1 | UniRef cluster | --------------------------------------------------------MFTVIFGRPGCPYCVRAKELAEKLSKERDDFNYRYIDIHAEGITKADLEKTVGKPVETVPQIFVDQ---KHIGGCTDFEAWAKENLNLFA--------------------------------- |
| Q5PGP0 | UniRef cluster | --------------------------------------------------------MFTVIFGRPGCPYCVRAKELAEKLSKERDDFNYRYIDIHAEGITKADLEKTVGKPVETVPQIFVDQ---KHIGGCTDFEAWAKENLNLFA--------------------------------- |
| Q57R89 | UniRef cluster | --------------------------------------------------------MFTVIFGRPGCPYCVRAKELAEKLSKERDDFNYRYIDIHAEGITKADLEKTVGKPVETVPQIFVDQ---KHIGGCTDFEAWAKENLNLFA--------------------------------- |
| A9MSN2 | UniRef cluster | --------------------------------------------------------MFTVIFGRPGCPYCVRAKELAEKLSKERDDFNYRYIDIHAEGITKADLEKTVGKPVETVPQIFVDQ---KHIGGCTDFEAWAKENLNLFA--------------------------------- |
| A6T6U5 | UniRef cluster | --------------------------------------------------------MFTVIFGRPGCPYCVRAKELAEKLTNERDDFNYRYVDIHAEGISKADLEKTVGKPVETVPQIFVDQ---KHIGGCTDFEAWAKENLGLFA--------------------------------- |
| Q6LPH3 | UniRef cluster | ---------------------------------------------------MRRDIMFVVIFGRPGCPFCVRAKDLAEKLKEERDDFNFRYVDIHAEGISKSDLEKTVGKPVETVPQIFVDQ---DHVGGYTEFEAYAKENLSLFQ--------------------------------- |
| Q3IJ59 | UniRef cluster | --------------------------------------------------------MLTVIFGREGCPFCVRAKDVAEQLSNERDDFKFRYIDIIKEGISKEDLEKSAGKPCPTVPQIFVDQ---KHIGGFTEFEAYAKENLGLYQ--------------------------------- |
| Q1Z1H8 | UniRef cluster | --------------------------------------------------------MFVVIFGRPGCPFCVRAKDLAEKLKEERDDFNFRYVDIHAEGISKADLEKTVGKPVETVPQIFVDQ---DHVGGYTEFEAYAKENLSLFQ--------------------------------- |
| A8GCA5 | UniRef cluster | --------------------------------------------------------MFAVIFGRPGCPYCVRAKELAEKLTEERDDFNFRYVDIHAEGITKADLEKTVGKPVETVPQIFLDE---KHIGGCTDFEAYAKENLNLFQ--------------------------------- |
| A4W8L5 | UniRef cluster | --------------------------------------------------------MFAVIFGRPGCPYCVRAKELAEKLTAERDDFNFRYVDIHAEGISKSDLEKTVGKPVETVPQIFLDQ---THIGGFTDFEAYAKENLGLFAAQ------------------------------- |
| A1JM48 | UniRef cluster | --------------------------------------------------------MFAVIFGRPGCPYCVRAKELAEKLETERDDFKFRYIDIHAEGITKADLEKTVGKPVETVPQIFIDE---KHIGGCTDFEAYAKENLSLFQ--------------------------------- |
| A4CCU8 | UniRef cluster | --------------------------------------------------------MFTVIFGRDGCPYCVRAKELATKLSNERDDFSFKYVDIIKEGISKADLEKSAGVPVETVPQIFLDQ---KHIGGFTDFEAYAKANLGLYQ--------------------------------- |
| Q7N6G9 | UniRef cluster | --------------------------------------------------------MFTVIFGRPGCPYCVRAKELAERLKEQRDDFDFRYVDIHAEGITKADLEKTVGKPVETVPQIFVDE---KHIGGCTDFEAYVKEHQLLQ---------------------------------- |
| A0Y304 | UniRef cluster | --------------------------------------------------------MLTVIFGREGCPFCVRAKDVAEQLANERDDFKFRYIDIIKEGISKADLEKSAGKPCPTVPQIFVDQ---NHIGGFTEFEAYAKENLGLYK--------------------------------- |
| A0KLI3 | UniRef cluster | --------------------------------------------------------MFAVIFGRPGCPYCVRAKQIAEQLVEQRDDFKFRYVDIHAEGITKEDLAKSAGKPVNTVPQIFLDE---QHIGGCTDFEAYAKENLGLYQ--------------------------------- |
| A4SLJ5 | UniRef cluster | --------------------------------------------------------MFAVIFGRPGCPYCVRAKQIAEQLVEQRDDFNFRYVDIHAEGITKEDLAKSAGKPVNTVPQIFLDE---QHIGGCTDFEAYAKENLGLYQ--------------------------------- |
| GLRX | UniRef cluster | --------------------------------------------------------MFVVIFGRPGCPYCVRAKEHAETLKAKRDDFNYRYVDIHAEGITKADLEKTIGKPVETVPQIFIDE---QHIGGCTDFEAYAKENLGLFD--------------------------------- |
| A6XYR3 | UniRef cluster | ---------------------------------------------------MKEKDMFVVIFGRPGCPYCVRAKEHAETLKAKRDDFNYRYVDIHAEGITKADLEKTIGKPVETVPQIFIDE---QHIGGCTDFEAYAKENLGLFD--------------------------------- |
| A6ABB5 | UniRef cluster | ---------------------------------------------------MKEKDMFVVIFGRPGCPYCVRAKEHAETLKAKRDDFNYRYVDIHAEGITKADLEKTIGKPVETVPQIFIDE---QHIGGCTDFEAYAKENLGLFD--------------------------------- |
| A6A3R3 | UniRef cluster | ---------------------------------------------------MKEKDMFVVIFGRPGCPYCVRAKEHAETLKAKRDDFNYRYVDIHAEGITKADLEKTIGKPVETVPQIFIDE---QHIGGCTDFEAYAKENLGLFD--------------------------------- |
| A5F280 | UniRef cluster | ---------------------------------------------------MKEKDMFVVIFGRPGCPYCVRAKEHAETLKAKRDDFNYRYVDIHAEGITKADLEKTIGKPVETVPQIFIDE---QHIGGCTDFEAYAKENLGLFD--------------------------------- |
| A3GYM9 | UniRef cluster | ---------------------------------------------------MKEKDMFVVIFGRPGCPYCVRAKEHAETLKAKRDDFNYRYVDIHAEGITKADLEKTIGKPVETVPQIFIDE---QHIGGCTDFEAYAKENLGLFD--------------------------------- |
| A3GJ29 | UniRef cluster | ---------------------------------------------------MKEKDMFVVIFGRPGCPYCVRAKEHAETLKAKRDDFNYRYVDIHAEGITKADLEKTIGKPVETVPQIFIDE---QHIGGCTDFEAYAKENLGLFD--------------------------------- |
| A2PUT3 | UniRef cluster | ---------------------------------------------------MKEKDMFVVIFGRPGCPYCVRAKEHAETLKAKRDDFNYRYVDIHAEGITKADLEKTIGKPVETVPQIFIDE---QHIGGCTDFEAYAKENLGLFD--------------------------------- |
| A2PI14 | UniRef cluster | ---------------------------------------------------MKEKDMFVVIFGRPGCPYCVRAKEHAETLKAKRDDFNYRYVDIHAEGITKADLEKTIGKPVETVPQIFIDE---QHIGGCTDFEAYAKENLGLFD--------------------------------- |
| A2P776 | UniRef cluster | ---------------------------------------------------MKEKDMFVVIFGRPGCPYCVRAKEHAETLKAKRDDFNYRYVDIHAEGITKADLEKTIGKPVETVPQIFIDE---QHIGGCTDFEAYAKENLGLFD--------------------------------- |
| A1F5S1 | UniRef cluster | ---------------------------------------------------MKEKDMFVVIFGRPGCPYCVRAKEHAETLKAKRDDFNYRYVDIHAEGITKADLEKTIGKPVETVPQIFIDE---QHIGGCTDFEAYAKENLGLFD--------------------------------- |
| A1EJ99 | UniRef cluster | ---------------------------------------------------MKEKDMFVVIFGRPGCPYCVRAKEHAETLKAKRDDFNYRYVDIHAEGITKADLEKTIGKPVETVPQIFIDE---QHIGGCTDFEAYAKENLGLFD--------------------------------- |
| Q7CHG3 | UniRef cluster | --------------------------------------------------------MFAVIFGRPECPYCVRAKELAEKLASERDDFKFRYVDIHAEGISKADLEKTVGKPVETVPQIFIDE---KHIGGCTDFEAYAKEHLSLYK--------------------------------- |
| Q66CP4 | UniRef cluster | --------------------------------------------------------MFAVIFGRPECPYCVRAKELAEKLASERDDFKFRYVDIHAEGISKADLEKTVGKPVETVPQIFIDE---KHIGGCTDFEAYAKEHLSLYK--------------------------------- |
| Q1CGA0 | UniRef cluster | --------------------------------------------------------MFAVIFGRPECPYCVRAKELAEKLASERDDFKFRYVDIHAEGISKADLEKTVGKPVETVPQIFIDE---KHIGGCTDFEAYAKEHLSLYK--------------------------------- |
| Q1CAD6 | UniRef cluster | --------------------------------------------------------MFAVIFGRPECPYCVRAKELAEKLASERDDFKFRYVDIHAEGISKADLEKTVGKPVETVPQIFIDE---KHIGGCTDFEAYAKEHLSLYK--------------------------------- |
| B0HUE8 | UniRef cluster | --------------------------------------------------------MFAVIFGRPECPYCVRAKELAEKLASERDDFKFRYVDIHAEGISKADLEKTVGKPVETVPQIFIDE---KHIGGCTDFEAYAKEHLSLYK--------------------------------- |
| B0HF96 | UniRef cluster | --------------------------------------------------------MFAVIFGRPECPYCVRAKELAEKLASERDDFKFRYVDIHAEGISKADLEKTVGKPVETVPQIFIDE---KHIGGCTDFEAYAKEHLSLYK--------------------------------- |
| B0H336 | UniRef cluster | --------------------------------------------------------MFAVIFGRPECPYCVRAKELAEKLASERDDFKFRYVDIHAEGISKADLEKTVGKPVETVPQIFIDE---KHIGGCTDFEAYAKEHLSLYK--------------------------------- |
| B0GQR2 | UniRef cluster | --------------------------------------------------------MFAVIFGRPECPYCVRAKELAEKLASERDDFKFRYVDIHAEGISKADLEKTVGKPVETVPQIFIDE---KHIGGCTDFEAYAKEHLSLYK--------------------------------- |
| B0GEV7 | UniRef cluster | --------------------------------------------------------MFAVIFGRPECPYCVRAKELAEKLASERDDFKFRYVDIHAEGISKADLEKTVGKPVETVPQIFIDE---KHIGGCTDFEAYAKEHLSLYK--------------------------------- |
| A9ZV57 | UniRef cluster | --------------------------------------------------------MFAVIFGRPECPYCVRAKELAEKLASERDDFKFRYVDIHAEGISKADLEKTVGKPVETVPQIFIDE---KHIGGCTDFEAYAKEHLSLYK--------------------------------- |
| A9Z3U6 | UniRef cluster | --------------------------------------------------------MFAVIFGRPECPYCVRAKELAEKLASERDDFKFRYVDIHAEGISKADLEKTVGKPVETVPQIFIDE---KHIGGCTDFEAYAKEHLSLYK--------------------------------- |
| A9R4U8 | UniRef cluster | --------------------------------------------------------MFAVIFGRPECPYCVRAKELAEKLASERDDFKFRYVDIHAEGISKADLEKTVGKPVETVPQIFIDE---KHIGGCTDFEAYAKEHLSLYK--------------------------------- |
| A7FK35 | UniRef cluster | --------------------------------------------------------MFAVIFGRPECPYCVRAKELAEKLASERDDFKFRYVDIHAEGISKADLEKTVGKPVETVPQIFIDE---KHIGGCTDFEAYAKEHLSLYK--------------------------------- |
| A6BST8 | UniRef cluster | --------------------------------------------------------MFAVIFGRPECPYCVRAKELAEKLASERDDFKFRYVDIHAEGISKADLEKTVGKPVETVPQIFIDE---KHIGGCTDFEAYAKEHLSLYK--------------------------------- |
| A4TN82 | UniRef cluster | --------------------------------------------------------MFAVIFGRPECPYCVRAKELAEKLASERDDFKFRYVDIHAEGISKADLEKTVGKPVETVPQIFIDE---KHIGGCTDFEAYAKEHLSLYK--------------------------------- |
| Q6D3R3 | UniRef cluster | --------------------------------------------------------MFAVIFGRPACPYCVRAKELAEKLAEQRDDFSFRYVDIHAEGISKEDLSKTVGKPVETVPQIFLDE---KHIGGCTDFEAYAKEHLALFQS-------------------------------- |
| A6D6Y9 | UniRef cluster | --------------------------------------------------------MFVVIFGRPGCPFCVRAKEHAEKLKEKRDDFNYRYVDIHAEGISKADLEKTVGKPVETVPQIFIDQ---EHIGGCTEFEAYAKENLGLFDE-------------------------------- |
| Q2NUH7 | UniRef cluster | --------------------------------------------------------MFAVIFGRPGCPYCVRAKSLAEKLTAERDDFTYRYIDIHAEGISKTDLSKTVGKLVETVPQIFVDQ---IHIGGCTDFEAYAKENLSLYSS-------------------------------- |
| A6VN61 | UniRef cluster | --------------------------------------------------------MFTVIFGRPGCPYCVRAKNLAEKLKNEVADFDYRYVDIHAEGITKEDLSKSVGKPVETVPQIFIDE---KPIGGCTDFEALMKEKFGIVA--------------------------------- |
| Q2BYZ5 | UniRef cluster | --------------------------------------------------------MFVVIFGRPGCPFCVRAKELAEKIKVERDDFNFRYVDIHAEGISKADLEKTVGKPVETVPQIFVDQ---EHIGGCTEFEAYSKEHNLLFQ--------------------------------- |
| Q1ZQR7 | UniRef cluster | --------------------------------------------------------MFVVIFGRPGCPFCVRAKELAEKIKLERDDFNFRYVDIHAEGISKADLEKTVGKPVETVPQIFVDQ---EHIGGCTEFEAYSKEHNLLFQ--------------------------------- |
| A3WL71 | UniRef cluster | --------------------------------------------------------MFTVIFGRPGCPFCVRAKEVAEQLKADRDDFDYRYVDIHEEGITKADLEKTVGKPVHTVPQIFVDE---THVGGFTEFEAYAKEHLGLYAA-------------------------------- |
| Q5QZ55 | UniRef cluster | --------------------------------------------------------MFTVIFGRPGCPFCVRAVEVADKLKADMEDFDYRYVDIHAEGISKADLEKTVGKPVQTVPQIFVDE---QHVGGFTEFEAYAKENLGLYTA-------------------------------- |
| Q5E6G1 | UniRef cluster | --------------------------------------------------------MFVVIFGRPGCPFCVRAKEHAETLTEKRDDFKFRYVDIHAEGISKADLEKTVGKPVETVPQIFIDE---KHIGGCTEFEAYAKENLGLFD--------------------------------- |
| A9IGK6 | UniRef cluster | --------------------------------------------------------MFVVIFGRPGCPFCVRAKEHAETLTEKRDDFKFRYVDIHAEGISKADLEKTVGKPVETVPQIFIDE---KHIGGCTEFEAYAKENLGLFD--------------------------------- |
| Q8D8Y8 | UniRef cluster | --------------------------------------------------------MFVVIFGRPACPYCVRAKEHAETLKAKRDDFNYRYVDIQAEGISKADLEKTVGKPVETVPQIFIDQ---QHIGGCDDFEAYAKEHLGLFD--------------------------------- |
| Q7MLI8 | UniRef cluster | --------------------------------------------------------MFVVIFGRPACPYCVRAKEHAETLKAKRDDFNYRYVDIQAEGISKADLEKTVGKPVETVPQIFIDQ---QHIGGCDDFEAYAKEHLGLFD--------------------------------- |
| A1S7D0 | UniRef cluster | --------------------------------------------------------MYVVIFGRPGCPYCVRAEQLCEQLAEKREDFRFRYVDIHAEGISKEDLSKTVGKPVETVPQIFVDK---THVGGCTDFEAYVREHNLLA---------------------------------- |
| A4P0D2 | UniRef cluster | --------------------------------------------------------MFVVIFGRPGCPYCVRAKNLAEKLKGELADFDYRYVDIHAEGITKEDLSKSVGKPVETVPQIFIDE---KPIGGCTDFEALMKEQFGIVA--------------------------------- |
| GLRX | UniRef cluster | --------------------------------------------------------MFVVIFGRPGCPYCVRAKNLAEKLKGEVADFDYRYVDIHAEGITKEDLSKSVGKPVETVPQIFIDE---KPIGGCTDFEALMKEQFGIVA--------------------------------- |
| Q87QB7 | UniRef cluster | --------------------------------------------------------MFVVIFGRPACPYCVRAKEHAETLKAKRDDFNYRYVDIQAEGISKADLEKTVGKPVETVPQIFIDQ---EHIGGCDDFEAYAKEHLGLFDE-------------------------------- |
| Q4QKP4 | UniRef cluster | --------------------------------------------------------MFVVIFGRPGCPYCVRAKNLAEKLKGEVADFDYRYVDIHAEGITKEDLSKSVGKPVETVPQIFIDE---KPIGGCTDFEALMKEQFGIVA--------------------------------- |
| A6B943 | UniRef cluster | --------------------------------------------------------MFVVIFGRPACPYCVRAKEHAETLKAKRDDFNYRYVDIQAEGISKADLEKTVGKPVETVPQIFIDQ---EHIGGCDDFEAYAKEHLGLFDE-------------------------------- |
| A5UCC7 | UniRef cluster | --------------------------------------------------------MFVVIFGRPGCPYCVRAKNLAEKLKGEVADFDYRYVDIHAEGITKEDLSKSVGKPVETVPQIFIDE---KPIGGCTDFEALMKEQFGIVA--------------------------------- |
| A4NMR7 | UniRef cluster | --------------------------------------------------------MFVVIFGRPGCPYCVRAKNLAEKLKGEVADFDYRYVDIHAEGITKEDLSKSVGKPVETVPQIFIDE---KPIGGCTDFEALMKEQFGIVA--------------------------------- |
| A4NFG7 | UniRef cluster | --------------------------------------------------------MFVVIFGRPGCPYCVRAKNLAEKLKGEVADFDYRYVDIHAEGITKEDLSKSVGKPVETVPQIFIDE---KPIGGCTDFEALMKEQFGIVA--------------------------------- |
| A4N9S0 | UniRef cluster | --------------------------------------------------------MFVVIFGRPGCPYCVRAKNLAEKLKGEVADFDYRYVDIHAEGITKEDLSKSVGKPVETVPQIFIDE---KPIGGCTDFEALMKEQFGIVA--------------------------------- |
| B0TPY2 | UniRef cluster | --------------------------------------------------------MFVVIFGRPGCPYCVRAVQVAEQLTEKRDDFKFKYVDIHAEGISKADLEKTVGKPVETVPQIFVDQ---EHVGGCTEFEQYVRDNELMPAA-------------------------------- |
| A9KY54 | UniRef cluster | --------------------------------------------------------MFVVIFGRPGCPYCVRAVQLSEQLVEKRDDFKFRYVDIHAEGISKADLEKTVGKPVETVPQIFVDE---KHVGGCTDFEQYVRENNLLD---------------------------------- |
| A6WM50 | UniRef cluster | --------------------------------------------------------MFVVIFGRPGCPYCVRAVQLSEQLVEKRDDFKFRYVDIHAEGISKADLEKTVGKPVETVPQIFVDE---KHVGGCTDFEQYVRENNLLD---------------------------------- |
| A5NIM8 | UniRef cluster | --------------------------------------------------------MFVVIFGRPGCPYCVRAVQLSEQLVEKRDDFKFRYVDIHAEGISKADLEKTVGKPVETVPQIFVDE---KHVGGCTDFEQYVRENNLLD---------------------------------- |
| A4N0R2 | UniRef cluster | --------------------------------------------------------MFVVIFGRPGCPYCVRAKNLAEKLKGEVADFGYRYVDIHAEGITKEDLSKSVGKPVETVPQIFIDE---KPIGGCTDFEALMKEQFGIVA--------------------------------- |
| A3QD50 | UniRef cluster | --------------------------------------------------------MFVVIFGRPGCPYCVRAVQLSEQLVESRDDFKFKYVDIHAEGITKADLEKTVGKPVETVPQIFIDQ---EHIGGCTEFEQYVRDNNLL----------------------------------- |
| A3D3E6 | UniRef cluster | --------------------------------------------------------MFVVIFGRPGCPYCVRAVQLSEQLVEKRDDFKFRYVDIHAEGISKADLEKTVGKPVETVPQIFVDE---KHVGGCTDFEQYVRENNLLD---------------------------------- |
| Q8EDK1 | UniRef cluster | --------------------------------------------------------MYVVIFGRPGCPYCVRAVQLSEQLVEKRDDFKFRYVDIHAEGISKADLEKTVGKPVETVPQIFVDE---KHVGGCTDFEQYVRDNNLLG---------------------------------- |
| Q1QWI3 | UniRef cluster | --------------------------------------------------------MLAVIFGRPGCPFCVRAKELAEQLSEARDDFNFRYIDIHEEGITKADMEKTIGKPVETVPQIFVDQ---THIGGFTEFDEHVRANELMPATQR------------------------------ |
| Q0HTY4 | UniRef cluster | --------------------------------------------------------MYVVIFGRPGCPYCVRAVQLSEQLVEKRDDFKFRYVDIHAEGISKADLEKTVGKPVETVPQIFVDE---KHVGGCTDFEQYVRDNNLLG---------------------------------- |
| Q0HHN2 | UniRef cluster | --------------------------------------------------------MYVVIFGRPGCPYCVRAVQLSEQLVEKRDDFKFRYVDIHAEGISKADLEKTVGKPVETVPQIFVDE---KHVGGCTDFEQYVRDNNLLG---------------------------------- |
| A3XX97 | UniRef cluster | --------------------------------------------------------MFVVIFGRPACPFCVRAKEHAETLKAKRDDFNYRYVDIHAEGISKADLEKTVGKPVDTVPQIFIDQ---DHIGGCTEFEAYAKENLGLFD--------------------------------- |
| A0KY85 | UniRef cluster | --------------------------------------------------------MYVVIFGRPGCPYCVRAVQLSEQLVEKRDDFKFRYVDIHAEGISKADLEKTVGKPVETVPQIFVDE---KHVGGCTDFEQYVRDNNLLG---------------------------------- |
| A4Y7Y9 | UniRef cluster | --------------------------------------------------------MFVVIFGRPGCPYCVRAVQLSEQLVEKRDDFKFRYVDIHAEGISKADLEKTVGKPVETVPQIFVDE---KHVGGCTDFEQYVRDNNLLG---------------------------------- |
| A2V3Y6 | UniRef cluster | --------------------------------------------------------MFVVIFGRPGCPYCVRAVQLSEQLVEKRDDFKFRYVDIHAEGISKADLEKTVGKPVETVPQIFVDE---KHVGGCTDFEQYVRDNNLLD---------------------------------- |
| A1RIJ9 | UniRef cluster | --------------------------------------------------------MFVVIFGRPGCPYCVRAVQLSEQLVEKRDDFKFRYVDIHAEGISKADLEKTVGKPVETVPQIFVDE---KHVGGCTDFEQYVRDNNLLG---------------------------------- |
| A8H5P3 | UniRef cluster | --------------------------------------------------------MFVVIFGRPGCPYCVRAVQVAEQLTEKRDDFKFKYVDIHAEGISKADLEKTVGKPVETVPQIFVDQ---VHVGGCTEFVQYVSDNELMPAA-------------------------------- |
| A4NRJ1 | UniRef cluster | --------------------------------------------------------MFVTIFGRPGCPYCVRAKNLAERLKGEVADFDYRYVDIHAEGITKEDLSKSVGKPVETVPQIFIDE---KPIGGCTDFEALMKEQFGIVA--------------------------------- |
| Q2SEA7 | UniRef cluster | --------------------------------------------------------MYTVIFGRSGCPYCVRAKQIAEQLKDQRQDFDFRYIDIHEEGISKADLEKTVGKPVLTVPQIFIDK---EHIGGCNEFENYVRQNLTH----------------------------------- |
| A7MSH8 | UniRef cluster | --------------------------------------------------------MFVVIFGRPACPFCVRAKEHAETLKAKRDDFNYRYVDIHAEGISKADLEKTVGKPVETVPQIFIDQ---THIGGCDDFEAYAKEHLGLFDE-------------------------------- |
| A6AV76 | UniRef cluster | --------------------------------------------------------MFVVIFGRPACPFCVRAKEHAETLKAKRDDFNYRYVDIHAEGISKADLEKTVGKPVETVPQIFIDQ---THIGGCDDFEAYAKEHLGLFDE-------------------------------- |
| A5UEH3 | UniRef cluster | --------------------------------------------------------MFVTIFGRPGCPYCVRAKNLAEKLKGKVADFDYRYVDIHAEGITKEDLSKSVGKPVETVPQIFIDE---KPIGGCTDFEALMKEQFGIVA--------------------------------- |
| Q1V7R2 | UniRef cluster | --------------------------------------------------------MFVVIFGRPACPFCVRAKEHAETLKAKRDDFNYRYVDIHAEGISKADLEKTVGKPVETVPQIFIDQ---EHIGGCDEFEAYAKEHLGLFDE-------------------------------- |
| Q65UJ8 | UniRef cluster | --------------------------------------------------------MFVVIFGRPGCPYCVRAKNLAEKLKNSLDDFDYRYVDIIAEGISKADLSKSVGKEVETVPQIFIDE---KPIGGCTDFEALMKEQFNIVA--------------------------------- |
| Q0I336 | UniRef cluster | --------------------------------------------------------MYVVIFGRSGCPYCVRAKNLAEKLKNTLEDFDYRYIDIVAEGISKADLSLSVGKPVETVPQIFIDE---KPIGGCTDFEALMKAQFNVVA--------------------------------- |
| B0UT29 | UniRef cluster | --------------------------------------------------------MYVVIFGRSGCPYCVRAKNLAEKLKNTLEDFDYRYIDIVAEGISKADLSLSVGKPVETVPQIFIDE---KPIGGCTDFEALMKAQFNVVA--------------------------------- |
| A8T1W0 | UniRef cluster | --------------------------------------------------------MFVVIFGRPACPFCVRAKEHAETLKAKRDDFNYRYVDIHAEGISKADLEKTVGKPVETVPQIFIDQ---DHIGGCDEFEAYAKEHLGLFDE-------------------------------- |
| A9DEN6 | UniRef cluster | -------------------------------------MQFKRDYGIKVATKFSGVHMFVVIFGRPGCPYCVRAVQLSEQLVEARDDFKFKYIDIHAEGISKADLEKTVGKPVETVPQIFIDK---EHVGGCTEFEQYVRDNQLLSTPA------------------------------- |
| A8FUE9 | UniRef cluster | --------------------------------------------------------MFVVIFGRPGCPYCVRAVQLSEQLVEAREDFKFKYVDIHAEGISKADLEKTVGKPVETVPQIFVDK---EHVGGCTEFEQFVRENQLLTQPA------------------------------- |
| A6FE29 | UniRef cluster | --------------------------------------------------------MFTVIFGRPGCPYCVRAKQIAETLKAEHEDFDFNYIDMLAEGISKADLEKSAGVPVNTVPQIFIDE---KHVGGCTEFEQFAKENLGLYA--------------------------------- |
| Q084J7 | UniRef cluster | --------------------------------------------------------MFVVIFGRPGCPYCVRAVELSEKLAAQSEDFKFKYIDIHAEGITKADLSKSVGKTVETVPQIFVDE---QPIGGCTEFEAYVRQNNLLN---------------------------------- |
| Q12MF4 | UniRef cluster | --------------------------------------------------------MFVVIFGRPGCPYCVRAVELAEKLTEQREDFKFKYVDIHAEGISKADLSKSVGKTVETVPQIFVDE---VPVGGCTEFEQYVRENKLLS---------------------------------- |
| Q9CMJ7 | UniRef cluster | --------------------------------------------------------MFVVIFGRAGCPYCVRAKNLADKLKQSLPDFDYRYVDIVAEGISKADLSKSVGKPVETVPQIFIDE---KPIGGCTDFEALMKAQFNIVA--------------------------------- |
| A7JX81 | UniRef cluster | --------------------------------------------------------MFVEIYGRLSCPYCVRAKTLAEKMKQELSDFDFKFIDMIAENISKQDLEPRVGKPVATVPQIFVDN---NHVGGCTDFQAFVEEKFGITV--------------------------------- |
| B0BRL2 | UniRef cluster | --------------------------------------------------------MFVEIYGRMTCPYCTRAKALAEKMKGELADFDFKFIDMIAEGISKEDLEPRVGKPVATVPQIFLDN---VHVGGCTDFQALVKEKFGIEV--------------------------------- |
| A3MYC6 | UniRef cluster | --------------------------------------------------------MFVEIYGRMTCPYCTRAKALAEKMKGELADFDFKFIDMIAEGISKEDLEPRVGKPVATVPQIFLDN---VHVGGCTDFQALVKEKFGIEV--------------------------------- |
| B0QSV2 | UniRef cluster | --------------------------------------------------------MFVEIYGRLSCPYCVRAKQLAEKMKAELPDFDFKFVDMIAEGIEKQNLEPRVGKPVATVPQIFLDE---VHVGGYSDFAPLIEEKFGIKL--------------------------------- |
| A4N721 | UniRef cluster | --------------------------------------------------------MFVVIFGRPGCPYCVRAKNLAEKLKGEVADFDYRYVDIHAEGITKEDLSKSVGKPVETVPQIFY----------------------------------------------------------- |
| Q7VP11 | UniRef cluster | --------------------------------------------------------MLVEIYGCLSCLYCVRAKQLAEKMAIELADFEFKFIDMIAEGISKQDLAVRVGKAVDTVPQIFLDD---QAVGGCTEFQQLVKQKFDIEL--------------------------------- |
| Q2BKD5 | UniRef cluster | -------------------------------------------------------MKRITIFGREGCGFCKRAKEVCEAKN-----LDFRYVDIHKEGISKADLEKTVGKPVETVPQVFCGQ---EHIGGYTELAAFLEAEGV------------------------------------ |
| A3JA59 | UniRef cluster | -------------------------------------------------------MEKVTIFGRTSCGFCVGAIRLCEARD-----FEFKWVDMIEEDITKADIAQKIGKPVHTVPQIFVGE---QHIGGYDEFSAFARQQETADH--------------------------------- |
| A6F3U3 | UniRef cluster | -------------------------------------------------------MDHVTIYGRASCGFCMRARQLCEIRD-----IPYRYVDMIEEGISKADLEKTTGKPVHTVPQIFVGD---EHVGGFDHFSRYVQQFESASR--------------------------------- |
| Q935D9 | UniRef cluster | --------------------------------------------------------MKVVIYGRDNCSYCKRAVELAKQLRG-HGFGDYEYIDIVSAGIDKEKLSYLVSKPVETIPQVFVNG---ESIGGYEEFAALVSTL-------------------------------------- |
| A1U3B0 | UniRef cluster | -------------------------------------------------------MEQVTIYGRSSCGFCVRARDLCESRN-----IPYVWVDMIEKGMSKQDIADRIGHPVYTVPQILVGS---EYVGGFDDFSAYVRRHEAQTAS-------------------------------- |
| A3YCV9 | UniRef cluster | -------------------------------------------------------MSRYTIFGHDNCGFCRRAKQLLDEQG-----LPYRYVNIHDEGITQDALSALVGKDVRTVPQIFKGK---EYVGGFDDLNASLDTKAA------------------------------------ |
| Q5NHD0 | UniRef cluster | --------------------------------------------------------MKVKIYTRNGCPYCVWAKQWFEENNIAFDETIIDDYAQRSKFYDEMNQSGKVIFPISTVPQIFIDD---EHIGGFTELKANADKILNKK---------------------------------- |
| Q14IT2 | UniRef cluster | --------------------------------------------------------MKVKIYTRNGCPYCVWAKQWFEENNIAFDETIIDDYAQRSKFYDEMNQSGKVIFPISTVPQIFIDD---EHIGGFTELKANADKILNKK---------------------------------- |
| A7YTV5 | UniRef cluster | --------------------------------------------------------MKVKIYTRNGCPYCVWAKQWFEENNIAFDETIIDDYAQRSKFYDEMNQSGKVIFPISTVPQIFIDD---EHIGGFTELKANADKILNKK---------------------------------- |
| A7JM21 | UniRef cluster | --------------------------------------------------------MKVKIYTRNGCPYCVWAKQWFEENNIAFDETIIDDYAQRSKFYDEMNQSGKVIFPISTVPQIFIDD---EHIGGFTELKANADKILNKK---------------------------------- |
| A7JHX5 | UniRef cluster | --------------------------------------------------------MKVKIYTRNGCPYCVWAKQWFEENNIAFDETIIDDYAQRSKFYDEMNQSGKVIFPISTVPQIFIDD---EHIGGFTELKANADKILNKK---------------------------------- |
| A7JBI1 | UniRef cluster | --------------------------------------------------------MKVKIYTRNGCPYCVWAKQWFEENNIAFDETIIDDYAQRSKFYDEMNQSGKVIFPISTVPQIFIDD---EHIGGFTELKANADKILNKK---------------------------------- |
| A0Q6K5 | UniRef cluster | --------------------------------------------------------MKVKIYTRNGCPYCVWAKQWFEENNIAFDETIIDDYAQRSKFYDEMNQSGKVIFPISTVPQIFIDD---EHIGGFTELKANADKILNKK---------------------------------- |
| Q2A3K9 | UniRef cluster | --------------------------------------------------------MKVKIYIRNGCPYCVWAKQWFEENNIAFDETIIDDYAQRSKFYDEMNQSGKVIFPISTVPQIFIDD---EHIGGFTELKANADKILNKK---------------------------------- |
| Q0BM19 | UniRef cluster | --------------------------------------------------------MKVKIYIRNGCPYCVWAKQWFEENNIAFDETIIDDYAQRSKFYDEMNQSGKVIFPISTVPQIFIDD---EHIGGFTELKANADKILNKK---------------------------------- |
| B0TZT0 | UniRef cluster | --------------------------------------------------------MKVKIYTRNGCPFCVWAKQWFDENEIVFEETIIDDHSQRLRFYEEMNQSGKVAFPISTVPQIFIDD---EHIGGFTDLKANATEILAKK---------------------------------- |
| A7NC11 | UniRef cluster | --------------------------------------------------------MKVKIYIRNGCPYCVWAKQWFEENNIAFDETIIDDYAQRSKFYDEMNQSGKVIFPISTVPQIFIDD---EHIGGFTELKANADKILNKK---------------------------------- |
| A4KRC8 | UniRef cluster | --------------------------------------------------------MKVKIYIRNGCPYCVWAKQWFEENNIAFDETIIDDYAQRSKFYDEMNQSGKVIFPISTVPQIFIDD---EHIGGFTELKANADKILNKK---------------------------------- |
| Q5E4Q5 | UniRef cluster | ----------------------------------------------------MLNTHQTIIFSKTVCPFCVKAKAILDDKGIEYKVLTLDEDLTKEEMVALIQEKE--NITVNTVPQIYLDR---KYIGGHDDLVAFFERQDTDMDLGDFEL--------------------------- |
| A9IBV2 | UniRef cluster | ----------------------------------------------------MLNTHQTIIFSKTVCPFCVKAKAILDDKGIEYKVLTLDEDLTKEEMVALIQEKE--NIAVNTVPQIYLDR---KYIGGHDDLVAFFERQDTDMDLGDFEL--------------------------- |
| Q73H18 | UniRef cluster | -------------------------------------------------------MKNVVIYVKKGCPYCIRGKDLLDKK-GVKYEEIDVLKNSDLFNDIKSKYN---VRTVPQIFINDK------HIGGCDKLMDLEKE--GKLDDMLNNNDNHTDVTTYTNSNDECGECVIPHDDFM |
| A4IY37 | UniRef cluster | --------------------------------------------------------MKVKIYTRNGCQYCVWAKQWFEENNIAFDETIIDDYAQRSKFYDEMNQSGKVIFPISTVPQIFIDD---EHIGGFTELKANADKILNKK---------------------------------- |
| A6WXP6 | UniRef cluster | -------------------------------------------------------MVDVTIYTRVGCPYCTRAKDLLTRK-GVAYNEIDAGASPELRAEMQQRS---GRNTFPQIFVGSV------HVGGCDDLLELEDQ--GKLDGLLKTGELV------------------------ |
| A0L3B6 | UniRef cluster | ----------------------------------------------------MKALKNVVIYTKDHCPFCARVKNYLTAE-KVDFKQIRVDDDPKTYLELKERTN---LQTVPQVFVDGE------FIGSATDFFSWIDS--------------------------------------- |
| Q6G0I2 | UniRef cluster | -------------------------------------------------------MKEVTLYTRPNCPYCAKARDLLDKK-GVKYTDIDASTV--LREEMVQRAN--GRNTFPQIFIGDY------HVGGCDDLYALNAK--GELNSLLQSVE-------------------------- |
| A9IQF1 | UniRef cluster | -------------------------------------------------------MKEIIIYTRPGCPYCTKARDLLDKK-DVKYKDIDASTS--LRQEMVQRAN--GRNTFPQIFIGDY------HVGGCDDLYALEAE--GKLNSLLQDL--------------------------- |
| Q2BP81 | UniRef cluster | --------------------------------------------------------MKVEIYTKGYCPYCRAAKKLLKQLNWEYKEFEITNR----PALQKEMKLRSRRHTVPQIFINNQ------HIGGFDDFSVFLSN--KVS---------------------------------- |
| A3WC26 | UniRef cluster | -----------------------------------------------------MSTPQIDMYTKFACPFCVRAKHLLQKK-GVEFNEYDITMGGPKREEMMERAP--LARTVPQIFIGDV------HVGGSDDLAALEEA--GKLDALLAG---------------------------- |
| A1WDX6 | UniRef cluster | -------------------------------------------------------MQAVKMYTTAVCPYCVRAKQLLKARGVEQIEELRIDADPAARQQMMALT---GRRTVPQIFIGQT------HVGGYDDLVALDGR--GQLMPLLGAA--------------------------- |
| A7CAG7 | UniRef cluster | -------------------------------------------------------MAHVVMYSTTVCPYCVAAERLLKQRGVEQIEKILIDREPGKREEMMTRT---NRRTVPQIYIDER------HIGGFDDLSALDRE--GGLVPLLAA---------------------------- |
| A1HCE7 | UniRef cluster | -------------------------------------------------------MAHVVMYSTTVCPYCVAAERLLKQRGVEQIEKILIDREPGKREEMMTRT---NRRTVPQIYIDER------HIGGFDDLSALDRE--GGLVPLLAA---------------------------- |
| GLRX1 | UniRef cluster | --------------------------------------------------MNKSILHTIIIYTLASCPYCIKAKALLDKKNVIYEEIEVSNLTQEEKEKFIKKSGG--KSTVPQIFIDNM------HVGGCDDLFNLEKE--GRLDKLLEHQPKN------------------------ |
| Q8XG98 | UniRef cluster | -------------------------------------------------------MANIEIYTKATCPFCHRAKALLNSK-GVSFQEIAIDGDAVKREEMIKRSG---RTTVPQIFIDAQ------HIGGCDDLYALDAR--GGLDPLLR----------------------------- |
| Q7CPH7 | UniRef cluster | -------------------------------------------------------MANIEIYTKATCPFCHRAKALLNSK-GVSFQEIAIDGDAVKREEMIKRSG---RTTVPQIFIDAQ------HIGGCDDLYALDAR--GGLDPLLR----------------------------- |
| Q5PBZ4 | UniRef cluster | -------------------------------------------------------MANIEIYTKATCPFCHRAKALLNSK-GVSFQEIAIDGDAVKREEMIKRSG---RTTVPQIFIDAQ------HIGGCDDLYALDAR--GGLDPLLR----------------------------- |
| Q57ID1 | UniRef cluster | -------------------------------------------------------MANIEIYTKATCPFCHRAKALLNSK-GVSFQEIAIDGDAVKREEMIKRSG---RTTVPQIFIDAQ------HIGGCDDLYALDAR--GGLDPLLR----------------------------- |
| A9MVK4 | UniRef cluster | -------------------------------------------------------MANIEIYTKATCPFCHRAKALLNSK-GVSFQEIAIDGDAVKREEMIKRSG---RTTVPQIFIDAQ------HIGGCDDLYALDAR--GGLDPLLR----------------------------- |
| A9MKR4 | UniRef cluster | -------------------------------------------------------MANIEIYTKATCPFCHRAKALLNSK-GVSFQEIAIDGDAVKREEMIKRSG---RTTVPQIFIDAQ------HIGGCDDLYALDAR--GGLDPLLR----------------------------- |
| GLRX3 | UniRef cluster | -------------------------------------------------------MANVEIYTKETCPYCHRAKALLSSK-GVSFQELPIDGNAAKREEMIKRSG---RTTVPQIFIDAQ------HIGGCDDLYALDAR--GGLDPLLK----------------------------- |
| GLRX3 | UniRef cluster | -------------------------------------------------------MANVEIYTKETCPYCHRAKALLSSK-GVSFQELPIDGNAAKREEMIKRSG---RTTVPQIFIDAQ------HIGGCDDLYALDAR--GGLDPLLK----------------------------- |
| GLRX3 | UniRef cluster | -------------------------------------------------------MANVEIYTKETCPYCHRAKALLSSK-GVSFQELPIDGNAAKREEMIKRSG---RTTVPQIFIDAQ------HIGGCDDLYALDAR--GGLDPLLK----------------------------- |
| Q3YVX5 | UniRef cluster | -------------------------------------------------------MANVEIYTKETCPYCHRAKALLSSK-GVSFQELPIDGNAAKREEMIKRSG---RTTVPQIFIDAQ------HIGGCDDLYALDAR--GGLDPLLK----------------------------- |
| Q329P4 | UniRef cluster | -------------------------------------------------------MANVEIYTKETCPYCHRAKALLSSK-GVSFQELPIDGNAAKREEMIKRSG---RTTVPQIFIDAQ------HIGGCDDLYALDAR--GGLDPLLK----------------------------- |
| Q31V11 | UniRef cluster | -------------------------------------------------------MANVEIYTKETCPYCHRAKALLSSK-GVSFQELPIDGNAAKREEMIKRSG---RTTVPQIFIDAQ------HIGGCDDLYALDAR--GGLDPLLK----------------------------- |
| Q1R4Y4 | UniRef cluster | -------------------------------------------------------MANVEIYTKETCPYCHRAKALLSSK-GVSFQELPIDGNAAKREEMIKRSG---RTTVPQIFIDAQ------HIGGCDDLYALDAR--GGLDPLLK----------------------------- |
| Q0TBJ6 | UniRef cluster | -------------------------------------------------------MANVEIYTKETCPYCHRAKALLSSK-GVSFQELPIDGNAAKREEMIKRSG---RTTVPQIFIDAQ------HIGGCDDLYALDAR--GGLDPLLK----------------------------- |
| Q0SYD6 | UniRef cluster | -------------------------------------------------------MANVEIYTKETCPYCHRAKALLSSK-GVSFQELPIDGNAAKREEMIKRSG---RTTVPQIFIDAQ------HIGGCDDLYALDAR--GGLDPLLK----------------------------- |
| A8ARJ7 | UniRef cluster | -------------------------------------------------------MANIEIYTKVTCPFCHRAKALLSSK-GVSFQELPIDGDAVKREEMIKRSG---RTTVPQIFIDAQ------HIGGCDDLYALDAR--GGLDPLLR----------------------------- |
| A8A675 | UniRef cluster | -------------------------------------------------------MANVEIYTKETCPYCHRAKALLSSK-GVSFQELPIDGNAAKREEMIKRSG---RTTVPQIFIDAQ------HIGGCDDLYALDAR--GGLDPLLK----------------------------- |
| A7ZTG4 | UniRef cluster | -------------------------------------------------------MANVEIYTKETCPYCHRAKALLSSK-GVSFQELPIDGNAAKREEMIKRSG---RTTVPQIFIDAQ------HIGGCDDLYALDAR--GGLDPLLK----------------------------- |
| A6TFK5 | UniRef cluster | -------------------------------------------------------MANIEIYTKATCPFCIRAKALLNSK-GVTFHELPIDGDAAKREEMIQRSG---RTTVPQIFIDAQ------HIGGCDDLYALDSR--GGLDPLLR----------------------------- |
| Q8Y2I1 | UniRef cluster | -------------------------------------------------------MAHVVMYSTTVCPYCVAAEKLLKQRGVAHIEKILIDREPGRREEMMTRT---NRRTVPQIYIDDR------HIGGFDDLSALDRE--GGLEPLLAA---------------------------- |
| Q1ZSE1 | UniRef cluster | ------------------------------------------MTEVKNLTIEQLEQYDNLVITQPNCPFCVKAKALLDERGIEYTTLVLGLNLSKLVMVDFIEKKT--GQTVRTVPQIILDD---KYIGGHDDLVAFLERQVEATEFDDFEL--------------------------- |
| A3RSA9 | UniRef cluster | -------------------------------------------------------MAHVVMYSTTVCPYCVAAEKLLKQRGVAHIEKILIDREPGRREEMMVRT---NRRTVPQIYIDDR------HIGGFDDLSALDRE--GGLEPLLAA---------------------------- |
| GLRX1 | UniRef cluster | --------------------------------------------------MNKSILHTIIIYTLASCPYCIKAKALLDKKNVIYEEIEVSNFTQEEKEAFIKKSGG--KNTVPQIFIDNM------HVGGCDDLFNLEQD--GRLDKLLETQPKNKNSLTVSGA--------------- |
| Q83PP6 | UniRef cluster | -------------------------------------------------------MANVEIYTKETCPYCHRAKALLSSK-GVSFQELPIDGNAAKREEMIKRSG---CTTVPQIFIDAQ------HIGGCDDLYALDAR--GGLDPLLK----------------------------- |
| A6VDP7 | UniRef cluster | -------------------------------------------------------MPPVVIYTTAWCPYCIRAKQLLQRK-GVDFQEIACDGKPELRAELARKAG---SSTVPQIWIGET------HVGGCDDLHALERA--GKLDALLSA---------------------------- |
| A5P7P8 | UniRef cluster | -----------------------------------------------------MSQPHIDIYTKFGCGFCVRAKRLLDEK-GADYHEHDITMGGPKREEMLQRAP--QARTVPQIFIGET------HVGGSDELAALERS--GKLDPLLEG---------------------------- |
| A3LJ01 | UniRef cluster | -------------------------------------------------------MPPVVIYTTAWCPYCIRAKQLLQRK-GVDFQEIACDGKPELRAELARKAG---STTVPQIWIGEN------HVGGCDDLHALERA--GKLDALLSA---------------------------- |
| GLRX | UniRef cluster | -------------------------------------------------------MPPVVIYTTAWCPYCIRAKQLLQRK-GVDFQEIACDGKPELRAELARKAG---STTVPQIWIGET------HVGGCDDLHALERA--GKLDALLSA---------------------------- |
| Q02EN7 | UniRef cluster | -------------------------------------------------------MPPVVIYTTAWCPYCIRAKQLLQRK-GVDFQEIACDGKPELRAELARKAG---STTVPQIWIGET------HVGGCDDLHALERA--GKLDALLSA---------------------------- |
| A8F0W0 | UniRef cluster | -------------------------------------------------MMNKAILHTIIIYTLASCPYCIKAKALLDEKNVAYEEIEVSNFTQEEKEKFIKKSGG--KKTVPQIFIDNM------HVGGCDALFDLEKE--GRLDKLLENQPKKTSPAAGA----------------- |
| GLRX1 | UniRef cluster | --------------------------------------------------MNKAILHTIIVYTLASCPYCIKAKALLDEKNVAYEEIEVSNFTQEEKEKFIKKSGG--KKTVPQIFIDNM------HVGGCDALFDLEKE--GRLDKLLENQPKTTSPAAGA----------------- |
| Q8D215 | UniRef cluster | -------------------------------------------------------MKNVEIYIKSTCSFCVKAKNLLKQNNIKFKEIFVENS---TANLSKMIKRSK-KTTVPQIFINKV------HIGGYEDLIIFLKK--NI----------------------------------- |
| Q7PB08 | UniRef cluster | --------------------------------------------------MNKAILHTIIVYTLASCPYCIKAKALLDEKNVAYEEIEVSNFTQEEKEKFIKKSGG--KKTVPQIFIDNM------HVGGCDALFDLEKE--GRLDKLLENQPKRTSPAAGA----------------- |
| Q2NAA9 | UniRef cluster | -----------------------------------------------------MSEPKVDIYTKFGCGYCVRAKRLLDEK-GVDYEEFDITMGGPKRDEMRERAP--GAMTVPQIFIGDT------HVGGSDELHALERE--GKLDPLLAG---------------------------- |
| Q2C2C0 | UniRef cluster | ------------------------------------------MTEVKNLTIEQLEQYDNLVITQPNCPFCVKAKALLDERGIEYTTLVLGLNLSKLVMVDFIEQKM--GLTVRTVPQIILDG---KYIGGHDDLVAFLERQVNATEFDDFEL--------------------------- |
| B2AGP9 | UniRef cluster | -------------------------------------------------------MARVVMYSTVVCPYCVMAERLLKSRGVETIEKILIDREPGKREEMMSRT---GRRTVPQIYIDDT------HVGGFDDLSALDRQ--GGLVPLLAA---------------------------- |
| A4W535 | UniRef cluster | -------------------------------------------------------MANIEMYTKATCPFCHRAKALLSSK-GVTFQELPIDGDAAKREEMIKRSG---RTTVPQIFIDAQ------HIGGCDDLYALDAR--GGLEPLL------------------------------ |
| A0FVS8 | UniRef cluster | -------------------------------------------------------MSAITIYTTPTCPYCHAAKALLMNKGLS-YREVDVQNDRVTAVALMERT---GRRTVPQIFIGET------HVGGFDDLNALETA--GRLDRLLEANAPDHAAR-------------------- |
| Q3IIE2 | UniRef cluster | -------------------------------------------------------MSNVVLYTKAYCPFCQRARALLDSK-GVQYTNFDIGVQPELRDEMIAKAG--GASTVPQIFINDE------HIGGCDDMMAIEAQ--GQLDKKLNATLNA------------------------ |
| Q1LRT8 | UniRef cluster | -------------------------------------------------------MARVVMYSTVVCPYCQMAERLLKQRGVEAIEKILIDREPGKREEMMTRT---GRRTVPQIYIDET------HVGGFDDLSALDRQ--GGLVPLLAA---------------------------- |
| Q146B8 | UniRef cluster | -------------------------------------------------------MNKVIMYSTQVCPYCQMAERLLKSRGVEHVEKVLIDKDPARREEMMTRT---GRRTVPQVFIGET------HVGGYDDLSALDRA--GGLMPLLEATA-------------------------- |
| Q0KET6 | UniRef cluster | -------------------------------------------------------MARVVMYSTVVCPYCLMAERLLKSRGVETIEKVLIDREPGKREEMMART---GRRTVPQIYIDET------HVGGFDDLSALDRQ--GGLVPLLAA---------------------------- |
| A6VT71 | UniRef cluster | -------------------------------------------------------MATVTIYSSDYCPFCVRAKQLLTAK-GVAFNEIRVDGERELRQEMMEKSG---RHTVPQIWIGEQ------HVGGCDDLYALERE--QKLDALLA----------------------------- |
| A4Y075 | UniRef cluster | -------------------------------------------------------MADVVIYSSDWCPYCIRAKQLLASK-GVDYQEIRVDGQPAVRAEMTRKAG---RTSVPQIWIGST------HVGGCDDLYALERA--GKLDALLQSPA-------------------------- |
| A0GQK2 | UniRef cluster | -------------------------------------------------------MNKVIMYSTQVCPYCQMAERLLKSRGVEHVEKVLIDKDPARREEMMTRT---GRRTVPQVFIGET------HVGGYDDLSALDRA--GGLMPLLEATA-------------------------- |
| A0H7I5 | UniRef cluster | -------------------------------------------------------MQAVKMYTTAVCPYCIRAKQILQSKGVEQIEEVRIDFDTAAREHMMQTT---GRRTVPQIFIGDT------HVGGCDDLMALDAK--GGLLPLLQG---------------------------- |
| GLRX1 | UniRef cluster | --------------------------------------------------MNKAILHTIIIYTLASCPYCIKAKALLDEKNVVYEEIEVSNFTQEEKEKFIKKSGG--KKTVPQIFIDNI------HVGGCDALFDLEKE--GRLDKLLEGQPKKKMPAAGA----------------- |
| GLRX1 | UniRef cluster | --------------------------------------------------MNKAILHAIIIYTLAGCPYCMKAKALLDKKEVAYEEIEVQNSQDPNVAVLRKKLNNPDRLTFPQIFIDNM------HIGGCDDLYDLDKE--GRLDKLLEGQPKKD----------------------- |
| A8GV87 | UniRef cluster | --------------------------------------------------MNKAILHAIIIYTLAGCPYCMKAKALLDKKEVAYEEIEVQNSQDPNVAVLRKKLNNPDRLTFPQIFIDNM------HIGGCDDLYDLDKE--GRLDKLLEGQPKKD----------------------- |
| A8GMJ2 | UniRef cluster | --------------------------------------------------MNKAILHTIIIYTLVSCPYCIKAKALLDEQNIAYEEIEVSNLTQAEKEKFIKKSGG--KGTVPQIFIDNM------HVGGCDDLFDLEKE--GRLDKLLEGQPKKSI---------------------- |
| Q4JN11 | UniRef cluster | -------------------------------------------------------MKKITIYSTRICPYCVRAKNFFNKKNLE-YTEIMIDRDPEQMREMMEKS---GRQSVPQIFIGDY------HVGGFDDLIEYDMD--GKLEGLLG----------------------------- |
| Q4IVI0 | UniRef cluster | -------------------------------------------------------MPHIVIYTSAWCPYCIRAKQLLDRK-GVAYREIGVDGKPELRAEMTRKAG---RTSVPQIWIGDT------HVGGCDDLHALERA--GRLDALLED---------------------------- |
| A4T094 | UniRef cluster | -------------------------------------------------------MQQVTMYSTQVCPYCVMAEKLLQKKGVNNLEKILIDLDPAQREVMMTRT---GRRTVPQIYIGET------HVGGYDDLVALDRA--GKLDPLLM----------------------------- |
| A1URT4 | UniRef cluster | -------------------------------------------------------MKEVTLYTRPNCPYCTKARILLDKK-GIKYTDIDASTS--LRQEMVKRAN--GRNTFPQIFIGDY------HVGGYDDLHSLDAE--GKLDSLLNNAQ-------------------------- |
| Q2SMA5 | UniRef cluster | ----------------------------------------------------MAQFKPVTIYTTEFCPYCIRAKRLLEAK-GASFEEIKVDFNAALRQEMMQKSG---RRTVPQIWVGEE------HVGGCDELYGLERA--GTLDALLQSGASDESVSPS------------------ |
| Q1GS70 | UniRef cluster | -------------------------------------------------------MARIEVYTKAFCPYCTRAKRLLDGK-GADFSEIDVTMDRAGFDAMVARAG--GRRTVPQVFIDDR------HVGGSDELAALDAK--GELDALIGRA--------------------------- |
| B0PSV3 | UniRef cluster | -----------------------------------------------------MSAPRITVYTKQNCPYCVRAKRLLEKKGVAFEEISVEGKDE-LRTWLAEKTG---QLTVPQIFAGER------SLGGFSDLDALEQR--GELDPILRGG--------------------------- |
| A7MID5 | UniRef cluster | -------------------------------------------------------MANIEMYTKATCPYCHRAKALLNSK-GAAFLELPIDGDTAKREEMIQRSG---RTTVPQIFIDGQ------HIGGCDDLHALDAR--GGLDPLLR----------------------------- |
| Q9A9Y2 | UniRef cluster | -------------------------------------------------------MAKVTIYTRPFCPYCSRAVALLTDK-GADFTEIEAGMDPALRQEMMQRS---GRNTFPQIFVGEQ------HIGGCDDMMALEDQ--GKLDSLLSA---------------------------- |
| Q87UH2 | UniRef cluster | -------------------------------------------------------MAQVIVYSSDYCPYCIRAKQLLQSK-SVAFEEIRVDGKPQLRAEMTKKAG---RTSVPQIWIGPT------HVGGCDDLFALERA--GKLDALLA----------------------------- |
| Q10X15 | UniRef cluster | ------------------------------------------------------MSVNIEIYTWSSCPFCISAKALLDKK-QVNYQEYPIDGDDIEREKMACRAN--GRNSLPQIFIDEE------HIGGCDDLYGLEAQ--GKLDRLLKK---------------------------- |
| A9BUZ5 | UniRef cluster | -------------------------------------------------------MQAVKMYTTAVCPYCIRAKQILKSKGVEQIEEIRIDTDPAARDVMMQST---GRRTVPQIFIGDY------HVGGCDDLMALDAD--GGLVPLLQS---------------------------- |
| A7IJX6 | UniRef cluster | -------------------------------------------------------MKPIEIYTKSWCPYCHSAKELLRRK-GLTFTEIDVTTDKDGQEEMTRRAG--GRTSVPQIFIGET------HVGGCDDLYALEDA--GRLDQLAA----------------------------- |
| A1WBJ1 | UniRef cluster | -------------------------------------------------------MQAVKMYTTAVCPYCIRAKQLLKLKGVEQIEEIRVDTDPAARQHMMEIT---GRRTVPQIFIGDT------HVGGHDDLVALDSR--GGLVPLLGA---------------------------- |
| Q4FKX1 | UniRef cluster | -------------------------------------------------------MKNVTIYTGPLCNFCDAAKRLLARNNVEYKEINIATV---DGAMDEMITKANGKRTIPQIFFDDN------HIGGYDDVRALEKE--NKLLELLK----------------------------- |
| Q48C89 | UniRef cluster | -------------------------------------------------------MAQVIVYSSDYCPYCIRAKQLLQSK-SVAFEEICVDGKPQLRAEMTKKAG---RTSVPQIWIGST------HVGGCDDLFALERA--GKLDALLA----------------------------- |
| Q476J5 | UniRef cluster | -------------------------------------------------------MARVVMYSTQVCPYCVMAERLLKSKGVETIEKVLIDREPGKREEMMSRT---GRRTVPQIYIGDT------HVGGFDDLSALDRQ--GGLTPLLAA---------------------------- |
| Q1V095 | UniRef cluster | -------------------------------------------------------MKNVTIYTGPLCNFCDAAKRLLARNNVEYKEINIATV---DGAMDEMITKANGKRTIPQIFFDDN------HIGGYDDVRALEKE--NKLLELLK----------------------------- |
| Q0BBK7 | UniRef cluster | -------------------------------------------------------MSKVLMYSTQVCPYCIQAERLLKLRGVEQIEKVLIDRDPARRDEMMTRT---GRRTVPQIYIGET------HVGGYDDLSKLDRE--GGLLPLLQAA--------------------------- |
| A4AZF7 | UniRef cluster | -------------------------------------------------------MSKVELYTKGHCPYCHRAKALLTQK-GVEFIEYPIDVKPELRDEMIERAN--GGWTVPQIFIDDQ------HVGGCDDMMALEAQ--SKLSPMLGLA--------------------------- |
| A2W776 | UniRef cluster | -------------------------------------------------------MNKVLMYSTQVCPYCMQAERLLKLRGVEQIEKVLIDRDPARREEMMSRT---GRRTVPQIYIGDT------HVGGYDDLSKLDRE--GGLLPLLQAA--------------------------- |
| A0FQK7 | UniRef cluster | -------------------------------------------------------MNRVIMYSTQVCPYCQMAERLLKSRGVEHIEKVLIDKDPARRQEMMTRT---GRRTVPQVYIGET------HVGGYDDLSALDRA--GGLTPLLEAVA-------------------------- |
| Q5N151 | UniRef cluster | -------------------------------------------------------MANVEIYTWSACPFCVRAKALLTRK-GVAFQEYVIDGDEAARAVMAQRAN--GRRSVPQIFIDDQ------HIGGCDDLHALDRQ--GGLDPLLGLSA-------------------------- |
| Q491X8 | UniRef cluster | -------------------------------------------------------MAYIEIYTKKNCPYCERAKALLKKKSLDFREISVDCSNLSDSLSIEMRQRSGGRATFPQIFIDGL------HIGGSDDLILLNDQ--GKLDLILMNTNS------------------------- |
| Q31KR6 | UniRef cluster | -------------------------------------------------------MANVEIYTWSACPFCVRAKALLTRK-GVAFQEYVIDGDEAARAVMAQRAN--GRRSVPQIFIDDQ------HIGGCDDLHALDRQ--GGLDPLLGLSA-------------------------- |
| Q1BTB3 | UniRef cluster | -------------------------------------------------------MNKVLMYSTQVCPYCMQAERLLKLRGVEQIEKVLIDRDPARREEMMTRT---GRRTVPQIYIGDT------HVGGYDDLSKLDRE--GGLVPLLQAA--------------------------- |
| Q0AIA3 | UniRef cluster | ---------------------------------------------MDDNGAERDKMPKVVMYVSGFCPYCTMAEKLLRARGVEEIEKIRVDLQPGQRTEMMRRS---GRRTVPQIYIGQT------HVGGYDDLSMLDRQ--GELASLLAG---------------------------- |
| A9AEJ9 | UniRef cluster | ------------------------------------------------------------MYSTQVCPYCMQAERLLKLRGVEQIEKVLIDRDPARREEMMART---GRRTVPQIYIGET------HVGGYDDLSKLDRE--GGLVPLLQAA--------------------------- |
| A8HVB2 | UniRef cluster | -------------------------------------------------------MKSIVIYTKSWCSYCHAAKELLRRK-GWTFTEIDVTTDPAGQQEMSKKAG--GRTSVPQIFIGDT------HVGGCDDLYALEDA--GRLDALMAA---------------------------- |
| A4JI43 | UniRef cluster | -------------------------------------------------------MNKVLMYSTQVCPYCMQAERLLKLRGVEQIEKVLIDRDPARREEMMTRT---GRRTVPQIYIGDT------HVGGYDDLSKLDRE--GGLLPLLQAA--------------------------- |
| A2VV66 | UniRef cluster | -------------------------------------------------------MNKVLMYSTQVCPYCMQAERLLKLRGVEQIEKVLIDRDPARREEMMTRT---GRRTVPQIYIGDT------HVGGYDDLSKLDRE--GGLVPLLQAA--------------------------- |
| A0KAS8 | UniRef cluster | -------------------------------------------------------MNKVLMYSTQVCPYCMQAERLLKLRGVEQIEKVLIDRDPARREEMMTRT---GRRTVPQIYIGDT------HVGGYDDLSKLDRE--GGLVPLLQAA--------------------------- |
| Q82SU3 | UniRef cluster | -------------------------------------------------------MPKIVMYVSGYCPYCTMAEKLLRARGVEEIEKIRVDLQPGLRAEMMQRT---GRRTVPQIYIGPV------HVGGYDDLAMLDRQ--GELSGLLAG---------------------------- |
| Q2RWF3 | UniRef cluster | -------------------------------------------------------MVAVEIFTTPSCPYCRRAKALLGDK-GVAYREIDVSGDPRLREEMTRRAG--GRSTVPQIFIDGR------ALGGCDDIHALDRL--GKLDGLLSGSAP------------------------- |
| A6TW36 | UniRef cluster | -------------------------------------------------------MKKVVLYTKDFCPFCHRALDLLKSK-EVEFTNVDVTHDLETFKTVIKRTG---SDTVPQIFIDDE------FIGGCDDLIALDQE--DTLDSKLGL---------------------------- |
| A3YAN7 | UniRef cluster | -------------------------------------------------------MSQVTIYSSDYCPFCIRAKQLLTMK-QVEFNEINVDGQSALRREMTEKSG---RTSVPQIWIQEH------HVGGCDELFALERS--GKLDALLSQ---------------------------- |
| A1FTK5 | UniRef cluster | -----------------------------------------------MTAQTAGGAPAITIYSTAVCPYCVAAKNFLKSKGQQ-WTEVRIDLDPVEREKMMART---RRTSVPQIFVGDV------HVGGYDDMMALHRE--GKLEPLLAGQGQA------------------------ |
| Q5NPI3 | UniRef cluster | -------------------------------------------------------MAHIEIYTTRVCPYCKRAKALFAEK-GVSFDEYDVTDDSAKRTEMIKRSG--GR-TVPQIFIDDK------HIGGCDDLVKLNSE--GKLDPLLAK---------------------------- |
| Q39CN8 | UniRef cluster | ------------------------------------------------------------MYSTQVCPYCIQAERLLKLRGVEQIEKVLIDRDPARRDEMMTRT---GRRTVPQIYIGDT------HVGGYDDLSKLDRE--GGLVPLLQAA--------------------------- |
| Q15PS3 | UniRef cluster | -------------------------------------------------------MSKVEIYTKGHCPYCHRAKALLEQK-GVTYTEFKVDVQPELRPEMITRAN--GGSTVPQIFIGEH------HVGGCDEMFALESQ--NKLDTLLSA---------------------------- |
| A8ZZB5 | UniRef cluster | ------------------------------------------------------MIKAIEIYTTGTCPFCIRAKALLDRK-KVSYTEFRVDHTPAYVDEAVKRSG--GRTTVPQIFIGEV------HVGGCDELYALEKE--KKLDALLGL---------------------------- |
| A4VRV0 | UniRef cluster | -------------------------------------------------------MPNVVIYTTAWCPFCIRAKALLDRK-GVAYEEIPVDGNPTLRAEMASKAG---RTSVPQIWIGDK------HVGGCDELHALERA--GRLDPLLQA---------------------------- |
| A3TWM5 | UniRef cluster | -------------------------------------------------------MKPVEIYTTPICGFCSAAKRLLDGK-GVDYREIDVMRDREKKQEMMQRAG---RHTVPQIFIDGQ------HVGGCDELYELERA--GKLDPMLTA---------------------------- |
| A1VKR8 | UniRef cluster | -------------------------------------------------------MQTVKIYTTGSCPYCIQAKQLLKERGVTELNEIRVDMLPGERQKMMQIS---GRRTVPQIFIGAT------HVGGCDDLMALDGR--GGLMPLLSAAA-------------------------- |
| Q63XU5 | UniRef cluster | -------------------------------------------------------MNKVVMYSTQVCPYCMQAERLLKLRGVEHIEKVLIDKEPERRAEMMERT---GRRTVPQIYIGDT------HVGGYDDLSKLDRE--GGLKPLLEAA--------------------------- |
| Q62F45 | UniRef cluster | -------------------------------------------------------MNKVVMYSTQVCPYCMQAERLLKLRGVEHIEKVLIDKEPERRAEMMERT---GRRTVPQIYIGDT------HVGGYDDLSKLDRE--GGLKPLLEAA--------------------------- |
| Q3A7Z4 | UniRef cluster | -------------------------------------------------------MKKIELYTKSHCPYCRRAKDLLHIK-KAIFVEYDVTNDPAKEQEMRERS---GRMTVPEIFIDES------LVGGCDDLYALEQQ--GILDGMLRD---------------------------- |
| A9JZY3 | UniRef cluster | -------------------------------------------------------MNKVVMYSTQVCPYCMQAERLLKLRGVEHIEKVLIDKEPERRAEMMERT---GRRTVPQIYIGDT------HVGGYDDLSKLDRE--GGLKPLLEAA--------------------------- |
| A8KJC0 | UniRef cluster | -------------------------------------------------------MNKVVMYSTQVCPYCMQAERLLKLRGVEHIEKVLIDKEPERRAEMMERT---GRRTVPQIYIGDT------HVGGYDDLSKLDRE--GGLKPLLEAA--------------------------- |
| A7HTX3 | UniRef cluster | -------------------------------------------------------MADVTIYTTMMCPYCHRAKGLLQKK-GVSFTEVDVGMDADKRQEMMKRAH--GSHTVPQIFIGDK------HVGGCDDLYALDHA--GKLDPMLAA---------------------------- |
| A5XSB7 | UniRef cluster | -------------------------------------------------------MNKVVMYSTQVCPYCMQAERLLKLRGVEHIEKVLIDKEPERRAEMMERT---GRRTVPQIYIGDT------HVGGYDDLSKLDRE--GGLKPLLEAA--------------------------- |
| A5TIM2 | UniRef cluster | -------------------------------------------------------MNKVVMYSTQVCPYCMQAERLLKLRGVEHIEKVLIDKEPERRAEMMERT---GRRTVPQIYIGDT------HVGGYDDLSKLDRE--GGLKPLLEAA--------------------------- |
| A5J6D5 | UniRef cluster | -------------------------------------------------------MNKVVMYSTQVCPYCMQAERLLKLRGVEHIEKVLIDKEPERRAEMMERT---GRRTVPQIYIGDT------HVGGYDDLSKLDRE--GGLKPLLEAA--------------------------- |
| A4LJQ2 | UniRef cluster | -------------------------------------------------------MNKVVMYSTQVCPYCMQAERLLKLRGVEHIEKVLIDKEPERRAEMMERT---GRRTVPQIYIGDT------HVGGYDDLSKLDRE--GGLKPLLEAA--------------------------- |
| A3NR11 | UniRef cluster | -------------------------------------------------------MNKVVMYSTQVCPYCMQAERLLKLRGVEHIEKVLIDKEPERRAEMMERT---GRRTVPQIYIGDT------HVGGYDDLSKLDRE--GGLKPLLEAA--------------------------- |
| A3N5B2 | UniRef cluster | -------------------------------------------------------MNKVVMYSTQVCPYCMQAERLLKLRGVEHIEKVLIDKEPERRAEMMERT---GRRTVPQIYIGDT------HVGGYDDLSKLDRE--GGLKPLLEAA--------------------------- |
| A3MQ25 | UniRef cluster | -------------------------------------------------------MNKVVMYSTQVCPYCMQAERLLKLRGVEHIEKVLIDKEPERRAEMMERT---GRRTVPQIYIGDT------HVGGYDDLSKLDRE--GGLKPLLEAA--------------------------- |
| A2S627 | UniRef cluster | -------------------------------------------------------MNKVVMYSTQVCPYCMQAERLLKLRGVEHIEKVLIDKEPERRAEMMERT---GRRTVPQIYIGDT------HVGGYDDLSKLDRE--GGLKPLLEAA--------------------------- |
| A1UZX7 | UniRef cluster | -------------------------------------------------------MNKVVMYSTQVCPYCMQAERLLKLRGVEHIEKVLIDKEPERRAEMMERT---GRRTVPQIYIGDT------HVGGYDDLSKLDRE--GGLKPLLEAA--------------------------- |
| Q3JWH5 | UniRef cluster | ----------------------------------------------------------MVMYSTQVCPYCMQAERLLKLRGVEHIEKVLIDKEPERRAEMMERT---GRRTVPQIYIGDT------HVGGYDDLSKLDRE--GGLKPLLEAA--------------------------- |
| Q2T1H3 | UniRef cluster | ----------------------------------------------------------MVMYSTQVCPYCMQAERLLKLRGVEHVEKVLIDKEPERRAEMMERT---GRRTVPQIYIGDT------HVGGYDDLSKLDRE--GGLKPLLEAA--------------------------- |
| Q21YV8 | UniRef cluster | ----------------------------------------------------MSPMQTVKMYTTAVCPYCMQAKQILKAKGVAQIEEIRIDTDAQARAQMMEIT---GRRTVPQIFIGQT------HVGGCDDLIALDAR--DGLLPMLNAA--------------------------- |
| A5W3Y8 | UniRef cluster | -------------------------------------------------------MSPVTIYTTPHCPYCLSAKRLLSSKGI-TPDEINVEASP-LHLAEMMQRS--QRRTVPQIFVGKV------HVGGFDDLARLDSK--GQLEALLHA---------------------------- |
| A5GW94 | UniRef cluster | ------------------------------------------------------MAASVEIYTWRACPFCIRAKDLLSRK-GVSYTDHAIDGDEAARDQMATKTG--GRRSVPQVFINGQ------HVGGCDDLYALERS--GQLDGLLA----------------------------- |
| A1JHY3 | UniRef cluster | -------------------------------------------------------MAKIEIYTKATCPFCHRAKALLNSK-GAAFHEIAIDNDPAKREEMIARSG---RTTVPQVFIDGQ------HIGGCDDLHALDAR--GGLDPLL------------------------------ |
| Q6NC74 | UniRef cluster | ------------------------------------------------------MPAAIEIFTRPGCGYCSAAKSLLNRK-KAAFTEYDVAVDPGFRVKMDERAG--PGATYPQIFIGDL------HVGGCDDLYALDRE--GKLDALLADEKAAS----------------------- |
| Q2W6W5 | UniRef cluster | -------------------------------------------------------MAEIEIYTTDVCPYCVKAKKLFAKK-GVAYTEINVSTDDGLRQYMTNRAG--GRRSVPQIFIDGV------HVGGCDDLYALDKD--GKLDPMLAGVQ-------------------------- |
| B0IKE5 | UniRef cluster | ------------------------------------------------------MPAAIEIFTRPGCGYCSAAKSLLNRK-KAAFTEYDVAVDPGFRVKMDERAG--PGATYPQIFIGDL------HVGGCDDLYALDRE--GKLDALLADEKAAS----------------------- |
| A8ZM91 | UniRef cluster | -------------------------------------------------------MANIEIYTWSTCPFCRRAKQLLDQK-GATYTEYQIDGDEAARDQMVARGTN-GRRSVPQIFINDQ------HIGGSDDLYALERQ--GALDKLLGSTEVLV----------------------- |
| A4YKU2 | UniRef cluster | ------------------------------------------------------MTAAIEIYTRPGCGYCTAAKSLLTRK-NVPFTEYDAGKDPNVRQQMYDRVG--PGSTFPQIFIGKT------HIGGCDDLYALDRE--GRLDAMLAGDKATS----------------------- |
| A4G996 | UniRef cluster | ------------------------------------------------------MTAHVVMYSSGVCPYCTMAERLLTAKGISNIEKIRIDLDPAQRAAMMQKT---GRRTVPQIYVGDT------HVGGFDDLNALERL--GKLDALLQGA--------------------------- |
| Q8DKF2 | UniRef cluster | -------------------------------------------------------MAKVEIYTWSRCPFCIRAKQLLTQK-GVKFTEYVIDGDEVARDAMAKRAH--GRRSLPQIFIDNE------HIGGCDDLYALEAQ--GKLDALLQGVA-------------------------- |
| A8YH34 | UniRef cluster | ------------------------------------------------------MAANVEIYTWSSCPFCIRAKALLKKK-GVEFTEYCIDGDEGARAKMSDRAN--GRTSVPQIFINDQ------HIGGCDDIYALERS--GGLAPLLQN---------------------------- |
| A0L7H0 | UniRef cluster | -------------------------------------------------------MADITIYSTTICPFCVRAKQLFKKK-GVDFTEINLDKQPDRRDEMLAKSG--GRRTVPQIFIGDR------HVGGCDDLYELELD--GELDPLLGL---------------------------- |
| GLRX2 | UniRef cluster | ----------------------------------------------------MAVSAKIEIYTWSTCPFCMRALALLKRK-GVEFQEYCIDGDNEAREAMAARAN--GKRSLPQIFIDDQ------HIGGCDDIYALDGA--GKLDPLLHS---------------------------- |
| Q7CLB8 | UniRef cluster | -------------------------------------------------------MAKIEMYTKATCPFCHRAKALLNAK-GAAFHEIAIDNDPAKREEMIARSG---RTTVPQIFIDGQ------HIGGCDDLHALDAR--GGLDPLL------------------------------ |
| Q66GC0 | UniRef cluster | -------------------------------------------------------MAKIEMYTKATCPFCHRAKALLNAK-GAAFHEIAIDNDPAKREEMIARSG---RTTVPQIFIDGQ------HIGGCDDLHALDAR--GGLDPLL------------------------------ |
| Q2JWV1 | UniRef cluster | --------------------------------------------------------MAVEIYTWRFCPFCIRAKQLLDRK-GVKYIEYAIDGDEAARAEMAKRAN--GRRSVPQIFINNQ------HIGGCDDLYALEAQ--GKLDLLLQQAS-------------------------- |
| Q2JPZ5 | UniRef cluster | --------------------------------------------------------MTVEIYTWRFCPFCIRAKQLLDRK-GVKYIEYAIDGDEAARAEMAKRAN--GRRSVPQIFINDQ------HIGGCDDLYALEAR--GQLDLLLQQAS-------------------------- |
| Q13EZ3 | UniRef cluster | ------------------------------------------------------MSAAIEIFTRPGCGYCGAAKSLLNRK-KAAFTEYDVSVDPGFRVKMDERAG--PGATYPQIFIGSF------HVGGCDDLYALDRE--GKLDALLAGELAAGEKAIS------------------ |
| Q8KRM3 | UniRef cluster | -------------------------------------------------------MANIDIYTKATCPFCHRAKALLNSK-GAAFNEIAIDGDNAKREVMIERSG---RTTVPQIFIDGR------HIGGCDDLYELDAR--GGLDPLL------------------------------ |
| Q1CD19 | UniRef cluster | -------------------------------------------------------MAKIEMYTKATCPFCHRAKALLNAK-GAAFHEIAIDNDPAKREEMIARSG---RTTVPQIFIDGQ------HIGGCDDLHALDAR--GGLDPLL------------------------------ |
| Q1C284 | UniRef cluster | -------------------------------------------------------MAKIEMYTKATCPFCHRAKALLNAK-GAAFHEIAIDNDPAKREEMIARSG---RTTVPQIFIDGQ------HIGGCDDLHALDAR--GGLDPLL------------------------------ |
| Q0I6S6 | UniRef cluster | -------------------------------------------------------MAKVEIYTWSTCPFCVRAKGLLDRK-GVAYSEVSVDGDEPGRDAMAARGN--GKRSVPQIFINDQ------HVGGCDELHGLERA--GKLDGLLAGQA-------------------------- |
| Q0BXE9 | UniRef cluster | -------------------------------------------------------MAKVTIYTRAFCPYCTRALQVLKDK-GVELNEIDAGMDPALREEMVQRSN--GGRTFPQIFVGET------HIGGCDDMLALDRS--GKLDPMLASV--------------------------- |
| B0ULR2 | UniRef cluster | -------------------------------------------------------MQPVTIYTTSWCPYCAAAKSLLREK-GAAFTEIDVEARAGARREMIDRAG--GRTSVPQIFVGAT------HVGGCDDLYALDRA--GRLEPLLAG---------------------------- |
| B0HYY2 | UniRef cluster | -------------------------------------------------------MAKIEMYTKATCPFCHRAKALLNAK-GAAFHEIAIDNDPAKREEMIARSG---RTTVPQIFIDGQ------HIGGCDDLHALDAR--GGLDPLL------------------------------ |
| B0HLD0 | UniRef cluster | -------------------------------------------------------MAKIEMYTKATCPFCHRAKALLNAK-GAAFHEIAIDNDPAKREEMIARSG---RTTVPQIFIDGQ------HIGGCDDLHALDAR--GGLDPLL------------------------------ |
| B0HAG0 | UniRef cluster | -------------------------------------------------------MAKIEMYTKATCPFCHRAKALLNAK-GAAFHEIAIDNDPAKREEMIARSG---RTTVPQIFIDGQ------HIGGCDDLHALDAR--GGLDPLL------------------------------ |
| B0GPJ9 | UniRef cluster | -------------------------------------------------------MAKIEMYTKATCPFCHRAKALLNAK-GAAFHEIAIDNDPAKREEMIARSG---RTTVPQIFIDGQ------HIGGCDDLHALDAR--GGLDPLL------------------------------ |
| B0GDS6 | UniRef cluster | -------------------------------------------------------MAKIEMYTKATCPFCHRAKALLNAK-GAAFHEIAIDNDPAKREEMIARSG---RTTVPQIFIDGQ------HIGGCDDLHALDAR--GGLDPLL------------------------------ |
| A9ZYX6 | UniRef cluster | -------------------------------------------------------MAKIEMYTKATCPFCHRAKALLNAK-GAAFHEIAIDNDPAKREEMIARSG---RTTVPQIFIDGQ------HIGGCDDLHALDAR--GGLDPLL------------------------------ |
| A9Z461 | UniRef cluster | -------------------------------------------------------MAKIEMYTKATCPFCHRAKALLNAK-GAAFHEIAIDNDPAKREEMIARSG---RTTVPQIFIDGQ------HIGGCDDLHALDAR--GGLDPLL------------------------------ |
| A9R689 | UniRef cluster | -------------------------------------------------------MAKIEMYTKATCPFCHRAKALLNAK-GAAFHEIAIDNDPAKREEMIARSG---RTTVPQIFIDGQ------HIGGCDDLHALDAR--GGLDPLL------------------------------ |
| A9BD40 | UniRef cluster | -------------------------------------------------------MAQVEIYTWQFCPFCIRAKNLLNKK-GVDYKEYPIDGDPEGRLKMSERAG--GRTTVPQIFINEK------GIGGCDELYALEDS--HQLDSLLN----------------------------- |
| A8GLC0 | UniRef cluster | -------------------------------------------------------MANIDIYTKATCPFCHRAKALLNSK-GAAFNEIAIDGDNEKREAMIARSG---RTTVPQIFIDGQ------HIGGCDDLYELDAR--GGLDPLL------------------------------ |
| A7FCV0 | UniRef cluster | -------------------------------------------------------MAKIEMYTKATCPFCHRAKALLNAK-GAAFHEIAIDNDPAKREEMIARSG---RTTVPQIFIDGQ------HIGGCDDLHALDAR--GGLDPLL------------------------------ |
| A6T313 | UniRef cluster | ------------------------------------------------------MTAHVVMYSTGVCPYCTMAERLLTAKGIANIEKIRIDLDPAQRVAMMEKT---GRRTVPQIYVGDT------HVGGFDDLNALERQ--GKLDALLQSA--------------------------- |
| A6BW64 | UniRef cluster | -------------------------------------------------------MAKIEMYTKATCPFCHRAKALLNAK-GAAFHEIAIDNDPAKREEMIARSG---RTTVPQIFIDGQ------HIGGCDDLHALDAR--GGLDPLL------------------------------ |
| A4TSC0 | UniRef cluster | -------------------------------------------------------MAKIEMYTKATCPFCHRAKALLNAK-GAAFHEIAIDNDPAKREEMIARSG---RTTVPQIFIDGQ------HIGGCDDLHALDAR--GGLDPLL------------------------------ |
| A2BNX4 | UniRef cluster | -------------------------------------------------------MSKVEIYTWQYCPFCIRAKSLLKKK-NVNFTEYKIDGDEDARALMIERAD--GRRTLPQIFIDNE------GIGGCDDLYALENE--NKLEALLN----------------------------- |
| Q9PAC3 | UniRef cluster | --------------------------MRGIGYHWTSKSTRSFLMKQDATRDEAGSTPKITIYSTAICPYCVAAKNFLKSKGYT-WTEIMIDVNAAEREKMIART---QRTSVPQIFVGDT------HVGGYDDMMALHHA--GKLEPLLTNHSPQG----------------------- |
| Q88IP1 | UniRef cluster | -------------------------------------------------------MSPVTIYTTPHCPYCLSAKRLLSSKGI-TPDEINVETSP-LHLAEMMQRS--QRRTVPQIFVGKV------HVGGFDDLARLDRK--GQLEALLHA---------------------------- |
| Q6LSR2 | UniRef cluster | ---------------------------------------------MSTMTIEQLSEYKNLVITQPHCPFCVKAKALLDDRDTEYTTLVLGTDLEKTEMVAFIEQVA--NTTVRTVPQIMLDG---KFIGGHDDLVAFLERQVNTEELGDFEL--------------------------- |
| Q31E79 | UniRef cluster | -----------------------------------------------------------------------MAKSLLDGK-GLKYDVIDVGTDRSLWQELQEKTG---RNTVPQVFVGDH------HIGGFDDLSAADRS--GELDQILAK---------------------------- |
| B0JQ91 | UniRef cluster | ------------------------------------------------------MAANVEIYTWSSCPFCIRAKALLKKK-GVEFTEYCIDGDERARAKMSDRAN--GRTSVPQIFINDQ------HIGGCDDIYALDRS--GGLAPLLQN---------------------------- |
| A9H3J1 | UniRef cluster | -------------------------------------------------------MQPVTIYTTSWCPYCTAAKSLLREK-GAAFTEIDIEVKAGARREMIGKAG--GRTSVPQIFIGST------HVGGCDDLYALDRA--GRLDPLLAG---------------------------- |
| A8E918 | UniRef cluster | ------------------------------------------------------------MYSTQVCPYCMQAERLLKLRGVEHIEKVLIDKEPERRAEMMERT---GRRTVPQIYIGDT------HVGGYDDLSKLDRE--GGLKPLLEAA--------------------------- |
| GLRX | UniRef cluster | -------------------------------------------------------MQTVTMYTGPFCPYCAMAKRLLHAAGVGHIDEIRVDASP-EAFAEMQQLS--GQRSVPQIFIGET------HVGGFTDLYRLQQE--GGLDGLLNP---------------------------- |
| GLRX | UniRef cluster | -------------------------------------------------------MQTVTMYTGPFCPYCTMAKRLLHAAGVGHIDEIRVDASP-EAFAEMQQLS--GQRSVPQIFIGET------HVGGFTDLYRLQQE--GGLDGLLNP---------------------------- |
| Q87A59 | UniRef cluster | -------------------------------------------MKQDSTRDEAGSTPKITIYSTAICPYCVAAKNFLKSKGYT-WTEIMINVNSAEREKMIART---QRTSVPQIFVGDT------HVGGYDDMMALHHA--GKLEPLLTNHSPQG----------------------- |
| Q7U3W7 | UniRef cluster | -------------------------------------------------------MAKVEIYTWRTCPFCVRAKGLLDRK-GVSYTEHAVDGDEPGRDAMAARGD--GRRSVPQIFIDDR------HIGGCDDLHALERS--GELDPLLNA---------------------------- |
| Q3R565 | UniRef cluster | -------------------------------------------MTQDSTRDEAGSTPKITIYSTAICPYCVAAKNFLKSKGYT-WTEIMINVNSAEREKMIART---QRTSVPQIFVGDT------HVGGYDDMMALHHA--GKLEPLLTNHSPQG----------------------- |
| B0SW74 | UniRef cluster | -------------------------------------------------------MAKVTIYTRPFCGYCARALKLLNDK-GADFTEVEAGMDPALRKEMMDRS---GRATFPQIFVGEQ------HIGGCDDMMALERA--GKLDALLAA---------------------------- |
| A9M1W6 | UniRef cluster | -----------------------------------------------MQNFRKPNMQTVTMYTGPFCPYCAMAKRLLHAAGVGHIDEIRVDASP-EAFAEMQQLS--GQRSVPQIFIGET------HVGGFTDLYRLQQE--GGLDGLLNP---------------------------- |
| A7W6D9 | UniRef cluster | -------------------------------------------------------MQPVTIYTTAWCPYCSAAKSLLREK-GVSFHEIDVEKTAGSRATMVQRAG--GRTSVPQIFVGDR------HVGGCDDLYALERA--GDLDPLLAA---------------------------- |
| A3VD51 | UniRef cluster | -------------------------------------------------------MQPVTIYTTPFCGFCHAAKRLLTSK-GVAFDEIDVSVDPALRQDMMAKAG---RHTVPQIWVGET------HVGGFDDLNALERS--GKLDPLLAA---------------------------- |
| A3IMZ9 | UniRef cluster | ---------------------------------------MFNIFNSLLGRHPEKMKADVEIYTWQTCPFCIRAKLLLWWK-GVNFTDYKIDGDETAREKMAQRSQ--GKRTVPQIFINNQ------HIGGCDDLYSLDQQ--NKLDSLLTKEN-------------------------- |
| A1KSA6 | UniRef cluster | -----------------------------------------------MQNFRKPNMQTVTMYTGPFCPYCAMAKRLLHAAGVGHIDEIRVDASP-EAFAEMQQLS--GQRSVPQIFIGET------HVGGFTDLYRLQQE--GGLDGLLNP---------------------------- |
| Q47IG2 | UniRef cluster | ------------------------------------------------------MTAPVLMYTTAVCPYCIRAKQLLAARGVTQIEEVRVDLDPERRDEMMLKT---KRRTVPQIFIGDT------HVGGCDDLYALDAA--GQLKPLLEG---------------------------- |
| Q1Z0T0 | UniRef cluster | ---------------------------------------------MSTMTIEQLSEYKNLVITQPHCPFCVKAKALLDDRGTEYTTLVLGTDLEKTEMVAFIEQVA--NTTVRTVPQIMLDG---KFIGGHDDLVAFLERQVNTEELGDFEL--------------------------- |
| Q1GZ84 | UniRef cluster | -------------------------------------------------------MANVLMYTTGTCPYCMSAERLLLSKGVQQINKVRVDLEPELRVKMMEQT---GRRTVPQIYIDDV------HVGGFDDLRALDMA--GKLDAMLAK---------------------------- |
| A9W702 | UniRef cluster | -------------------------------------------------------MQPVTIYTTAWCPYCSAAKSLLREK-GVSFHEIDVEKTAGSRAAMVQRAG--GRTSVPQIFVGDR------HVGGCDDLYALERA--GDLDPLLAA---------------------------- |
| A5ETE8 | UniRef cluster | ------------------------------------------------------MTAAIEIYTRPGCGYCTAAKSLLTRK-NVPFTEHDAGKDPTIRQKMYDRVG--PGSTFPQIFIGTT------HVGGCDDLYALDRE--GKLDAMLAGETTS------------------------ |
| Q5FUT1 | UniRef cluster | -------------------------------------------------------MSKVEIYTQPGCPYCVHAVSLLRSK-GIEFQEINAPHGSAEREESIRRSG---RRTVPQTFVDGK------GLGGCDDLMALDRA--GRLDALLGKV--------------------------- |
| Q31D02 | UniRef cluster | -------------------------------------------------------MSKVEIYTWQYCPFCIRAKSLLKKK-NVNFTEYKIDGDEDARELMIERAD--GRRTLPQIFIDNE------GIGGCDDLYALENE--NKLEALLN----------------------------- |
| Q2K3S5 | UniRef cluster | -----------------------------------MQGLSPSLYDRNTRHPSGDIMVPVTIYTRQFCGYCSRAKSLLEEK-GVDYVEHDATYSADLRQEMIGKSN--GRTTFPQIFIGTE------HVGGCDDLFALDRA--GKLDPMLAA---------------------------- |
| Q3RG42 | UniRef cluster | -------------------------------------------MKQDSTRDEAGSTPKITIYSTAICPYCVAAKNFLKSKGYT-WTEIMINVNSAEREKMIALT---QRTSVPQIFVGDT------HVGGYDDMMALHHA--GKLEPLLTNHSPQG----------------------- |
| Q3R688 | UniRef cluster | -------------------------------------------MKQDSTRDEAGSTPKITIYSTAICPYCVAAKNFLKSKGYT-WTEIMINVNSAEREKMIALT---QRTSVPQIFVGDT------HVGGYDDMMALHHA--GKLEPLLTNHSPQG----------------------- |
| Q2BHX2 | UniRef cluster | -------------------------------------------------------MQDVTIYTTAWCPFCIRAKMLLDHK-QIPYSEIKVDGDPAKRQEMTKLSG---GHTVPQIFIGET------PIGGCDDMFALERQ--GKLDEMLNAS--------------------------- |
| Q11DZ8 | UniRef cluster | -------------------------------------------------------MADVTIYTRMGCGYCVAAKRLLERK-GIAYTEQDASFSPELRKEMIGRAN--GRSTFPQIFIGNI------HVGGSDDLHALERE--GRLDALLEERDIVGSSR-------------------- |
| Q10XD7 | UniRef cluster | ---------------------------------------MLDFLNPILGRHPERMKAKVEIYTWQTCPYCIRAKLLLWWK-GVNYTEYKIDGNESARNSMSERAN--RSRTVPQIFINNQ------HIGGCDDIYALDNK--GQLEPLLIEVLPD------------------------ |
| B0U5K0 | UniRef cluster | -------------------------------------------MKQDSTRDEAGSTPKITIYSTAICPYCVAAKNFLKSKGYT-WTEIMINVNSAEREKMIALT---QRTSVPQIFVGDT------HVGGYDDMMALHHA--GKLEPLLTNHSPQG----------------------- |
| A8CY93 | UniRef cluster | ------------------------------------------------MSDTASPTPAIVIYTTAICPYCVAAKNFLKSKGQS-WSEVRIDLDPAEREKMVART---KRTSVPQIFIGDT------HVGGYDDMMALHRA--GGLEPLLGGGA-------------------------- |
| A3UGI4 | UniRef cluster | -------------------------------------------------------MAAVTIYTRPMCPYCVRAVSLLKKK-GVSFDEIDAGFDVKKKKEMVDRAN--GARTFPQIFIGDT------HVGGCDEMMAMENA--GKLDDLLRNEGAL------------------------ |
| Q21NG9 | UniRef cluster | -------------------------------------------------------MPEVVIYSSNYCGFCFRAKQLLQSK-GVKYKEIVVDGDTQLRTELARKAG---ARTVPQIWIGDN------HVGGCDELYTLERT--GSLDDLLAL---------------------------- |
| Q1YUL6 | UniRef cluster | -------------------------------------------------------MSHVVLYGTRFCPFCTAARRLLTAK-EIDYQDISVDNNPELRGKLITKSG---RNTVPQIWFGNQ------HIGGFDELRDLERQ--GTFNATLKAGVESGETVSI------------------ |
| B0J731 | UniRef cluster | -------------------------------------------------------MVRVTIYTRQFCGYCTRAKSLLEEK-GVEYVEHDATFSPDLRQEMIGKSN--GRTTFPQIFIGAD------HVGGCDDLFALDRA--GKLDPLLAA---------------------------- |
| A4U2A5 | UniRef cluster | -------------------------------------------------------MAQVEIYTTQTCPYCIRAKRLLTTK-GVAFQEYDVSNDPELRSAMTARAH--GGRTVPQIFINGE------HVGGCDDLHSLDGA--GELDVLLAKESA------------------------- |
| Q8PNL1 | UniRef cluster | -----------------------MEIPVARYHVSDLTPTTEFPVSQDHTDQDAASGPQITLYSSAICPYCVAAKNFLKSKGKT-WTEVRIDLDPAERDKMVALA---KRTSVPQIFVGDV------HVGGYDDMMAMHRA--GKLEPLLAGSAGGQA---------------------- |
| Q7VE11 | UniRef cluster | -------------------------------------------------------MAKVEIYTWQYCPFCIRAKALLDLK-KIDYDEYPIDGNQAEREKMSIRAK--GKTTVPQIFINNQ------SVGGCDELYALEES--NQLDNLINQNK-------------------------- |
| Q5GWK3 | UniRef cluster | -----------------------MEIPVARYHVSDPIPTTEFPVSQDHTDQGAASGPQITLYSSAICPYCVAAKNFLKSKGKT-WTEVRIDLDPAERDKMVALA---KRTSVPQIFVGDV------HVGGYDDMMAMHRA--GKLEQLLAGSAGGQA---------------------- |
| Q5FAB3 | UniRef cluster | -------------------------------------------------------MQTVTMYTGPFCPYCAMAKRLLHAAGVGHIDEIRVDASP-EAFAEMQRLS--GQRSVPQIFIGET------HVGGFTDLYRLQQE--GGLDGLLNP---------------------------- |
| Q3JF21 | UniRef cluster | -------------------------------------------------------MPKVVMYATLWCPYCIGARRLLDSK-GIDYTEIRVDLEPEQRAVMINKSH---RRTVPQIFINDQ------PIGGYDELAQLERA--RELDVLLGLA--------------------------- |
| Q3AGV9 | UniRef cluster | -------------------------------------------------------MAKVEIYTWRTCPFCVRAKGLLDRK-GVSYTEHSVDGDEPARDAMAARGD--GRRSVPQVYIDDR------HIGGCDDLHALDRG--GELDALLKA---------------------------- |
| Q2NZR1 | UniRef cluster | -----------------------MEIPVARYHVSDPIPTTEFPVSQDHTDQGAASGPQITLYSSAICPYCVAAKNFLKSKGKT-WTEVRIDLDPAERDKMVALA---KRTSVPQIFVGDV------HVGGYDDMMAMHRA--GKLEQLLAGSAGGQA---------------------- |
| Q6SFQ8 | UniRef cluster | ---------------------------------------------------------MIKVYTSHSCFYCTRAKNYLDNLDIDYQTLN-----IQEDVEARDFFIKSGFRTVPQIFVDDQ----LLCKGGSDGLVQMTKEDIQNKVIELTGVRS------------------------- |
| Q28VP9 | UniRef cluster | -------------------------------------------------------MAIVTLYTSPLCGFCHAAKRMLTDK-GVSYAEIDVAADPSKRQEMMSRAN--GRHTVPQIFIGDA------HVGGYDDMAALERT--GKLDPMLSAD--------------------------- |
| A3K2U1 | UniRef cluster | -------------------------------------------------------MQNVEIYSSPLCGYCHAAKRLLTSK-GVAFHEINVLEEPARRSEMMDRAH--GRHTVPQIFVGDT------HVGGYDDLAALERE--GKLDPLLAG---------------------------- |
| A3IP05 | UniRef cluster | ------------------------------------------------------MAANVEIYTWSTCPFCIRAKALLVKK-GAEFTEYCIDGDEEAREEMTERAN--GKRSLPQIFINDR------HVGGCDELYHLDST--GKLDSLLEQSAA------------------------- |
| A2BUF6 | UniRef cluster | -------------------------------------------------------MSTVEIYTWRFCPFCIRAKSLLEKK-NITFTEHKIDGDDDARELMTKRAN--GKRTVPQIFINNK------SIGGCDELYELEKE--EKLDLLLN----------------------------- |
| A1K9C2 | UniRef cluster | -----------------------------------------------------MSTSSIRMYATAVCPYCVRAEQLLRRKGVTAIDKIRIDLDPARRDEMMELT---GRRTVPQIFIGDL------HVGGCDDLYELDRS--GGLDPLLAKLAG------------------------- |
| Q8PBZ1 | UniRef cluster | --MRAARRVGVNRQLIHSVAVTTSKFQSPVTICQTTDPTTEFPVSQDHTGQQAATGPQITLYSTAICPYCVAAKNFLKSKGQT-WTEVRIDLDPAERDKMVALA---KRTSVPQIFVGDI------HVGGYDDMMAMHRA--GKLEPLFAAAGGQA----------------------- |
| Q7VRK6 | UniRef cluster | ------------------------------------------------------MSYDIVIYTKKNCPYCDRAKFFLVEQSLIFKEIVLSDD-KSDPAYLEMYNRSKGCVTVPQVFINNN------HVGGSDDLLKLGRN--RKDLEQLLK---------------------------- |
| Q4URK9 | UniRef cluster | --MRAARRVGVNRQLIHSVAVTTSKFQSPVTICQTTDPTTEFPVSQDHTGQQAATGPQITLYSTAICPYCVAAKNFLKSKGQT-WTEVRIDLDPAERDKMVALA---KRTSVPQIFVGDI------HVGGYDDMMAMHRA--GKLEPLFAAAGGQA----------------------- |
| Q3BWQ8 | UniRef cluster | --------------------------------MSDLTPTTEFPVSQDHTDQDAASGPQITLYSSAICPYCVAAKNFLKSKGKT-WTEVRIDLDPAERDRMIALA---KRTSVPQIFVGDV------HVGGYDDMMAMHRA--GKLEPLLAGSAGGQA---------------------- |
| B0RU09 | UniRef cluster | --MRAARRVGVNRQLIHSVAVTTSKFQSPVTICQTTDPTTEFPVSQDHTGQQAATGPQITLYSTAICPYCVAAKNFLKSKGQT-WTEVRIDLDPAERDKMVALA---KRTSVPQIFVGDI------HVGGYDDMMAMHRA--GKLEPLFAAAGGQA----------------------- |
| Q2J473 | UniRef cluster | ------------------------------------------------------MSAAIEIFTRPGCGYCSAAKSLLNRK-KAAFTEYDVAVDPAHRKTMDARTY--PGSTYPQIFIGGT------HVGGCDDLYALDRE--GKLDALLAGEKATS----------------------- |
| Q1MBA6 | UniRef cluster | -------------------------------------------------------MVPVTIYTRQFCGYCTRAKSLLEEK-GVEYVEHDATFSPDLRQEMIGKSN--GRTTFPQIFIGAD------HVGGCDDLFALDRA--GKLDPMLAA---------------------------- |
| Q12F48 | UniRef cluster | -------------------------------------------------------MQPVKIYTTATCPYCIHAKQLLKQRGVAELDEIRVDMLPGERQKMMDIT---GRRTVPQIFIGGT------HVGGCDDLVALDGR--GGLMSLLNGG--------------------------- |
| Q0G7Z1 | UniRef cluster | -------------------------------------------------------MPDITIYTRQLCGFCARAKRLLDEK-GVVYDEKDATGSPVLRQEMRERAK--GGATFPQIFIGET------HVGGCDDLFALDRA--GKLDQLLSA---------------------------- |
| A5GP83 | UniRef cluster | -------------------------------------------------------MPSVEIYTWRTCPFCVRAKQLLDRK-GVTYTEYSVDGDEPARDAMAARGN--GRRSVPQIFIADQ------HIGGCDELHALERA--GKLDALLS----------------------------- |
| Q8YMR0 | UniRef cluster | ---------------------------------------MSNLFNQLFGRSPAKIKANVEIYTWQTCPYCIRAKLLLWWK-GVQFTEYKIDGDEAARANMAERAN--GRRTVPQIFINNQ------HIGGCDDLYELDTK--GQLDPLLVQPA-------------------------- |
| Q89XU6 | UniRef cluster | ------------------------------------------------------MPAAVEIYTRPGCGYCSAAKSLLTRK-KATFTEFDVARNPSWRDEMYDRAG--EGSTFPQIWIGGS------HVGGCDDLYALDRE--GKLDGMLESVKAES----------------------- |
| Q3MB73 | UniRef cluster | ---------------------------------------MLNLFNQFFGRSPEKIKANVEIYTWQTCPYCIRAKLLLWWK-GVQFTEYKIDGDEAARANMAERAN--GRRTVPQIFINNQ------HIGGCDDLYELDTK--GQLDPLLVQPA-------------------------- |
| A4CY94 | UniRef cluster | -------------------------------------------------------MPSVEIYTWRTCPFCIRAKQLLDRK-GVAYTEYSVDGDEPARDAMAARGN--GRRSVPQIFIADQ------HIGGCDELHALERA--GTLDALLS----------------------------- |
| Q7NF43 | UniRef cluster | ------------------------------------------------------MNPKVEIYTWQFCPFCIRAKALLKQK-SVAFSEYAIDGDEAARSAMAERAD--GRRSVPQIFIDGK------HIGGCDDLYALDRS--GQLDPLLVAS--------------------------- |
| Q5P7N2 | UniRef cluster | -------------------------------------------------------MAKIRMYATNVCPYCVRAEQLLKRKGVTDIEKIRVDQDPSLRDEMIRLT---GRRTVPQIFIGDL------HVGGCDDLFDLDHA--GKLDSLLAAP--------------------------- |
| Q21BZ2 | UniRef cluster | --------------------------------MMSMVRKPESLFGIMLQQPECDMSAAIEIFTRPGCGYCSAARSLLTRK-KAAFTEYDVSLDPSFREQMTKRVG--AGATYPQIFIGEL------HVGGCDDLYDLDRA--GKLDSLLAGETAAS----------------------- |
| A8G2I5 | UniRef cluster | -------------------------------------------------------MSKVEIYTWQYCPFCIRAKSLLKKK-NINFTEYKIDGDEDARALMTERAD--GRGTLPQIFIDNE------GIGGCDDLYTLENE--NKLDSLLG----------------------------- |
| A4EH01 | UniRef cluster | -----------------------------------------------------------------------MAKRLLNSK-DISYAEVNISAQPERRAEMIQRAN--GGSTVPQIFVDGT------HVGGCDDLFALERG--GKLDALLAA---------------------------- |
| A3Z381 | UniRef cluster | -------------------------------------------------------MAAVEIYTWRFCPFCVRAKQLLDRK-GVAYTEYAIDGDEPARDAMAARGD--GRRSVPQIFIADR------HIGGCDDLHAMERA--GELDALLAGAA-------------------------- |
| A3VV00 | UniRef cluster | ----------------------------------------------------MSDMQSVIMYTTPMCPYCARARRLLEEK-GATIEEKRAGLNVDLKNEMIEKSG--GARTFPQIFIGDT------HVGGCDDLMALNEE--GKLDRQLAGDR-------------------------- |
| Q7V4A1 | UniRef cluster | -------------------------------------------------------MAKVEIYTWQSCPFCLRAKALLDRK-GVSYQEHAIDGDQAARAVMASRAG--GKNTLPQIFIDDL------SIGGCDELHALEGA--QKLDGLLQGKV-------------------------- |
| B0BYI1 | UniRef cluster | ---------------------------------------MPNFLNNLLGRHPERVKANTEIYTWQTCPFCIRAKLLLSWK-GVKYTEYKIDGDEAARAKMAERAN--NRRSVPQIFINNQ------HIGGCDDLYQMDTM--GQLDDLLSQPAAS------------------------ |
| A3PAP7 | UniRef cluster | -------------------------------------------------------MSKVEIYTWQYCPFCIRAKSLLKKK-NIIFTEYKIDGDEDARALMTERAD--GRRTLPQIFIDNE------GIGGCDDLYTLENE--NKLDALLN----------------------------- |
| Q3IYK8 | UniRef cluster | -------------------------------------------------------MKSVEIYTTPTCGYCQAAKSLLRRK-GVSYAETDVSTDPSLRAAMTQRAH--GRRTVPQIFIGGQ------HVGGCDDLYALEDA--GKLDPMLAD---------------------------- |
| Q4C5G3 | UniRef cluster | ---------------------------------------MLNLFNSLLGRDPQKIKADVEIYTWQTCPFCIRAKLLLWWK-GVNFTEYKIDGDETAREKMAQRSS--GKRSVPQIFVNNQ------HIGGCDDLYSLDGQ--NQLDPLLIMDNG------------------------- |
| A7JLG1 | UniRef cluster | ------------------------------------------------MNNFKSIVLYRMVTPEKICPYGLKAKALFEQKGWSFEDNHLKTRAETDAFKAKYNLQT--------TPLIFIDG---QQIGGYSDLLEFLGEK-------------------------------------- |
| A7JHB2 | UniRef cluster | ------------------------------------------------MNNFKSIVLYRMVTPEKICPYGLKAKALFEQKGWSFEDNHLKTRAETDAFKAKYNLQT--------TPLIFIDG---QQIGGYSDLLEFLGEK-------------------------------------- |
| A6UXI1 | UniRef cluster | ------------------------------------------------------MTTDVLLYTTNWCPFCRRAKALLKEK-GVRWKELDIEADPAHRQAMAEASG---RSSVPQIFINGT------LIGGSDELFALDVR--GELDKLLGRNPPAT----------------------- |
| A3PNP1 | UniRef cluster | -------------------------------------------------------MKSVEIYTTPTCGYCQAAKSLLRRK-GVSYAETDVSTDPSLRAAMTQRAH--GRRTVPQIFIGGQ------HVGGCDDLYALEDA--GKLDPMLAD---------------------------- |
| A2CDA7 | UniRef cluster | -------------------------------------------------------MAKVEIYTWQSCPFCLRAKALLDRK-GVSYQEHAIDGDQDARAAMANRAG--GSNTLPQIFIDDL------SIGGCDELHALEGA--QKLDGLLQGKG-------------------------- |
| A0Q5Y8 | UniRef cluster | ------------------------------------------------MNNFKSIVLYRMVTPEKICPYGLKAKALFEQKGWSFEDNHLKTRAETDAFKAKYNLQT--------TPLIFIDG---QQIGGYSDLLEFLGEK-------------------------------------- |
| Q6MNE8 | UniRef cluster | -----------------------------------------MKPCLPHLPMSKRIMAKVLIYKKIPCPYCDRAMHLMDDR-GIDYDVVDLTDKPEEIERIKTETG---WRTVPIIMINGK------LIGGYTDLKALDEE--GKLMPLLQE---------------------------- |
| Q4C787 | UniRef cluster | ------------------------------------------------------MAANVEIYTWSTCPFCIRAKALLDKK-GVNYTEYCIDGDEDAREIMAERAN--GKRSLPQIFINDG------HVGGCNELYDTELA--GELDSLLEQSVAS------------------------ |
| A9DYH2 | UniRef cluster | -------------------------------------------------------MQPVEIYTSPLCGFCHSAKRLLTQK-GAAFDEVDVLSEPERKKEMIQRAG--GARTVPQIFIGDV------HVGGCDELYALDRA--GKLDALLQDT--------------------------- |
| A0NNY4 | UniRef cluster | -------------------------------------------------------MAEVVIYTRQLCGFCTAAKRLLDKK-GVAYTEHDATFDPGLRKEMVQKAN--GHSTFPQVFVGKT------HVGGCDDLHDLERA--GKLDALLAS---------------------------- |
| Q92MJ8 | UniRef cluster | ----------------------------------------------------METMASVVIYTRQFCGYCTRAKKLLESK-GVDFTEYDATYAPELRQEMIEKSR--GGRTFPQIIINDV------PVGGCDDLHALDRA--GKLDDLLAA---------------------------- |
| Q3AVY9 | UniRef cluster | -------------------------------------------------------MAKVEIYTWRTCPFCIRAKALLDGK-GAAYTEISVDGDEPGRDAMAARGN--GRRSVPQIFINDA------HVGGCDELHGLERA--GELDALLNA---------------------------- |
| B0C104 | UniRef cluster | -------------------------------------------------------MANVEIYTWSTCPFCRRAKHLLDQK-GVTYTEYVLDGDETARDAMVARGTQ-GRRSVPQIFINDQ------HIGGSDALYDLEQQ--GKLDGLLNSSLAGATPLKPYQRGRNISGT-------- |
| Q1ZEK7 | UniRef cluster | -------------------------------------------------------MVAVTIYTTPWCPYCIRALRLLDNK-KVQYTQIDVS-DPTERAKMQALTG---GHTVPQILINAQ------PIGGCDELYALEHE--AKLDALLNAS--------------------------- |
| Q061J7 | UniRef cluster | -------------------------------------------------------MAKVEIYTWRTCPFCIRAKALLDGK-GAAYTEISVDGDEPGRDAMAARGN--GRRSVPQIFINDA------HVGGCDELHGLERA--GKLDSLLNA---------------------------- |
| B0JIU4 | UniRef cluster | ---------------------------------------MLNLFNSLFGRYNENIKANVEIYTWATCPYCIRAKWLLGWK-GVKYTEYKIDGDESARQAMAERSN--GKRSVPQIFINNE------HIGGCDDLYALDGQ--KKLDNLLAKCGTI------------------------ |
| A8YHC5 | UniRef cluster | ---------------------------------------MLNLFNSLFGRYNENIKANVEIYTWATCPYCIRAKWLLGWK-GVKYTEYKIDGDESARQAMAERSN--GKRSVPQIFINNE------HIGGCDDLYALDDQ--KKLDNLLAKCGTI------------------------ |
| A6UCF9 | UniRef cluster | -------------------------------------------------------MASVVIYTRQFCGYCTRAKRLLESK-GVDFIEYDATYAPELRQEMIEKSK--GGRTFPQIIINDV------PVGGCDDLHALDRA--GKLDDLLAA---------------------------- |
| Q605Y9 | UniRef cluster | -------------------------------------------------------MLPVRMYSTAMCPFCSAAERLLKSKGVVEIEKIRVDLDPARLQEMMTIT---HRRTVPQIFIGDR------HVGGFDDLAALERT--GELGELLSGPA-------------------------- |
| A0YQC8 | UniRef cluster | ---------------------------------------MFDFLNPILGRHPEQVKANVEIYTWQTCPYCIRAKLLLWWK-GVNYTEYKIDGDNTARNQMAERAN--GRRSVPQIFINHQ------HIGGCDELYQLNSQ--GSLDPLLAESVAI------------------------ |
| Q3SJS1 | UniRef cluster | -------------------------------------------------------MNAVKVYSTGTCPICVKAKAFLDKRGIG-YDEVRIDLDREAMKEFSVVTN--GARTVPQIVVDGT------CIGGFTELTELDMD--GGLDHLHPASPGGQGG--------------------- |
| Q05VL8 | UniRef cluster | -------------------------------------------------------MAKVEIYTWRTCPFCVRAKALLDGK-GVAYTEHSVDGDEPARDAMAARGD--GRRSVPQVFINDV------HIGGCDDLHARERA--GDLDALLAQG--------------------------- |
| A5CVT5 | UniRef cluster | -------------------------------------------------------MKKNIIYCSDSCFFCQKAYQLLEKKGIHFKKH---YVRTQVDWNEVKEKTN--RTTVPQIFINDF------YIGGFDDLSAAEQS--GKLDEILNKT--------------------------- |
| A3V2C0 | UniRef cluster | -------------------------------------------------------MQTIEIYTKPTCGFCHMAKRVLTAK-GVSFTEVNITAQPEKRAEMIQRAK--GGSTVPQIFIGGK------HIGGCDDLMALDRQ--GKLDGLLSA---------------------------- |
| Q0AMD8 | UniRef cluster | -------------------------------------------------------MTDITIYTRPMCGYCARAVSLLKQK-GVAFTEIDAGFDQAKRQEMIQRSN--GGRTFPQIFIGDM------HVGGCDDLMALERG--NKLDALLQA---------------------------- |
| A6FL83 | UniRef cluster | -------------------------------------------------------MQPVEIYTSPLCGFCHAAKRLLKQK-GANFTEVDVLVEPARKPEMIQRAG--GKKTVPQIFIGDI------HVGGCDELYALERA--GKLDALLSA---------------------------- |
| A3SUM6 | UniRef cluster | -------------------------------------------------------MQPVEIYTSPLCGFCHAAKRLLNEK-GVSFAEVDVLAQPERKSEMIERAN--GGRTVPQIFIGDT------HVGGCDDLYALERA--GKLDALLAS---------------------------- |
| A3S9R9 | UniRef cluster | -------------------------------------------------------MQPVEIYTSPLCGFCHAAKRLLNEK-GVSFAEVDVLAQPERKSEMIERAN--GGRTVPQIFIGDT------HVGGCDDLYALERA--GKLDALLAS---------------------------- |
| A0ZBB8 | UniRef cluster | ---------------------------------------MQNFLNPLFGRHPEQVKANVEIYTWQTCPYCIRAKMLLWWK-GVRFTEYKIDGDEAARAKMAERAN--GRRSVPQIFINHQ------HIGGCDDLYQLDTK--AQLDSLLAQPAI------------------------- |
| A6E4Y2 | UniRef cluster | -------------------------------------------------------MKPVEIYTSPLCGFCHSAKRLLQKK-GVNFSEINVLAQPARKSEMMKRAN--GRHTVPQIFIGTT------HVGGCDDLYALEQA--GKLDALLRG---------------------------- |
| A4E890 | UniRef cluster | ----------------------------------------------------MATDHELTLYVMTGCPYCIKVKHFLADNGVTIPERN-----ISTDSDAEQTLIAVGGKRQVPCLFIDGEP----LYESNDIIAWVQENLL------------------------------------- |
| A0Z8E4 | UniRef cluster | -------------------------------------------------------MTEIVMYTTRWCPFCTGAKTLLNKK-GVEFSEIPVDGDPALRASMAERAG---ATSVPQIWIGDA------HIGGCDELFSLEQQ--GRLDSMLSGQR-------------------------- |
| Q5GSM1 | UniRef cluster | ---------------------------------------------------MKNAKRKVVIYIKQYCPFCKRAKELLDEK-GVKYEEIDVFKNSDLFSNIKLKYN---VRTVPQIFIADKNGNYVHHIAGCDKLMDLERE--GKLDDMLSTNYDKTDVTTYTSNSDEYEEYVASHDDFM |
| Q4FRF5 | UniRef cluster | ------------------------------------------------------MTVSVKVYTTPICPYCSNAKQLLKSKGVDYEEIGMHDMSSDDRRALMQKTNN--YRTVPQIFVGET------FVGGFDELNQMNQQ--GKLDELLAG---------------------------- |
| Q1Q9Y9 | UniRef cluster | ------------------------------------------------------MTVSVKVYTTPICPYCSNAKQLLKSKGVDYEEIGMHDMSSDDRRALMQKTNN--YRTVPQIFVGET------FVGGFDELNQMNQQ--GKLDELLAG---------------------------- |
| Q167B3 | UniRef cluster | ------------------------------------------------------MPPEITLYTKGYCPHCKAARALLSAK-GVRFVNHDIDITPERRGEMIARAG--GRTTVPQIFIADF------HVGGNSDLTALNSS--GTLDALLNIKQPA------------------------ |
| Q162E9 | UniRef cluster | ------------------------------------------------------MPPEITLYTKGYCPHCKAARALLSAK-GVRFVNHDIDITPERRGEMIARAG--GRTTVPQIFIADF------HVGGNSDLTALNTS--GTLDALLNIKQPA------------------------ |
| A9H6T3 | UniRef cluster | -------------------------------------------------------MKNVEIYTSPLCGFCHAAKRLLSQK-GITFAEVDVLADPGRKPEMVQRAN--GSRTVPQIFVGDL------HVGGCDDLYALERA--GKLDQLLAA---------------------------- |
| Q1GWX1 | UniRef cluster | ------------------------------------------------------MKPNITLYTKDYCPFCHRAKAHLRAKGVTDWIEIDVEDDPVQFRAMQTASG--GRRTVPQIFINGT------HVGGSDDLVALDAD--GGLDLLLREKMLV------------------------ |
| Q0FV64 | UniRef cluster | -------------------------------------------------------MPAIEIYTTPICGFCHAAKRLLTQK-NAEFTEIDVMQEPKRRSEMTQRAN--GGRTVPQIFIGET------HVGGCDELYALERA--GKLDALLAG---------------------------- |
| A3YUF7 | UniRef cluster | ----------------------------------------------------MNTPAKVEIYTWRFCPFCIRAKALLDRK-GVAYEEYAIDGDQAARGAMAQRAD--GRSSLPQIFINDQ------GIGGCDELHSLERA--GRLDPLLQGA--------------------------- |
| B0VEX3 | UniRef cluster | ------------------------------------------------------MSAKVIVFSTPSCIWCKKAKEYLKSINQNFTDIDVSRDIAARNDMIRKSGQEAVPQIWINNIPVVG-----FDLEKINRLLNLYNTKKGESNG-------------------------------- |
| A9F1N3 | UniRef cluster | -------------------------------------------------------MKPVEIYTSPLCGFCHAAKRLLTQK-GVSFDEIDVLANPGRKAEMIERAN--GGRTVPQIFVGET------HVGGCDDLYALDRS--GKLDPLLAA---------------------------- |
| GLRX1 | UniRef cluster | ------------------------------------MANLFNWLPLLSGRQADGIKAKVEIYTWQTCPFCIRAKLLLWWK-GVKFIEYKIDGDDQARQAMAARAE--GRRTVPQIFVNDQ------GIGGCDQLYGLDSR--GQLDPLLATPPNPA----------------------- |
| Q98G79 | UniRef cluster | -------------------------------------------------------MVDVTIYTRMMCGYCTAAKRLLERK-GVAYTEHDASFSPELRQEMISRAH--GRTTFPQIFIGET------HVGGCDDLHELEAE--GRLDRLLANGATI------------------------ |
| Q1GCI1 | UniRef cluster | -------------------------------------------------------MKPVEIYTSPLCGFCHAAKRLLNQK-GVSFSEVDVLANPDRKSEMIQRAN--GGRTVPQIFVGDI------HVGGCDELHALDRA--GKLDSLLAS---------------------------- |
| Q0FE13 | UniRef cluster | -------------------------------------------------------MNKIEIYTGNFCGFCTAAKRLLNKK-GANFSEINIHDNVEKRAEMIQRSN--GGRTVPQVFINDT------HVGGCDDLYDLENM--GHLDKLLIA---------------------------- |
| Q07VC0 | UniRef cluster | ------------------------------------------------------MTAAIEIFTRPGCGYCSAARSLLTRK-NAAFIEYDVSVDPNFRLKMIDRVG--AGATYPQIFIGKL------HVGGCDELYALDRE--GRLDSLLAGETAAS----------------------- |
| A3W7L4 | UniRef cluster | -------------------------------------------------------MQPVEIYTSPLCGFCHSAKRLLQKK-GVNFSEINVLAQPARKSEMLKRAN--GRHTVPQIFIGST------HVGGCDELYALDQA--GKLDVLLRG---------------------------- |
| Q47VY4 | UniRef cluster | -------------------------------------------------------MAKVEIYTKEYCPYCTHAVALLKSK-QTEYNEIKIDDDMEMRSTMIERTN--GGYTVPQIFINDV------HIGGCDQLVALERN--TQLDALLDAK--------------------------- |
| A8TK57 | UniRef cluster | ------------------------------------------------------MAKKIEIYTTMFCPFCHRAKALLKNK-GVAFTEYDVGGSSDERARMRERAD--GRHTVPQIFIDGV------GIGGSDELAALDRQ--GKLDPMLGLTA-------------------------- |
| A4BAV9 | UniRef cluster | --------------------------------------------------------MQDIKVLGSGCAKCEKTASLIEKLAQEQGASVTVEKETRPEALITYGVMRTPAVVIDGQLVHSG------GIPATTDIVSWFKQN-------------------------------------- |
| A1SZI1 | UniRef cluster | -------------------------------------------------------MATIVIYTTSWCPFCTRAKKLLDHK-NVTYTEVDVS-SSDARAKMVALTG---GSTVPQLLINDK------PEGGCDELYALERS--GKLDKLLSQ---------------------------- |
| B0TZX3 | UniRef cluster | -----------------------------------------------MTQQFNKIVLYRMVTPEKTCPYGLKAKALFEEKGWCFEDHILKTRAETDAFKHKYNLET--------TPLIFIDG---KQIGGYSDLLEFLGQK-------------------------------------- |
| A9HMS2 | UniRef cluster | ------------------------------------------------------MPPEITLYTKGYCPHCKAARALLAAK-GVRFVNHDIDITPARRREMIARAG--GRTTVPQIFIADF------HVGGNSDLTALNTS--GTLDALLKNKQTA------------------------ |
| A3X778 | UniRef cluster | ------------------------------------------MAQNIDITQLETRMKSVEIYTSPLCGFCHAAKRLLNQK-GVEFSEVNVLTNPGRKQEMIQRAN--GGYTVPQIFIGDT------HVGGCDDLYALEQA--GKLDSLLAA---------------------------- |
| A5WDL9 | UniRef cluster | ------------------------------------------------------MTAPVTVYTTPICPYCSNAKQLLKSKGIEFKEIGMHDISSDERMELMKKTNN--YRTVPQIFIGDT------FVGGFDQLNQLNQS--GKLDEMVNG---------------------------- |
| A4ETC8 | UniRef cluster | ---------------------------------------------------METCMKPVEIYTSPLCGYCHAAKRLLKQK-GVTFSEVNVLADPGRKGEMIKRAN--GGRTVPQIFIGDT------HVGGCDDLYALEQA--GKLDSLLAA---------------------------- |
| Q097W0 | UniRef cluster | ----------------------MARLFLSQDKVSPAVQEFVGQFHRSVVETVAGTVAREHIVVVGMAQNPFVRRARKLLDEEKLKFTYLEYGSYFSMWKERLALKMWAGFPTFPMVFIDG-----TLVGGFTDLKALKER--GQLR--------------------------------- |
| Q2CJR1 | UniRef cluster | -------------------------------------------------------MANIEIYTTQTCGYCHAAKRLLTQK-GAGYTEIDAS-DPARRAEMTQRAK--GGRTVPQIFIGDT------HVGGCDELFALERA--GKLDALLAA---------------------------- |
| Q223J5 | UniRef cluster | ----------------------MPRPILAESNIHPAIREKIATHEQGIVREVQAAVAKHAVVVVGMGLNPFPKKARKALDAAGVAHHYLEYGNYFSNWRQRNALKLWTGWPTLPMVFVKG-----VLVGGADDLIKLIDN--GELTKLLA----------------------------- |
| A9D7R9 | UniRef cluster | ---------------------------------------------------MEIMMADVTLYTRQFCGFCTAAKRLLDSK-NVAYTEHDATFSPELKQEMIGKAN--GRATFPQIFIDGL------HVGGCDELHALEHA--GKLDPLLEGA--------------------------- |
| A1VUM3 | UniRef cluster | ----------------------MPRPILEESRIHPAIRSKVAGNQQAIVQEVIKAVRENDVVVVGMGMNPFPKKACKALDQAGQPYRYLEYGNYFNTWRERNALKLWTGWPSFPMVFVKG-----MLVGGATDLQALIGS--GELKKMLALPAHGA----------------------- |
| A8LQ00 | UniRef cluster | -------------------------------------------------------MQPVEIYTSPLCGYCHAAKKLLSQK-GVSFAETDVWRAPQKKPEMIERAG--GRTSVPQIFIGAT------HVGGCDELYALERA--GKLDALLAG---------------------------- |
| A8IF94 | UniRef cluster | ------------------------------------------------------MNERWVLITQPRCRPCEEAKQMLTERGIPFVAFD-----IVERPDMRDFLKTLLPNPTTPQVFRDG-----TRIGGRDDLREYFRA--ADLCERF------------------------------ |
| Q1YU85 | UniRef cluster | ----------------------MSRHILDSAKIHPAIQETVASKNLDIVLEVQQAIASNKIVVVGMAQNPAPKRARKTLDALGAEHAYLEYGSYFSLWYRRNALKMWTGWPSFPMIFVDG-----VLIGGNSDLNALIES--GEFAKMTGK---------------------------- |
| A9GNQ4 | UniRef cluster | ---------------------MAERPTLAPEKISDTVKSQIAGFHRSIVDEVAAAVSRDRVVVVGMAQNPFVKKARQLLDGEGVKYTYLEYGSYLSMWKERLAIKLWAGFPTFPMVFVGG-----VLEGGYAELVKLRDQ--GKLKGSP------------------------------ |
| A6GBX2 | UniRef cluster | -----------------MADSNSPRPVHPSDACSETVRRQQAAFHPDVLEAVINAVNSQDVVVVGMATNPHVKKARLGLRNADIPFTYLEYGSYTKGWRQRLAIKMWSGYPTFPQVFVKG-----TLVGGNAELQGMLSD--GSLAAQLAG---------------------------- |
