# Supplementary material for: Evolution based on domain combinations: the case of glutaredoxins
Source: BMC Evol Biol. 2009 Mar 25;9:66. doi: 10.1186/1471-2148-9-66 (PMC2679010; doi:10.1186/1471-2148-9-66)
Supplement: Additional File 3 — GRX sequences from eukaryotes. UNIPROT links to the GRX sequences from eukaryotes, together with protein and DNA alignments and links to other sequences that have over 90% sequence identity. [file 1471-2148-9-66-S3.htm]

Supplementary file 3: Eukaryotes


#### Alignment for Eukaryote GRXs

|  |  |  |  |
| --- | --- | --- | --- |
| UNIPROT accession number | UNIPROT 90\% similarity cluster | Aligned Sequence | DNA Sequence |
| Q7QC85 | UniRef cluster | -----------------MNVLTRNLAQTLFKYNGAGLIQAAARSFSAPALDGKEIEKLVSNNKVVVFMKGNPDAPRCGFSNAVVQILR---MHSVKYDSHDVLQ----NEALRQGIKDFSNWPTIPQVFINGEFVGGCDILLQMHQNGELIDELKKAGIESALAKESEK----------------------- | atgaacgtccttacgcgaaatttggcccaaactctgttcaaatacaatggggccgggttg atccaggcggccgcccgcagcttcagtgcgccggcactggatggcaaggagatcgagaag ctggtgagcaacaataaggtggtcgttttcatgaaaggcaacccggacgcaccgcggtgc ggcttcagcaatgcggtggtacagatactgcggatgcattcggtcaaatacgacagtcac gatgtgctgcagaatgaagccctccggcaaggcattaaggacttttcgaactggcccacc atcccgcaagtgttcatcaacggagagttcgttggcgggtgcgacatactgctgcagatg caccaaaacggcgagctgatcgatgagctgaaaaaggccggcatcgagagcgcactggcg aaggagtccgagaagtga |
| A8PJS6 | UniRef cluster | -----------------MFGLTKIIRQVAFRAPG-SYLFSSASSIPLPEALKMRIENMISSAPVVVFMKGTQLEPMCGFSKNVKLVLD---FHEVKFKDYNVLG----DDDLREGIKTYSDWPTIPQVYVNGNFIGGCDILVQMHKKGEITDLFEKEGIKTRFSNALNDEIKTLG----------------- | atgtttggcttgacaaaaattattaggcaagttgcatttcgtgcacccggttcatatctt ttttcctctgcatcgtcaataccgttacctgaagcgttgaaaatgcgaattgaaaatatg atctcatctgctccggtagtagttttcatgaaaggtacgcagctggaaccaatgtgcggc tttagcaaaaatgtcaaattggtattagattttcatgaggttaaattcaaagattataac gtactcggcgatgatgacctaagagaaggtattaaaacatatagtgattggcctacgata cctcaagtttacgtgaatggcaacttcataggtggttgtgatattcttgttcaaatgcac aaaaagggtgaaataacggacctcttcgagaaagaaggtataaaaacacgattttcgaat gctttaaacgacgaaataaaaacattaggatga |
| Q02784 | UniRef cluster | -----------------MFLPKFNPIRSFSPILRAKTLLRYQNRMYLSTEIRKAIEDAIESAPVVLFMKGTPEFPKCGFSRATIGLLGNQGVDPAKFAAYNVLE----DPELREGIKEFSEWPTIPQLYVNKEFIGGCDVITSMARSGELADLLEEAQALVPEEEEETKDR--------------------- | atgtttctcccaaaattcaatcccataaggtcattttcccccatcctccgggctaagact cttcttcgttaccaaaatcggatgtatttgagcacagagataagaaaagctattgaagat gccatcgaatcggctccagtggttcttttcatgaaaggtactcctgaatttcccaagtgt ggattttcaagagcaaccattggattattaggaaatcaaggcgttgacccggccaaattt gcggcttataatgttttagaagacccagagctacgtgaaggtatcaaagagttttcagaa tggccaactattccacagttatatgtaaacaaagaattcattggtggatgtgatgttatt acaagtatggcacgctctggtgaattggccgatttgctagaagaggcacaggcattggta cctgaagaagaagaagaaaccaaagatcgttga |
| Q17M81 | UniRef cluster | -----------------MSLLVRKLGSSAFGSRTS---FFLARTLCANAVDAKEIDKLVHNNKVVVFMKGNPEQPRCGFSNAVVQILR---MHAVNYDSHDVLQ----SDALRQAIKDYSNWPTIPQVFINGEFVGGCDIMLQMHQNGELIDELKKVGITSALVDAAGAEEKK------------------- | atgagtctactggtgagaaaacttggatccagtgccttcggcagccgcaccagtttcttc ctggcgcgaacgctttgcgccaacgccgtagatgccaaggagattgacaaacttgtacac aacaacaaggtcgtcgtgttcatgaagggaaacccggaacaaccgcggtgtggattcagc aatgccgtggtccagatcctgcggatgcatgccgtgaattacgacagccacgacgttctg cagagtgatgccttgcgacaggccatcaaagactattccaactggccaacgattccgcag gtttttatcaacggagaatttgtcggaggatgcgacatcatgctgcagatgcaccaaaac ggagaactcatcgatgagctgaagaaggtcggcattacgagtgccttagtggatgcggcc ggagcagaagagaagaagtag |
| A6ZWI9 | UniRef cluster | -----------------MFLPKFNPIRSFSPILRAKTLLRYQNRMYLSTEIRRAIEDAIESAPVVLFMKGTPEFPKCGFSRATIGLLGNQGVDPAKFAAYNVLE----DPELREGIKEFSEWPTIPQLYVNKEFIGGCDVITSMARSGELADLLEEAQALVPEEEEETKDR--------------------- | atgtttctcccaaaattcaatcccataaggtcattttcccccatcctccgggctaagact cttcttcgttaccaaaatcggatgtatttgagcacagagataagaagagctattgaagat gccatcgaatcggctccagtggttcttttcatgaaaggtactcctgaatttcccaaatgt ggattttcaagagcaaccattggattattaggaaatcaaggcgttgacccggccaaattt gcggcttataatgttttagaagacccagagctacgtgaaggtatcaaagagttttcagaa tggccaactattccacagttgtatgtaaacaaagaattcataggtggatgtgatgttatt acaagtatggcacgctctggtgaattggccgatttgctagaggaggcacaggcattggta cctgaagaagaagaagaaaccaaagatcgttga |
| Q5B576 | UniRef cluster | ---------MFSRTAISFTLRRSLFRPQLSPQFP-NQLPSVLQARLLSTETKAAIDKAVASAPVVLFMKGTPETPQCGFSRASIQILGLQGVDPKKFVAFNVLE----DPELRQGIKEYSDWPTIPQLYLNKEFIGGCDILMSMHQNGELSKLLEEKGVLVAE----------------------------- | atgttctcaagaacagcaatctccttcacgctccgacggtctctgtttcggccacaactc tcccctcagtttccaaaccagcttccatctgtgctccaagcccgtctactttcaacagag accaaagcagcaattgataaagctgttgcatcggctccggtggttcttttcatgaagggc actccggaaactccgcagtgcgggttctctcgcgcaagcatccagattttgggtttacaa ggtgttgatcccaagaaattcgtagcgtttaacgtgctagaggatccggagctgcgtcaa ggtatcaaggagtactcggactggcctacgattccacaattatacctgaacaaggagttt attggtgggtgtgatattctgatgtcaatgcatcagaatggcgaactttcgaagctctta gaagagaagggtgtcctagttgctgaatag |
| A7TMJ7 | UniRef cluster | ---------------MFLKGFNPLVRTSISQLSRSNILFRYQSRLFLSAETKKAIDDAVSSAPVVLFMKGTPEFPQCGFSRATINILGQQGVDPQKFAAYNVLE----DPELRQGIKEYSEWPTIPQLYVNKEFVGGCDIITTMAQDGQLADLLEEADVLVPEDHE-------------------------- | atgtttttaaaaggctttaatccgttagttagaacttctatatcacaattatctcgatcg aatattttatttaggtatcaatcacgattattcctaagtgctgagaccaaaaaggctata gatgatgcagtaagctctgctcctgtagtattatttatgaagggtactccagaatttcca caatgtggtttctcgagagcaactattaatatattaggtcaacaaggtgttgacccacag aaatttgcggcttataatgtattagaagatccagaattacgtcaaggcattaaggaatat agtgagtggccaaccattccgcaattatatgtgaataaggagtttgttggtggttgtgat attattactactatggcccaagatggccaattagctgatctcttagaggaagctgatgtt ctagtgccagaagaccatgaatga |
| Q1XDA3 | UniRef cluster | ----------------------------------------------MDTETKKRIDQLLNDHKIVLFMKGNKTMPMCGFSNTATQILN---TLNIKYFTYDILE----DENIRKAIKEYSSWPTIPQLYINREFIGGADIMLELFETGELQAQVEALLAT-------------------------------- | atggatacagaaacaaaaaaaaggattgatcagttattaaatgatcacaagatagtgtta tttatgaaaggcaataaaacaatgccaatgtgtggattttcaaatacagcaactcaaatt ttgaatactttgaatattaaatactttacttatgatatcttggaagatgaaaatattcgt aaagctataaaggaatactctagctggccaactatccctcagttatatattaatcgagaa tttattggaggggcagacatcatgctagaactttttgagacaggtgaattacaggctcag gtagaggctttgctagctacataa |
| Q2UG28 | UniRef cluster | ---------MFSRVAFSSAFRPLSRPQSLPLRPYGFQLPSALYARLLSSETKSAIDKAIASAPVVLFMKGTPETPQCGFSRASIQILGLQGVDPKKFVAFNVLE----DAELRQGIKEYSDWPTIPQLYLDKEFVGGCDILMSMHQNGELAKLLEDKNVLVAAD---------------------------- | atgttctcaagagtggctttctcgtcggcattccgaccgctttcgcggccacagtcgctg ccgctacgaccctatggttttcagttaccatctgccctttacgctcgactactctcatct gagacgaaatcggccattgataaagccattgcatctgccccggttgtccttttcatgaag ggtacccccgagactcctcagtgtggcttctcgcgagcaagtatccagatcttgggcttg caaggggtggatcccaagaagtttgtcgcattcaacgtgctggaggatgctgaattgcgt cagggtatcaaagaatactccgactggccaaccatcccacagttatacttggataaggag tttgttggtggctgtgatattctaatgtctatgcatcagaatggggaacttgcaaaattg cttgaagacaagaatgttctggtggcggctgactaa |
| A2QHK0 | UniRef cluster | -----------------------MNRSTLQPRFAGIQLRSNLYARYLSSETRTAIDKAVASAPVVLFMKGTPETPQCGFSRASIQILGLQGVDPKKFVAFNVLE----DSELRQGIKEYSDWPTIPQLYLEKEFIGGCDILMSMHQNGELAKLLEEKGVLVAAD---------------------------- | atgaacagatcaactttgcaacccagatttgctggcattcagctgcgctctaacctctat gcccggtatctgtcttcggagacacggacagctattgacaaagctgtcgcatctgcacct gtcgttcttttcatgaagggcacgccagagacgccgcagtgcggtttctctcgggccagc atacaaattttgggactgcagggagtggacccgaagaaattcgttgcattcaatgtgttg gaagattcagaacttcgtcagggtatcaaagagtattcagactggcccacaattccgcaa ttgtatttggagaaggagttcatcggtggttgcgatatattgatgtctatgcatcaaaat ggagaacttgctaaattgctcgaggagaagggggtccttgtggcggccgactaa |
| Q6YFE4 | UniRef cluster | -----------------MFG--RISTRALLRPAFTHRIPSVSLSRFLSTETKQAIESAIESAPVVLFMKGTPEFPQCGFSKATINMLGQQGVDPMKFAAYNVLE----DAELREGVKEFSEWPTIPQLYVNKEFVGGCDIVMNMAQTGELAKLLEDADALVPEEEE-------------------------- | atgtttggtagaatatccactagagctcttttgcgcccagcttttacccacagaatacct tccgttagcctttctaggtttttgagtacagagactaaacaagcaattgaatccgcaatc gaatctgctccggtcgttctgtttatgaaaggtaccccagagttccctcaatgtggattt tcaaaggccactatcaatatgttaggccaacaaggcgtagacccaatgaagtttgctgct tacaatgtgcttgaggatgccgaactgcgtgaaggtgttaaagaattcagtgaatggcca actatcccacagctatacgttaacaaagaatttgttggtggttgtgatattgtcatgaac atggctcaaactggagagctagctaaattattagaggatgctgatgcactagtccccgaa gaagaggaatga |
| Q6FJD0 | UniRef cluster | -----------------MFATRLLGKSALLGTTSTRMVFRSQMARYMSTETKKAIEGAIASAPVVLFMKGTPEFPQCGFSRATISMLGQQGVDPEKFAAYNVLE----DPELREGIKEFSNWPTIPQLYVNKEFIGGCDVITSMARSGELADVLEEANALVPEEKEEGTD---------------------- | atgttcgctacaagattattaggaaagagtgctttattgggcaccaccagtactaggatg gtgttcaggtcacaaatggcacgttacatgagtactgagaccaagaaagctattgaaggt gctattgcatctgctcctgttgttctattcatgaaaggtacacctgagtttccacaatgt gggttttctagggccaccatctcgatgcttggtcagcaaggtgttgatccagagaagttt gccgcatataatgtcttggaagacccagagttacgtgaaggtatcaaggagtttagcaac tggccaaccattccacagctgtatgttaacaaagaatttatcggtggttgcgatgttatc actagtatggcccgttctggtgaattagcagatgtactggaagaggcaaatgcattagta ccagaagagaaagaggaaggaactgactaa |
| Q6CVT2 | UniRef cluster | -----------------MFSRFLATKSPLLRPAFRS---HLSFQRFISQETKKAIEDAIESAPVVLFMKGTPEFPQCGFSRATINMLGQQGVDPVKFAAYNVLE----DPELREGIKEISEWPTIPQLYVNKEFVGGCDIVMNMAQTGELAKLLEESDALVPEEED-------------------------- | atgttctcaagatttttggccactaagagtccgctcctacgtcctgctttcagatcacat ctaagtttccaaagattcatatcacaagagacaaagaaagctattgaagatgctattgaa tcagcaccggtggttttattcatgaagggcactccagaattcccacaatgtggattctct agagctacaatcaatatgttaggtcaacaaggtgtcgatccagtgaagtttgctgcttac aatgtacttgaagaccctgagttacgtgaaggtatcaaggaaatctccgaatggccaacc atcccacaactatacgttaacaaggaatttgtcggtggatgtgacattgtgatgaacatg gctcaaacgggtgaattagctaaactgttggaagagtccgatgctttggtcccagaagaa gaagattaa |
| A9RF74 | UniRef cluster | -----------------------------------------------------MGIQDVKENPVLIYMKGVPDAPQCGFSAMVVHILDEYGVR---YKTRNVLS----DPELRNAIKEYSNWPTIPQVYVNGEFVGGSDILISMHRSGELKTLLKDVKNIR------------------------------- | atggggatacaggacgtcaaggaaaatccagtattgatttacatgaaaggagttcctgat gcacctcagtgcggattcagtgccatggttgttcacatattggatgaatatggagtacgg tataagacgaggaatgttctgtccgacccggagcttagaaatgccatcaaagagtacagt aattggccaacaattccacaagtttacgtgaacggcgagtttgttggaggatccgacatt ctcatctccatgcaccggtcaggggagctgaaaacattgctgaaagacgtgaagaacatc aggtaa |
| Q4CNA8 | UniRef cluster | --------------------------MFASRLLLLRLTRASHGPEKVAPALAASIKDIILKDRVVIFLTGTPEEPRCRFTAQMVDMMQ---QMGVQYSFFNVLD----DDDVCEGLKTYSDWPTYPQLYIDGELVGGYDVCKNMLLNGQLTRLFQEKKLL-------------------------------- | atgtttgcgtctaggcttctgttgctgcgccttacgagggcaagccatgggcccgaaaag gtggctcccgcactcgcagcatccatcaaagacatcatcttaaaagatcgtgtggtcatt tttttaaccggcacaccggaagaacctcgctgccgatttacggcacagatggtagatatg atgcagcagatgggagtccagtatagctttttcaatgttcttgacgatgatgatgtctgt gagggacttaagacgtacagtgactggccgacgtatccgcaactttatatcgatggggaa cttgttggaggatacgatgtgtgcaaaaacatgttactaaatggacaattaacaaggctt tttcaggagaagaaactgctttga |
| A8QAM5 | UniRef cluster | ------------------------------------MRASPSLVRLLSTETRTKIQNAVKEDPLVVFMKGSPSMPQCGFSRAVLQILQVQGVNPDKVATYNCLE----DQELRDGIKEFSDWPTIPQVYINGEFVGGCDIMLNMHQTGELESTLRKAGLLLEPLDADKSSSSN------------------- | atgcgcgcgtctccgtcgctcgtgcgtctgctttcgacagagacacgcaccaagattcaa aatgccgtgaaagaggacccccttgtcgtgttcatgaaaggttcgcctagtatgccgcaa tgtggattcagccgtgccgtgctccaaattctccaggtgcaaggcgtgaaccctgacaag gttgcgacgtacaattgtctcgaggatcaggaactgcgtgacggcatcaaagaatttagt gactggccaaccattcctcaagtatacattaatggcgagtttgtcggcggttgtgatatc atgctcaatatgcatcagacgggcgagctcgaaagcactcttcgtaaggcaggcctcctt cttgagccgctggacgccgacaagtcttcgtcatcgaactaa |
| A7EYX9 | UniRef cluster | ---------MIARPMFSSVFRQAVRPATFASKFSAPRFS-PIASRYLSTEVRKQIDQVVGSKPVVLFMKGTPENPQCGFSKATIQILSLQGLDPEKFAALNVLE----DEGLRQGIKEYSEWPTIPQLYVNKEFIGGCDILITMHQNGELAKVLEENKVLVQESS--------------------------- | atgattgccagaccaatgttttcgtcggttttccgacaagctgtccgcccagcaacattt gcctccaaattctcggcccctcggttctctcccatcgcctctcgatatctctcaacggaa gtccgcaagcagattgatcaagttgttggttcgaagcctgttgtacttttcatgaaaggt acaccggaaaatccacagtgtggcttctcaaaggcaaccatccaaatcttgagtctacag ggcttggacccagagaagttcgctgcccttaacgttctagaggatgaaggtttaagacaa ggaattaaggaatactccgaatggccaaccataccccaactttatgtcaataaggagttt ataggaggatgcgatatcttgattacaatgcaccaaaacggagaattggccaaggtattg gaagagaataaggtcttggttcaggaatcatcataa |
| A7PA82 | UniRef cluster | ------------------------------MSPSLRTHHSFPPLLTLTPELKTTLDKVVTSNKVVLFMKGTKEFPQCGFSNTVVQILN---SLNVPFETINILE----NEILRQGLKEYSNWPTFPQLYIEGEFFGGCDITVDAYNSGQLQEQLEKAMCS-------------------------------- | atgtcaccttctttgaggactcaccattcttttccaccacttctgacactaactcctgag ctgaaaactacattggataaagttgttacatcaaacaaagtggttctttttatgaaggga accaaggagttcccacaatgtggattttcaaacactgtggtgcagatactcaattccctg aatgttccttttgaaacaataaacattttagaaaatgaaatattgcgtcaggggttgaag gaatattccaactggcctacctttcctcaactttacattgaaggggagttctttggtggc tgtgacattactgttgatgcatacaacagcggccaattgcaggaacagttggagaaggcg atgtgctcctga |
| Q9XTU9 | UniRef cluster | -----------------MLRVLSRQAFSMVRAANFSASASSAGGGGLSDEVRKRIDGIVKKDDVVVFMKGTQQEPACGFSRNVKLVLD---FHNVKFQDYNVLT----DQELREGVKIFSEWPTIPQVYVKGEFVGGCDILISMHKDGEISDFLDEKGISNKYGKQ-------------------------- | atgttgcgcgtactatcacgtcaagcgttctcaatggttcgagcagccaacttctcggct tcagcttcatcagccggtggtggcggacttagcgatgaagttcgcaaacgaatcgacgga atcgtgaaaaaagatgatgttgtcgtttttatgaagggaacacaacaggagccagcgtgt ggattctcgagaaatgttaaattggttctcgacttccacaatgtaaaattccaagactac aacgttctgaccgatcaggagctaagagaaggtgtcaagattttcagcgaatggccaaca attccacaagtctatgtgaagggagagttcgtcggcggatgtgatattctcatttcaatg cacaaagacggagagatttccgactttttagacgaaaagggaatctcgaataaatatggg aagcaatga |
| Q75A65 | UniRef cluster | -----------------------MFATYSASILRSTLPLRSVGVRLLSQETRRAIEGAISSAPVVLFMKGTPEFPQCGFSKAAIEILGRQGVDPAKFAAFNVLE----DSELRSGIKEYSEWPTIPQLYVNKEFVGGCDILTNMAQSGELTTMLEEASVLVPDTE--------------------------- | atgtttgcaacatattcggctagtattctccgttcgactttacccctgcgtagcgttggc gtcagactgctgagccaggaaactcggcgggccattgagggcgccatttcctctgcccca gtggttctgttcatgaagggcaccccagagttccctcaatgtggcttttcgaaggccgcc attgagatcctgggcagacagggcgtggatcctgcgaagtttgcggcgttcaacgtgctg gaggattctgagctgcggagcgggataaaggaatattccgagtggcctacaattccacag ctctacgtcaacaaggaatttgttggggggtgcgacatcctcaccaacatggcgcaatcc ggcgagctaactactatgctcgaggaggcatccgttcttgtgccggatactgagtga |
| A9U0J9 | UniRef cluster | -------------------------------------MLRGLCTSALTPELEQAIDKFLSENKVVLFMKGNKQFPQCGFSNTCVQILN---TLNVPYETVNILE----DDNLRQGMKEYSAWPTFPQLYIDGEFFGGCDITYESYNSGELKELLDRAMLS-------------------------------- | atgttgcgtggattgtgcacatcagccctgacccctgaattagagcaagctattgacaag tttctcagcgagaacaaggtggtcttgttcatgaagggtaacaagcaattccctcaatgt ggattctccaacacctgtgtgcaaatcctcaacaccttgaacgtcccctacgaaactgtc aacattctagaggatgataatctgcgacagggcatgaaagaatactctgcttggcctact tttccacagctgtacattgacggtgaattttttggaggatgcgacattacttacgagtcc tacaatagtggggagttgaaggagcttttggacagggccatgttgtcttga |
| A4RRR6 | UniRef cluster | ----------------------------------------------MGPELRESIDAFVAEHRVVLFMKGTKDAPRCGFSNTCVQILN---SMNVPFADVDILA----NEDLRQGMKDYSSWPTFPQLYVGGEFFGGCDITVDAFKDGSLKEELERAMLE-------------------------------- | atggggcccgagctgcgcgaatccatcgacgcgttcgtcgcggaacaccgcgtggtgctc ttcatgaaggggacgaaggacgcgccgaggtgtgggttctcgaacacgtgcgtgcagatt ttgaactcgatgaacgtgccgttcgcggacgtcgatatcctggcgaacgaggacttgaga caggggatgaaggattattcgtcgtggccgacgtttccgcagctgtacgtaggcggagaa ttcttcggtgggtgcgacatcaccgtcgacgcgttcaaggacgggtcgctcaaggaggaa ctcgagcgcgcgatgttggagtga |
| Q389T5 | UniRef cluster | ----------------------MLRRIFASSPAFLRCTLPNRAPDQVKPELAAAIRRIIAEDRIVIFLTGTPQEPRCGFTVKMVDMMH---QLGVKYSFYNILE----DDEVCEGLKIYSDWPTYPQLYIDGDLVGGYDVCKGMLLSGQLTKLLKEKDLL-------------------------------- | atgctgcgccgcatttttgcatccagtcccgctttcctgcgttgcacgctaccgaaccgt gctccagatcaggttaaacctgaacttgcggcggcaatacgcaggattatcgctgaggat cggattgtcattttccttacgggcacgccccaggagcctcgctgcgggtttacggtgaaa atggtggatatgatgcatcaactaggcgtgaagtatagcttttacaacattctggaggac gatgaggtgtgcgagggactgaagatatatagcgactggccaacgtaccctcagttgtac attgacggtgacctcgttggtggctatgatgtttgtaagggtatgcttctcagtggccaa ctgactaagcttctaaaggagaaggatcttctctga |
| B0B1V1 | UniRef cluster | ----------------------MLRRIFASSPAFLRCTLPNRAPDQVKPELAAAIRRIIAEDRIVIFLTGTPQEPRCGFTVKMVDMMH---QLGVKYSFYNILE----DDEVCEGLKIYSDWPTYPQLYIDGDLVGGYDVCKGMLLSGQLTKLLKEKDLL-------------------------------- | atgctgcgccgcatttttgcatccagtcccgctttcctgcgttgcacgctaccgaaccgt gctccagatcaggttaaacctgaacttgcggcggcaatacgcaggattatcgctgaggat cggattgtcattttccttacgggcacgccccaggagcctcgctgcgggtttacggtgaaa atggtggatatgatgcatcaactaggcgtgaagtatagcttttacaacattctggaggac gatgaggtgtgcgagggactgaagatatatagcgactggccaacgtaccctcagttgtac attgacggtgacctcgttggtggctatgatgtttgtaagggtatgcttctcagtggccaa ctgactaagcttctaaaggagaaggatcttctctga |
| Q9HDW8 | UniRef cluster | --------------------------MNSMFRFWIPKTSISMQLRMLSTQTRQALEQAVKEDPIVLFMKGTPTRPMCGFSLKAIQILSLENVASDKLVTYNVLS----NDELREGIKEFSDWPTIPQLYINGEFVGGSDILASMHKSGELHKILKEINALAPEQPKDSEEETTKKD---------------- | atgaattcgatgtttagattttggattcctaaaaccagtatatccatgcaacttcgcatg ctgtccacacaaactcgtcaagctcttgaacaggccgtcaaagaggatcctatcgtttta ttcatgaaaggtacacctacccgcccaatgtgtggattttcattaaaggcgattcaaatt ctttcattagaaaacgttgcttccgataaattagttacgtacaacgttttgagtaatgat gagctacgtgaaggtataaaggagttcagcgactggcctacgattcctcaactttatatt aacggagaattcgtgggcggtagtgatatcttggcgtccatgcataaatcaggagagctt cacaaaattctaaaagaaataaatgctttggcccctgaacagccaaaggatagtgaagag gaaactactaaaaaggattga |
| P51384 | UniRef cluster | ----------------------------------------------MDIETKKVIEQILDNNKIVLFMKGSKLMPMCGFSNTAIQILN---TLNTDYFTYDILE----NENIRQAIKEHSSWPTIPQLYINREFVGGADIMLELFEQGELQAQVETLLAA-------------------------------- | atggatatagaaactaagaaagtaatagagcaaatactggataataataaaatagtatta tttatgaaaggtagtaaattgatgccaatgtgtggtttctctaatacagctattcaaatc ttaaatactcttaatactgactattttacgtacgatattttagaaaatgaaaatattcgt caagctataaaagaacactctagttggccgacaattccgcaattgtatattaatagagaa tttgtcggtggagccgatattatgcttgaactttttgaacaaggtgaattacaagcacaa gtagaaactttgctagctgcataa |
| A8XE02 | UniRef cluster | -----------------MLRLATRQATAMLRSS---YSTASGAAGGLSDETRQRIDGIVKKDDVVVFMKGTQQEPACGFSRNVKLVLD---FHNVKFRDYNVLT----DAELREGVKIYSEWPTIPQVYVKGEFVGGCDILVAMHKDGEISDFLDEKGIPNKYGSK-------------------------- | atgctgcgtctagcgactcggcaagccaccgcgatgctccgttcgagctattcaacggct tctggagccgccggaggtctcagcgatgagacacgtcaaagaatcgatggaatcgtcaag aaggatgatgtcgtcgtttttatgaagggtacacaacaagaaccagcgtgcggattctcg agaaatgtcaaattggttctggacttccacaacgtgaaattccgtgactacaacgtgctg acggacgcagaattgcgagaaggcgtcaaaatctacagtgaatggccgacgatcccgcag gtctacgtgaaaggagagttcgttggcggatgtgatattctcgttgcgatgcataaggat ggcgagatttccgactttttggatgagaagggcatcccgaataagtatggatccaagtag |
| A8JH05 | UniRef cluster | -----MASLQCNMRPATTILTRRPAQVARKSIGVARSQTVRVTASGMAPDLKKSIDELIASNKVVVFMKGTRQFPMCGFSNTVVQILN---VMDVPYQTVNILE----DDAIRSGMKEYSQWPTFPQVYINGDFFGGCDIMMEAYQSGELKEQLEIALNS-------------------------------- | atggcctcgcttcagtgcaatatgcgtccggctaccaccattttgacgcggcgcccggct caggttgcgcgcaaatctattggcgtagcgcgaagccagactgtgcgcgtcacggcttct ggaatggcccccgatttgaagaagtccattgacgagcttattgcatccaacaaagtggtg gttttcatgaagggcacccgccagttcccaatgtgtggcttctccaacaccgtggtgcag atcctgaacgtgatggatgtgccgtaccagaccgttaacattctggaggacgacgccatc cgcagcggcatgaaggagtacagccaatggcccaccttcccgcaggtttatattaacggc gacttcttcggcggttgcgacattatgatggaggcctaccagtccggcgagctgaaggag cagctggagattgccctcaactcctag |
| A5DTY7 | UniRef cluster | -------------------------------------------------------------------MKGTPEFPQCGFSRATIQTLGQQGVDPAKFAAYNVLE----DAELRDGIKEFSSWPTIPQLYVNGEFVGGCDIVMSMAQSGELAEFLEKEGALIPEENDDVQSAEVKPRRD-------------- | atgaaaggaacaccagagttcccgcaatgtggattttctcgtgctacgatccaaacattg ggccaacagggtgtcgatccagccaagtttgctgcttacaacgttttggaggatgctgaa ttacgtgatggaattaaagagtttagctcctggcccacgattccacaattatacgtgaat ggtgaatttgttggtgggtgtgatattgtgatgtcaatggctcaaagtggcgagttagcc gagtttttggaaaaagaaggtgcattaattccagaggaaaatgatgatgtgcaaagcgcc gaagttaaacctagacgcgattag |
| A1CGQ3 | UniRef cluster | -------------------------------------------------------------------MKGTPETPQCGFSRASIQILGLQGVDPKKFVAFNVLG----DPELRQGIKEYSDWPTIPQLYLDKEFVGGCDILMSMHQNGELAKLLEEKGVLVAAD---------------------------- | atgaagggtacaccggaaactccacagtgcggtttctctcgagctagcatacagatcctg ggcctccagggcgtagatcccaaaaaatttgttgctttcaacgttttgggggatccagaa ctgcgtcagggtatcaaagaatattccgattggccgacaatacctcaactttacttggac aaggaatttgttgggggttgtgatatcctgatgtctatgcatcagaatggagagcttgcg aagctacttgaagagaaaggcgttttagtggcagcggattag |
| A0BR34 | UniRef cluster | ---------MASPSQLNNNHDHNHNHDHSDQDVQPQQKAQPQSMQEQIQQKHKEIDNIIKSNQVVLFMKGTPAQPRCGFSYYAVQILEFY--QVQNYHSVDVLP----DDLMRQEIKIYSNWSTFPQLYVKQELLGGTENIMKMHKDGRLKELFNTI----------------------------------- | atggcttcaccctcttagcttaacaataatcacgatcacaatcacaatcacgatcattct gatcaagacgtttagccataacaaaaggcttagccataaagcatgcaagaatagatctaa tagaaacataaggaaatagataatatcataaaatcaaactaagttgtgttattcatgaag ggaactccagcccaaccaaggtgtggattttcgtattatgctgtttagattcttgaattt tactaagtctaaaattatcattctgttgatgtccttcctgacgatttgatgagataagaa atcaagatatattcaaattggtcaacattcccataattgtatgttaaataagagttactt ggtggaaccgaaaacataatgaaaatgcacaaagacggcagattaaaagaattatttaat acaatatga |
| A8Q1F7 | UniRef cluster | --------------------------------------------MGVQVISSYDQFKQVTGGDKVVVIDFWATWCGPCKMIGPIFEKISETPAGEKIGFYKVDVDEQSQISSEVGIRAMPSFVFFKNGEKVETVVGADPSKLQVCIQSTRAWLWEETAPHRHGQD--------------------------- | atgggtgtgcaagtgatttcctcctacgaccagttcaagcaggtgaccggcggtgacaag gtcgttgtgatcgacttttgggctacatggtgtggtccctgcaagatgatcggcccgatc tttgagaagatctccgagacgcctgctggcgagaagatcggcttctacaaggtcgatgtc gatgagcagagccagatctcgtcggaggtcggtatccgtgctatgcccagctttgtcttt ttcaagaacggcgaaaaggtcgagacggtcgttggcgctgacccatcgaagctccaggta tgtatacaaagcacgcgagcgtggttgtgggaagagacagcgccgcaccggcacggacag gactga |
| Q4Z071 | UniRef cluster | -------------------------------------MEQKEFNSELSQNNIDLIENILKKHKLVLFMKGTALNPFCKYSKLAINILKLN--KAKEIYTVNILN----DDMLKHSLKIYSNWPTFPQLYINGKFVGGIDKIQELHDNKKLQEMLQTM----------------------------------- | atggaacaaaaggaatttaatagtgaattaagtcaaaataatattgacttaattgaaaat atattaaaaaaacataaactagttttatttatgaaaggaacagctttaaatccattctgt aaatatagcaaattagcaataaatattttaaaattaaataaagcaaaagaaatatatact gtaaatattctaaatgacgatatgttaaaacattctttaaaaatatattctaattggcct acatttcctcaattatatataaatgggaaatttgtaggaggaatagacaaaatacaggaa ttacatgataataaaaaacttcaagaaatgttacaaactatgtaa |
| Q4S0R6 | UniRef cluster | ------------------------------------------MVVIVIESEAQFDDYIKNIEGKLVVVDFTAQWCGPCKHIGPVFKSLSDMGDNKNVIFLKVDVDELEDLAARCKVSAMPTFLFFKDGVKIDEVIGANQPQLVEKIQKHKP----------------------------------------- | atggtggtgatcgttatcgagtcagaggcccaatttgatgactacatcaaaaacattgaa ggcaagctggtggtggtggacttcacagcccagtggtgtggcccctgtaaacacatcgga ccagtgtttaagagcctttcggacatgggcgacaacaagaacgtgatcttcctgaaggtg gacgtcgatgagttagaggacttggctgccaggtgcaaagtttctgccatgcccacgttc ctgtttttcaaagacggagtgaaaatagacgaagtaattggagctaatcaaccccaactg gtggagaaaatccagaaacacaaaccatga |
| A9P0Z6 | UniRef cluster | ----MDEHIESAGEVPPGNESWNITWMEKERPVKADLRLKPDRYMPFSLPLEQLVDKLFNENEVLVFIKGSRTDPKCPQSCRLLTILYEQMVDYETIDVFDEVYN----RGVRKALKVYSDWPTFPQVFVRGNLIGGADELDKMADKGELYELFKK------------------------------------ | atggatgagcacatagagtctgcaggtgaggtaccccctgggaatgaatcttggaacatc acatggatggagaaggagaggccagttaaggctgatcttcggttaaaacctgatcgttac atgccattcagcttacctttggaacaattggtagacaaattgttcaatgaaaatgaggtg ttagttttcattaaaggttcaagaacagatccaaaatgcccacaatcttgtagactattg acaatattgtatgagcaaatggtggattatgaaacaattgacgtgtttgatgaagtgtac aatcgtggtgtgagaaaggctttaaaggtatatagtgattggcctactttcccgcaggtc tttgtgcgtggaaatctcattgggggtgctgatgaattagataagatggcagataaaggg gagttgtatgaactctttaagaagtag |
| Q9FRT3 | UniRef cluster | ------------------------MAAASATAQAEGTVIAIHSLDEWTIQIEEANSAKKL-----VVIDFTASWCGPCRIIAPVFADLAK-KHT-NAVFLKVDVDELKPIAEQFSVEAMPTFLFMKEGDVKDRVVGAMKDELASKLELHMAM---------------------------------------- | atggcggcggcctcagcgacggcgcaggcggagggaacggtgatcgcgatccacagcctc gacgagtggaccatccagatcgaggaggccaacagcgccaagaagctggttgtgattgac ttcactgcatcatggtgcggaccatgccgcatcattgctccagtttttgccgatctagca aagaagcacacaaatgctgtttttctgaaggttgacgtcgatgaactgaagcctattgct gagcaattcagtgttgaggctatgccaacattcctgtttatgaaggagggagatgttaaa gacagggttgtcggtgctatgaaggatgaactggcgagcaagcttgagctacatatggcc atgtag |
| Q6L4X5 | UniRef cluster | ------------------------MAAASAAAQAEGTVIAIHSLDEWTIQIEEANSAKKL-----VVIDFTASWCGPCRIIAPVFADLAK-KHT-NAVFLKVDVDELKPIAEQFSVEAMPTFLFMKEGDVKDRVVGAMKDELASKLELHMAM---------------------------------------- | atggcggcggcctcagcggcggcgcaggcggagggaacggtgatcgcgatccacagcctc gacgagtggaccatccagatcgaggaggccaacagcgccaagaagctggttgtgattgac ttcactgcatcatggtgcggaccatgccgcatcattgctccagtttttgccgatctagca aagaagcacacaaatgctgtttttctgaaggttgacgtcgatgaactgaagcctattgct gagcaattcagtgttgaggctatgccaacattcctgtttatgaaggagggagatgttaaa gacagggttgtcggtgctatgaaggatgaactggcgagcaagcttgagctacatatggcc atgtag |
| Q5S1X7 | UniRef cluster | ------------------------------------------MVLQIVESKEDFEKKLEEAGDKLVVVDFFATWCGPCKMVEPFLKQQSE-ILKDVVIFLKVDVDENEEITQEYEIACMPTFLFIKNKTKLDEISGANEESIKEMLDKHK------------------------------------------ | atggtccttcaaatcgtcgagagcaaggaggacttcgaaaagaagctggaagaggccggc gacaagctygtggtggtggacttctttgccacgtggtgcggcccttgcaagatggtggag cccttcctgaagcaacagtccgaaattctcaaggatgttgtcatcttcctcaaggttgat gtggacgagaatgaagagattacgcaggagtacgagattgcgtgcatgccaaccttcctc ttcatcaagaacaaaaccaagttggacgaaatctccggagccaacgaagagtccatcaag gaaatgttggacaagcacaagtaa |
| Q6PV94 | UniRef cluster | ---------MGAILSALTGGAATAATSSPESS--ASRVQSFHSSARWQLHFNELKETNKL-----VVIDFSASWCGPCKFIEPAIHAMSE-KFT-DVDFVKIDVDELPDVAKEFNVEAMPTFVLCKKGKEVDKVVGAKKDELEKKIEKHRSQS--------------------------------------- | atgggcgctatactttctgccttaactggcggtgccgcaacggcggcgacgtcttcgccg gagagttctgcttctagggtccagtctttccactcttcggcgcgctggcagctccacttc aacgagctcaaagaaaccaataagctcgttgtgatagatttctcggcgtcgtggtgcggc ccttgcaaattcatagagccagcgattcacgccatgtccgaaaagttcaccgacgttgac ttcgtcaagatcgacgtcgacgaattgccggatgtggcgaaggagtttaatgtggaggcg atgccgacgttcgtgttgtgtaagaaagggaaggaagtcgacaaggttgtgggcgcaaag aaggacgagctcgaaaagaagattgagaaacatcgatcgcaatcttag |
| Q25549 | UniRef cluster | ----------------------------------------------------MDEFNEALKHDKLVVADFTASWCGPCQYISPIFAAMS--TQYEDVKFLKIDVDECQDIALEYGIEAMPTFQFFKNGTKVDEVQGADPDSLEQLVKKYIQQ---------------------------------------- | atggatgagtttaacgaggccttgaaacatgacaagttagttgttgcagactttactgca tcctggtgtggaccttgccaatatatttcaccaatatttgcagccatgtctactcaatat gaagatgtcaagtttttaaagattgatgtggatgaatgtcaagacattgctttggagtat ggtattgaagcaatgcctactttccaattctttaagaacggcaccaaagtcgatgaggtt caaggagcggatccagacagcctcgaacaactcgttaaaaagtacattcaacaatag |
| A5E6L2 | UniRef cluster | --------------------------------------------MITNITSKALFQGALS-FKGLVVVDFFATWCGPCKMISPLLEKFSN--EYKQVKFLKVDVDQSPEIALAYQVSSMPTFLFFKNGEQIHKVVGANPSALKQAIDSNA------------------------------------------ | atgatcaccaatatcacatcaaaagcactcttccaaggagccctttctttcaaaggattg gttgttgttgatttctttgcaacttggtgtggtccatgcaagatgatctcacctctcttg gaaaaattcagcaatgaatacaaacaggtgaaattcttaaaggttgacgttgaccaatcg ccagagatagcactggcctaccaagtgagtagtatgcctacttttcttttcttcaagaat ggagaacaaatacataaggttgtcggcgcaaacccttctgcattgaaacaagcaattgac tccaacgcgtga |
| Q6DGI6 | UniRef cluster | -------------------------------------------MVLEIEDKAAFDNALKNAGDKLVVVDFTATWCGPCQTIGPYFKLLSEKPENKNVVFLKVDVDDAQDVAALCGISCMPTFHFYKNGKKVDEFSGSNQSKLEEKINSHK------------------------------------------ | atggttctcgaaattgaagataaggctgccttcgacaacgccctaaaaaacgccggggac aagttggtggtggtggacttcacggccacatggtgcgggccctgccagaccatcgggccg tactttaaattgctgtctgaaaaaccagagaacaaaaatgtggtgtttctaaaggtggat gtggatgatgcacaggatgtggccgctttatgtggcatctcatgcatgccaacattccat ttctacaagaatggaaagaaggttgatgaattttctggatctaaccaatccaagctggag gagaagatcaactcgcataaatga |
| Q6FVN1 | UniRef cluster | ------MLLFRTARCQVPTLGRFATHPRIAPSIAMRFQSSGGYASIKQVKTLEEVQKLMK-DSNLSVIDFYATWCGPCKAMVPFLSKFVD--QYKDVKFYKVDVDESPDVAEYYGVSAMPTFVFTKDDDILHKIRGANPKGLAKAIEEFK------------------------------------------ | atgcttcttttccgtaccgctagatgccaagtaccaacattaggccgttttgccacacac ccaagaattgccccatcgattgcgatgaggttccagtccagtggcggatatgcttctatt aagcaggtcaagacgctagaagaagtccagaagctgatgaaggactctaacttgtcggtc atcgacttctacgccacctggtgtggtccatgtaaggctatggtgccattcttgtccaag tttgtggaccagtacaaagacgtcaagttctacaaggtcgacgtcgatgaaagtcccgat gttgctgaatactatggtgtatcggcaatgccaacttttgtgttcactaaggatgatgac atcttgcataagataagaggtgcaaaccctaaagggttggccaaagccatcgaagagttt aaatga |
| Q9LDX4 | UniRef cluster | --------------------MAASAATATAAAVGAGEVISVHSLEQWTMQIEEANAAKKL-----VVIDFTASWCGPCRIMAPIFADLAK-KFP-AAVFLKVDVDELKSIAEQFSVEAMPTFLFMKEGDVKDRVVGAIKEELTNKVGLHAAQ---------------------------------------- | atggcggcgtcggcggcgacggcaacggcggcggcggtgggggcgggggaggtgatctcc gtccacagcctggagcagtggaccatgcagatcgaggaggccaacgccgccaagaagctg gtggtcattgacttcactgcatcgtggtgtggaccctgccgcattatggctccaattttc gctgatctcgccaagaagttcccagctgctgttttcctcaaggtcgatgttgatgaactg aagtccattgctgagcaattcagcgtggaggccatgccaaccttcctgttcatgaaggaa ggagacgtcaaggacagggttgtcggagctatcaaggaggaactgacgaacaaggttggg ctacacgcggcccagtaa |
| A2I3V9 | UniRef cluster | -------------------------------------------MVYHIKDASDLEQQLSEAGSKLVVIDFFAAWCGPCKFISPKLEELST-VETD-VVFLKIDVDECEDLAEAYEISSMPTFIFIKNKKKVDSFSGANADKLKEIVDKLKSA---------------------------------------- | atggtttaccacattaaagacgccagtgatctggagcagcaattgagcgaagccggcagc aaactggttgtaatcgatttctttgccgcctggtgtgggccgtgtaaatttattagtccg aaattagaggagttgtccactgttgaaacggatgttgtatttttgaagattgatgtagac gagtgtgaagatcttgccgaagcttacgaaatcagttcgatgccaacgttcatttttatt aaaaacaagaaaaaggtggacagtttttcaggagcaaacgcggacaaacttaaagaaata gttgataagttgaaaagtgcgtaa |
| Q9XIF4 | UniRef cluster | ----MGSNVSSVHDVHSSMEITSNG----------------FVVEIESRRQWKSLFDSMKGSNKLLVIDFTAVWCGPCKAMEPRVREIAS--KYSEAVFARVDVDRLMDVAGTYRAITLPAFVFVKRGEEIDRVVGAKPDELVKKIEQHRV----------------------------------------- | atgggttccaatgtttcatctgtgcatgatgttcattcatcaatggaaattaccagtaat ggatttgttgtggaaatcgaatcaagaagacaatggaaatctctctttgattccatgaaa ggctcaaacaaattgctagtaattgatttcacagctgtatggtgtggaccttgtaaagca atggaacctagagttagggagattgcttcaaagtactcagaagctgtgtttgctagggtt gatgtggataggctaatggatgtggctgggacatatagagctattacacttccagctttt gtttttgtgaagagaggagaagagattgatagggttgttggagccaaacctgatgagctc gtgaagaagattgaacaacatagggtttaa |
| O64394 | UniRef cluster | ------------------MAASAATATATAAAVGAGEVISVHSLEQWTMQIEEANAAKKL-----VVIDFTASWCGPCRIMAPIFADLAK-KFP-AAVFLKVDVDELKPIAEQFSVEAMPTFLFMKEGDVKDRVVGAIKEELTTKVGLHAAQ---------------------------------------- | atggcggcgtcggcggcgacggcgacggcgacggcggcggcggtaggggcgggggaggtg atctccgtccacagcctggagcagtggaccatgcagatcgaggaggccaacgccgccaag aagctggtggtgattgacttcactgcatcatggtgcggaccatgccgcattatggctcca attttcgctgatctcgccaagaagttcccagctgctgttttcctcaaggtcgacgttgat gaactgaagcccattgctgagcaattcagcgtggaggccatgccaaccttcctgttcatg aaggaaggagatgtcaaggacagggttgtcggagctatcaaggaggaactgacgaccaag gttgggctacacgcggcccagtaa |
| Q42403 | UniRef cluster | -------------------------------MAAEGEVIACHTVEDWTEKLKAANESKKL-----IVIDFTATWCPPCRFIAPVFADLAK-KHL-DVVFFKVDVDELNTVAEEFKVQAMPTFIFMKEGEIKETVVGAAKEEIIANLEKHKTVVAAA------------------------------------ | atggccgcagaaggagaagttatcgcttgccacaccgttgaagattggaccgagaagctc aaagccgccaacgaatccaagaaactgattgtgatagacttcactgcaacatggtgccca ccttgccgtttcattgcacccgtctttgctgacttagccaagaagcacctcgacgtagtc ttcttcaaggtcgatgttgacgaattgaacactgttgctgaggagtttaaagttcaggca atgccaacgtttatcttcatgaaagaaggagagatcaaggagactgtggttggtgctgct aaagaagaaatcattgccaatctcgagaagcacaagacagttgttgctgctgcttga |
| Q7XZK2 | UniRef cluster | ------------------------MAASATAAAVAAEVISVHSLEQWTMQIEEANTAKKL-----VVIDFTASWCGPCRIMAPVFADLAK-KFP-NAVFLKVDVDELKPIAEQFSVEAMPTFLFMKEGDVKDRVVGAIKEELTAKVGLHAAAQ--------------------------------------- | atggcggcgtcggcaacggcggcggcagtggcggcggaggtgatctcggtccacagcctg gagcagtggaccatgcagatcgaggaggccaacaccgccaagaagctggtggtgattgac ttcactgcatcatggtgcggaccatgccgcatcatggctccagttttcgctgatctcgcc aagaagttcccaaatgctgttttcctcaaggtcgacgtggatgaactgaagcccattgct gagcaattcagtgtcgaggccatgccaacgttcctgttcatgaaggaaggagacgtcaag gacagggttgtcggagctatcaaggaggaactgaccgccaaggttgggcttcacgcggcg gcccagtaa |
| Q7FT21 | UniRef cluster | ---------------MAAAATATTTAAATAAAVGPGEVISVHSLEQWTMQIEEANAAKKL-----VVIDFTASWCGPCRIMAPIFADLAK-KFP-AAVFLKVDVDELKPIAEQFSVEAMPTFLFMKEGDVKDRVVGAIKEELTTKVGLHAAA---------------------------------------- | atggcggcggcggcgacggcgacgactacagcggcggcgacggcggcggcggtggggccg ggggaggtgatctccgtccacagcctggagcagtggaccatgcagatcgaggaggccaac gccgccaagaagctggtggtgattgacttcactgcatcatggtgcggaccatgccgcatc atggctccaatttttgctgatctcgccaagaagttcccagctgctgttttcctcaaggtc gacgttgatgaactgaagcccattgctgagcaattcagcgtcgaggccatgccaaccttc ctgttcatgaaggaaggagacgtcaaggacagggttgtcggagctatcaaggaggagctg acgaccaaggttgggctccacgcggctgcctag |
| O64395 | UniRef cluster | ---------------MAAAATATTTAAATAAAVGPGEVISVHSLEQWTMQIEEANAAKKL-----VVIDFTASWCGPCRIMAPIFADLAK-KFP-AAVFLKVDVDELKPIAEQFSVEAMPTFLFMKEGDVKDRVVGAIKEELTTKVGLHAAA---------------------------------------- | atggcggcggcggcgacggcgacgactacagcggcggcgacggcggcggcggtggggccg ggggaggtgatctccgtccacagcctggagcagtggaccatgcagatcgaggaggccaac gccgccaagaagctggtggtgattgacttcactgcatcatggtgcggaccatgccgcatc atggctccaatttttgctgatctcgccaagaagttcccagctgctgttttcctcaaggtc gacgttgatgaactgaagcccattgctgagcaattcagcgtcgaggccatgccaaccttc ctgttcatgaaggaaggagacgtcaaggacagggttgtcggagctatcaaggaggagctg acgaccaaggttgggctccacgcggctgcctag |
| Q38879 | UniRef cluster | ---------MGGALSTVFGSGE-DATAAGTES-EPSRVLKFSSSARWQLHFNEIKESNKL-----LVVDFSASWCGPCRMIEPAIHAMAD-KFN-DVDFVKLDVDELPDVAKEFNVTAMPTFVLVKRGKEIERIIGAKKDELEKKVSKLRA----------------------------------------- | atgggaggagctttatcaactgtgtttggaagtggagaagatgcaacagctgcagggacc gaatctgagccaagtcgcgtcctaaagtttagctcttccgctcggtggcaacttcatttc aacgagatcaaagaatcaaacaaactgctggtggttgatttctcggcctcatggtgcgga ccatgtaggatgattgagcctgcgattcatgccatggctgataagttcaatgatgttgat ttcgtcaaattagatgttgatgaacttcctgatgtggctaaagagtttaatgtgacggca atgcctacctttgtgctggtgaaaaggggtaaagaaattgaaagaatcattggtgccaaa aaggacgaacttgagaagaaagttagcaaactcagagcataa |
| Q8L9A6 | UniRef cluster | ---------MGGALSTVFGSGE-DAAAAGTESSEPSRVLKFSSSARWQLHFNEIKESNKL-----LVVDFSASWCGPCRMIEPAIHAMAD-KFN-DVDFVKLDVDELPDVAKEFNVTAMPTFVLVKRGKEIERIIGAKKDELEKKVSKLRA----------------------------------------- | atgggaggagctttatcaactgtgtttggaagtggagaagatgcagcagctgcagggact gagtcttccgagccaagtcgcgtcctcaagtttagctcttccgctcggtggcaacttcat ttcaacgagatcaaagaatcaaacaaactgctggtggttgatttctcggcttcatggtgt ggaccatgtaggatgattgagcctgcgatccatgccatggctgataagttcaatgatgtt gatttcgtcaaattagatgttgatgaacttcctgatgtggctaaagagtttaatgtgacg gcaatgcctacctttgtgctggtgaaaaggggtaaagaaattgaaaggatcattggtgcc aaaaaggacgaacttgagaagaaagttagcaaactcagagcataa |
| A9NQW4 | UniRef cluster | -----------MGNCFSEKTTWIEHPGNQQMAGSHGNVRIITANQEWEAEILEANTTGKI-----VVVDFTAAWCGPCKMITPFYSELSE-KYP-QLVFLKVDVEEMPELSATWDVQAMPTFFFIKDGKQIDKLVGANKPELEKKVISYATASQ-------------------------------------- | atgggcaactgtttcagcgaaaagactacatggattgaacatcctggtaatcaacagatg gcgggctctcatggaaatgtacgcattatcactgccaatcaagagtgggaggcagaaata ctcgaggcaaacaccactggaaaaattgtagttgtagatttcactgcagcatggtgtggt ccctgcaaaatgatcactcctttctatagtgagctttctgagaaatatccacagctggtc ttcctcaaagtggatgttgaagaaatgcctgagctgagtgcaacgtgggatgttcaagca atgccgactttttttttcataaaagatgggaaacagattgataagttggtgggagctaat aaacctgagttggagaagaaagtcatcagctatgctactgcatctcagtga |
| Q9DGI3 | UniRef cluster | -------------------------------------------MVVHIENLNAFSAALKNAGDKLVVVDFTATWCGPCQKIGPIFETLSKSEDYQNVVFLKVDVDDAADVSSHCDIKCMPTFHFYKNGQKIDEFSGANEQTLKQKINDHK------------------------------------------ | atggttgtacatatcgagaacttgaatgctttcagtgccgccctgaaaaatgcaggggac aaattagtggtggtggacttcacagccacatggtgcggcccctgccaaaagattggcccc atctttgaaactctgtccaagagtgaagactatcagaatgtggtttttctgaaggtggat gtggacgatgcagcggatgtatccagtcactgtgatatcaaatgcatgccaactttccac ttctacaagaatgggcaaaagattgatgagttttctggggccaatgaacaaacactgaaa cagaagatcaacgatcataaatga |
| Q6GQ64 | UniRef cluster | -------------------------------------------MVRHIENLEEFQLVLKEAGGKLVVVDFTATWCGPCKMIAPVFEKLS--VDNPDAVFLKVDVDDAQDVAAHCDVKCMPTFQFYKNGIKVDEFSGANQSSLIQKVEALK------------------------------------------ | atggttcgacacatcgagaatttggaagagttccaattggtactgaaagaagctgggggg aaacttgtagttgttgatttcacagcaacatggtgtggtccatgcaaaatgattgcgcct gtctttgagaaactaagtgtagataatccagatgctgttttcctgaaagtggatgttgat gatgcacaagatgttgcggcccactgtgatgtcaaatgcatgccgacctttcaattctat aaaaatggaataaaggtagatgaattcagtggagcgaatcaatctagccttattcagaaa gtggaggcgctgaaataa |
| Q4W1F6 | UniRef cluster | ------------------------------MASEQGVVIACHSKAEFDAHMTKAQEAGKL-----VVIDFTAAWCGPCRAIAPLFVEHAK-KFT-QVVFLKVDVDEVKEVTAAYEVEAMPTFHFVKNGKTVATIVGAKKDELLALIEKHAAPAPASASA--------------------------------- | atggcgtccgagcagggagtcgtgatcgcgtgccacagcaaggctgagtttgacgcccac atgaccaaggcccaggaagccggcaagctggtggtcatcgacttcactgccgcctggtgc ggtccatgccgcgccatcgccccactgttcgtcgaacacgccaagaagttcactcaggtc gtgttcctgaaggtggacgtggacgaagtgaaggaagtcaccgcggcctacgaggtcgag gcgatgccgaccttccacttcgtcaagaacggcaagacggtcgcgaccatcgtgggtgcc aagaaggacgagctcctggccctgatcgagaagcatgccgcgcctgcgcctgcgtctgcg tctgcctaa |
| A2Y6C8 | UniRef cluster | ------------------------MAAASAAAQAEGTVIAIHSLDEWTIQIEEANSAKKLTCSPKVVIDFTASWCGPCRIIAPVFADLAK-KHT-NAVFLKVDVDELKPIAEQFSVEAMPTFLFMKEGDVKDRVVGAMKDELASKLELHMAM---------------------------------------- | atggcggcggcctcagcggcggcgcaggcggagggaacggtgatcgcgatccacagcctc gacgagtggaccatccagatcgaggaggccaacagcgccaagaagctgacttgcagtcct aaggttgtgattgacttcactgcatcatggtgcggaccatgccgcatcattgctccagtt tttgccgatctagcaaagaagcacacaaatgctgtttttctgaaggttgacgtcgatgaa ctgaagcctattgctgagcaattcagtgttgaggctatgccaacattcctgtttatgaag gagggagatgttaaagacagggttgtcggtgctatgaaggatgaactggcgagcaagctt gagctacatatggccatgtag |
| A5E6L1 | UniRef cluster | --------------------------------------------MVSQVSSAKEFEDTIKSFQGLVVVDFFATWCGPCKMIAPLLEKFST--QYTEVKFLKVDVDAVQELAQQYQVTSMPTLLFFKNGEVIEKVIGANPSAIKKAIETNK------------------------------------------ | atggtttcacaagtcagcagtgctaaagaattcgaagacaccattaagagtttccaagga ttggttgtggttgactttttcgccacttggtgtggtccatgtaagatgattgctccattg ttggagaaattctcaacccaatacactgaagtcaaattcctcaaggttgatgttgatgcc gtacaggaattggctcaacaataccaagttacaagtatgccaaccttgttgttcttcaag aacggtgaagttattgaaaaagttattggtgccaacccatcagctatcaagaaagccatt gaaacaaacaagtaa |
| A1DAE3 | UniRef cluster | --------------------------------------------MPVTAITSFKQFKELIDGDKPVIIDFWATWCGPCRAISPIFEQLSDNPDFGDVGFYKVDVDEQEQISQEVGIRAMPTFVLFKNGDKVGELMGAVPQRLQALLDTARSLA--------------------------------------- | atgcctgttaccgccatcacctcgttcaaacagttcaaagaactcattgacggtgataag cctgttataatcgacttctgggcaacatggtgtggaccttgccgagctatttcacccatc ttcgagcagctgtcggataatcccgactttggcgatgtcggattctacaaagttgatgtc gacgaacaagagcagatttctcaagaggtcggcatccgtgcaatgccaacctttgttctc tttaaaaacggggacaaggttggtgaacttatgggagccgttccacaacgtctgcaagcc cttcttgacacggcacgctctctagcatga |
| Q8GUR9 | UniRef cluster | -------------------------------MAEEGQVIGVHTVDAWKEQLEKGKASKKL-----IVVDFTASWCGPCRFIAPILAEIAK-KLT-HVTFLKVDVDELKTVSEEWGIEAMPTFLFLKDGELVDKVVGAKKEELQLKIDKHAA----------------------------------------- | atggcggaagagggacaagtgatcggtgttcacaccgtggatgcttggaaggaacagtta gagaagggaaaagcctcaaagaaactgattgtagttgatttcactgcttcttggtgcggt ccatgccgttttattgccccaattttggcagagattgctaaaaagcttacacatgtcact ttccttaaggttgacgtggatgaattgaagactgtttccgaggagtggggaattgaagct atgccaacattcttgttcttgaaagatggtgaacttgtggacaaagttgtgggtgccaag aaggaggagctgcaattgaaaattgacaagcatgcagcttga |
| A9RQ57 | UniRef cluster | ------------------------------MAADHGNIHIVNNTVEWKTKLDEATSSGKI-----VVVDFTATWCGPCRMMAPIFADLSK-KFE-KLLFLKVDVDAVQEVTQEWEVRAMPTFLFIKDGKLLDKIVGANKDELEKKCNQYASQPVVATA---------------------------------- | atggctgccgaccacgggaacatccatattgtgaataatactgtagagtggaagactaag ttggacgaggcgacgagctctggaaaaattgttgtagtcgatttcactgcaacatggtgc ggcccttgccgtatgatggctccaattttcgctgacttgagcaagaagtttgagaagctg ctctttctgaaggtcgacgtcgacgcagttcaggaggtcactcaagagtgggaagttcgt gcaatgcccactttcttgttcatcaaggatgggaaactgctcgacaagattgttggtgcc aacaaggacgagctcgagaagaagtgcaaccaatacgctagccagcctgttgttgccact gcctga |
| A9VBR7 | UniRef cluster | -----------------MASLLRRALPLTRMSPATSALRQLRLSSHSIQVNNDNFDETIAEATKPVLVYFTASWCGPCRMLKPILEKAVDARNGD-VVLAKIDVDEQQELSARFQVASVPQVFAVKEGEIVDQFIGAQPLDKVNSFLDKIVKA--------------------------------------- | atggcttctttgctgcgccgcgcgcttcctctgacgcgcatgtctcctgcgacaagcgct ctgcgtcagcttcgtctgagctcgcactctattcaggtcaacaacgacaactttgatgag accattgccgaggccaccaagcctgtcctcgtctacttcacggccagttggtgcggtcct tgccgtatgctcaaacccattcttgagaaggctgtggatgcgcgcaacggagacgtggtc ttggccaaaattgacgtggacgagcagcaggaactctcggcccgcttccaggtggcctcg gtgccccaggtctttgccgtcaaggaaggcgagattgtggatcagtttatcggtgctcag cctttggacaaggtgaattcgtttttggataagattgtcaaggcctaa |
| Q8GZT3 | UniRef cluster | ---------MGGVLSSVLGGGGGDEPLAGNES--ESRVMKFSSSARWQLHFNEIKESSKL-----LVVDFSASWCGPCRMIEPAFIAMSA-KFS-DVEFLKLDVDELPDVAKEFNVTGMPTFVLVKNGKEIERIVGARKDELEKKVLKHRA----------------------------------------- | atgggaggtgtattatcatctgtgttaggaggtggtggtggagatgaaccactcgcgggg aatgaatccgaaagtcgcgtcatgaagtttagctcatcggctcggtggcagcttcacttc aacgagatcaaagaatcctcgaaactgctggtggttgacttctcagcatcctggtgtgga ccttgcaggatgatagagcctgcgttcatcgccatgtctgccaagttcagtgatgttgaa ttcctcaaattggatgtcgatgaacttcccgatgtggctaaagagttcaatgtgacgggg atgccaacttttgtgctggtgaaaaatggtaaagagattgaaaggatcgttggagcaagg aaagatgaacttgagaagaaagttctcaaacacagggcatag |
| Q84LP7 | UniRef cluster | -------------------------------MAEEGQVISCHTVESWNEQLQKGIAAKKL-----IVVDFTASWCPPCKLMSPILSELAK-KLP-AVIFLKVDVDELQSVAEEWAVEAMPTFVLTKDGKVLERIVGAKKDELQLAVEKHATTVENATTANA------------------------------- | atggccgaggagggacaagtgatcagctgccacactgttgaatcctggaacgagcagctc caaaagggcattgcggcaaagaaactgatagtggtggattttacggcttcatggtgcccg ccatgcaagttgatgtctccaattttgagtgaattggcgaagaagctgcccgctgtcata ttcttgaaggtggatgttgatgaattgcagtccgtcgctgaggaatgggctgtggaagcc atgccgacctttgtactgacgaaagacggcaaagtcctggagaggattgtgggagcgaag aaagatgagctgcagctggctgtcgaaaagcacgcaacaactgttgagaatgcaactact gctaatgcttga |
| A9SJH2 | UniRef cluster | --------------------------------MDHGKVHVINNSAAWDAKLAEATSTGKI-----VLVDFTATWCGPCRLMAPIFVELSK-KYE-NIIFLKVDVDEVKDVTSQWEVRAMPTFIFIKDGKSIHKVVGANKDELEKKCQQFASLPSTV------------------------------------ | atggatcatggaaaggttcatgttattaacaactcggcagcctgggacgccaagttggca gaggccacgagcactggaaagatagttctcgtcgattttaccgccacttggtgcggacct tgccggctgatggcacctattttcgttgaacttagcaagaagtatgaaaacatcatattt ttgaaggtggacgtcgacgaagttaaggacgtgacatctcagtgggaagtgcgcgctatg cccacattcatatttatcaaagacggaaagagtattcacaaggtagttggtgcaaacaag gatgagctcgagaagaagtgtcaacaattcgctagcctccccagcacagtctga |
| A7Q2Q0 | UniRef cluster | ------------------------MGASHSSSSKSSRVLTFNSSASWKIHFEEAKSTGKL-----MVIDFSATWCGPCRFMEPVINEFAE-KYT-DVEFVKIDVDELSDVAQEFGVQGMPTFLLIKRGTEVDKVVGAKKEELQKKIEAHRKN---------------------------------------- | atgggagccagccactcgtcttcttcaaagtcatctcgtgtcctcaccttcaattcctca gcatcatggaagatacattttgaagaagccaaaagcactggcaaactgatggttatcgat ttctcggctacatggtgtggaccttgccgattcatggaacccgtcattaatgagtttgct gagaaatatacagacgtggagttcgtcaagattgatgtggacgagttgtcggatgtggct caagaatttggagtgcagggaatgccaacattcttactgatcaagagaggaacagaagtt gataaagtggtgggagccaagaaggaagaactccagaagaagatcgaggctcacaggaaa aattga |
| Q9UW02 | UniRef cluster | ---------------------------------------------MVQVISNLDEFNKLTNSGKIIIIDFWATWCGPCRVISPIFEKFSEKYGANNIVFAKVDVDTASDISEEAKIRAMPTFQVYKDGQKIDELVGANPTALESLVQKSLA----------------------------------------- | atggtccaagttatatccaacctcgatgagttcaacaagctcaccaacagcgggaagatc atcatcattgatttctgggctacatggtgtggtccttgccgtgtcattagtcccatcttc gagaagttctcagaaaaatacggtgccaataacatcgtcttcgcaaaggtggatgtagac actgcctccgatatctctgaggaagcaaaaatccgtgctatgcccactttccaggtttat aaggatggtcagaagatcgatgagctcgttggtgctaacccaactgcacttgagtccctt gtccagaagtctttagcgtga |
| Q9V429 | UniRef cluster | -----------------------------------MMILLRDSTNLHFHLQADLDGQLTKASGKLVVLDFFATWCGPCKMISPKLVELST-QFADNVVVLKVDVDECEDIAMEYNISSMPTFVFLKNGVKVEEFAGANAKRLEDVIKANI------------------------------------------ | atggtgtaccaggtgaaagataaggccgatctcgatggacagctgaccaaggcatccggc aagctggtggtgctggatttcttcgccacttggtgcggaccctgcaagatgatctcgccc aaactggttgaactctccacgcagttcgccgacaacgtcgttgtcctgaaggtcgatgtg gacgaatgcgaagacattgcaatggaatacaacatctccagcatgcccaccttcgtgttc ctcaagaacggcgtcaaggtcgaagagttcgccggagccaacgccaagcgtctggaggat gtcatcaaggccaatatctaa |
| Q6Z4I3 | UniRef cluster | ---------------MGGAFSTSKPKPAAGEEGGESAVVAVHSKAKWDELWDAHKNTTKL-----VVIDFSASWCGPCKMMEPVFKEMAG-RFT-DVAFLKVDVDELAEVARTWRVEAMPTFVLARGGEEVGRIVGADKDELEKTINTLRSSSSSTATTT-------------------------------- | atgggcggcgccttctcgacgtcgaagccgaagcccgccgccggcgaggagggcggcgag tccgccgtggtggccgtccactccaaggccaagtgggacgagctgtgggacgcccacaag aacaccacaaagctggtggtgatagacttctcggcgtcgtggtgtgggccgtgcaagatg atggagccggtgttcaaggagatggccggccgcttcaccgacgtcgccttcctcaaggtc gacgtcgacgagctcgcggaggtggcgcggacctggcgggtggaggcgatgccgacgttc gtgctggcgaggggcggcgaggaggtcggccgcatcgtcggcgccgacaaggacgagctc gagaagaccatcaacacgctcaggtcatcgtcgtcgtcgacggcgacgacgacgtga |
| A5BD80 | UniRef cluster | ---------MGSVVSGLLGGGAADASDSTLEG---SGVNVFHSSERWLLHFNASKESNQL-----MVIDFAATWCGPCKFMEPAVKSMAS-KYT-DVDFVKIDVDELPDVAQEFTVQAMPTFVLLKKGKELERVIGAKKDELEKKIQKHRAVSNA------------------------------------- | atgggatctgttgtgtcgggtttgttgggaggaggagctgcagacgcatcagattcgact ttggagggatctggagtcaatgtatttcactcatctgagagatggttgctccacttcaat gcttctaaagaatccaaccaattgatggtgattgacttcgctgcgacctggtgtgggcct tgcaaattcatggagcctgcagtgaagtccatggcttccaagtacactgacgtcgacttc gtcaagatcgatgttgatgagttgcctgatgtggcgcaggagtttactgtgcaggccatg ccgacgtttgtgttgttgaaaaaagggaaggaattggaaagggtgatcggggccaagaag gatgagctggagaagaagattcagaaacacagggcagtttccaatgcttaa |
| A2YJ11 | UniRef cluster | ---------------MGGAFSTSKPKPAAGEEGGESAVVAVHSKAKWDELWDAHKNTTKL-----VVIDFSASWCGPCKMMEPVFKEMAG-RFT-DVAFLKVDVDELAEVARTWRVEAMPTFVLARGGEEVGRIVGADKDELEKTINTLRSSSSSTATTT-------------------------------- | atgggcggcgccttctcgacgtcgaagccgaagcccgccgccggcgaggagggcggcgag tccgccgtggtggccgtccactccaaggccaagtgggacgagctgtgggacgcccacaag aacaccacaaagctggtggtgatagacttctcggcgtcgtggtgtgggccgtgcaagatg atggagccggtgttcaaggagatggccggccgcttcaccgacgtcgccttcctcaaggtc gacgtcgacgagctcgcggaggtggcgcggacctggcgggtggaggcgatgccgacgttc gtgctggcgaggggcggcgaggaggtcggccgcatcgtcggcgccgacaaggacgagctc gagaagaccatcaacacgctcaggtcatcgtcgtcgtcgacggcgacgacgacgtga |
| Q4WB53 | UniRef cluster | ------------------------------------MPVTEITSAKLSTHGRFEQFKELTDGDKPVVIDFWATWCGPCKAISPLFEKMSDNPEYGNVGFYKVDVDEQEQVSQEVGIRAMPTFVLFKNGYKIGEAVGAAPPRLEGLLNTARSL---------------------------------------- | atgcctgttaccgagatcacctccgctaaactttctacacacggcagattcgaacagttc aaagaactcactgacggtgacaagcctgttgtaatcgacttttgggcaacatggtgtggg ccgtgcaaggctatttcgccccttttcgagaagatgtcggataatcccgaatatggcaat gtcgggttctacaaagttgatgtcgatgagcaggagcaggtctctcaagaggtcggcatc cgtgcaatgccaacctttgttctctttaagaacgggtataagattggtgaagctgtggga gccgctccaccacgtctggaaggacttctcaacactgcacgctctctatag |
| B0YAQ9 | UniRef cluster | ------------------------------------MPVTEITSAKLSTHGRFEQFKELTDGDKPVVIDFWATWCGPCKAISPLFEKMSDNPEYGNVGFYKVDVDEQEQVSQEVGIRAMPTFVLFKNGYKIGEAVGAAPPRLEGLLNTARSL---------------------------------------- | atgcctgttaccgagatcacctccgctaaactttctacacacggcagattcgaacagttc aaagaactcactgacggtgacaagcctgttgtaatcgacttttgggcaacatggtgtggg ccgtgcaaggctatttcgccccttttcgagaagatgtcggataatcccgaatatggcaat gtcgggttctacaaagttgatgtcgatgagcaggagcaggtctctcaagaggtcggcatc cgtgcaatgccaacctttgttctctttaagaacgggtataagattggtgaagctgtggga gccgctccaccacgtctggaaggacttctcaacactgcacgctctctatag |
| A3GI91 | UniRef cluster | --------------------------------------------MVATVASKQEFTEALT-HDGLVVVDFFATWCGPCKMIAPLLEKFSK--EYTTAKFIKVDVDELGEIAQEYEVSSMPTIIFFKGGKIVTKVIGANPAALKQAIAANA------------------------------------------ | atggttgccactgttgctagcaaacaagaattcaccgaggctttgacccacgacggtttg gttgttgtcgacttctttgccacatggtgtggtccttgtaagatgattgctccattgttg gagaaattctccaaggaatacactaccgccaagttcatcaaggttgatgtcgatgaactc ggagaaattgcccaagaatacgaagtttcttcgatgccaactatcatcttcttcaagggt ggtaagatcgttaccaaggttattggtgctaacccagctgccttgaagcaagccattgct gccaacgcttaa |
| A8HTK0 | UniRef cluster | ----------------------------MAGSSEEGQVISCHTVEEWNDQLQKGNESKKL-----IVVDFTASWCGPCRFIAPFLAELAK-KFT-SVIFLKVDVDELKSVSQDWAIEAMPTFVFVKEGTLLDKVVGAKKDELQQKIQKHVASSNA------------------------------------- | atggcaggttcatcagaagagggacaagtcattagctgccacaccgttgaagaatggaac gatcaactccagaagggcaacgaatccaagaaactcattgttgtggattttactgcttct tggtgtggaccatgccgtttcattgcaccattcttggctgagctggctaagaagttcaca agtgtcatattcctaaaggtggatgtggacgaattaaagagtgtttctcaagattgggct attgaggctatgcccacttttgtgtttgtgaaagagggaacgcttctggacaaagtggtg ggagcaaagaaggatgagctgcagcagaaaatacagaaacatgtggcttcatctaatgct tag |
| A5BIE9 | UniRef cluster | ------------------------MGASHSSSSKSSRVLTFNSSASWKIHFEEAKSTGKL-----MVIDFSAXWCGPCRFMEPVINEFAE-KYT-DVEFVKIDVDELSDVAQEFGVQXMPTFLLIKRGTEVDKVVGAKKEELQKKIZAHRKN---------------------------------------- | atgggagccagccactcgtcttcttcaaagtcatctcgtgtcctcaccttcaattcctca gcatcatggaagatacattttgaagaagccaaaagcactggcaaactgatggttatcgat ttctcggctrcatggtgtggaccttgccgattcatggaacccgtcattaatgagtttgct gagaaatatacagacgtggagttcgtcaagattgatgtggacgagttgtcggatgtggct caagaatttggagtgcaggsaatgccaacattcttactgatcaagagaggaacagaagtt gataaagtggtgggagccaagaaggaagaactccagaagaagatcsaggctcacaggaaa aattga |
| A5AML5 | UniRef cluster | -------------------------------MAEEGQVVGCHSVESWKEQFQHGIESKKL-----VVVDFTASWCGPCRVISPFLAELAK-KMP-NVIFLKVDVDELETVAKEWEVEAMPTFLFLKEGNVVDKVVGAKREELVQKTEKHATA---------------------------------------- | atggcggaagagggacaagttgtcggttgccacagtgtggaatcgtggaaggagcaattc cagcatggaatagagtccaagaaactggtggtggtggatttcactgcttcatggtgtggg ccatgtcgtgtcattagcccatttctggcagagcttgccaagaagatgcctaatgtcata tttctcaaggtggatgtggatgaattggagactgttgctaaggagtgggaagtggaggcc atgccaacctttctgttcctgaaagaaggaaacgtagtggacaaggttgtgggtgcaaaa agagaagaactggtgcagaagacagagaagcatgcaactgcttga |
| A2Q2R1 | UniRef cluster | ----------MGSFLSSLVGGDSATASQSSESSENSSVKTFHSSARWQLHFNELKDSPRL-----VVIDFSATWCGPCKMMEPILQAMAN-EFT-DVEFIKIDVDELSDVAQEFKVQAMPTFLLLKNGKEVDKVVGAKKDELKNKVQKHKA----------------------------------------- | atgggatcatttctctcttccttagtcggcggcgactccgccaccgcctcacagtcatca gaatcatcggagaactcttccgtcaagactttccactcatctgctcgctggcaacttcac ttcaacgaactcaaagattctcctcgtcttgtggtgattgatttctctgctacatggtgt ggaccttgtaaaatgatggaaccgattcttcaagctatggctaatgaattcactgatgtt gaattcatcaaaattgatgttgatgaattatcggatgtggcgcaggagtttaaggtgcag gctatgccaacgtttctgttgttgaagaatgggaaagaggttgacaaggttgttggagca aagaaggatgaacttaaaaacaaggttcaaaagcacaaagcttag |
| Q8L907 | UniRef cluster | ------------------------------MAAEEGQVISCHTNDVWTVQLDKAKESNKL-----IVIDFTASWCPPCRMIAPIFNDLAK-KFMSSAIFFKVDVDELQSVAKEFGVEAMPTFVFIKAGEVVDKLVGANKEDLQAKIVKHTGVTTA------------------------------------- | atggcggcagaagagggtcaagtgattagttgtcacacgaacgatgtatggactgtgcaa cttgataaagccaaagaatccaacaagctgattgtgattgatttcactgcttcatggtgt ccaccatgccgcatgattgctccaattttcaacgatttggccaagaagttcatgtcaagt gccatcttcttcaaggtggatgttgatgaacttcagagtgttgctaaagagtttggtgtg gaggcaatgccaacctttgtgttcattaaagccggcgaagttgtggataagctcgttggt gcgaataaagaagatcttcaggcgaaaatagtgaagcatactggtgttacaactgcgtaa |
| Q6BJ80 | UniRef cluster | --------------------------------------------MVAAITSKQEFTDALS-HDGLVVVDFFATWCGPCKMIAPLLDKFSA--EYNQAKFIKVDVDEFGEIAQEYEVSSMPTVIFFKGGEVVNKVIGANPGALKQVISANA------------------------------------------ | atggttgctgctatcacttctaaacaagaatttaccgatgctttatctcacgatggatta gtcgttgttgactttttcgctacatggtgtggaccatgtaagatgattgctccattattg gacaagttctcagccgaatacaaccaagctaagtttataaaggttgacgttgatgaattt ggtgaaattgctcaagaatatgaagtcagttctatgccaactgttatcttcttcaaggga ggtgaagtcgtcaacaaggtcatcggtgccaacccaggtgctttgaagcaagtcatttcg gccaatgcttaa |
| Q39362 | UniRef cluster | ------------------------------MAAEEGQVIGCHEIDVWAVQLDTAKQSNKL-----IVIDFTASWCPPCRMIAPVFADLAK-KFMSSAIFFKVDVDELQNVAQEFGVEAMPTFVLIKDGNVVDKVVGARKEDLHATIAKHTGVATA------------------------------------- | atggctgcagaagagggacaagtgatcggttgccacgagattgatgtatgggccgtgcag ctcgacacagccaaacaatccaacaagctgattgtgatagatttcactgcttcatggtgc ccaccatgccgcatgattgctccagttttcgcagatctggccaagaagttcatgtcaagt gccatcttcttcaaggtggacgtcgatgaactgcagaatgtagctcaagagtttggtgtg gaggcaatgccgacctttgttctcatcaaagacggaaatgttgttgataaggtcgttggt gcaaggaaggaagatcttcatgccacaatagccaagcatactggtgttgctactgcttaa |
| A2VDE6 | UniRef cluster | -------------------------------------------MVRHVETLEEFQNVLQEAKEKLVVVDFTATWCGPCKMIAPVFEKLS--VENPDVVFLKVDVDDAQDVAAHCEVKCMPTFHFYKNGLKVFEFSGANESSLVQKVAELK------------------------------------------ | atggttcggcacgtggagactttggaagagttccaaaatgtattgcaagaagccaaggaa aaacttgtagtcgttgatttcacagcaacatggtgtggtccatgcaaaatgattgcccct gtctttgagaaactgagtgtagaaaatccagatgttgttttcctgaaagtggatgttgat gatgcacaagatgttgctgcccactgtgaggtcaaatgcatgccgacctttcatttctat aaaaatggactaaaggtatttgaattcagcggagccaacgaatctagtcttgttcagaaa gtggcagagctgaaataa |
| Q9CAS1 | UniRef cluster | ----MGANVSTPDQRFQVTHFRSTKPWTPRPEIYPFKVNSPCIVEIKNMNQWKSRLNALKDTNKLLVIEFTAKWCGPCKTLEPKLEELAA--KYTDVEFVKIDVDVLMSVWMEFNLSTLPAIVFMKRGREVDMVVGVKVDELERKLNKYTQSFF-------------------------------------- | atgggtgctaacgtttctactccagaccagagatttcaggtaacccacttccgttcaacg aagccatggacacctcgacctgagatttatcctttcaaagtcaacagtccttgtattgtg gagatcaagaacatgaatcagtggaaatctaggctcaacgctcttaaagacaccaacaag ctgctggtgatcgagttcacagctaaatggtgtggaccatgtaaaacccttgaaccaaag ctcgaagagttggctgctaaatacaccgatgttgagttcgtgaagattgatgtcgatgtc ttaatgagcgtgtggatggagttcaaccttagcactttacctgcgattgtattcatgaaa agaggccgagaagtagacatggttgtgggtgtgaaggttgatgagttagagaggaaactc aataaatacacacaatccttcttctga |
| Q9AR82 | UniRef cluster | ----------------------------MAGSSEEGQVISCHSVDAWNDILHRGNESKKL-----IVVDFTASWCGPCRFIAPFLGELAK-KFT-NVIFLKVDVDELKSVAQDWAVEAMPTFVFVKEGTILGKVVGAKKEELQQTIERHVASSNA------------------------------------- | atggcaggttcatcagaagagggacaagttatcagctgtcacagcgttgatgcatggaac gatatccttcacagaggcaatgaatccaagaaactgattgttgtggactttactgcttct tggtgtggaccatgccgtttcattgcaccattccttggtgaattggctaagaagtttaca aatgtcatattccttaaggtggacgtggacgaactaaagtctgttgctcaagattgggct gttgaggctatgccaacatttgtgtttgtgaaagaaggaacgattttgggcaaagtggtt ggagcaaagaaagaagaactgcagcagacaattgaaagacatgtggcttcatctaatgct tag |
| Q8H9E2 | UniRef cluster | -------------------------------MAVEGQVIPLRDAEEFDAIIDKEKESGRL-----IVIDFTASWCPPCRFIAPVFAELAK-AHV-HVTFLKVDVDNVKEIAKRFEVTAMPTFVFLKGGNEVHRIVGADKVELGVKVLELSAAPATCAS---------------------------------- | atggctgttgaaggtcaagtgattccacttcgcgatgctgaagaattcgatgcgataatc gataaagagaaggaatctggcagactgattgtgatcgattttactgcttcctggtgcccg ccatgccgtttcattgctccagtattcgcagagttggctaaggcgcacgtccatgtcact ttcttgaaagtggacgtcgataatgtcaaggaaattgctaagaggttcgaggtgacagcg atgccgacctttgttttcctgaaaggaggaaacgaagttcacaggattgttggtgcggac aaggtggagctaggggttaaagtactggagttaagcgctgcacctgctacttgtgcttct tag |
| A1ECJ9 | UniRef cluster | -------------------------------MAEEGQVISCHTVESWNEQLQKGIAAKKL-----IVVDFTASWCPPCKLMSPILSELAK-KLP-AVIFLKVDVDELKSVAEEWAVEAMPTFVLTKEGKVLERIVGAKKDELQLN----------------------------------------------- | atggccgaggagggacaagtgatcagctgccacactgttgaatcctggaacgagcagctc caaaagggcattgcggcaaagaaactgatagtggtggattttacggcttcatggtgcccg ccatgcaagttgatgtctccaattttgagtgaattggcgaagaagctgcccgctgtcata ttcttgaaggtggatgttgatgaattgaagtctgtcgctgaggaatgggctgtggaagcc atgccgacctttgtactgacgaaagaaggcaaagtcctggagaggattgtgggagcgaag aaagatgagctgcagctgaattag |
| A5DPF9 | UniRef cluster | --------------------------------------------MIVEVKSKQQLIDAIQ-YHGLVVVDFYAQWCGPCKTVSPILEKLSD--EYDEVKFLKVDVDALSELTLEYEVTSMPTILFFKEGNKEGRVVGANIPALRQVIAALA------------------------------------------ | atgatagtcgaggttaaatccaagcaacagctaatagatgctatacaataccatggctta gtggtggtagatttctatgcgcaatggtgtggtccttgtaaaaccgtctcacctattttg gaaaaattatctgacgagtatgatgaagtgaaatttttaaaagttgatgttgatgcattg agcgagttgacactggaatatgaagtcactagtatgcctacaattttatttttcaaggaa ggaaataaagaaggaagggtagttggtgccaatattcctgcgttgagacaagtaattgct gcacttgcttaa |
| Q39239 | UniRef cluster | ------------------------------MAAEEGQVIGCHTNDVWTVQLDKAKESNKL-----IVIDFTASWCPPCRMIAPIFNDLAK-KFMSSAIFFKVDVDELQSVAKEFGVEAMPTFVFIKAGEVVDKLVGANKEDLQAKIVKHTGVTTA------------------------------------- | atggcggcagaagagggtcaagtgattggttgtcacacgaacgatgtatggactgtgcaa cttgataaagccaaagaatccaacaagctgattgtgattgatttcactgcttcatggtgt ccaccatgccgcatgattgctccaattttcaacgatttggccaagaagttcatgtcaagt gccatcttcttcaaggtggatgttgatgaacttcagagtgttgctaaagagtttggtgtg gaggcaatgccaacctttgtgttcattaaagccggcgaagttgtggataagctcgttggt gcgaataaagaagatcttcaggcgaaaatagtgaagcatactggtgttacaactgtcgta aatcagtttgaggcataa |
| Q5ZF48 | UniRef cluster | ----------------------------MATSSEEGQVYSVHSVEEFKDHLEKSKEAKKL-----VVIDFTASWCGPCRFIAPILAELAK-KTP-HVMFLKVDVDELKAISVEYEVEAMPTFVFLKDGKPIDRLVGAKKEDLLAKITTHGTVVA-------------------------------------- | atggctacttcttcagaagagggacaagtgtacagcgttcactccgttgaggagtttaag gatcatcttgagaagagcaaggaggccaagaagctggtggttattgacttcactgcttcg tggtgtggaccatgccgcttcatcgccccaatcttggctgagctcgcgaagaagacacca catgttatgtttctcaaggtcgatgttgatgaactgaaggctatttcagtggagtacgaa gtcgaggctatgccgacttttgttttcctcaaggacgggaagccaattgacaggcttgtt ggtgcaaagaaggaagatctgctggcaaagattaccacacatggtactgttgtggcttga |
| Q541W4 | UniRef cluster | ------------------------------MAAEEGQVIGCHTNDVWTVQLDKAKESNKL-----IVIDFTASWCPPCRMIAPIFNDLAK-KFMSSAIFFKVDVDELQSVAKEFGVEAMPTFVFIKAGEVVDKLVGANKEDLQAKIVKHTGVTTA------------------------------------- | atggcggcagaagagggtcaagtgattggttgtcacacgaacgatgtatggactgtgcaa cttgataaagccaaagaatccaacaagctgattgtgattgatttcactgcttcatggtgt ccaccatgccgcatgattgctccaattttcaacgatttggccaagaagttcatgtcaagt gccatcttcttcaaggtggatgttgatgaacttcagagtgttgctaaagagtttggtgtg gaggcaatgccaacctttgtgttcattaaagccggcgaagttgtggataagctcgttggt gcgaataaagaagatcttcaggcgaaaatagtgaagcatactggtgttacaactgcgtaa |
| A9P8G6 | UniRef cluster | ------------------------------MAAEDGQVIGCHTVEAWDEQLQRGNESKKL-----VVIDFAASWCGPCRVIAPFLAELAR-KLP-DVIFLKVDVDELKTVAQDWAVEAMPTFMFLKEGKIVDKVVGARKDELQQAIAKHTAPAAATASA--------------------------------- | atggcagctgaagatggacaagtgatcgggtgccacactgttgaggcgtgggacgagcag ttgcagagaggaaatgaatctaagaagctggtggtgattgattttgctgcttcatggtgt ggtccgtgccgtgtcattgctcctttcctggctgagctggctaggaaacttcccgatgtt atcttccttaaggttgatgttgatgaattgaagactgtcgctcaggattgggctgtggag gcaatgccaactttcatgttcctgaaagaggggaagattgtggacaaagttgtgggagca aggaaggatgaactgcagcaggctatagcaaagcacacagctcctgctgctgctactgct tctgcttga |
| Q1W2C2 | UniRef cluster | -------------------------------------------MVHHVKDEKDLEAKLKEAGNNLVVIDFFATWCGPCKLIAPHIETMDE-EFPD-VMFLKVDVDECEGIAAQYEISSMPTFVFIKNSKQLENFAGANAEKLKETVNKHK------------------------------------------ | atggttcatcacgtaaaggacgagaaagacctcgaagccaagctgaaggaggctggtaac aacttggttgttatcgatttcttcgccacctggtgcggaccctgcaagctgatcgctccc cacattgagacgatggatgaagagttcccggatgtgatgttcctgaaagtggacgtagat gagtgtgagggcatcgctgcccaatatgagatctcctcaatgccaacctttgtattcata aagaatagcaaacaattggaaaactttgccggagcaaacgctgagaagctgaaagaaacc gtcaacaagcataaatga |
| B0EFZ8 | UniRef cluster | --------------------------------------------MAVLHINALDQLTALLSTEKVIVIDFFATWCGPCRSIGPYFEELAG--QHNNIKFVKVDVDQAEEICVNYKVRSMPTFVLVKDGIEQKRFSGADRNALKQMIETA------------------------------------------- | atggctgtacttcatatcaatgcccttgatcaacttactgctcttctttctactgaaaaa gttatcgttattgatttctttgctacatggtgtggtccttgcagatctattggcccatat tttgaagaacttgctgggcaacacaataacattaaattcgttaaagttgatgtagatcaa gctgaagaaatttgtgttaattataaggttagatcaatgccaacatttgttcttgttaaa gacggaatagaacaaaaacgttttagtggagcagatagaaatgctttaaaacaaatgatt gaaacagcataa |
| Q6XHI1 | UniRef cluster | -------------------------------------------MVYQIKDKADLNGQLTKASGKLVVLDFFATWCGPCKMISPKLAELST-QYADTVVVLKVDVDECEDIAMEYNISSMPTFVFLKNGVKVEEFAGANAQRLEDVIKANI------------------------------------------ | atggtgtaccagattaaagataaggccgaccttaatggacagctgaccaaggcatccggc aagctggtggtgctggacttcttcgccacctggtgcggaccctgcaagatgatctcgccc aagctggctgagctctccacgcagtacgccgacaccgtcgtcgtcctgaaggtcgatgtg gacgaatgcgaagacattgcaatggaatacaacatctccagcatgcccaccttcgtgttc ctcaagaacggcgtcaaggtcgaggagttcgccggtgccaacgcccagcgtttggaggat gtcatcaaggctaacatc |
| Q7ZUI4 | UniRef cluster | -------------------------------------------MIVVIEDQDGFDKALAGAGDKLVVVDFTATWCGPCQSIAPFYKGLSENPDYSNVVFLKVDVDDAQDVAQSCEIKCMPTFHFYKNGKKLDDFSGSNQTKLEEMVKQHKN----------------------------------------- | atgatcgtcgtcatcgaagaccaggatggctttgacaaagctttggctggggcaggtgat aagctggtagtggtggacttcacagccacgtggtgtgggccttgtcagagcatcgcacct ttttacaagggtctgtctgaaaatcctgactattctaatgtggtcttcctcaaagtagac gtggacgacgcacaggatgtagcgcagtcgtgcgagatcaaatgtatgccaacattccac ttctacaagaacgggaagaagctcgatgatttctctgggtccaaccagactaagctggaa gagatggtgaaacaacacaaaaactga |
| Q1HFX6 | UniRef cluster | -------------------------------------------MKKVEEATDLMDYFTKVEGQKAVIIDFNADWCGPCQVIKPIFAQYSE--EYPNIKFISVNTEKNKEVAQQFGIQSLPTFITLHEGEISATWKGANQVNLKNNLDKLAEKLK-------------------------------------- | atgaagaaagttgaagaagcaactgatttaatggattactttactaaggtagagggacaa aaagcagtgattatcgactttaacgctgattggtgtggaccttgttaagtaatcaagcca atatttgcttaatatagtgaagaatacccaaatatcaaatttatttctgtaaatacagaa aaaaacaaggaggttgcccaataatttggcatttaatctctccccaccttcatcaccctt catgaaggtgaaatatctgcaacttggaaaggagctaatcaagtaaacttaaaaaataac cttgataaattagcagaaaagcttaaatga |
| A8XGT0 | UniRef cluster | -------------------------------------MSLSKEPILELSDMAEFNHLVRQHPEKVIVLDFFATWCGPCKAIAPLYRELAI--EYKGVIFCKVDVDEAEDLCAKYDVKMMPTFVFTKNGETLETLEGGVEEELRRKVREHSLAASSSAVAH-------------------------------- | atgtctctctccaaggaaccgatcctcgagttgtccgatatggccgagttcaatcatctg gttcgccagcatccagagaaagtcatcgtcttggacttcttcgccacgtggtgtggacca tgtaaggcgatcgctccattgtacagggaactggccatcgagtacaagggagtcatcttt tgcaaagtcgatgttgatgaagccgaagacctctgcgccaagtacgacgtcaagatgatg cccacgttcgtcttcaccaaaaacggagagacactggagactctggaaggtggtgtcgag gaggaacttcgccgaaaagtacgcgagcactcgttggcggcttcttcctcagccgttgct cattga |
| Q240X9 | UniRef cluster | ------------------------------------------MSHPYIEITSTKQFEDILEKNEYVLVDFFASWCGPCKILAEQFEPIKKDHKNLTIVKVNIDEEDLESIVESHNVSSLPHVFLYHKKTVVKQFIGNQKDQLWEMAKLSDAK---------------------------------------- | atgtctcatccctacatagaaattacaagcaccaagcaattcgaagatattctcgaaaag aacgaatatgttttagttgatttctttgcttcctggtgtggcccttgcaaaattttagct gaataattcgaacctattaagaaagatcacaagaacctaactattgttaaggttaacatt gacgaagaagatttagaatctattgttgaatctcacaatgtttcttctcttcctcacgtt ttcttataccacaagaagactgttgttaaataattcattggtaaccaaaaagaccaatta tgggaaatggctaaactcagtgatgctaagtga |
| Q1HFX5 | UniRef cluster | ------------------------------------------MSHPYIEITSTKQFEDILEKNEYVLVDFFASWCGPCKILAEQFEPIKKDHKNLTIVKVNIDEEDLESIVESHNVSSLPHVFLYHKKTVVKQFIGNQKDQLWEMAKLSDAK---------------------------------------- | atgtctcatccctacatagaaattacaagcaccaagcaattcgaagatattctcgaaaag aacgaatatgttttagttgatttctttgcttcctggtgtggcccttgcaaaattttagct gaataattcgaacctattaagaaagatcacaagaacctaactattgttaaggttaacatt gacgaagaagatttagaatctattgttgaatctcacaatgtttcttctcttcctcacgtt ttcttataccacaagaagactgttgttaaataattcattggtaaccaaaaagaccaatta tgggaaatggctaaactcagtgatgctaagtga |
| Q4N2X5 | UniRef cluster | ---------------------------------------------MVHEVTSKEEFEKTLSGDSVVVVDFYADWCGPCMRFAPQFDALAT--EHPSLLFVKVNVDKLQELAQKYNVTSLPTFKVFKSGQVLGEFLGASKEGLKNTLLK-------------------------------------------- | atggttcatgaagttacttcaaaagaagaatttgaaaagacactttctggtgattccgta gttgtcgttgacttttacgctgattggtgtggcccttgcatgagatttgctcctcaattt gacgctttggctactgaacacccttcacttttatttgtcaaggtaaacgtcgacaagcta caggaacttgcacaaaagtataacgtcacaagccttccaacctttaaagttttcaaatct ggacaagttcttggcgagtttcttggcgcaagcaaagaaggactaaagaacacattgtta aagtaa |
| A2Q7Z6 | UniRef cluster | -----------------------------------------MSHKVHDITSKAEFAEKVTNSTDAIVLDCFATWCGPCKAISPKVEEFSNTY--PNAKFYKIDVDELSEVAAELGIRAMPTFLLFKDGKKFDDLTGANPKGLEQKIQALLA----------------------------------------- | atgtctcacaaggttcacgacatcacctctaaggccgagttcgccgagaaggttaccaac tccaccgacgccatcgtccttgactgcttcgctacctggtgcggtccttgcaaggctatc tcccccaaggtcgaggagttcagcaacacctaccccaacgccaagttctacaagatcgac gtcgacgaactgtccgaggttgctgccgagctcggtatccgtgccatgcccactttcctg ctcttcaaggacggcaagaagttcgacgacctcaccggtgccaaccccaagggtctggag cagaagatccaggctctgcttgcataa |
| A4H351 | UniRef cluster | --------------------------------------------MPFTEVYGVEQFREIANEPTLTVVCFSAVWCGPCKTIEKDLDRLTY--EFSNVRFAKVDADNNTEIVTKCRVMQLPTFMLVKAGQMLGYVIGADLAQLKARIREEANKS--------------------------------------- | atgccctttacggaggtgtatggcgtggagcagttccgtgaaatcgcgaacgagcccact ctcaccgtcgtgtgctttagcgcggtgtggtgcggtccatgcaagaccatcgagaaggat ctggacaggctcacttacgagttttcgaatgtgcgctttgcgaaggtggacgcagacaac aacacggaaatcgtaaccaagtgccgagtgatgcagctgccgaccttcatgctggtgaag gcggggcagatgctcgggtatgtcatcggggcagacttagcgcagctcaaggcaaggata cgagaagaggcgaacaagtcgtga |
| Q9TXY8 | UniRef cluster | -------------------------------------------MSIAIKDDDEFKTIFAEKKTQPVILFFTASWCGPCQMIKPRVEELAA-EHKDRLSILKIDVDECDGVGEEYEINSMPTFLLIVDGIKKDQFSGANNTKFEEMVKAALQ----------------------------------------- | atgtctatcgcaatcaaggacgatgacgaattcaagacaatttttgccgagaagaaaacc cagccagtgattctatttttcacggcttcatggtgcggtccgtgccaaatgatcaagcca cgtgtggaggagctggccgccgagcacaaggatcgcctatcgatccttaaaattgatgtg gacgagtgtgacggtgtcggtgaagaatatgagatcaactcgatgcccaccttccttctg attgtcgacggaatcaagaaggatcagttcagtggagcgaacaatacaaagttcgaggaa atggtcaaggcagctcttcaatga |
| A4HRE8 | UniRef cluster | --------------------------------------------MPFTEVYGVEQFRDIANDPTLTVVCFSAVWCGPCKTIEKDLDRLTY--EFANVRFAKVDADNNTEIVSKCRVMQLPTFMLVRAGQMLGYVIGADLAQLKAKIREEANKS--------------------------------------- | atgcccttcacggaggtgtatggcgtggagcagttccgtgacatcgcgaacgatcccacg ctcaccgtcgtgtgcttcagcgcggtgtggtgcggcccatgcaagaccatcgagaaggat ctggacaggctcacctatgagtttgcgaatgtgcggtttgcgaaggtggacgcagacaac aacacggaaatcgtgtccaagtgccgcgtgatgcagctgccaacgttcatgctggtgagg gcggggcagatgctcggctacgttatcggagcagacttagcgcagctgaaggcaaagata cgagaggaggcgaacaagtcgtga |
| Q84XS1 | UniRef cluster | -------------------------------------MGGRIIQVKSAEEWTSHMRECRAFGGKSYLVDFTASWCGPCQKIAPLYEKYS--AENPNITFLKVDVDELADVAGECDVKAMPTFIGYFNGEQVDTVVGADDAKLRELIAT-------------------------------------------- | atgggcggtcggattatccaggtgaagagcgcggaggagtggacaagccatatgcgggaa tgccgcgcattcggaggcaaatcctatctcgtggacttcacggcatcgtggtgcggaccc tgccaaaaaatcgccccgctctatgagaagtacagcgccgagaacccaaacatcacattc ctcaaggtggacgtcgacgaactggcggacgtggcgggcgagtgcgacgtcaaggccatg cccactttcatcggctacttcaacggcgagcaggtggacacagtggtgggcgccgacgac gccaagctgcgtgagctcatcgccacgtga |
| A2FVZ8 | UniRef cluster | ----------------------------------------MSDPIVHFQGSNQDLLSRIKEASGLVVVDFFATWCPPCQYLGKILPSISQ--DNKDVTFIKVDIDQNDDATTAFNVSSIPSLFIMKKDGDEITTLDHFVGADVARIMLDISKFK-------------------------------------- | atgtccgatccaattgttcacttccaaggcagcaatcaagatctactcagcaggataaag gaagcttctggtttagtagttgtagatttctttgcaacttggtgtccaccatgccaatac cttggcaagatacttccatcaatttcacaagataacaaagacgtcacattcattaaagtt gatatagatcaaaatgatgatgctacaacagccttcaatgtttcatcgattccatctctt ttcatcatgaagaaggatggtgacgaaattactacgcttgatcattttgttggtgcagat gtagctcgtatcatgcttgatatctcgaaattcaaataa |
| A2F5G9 | UniRef cluster | ----------------------------------------MTDAIIHFNGSADELESTIKAKKGLVVVDFFATWCGPCKRLGQILPGIAT--ENSDVTFIKVDIDQNDDAASKFGVSSIPHIAFCKANDGNYEVINTVVGCNVEAINKLIAANK-------------------------------------- | atgactgacgcgatcatccacttcaacggttctgctgacgagctcgagagcacaatcaag gctaagaagggcctcgttgttgttgatttcttcgctacatggtgcggcccatgcaagcgt cttggacagatcctcccaggcattgctacagaaaacagtgatgttacattcatcaaggtc gatattgaccagaacgatgatgctgcttccaaatttggcgtttcatctatcccacacatt gctttctgcaaggctaacgatggcaactatgaagtcattaacacagttgtcggctgcaat gtcgaagctatcaacaagttgatcgctgctaacaagtaa |
| A7R223 | UniRef cluster | -------------------------MENQEPEENKSRVIKVVSEELWDFYISQATTQGCP-----VVVHFTAAWCIPSVAMNQFFEELAS-NYP-DALFLTVDVDEVKAVAVKMEVKAMPTFLLMKEGAQVDRLVGANPDEIRKRIDALVQSFRVYVA---------------------------------- | atggaaaatcaggagccggaggaaaacaagtctagagttattaaggtggtgtctgaggag ttatgggatttctatatcagccaagccaccacccaaggctgccctgttgttgtgcacttc actgctgcatggtgcatcccctctgtggccatgaaccagttttttgaagaactggcttca aattacccagatgctctgttcctcactgttgatgtggatgaagttaaggcggttgcggtc aaaatggaagtaaaggccatgcccacatttctgctgatgaaggaaggagctcaggtggac aggctggtgggtgccaatccagatgagataaggaaaaggatagatgctttggtgcagtcc tttcgcgtgtatgtagcctag |
| A5DP99 | UniRef cluster | ------MFSLKAIRTSRPVFRNTNVWASPVKFTPMRFYSAAHSGSVKELTELAGFFDFIK-KENVSVVDFYATWCGPCKALEPIYNMFAE--RIPEVQFGRVDVDEAQDIATEYAISAMPTCLIFKDGENVGKIVGADPHKLLEMIQEHANVDLKSIR---------------------------------- | atgttttcattaaaagcaatcagaacatccagaccagtatttaggaacacaaatgtctgg gcttctcccgtgaaattcactcctatgcggttctattcagcagcacattcaggatctgtc aaagaattgaccgagcttgcgggtttcttcgactttatcaaaaaagaaaatgtgtctgtt gttgacttctacgctacttggtgcggtccatgtaaggcattggagcctatttacaacatg tttgcggagagaattcctgaagtacaattcggaagagtagacgtagacgaagcccaggac attgctactgagtatgcgatttcggctatgcccacatgtttgattttcaaagatggcgag aatgtcgggaagatcgtgggcgcagatccacataagcttcttgaaatgatccaagaacat gccaatgttgatttgaagtcgataaggtag |
| Q9VUG9 | UniRef cluster | ------------------------------------MAAMQKKVIIVDSKSYFDKLIDDAGTNKYVLVEFFATWCGPCAMIGPRLEQLAS-DYFGRMLVLKIDVDENEDLAVQYEVNSMPTFLIIKNRVTLIQFVGGNVERVVSTVEKFVGKVEDSKEHKSKEGGASSATVPKLER---------------- | atggctgccatgcagaagaaggtcatcattgtggattcgaagagttactttgataagctc atcgatgatgcgggaaccaacaaatacgtacttgtcgagttcttcgccacctggtgtggt ccttgcgcgatgattggtccccgcttggagcaactggcatcggattacttcggacggatg ttggtccttaagattgacgttgatgagaacgaagatctggccgttcagtacgaagtcaac agcatgcccacatttctgatcatcaaaaaccgagtgacactaatccagttcgtgggcggc aatgtcgaaagggtcgtcagcacggtggagaagtttgtgggcaaggtggaggactccaag gagcacaagtcgaaagagggaggtgctagttcagctaccgtgccgaaattggaacgctaa |
| A2GDJ3 | UniRef cluster | ----------------------------------------MSSNIISFEGNYSELVEAIKSKTGLVVINFFATWCEPCKKLGMQLPKIAQ--ENSNVTFFKVDIDSNEQIAKMYNIIHIPQLSFMRVEGKTINDLDTILGFDVNKINNGLSRYR-------------------------------------- | atgtcttctaatatcataagcttcgaaggaaattattccgagttagtagaagcaatcaaa tcaaaaacaggtctagtagtaatcaacttctttgcaacatggtgcgaaccttgcaaaaaa ttaggaatgcaattaccgaaaattgcgcaggaaaactccaatgttacattctttaaagtc gatattgattcgaatgaacaaattgccaagatgtacaacattattcatatcccacaactc tcttttatgcgtgttgaagggaagacaattaatgatttagatactattctcgggttcgat gtgaataagatcaacaatggcctttcccgttacagataa |
| O96952 | UniRef cluster | -------------------------------------------MVNFLKTKADFDQALKDAGDKLVVIDFTASWCGPCQRIAPKYVEMAK--EFPDVIFYKVDVDENDETAEAEKIQAMPTFKFYKSGKALSDYVQGANEAGLREKIKKNK----------------------------------------- | atggtgaattttctaaagacgaaggctgacttcgatcaggccctgaaggacgcgggcgac aagctggttgtcatcgacttcacggcctcctggtgtggcccgtgccagaggatcgccccc aagtacgtggagatggccaaggagttcccggacgtaatcttctacaaggtggatgtggat gaaaatgacgagactgcagaggcggagaaaatccaagccatgccgaccttcaagttctac aagagcgggaaagcactgtccgactatgtacagggagccaatgaggcgggtctgagagag aagatcaagaagaacaagtag |
| Q09433 | UniRef cluster | ------------------------------------MLKRCNFKNQVKYFQSDFEQLIRQHPEKIIILDFYATWCGPCKAIAPLYKELAT--THKGIIFCKVDVDEAEDLCSKYDVKMMPTFIFTKNGDAIEALEGCVEDELRQKVLEHVSAQ--------------------------------------- | atgttgaaacgatgcaacttcaaaaatcaagtaaaatattttcagagtgactttgagcaa ttgatccgtcaacatccggagaagatcattattcttgatttctatgcaacttggtgcgga ccatgcaaagcaattgcaccattatacaaagaattagctacaactcacaaaggaatcatc ttctgcaaagttgatgtcgatgaagcggaagatctttgttccaaatatgatgtcaagatg atgccgactttcattttcaccaagaatggagacgcaattgaggcactggaaggctgcgtt gaggacgaactgcgtcaaaaagtgttggagcacgtatctgctcaatga |
| Q25345 | UniRef cluster | --------------------------------------------MPFTEVYGVEQFRDIANDPTLTVVCFSAVWCGPCKTIEKDLDRLTY--EFASVRFAKVDADNNTDIVSKCRVMQLPTFMLVRAGQMLGYVIGADLAQLKAKIREEANKS--------------------------------------- | atgcccttcacggaggtgtatggcgtggagcagttccgtgacatcgcgaacgatcccacg ctcaccgtcgtgtgcttcagcgcggtgtggtgcggtccatgcaagaccatcgagaaggat ctggacaggctcacctatgagtttgcgagtgtgcggttcgcgaaggtggacgcagacaac aacacggacatcgtgtccaagtgccgtgtgatgcagctgccaacgttcatgctggtgcgg gcggggcagatgctcggctacgttattggagcagacttagcgcagctgaaggcaaaaata cgcgaggaggcgaacaagtcgtga |
| A2Z435 | UniRef cluster | -------------------------------------MAGTSNSVVMTIDSWQQLIDSLKGNVVVLEFMAPWSEPSKFMEQPFKEVASEFKDKNSNVKFAALNFDNSKNLARRLQVEALPTFLVVNNFAVVDRILALSKTELQQKINDKLAQTNY------------------------------------- | atggccggtacttcaaactctgtcgtgatgaccatcgattcatggcaacagctgatagat tctctcaagggtaacgtggtggttctggagttcatggcgccttggtcggagccatccaag ttcatggaacagccgttcaaggaagtcgcctccgaattcaaagacaagaattcgaacgtc aagttcgccgctctcaactttgacaattccaagaatttagcgaggagactccaagtggag gcgctgccgacgttcctggtggtgaataatttcgcggtggtagaccgcatcctcgccctc tccaagactgagcttcagcaaaagatcaacgacaagctagctcaaactaattattaa |
| A2QGF5 | UniRef cluster | -------------------------------------MSHGKVIEVDNPVIFKALTSNGP-----VVVDFFATWCGPCKAVAPVVGKLSE-TYT-NVRFIQVDVDKVRSVAQELEVRAMPTFVLYKDGQLQEKRVVGGNMKELEAAIKEITA---------------------------------------- | atgtctcacggaaaggttattgaggttgataaccctgttatcttcaaggcgcttacctcc aacggtcccgtggtcgtcgacttcttcgcaacctggtgcggtccgtgcaaggccgtcgcg ccagtggtcggcaagctcagcgaaacctacaccaatgtgcgcttcattcaggtcgacgtt gataaggtccgttcggtggcacaggaactggaggtgcgcgctatgcccactttcgtcctg tacaaggatggccagctgcaggagaagcgcgtcgtgggtggaaacatgaaggagctggag gcagccatcaaggaaattactgcataa |
| Q9N456 | UniRef cluster | -------------------------------------------------MSKAFVDGLLQSSKVVVFSKSYCPYCHKARAALESVNVKPDALQWIE------IDERKDCNEIQDYLGSLTGARSVPRVFINGKFFGGGDDTAAGAKNGKLAALLKETGAL-------------------------------- | atgtcaaaagcctttgtcgacggacttctccaatcttccaaagttgtcgtattcagcaaa tcgtactgcccgtactgccataaggctcgtgctgctcttgaatcggtcaacgtgaagccc gacgctctgcaatggatcgaaatcgacgagcgcaaggattgcaatgagattcaggattat cttggctctctaaccggagcccgctcggtgccacgtgtcttcatcaacggcaagttcttc ggcggcggtgacgacacagcggccggagccaagaacggaaaactcgccgcactgctcaag gaaaccggagctctatag |
| Q5AH28 | UniRef cluster | -------------------------MIDKMSSILAWGFNLWYQPPPPTAQTEKEIEHTINSHKIVIYSKTYCPFCDQTKHLLNEQYPQES------YEVINLNILD-DGLTIQNQLYANTGQYMVPIIFINGQHVGGNSEVQQLHT----NGKLQELLNPQKY----------------------------- | atgatagacaaaatgctgctgattcttgcctggggattcaatttgtggtatcaaccacct ccacctactgcacaaactgagaaagaaatcgaacacactattaactctcacaagattgtt atttattctaaaacttattgtccattttgtgaccaaaccaaacatctattaaatgaacaa tatccacaagaatcgtacgaagtcataaacttgaatattctcgatgacggattgactatt cagaatcaattgtatgctaatactggtcaatatatggtgcccataatcttcataaacgga caacacgttggaggaaattcagaagttcagcaattgcacaccaatgggaaattgcaagaa ttattgaatcctcagaaatattga |
| Q6DH06 | UniRef cluster | -----------------------------------MGNFSSSAPGLSSSACGQFVQDIVSSNCVVIFSKTTCPYCKMAKGVFNEIGA--TYKVVE----LDEHN---DGRRLQETLAELTGARTVPRVFINGQCIGGGSDTKQLHQQGKLLPLIEQCRPCCLNMTPEGSGNSQNQPHQ-------------- | atggggaacttctcgtcatctgcgcctgggttgtcaagctcagcttgtggtcagtttgtg caggatattgtgtccagtaactgtgttgtaatattctccaaaacaacatgtccttactgt aagatggcaaagggtgtctttaatgagattggagctacgtataaagttgttgaactggac gagcacaacgatggtcgacgtctccaggagactttagcggaactgacaggtgccagaaca gtgccaagagtttttattaatggacagtgcattggaggaggctcagatacaaaacagctc caccagcaaggaaaacttctgcctcttattgaacagtgtaggccgtgctgtttaaatatg acacctgagggctcaggaaatagtcaaaatcagcctcatcagtga |
| Q16HL8 | UniRef cluster | ----------------------------------MGSFVSRSPPANMSGPVAEFVKSAIAKDKVVIFSKTYCPYCTMAKEPFKKLNQ--PVACYE----LDQRN---DGDEIQVVLGNLTGARTVPRVFINGNFVGGGTDIKKMYSDGRLEKLLL------------------------------------- | atgggttctttcgtcagtagatctcctcctgccaatatgagtggacctgtagccgaattc gtgaagagcgccatcgccaaagacaaggtggtcattttttccaagacctattgtccgtac tgcacaatggccaaagagcctttcaagaagctcaatcaaccagttgcctgctatgaactg gatcaaaggaatgatggcgatgaaatccaggtggtactcggaaatttaacaggtgccaga acggtaccaagagtattcataaacgggaattttgtcggcgggggaaccgacattaaaaag atgtactccgatggacggttagaaaaactgttattatag |
| Q9FVX1 | UniRef cluster | ------------------MVDQSPRRVVVAALLLFVVLCDLSNSAGAANSVSAFVQNAILSNKIVIFSKSYCPYCLRSKRIFSQLKEE--PFVVE----LDQRE---DGDQIQYELLEFVGRRTVPQVFVNGKHIGGSDDLGAALESGQLQKLLAAS----------------------------------- | atggttgaccagagtcctcgccgtgttgtcgtggcggcgctcctattgtttgtggttctg tgcgatctttcgaattctgcgggagctgcgaattctgtgtcagctttcgttcagaacgcc atcttgtccaacaagattgtcatcttctccaaatcctactgcccgtattgtttgcggtcg aaacgtatattcagccaacttaaggaagagccatttgttgtggagcttgatcagagagag gacggagatcaaatccagtatgagcttttagaattcgttggtcgtcgtactgtcccgcaa gtttttgttaacggcaagcatattggtggatcagatgatcttggagctgctttggagagt ggtcagttgcaaaagcttcttgctgcaagttga |
| O65169 | UniRef cluster | --------------MKRNGGNYASLCYLRNVMAVIMVGMIWMNEAMASNSASAFVNNVIYSNRIAVFSKSYCPYSVRAKRVFSDLQER--PFVVE----LDLRD---DGSEIQDVLLELVGRRTVPQVFVNGKHIGGSDDLHSAVMSGLLQKHLSTS----------------------------------- | atgaagagaaatggcggaaattatgcgagtttgtgttatctaaggaatgtgatggcggtg ataatggtgggaatgatttggatgaacgaagctatggccagcaattcagcttccgcattt gttaacaacgtcatctactccaaccgaattgcagttttctccaaatcctactgcccatat tctgtgcgtgcgaagcgtgtatttagtgacctgcaggaacggccatttgttgttgagctt gatcttcgagatgatgggtctgaaattcaggatgttcttcttgaattggttggtcgacgc acagtcccacaagttttcgtgaatgggaaacatataggaggctctgatgatctccacagt gcagtcatgagcgggctgttgcagaagcatctctcaactagttga |
| Q1HQT9 | UniRef cluster | ----------------------------------MGSFVSRSPPANMSGPVAEFVKSAIAKDKVVIFSKTYCPYCTMAKEPFKKLNQ--PVACYE----LDQRN---DGDEIQVVLGNLTGARTVPRVFINGNFVGGGTDIKKMYSDGRLEKMLL------------------------------------- | atgggttctttcgtcagtagatctcctcctgccaatatgagtggacctgtagccgaattc gtgaagagcgccatcgccaaagacaaggtggtcattttttccaagacctattgtccgtac tgcacaatggccaaagagcctttcaagaagctcaatcaaccagttgcctgctatgaactg gatcaaaggaatgatggcgatgaaatccaggtggtactcggaaatttaacaggtgccaga acggtaccaagagtattcataaacgggaattttgtcggcgggggaaccgacattaaaaag atgtactccgatggacggttagaaaaaatgttattatag |
| B0WPR4 | UniRef cluster | ----------------------------------MGSFVSRSSPANMSGPVAEFVKGAIAKDKVVIFSKTYCPYCTMAKEPFKKLNH--PVTCYE----LDHRK---DGGEIQAVLGEMTGASTVPRVFINGNFVGGGTDIKKMYSDGRLEKMLA------------------------------------- | atgggttcattcgtcagtagatcatcgccagccaacatgagtggaccagtggccgaattc gtcaagggcgccatcgccaaggacaaggtcgtaatcttttccaagacctactgcccgtac tgcacaatggccaaggagcccttcaagaagctgaaccatcccgtcacttgctacgagctg gaccaccgaaaggacgggggcgagattcaggccgtgctcggtgagatgaccggagccagc acggtgccacgtgtgttcatcaacgggaactttgtcggtggcggcacggacatcaagaag atgtactcggacggccgattagagaagatgctggcctaa |
| Q9W2D1 | UniRef cluster | ------------------------------MGTVVSTLQRPTLYVSMDSSHAQFVRDTISGNKVVIFSKSYCPYCSMAKEQFRKINV--KATVIE----LDQRD---DGNEIQAVLGEMTGSRTVPRCFIDGKFVGGGTDVKRLYEQGILQKYFQ------------------------------------- | atgggtacggtggtcagcaccctgcagcgacccactctctacgtgagcatggacagctcg catgcgcagttcgtgcgggacacaatcagcggcaacaaggtggtgatctttagcaagagc tactgcccctactgcagcatggccaaggaacagttccgaaagatcaacgtcaaggcaacg gtgattgagctggaccagcgggatgatggcaacgagatccaggcggttcttggcgagatg acgggctcgaggaccgttccacgttgcttcatcgatggcaagttcgtgggtggcggcacc gacgtgaagcggctatacgaacagggcatactgcagaagtattttcagtga |
| Q55TF6 | UniRef cluster | ----MSFTALHRLSTLSTHIIRAPISTRYFATTQTAKIGSSISTPNMTADVKSLVDKAIADNKVVVFSKTYCPYCKRAKSYLAEDTKD---IEILE------LDEREDGAAIQAYLKELNGQGTVPHVYINKEFIGGSSDLLKLSHEQVKQKISAAASA--------------------------------- | atgtcttttactgctcttcatcgtctctcaacactctcaacccacattatcagagcaccc atttctactaggtatttcgctactacccaaaccgccaagataggatcatccatttcaact cccaacatgaccgccgacgttaaatctttggtcgacaaggccatcgccgataacaaggtc gtcgtattttccaagacctactgcccttactgcaagcgggccaaatcttacctcgctgag gacactaaggacatcgagatccttgagctcgacgagcgtgaggacggcgctgccatccag gcttacctcaaggaactcaacggccaaggcactgttccccatgtctacatcaacaaggag ttcatcggcggttccagcgacctcctcaaactctcccacgagcaggtcaagcagaagatc tctgctgccgcgtccgcttag |
| A7EPJ4 | UniRef cluster | -----------------------------------------------MSAAQTKTDSIIAENAVAVFSKSYCPYCKATKQLLNDLNAKYYS--------IELDQVD-DGSAIQAYLKEKTNQGSVPNIFIGQKHVGGNSDLQAKN-----KKELEAQLKELNVIVA-------------------------- | atgtctgctgctcaaacaaagactgatagcattattgctgagaatgctgttgccgtattc agtaaatcttactgcccatactgcaaagctaccaagcagcttttgaatgatttaaacgct aaatactactccatcgaattggaccaagttgacgatggatccgcaatccaagcctacctc aaagaaaaaacaaaccaaggttccgttccaaacatcttcatcggccagaaacacgtcggt ggtaactcggatttgcaagccaagaataagaaggagctcgaggcgcagttgaaggagttg aatgttattgttgcgtaa |
| Q6K953 | UniRef cluster | -----------------------MGMAQSSSSSSRPSDSEQLEEPSKPVMALDKAKEIVASSPVVVFSKTYCPFCARVKRLLAELAASYKAVELD----VESD-----GSELQSALADWTGQRTVPCVFIKGKHIGGCDDTMAMHKGGNLVPLLTEAGAIATPSL--------------------------- | atggggatggcacagtcgtcttcgtcttcctcgcgcccctccgactccgagcagctagag gagcccagcaagccggtcatggcgctcgacaaggccaaggagatcgtcgcctcctccccc gtcgtcgtcttcagcaagacttattgccctttctgcgcccgagtgaagcgattgctggca gagctggcagcaagttacaaggctgttgaattggatgtggaaagtgatgggtctgagctg cagtcagctcttgccgattggactggacagagaactgttccttgtgtcttcattaaaggg aaacatattggtggctgtgacgataccatggcgatgcacaaaggagggaacttggtccct ctgctgacggaggcaggagcaatcgccactccttccctgtag |
| A3A940 | UniRef cluster | -----------------------MGMAQSSSSSSRPSDSEQLEEPSKPVMALDKAKEIVASSPVVVFSKTYCPFCARVKRLLAELAASYKAVELD----VESD-----GSELQSALADWTGQRTVPCVFIKGKHIGGCDDTMAMHKGGNLVPLLTEAGAIATPSL--------------------------- | atggggatggcacagtcgtcttcgtcttcctcgcgcccctccgactccgagcagctagag gagcccagcaagccggtcatggcgctcgacaaggccaaggagatcgtcgcctcctccccc gtcgtcgtcttcagcaagacttattgccctttctgcgcccgagtgaagcgattgctggca gagctggcagcaagttacaaggctgttgaattggatgtggaaagtgatgggtctgagctg cagtcagctcttgccgattggactggacagagaactgttccttgtgtcttcattaaaggg aaacatattggtggctgtgacgataccatggcgatgcacaaaggagggaacttggtccct ctgctgacggaggcaggagcaatcgccactccttccctgtag |
| A2X798 | UniRef cluster | -----------------------MGMAQSSSSSSRPSDSEQLEEPSKPVMALDKAKEIVASSPVVVFSKTYCPFCARVKRLLAELAASYKAVELD----VESD-----GSELQSALADWTGQRTVPCVFIKGKHIGGCDDTMAMHKGGNLVPLLTEAGAIATPSL--------------------------- | atggggatggcacagtcgtcttcgtcttcctcgcgcccctccgactccgagcagctagag gagcccagcaagccggtcatggcgctcgacaaggccaaggagatcgtcgcctcctccccc gtcgtcgtcttcagcaagacttattgccctttctgcgcccgagtgaagcgattgctggca gagctggcagcaagttacaaggctgttgaattggatgtggaaagtgatgggtctgagctg cagtcagctcttgccgattggactggacagagaactgttccttgtgtcttcattaaaggg aaacatattggtggctgtgacgataccatggcgatgcacaaaggagggaacttggtccct ctgctgacggaggcaggagcaatcgccactccttccctgtag |
| Q9VVT6 | UniRef cluster | ------------------------------MGAVGSALRSP--IVDMSTKQAKFVENTIASNKVVIFSKTYCPYCTMAKEPFKKLNV--DATIIE----LDGNP---DGNEIQAVLGEITGARTVPRVFIDGKFIGGGTDIKRMFETGALQKYFQ------------------------------------- | atgggtgcagttggatccgctttgagatccccaatagtcgacatgtccaccaagcaggcg aaattcgttgaaaacaccattgccagcaacaaagtggtgatattcagcaagacctactgt ccctactgcacgatggccaaagagcccttcaaaaagctcaatgtggacgccaccataata gaactcgatggaaatcccgatggcaacgaaattcaggcagttctgggcgagattaccggt gccagaacggttccccgcgtctttatcgatggcaaattcattggcggtggcactgacatc aaacgaatgttcgagacaggagctctgcaaaaatatttccaataa |
| Q7QHA7 | UniRef cluster | ----------------------------------MGSLVSRSVPANMSGPVAEFVKSAIAKDKVVIFSKTYCPYCTMAKEPFKKLNQ--EYACYE----LDKRN---DGDEIQSVLGELTGARTVPRVFIGGNFVGGGTDIKKMYDDGRLQKMLA------------------------------------- | atgggttcattggttagccgaagcgttccagcaaacatgagcggtcccgtagcggaattt gtgaagagcgctatcgcgaaggataaggtggttatcttctccaagacctactgcccttac tgtaccatggcaaaggagcccttcaagaagctgaaccaggagtacgcctgctacgagctg gacaagcgtaacgatggtgacgagattcagtccgtcctcggcgagctgaccggtgcccgc acggtgccgcgcgtgttcattggcggcaattttgtcggtggcggtaccgacatcaagaag atgtacgacgacggtcggttacagaaaatgttggcctaa |
| A9NMQ2 | UniRef cluster | ----------------------------------------------------------MYSFFGSCCSKTYCPYCTQVKQLLSSLGAKTKVVELD----TESD-----GKEIQTALQEWTGQRTVPNVFIGGTHIGGCDDTVAKHNSGKLVPLLTEAGGV-------------------------------- | atgtattctttttttggttcttgctgcagcaagacatactgcccctattgtacacaggtc aaacagctgttaagtagcttgggtgccaagactaaggtggtagagctggatactgaaagt gatggaaaggagatacagacagctcttcaggaatggacaggacagcggactgtcccaaat gtatttattggaggcacacacattggcggctgtgatgacaccgttgcgaaacataacagt gggaagcttgtaccattgttgacagaagctggcggtgtttga |
| Q16HL7 | UniRef cluster | ----------------------------------------------MSGPVAEFVKSAIAKDKVVIFSKTYCPYCTMAKEPFKKLNQ--PVACYE----LDQRN---DGDEIQVVLGNLTGARTVPRVFINGNFVGGGTDIKKMYSDGRLEKLLL------------------------------------- | atgagtggacctgtagccgaattcgtgaagagcgccatcgccaaagacaaggtggtcatt ttttccaagacctattgtccgtactgcacaatggccaaagagcctttcaagaagctcaat caaccagttgcctgctatgaactggatcaaaggaatgatggcgatgaaatccaggtggta ctcggaaatttaacaggtgccagaacggtaccaagagtattcataaacgggaattttgtc ggcgggggaaccgacattaaaaagatgtactccgatggacggttagaaaaactgttatta tag |
| A7QE38 | UniRef cluster | --------------------MKARHHSVVLVILLGVLVANAPGQTLASNSVPAFVQNTIYANKIAIFSKSYCPYCLRAKRIFSELHEE--PFVVE----LDHRD---DGTQIQNVLLDLVGRSTVPQIFVNGKHIGGSDDLRNAVLSGQLQKQLGTS----------------------------------- | atgaaggcccggcatcacagtgtggttcttgtgatactgctgggtgttttggtggcaaat gctccgggtcaaaccctagcttccaattcagttccagccttcgtgcaaaacacaatctac gccaacaagattgccattttctccaaatcctattgcccgtattgcttgcgggcaaagcgc atctttagcgaactgcatgaggaaccttttgttgtggagcttgatcatagagatgatggg actcaaattcagaatgtccttctagatttggtgggtcgaagcactgttccacaaatattt gtgaatggcaagcatattggtggctctgatgacctcagaaatgcagtcctgagcggtcag ctgcagaaacaactcggtacaagttga |
| A8JHA9 | UniRef cluster | -------------------------MLATRSAAFASVAGRRTLVTRAMATKLDSIRETVAKNKVVVYSKTHCPYCMKAKSSINQFLQPSQYTVIE----LDGRA---DMDEMQDALRELTGARSVPRVFVGGKFLGGGDDTAAAAANGTLKKLLQEAGAL-------------------------------- | atgctcgctactcgttctgctgccttcgcctcggtcgctggccggcggactcttgtcact cgcgctatggcaaccaagctggactcaattagggagacggttgccaagaacaaggtggtc gtgtacagcaagactcactgcccctactgcatgaaggccaagagctccatcaaccagttc ctgcagcccagccagtacaccgtcatcgagctggacggccgtgccgacatggacgagatg caggacgccctgcgcgagctgacgggggcccgctccgtgccccgcgtgttcgtgggcggg aagttcctgggcggtggcgatgacaccgccgccgccgccgccaacggcaccctgaagaag ctgctgcaggaggccggtgctctgtaa |
| A5B8K3 | UniRef cluster | -------------------------------------------------MALQKAQEMVSSNPVVVFSKTYCPFCVSVKKLLSELGATFKVVELD----TESD-----GADLQSALAGWTGQRTVPNVFIGGKHIGGCDTATALHSDGKLVPLLTEAGAITSSSNEGSSVTC-------------------- | atggcgttgcaaaaggcccaggagatggtttcttcaaatcctgtcgtcgtcttcagcaag acgtattgtccgttctgcgtgagcgtgaagaagttgttatcggagctgggagctactttc aaggtcgtcgaattggatactgaaagtgatggagctgatcttcaatcagcacttgctggg tggaccggacagcgtactgtgccaaacgtgtttatcggtggaaagcacattggtggctgt gacaccgccacagccttgcacagtgatgggaaacttgttcctttgcttactgaagctgga gctattacgtcctcctctaacgaagggagttctgttacctgttga |
| Q9ZR41 | UniRef cluster | -------------------------------------------------MSLAKAKEIVSGNPVAVFSKTYCPFCVSVKDLLSKLGATFKAVELD----SEKD-----GSEIQAALAEWTGQRTVPNVFIGRKHIGGCDATTALHREGKLLPLLTEAGAIAKTSTA-------------------------- | atgtcacttgccaaggctaaggaaattgtttctggaaatccagttgcggtcttcagcaag acgtattgtcccttctgtgttagcgtcaaggatttgttgtcgaagcttggtgctactttt aaggctgttgagttagattctgaaaaggatggaagtgagatccaggctgcactggctgag tggactggtcagcgaactgtgccaaacgtcttcataggccgaaagcacattggtggctgt gacgcgacaactgcgttgcacagggaagggaagcttcttcctctgctaaccgaggctgga gcaattgctaaaacttctacagcttag |
| A8Y1E0 | UniRef cluster | -------------------------------------------------MSKAFVDGLLQSHKVVVFSKSYCPYCHKARAALDSVNVKPDALQWIE------IDDRKDCDEIQNYLGSLTGARSVPRVFINGKFFGGGDDTAAAAKNGKLAKLLQEAGAI-------------------------------- | atgtcaaaggcattcgtcgacggactcctccaatcgcacaaagtggtggtgttcagtaaa tcgtactgtccctactgtcataaggctcgcgctgctttggactcggtcaacgtcaagccg gacgctcttcaatggatcgaaatcgacgatcgcaaggattgtgatgagatccagaactat cttggatctctcaccggagctcgctcggtcccacgtgtcttcatcaacggaaaattcttc ggtggaggtgacgacaccgcggctgccgccaaaaacggaaaactcgcgaaacttctccaa gaggctggagccatctaa |
| A7Q1G5 | UniRef cluster | ------------------------------------------MASSFGSRLEETVKKTVDENPVVVYSKTWCSYS---SEVKSLFKRLGVEPFVI----ELDEMGP-QGPQLQKVLERLTGQHTVPNVFIGGKHIGGCTDTVKLYRKGELEPLLSEASTRKTES---------------------------- | atggcttcctcgtttgggtctcgcttggaagagaccgtgaagaagacagtggatgagaac ccagttgttgtctactccaaaacctggtgttcgtattcttctgaggtcaaatctttgttc aagcgacttggtgtagaaccttttgtgattgaattggacgagatgggaccccaagggcca cagctgcagaaggtgctggaaaggcttactggacaacatactgttccaaatgtttttatt gggggcaaacacattggtggttgtacagatactgtgaagctataccggaaaggagaactt gaacctttgctatcagaagccagcaccagaaagacagagagctag |
| A8C9L0 | UniRef cluster | ------------------------------------------------MTKVDEIKLKVNGNKVVVYSKTYCPFCKKAKTALADAGLK-DYVLIE----LDELP---DGDAYQDALLEITKGRSVPRVFIGGKFVGGGDDVKKLQDTGKLKPMLKEAGAL-------------------------------- | atgacaaaagtcgacgaaatcaagcttaaagtcaacggaaacaaagtagtagtgtattcc aaaacctactgcccgttttgtaagaaagcgaaaactgccttggctgacgctggcttgaaa gactatgtgcttatcgaactggatgagttgcccgatggtgatgcttatcaagacgccctt ctggaaattacaaaggggagatcggttccaagggtcttcatcggtggcaaatttgtcgga ggtggtgatgatgtcaagaagctgcaagatactggaaaattgaagccaatgctgaaagaa gctggagctttgtga |
| A6RZ50 | UniRef cluster | -----------------------------------------------MSAAQTKADGIIADNAVAVFSKSYCPYCNATKKLLTDLKANFYS--------IELDQVD-DGSAIQSYLAEKTGQTSVPNIFIGQKHVGGNSDLQAKN-----KKDLESQLKELNAVQA-------------------------- | atgtctgctgctcaaaccaaagccgatggcatcattgccgacaacgctgttgccgtcttc agcaaatcctactgtccatactgcaacgcaaccaagaagctcttgaccgatttgaaagca aacttctactctatcgagctggatcaagttgacgacggatccgcaatccaatcctacctc gccgagaagacgggtcaaacctctgtccccaacatcttcatcggtcagaaacacgtgggt ggaaactcggatttgcaggcgaagaataagaaggatttggagagtcagttgaaggagttg aatgctgttcaggcttag |
| A7SJ69 | UniRef cluster | --------------------------------------------MSTNQAVKDFVEGEISSHKVMMFSKTYCPFCTKAKKALQKAGLQ--DFHVIE------IENRSDGGEIQDYLNKRNRSRTVPQVHINGKFIGGGTETEDLERSGKLLEMLKACGAL-------------------------------- | atgtcaacaaatcaagccgtaaaagactttgttgagggagagatctcctcacacaaggtg atgatgttctcgaaaacgtattgccccttctgcaccaaggcgaaaaaggccctccagaag gctgggcttcaagactttcacgtcatcgaaatcgagaatcgcagtgatggcggagaaatt caagattacctgaataaaaggaatagaagccgaactgttccacaagttcatataaatggg aagttcatcggaggaggcacagaaaccgaggaccttgaacgaagtggaaaattgctagag atgctgaaagcctgtggtgccttgtaa |
| A9PC68 | UniRef cluster | -------------------------------------------------MAMNKAKELVSTNPVVVFSKTSCPFCVKVKQLLNQLGAKYTTVELD----TEKD-----GGEIQSALHEWTGQRTVPNVFIGGNHIGGCDKTTGMHQEGKLVPLLADAGAVASASASA------------------------- | atggcaatgaacaaggcgaaggagctggtatccaccaatcccgtggtggttttcagcaag acatcctgtccattttgcgtcaaagtgaagcagcttctgaatcaattaggagccaaatac actactgtggaattggataccgagaaggatggaggtgaaatacaatcagcgttgcatgag tggactggacaacgcaccgtgccaaatgttttcattggtggcaaccacatcggcggctgt gacaaaaccacaggcatgcaccaggaaggaaagctggttcctctgcttgctgatgctgga gctgttgcctctgcttctgcttctgcttaa |
| A8IYH1 | UniRef cluster | ---------------------------------------------MGKAEAINEIQKAVASNKVIVYSKTYCPYCVKAKNALNQFIAG-KYTVVE----LENRA---DCDAMQDALLDITGGRSVPRVFINGKFLGGGDDTAAAASNGTLEKLLQEAGAL-------------------------------- | atgggcaaggcggaggccatcaacgagattcagaaggcggtcgccagcaacaaggtgatc gtgtacagcaaaacgtactgcccctactgcgtcaaggccaagaatgcgctgaaccaattc atcgccggcaagtacaccgtcgtcgagctggagaaccgcgccgactgcgacgccatgcag gacgcgctgctggacatcaccggcggccgctccgttccgcgcgtgttcatcaacggcaag ttcctgggtggcggcgacgacaccgcggccgccgccagcaacggcaccctggagaagctg ctgcaggaggcgggcgccttgtga |
| Q5KIY1 | UniRef cluster | ----------------------------------------------MTADVKSLVDKAIADNKVVVFSKTYCPYCKRAKSYLAEDTKD---IEILE------LDEREDGAAIQAYLKELNGQGTVPHVYINKEFIGGSSDLLKLSHEQVKQKISAAASA--------------------------------- | atgaccgccgacgttaaatctttggtcgacaaggccatcgccgataacaaggtcgtcgta ttttccaagacctactgcccttactgcaagcgggccaaatcttacctcgctgaggacact aaggacatcgagatccttgagctcgacgagcgtgaggacggcgctgccatccaggcttac ctcaaggaactcaacggccaaggcactgttccccatgtctacatcaacaaggagttcatc ggcggttccagcgacctcctcaaactctcccacgagcaggtcaagcagaagatctctgct gccgcgtccgcttag |
| Q6H628 | UniRef cluster | ---------------------MAAARAAVPIAVFLLLVLAEADPAAATRSPSAFVQNAIYSNRITIFSKTYCPYSMRAKRIFRDLKEN--PYIVE----LDLRE---DGREIQSVLLDLVGRHTVPQVFVNGQHVGGSDDTANAHSNGQLQKLLGNSQSQR------------------------------- | atggctgcggcgagagcagcggtccccatcgccgtcttcctcctcctcgtcctcgccgag gccgaccccgcggcggcgacgcgctcgccttcggccttcgtgcagaacgccatctactcc aatcgcatcaccatcttctctaaaacctactgcccgtactctatgcgtgctaagcgtata tttagagatctcaaggagaatccttatattgttgaactagatctcagagaggatggtaga gaaattcaaagtgttcttctagacttagttggccgtcatactgtgccacaggtgttcgtg aatggccagcacgttggtggctcagatgatacagcaaatgctcattctaatggacagctt cagaaacttcttggaaatagccaatcacagcgatga |
| A3A9K2 | UniRef cluster | ---------------------MAAARAAVPIAVFLLLVLAEADPAAATRSPSAFVQNAIYSNRITIFSKTYCPYSMRAKRIFRDLKEN--PYIVE----LDLRE---DGREIQSVLLDLVGRHTVPQVFVNGQHVGGSDDTANAHSNGQLQKLLGNSQSQR------------------------------- | atggctgcggcgagagcagcggtccccatcgccgtcttcctcctcctcgtcctcgccgag gccgaccccgcggcggcgacgcgctcgccttcggccttcgtgcagaacgccatctactcc aatcgcatcaccatcttctctaaaacctactgcccgtactctatgcgtgctaagcgtata tttagagatctcaaggagaatccttatattgttgaactagatctcagagaggatggtaga gaaattcaaagtgttcttctagacttagttggccgtcatactgtgccacaggtgttcgtg aatggccagcacgttggtggctcagatgatacagcaaatgctcattctaatggacagctt cagaaacttcttggaaatagccaatcacagcgatga |
| A2X7T2 | UniRef cluster | ---------------------MAAARAAVPIAVFLLLVLAEADPAAATRSPSAFVQNAIYSNRITIFSKTYCPYSMRAKRIFRDLKEN--PYIVE----LDLRE---DGREIQSVLLDLVGRHTVPQVFVNGQHVGGSDDTANAHSNGQLQKLLGNSQSQR------------------------------- | atggctgcggcgagagcagcggtccccatcgccgtcttcctcctcctcgtcctcgccgag gccgaccccgcggcggcgacgcgctcgccttcggccttcgtgcagaacgccatctactcc aatcgcatcaccatcttctctaaaacctactgcccgtactctatgcgtgctaagcgtata tttagagatctcaaggagaatccttatattgttgaactagatctcagagaggatggtaga gaaattcaaagtgttcttctagacttagttggccgtcatactgtgccacaggtgttcgtg aatggccagcacgttggtggctcagatgatacagcaaatgctcattctaatggacagctt cagaaacttcttggaaatagccaatcacagcgatga |
| Q9UTI2 | UniRef cluster | ----------------------------------------------MTSIAKAFVEKAISNNPVTVFSKSFCPFCKAAKNTLTKYSAP---YKAYE------LDKIENGSDIQAYLHEKTKQSTVPSIFFRNQFIGGNSDLNKLRSSGTLTKMIAELKENKSSIL--------------------------- | atgacttctatagcaaaagcttttgttgaaaaggcgatctcaaacaatccggttactgtc tttagtaaatcgttctgtcctttctgcaaagcagccaaaaatactcttacaaagtactct gctccgtataaggcttatgagttagataaaatagaaaatggatctgacatccaagcttat ttacatgaaaagaccaaacagtctacagtacccagcatattctttcgcaaccaattcatt ggaggcaattccgatttgaacaaacttcgtagctcaggcactttgacgaagatgatagca gagcttaaggaaaacaaatcttcaattttatga |
| Q54GP8 | UniRef cluster | ---------------------------------------------------MDKVKALIKAHKLIIFSKTTCPYCISVKDLFKKLKVVPFVVELD----LESD-----GSELQSAAGQISGVRTVPQVFINEKFIGGCDATTKLHSQGKLIPLLQEAGFL-------------------------------- | atggataaagttaaagcattaatcaaagctcataaattaattattttctcaaaaactaca tgtccatactgcatttcagttaaagatttattcaaaaaattaaaggttgtaccatttgtt gttgaattagatttagaatctgatggttctgaattacaatcagcagctggtcaaatctct ggtgttagaactgtaccacaagtttttattaatgaaaagtttattggtggttgtgatgct accactaaattacattctcaaggtaaattaattccattactccaagaagcaggtttttta taa |
| Q3UQ95 | UniRef cluster | -----------------------------------MGNSTSSFWGKSTTTPVNQIQETISNNCVVIFSKTSCSYCSMAKKIFHDMNV--NYKAVE----LDMLE---YGNQFQDALHKMTGERTVPRIFVNGRFIGGAADTHRLHKEGKLLPLVHQCYLKKKQEERH------------------------- | atgggaaacagcacatcgtcgttttgggggaagtctacaactactcctgtgaaccagatc caagaaacaatttctaacaattgtgtggtgatcttctcaaaaacatcctgctcttactgt tccatggccaagaagattttccatgacatgaatgtcaactacaaggctgtggagttggat atgctggaatatggcaaccagtttcaagatgcgcttcacaagatgactggggaaagaacc gttcccaggatatttgtcaatggacgatttattggaggcgcagcggacactcacaggctt cacaaagaagggaaattgctgcctctggttcatcagtgttatttaaaaaaaaaacaagag gaaagacattga |
| A9PD09 | UniRef cluster | --------------------------------MGSLLSSSIKMSKQELDAALKKAMELASSAPVVVFSKTYCGYCNRVKQLLTQVGATYKVVELD----EISD-----GSQLQSALAQWTGRGTVPNVFIGGKNIGGCDTVVEKHQRNELLPLLQDAAATAKNSAQL------------------------- | atgggttcgctgttaagttcttcaatcaagatgagcaagcaagaacttgatgctgcgctt aaaaaggccatggaactcgcctcctctgctcctgtcgttgttttcagcaaaacctactgt ggctattgcaatagggtgaagcagctgctgacacaggtaggagcaacttacaaagtcgtt gagctggatgagataagtgatggatctcaacttcaatcagcactagcacagtggactggg cgagggacagtgcctaatgtgttcatcggagggaaaaacattggtggttgcgacaccgtt gtggagaagcaccaacgcaacgaactcttgcctcttctccaagatgctgctgccacagct aaaaactctgcccagctttga |
| Q5PSJ1 | UniRef cluster | --------------------------------MGSLLSSSIKMSKQELDAALKKAKELASSAPVVVFSKTYCGYCNRVKQLLTQVGASYKVVELD----ELSD-----GSQLQSALAHWTGRGTVPNVFIGGKQIGGCDTVVEKHQRNELLPLLQDAAATAKTSAQL------------------------- | atgggttcgctgttaagttcttcaatcaagatgagcaagcaagaacttgatgcagcgcta aaaaaggctaaggaactcgcctcctctgctcctgtcgttgttttcagcaaaacctactgc ggctattgcaatagggtgaagcagctgctgacacaggtaggagcatcttacaaggtcgtt gagctggatgagctaagtgatggatctcaacttcaatcagcactagctcactggactggg cgagggacagtgcctaatgtgttcatcggagggaaacaaatcggtggttgcgacaccgtt gtggagaagcaccaacgcaacgaactcttgcctcttctccaagatgctgctgccaccgct aaaacctctgcccagctttga |
| Q4FYP1 | UniRef cluster | -------------------------------------MFSSRFLYRSSSTMPATVAELITQHKVVVFSWVHCPYCSRAKEILKSLAKDIQVYECDQ---------MDNGEELRTQILQAYNHDTVPAIFINGEFIGGCSDLQAIQKSGELAAKLA------------------------------------- | atgttctccagccgttttctctaccgatcctcttcaacgatgcccgctaccgtcgccgag ctcatcacccaacacaaggtggtggttttctcgtgggtgcactgcccgtattgctctcgc gccaaggaaatcctcaagtcgctcgcaaaggatatacaagtttacgagtgcgaccagatg gacaacggcgaggaacttcgtacacagattctgcaggcgtacaatcacgacacagtgccg gcgatcttcatcaatggtgagttcattggcgggtgcagcgacttgcaggccatccagaag agcggcgaactggctgcgaagcttgcgtaa |
| Q6BQC7 | UniRef cluster | ----------------------------------------------MSKEYIERAQELVNNHPYLMLSKSWCPDCHYTYEIWNQYNVKEKIYIIELD---KFE-DQNEAEELEKAFTEIAGRKWVPTIFFHGKILGTEEDLKRWTKEGKLSEIFKDSHLIN------------------------------- | atgtctaaggaatatatcgaaagggcacaagagttggtcaataaccatccctatttaatg ttatctaagtcatggtgtcctgattgtcattatacctacgaaatttggaaccaatacaat gttaaagaaaaaatttacatcatcgagttagataagtttgaagatcaaaacgaagcagaa gaattagagaaagcttttacagaaatcgctggtagaaaatgggttccaacgatctttttc catggaaaaattctaggcactgaagaagatttgaaaaggtggacaaaggaaggtaaattg tccgagatctttaaagattctcatcttattaattaa |
| A9TU36 | UniRef cluster | ------------------------------------------MAAGSGSDLELWIKKKNSSEPVVVYSKTYCPYYRYCMRVKKLFSTLGYDFEVI----ELDAGGQ-LG--LQDALERVSGQYTVPNVFIGGKHIGGCDDTVALHSKGQLEPLLQAAGANRS------------------------------ | atggcagcaggttcggggagcgacctggaattatggatcaagaagaagaattcctctgaa cctgtggtcgtatattccaaaacttattgcccgtactacaggtattgcatgcgcgtgaag aagctttttagcactctgggctacgattttgaagtaattgaacttgatgcaggcgggcaa cttggtttacaagatgcgttggagcgagttagtgggcaatacactgttccgaacgttttc attggtgggaaacatattggtggctgtgatgacacggtggccttgcattctaaaggccag ctggagcccttgcttcaggcagctggtgcaaacaggagttga |
| A7SXB8 | UniRef cluster | ---------------------------------------------MAAKTALHFVRSVTRSNNIVVFSKTACSFSIMAKKLLRDVGVS-EMVVYE----LEQRE---DGHFIQDALKELTGRGTVPNVFVKGQSIGGGMETAELYQSGKLKQLLQDHGLLDENQ---------------------------- | atggcggcgaagacggcgttacactttgttcgttctgtaacgcgaagtaataacatcgta gtgttctctaagactgcttgttcattttcaattatggctaagaagttactaagggatgtt ggagtatcagagatggtagtttatgaacttgagcaacgggaagacgggcattttatacag gatgccttaaaagagttaacaggaagaggaacggtgcccaatgtcttcgtcaaagggcaa agcattggcggagggatggaaacagctgaattatatcaatctggcaaattaaaacagtta ctacaagatcatgggttgcttgatgaaaatcagtaa |
| Q5CGG8 | UniRef cluster | -----------------------------------------------MNSIKLLVESFISSGDICVISKSYCPYCIKAINSLK-SAG-Y-SPLVMQ------IDGRVDTKEIQDYCRELTGSGTVPRVFVKGRFIGGCDDTLKLLEDGSLSSFVETI----------------------------------- | atgaactcaataaagttattagtagaatcattcataagttcaggagatatctgtgttata tcgaagagttattgcccgtactgcataaaagcaatcaatagcttgaaaagcgctggatat tcacctttggttatgcaaatcgacggtagagtagatactaaagaaattcaagattactgt agggagctaactggaagtggaactgtcccaagagtatttgttaaagggaggtttatagga ggatgtgatgataccctgaagctgttagaagacggctcattgagtagctttgttgaaacg atctag |
| Q19297 | UniRef cluster | ------------------MLRILTVVLALVTIATVHAELSKKKEDKTLKDLEDKIVNDVMTHKVMVYSKTYCPWSKRLKAILANYEID-DMKIVE----LDRSN---QTEEMQEILKKYSGRTTVPQLFISGKFVGGHDETKAIEEKGELRPLLEKAHALFTNRVPVPDNGA-------------------- | atgctcaggatactcacagtcgtcttagcactcgtaacgattgctactgttcacgcagaa ttatccaagaagaaggaagataaaacgttgaaggatttggaggataaaattgttaatgat gttatgacgcataaagttatggtatacagtaaaacctattgcccatggagcaaacgtctg aaggctattcttgccaactatgaaattgatgacatgaagattgttgagcttgatcgatca aatcagactgaggaaatgcaggaaattctcaaaaagtactctggaagaacgacagttccc caattattcatcagtgggaagttcgtgggtggtcacgatgagactaaggcaatcgaggag aaaggagagctgaggccacttttggagaaagctcacgccttgttcactaaccgagtgcct gtccctgacaacggtgcgtga |
| A7NW71 | UniRef cluster | --------------------------------------------------MERAVARLASERPVVIFSKSSCCMCHTIKTLFSDFGVNPAVHELD----EMPRG----REIEQALARLG-CNPTVPTVFIGGERVGGTNEIMTLHLNRSLIPMLKRAGALWV------------------------------ | atggagagggcagtggcaaggttggcatcagagaggccagtggtgatattcagcaagagc tcatgctgcatgtgccacaccatcaagacccttttctcggactttggagttaacccggcc gtccacgagctggatgaaatgccgagagggcgtgaaattgagcaggccttggccaggctt gggtgcaaccccacggtgccaacagtgttcattggtggtgaacgggtgggtggaaccaat gagatcatgacccttcacctcaatagatccttaatccccatgctcaagagggctggtgcc ttatgggtctga |
| P12309 | UniRef cluster | -------------------------------------------------MAQAFVNSKIQPGKVVVFIKPTCPFCRKTQELLSQLPFKEGLLEFVD------ITATSDTNEIQDYLQQLTGARTVPRVFIGKECIGGCTDLESMHKRGELLTRLQQIGALK------------------------------- | atggctcaagcatttgtgaacagcaaaatccagcctgggaaggtggtagttttcatcaag cccacctgccccttctgcagaaagacacaggagctcctcagccaattgcccttcaaagaa gggcttctggaatttgtcgatattacagccaccagtgacaccaacgagattcaagattat ctgcaacagctcacaggagccagaacggtacctcgggtctttatcggtaaagagtgtata ggtggatgcactgatctagaaagtatgcacaagagaggggagctcttgacccgcctgcag caaattggagctctgaaataa |
| Q9ESH6 | UniRef cluster | -------------------------------------------------MAQEFVNCKIQSGKVVVFIKPTCPYCRKTQEILSQLPFKRGLLEFVD------ITATNNTNAIQDYLQQLTGARTVPRVFIGKDCIGGCSDLLSMQQNGELTARLKQIGALQL------------------------------ | atggctcaggagtttgtgaactgcaagattcagtctggaaaggtggtcgtgttcatcaaa cccacctgcccctattgcagaaagactcaagaaatcctcagtcaactgcctttcaaacgt ggtctcctggaatttgtggacatcacagctactaacaacaccaatgcgattcaagattat ttacaacagctcaccggagcaagaacagttcctcgggtcttcataggtaaagactgcata ggcggatgcagtgatctactctccatgcaacagaatggggagctgacggcccggctgaag cagattggagctctgcagttatga |
| Q4V9D0 | UniRef cluster | --------------------------------------------------MAAFVKAQIKNGKVVVFLKPPCPYCVLAKDVLSKYKFKAGHLELVD------ISARSDMDSIQDYLQQITGARTVPRVFIGEDCVGGGSDVEGLDRSGKLEGMLQAIGCLQ------------------------------- | atggcagcgtttgttaaagcccaaattaagaacgggaaagtagttgtatttttgaaacca ccctgcccttactgcgtactcgcaaaggatgttttatcgaagtataaattcaaagctgga catttggaattggtcgatataagcgcacgcagcgacatggacagcattcaggactatctg caacagatcacgggcgcgcgcactgttcctcgagttttcataggagaagattgtgttgga ggaggaagtgatgttgagggtcttgaccgttctggaaaactggaaggcatgttgcaggcc atcggatgtctgcagtga |
| P10575 | UniRef cluster | -------------------------------------------------MAQAFVNSKIQPGKVVVFIKPTCPYCRKTQELLSQLPFKQGLLEFVD------ITAAGNISEIQDYLQQLTGARTVPRVFIGQECIGGCTDLVNMHERGELLTRLKQMGALQ------------------------------- | atggctcaagcgttcgtcaacagcaagatccagcctgggaaggtggtcgtgttcatcaag cccacctgcccctactgcagaaagactcaggagcttctcagccaactgcccttcaaacaa gggcttttggaatttgtcgatattacagccgccggtaacatcagtgagattcaagattac ttgcagcagctcaccggagccagaacggtacctcgggtcttcatcggtcaagagtgcata ggtggatgcacagatctagtaaatatgcacgagagaggggaactgttgacacggctaaag caaatgggagctctgcaataa |
| Q9D6F8 | UniRef cluster | -------------------------------------------------MAQEFVNCKIQSGKVVVFIKPTCPYCRETQEILSQLPFKQGLLEFVD------ITATNNTSAIQDYLQQLTGARTVPRVFIGKDSIGGCSDLISMQQTGELMTRLKQIGALQL------------------------------ | atggctcaggagtttgtgaactgcaagatccagtctgggaaggtggtcgtgttcatcaag cccacctgcccctactgcagagagacccaagaaatcctcagtcaactgcctttcaaacaa ggtcttctggagtttgtggacatcacagccactaacaacaccagtgcgattcaagattat ttacaacagctcaccggagcgagaacagttcctcgggtcttcataggtaaagacagcata ggcggatgcagtgatctaatctccatgcaacagactggggagctgatgactcggctgaag cagattggagctctgcagttataa |
| Q7RDW5 | UniRef cluster | -------------------------------------------MGSTYEAIKKFVHKIIDENKIAVFSKTECPYCVKAISILKGYNPN---VYVEQ------IEKXPNMADIQSYFKELTGKSSVPRIFINKEFVGGCDDLVKENETGKLQERLKSIGMIN------------------------------- | atgggatccacatacgaagctattaaaaaatttgtacataaaatcatcgatgaaaataaa attgctgtattttcaaaaactgaatgcccatattgcgttaaagcaatatccattttaaaa ggatataatcctaatgtgtatgtagagcaaattgagaaamaccctaatatggcagatatc caatcatatttcaaggaattaactggaaaaagttccgtaccaagaatatttattaacaag gaatttgtcggtggatgtgatgacttggtcaaggaaaacgaaaccggaaaacttcaagag agactcaagagcatcggratgattaactag |
| Q9QUH0 | UniRef cluster | -------------------------------------------------MAQEFVNCKIQSGKVVVFIKPTCPYCRKTQEILSQLPFKQGLLEFVD------ITATNNTSAIQDYLQQLTGARTVPRVFIGKDCIGGCSDLISMQQTGELMTRLKQIGALQL------------------------------ | atggctcaggagtttgtgaactgcaagatccagtctgggaaggtggtcgtgttcatcaag cccacctgcccctactgcagaaagacccaagaaatcctcagtcaactgcctttcaaacaa ggtcttctggagtttgtggacatcacagccactaacaacaccagtgcgattcaagattat ttacaacagctcaccggagcgagaacagttcctcgggtcttcataggtaaagactgcata ggcggatgcagtgatctaatctccatgcaacagactggggagctgatgactcggctgaag cagattggagctctgcagttataa |
| Q3U6L3 | UniRef cluster | -------------------------------------------------MAQEFVNCKIQSGKVVVFIKPTCPYCRKTQEILSQLPFKQGLLEFVD------ITATNNTSAIQDYLQQLTGARTVPRVFIGKDCIGGCSDLISMQQTGELMTRLKQIGALQL------------------------------ | atggctcaggagtttgtgaactgcaagatccagtctgggaaggtggtcgtgttcatcaag cccacctgcccctactgcagaaagacccaagaaatcctcagtcaactgcctttcaaacaa ggtcttctggagtttgtggacatcacagccactaacaacaccagtgcgattcaagattat ttacaacagctcaccggagcgagaacagttcctcgggtcttcataggtaaagactgcata ggcggatgcagtgatctaatctccatgcaacagactggggagctgatgactcggctgaag cagattggagctctgcagttataa |
| Q9BH70 | UniRef cluster | MSSVKYLVLFTLVCVVSLFQPSQCRLDYHFFSDFLSKYSFTLPFKMAEKTPKDWVDSLVKKHKVVVFSKSYCPYCTRAKDALKKLNLH--DLHVEE------LDSNPNMDQVQDYLNQLTGARSVPRVFVNGRFYGDSTKTVSDVESGKFMEHYKKTDL--------------------------------- | atgagttcagtgaagtatttagttttatttactttggtttgtgttgtgagtttatttcag ccttcccagtgtcgtctggactatcatttcttctctgatttcctgagtaaatattccttc acgctgccgtttaaaatggccgaaaaaacgcccaaggactgggtcgacagcctcgtcaaa aaacacaaagtcgtcgtcttctccaagtcgtactgcccctactgtaccagagccaaagac gcactcaaaaaactcaacctacacgatctccacgtggaggagttggattctaatccgaac atggaccaggtccaagattacttaaaccaactcacaggagcgaggagtgtcccgcgtgtg tttgtgaatggtcgtttctacggagattccactaaaactgtttcagacgtcgagagtggg aagttcatggaacattacaagaaaactgacttataa |
| O23421 | UniRef cluster | ---------------------------------------------------MENLQKMISEKSVVIFSKNSCCMSHTIKTLFLDLGVNPTIYELD----EISRG----KEIEHALAQLG-CSPTVPVVFIGGQLVGGANQVMSLHLNRSLVPMLKRAGALWL------------------------------ | atggagaatctacaaaagatgatctccgagaagtcggtagtgatctttagcaagaactct tgctgcatgtctcacacaatcaagactctcttcttagaccttggcgtgaacccgacgatc tatgaactcgatgagattagcagaggaaaggagatagagcatgcattggctcagctcggg tgcagcccgacagtgccagtggtgttcataggagggcagcttgttggtggagccaatcaa gtcatgagtctccatctcaaccgctcccttgttccaatgcttaagcgcgctggagcttta tggctttga |
| P12864 | UniRef cluster | --------------------------------------------------AQEFVNSKIQPGKVVVFIKPTCPYCRKTQEILSQLPFKQGLLEFVD------ITATSDMSEIQDYLQQLTGARTVPRVFLGKDCIGGCSDLIAMQEKGELLARLKEMGALRQ------------------------------ |  |
| P35754 | UniRef cluster | -------------------------------------------------MAQEFVNCKIQPGKVVVFIKPTCPYCRRAQEILSQLPIKQGLLEFVD------ITATNHTNEIQDYLQQLTGARTVPRVFIGKDCIGGCSDLVSLQQSGELLTRLKQIGALQ------------------------------- | atggctcaagagtttgtgaactgcaaaatccagcctgggaaggtggttgtgttcatcaag cccacctgcccgtactgcaggagggcccaagagatcctcagtcaattgcccatcaaacaa gggcttctggaatttgtcgatatcacagccaccaaccacactaacgagattcaagattat ttgcaacagctcacgggagcaagaacggtgcctcgagtctttatcggtaaagattgtata ggcggatgcagtgatctagtctctttgcaacagagtggggaactcgtgacgcggctaaag cagattggagctctgcagtaa |
| A8QBM8 | UniRef cluster | -----------------------------------MGFLVSKHVPQDMSEVQQFIDELIALKKIVVISKSWCIYCKRTRKALASYPLEGDAMEWID------INKRSDGKEILDYMEQITGSRRVPRIFIGGEFFGGCAEICAAKRDGILERKLTAIGAI-------------------------------- | atgggcttcttggtttccaaacatgttccgcaggatatgagtgaggtacaacaatttatt gacgaattgattgcactgaaaaaaatagtagtaatatcaaagagttggtgcatatattgt aaacgaacacgtaaagcgcttgcctcgtatcctctcgaaggagatgcaatggaatggatc gacatcaacaaaaggtctgatggaaaagaaattttggattatatggaacagataactgga tcaagaagagtgccgcgaattttcatcggcggtgaattttttggaggttgcgctgaaatt tgtgcagcaaaaagggacggtatcttagaacgcaagcttactgcaattggggccatatga |
| Q20CD1 | UniRef cluster | ---------------------------------------------------MDVVKGLVQERPVVIFSKANCPVSHSMRQLISGFGANPTVYELD----QMPNG----REIERVLQMMG-RKPTVPSMFIGGNLVGGPNDLISLQVQGKLVQMLMDAGAIWIWKRN-------------------------- | atggacgttgtgaagggtttagtacaagaaaggccggtggtcatcttcagtaaggcaaac tgtcctgtaagccactcgatgagacagctcattagtgggtttggcgctaatcctactgta tatgaactcgatcaaatgccaaatggacgggaaattgagagagttttacagatgatgggg cgtaaacccactgtaccatctatgttcattggtggaaacttggtaggtggacctaatgac ctgatcagccttcaggtgcaaggcaagcttgtacaaatgctgatggatgccggagctatt tggatttggaagaggaactag |
| O23419 | UniRef cluster | ---------------------------------------------------MDKLQKMISEKSVVIFSKNSCCMSHTIKTLFIDFGVNPTIYELD----EINRG----KEIEQALAQLG-CSPTVPVVFIGGQLVGGANQVMSLHLNRSLVPMLKRVGALWL------------------------------ | atggataagctacagaagatgatctccgagaagtcggtagtgatctttagcaaaaactca tgttgcatgtctcacactatcaagactctcttcatagactttggcgtgaatccaacgatc tatgagctagatgagatcaacagaggaaaggagatagagcaagcattggctcagcttggc tgcagcccaaccgtgcctgtggtgtttattggagggcagcttgttggtggagccaatcaa gtcatgagtctccatctcaatcgctctctggttcctatgctaaagagggttggagcacta tggctttga |
| A9V2E4 | UniRef cluster | --------------------------------MGQYLSAGSSDNTTNNMSANAIVQSAITNNDLMVFSKSYCPFCTQAKRELSQAGLEYNVIELDQG-AVSYDGQEAEGSDVQGIIKSQYKHRTVPAVFVKGKLLGGCDDTVAAIRNGKLKEMLA------------------------------------- | atgggccagtacttgtccgccggttcatcggataacaccaccaacaacatgagtgccaac gccatcgtccaaagcgccattaccaacaacgacctcatggtcttctccaagagctactgc cccttttgcactcaagccaagcgcgagctctcccaggctggcctcgagtacaatgtcatc gagcttgatcagggcgccgtatcatacgacggtcaggaggcagagggctccgacgtgcag ggcatcatcaaatcccagtacaagcaccgcacggttcccgccgtctttgtcaaaggcaaa ctgctgggcggatgcgatgacacggtcgctgccatccgcaacggcaagctgaaagagatg ctggcctga |
| Q387B5 | UniRef cluster | ---------------------------------------------------MPSIASMIKGNKVVVFSWVTCPYCVRAEKLLHARTKDITVHYVDK---------MSEGEQLRGEIYQAYKHETVPAIFINGNFIGGCSDLEALDKEGKLDGLLS------------------------------------- | atgccctctatcgcttcgatgattaaagggaataaagtggttgttttctcatgggtcact tgcccctactgcgtccgagcagagaagttacttcacgcacgcactaaggatattaccgta cattatgtggataagatgagcgaaggagaacaactgcgcggtgagatttaccaagcatac aaacacgaaacggttcccgccatatttattaatggaaatttcattggcggttgcagcgat ttggaggcccttgacaaagagggaaaactcgatggtttgctgagctaa |
| O23417 | UniRef cluster | ---------------------------------------------------MEKIQKMISEKSVVIFSNNSCCMSHTIKTLFLDLGVNPTIYELD----EINRG----KEIEYALAQLG-CSPTVPVVFIGGQLVGGANQVMSLHLNRSLIPMLKRFGALWL------------------------------ | atggagaagatacaaaagatgatctccgagaagtcggtagtaatatttagcaataactct tgttgcatgtcacacacaatcaagactctcttcttagaccttggcgtgaacccgacaatc tatgagctagacgagatcaacagaggaaaagagatagagtatgcattggctcagcttggc tgcagcccgactgtgccagtggtgttcataggagggcagcttgttggtggagccaatcaa gtcatgagtctccatctcaaccgttctctcattccaatgcttaaacgctttggggcttta tggctttga |
| Q6NLU2 | UniRef cluster | ---------------------------------------------------MEKLQKMTSEKSLVIFSKNSCCMSHTIKTLFLDLGVNPTIYELD----EINRG----KEIEQALAQLG-CSPTVPVVFIGGQLVGGANQVMSLHLNRSLIPMLKRVGALWL------------------------------ | atggagaagctacagaagatgacctcggagaagtcgttagtgatatttagcaaaaactca tgctgcatgtcgcacacaatcaagactctcttcttagaccttggcgtaaatccgacgatt tatgagctagatgagatcaacagaggaaaagagatagagcaagcattggctcagcttggc tgcagcccgaccgtgccagtggtgttcataggagggcagcttgtcggtggagccaatcaa gtcatgagtctccatctcaaccgttctctcattccaatgcttaaacgcgttggggcgtta tggctttga |
| O23420 | UniRef cluster | ---------------------------------------------------MENLQKMISEKSVVIFSKNSCCMSHTIKTLFLDFGVNPTIYELD----EINIG----REIEQALAQLG-CSPTVPVVFIGGQLVGGANQVMSLHLNRSLVPMLKRAGALWL------------------------------ | atggagaacctacagaagatgatctctgagaagtcggtagtaatttttagcaagaactca tgctgcatgtctcatacaattaagactctcttcttagactttggcgtgaacccgactatc tatgagctcgacgagatcaacataggaagggagatagagcaagcattggctcagctcgga tgcagcccgaccgttccggtggtgttcattggagggcagcttgttggtggagccaatcaa gtcatgagtctccatctcaaccgctcccttgttcctatgcttaaacgtgctggagcatta tggctttaa |
| Q9SA68 | UniRef cluster | ---------------------------------------------------MEKISNLLEDKPVVIFSKTSCCMSHSIKSLISGYGANSTVYELD----EMSNG----PEIERALVELG-CKPTVPAVFIGQELVGGANQLMSLQVRNQLASLLRRAGAIWI------------------------------ | atggagaagatatcaaatttgttagaagacaagcccgtggtgatattcagcaagacgtcc tgctgtatgagtcactcgatcaagtcgcttatatctggttacggtgcgaattcaacagtg tatgagctagacgaaatgtctaatggaccagagatcgaacgagcacttgtagagcttggg tgcaaaccgactgtgccagctgtctttatagggcaagagctcgtaggtggtgcaaatcaa cttatgtctcttcaagtcaggaaccaactagcttcgttgctccgaagagctggagccata tggatttaa |
| A7AQH4 | UniRef cluster | -----------------------MILRNLGIFTTWIRDILPSSNNMRGSDISHWVNESINKSKVVVFSKTTCPYCIKANGILN-SVAPN-DLTIIQ------LDDNPDRAEIMEYFRETTGAATVPRVFIGGKFFGDCSKTVAANESGELKKVLEEAGCQ-------------------------------- | atgatattgcgaaatctcggtatttttaccacctggattcgtgacatattgccatcatcc aacaacatgaggggttctgacatatctcactgggtcaatgaatccattaacaagagcaag gtggttgtcttttcgaagactacctgcccatactgcattaaggcaaatggcatacttaat tcggtagctccaaacgatttgactattattcaacttgacgacaacccagaccgtgctgag attatggagtatttcagggagaccactggtgctgctacagttcctcgtgtattcattggt ggcaagttctttggtgactgcagtaaaacggtcgctgctaacgagtctggcgagttgaag aaggtattggaggaagctggttgccagtga |
| Q20CD3 | UniRef cluster | ---------------------------------------------------MDTVNRLVEEKALVIFSRSSCCISHSVLQLISSYGANATVYELD----DMSNG----QEVDKALQKLG-LRPGVPAVFIGQKLVGGAKEIISLQIQGKLMPMLKEAGAIWV------------------------------ | atggatactgtgaaccgattagtggaagagaaggcgttggtaatctttagtcgaagttct tgctgcataagccactcggtattgcaactcataagcagttatggagccaatgcaacagta tacgagctagatgacatgtctaatggccaagaagtagataaagctcttcaaaaactagga cttaggccgggtgttcctgctgtcttcataggccaaaagttagtcggtggcgctaaagag attattagcctacagatccaaggaaaacttatgccaatgctcaaggaagcaggagctata tgggtttag |
| Q20CC9 | UniRef cluster | ---------------------------------------------------MEAVNRLVEEKALVIFTRSSCCISHSVMQLVSSYGANATVYELD----EMSNG----QEVDKALQGLG-LRPSIPAVFIGQKLVGGAKEIISLQVQGKLMPMLKEADKKKDAIRRNKQDGRIDEATSNVSSNAVVYAFGLL | atggaggctgtgaaccgattagtggaagaaaaggcgttggttatctttactcggagttct tgctgtataagccactcagtaatgcaactcgtaagcagctatggagcgaatgcaacagtg tacgagctagatgagatgtcaaatggtcaagaagtagataaagcacttcaaggactaggg cttaggccgagtattcctgctgttttcataggccaaaagttagttggtggtgctaaagag attattagcctgcaagtccaaggaaaacttatgccaatgctaaaggaggcagataagaag aaagatgctattagaagaaacaagcaggatggaaggatagatgaagctactagtaatgta agcagtaatgcagtagtctacgcttttggtttgttgtaa |
| A7PTY4 | UniRef cluster | ---------------------------------------------------MDKVTRLASEKGVVIFSKSSCCLCYAVNILFQELGVTPTVHEID----QDPDG----REMEKALLRLG-CNAPVPAVFIGGKLVGSTNEVMSRHLSGSLIPLLKPYQQTLS------------------------------ | atggataaggtgacgagattggcttcagagaaaggggtggtgatcttcagcaagagctca tgctgcctgtgctatgctgtcaacattctgtttcaagaacttggggtcactcccacggtt catgaaatcgaccaagaccccgatggaagggaaatggagaaagccctcttgaggctggga tgcaatgctcctgtcccagctgtgttcataggtggaaagctcgtgggatccaccaacgaa gtcatgtcccgtcacctaagcggctccctcattcccctgctgaagccatatcagcagact ttatcttaa |
| B0D132 | UniRef cluster | --------------------------------------MSIYAAGEIDLDWERETRFLNKQYPIVVFSKTYCPYSKRAKELLAAYNIQPTPKIVE----VDMRD---DNNVIKLLLSRLTHHSTFPNILIQGKSIGGSDDLIALHNDRTLAKMLERAGVTIQSDLEF------------------------- | atgtctatatacgcggccggagaaatagacttggattgggagagagagaccaggtttttg aacaaacaatatcctatcgttgtcttcagcaagacgtactgcccgtactccaaacgagcc aaggagctccttgccgcgtataatattcaaccaacaccaaagatcgtcgaagttgatatg agagacgacaacaacgtgataaagctcctcctaagccgcctgacgcatcactcgacgttc ccaaatattcttatacaaggcaagtcaattggaggttcagacgacctcattgcgcttcac aacgaccgaacacttgcgaaaatgctggaacgtgctggtgtaacgatacagtcggacctc gaattctaa |
| Q9LYC8 | UniRef cluster | ---------------------------------------------------MESVRSLVEDKPVVIFSKSSCCMSHSIQTLISGFGAKMTVYELD----QFSNG----QEIEKALVQMG-CKPSVPAVFIGQQFIGGANQVMTLQVKNQLAAMLRRAGAIWV------------------------------ | atggaaagcgttagaagtttagttgaagacaaaccagtggtgatattcagcaaaagctct tgctgcatgagccactcaattcaaacactgatctcagggtttggggcaaagatgacggtc tacgagctagaccaattctcaaacggtcaggagatcgagaaggcattggtacagatgggg tgtaaacccagtgtaccagctgtgttcatagggcaacaattcatcggtggtgctaaccaa gtaatgactcttcaggtcaagaaccagctagccgcaatgctaagaagagccggagccata tgggtgtaa |
| P79764 | UniRef cluster | -------------------------------------------------MVDSFVQSKLRDNKVTLFVKGSCPYCKNAIVLLKEFNFLPGCLEVVD------ITG---MDDIQDYFQKTTGQRTVPRVFIGTKCIGGFSDLQKMEQ--QLPMMLRQIGALV------------------------------- | atggtcgattcttttgtacagagcaaactcagagataataaagttacccttttcgtgaag ggatcctgcccttactgcaagaatgccatagtattgctaaaggaattcaactttctgcca ggatgcctggaagtggttgatatcaccgggatggatgacatccaggattacttccagaaa acaacagggcagcgaaccgtccctcgtgtgtttattggaacaaaatgcattggaggattt tcagatctgcagaagatggaacagcagctccccatgatgctacgtcaaattggtgctctg gtgtag |
| A8QBN0 | UniRef cluster | -----------------------------------MGFLVSKHVP-----------------QIVVISKSWCIYCKRTRKALASYPLEGDAMEWID------INKRSDGKEILDYMEQITGSRRVPRIFIGGEFFGGCAEICAAKRDGILERKLTAIGAI-------------------------------- | atgggcttcttggtttccaaacatgttccgcagatagtagtaatatcaaagagttggtgc atatattgtaaacgaacacgtaaagcgcttgcctcgtatcctctcgaaggagatgcaatg gaatggatcgacatcaacaaaaggtctgatggaaaagaaattttggattatatggaacag ataactggatcaagaagagtgccgcgaattttcatcggcggtgaattttttggaggttgc gctgaaatttgtgcagcaaaaagggacggtatcttagaacgcaagcttactgcaattggg gccatatga |
| A5DH51 | UniRef cluster | -----------MVPQFSTSCQMTDKFHAITPQFFSESENFRCHNNYSMTQFEEKARDLIKKHEYLMLSKSWCPDCHYAYKVWDLYGVRDKIHIIEFD---KIP-DQTEAKSLEDAFVALAGIKWVPTLFFHGSYFGTEKDLKRWEAEGKLQEIFKKEGLIE------------------------------- | atggtccctcaattttcaacatcatgccagatgactgacaaatttcacgccattacacct cagtttttctcagaatcagaaaactttcgatgccacaacaactactccatgactcaattt gaagagaaagcccgcgatcttattaaaaaacatgaatatttgatgctatcgaagtcatgg tgcccggactgtcattatgcttataaagtttgggatctgtatggtgtaagagacaaaatc cacattattgagtttgacaagatccctgatcagaccgaagctaaatctttagaggatgct tttgttgctttggctggcataaagtgggtccctacattgttttttcacggatcctatttt ggaacagagaaggacttaaaaagatgggaggccgaaggcaagttgcaagagattttcaag aaggagggcttgatagaatga |
| Q5XJ42 | UniRef cluster | --------------------------------------------------MAEFVKAQIKDGKGVVFCKPTCSYCILAKDVLSKYKFKAGHFELID------ISARADMGSIQDYLQQITGARTVPRVFIGEDCVGGGSDVEGLDRSGKLEGMLQAIGCLQ------------------------------- | atggcagagtttgttaaagcgcaaattaaggacggcaaaggagttgtgttttgtaaaccg acctgcagttattgcatactcgcaaaggatgttttgtcgaagtataaatttaaagctgga cattttgagttgatcgatattagcgcacgcgctgacatgggcagcatacaggactatctg caacagatcacgggcgcgcgcactgttcctcgagttttcataggagaagattgtgttgga ggaggaagtgatgttgagggtcttgaccgttctggaaaactggaaggcatgttgcaggcc atcggatgtctgcagtga |
| Q20CD4 | UniRef cluster | ---------------------------------------------------MDTVNRLVEEKALVIFSRSSCCISHSVMQLISSYGANATVYELD----DMSNG----QEVDKALQRLG-LRPSVPAVFIGQKLIGSAKEIISLQVQGKLMPMLKEAGAIWL------------------------------ | atggacactgtgaacagattagtggaagagaaggcactagtaatcttcagccggagttct tgctgcattagccactcggtaatgcaactcataagcagttatggagccaatgcaacagtc tacgagctagatgacatgtcgaatggtcaagaagtagataaagcccttcaaagacttggg cttaggccgagtgttccggctgtttttataggccaaaagttaattggtagtgctaaagag attattagcctacaggtccaaggaaaacttatgccaatgcttaaggaggcaggagctata tggctttag |
| A9PJ56 | UniRef cluster | ---------------------------------------------------MDKVLRLTSEQGVVIFIKSTCCLCYAVKILFQEIGVDPLVHEID----QDPEG----REMEKALTRMG-CSAPVPAVFVGGKLLGSTNEVMSLHLSGSLNQMLKPYQSQT------------------------------- | atggataaggtgttgagattgacctctgagcagggggtagtgatattcatcaagagcaca tgttgcttgtgttatgcagtcaaaatcctgttccaagaaattggggtggaccctctggtt catgagattgaccaagaccctgaaggcagggaaatggaaaaggctctcacaaggatgggg tgtagcgcgcctgtaccggctgtattcgttggtggaaagctgctgggatccaccaatgaa gtcatgtccctccacctcagtggctccctcaatcaaatgctcaaaccctaccagtctcaa acttaa |
| A7NW72 | UniRef cluster | ---------------------------------------------------MDRVGKLASQKAVVIFSKSSCCMSHAIKRLFYEQGVSPAIHELD----EDSRG----KEMEWALMRLG-CNPSVPAVFIGGKFVGSANTVMTLHLNGSLKKMLKEAGAIWL------------------------------ | atggatcgtgttgggaagttggcgtcgcaaaaggcagtggtgatcttcagtaagagttcc tgttgcatgagccatgccatcaagagattgttctatgaacaaggggttagtccggcaatc cacgagctcgacgaggactccagagggaaagaaatggagtgggctctgatgaggctaggg tgcaacccctcagttccggctgtgttcattggggggaaatttgtgggctctgcaaatact gtgatgacccttcatctcaatggctcacttaagaaaatgctgaaagaagccggagctata tggctttag |
| Q9NLB2 | UniRef cluster | -------------------------------------------MAGTSEAVKKWVNKIIEENIIAVFAKTECPYCIKAISILKGYNLNS-HMHVEN------IEKNPDMANIQAYLKELTGKSSVPRIFINKDVVGGCDDLVKENDEGKLKERLQKLGLVN------------------------------- | atggctggtacaagtgaagcagttaaaaaatgggtaaacaaaataatagaagagaacatc attgctgtatttgcaaaaacggaatgcccatattgtattaaggcaatttcaattttgaag ggttacaaccttaacagccatatgcatgtagaaaacattgagaaaaatccagatatggct aatattcaagcatacttaaaagaattaacaggtaaaagtagtgtacctagaatattcatc aataaggacgttgtcggcggatgtgatgatttagttaaagaaaatgatgaaggaaagcta aaagaaagactacaaaaattgggattagtcaattga |
| Q7KXR4 | UniRef cluster | -------------------------------------------MAGTSEAVKKWVNKIIEENIIAVFAKTECPYCIKAISILKGYNLNS-HMHVEN------IEKNPDMANIQAYLKELTGKSSVPRIFINKDVVGGCDDLVKENDEGKLKERLQKLGLVN------------------------------- | atggctggtacaagtgaagcagttaaaaaatgggtaaacaaaataatagaagagaacatc attgctgtatttgcaaaaacggaatgcccatattgtattaaggcaatttcaattttgaag ggttacaaccttaacagccatatgcatgtagaaaacattgagaaaaatccagatatggct aatattcaagcatacttaaaagaattaacaggtaaaagtagtgtacctagaatattcatc aataaggacgttgtcggcggatgtgatgatttagttaaagaaaatgatgaaggaaagcta aaagaaagactacaaaaattgggattagtcaattga |
| A0EIB7 | UniRef cluster | -------------------------------------------------MQVLTPADLKSDKKVILYGATHCPYCSKAKALLANLNIEFEYRGTDVS---------AQFEQEREALGKHLNYETIPMIFVNNQFIGGNSDLHELHEKGGLLPLLK------------------------------------- | atgtaggtattaacaccagcagatctaaaatctgataaaaaagtaattttatatggagca acacactgtccatattgctctaaggcaaaagctcttttagcaaaccttaatatagaattc gagtatcgaggaactgatgtatcagcctaatttgaataagaaagagaagcattaggaaaa catttaaattacgagactattcctatgatttttgtaaacaattaattcataggtggcaac tcagatttgcatgagcttcatgaaaaaggaggtctgttgccattgttaaaatga |
| Q8L8Z8 | UniRef cluster | ---------------------------------------------------MDMITKMVMERPVVIYSKSSCCMSHTIKTLLCDFGANPAVYELD----EISRG----REIEQALLRLG-CSPAVPGVFIGGELVGGANEVMSLHLNGSLIPMLKRAGALWV------------------------------ | atggatatgataacgaagatggtgatggagagaccggtggtgatttacagcaagagctct tgctgtatgtctcacacgatcaagactttgctctgcgatttcggagcaaatccagcggtt tacgagctggatgagatatctagagggagggagatcgagcaggcgttgttgcggctcggg tgtagccccgcagttccgggcgttttcattggtggagagttggtcggtggagccaacgag gtcatgagtctacatcttaacggatccttgattcccatgcttaagcgggctggtgcattg tgggtttga |
| Q5ZF61 | UniRef cluster | ---------------------------------------------------MDKVMKIKSENGVVIFSKSSCCLSYAVQMLFQELRANAFIYEID----HSPDG----KEVEKALMRLG-SSGPIPAVFIGGKLVGSTNEVMSLHLSGSLIPLLKPTQANLSYKS--------------------------- | atggacaaggtgatgaaaatcaagtctgaaaatggagtggtgatcttcagcaagagcagt tgctgcttgagctatgcggttcaaatgctgttccaggaactgagggcgaacgctttcata tacgaaattgaccacagcccagatgggaaggaagtggagaaggcgctgatgaggctaggt agtagtgggcccatcccggcggttttcattggcgggaagctggtgggatcaaccaatgag gtgatgtcactccatttgagtggctcactcatccccctgctcaagccaactcaggctaac ttgtcttacaaatcttga |
| Q4QCU5 | UniRef cluster | -----------------------------------------MNQVLDPARAPQFLDSMLRRNRIVLISATYCQFSTKLKMLLIELKHRFVSLEIDIIP---------NGREVFQEVVARTGVHTVPQVFLNGKYLGGYDDLIALYHKRELSETLEKR----------------------------------- | atgaatcaagtcctagatcctgcacgtgcgccgcagtttctggactcgatgctgcgccgc aaccgcatcgtgctcatctccgccacctactgtcagttctccaccaagctgaagatgttg ctgattgagctgaagcatcgcttcgtctcgctggagattgacatcattcccaacgggcga gaggtttttcaggaggtggtggcccgcaccggcgtgcacacagtgccacaggtgttcctc aacggtaagtaccttggcggatacgacgacctcatcgcgctctaccacaagagagagctc tccgagactctggagaagcggtaa |
| A4HYU2 | UniRef cluster | -----------------------------------------MNQALDPARAPQFLDSMLRRNRIVLISATYCQFSTKLKMLLIELKHRFVSLEIDIIP---------NGREVFQEVVARTGVHTVPQVFLNGKYLGGYDDLIALYHKRELSETLEKR----------------------------------- | atgaatcaagccctagaccctgcacgtgcgccgcagttcctggactcgatgctgcgccgc aaccgcatcgtgctcatctccgccacctactgccagttctccaccaagctgaagatgttg ctgattgagctgaagcaccgctttgtctcgctggagatcgacatcattcccaacgggcga gaggtttttcaggaggtggtagcccgcaccggcgtgcacacagtgccgcaggtgttcctc aacggtaagtaccttggcggatacgacgacctcatcgcgctctaccacaagagagagctc tccgagactctggagaagcggtga |
| A6NLA2 | UniRef cluster | -------------------------------------------------MAQEFVNCKIQPGKLVVFIKPTCPYCRRAQEILSQLSTKQRLLEFVD------ITASNHTNKIQDYLQQLTGARMVPRVFIGKDCIGGCSDLVSMQQIGELLTRLKQIGALQ------------------------------- | atggctcaagagttcgtgaactgcaaaatccagcctgggaagctggttgtgttcatcaag cccacctgtccatactgcaggagggcccaagagatcctcagtcaattgtccaccaaacaa aggcttctggaatttgtcgatatcacagcctccaaccacactaacaagattcaagattac ttgcaacagctcacaggagcgagaatggtgcctcgagtctttatcggtaaagattgtata ggcggatgcagtgatctagtctctatgcaacagattggggaactgctgacgcggctaaag cagattggagctctgcagtaa |
| Q9LYC6 | UniRef cluster | ---------------------------------------------------MERIRDLSSKKAAVIFTKSSCCMCHSIKTLFYELGASPAIHELD----KDPEG----REMERALRALGSSNPAVPAVFVGGRYIGSAKDIISFHVDGSLKQMLKDAKAIWL------------------------------ | atggagagaataagagatttgtcgtcgaagaaagcggcggtgatattcacaaagagctca tgttgtatgtgccatagcatcaagacgctattctacgaactaggcgctagtccggcgatc catgagctcgacaaagaccctgaaggccgtgaaatggaacgggccctacgtgccctcggc tcatcgaacccggcggttccagctgttttcgttggaggaaggtacatcggatcagccaaa gacatcatctcattccacgtggacgggtcactcaagcagatgcttaaagacgctaaggcc atttggttatag |
| Q95WV3 | UniRef cluster | -------------------------------------------MGSTYETIKKFVHKIIDENKIAVFSKTECPYCIKAISILKGYNVN---MHVEQ------IEKNANMADIQSYFKELTGKSSVPRIFINKENVGGCDDWSRETKPETFRETP-DIGMLT------------------------------- | atgggatccacatacgaaactattaaaaaatttgtacataaaatcatcgatgaaaataaa attgctgtattctcaaaaactgaatgcccatattgcattaaagcaatatccattttaaaa ggatataatgttaatatgcatgtagagcaaattgagaaaaatgctaatatggcagatatc caatcatatttcaaggaattaactgggaaaagttccgtaccaagaatatttattaacaaa gaaaatgtcggcggatgtgatgactggtcaagggaaacgaaaccggaaaccttcagagag actccagacattggaatgcttacctag |
| Q4GZ42 | UniRef cluster | -----------------------------------------MNNALDPAKAPQFLDMMLRRNKMVMVSATYCQFCTKLKMLLIELKHRFVSLEIDIIP---------NGREVFAEVVGRTGVHTVPQVFLNGKYFGGYDELVAMYRAGHLSAEIERG----------------------------------- | atgaataacgcattggatccggcaaaggcaccacaatttcttgatatgatgttgcgccgc aataaaatggtaatggtgtccgccacgtattgtcagttctgcacaaaactgaagatgtta ctgattgaactcaaacaccgtttcgtttcacttgaaattgatattattcccaacggtcgt gaagtgtttgcagaagtggtgggacgcacgggtgtacatacggtgcctcaagtttttctt aatgggaagtattttggcggttatgatgaacttgttgcaatgtaccgggcagggcacctt tcagccgaaattgaaaggggctga |
| Q4DGB1 | UniRef cluster | -----------------------------------------MNKALDPAKAPQFLDMMLRRNQIVLISATYCEYCTKLKMLLIEMKHRFVSLEINIIP---------NGREVFAEVVGRTGVHTVPQMFHNGKYLGGYDEIVALYRRGELSATLERR----------------------------------- | atgaacaaagctttagatccagcaaaggcgccgcagtttcttgatatgatgttacgccgc aaccagattgtgcttatctccgctacatattgtgaatattgtacaaagctgaagatgtta ctaattgaaatgaaacaccgctttgtgtcgcttgaaatcaacatcattcccaacgggcga gaggtgtttgcggaagtcgttggacggacaggtgtacacaccgtgccgcagatgtttcac aacggcaaatatcttggtggttatgacgaaattgttgcattgtaccgccgcggggagctc tcagcgacgttggagaggcggtga |
| Q6BZU1 | UniRef cluster | ---------------------------MTHHKNNNKDNHDDELYIEVYQKYLEKAQRLIDEHKVLVVALSWSDACEEIKTCLQTIKGELEPFYLELD-----KEEAMDRVELQKAFVEITSSNAIPSVFVKGKHIGSGEEVMELFHDKKLSEVLAKAGVELPK----------------------------- | atgactcaccacaaaaacaacaacaaggacaaccacgacgacgagctctacattgaggtc taccagaagtacctggagaaggcccagcgcctaattgacgagcacaaggtgctggtagtg gcgctgtcttggtcggatgcgtgtgaggaaattaagacttgcctccagaccatcaagggc gagttggagcccttctacctggagttggacaaggaggaagccatggaccgtgtggagctg cagaaggcttttgtcgagatcacctccagtaacgccatcccatcagtcttcgtcaagggc aagcacattggatctggagaggaggtcatggaacttttccacgacaagaagctctctgag gttctggctaaggctggcgttgagcttcccaaataa |
| Q20CD2 | UniRef cluster | ---------------------------------------------------MDVVNRLVEDKALVIFTRSSCCISHSVLQLISSYGANATVYELD----DMLNG----QEVDKTLQRLG-LRPSVPAVFIGQKLVGGAKEIISLQVQGKLTSMLKEAGAIWV------------------------------ | atggatgtcgtcaacagattagtggaagataaggcgttggtaatcttcacccggagtagt tgctgcataagccactcggtattgcaactcataagcagttatggagccaatgcaacagtc tatgagctagatgacatgttgaatggccaagaagtagataaaactcttcaaagactaggg cttaggccgagtgttcctgctgtttttataggccaaaagctagttggtggtgctaaagag attattagcctacaggtccaaggaaaacttacgtcaatgctaaaggaggcaggagctata tgggtttag |
| Q1EPG3 | UniRef cluster | ---------------------------------------------------MDRVMKLASQKAVVIFSLSSCCMCHTIKRLFCELGVNPAVYELD----EDPRG----REMEKALVKLLGRNPSVPVVFIGGKLVGSTDRIMALHLGGKLTPLLREAGALWL------------------------------ | atggacagggtgatgaagttggcctcgcagaaggcagtggtaatcttcagcttgagctca tgctgcatgtgccacaccatcaagaggctcttctgcgagctgggagtcaatcctgctgtt tatgagcttgacgaagaccccagagggagagagatggagaaggcactggtcaagctgctc ggccgcaacccctcggtgccggtcgtgttcatcggaggaaagctcgttggatccaccgat aggatcatggcccttcatcttggtggcaagctgactccattgcttcgggaggctggtgct ctatggctttga |
| A9SY31 | UniRef cluster | -------------------------------------------------MGVEKVQELILQNPLIIFSKSYCPYCRNVKELLKGLGAEAKVVELD----REMPNVF-VGGEHIGGNDATKAAHKKGTLHPKLKNAGAFKKGKGEATKVEALTTKDTAKVEETKADVVQNMLKGVEDKTKVGLPVKDIVV--- | atgggagtcgagaaggttcaagagttgatactgcagaatcctctcattattttcagcaag agttactgcccctactgccgaaacgtcaaggagctcctgaagggtttaggggctgaagct aaagtggtagaactcgacagagaaatgcctaatgtttttgtcggaggtgaacacattggt ggtaatgatgccactaaagcagcacataagaaaggtaccctccaccctaaattgaagaat gctggcgctttcaaaaaaggcaaaggtgaggcgactaaggtagaagcacttactaccaag gatactgcgaaagtagaggagactaaggccgatgtggtgcagaatatgctgaagggcgtc gaggacaagacgaaggttggacttccagtgaaggatatcgtagtttag |
| O82254 | UniRef cluster | ---------------------------------------------------MERVRDLASEKAAVIFTKSSCCMCHSIKTLFYELGASPAIHELD----KDPQG----PDMERALFRVFGSNPAVPAVFVGGRYVGSAKDVISFHVDGSLKQMLKASNAIWL------------------------------ | atggaacgagtaagagatttggcatcggagaaggcggctgtgatattcacgaagagctcg tgttgcatgtgtcatagcatcaagactctcttctacgaactcggggcgagtcctgccatc catgagcttgacaaggacccgcaaggccctgacatggaacgggccctcttccgggtattc gggtctaaccctgctgtccctgcggttttcgtaggaggaaggtacgtcggctcagctaaa gacgtcatctccttccacgtggatggctccctcaagcagatgttaaaggcctctaacgcc atatggttgtga |
| Q4SD82 | UniRef cluster | ----------------------------------------------------------------------------MAHGVFSEYQFKPGHLECVD------ISGLSDMDKIQDYLRELTGARTVPRVFIGDKCVGGGSDVADLHESGELKDMLQSLGALQ------------------------------- | atggcgcatggggttttctccgagtaccagttcaagccgggacacctggagtgtgtggac atcagcggcctcagcgacatggacaagattcaggactacctccgggagctgacgggggcg cgcacggtgccgcgggtgttcatcggcgacaagtgtgtcgggggaggcagcgacgtggcc gacctgcacgagagcggcgagctgaaggacatgctgcagtccctcggggccctgcagtga |
| A8XNF9 | UniRef cluster | ------------------MLRILTITLAFVAIVSG--ELSKTKEDKTLKDLEDKIVNDIITHKVMVYSKTYCPWSKRLKVILANYEID-DMKIVE----LDRSN---QTEEMQN---------TVPQLFISGKFVGGHDETKAIEEKGELRPMLEKAHALFTNRVPVPDNGA-------------------- | atgctcaggatactcacaatcactctggctttcgtagcgattgtctccggagaactgtcg aaaaccaaagaagataaaacacttaaagatttggaggataaaattgttaatgacatcatc acacacaaagtcatggtatacagtaagacttattgtccatggagcaagcggctgaaggtg atccttgccaattatgagattgatgatatgaaaattgtggaattagatagatcaaatcag acagaggagatgcagaacaccgttcctcaactcttcatcagtggaaagtttgtgggtgga catgacgaaacaaaggctatcgaggagaaaggagagcttcgaccaatgctggagaaggct cacgctttgttcactaaccgagtacctgtacctgacaacggtgcgtga |
| Q28I60 | UniRef cluster | -------------------------------------------------MAQSFVQSKLKPSKVTMFEKPTCPFCVRAKGVLTKYNFKDGHLEIID------ICKLDFMSSLQQYFKQSTGESTVPRIYIGEKCIGGCSDLVPLENSGELEKALQSIGALSD------------------------------ | atggctcagagctttgtgcagagcaaactgaaaccctccaaggtgaccatgtttgagaag cccacgtgtcccttctgcgttagggccaagggggttttaactaaatacaactttaaggat ggacacctggaaatcattgacatctgcaagttggatttcatgtccagcctccaacagtat tttaagcagtcgacaggagagagtacggtaccccgtatatatattggtgaaaaatgtatt ggtggatgttctgacttggtccccctggagaacagcggagagctggaaaaagcgctgcag tctattggtgcattgagtgattaa |
| Q0IMV4 | UniRef cluster | ---------------------------------------------------MDRVMKLASERAVVIFTLSSCCMCHTVTRLFCDLGVNALVHELD----QDPRG----KEMERALLKLLGRGPPVPVVFIGGKLVGGTNKIMSLHLGGELIPMLKNAGALWL------------------------------ | atggaccgtgtgatgaagctagcatccgagcgtgcggtggtgatcttcaccttgagctca tgctgcatgtgccacaccgtgacacgcctcttctgtgatctcggtgtcaacgcgctagtg catgagctggaccaagaccctaggggcaaggagatggagagggcactcctcaagctgctc ggaagggggccgcctgtgccggtggtgttcattggtgggaagctcgttgggggaaccaac aagatcatgtccctccaccttggaggtgagctgatccccatgctcaagaatgcgggagcc ctctggctgtag |
| Q9LYC5 | UniRef cluster | ---------------------------------------------------MDKVMRMSSEKGVVIFTKSSCCLCYAVQILFRDLRVQPTIHEID----NDPDC----REIEKALVRLG-CANAVPAVFVSGKLVGSTNDVMSLHLSGSLVPLIKPYQSFHN------------------------------ | atggacaaggttatgagaatgtcatcggagaaaggagtcgtgatcttcaccaaaagttca tgttgtctctgctacgccgtgcaaatccttttccgtgatcttagggttcaaccaacaatc cacgagatcgacaacgatcctgactgccgtgagatcgagaaggccttagttcgtcttggc tgcgccaacgcggttcctgctgtctttgtaagtggcaagctcgtgggttcgaccaacgat gtcatgtcgcttcacctaagtggctccctcgttcccttgatcaagccgtatcagtcattt cataactag |
| A3E3J6 | UniRef cluster | -----------------------------------MGRQEVSVSQEEVQQAHDTLENLVKTEKCLIFSSTYCPWCDRAAEFFESLNRQCRK-----VELDVPAEGH--SPLLGAVLAQATQQRTVPNTFLFGRHVGGFDRLLSGAERCRKDGDFAAQFPDVCGFLSE------------------------- | atgggcagacaagaggtgtcagtctctcaagaagaggttcagcaagctcatgacacattg gaaaaccttgtcaagacggagaagtgcctgatcttctcctcgacctactgcccgtggtgc gaccgggccgcggagttcttcgagagcttgaaccgccagtgccgcaaggtggagctcgac gtgccggctgagggccactcgccgctcctcggcgccgtgctggctcaggcaacgcagcag cgcacggtgccgaacaccttcttgttcggccggcatgttggtggcttcgaccgtttgctg tcgggggccgagcgatgcaggaaagatggtgacttcgctgcacagttccctgacgtttgt ggctttctgtcggagtag |
| A3CI38 | UniRef cluster | ---------------------------------------------------MDRVMKLASERAVVIFTLSSCCMCHTVTRLFCDLGVNALVHELD----QDPRG----KEMERALLKLLGRGPPVPVVFIGGKLVGGTNKIMSLHLGGELIPMLKNAGALWL------------------------------ | atggaccgtgtgatgaagctagcatccgagcgtgcggtggtgatcttcaccttgagctca tgctgcatgtgccacaccgtgacacgcctcttctgtgatctcggtgtcaacgcgctagtg catgagctggaccaagaccctaggggcaaggagatggagagggcactcctcaagctgctc ggaagggggccgcctgtgccggtggtgttcattggtgggaagctcgttgggggaaccaac aagatcatgtccctccaccttggaggtgagctgatccccatgctcaagaatgcgggagcc ctctggctgtag |
| A2ZLA5 | UniRef cluster | ---------------------------------------------------MDRVMKLASERAVVIFTLSSCCMCHTVTRLFCDLGVNALVHELD----QDPRG----KEMERALLKLLGRGPPVPVVFIGGKLVGGTNKIMSLHLGGELIPMLKNAGALWL------------------------------ | atggaccgtgtgatgaagctagcatccgagcgtgcggtggtgatcttcaccttgagctca tgctgcatgtgccacaccgtgacgcgcctcttctgtgatctcggtgtcaacgcgctagtg catgagctggaccaagaccctaggggcaaggagatggagagggcactcctcaagctgctt ggaagggggccgcctgtgccggtggtgttcattggtgggaagcttgttgggggaaccaac aagatcatgtccctccaccttggaggtgagctgatccccatgctcaagaatgcgggagcc ctctggctgtag |
| Q7G8Y5 | UniRef cluster | ---------------------------------------------------MDRVNRLAAQRAVVIFSMSSCCMCHTVTRLFCELGVNPTVVELD----EDPRG----KEMEKALARLLGRSPAVPAVFIGGRLVGSTDKVMSLHLSGNLVPLLRNAGALWV------------------------------ | atggacagggtgaacaggctggcggcgcagcgggcggtggtgatcttcagcatgagctcg tgctgcatgtgccacaccgtgacgcgcctcttctgcgagctcggggtgaacccgacggtg gtggagctggacgaggacccgagggggaaggagatggagaaggcgctggcgaggctcctc ggccgcagccccgccgtgccggcggtgttcatcggcgggaggctcgtcggctccaccgac aaggtcatgtcgctgcacctcagcggcaaccttgtcccgctgcttcgcaatgcgggtgcc ctctgggtgtag |
| Q6P7J6 | UniRef cluster | -------------------------------------------------MAQNFVQSKVKPSKVTMFEKSSCPFCVRAKGILTKYKFKEGHLEIID------ISKLDFMSSLQQYFMQTTGESTVPRIYIGEKCIGGCSDLVPLENSGELEKALESMGALHV------------------------------ | atggctcagaactttgtgcagagcaaagtgaaaccctccaaggtgaccatgtttgagaag tcctcgtgtcccttctgcgttagggccaaggggattttaacaaaatacaagtttaaggag ggacacctggaaatcattgacatctccaagttggatttcatgtccagcctgcaacagtat tttatgcagacaacgggagagagtacggtaccccgtatatacattggagaaaaatgcatc ggtggatgttctgacttggtccccctggagaacagtggagagctggaaaaagcactggag tctatgggtgcattgcatgtttaa |
| A2ZT66 | UniRef cluster | ---------------------------------------------------MDRVNRLAAQRAVVIFSMSSCCMCHTVTRLFCELGVNPTVVELD----EDPRG----KEMEKALARLLGRSPAVPAVFIGGRLVGSTDKVMSLHLSGNLVPLLRNAGALWV------------------------------ | atggacagggtgaacaggctggcggcgcagcgggcggtggtgatcttcagcatgagctcg tgctgcatgtgccacaccgtgacgcgcctcttctgcgagctcggggtgaacccgacggtg gtggagctggacgaggacccgagggggaaggagatggagaaggcgctggcgaggctcctc ggccgcagccccgccgtgccggcggtgttcatcggcgggaggctcgtcggctccaccgac aaggtcatgtcgctgcacctcagcggcaaccttgtcccgctgcttcgcaatgcgggtgcc ctctgggtgtag |
| A2WQ16 | UniRef cluster | ---------------------------------------------------MDRVNRLAAQRAVVIFSMSSCCMCHTVTRLFCELGVNPTVVELD----EDPRG----KEMEKALARLLGRSPAVPAVFIGGRLVGSTDKVMSLHLSGNLVPLLRNAGALWV------------------------------ | atggacagggtgaacaggctggcggcgcagcgggcggtggtgatcttcagcatgagctcg tgctgcatgtgccacaccgtgacgcgcctcttctgcgagctcggggtgaacccgacggtg gtggagctggacgaggacccgagggggaaggagatggagaaggcgctggcgaggctcctc ggccgcagccccgccgtgccggcggtgttcatcggcgggaggctcgtcggctccaccgac aaggtcatgtcgctgcacctcagcggcaaccttgtcccgctgcttcgcaatgcgggtgcc ctctgggtgtag |
| Q9LIF1 | UniRef cluster | ---------------------------------------------------MDVVARLASQRAVVIFSKSTCCMSHAIKRLFYEQGVSPAIVEID----QDMYG----KDIEWALARLG-CSPTVPAVFVGGKFVGTANTVMTLHLNGSLKILLKEAGALWL------------------------------ | atggatgtggtagcaagattagcgtcgcaaagagcggtggtgatattcagcaagagtacg tgttgcatgtctcatgcaattaaacggttgttttacgagcaaggtgtgagcccggcaatt gtagagatcgaccaagacatgtatgggaaagatatcgagtgggccttggcccgattaggc tgtagccctacggttcctgcggtttttgttggagggaaattcgtaggaacggccaatact gtcatgactcttcatctcaatggatcattgaaaatattgctcaaggaggctggtgctttg tggctttga |
| A5DVV3 | UniRef cluster | -------------------MLKSVFRSTTFSPTSLYLRRYISQSNMVSQQVKDKVEKLIKEKPIFIASKTYCPYCQKTKQTIGSITKD--------AYIIELDESE-DGAEIQEALLEITGQRTVPNVFIGGQHVGGNSDVQALKS----EDKLEDKIKAAL------------------------------ | atgcttaaaagtgtttttcgttcaacaactttttcaccaacatcattgtatctgagacgt tatatatcacaatcaaacatggtttcacaacaagtcaaagacaaagtcgagaaattgatc aaggagaagccaatttttattgcttcaaagacttattgcccatactgtcaaaaaactaag cagacaattggatctatcaccaaggatgcatacattatagaattggatgagtcggaagat ggtgcagagattcaagaagctttgctcgaaattactggccaaagaactgtgccaaatgtc tttattggtggccaacatgttggtggtaactctgatgtgcaagcattgaagtctgaagac aagttggaggacaagatcaaggctgccttataa |
| A7PCX4 | UniRef cluster | ----------------------------------MGSVLGKGKSKEEVEMALAKAKEIVSSTPVVVFSKTYCGYCKRVKQLLSQLKATHKTIELD----QESD-----GAEIQSALREWTGQSTVPNVFIGGKHMGGCDSVMEKHQEGKLVPLLKEAGAIAEVSTQL------------------------- | atgggttccgttctcggcaagggaaagagcaaggaggaagtggaaatggccctcgcaaag gccaaagagatcgtttcttcaacacctgtagttgtcttcagtaagacttactgtggttat tgcaagagagtgaagcagctgctctcacagctaaaagcaacccacaagaccattgaactc gatcaggaaagtgatggagctgaaattcaatcagctctaagagaatggactggtcagagc actgtgcctaacgtatttattgggggaaaacacatgggtggatgtgactctgtgatggaa aagcaccaggaaggcaagcttgtgccccttctcaaagaggctggtgccattgctgaagtt tctacccagctctga |
| A9NY07 | UniRef cluster | ------------------------------------MGGSTSKNPQIIEMAMGKAKEIVSSNSVVVFSKTYCPYCTQVKQLLSSLGAKTKVVELD----TESD-----GKEIQTALQEWTGQRTVPNVFIGGTHIGGCDDTVAKHNSGKLVPLLTEAGGV-------------------------------- | atgggcggatcaaccagcaagaatccacagataatagaaatggcgatgggtaaagcgaag gagatcgtttcctctaattctgtcgtggttttcagcaagacatactgcccctattgtaca caggtcaaacagctgttaagtagcttgggtgccaagactaaggtggtagagctggatact gaaagtgatggaaaggagatacagacagctcttcaggaatggacaggacagcggactgtc ccaaatgtatttattggaggcacacacattggcggctgtgatgacaccgttgcgaaacat aacagtgggaagcttgtaccattgttgacagaagctggcggtgtttga |
| Q6BIM4 | UniRef cluster | ---------------------------------------------MVSQHTTDKVQQLIKTKPVFIASKSYCPYCAKTKNTISSITKD--------AYIIELDEVE-DGSEIQEALYELTGQKTVPNVFIGGEHIGGNSDVQELSS----GDKLESKIKAVL------------------------------ | atggtctctcaacatactaccgacaaggttcaacaattgattaaaactaagccagttttc atcgcttcaaagtcctactgtccatactgtgctaagactaagaataccatttcgtccatc actaaggatgcttacatcattgaattagatgaagtggaagatggtagtgaaattcaagaa gctttatacgaattaactggacaaaagaccgttccaaatgttttcattggaggtgaacac attggtggtaactctgatgttcaagaattgagttccggtgataaattggaaagcaagatc aaggctgtcttataa |
| Q945T3 | UniRef cluster | -------------------------------------------------MALPKAKEIVSGNPVVVFSKSYCPFCVSVKQLLEQIGASFKAIELD----NESD-----GSEIQAALAEWTGQRTVPNVFIGGKHIGGCDSTTAMHKNGKLIPLLTEAGAVATATATVTATASA------------------- | atggctttgccgaaggctaaggagatcgtttccgggaatccggtcgtcgttttcagcaag tcgtactgtccgttctgtgtgagcgtgaagcagttgcttgaacaaattggagcctctttc aaggccattgagcttgacaatgaaagtgatggaagtgaaattcaagcagctctggccgag tggactggtcagaggactgtgccaaatgttttcattggcgggaaacacattggtggctgt gactctacaacagctatgcacaagaatgggaagctgattcctctgctaaccgaagctgga gctgttgctactgctactgctactgttactgctactgcttccgcttaa |
| A9NK43 | UniRef cluster | ------------------------------------MGGSTSKNPQIIEMAMGKAKEIVSSNSVVVFSKTYCPYCTQVKQLLSSLGAKTKVVELD----TESD-----GKEIQTALQEWTGQRTVPSVFIGGTHIGGCDDTVAKHNSGKLVPLLTEAGGV-------------------------------- | atgggcggatcaaccagcaagaatccacagataatagaaatggcgatgggtaaagcgaag gagatcgtttcctctaattctgtcgtggttttcagcaagacatactgcccctattgtaca caggtgaaacagctgttaagtagcttgggtgccaagacgaaggtggtagagctggatact gaaagtgatggaaaggagatacagacagctcttcaggaatggacaggacagcggactgtc ccaagtgtatttattggaggcacacacattggcggctgtgatgacaccgttgcgaaacat aacagtgggaagcttgtaccattgttgacagaagctggcggtgtttga |
| Q5ABB1 | UniRef cluster | ------------------------------MFRTLLTKRLFNTSTMVSSQVKNKVEQLIKTKPVFIASKSYCPYCKATKSTIEAITKD--------AYILELDEVD-DGAEIQEALLEITGQRTVPNVFIGGQHIGGNSDVQALKS----SDKLDDKIKAAL------------------------------ | atgtttcgtacattattaaccaaaagactattcaatacatcaacaatggtttcatctcaa gttaagaacaaggtcgaacaattgatcaaaaccaaaccagttttcattgcctccaaatcc tattgtccatactgtaaggctaccaaaagcacaattgaagctataacaaaggatgcttac attcttgaattagacgaagttgacgacggtgctgaaatccaagaagcattattggaaatc actggtcaaagaaccgttccaaatgtctttattggtggtcaacatattggtggcaattcc gatgtgcaagctttgaagtctagtgacaaattagatgacaaaatcaaagctgctttataa |
| A9QXE9 | UniRef cluster | -------------------------------------------------MAMSKAKELVSSNGVVVFSKTYCSYCQTVKKLLTDIGASFKVIEMD----KESD-----GSEIQSALVEWTGQRTVPNVFIGGKHIGGCDLTTAMHKSGKLVPLLTEAGAVASKS---------------------------- | atggcgatgtctaaggcaaaggagctggtttcatccaatggagtcgtcgtcttcagcaaa acctattgttcatactgccagactgtgaagaaattgttgaccgacatcggtgcctctttc aaagtcatcgagatggacaaagaaagtgatgggagtgaaattcaatcagcactagtagag tggaccggacagcgtactgtgccgaatgtgttcattggcggaaagcacattggtggctgt gatttaacaacggccatgcacaagagtgggaaacttgttcctttgctgactgaagctgga gctgttgctagcaaatcttaa |
| Q6CCY8 | UniRef cluster | ---------------------------------------------MATESAIKTIKEHIAKDKVFVASKSYCPYCKQTKQLLSQFKEAKP-------VILELDELD-DGAELQAALAEITGQRTVPNVFIGGQHIGGNSDLQVLAQ----KDELADKIKAAL------------------------------ | atggccaccgagtccgctatcaagaccatcaaggagcacattgccaaggacaaggtcttc gtggcctccaagtcctactgcccctactgcaagcagaccaagcagctgctttcccagttc aaggaggccaagcccgtgattctcgagctcgacgagcttgacgatggtgccgagctccag gctgctctcgccgagatcactggccagcgaaccgttcccaacgttttcattggaggccag cacattggtggcaactccgatctgcaggttcttgcccagaaggacgagctggccgataag atcaaggctgccctttaa |
| Q9FNE2 | UniRef cluster | -------------------------------------------------MAMQKAKEIVNSESVVVFSKTYCPYCVRVKELLQQLGAKFKAVELD----TESD-----GSQIQSGLAEWTGQRTVPNVFIGGNHIGGCDATSNLHKDGKLVPLLTEAGAIAGKTATTSA----------------------- | atggcgatgcagaaagctaaggagatcgttaacagcgaatcagtcgttgttttcagcaag acttattgtccatattgcgtgagagtgaaggagcttttgcaacaattgggagctaagttc aaggccgttgagctcgacaccgaaagtgatggtagccaaattcaatcaggtctcgcagaa tggacaggacaacgtaccgtgcctaatgtgtttataggaggaaatcacatcggtggctgt gatgcaacatcaaacttgcataaagatgggaagttggttccgctgttaactgaagctgga gcgatcgcaggaaagactgcaacaacttctgcttaa |
| O50042 | UniRef cluster | -------------------------------------------------MALAKAKDLVASNPVVVFSKSYCPYCIRVKELLVKLKATYKVIELD----LESD-----GSAIQAALAEWTGQRTVPNVFIGGKHIGGCDKTMELYNSGKLQPLLVEAGALAA------------------------------ | atggctctcgcgaaggcaaaggatctcgtcgcctccaaccccgtcgtcgtcttcagcaag tcatactgtccctactgtatccgggttaaggagttgctggtgaagttgaaggcaacttac aaagtgattgaattggatctggaaagtgatggatcggcaatacaagctgcactagctgag tggaccggacaaaggacggtgccaaatgttttcatcggtgggaagcatatcggtggctgt gataagactatggagctgtacaactctggcaagctgcagccgttgttggtggaggctggg gctcttgctgcttag |
| O81187 | UniRef cluster | -------------------------------------------------MAMIKAQELVSSNSVVVFSKTFCPYCTSVKQLLNQLGAQFKVIELD----SESD-----GSDLQNALAEWTGQRTVPNVFIGGKHIGGCDKTTGMHQEGKLIPLLTEAGAVKA------------------------------ | atggcgatgataaaggcccaggagcttgtttcttccaactccgtcgttgttttcagcaag acgttttgtccttattgcacgagcgtcaagcagctcttgaatcaattaggagcacaattc aaggtcatcgagttggattccgagagtgatggaagtgatttacaaaatgcattggctgag tggactggacaacgcactgtgcccaatgttttcatcggcggcaagcacattggtggctgt gataaaactacaggcatgcaccaggaagggaagttgattcctctgctcactgaagccgga gctgtcaaggcttaa |
| Q94JS9 | UniRef cluster | -------------------------------------------------MALAKAKEIAASSPVVVFSKSYCPYCTRVKQLFTKLGASFKAIELD----VEGD-----GADMQSALAQWTGQKTVPNVFINGKHIGGCDDTLALEKSGKLVPLLREAGAISGSASKETMTA--------------------- | atggcgctcgccaaggccaaggaaatcgccgcctcttctcccgtcgtcgtcttcagcaag tcttactgcccttactgcacacgggtaaagcaattgttcacaaagctaggagcaagtttc aaggctattgagctagatgtggaaggtgatggagctgatatgcagtcagcccttgctcaa tggactggacagaagaccgttccaaatgtcttcatcaatgggaaacacatcggtggttgt gatgatacccttgcactggagaagtcagggaagctggtgcctctgctgagggaggccgga gcaatctctggttctgcttcgaaggaaaccatgactgcttag |
| A9T8R5 | UniRef cluster | -------------------MASRTGAVWVSFAVAGMILMQLGSPVEARTDSLAFVKKTLAEHPLVIFSKSYCPYCKRAKSVFESMSVK--PFVLE----LDERE---DGDDIQQALGKFVGRRTVPQVFINGVHLGGSDDTVAAQQSGRLKKLLAGSASAVNAENLKSEL---------------------- | atggcttcacggactggtgccgtatgggtttctttcgcagtggcgggtatgattctgatg caactggggtcacccgtggaagccagaactgattccttggctttcgtgaagaagaccttg gctgagcatcctttggtcatcttttctaagtcttattgcccatactgtaagcgtgcgaag tcggtgtttgagagcatgagcgtgaagccgtttgtgcttgagcttgacgagcgagaggat ggagatgacattcagcaggcactagggaaattcgttggtcgtagaacggtaccccaagta ttcatcaatggggtgcaccttggaggatctgatgacactgttgctgctcagcaaagtggc cgcctaaagaagctgctggctggttctgcctctgccgttaatgcagaaaatctgaagtct gaactatga |
| A3GFD7 | UniRef cluster | ---------------------------------------------MVSQATTEKVKTLIKTKPVFIASKSYCPYCKATKATIGAITQE--------AYVIELDEIA-DGAEIQEALFELTGQKTVPNVFIGGEHIGGNSDVQVLKS----QDKLDDKIKAVL------------------------------ | atggtttcccaagctacaaccgaaaaggtcaagaccttgatcaagaccaagccagtcttc attgcttccaagagttactgtccttactgtaaggccaccaaagctaccattggtgctatt acccaagaagcctacgtcattgaattggacgaaattgctgatggcgcagaaatccaagaa gctttgttcgaattgactggccaaaagaccgttccaaacgtcttcattggtggagaacac attggtggtaactccgatgtccaagtgttgaagtctcaagacaagttggacgataaaatc aaggctgtcttgtaa |
| P55143 | UniRef cluster | -------------------------------------------------MAMTKTKELVSSNAVVVFSKTYCPYCTSVKKLLDQLGAKYKVVELD----TESD-----GSEIQTALAEWTGQRTVPNVFIGGKHIGGCDSTTAKHSQGQLVPLLTEAGAV-------------------------------- | atggcgatgactaagaccaaagagcttgtttgctcaaacgctgttgtcgttttcagcaag acgtactgcccctattgcacgagcgtgaagaagctattggatcagttgggagctaaatat aaggtcgtggagttggataccgagagtgatggaagtgagattcaaacagctctggcagag tggaccggacaacgcaccgtgcccaatgttttcattggcggaaagcacatcggtggctgt gacagtacaacagccaagcacagccaagggcagctggttcctctgctcacagaagctgga gctgtatag |
| Q8S3L1 | UniRef cluster | -------------------MATRIRLPSILATAVTLTVLAASLTWAAGSPEATFVKKTISSHQIVIFSKSYCPYCKKAKGVFKELNQT--PHVVE----LDQRE---DGHDIQDAMSEIVGRRTVPQVFIDGKHIGGSDDTVEAYESGELAKLLGVASEQKDDFKLE------------------------- | atggcgacgaggataagattgccatcaatcttggctacagcagtaacattaacagtactt gcggcatcactcacctgggctgctggcagccctgaagctacttttgtcaaaaagaccatc tcttctcatcagatcgtcatcttctccaagtcttattgcccgtattgtaagaaggctaaa ggtgttttcaaagaactgaaccagacaccacatgttgtcgagctcgatcaaagagaggat gggcacgacattcaggatgccatgagtgaaattgttgggaggcgcaccgtgcctcaggtt ttcatagacgggaagcacattggtggctcagatgacaccgtggaagcatacgaaagtgga gaacttgctaagcttttaggagttgcttcagagcagaaagatgactttaaactagaatga |
| A9PBC3 | UniRef cluster | -------------------MATRIRLPSILATAVTLTVLAASLTWAAGSPEATFVKKTISSHQIVIFSKSYCPYCKKAKGVFKELNQT--PHVVE----LDQRE---DGHDIQDAMSEIVGRRTVPQVFIDGKHIGGSDDTVEAYESGELAKLLGVASEQKDDL---------------------------- | atggcgacgaggataagattgccatcaatcttggctacagcagtaacattaacagtactt gcagcatcactcacttgggctgcaggcagccctgaagctacttttgtcaaaaagaccatc tcttctcatcagatcgtcatcttctccaagtcttattgcccgtattgtaagaaggctaaa ggtgttttcaaagaactgaaccagacaccacatgttgtcgagctcgatcaaagagaggat gggcacgacattcaggatgccatgagtgaaattgttgggaggcgcaccgtgcctcaggtt ttcatagacgggaagcacattggtggctcagatgacaccgtggaagcatacgaaagtgga gaacttgctaagcttttaggagttgcttcagagcagaaagatgatctctaa |
| Q5B5G5 | UniRef cluster | -----------------------------------------------MSSAKVKAQQIIDENGVVVFSKSYCPYCKASKSLLSELGAKYYA--------LELDTID-DGADLQNALEEISGQRTVPNIYIAKKHIGGNSDLQGI------KKDLPALLKDAGAL---------------------------- | atgtcttccgcaaaggtcaaggcccagcaaatcattgatgagaacggcgttgtcgtcttc tccaagtcctactgcccctactgcaaggccagcaagagcctcctgagcgaactgggcgcc aaatactacgcgcttgagctggataccatcgatgacggagctgacctccagaacgccctt gaggaaatcagcggccagcgcaccgttcccaacatctacattgccaaaaagcacattggt ggaaactcggacctccagggtatcaagaaggatctgcccgcgctgttaaaggatgctgga gctctgtaa |
| Q2GX69 | UniRef cluster | -----------------------------------------------MEAAQKKAQALIDENAVMIFSKSYCPYCRDAKSVFSTRDVKYKA--------VELNQMD-DGDDIQDALQKMTGQRTVPNIFIGGTHIGGSSDLNNVVSSGKDGKSIDVLLKEAGAL---------------------------- | atggaagccgcacagaagaaagcccaggccttgatcgacgaaaatgccgtcatgatcttt agcaagtcgtactgcccttactgccgcgacgccaagagtgtcttcagcacccgggacgtc aaatacaaggcggtcgagctcaaccagatggacgacggcgacgatatccaggacgctttg cagaagatgaccggccagcgcaccgtccccaacatcttcatcggtggcactcacattgga gggagttcggatctcaacaacgtcgtctctagcggcaaggatggaaagtcgatcgacgtg ttgttgaaggaggctggtgctctgtga |
| Q7YUB8 | UniRef cluster | -----------------------------------------------MGKVNEYVDSVIAKNKVAVFSKTYCPYCDKAKQALNSFNIKPGALEVVE------LDKRDDGNEIQDYLAQLTGGRTVPRVFINGQFIGGGDDTARAKSNGSLEKKLTEIGAI-------------------------------- | atgggaaaagtcaacgaatacgtggacagcgtcattgcgaagaacaaggtcgcggtgttt tcgaagacctactgtccgtactgcgacaaggcgaagcaggcgctgaactcgttcaacata aagccgggcgcgctggaggtcgtcgagctggacaagcgtgacgacggcaacgagatccag gactatctcgctcagctgactggtggacggaccgttccgcgagtgttcatcaacggacag ttcatcggcggaggcgacgacaccgcgcgcgccaagtccaacggcagtctggagaagaag ctcaccgaaatcggtgctatctaa |
| P17695 | UniRef cluster | -----------METNFSFDSNLIVIIIITLFATRIIAKRFLSTPKMVSQETVAHVKDLIGQKEVFVAAKTYCPYCKATLSTLFQELNVPKS----KALVLELDEMS-NGSEIQDALEEISGQKTVPNVYINGKHIGGNSDLETLKK----NGKLAEILKPVFQ----------------------------- | atggtatcccaggaaacagttgctcacgtaaaggatctgattggccaaaaggaagtgttt gttgcagcaaagacatactgcccttactgtaaagctactttgtctaccctcttccaagaa ttgaacgttcccaaatccaaggcccttgtgttggaattagatgaaatgagcaatggctca gagattcaagacgctttagaagaaatctcgggccaaaaaactgtacctaacgtatacatc aatggcaagcacattggtggtaacagcgatttggaaactttgaagaaaaatggcaagtta gctgaaatattgaagccggtatttcaatag |
| Q7XY25 | UniRef cluster | -------------------------------------------------MALAKAKEIVASASVVVFSKSYCPFCVQVKKLLTQLGASFKAVEMD----TESD-----GTEIQSALAEWTGQRTVPNVFINGKHIGGCDDTIALNKGGKLVALLTEAGAISGSSSKTTVTA--------------------- | atggcgctcgccaaggccaaggagatcgtcgcctccgcttccgtcgtcgtcttcagcaag tcttactgccctttctgtgttcaagtgaagaagttgctcacgcagcttggagcaagtttc aaggccgttgagatggacactgaaagcgatggaactgagattcagtcagctcttgctgaa tggactgggcagaggactgttcccaatgtcttcatcaatggaaaacacatcggtggctgt gacgatactattgcactgaacaagggagggaagctggttgctctgctgacagaggctgga gcgatctccggttcttcttcgaagaccaccgtcactgcttag |
| A6ZZA0 | UniRef cluster | -----------METNFSFDSNLIVIIIITLFATRIIAKRFLSTPKMVSQETVAHVKDLIGQKEVFVAAKTYCPYCKATLSTLFQELNVPKS----KALVLELDEMS-NGSEIQDALEEISGQKTVPNVYINGKHIGGNSDLETLKK----NGKLAEILKPVFQ----------------------------- | atggagaccaatttttccttcgactcgaatttaattgttattatcattatcacgttgttt gccacaagaattattgctaaaagatttttatctactccaaaaatggtatcccaggaaaca gttgctcacgtaaaggatctgattggccaaaaggaagtgtttgttgcagcaaagacatac tgcccttactgtaaagctactttatctaccctctttcaagaattgaacgttcccaaatct aaggcccttgtgttggaattagatgaaatgagcaatggctcagagattcaagacgcttta gaagaaatctcgggccaaaaaactgtacctaacgtatacatcaatggcaagcacattggt ggtaacagcgatttggaaactttgaagaaaaatggcaagttagctgaaatattgaagccg gtatttcaatag |
| Q8L8T2 | UniRef cluster | ---------------------------------MGSMFSGNRMSKEEMEVVVNKAKEIVSAYPVVVFSKTYCGYCQRVKQLLTQLGATFKVLELD----EMSD-----GGEIQSALSEWTGQTTVPNVFIKGNHIGGCDRVMETNKQGKLVPLLTEAGAIADNSSQL------------------------- | atggaggtggtggtgaacaaggctaaagagatcgtctctgcttatcccgttgttgtcttc agcaagacatactgtggttattgccagagggtgaagcagttactgacgcagctaggagca acttttaaagtacttgagctcgatgaaatgagtgatggaggtgagatccaatcagcttta tcagagtggactggacagaccacagttccaaacgtcttcatcaaaggaaaccacatcggt ggatgcgatagagtgatggagaccaacaagcaaggcaagcttgtgcctctacttactgaa gctggggctattgcagataactcttctcaactttga |
| A4RP84 | UniRef cluster | ----------------------------------------------MSEAAKQKAQKLIDENAVMVFSKSYCPYCTATKSKLKDIGAKYNV--------LELDQID-DGSAIQDALQEITGQRSVPNIFIGQKHIGGNSDFQALG-----NS--ESLIKAAGAL---------------------------- | atgtctgaagcagcaaagcaaaaggctcaaaagctcattgacgagaacgccgttatggtg ttcagcaagtcatactgcccctactgcaccgccaccaagtcgaagctcaaggatataggc gcaaagtacaacgtcctcgagctggaccagattgatgacggcagcgccatccaggatgca cttcaggagatcaccggccagcgctcggttcccaacattttcatcggccagaagcacatt ggcggcaactcggacttccaggcactcggcaactcggaaagcttgatcaaggcggcaggt gctttgtga |
| Q0DAE4 | UniRef cluster | -------------------MAALLGRRFGMAAAALIALAALGSAASGTASKSSFVKSTVKAHDVVIFSKSYCPYCRRAKAVFKELELKKEPYVVE----LDQRE---DGWEIQDALSDMVGRRTVPQVFVHGKHLGGSDDTVEAYESGKLAKLLNIDVKEDL------------------------------ | atggcggcgctgctgggccggaggttcgggatggcggcggcggcgctcatcgccctcgcg gcgctcggatccgccgcctcggggacggcgtccaagtcgtccttcgtgaaatccaccgtc aaagcccacgacgtcgtcatattctccaagtcatactgcccgtactgtagaagagccaaa gctgtgttcaaggaacttgaactgaagaaggagccgtatgttgtggagcttgatcaacga gaggatggttgggagattcaggatgccttatctgacatggttggcaggcgaactgttcct caagtttttgtccatgggaagcacctgggtggctctgatgatactgttgaagcatatgag agtggcaagctagccaaacttttgaacattgatgtcaaagaagatctttga |
| A3BE95 | UniRef cluster | -------------------MAALLGRRFGMAAAALIALAALGSAASGTASKSSFVKSTVKAHDVVIFSKSYCPYCRRAKAVFKELELKKEPYVVE----LDQRE---DGWEIQDALSDMVGRRTVPQVFVHGKHLGGSDDTVEAYESGKLAKLLNIDVKEDL------------------------------ | atggcggcgctgctgggccggaggttcgggatggcggcggcggcgctcatcgccctcgcg gcgctcggatccgccgcctcggggacggcgtccaagtcgtccttcgtgaaatccaccgtc aaagcccacgacgtcgtcatattctccaagtcatactgcccgtactgtagaagagccaaa gctgtgttcaaggaacttgaactgaagaaggagccgtatgttgtggagcttgatcaacga gaggatggttgggagattcaggatgccttatctgacatggttggcaggcgaactgttcct caagtttttgtccatgggaagcacctgggtggctctgatgatactgttgaagcatatgag agtggcaagctagccaaacttttgaacattgatgtcaaagaagatctttga |
| A2YFU4 | UniRef cluster | -------------------MAALLGRRFGMAAAALIALAALGSAASGTASKSSFVKSTVKAHDVVIFSKSYCPYCRRAKAVFKELELKKEPYVVE----LDQRE---DGWEIQDALSDMVGRRTVPQVFVHGKHLGGSDDTVEAYESGKLAKLLNIDVKEDL------------------------------ | atggcggcgctgctgggccggaggttcgggatggcggcggcggcgctcatcgccctcgcg gcgctcggatccgccgcctcggggacggcgtccaagtcgtccttcgtgaaatccaccgtc aaagcccacgacgtcgtcatattctccaagtcatactgcccgtactgtagaagagccaaa gctgtgttcaaggaacttgaactgaagaaggagccgtatgttgtggagcttgatcaacga gaggatggttgggagattcaggatgccttatctgacatggttggcaggcgaactgttcct caagtttttgtccatgggaagcacctgggtggctctgatgatactgttgaagcatatgag agtggcaagctagccaaacttttgaacattgatgtcaaagaagatctttga |
| Q8LFQ6 | UniRef cluster | --------------------MTMFRSISMVMLLVALVTFISMVSSAASSPEADFVKKTISSHKIVIFSKSYCPYCKKAKSVFRELDQV--PYVVE----LDERE---DGWSIQTALGEIVGRRTVPQVFINGKHLGGSDDTVDAYESGELAKLLGVSGNKEAEL---------------------------- | atgacaatgtttagatctatctccatggtaatgctgctcgtcgcactagttacattcatt tctatggtttcttctgctgcttcgtccccagaagccgactttgttaagaagactatctct tcccataagatcgtcattttctccaaatcctactgcccctactgcaagaaagctaaatca gtgttcagagagctggatcaagttccttatgttgtcgagcttgatgaaagagaagatggt tggagcatccagactgcacttggagagattgttggaaggcgaacagtaccgcaagtcttc attaacggaaaacatctcggaggatcagatgataccgtagatgcgtatgagagcggtgaa ctcgccaagcttcttggtgtttccgggaacaaagaagctgaactctag |
| Q9P718 | UniRef cluster | ----------------------------------------------MSDAATQKAKQLINDNAVVVFSKSYCPYCSNTKQILDGLNAKYAT--------YELNQES-DGSDVQDALLKLTGQRTVPNIFIGKQHIGGNSDLEAVVKNGKNGKKIQELLQEAGAL---------------------------- | atgtccgacgccgctactcagaaggctaagcagctgatcaacgacaacgctgtcgttgtt ttcagcaagtcctactgcccctactgctccaacaccaagcaaatcctcgacggcctcaat gccaagtatgctacctacgagctcaaccaagaaagcgacggctccgatgtccaggacgct cttctcaagttgaccggccagcgcactgtcccaaacatcttcattggcaagcagcacatt ggcggtaactccgatctcgaggctgttgtcaagaacggcaagaacggcaagaagatccaa gagctcctccaagaggcgggcgctctgtaa |
| A5E7C5 | UniRef cluster | ---------------------------------MFEWITSWFQPTPVSPEIKQLIETTTQTNNIVVYSKTYCPYCTATKNLLSQYGVP--------YELIELNSVN-NGAEIQRALQEVTGQRTVPNIFINGKHIGGNSDLQALEQ----SNKLKQLLASSLN----------------------------- | atgtttgaatggattacttcttggtttcaaccaacaccagtttctccagagatcaaacag ttgatcgaaacaactacacagactaataatattgttgtatattccaaaacctattgtcca tactgcacagcaaccaagaatttgctttctcaatatggcgttccttacgagctcattgag ttgaattccgtcaacaatggtgcagagatacaaagagcattgcaagaggttacagggcaa agaacggttccaaacattttcatcaatgggaaacatattggaggaaactcggatttacaa gccttggaacagagtaacaagttgaagcaattacttgcttcctcacttaattag |
| Q0U8Y1 | UniRef cluster | ------MVPEARSGRNEVARLGTPTQKKTATISFLRHFFTRAPSPATMSATKTKVQSIIDENPVAVFSKSYCPYCRQAKELLSQSGAKFYA--------IELDQVD-DGSAIQSTLGEMTGQTTVPNIFIAKEHIGGNSDLQAK------KNNLKTLLKDAGAL---------------------------- | atggtccctgaagcgcgatccggacgaaacgaagtcgcgaggctcggaacgcccacccaa aaaaaaaccgccaccatttccttcctgcgccacttcttcacgcgcgccccctcgcccgcc accatgtccgccacaaagaccaaggtccaatccatcatcgacgagaaccccgtcgccgtc ttcagcaagagctactgcccttactgccgccaggcaaaggagctgctgagccagagcggc gccaagttctatgctattgagctggaccaagtcgatgacggctcagccattcagagcacc ctcggcgagatgacgggccagactaccgtgcccaacatcttcattgcgaaggagcacatt ggtggaaactcggacctgcaggccaagaagaacaacttgaagacgctgctcaaagatgct ggtgctttgtag |
| A5DE43 | UniRef cluster | ---------------------------------MLGYLLSWFKSEPSSPEVISKVQSLINANKIVVFSKSYCPYCSQTKQLLNQVGASD-------VNIIELNNVS-DGAAMQNALQEITGQRTVPNTFINQKHIGGNSELQQLHR----ANKLVPML---------------------------------- | atgttagggtacttgctttcctggttcaaaagtgaaccatcatctcctgaagtcatcagc aaggtccaaagtttgatcaatgcaaacaaaattgtggttttttcaaaatcttactgtcca tattgctcacaaaccaagcaattgttgaaccaagttggagccagtgacgtgaacattatt gagttgaacaatgtttctgatggagctgctatgcaaaatgcattgcaagagattaccgga caaagaactgtacccaacacttttataaaccaaaaacatattggaggaaacagtgaattg cagcagttgcaccgtgcaaacaaacttgtgccaatgttgtag |
| P55142 | UniRef cluster | -------------------------------------------------MALAKAKETVASAPVVVYSKSYCPFCVRVKKLFEQLGATFKAIELD----GESD-----GSELQSALAEWTGQRTVPNVFINGKHIGGCDDTLALNNEGKLVPLLTEAGAIASSAKTTITA---------------------- | atggcgctcgccaaggccaaggagaccgtcgcttccgctcccgtcgtcgtctacagcaag tcttactgtcctttttgcgtccgtgtgaagaagttgttcgggcagcttggagcaactttc aaggccattgagttggatggggagagtgatggatctgagctgcagtcggcacttgctgaa tggactggacaaaggactgttccaaatgtcttcatcaatgggaagcatattggtggctgt gatgatactttggcattgaacaatgaagggaagctggtgcctctgctgaccgaggctgga gcaattgccagttctgcaaagacgacaatcaccgcatag |
| Q28IG0 | UniRef cluster | -------------------------------------MGISSTKEVSETEATDIIKNTIAENCVVIFSKTTCPYCVMAKEAFKNIDV--QYTAVE----LDELE---NGRQMQVALQQLSGIRTVPQVYVNGKCIGGGTDTRNLEREGKLLKLVQECNLSAAT----------------------------- | atgggaatcagttctacaaaagaggtatctgagacagaagccactgatataataaagaac acaattgcagaaaactgtgtagtgatattctcaaaaaccacctgtccttactgtgtaatg gcaaaagaggccttcaaaaatatagatgtgcagtacacggcagttgaactggatgaacta gagaatggaagacagatgcaagtggcacttcagcaactaagtggaattaggactgtcccc caggtgtatgttaatggcaaatgtattggaggtggcactgacacacgtaatcttgaaagg gagggcaagttactgaaattggttcaagagtgtaatctcagtgctgcaacatag |
| Q01I75 | UniRef cluster | -------------------------------------------------MALAKAKETVASAPVVVYSKSYCPFCVRVKKLFEQLGATFKAIELD----GESD-----GSELQSALAEWTGQRTVPNVFINGKHIGGCDDTLALNNEGKLVPLLTEAGAIASSAKTTITA---------------------- | atggcgctcgccaaggccaaggagaccgtcgcctccgctcccgtcgtcgtctacagcaag tcttactgtcctttttgcgtccgtgtgaagaagttgttcgagcagcttggagcaactttc aaggccattgagttggatggggagagtgatggatctgagctgcagtcggcacttgctgaa tggactggacaaaggactgttccaaatgtcttcatcaatgggaagcatattggtggctgt gatgatactttggcattgaacaatgaagggaagctggtgcctctgctgaccgaggctgga gcaattgccagttctgcaaagacgacaatcaccgcatag |
| A3AVF9 | UniRef cluster | -------------------------------------------------MALAKAKETVASAPVVVYSKSYCPFCVRVKKLFEQLGATFKAIELD----GESD-----GSELQSALAEWTGQRTVPNVFINGKHIGGCDDTLALNNEGKLVPLLTEAGAIASSAKTTITA---------------------- | atggcgctcgccaaggccaaggagaccgtcgcctccgctcccgtcgtcgtctacagcaag tcttactgtcctttttgcgtccgtgtgaagaagttgttcgagcagcttggagcaactttc aaggccattgagttggatggggagagtgatggatctgagctgcagtcggcacttgctgaa tggactggacaaaggactgttccaaatgtcttcatcaatgggaagcatattggtggctgt gatgatactttggcattgaacaatgaagggaagctggtgcctctgctgaccgaggctgga gcaattgccagttctgcaaagacgacaatcaccgcatag |
| A2XVD7 | UniRef cluster | -------------------------------------------------MALAKAKETVASAPVVVYSKSYCPFCVRVKKLFEQLGATFKAIELD----GESD-----GSELQSALAEWTGQRTVPNVFINGKHIGGCDDTLALNNEGKLVPLLTEAGAIASSAKTTITA---------------------- | atggcgctcgccaaggccaaggagaccgtcgcctccgctcccgtcgtcgtctacagcaag tcttactgtcctttttgcgtccgtgtgaagaagttgttcgagcagcttggagcaactttc aaggccattgagttggatggggagagtgatggatctgagctgcagtcggcacttgctgaa tggactggacaaaggactgttccaaatgtcttcatcaatgggaagcatattggtggctgt gatgatactttggcattgaacaatgaagggaagctggtgcctctgctgaccgaggctgga gcaattgccagttctgcaaagacgacaatcaccgcatag |
| Q6CUY6 | UniRef cluster | ---------------------------------------------MPSAATIARVQGLINSSKIFVASKTYCPYCQATLKTLFEEKKVDKK----LATVLQLNQLE-DGSDIQDALAEITGQKTVPNIFINGKHIGGNSDLQELNN----SGDLDKLLASL------------------------------- | atgccaagtgccgcaacaatcgctcgtgtccaaggtttaatcaactcatctaagatcttt gtcgcttcaaagacatactgtccatactgccaagccactttgaaaactttgtttgaagaa aagaaggtggacaaaaagttggctactgttttgcaattgaaccaactagaagatggttcc gatattcaggatgctttggcagaaattaccggccaaaagactgttccaaacatctttatt aatggcaagcacatcggtggtaactctgatttgcaagagttgaacaactccggtgatttg gataagttgttggcttccttataa |
| P25373 | UniRef cluster | ---------------------------------------------MVSQETIKHVKDLIAENEIFVASKTYCPYCHAALNTLFEKLKVPRS----KVLVLQLNDMK-EGADIQAALYEINGQRTVPNIYINGKHIGGNDDLQELRE----TGELEELLEPILAN---------------------------- | atggtatctcaagaaactatcaagcacgtcaaggaccttattgcagaaaacgagatcttc gtcgcatccaaaacgtactgtccatactgccatgcagccctaaacacgctttttgaaaag ttaaaggttcccaggtccaaagttctggttttgcaattgaatgacatgaaggaaggcgca gacattcaggctgcgttatatgagattaatggccaaagaaccgtgccaaacatctatatt aatggtaaacatattggaggcaacgacgacttgcaggaattgagggagactggtgaattg gaggaattgttagaacctattcttgcaaattaa |
| A6ZTF5 | UniRef cluster | ---------------------------------------------MVSQETIKHVKDLIAENEIFVASKTYCPYCHAALNTLFEKLKVPRS----KVLVLQLNDMK-EGADIQAALYEINGQRTVPNIYINGKHIGGNDDLQELRE----TGELEELLEPILAN---------------------------- | atggtatctcaagaaactatcaagcacgtcaaggaccttattgcagaaaacgagatcttc gtcgcatccaaaacgtactgtccatactgccatgcagccctaaacacgctttttgaaaag ttaaaggttcccaggtccaaagttctggttttgcaattgaatgacatgaaggaaggcgca gacattcaggctgcgttatatgagattaatggccaaagaaccgtgccaaacatctatatt aatggtaaacatattggaggcaacgacgacttgcaggaattgagggagactggtgaattg gaggaattgttagaacctattcttgcaaattaa |
| A7TFA6 | UniRef cluster | -------------MGGFSDLDFLTLAIVVGIAFLTRTFLTSSPKKMVSQATVSRVKELIGQKKVFVAAKSYCPYCQASLQTLFTDYHVPKD----KSLVLQLNQME-DGDDIQAALAEITGQRTVPNIYIDGKHIGGNSDLQQLKS----SGKLDELLKAALA----------------------------- | atgggtggtttttcagatcttgattttctaacattagcaattgtggtcggtatagcattt ctaactaggacattcctaacaagctcaccaaaaaagatggtctctcaagcaacagtttca agagttaaagaattaattggccaaaagaaggtttttgttgccgcaaaatcttattgtcca tattgccaagctagtttgcaaactttattcactgattatcatgttccaaaggacaaatct ttggttttgcaattaaaccaaatggaagatggtgatgacattcaagctgctttagctgaa attactggtcaaagaactgtgccaaatatttacattgatggtaaacatattggtggtaac agtgacttgcaacaattgaagtcttctggtaaacttgatgaattgttgaaagctgctttg gcttaa |
| Q751W5 | UniRef cluster | ---------------------------------------------MVSPSVIKQVQALIQQNRVFIASKTYCPYCQAAKRTLLEEKRVPAS----AVKLLELDTMGEEGAVIQAALQELSGQRTVPNIYINGRHVGGNSDLEALKA----SGELDQLLEEALRE---------------------------- | atggtttccccttcggttattaaacaggtgcaggcgctaatccagcagaaccgcgtgttc attgcatccaagacgtactgtccgtattgccaggcggcaaagcgtacgttgctggaggag aagcgcgtcccggcaagcgcagtaaaactgttggagcttgacaccatgggcgaggagggc gcggtgatccaagcggcgttgcaggagctgagcgggcagcgcaccgtgcccaacatctac atcaacgggcgccatgtgggtggcaacagcgacctcgaggcgctgaaggcgtctggcgag ctggaccagctgttggaggaggcgctacgcgagtaa |
| A7TFF2 | UniRef cluster | ---------------------------------------------MVSQATIEKVKTMIGEKPVFVAAKSYCPHCRATRETLFEEYNLPRE----KALVLELDLMT-DGAEIQEALAEITHQDTVPNIFIYGQHVGGNSDLQALKK----DGQLKEMLDPVCQ----------------------------- | atggtcagtcaagcaaccatcgaaaaagttaaaacaatgattggtgaaaaaccagtgttc gttgcagccaagtcgtactgtcctcattgcagagcaacaagagaaaccctatttgaagag tacaacctaccaagggaaaaggctttagttttagaattagatttgatgacagatggtgct gaaattcaagaagcccttgctgaaataactcatcaagatactgtgccaaacatcttcatc tatggccaacatgttggtggtaattcagatttgcaagcattgaagaaggatggtcaattg aaggaaatgttagaccctgtctgccaataa |
| A2RAY5 | UniRef cluster | ----------------------------------------------MSAAAKTKAQTLINENGVVVFSKSYCPYCTASKNLLNELGAKYTT--------LELDQLP-DGADLQDALQEISNQRTVPNIFISQKHIGGNSDLQSKK-----NGELKGLLEAAGAL---------------------------- | atgtccgccgccgctaagactaaggcccagaccttgatcaacgagaatggcgttgtcgtc ttctccaagtcctactgcccctactgcaccgcaagcaagaacttgctgaatgagctcggc gcgaagtacactactctcgagttggaccagctgcccgacggagccgacctccaggacgct ctccaggaaatctccaaccagcgcaccgtccccaacatcttcattagccagaagcacatt ggtggaaactcggacttgcagagcaagaagaatggagagttgaaggggctgttggaggct gctggggctttgtag |
| A1D419 | UniRef cluster | -----------------------------------------------MSAAKTKAQNLINENAVVVFSKSYCPYCNASKRTLKNLGAKFYA--------LELDEID-DGTEIQNALYEITQQRTVPNIFIGQKHIGGNSELQAK------SAQLPALLKEAGAL---------------------------- | atgtcagccgcaaagaccaaagctcagaacctgattaacgagaatgccgttgtggtcttc tcgaaatcctactgcccctactgcaacgccagcaagaggacgctcaagaacctaggcgcc aagttttatgctctggagttggatgagattgacgacggaactgagattcagaatgcgctg tatgaaatcacgcagcagcggaccgtgccgaatatcttcatcgggcagaagcatattggt gggaactcggagttgcaggccaagtctgcgcagttgccggctttgttgaaggaggcgggt gcgttgtag |
| Q4WJG1 | UniRef cluster | -----------------------------------------------MSAAKTKAQNLINENAVVVFSKSYCPYCNASKKTLKDLGAKFYA--------LELDEID-DGREIQNALYEMTQQRTVPNIFIGQKHIGGNSELQAK------SAQLPALLKEAGAL---------------------------- | atgtcggccgcaaagaccaaagctcagaatctgatcaacgagaatgccgttgtggtcttc tcgaaatcctactgcccctactgcaatgccagcaagaagacgctcaaggacctaggtgcc aagttttatgctctggagttggatgagattgacgacggaagagagattcagaatgcgctg tatgaaatgacgcagcagcggactgtgccgaatatctttatcgggcagaagcacattggt gggaactcggagttgcaggccaagtctgcgcagttgccggctttgttgaaggaggcaggt gcgttgtag |
| B0XPH3 | UniRef cluster | -----------------------------------------------MSAAKTKAQNLINENAVVVFSKSYCPYCNASKKTLKDLGAKFYA--------LELDEID-DGREIQNALYEMTQQRTVPNIFIGQKHIGGNSELQAK------SAQLPALLKEAGAL---------------------------- | atgtcggccgcaaagaccaaagctcagaatctgatcaacgagaatgccgttgtggtcttc tcgaaatcctactgcccctactgcaatgccagcaagaagacgctcaaggacctaggtgcc aagttttatgctctggagttggatgagattgacgacggaagagagattcagaatgcgctg tatgaaatgacgcagcagcggactgtgccgaatatctttatcgggcagaagcacattggt gggaactcggagttgcaggccaagtctgcgcagttgccggctttgttgaaggaggcaggt gcgttgtag |
| Q4P4L5 | UniRef cluster | ------------------------------------------------MAAKQAAEKLISEHLVAVFSKSYCPYCSQAKSVIEKLGLDKSK-----VGILELDQMGSEGSDIQAYLLDKTSQRTVPNIFINQKHLGGCSDLLDAQKSGKLQQLLQ------------------------------------- | atggcggctaaacaggcagctgaaaagctcatctcggagcacctcgtggcggtcttcagc aagagctattgtccttactgctcacaggccaagagtgtcatagaaaagctcggtctggac aagagcaaagtcggaattctcgagctcgaccagatgggttccgaaggcagcgacatccag gcttacctcctcgacaagaccagccagcgtacggtccccaacatctttatcaaccagaaa cacctcggtggctgctccgacctgctcgacgctcagaagagtggaaagctccaacagctc ctccagtag |
| A8Q7Q7 | UniRef cluster | ------------------------------------------------MVAKQIAEKLISENAIAIFSKSYCPFCKRAKEVISGLSVEPSK-----IGTLELDEVN-DGPEIQNYLAEKTGQRTVPNIFISGKHVGGCDDLLRAQQSGELQQMVGKL----------------------------------- | atggtggcaaagcagattgctgaaaaactgatttctgaaaacgctattgcgatcttcagc aagtcctactgcccattctgcaagcgtgcgaaggaagtgatttccggcttgagcgtggaa cccagcaagatcggtactctggagctagatgaggtcaacgatggaccagagatccagaat taccttgctgagaagaccggccagcgtacggtgccaaacatctttatttcaggcaagcac gttggtggctgcgacgaccttctgcgtgctcagcaaagcggtgagctccagcagatggtg ggaaagctttag |
| Q1E158 | UniRef cluster | -----------------------------------------------MSAAKQKAQTIIADNAVVVFSKSYCPYCKATKSLLSSEGAKYFT--------MELDQVD-DGAAIQAALEEITNQRTVPNIFIDHKHIGGNSDLQAR------KSELPALLKAAGALQA-------------------------- | atgagcgccgcaaagcaaaaggcccaaaccatcatcgcggacaacgccgtcgttgtcttc tccaaatcctactgcccatactgcaaggccaccaagtccctgctgtcgtccgaaggggcc aagtacttcaccatggaattagaccaagtcgatgatggcgccgctatccaagccgctctc gaggagatcaccaaccagcgcaccgttcccaacatcttcatcgaccacaagcacatcggt ggaaactctgacttgcaagcccgcaagtctgaattgccggctttgctcaaggctgctggt gcgttgcaggcatga |
| A1CR54 | UniRef cluster | -----------------------------------------------MSTAKAKAQNLINDNAVVVFSKSWCPYCKASKQTLNELGAKFYA--------LELDQID-DGTEIQNALYEITQQRSVPNIFIGQKHIGGNSDLQAK------KAELPQLLKAAGAL---------------------------- | atgtccaccgcaaaggccaaggcccagaacctaatcaacgacaacgcagtcgtcgtcttc tccaagtcctggtgcccctactgcaaggccagcaagcagacgctcaacgagctcggcgcc aagttctatgccctcgagctggatcagattgacgacggaaccgagatccagaacgcgctg tacgaaatcacccagcagcgcagcgtgccgaatatcttcatcggccagaagcatatcggg ggtaactcggatctgcaggccaagaaggcggagttgccgcagttgttgaaggcggcgggt gcgctgtga |
| O36032 | UniRef cluster | -----------------------------------------------MSSVESFVDSAVADNDVVVFAKSYCPYCHATEKVIADKKIKAQV--------YQIDLMN-NGDEIQSYLLKKTGQRTVPNIFIHQKHVGGNSDFQALFK----KGELDSLFNTA------------------------------- | atgtctagtgttgaatcatttgttgactctgccgttgctgacaacgatgttgttgttttt gccaagtcttactgcccttactgccatgctactgaaaaggtgatcgctgataagaaaatt aaggctcaggtataccagattgaccttatgaacaacggtgatgagattcaatcttactta ttgaagaagaccggtcaacgtaccgtacccaatatctttattcaccaaaagcatgttggt ggtaactcggatttccaagcattattcaagaagggcgagctcgactctctttttaatact gcctaa |
| B2B5H0 | UniRef cluster | ------------------------------MSFFFRRFFSSATSPATMDAAQKKAQQLIDDNAVMVFSKSYCPYCNNTKRLLDSYDATYKA--------IELNQED-DGDDIQAALAKITGQRTVPNIFINKQHIGGNSDLEAVASKGKDGKKLEELLKEAGAL---------------------------- | atgtccttcttctttcgcagattcttctcctcagcaacctcaccagcaaccatggacgcc gcccagaagaaagcccagcagttgatcgatgacaatgctgtcatggtctttagcaagtcc tactgcccctactgcaacaacaccaagcgcctcctcgactcctacgacgccacgtacaag gccatcgagctcaaccaggaggatgacggcgacgacatccaggccgcgctcgccaagatc accggccagaggacggtgccaaacattttcatcaacaagcagcacattggtggcaacagc gatctcgaggccgtggccagcaagggcaaggacggcaagaagcttgaggagttgttgaag gaggctggtgctctctaa |
| A8N3H9 | UniRef cluster | -----------------------------------------------MASISELVDSTIEKNRVVIFSKSYCPYCRKAKNLFAEKFPQVEPKVLE----LDELD---NGSAIQDYLQQKTGQRTVPNVFVESQHIGGSDDTKAALESGKLAKLLTAA----------------------------------- | atggcctcgatctctgaactcgttgactctaccatcgagaagaacagggttgtgatcttc tctaaaagctactgcccatactgccgaaaggcaaagaacctgttcgcggaaaagttcccc caggttgaacccaaagttctcgagctcgacgagttggacaacggatctgccatccaggac tacctccaacaaaagaccggtcagcgaactgttcccaacgtgtttgtcgaatcacaacat attggaggcagtgacgataccaaggcagccttggagtcgggcaaacttgccaaactcctg accgccgcgtag |
| Q2UU38 | UniRef cluster | -----------------------------------------------MSAAKIKAQGIINANAVVVFSKSYCPYCKSSKSLLSQLDAKYLT--------IELDEES-DGSAIQDALVEISGQRTVPNIFIKQKHIGGNSDLQAR------KSELPALLKDAGAL---------------------------- | atgtctgccgctaagatcaaagcccagggcatcatcaacgccaacgccgtcgtcgttttc tccaagtcctactgcccctactgcaaatctagcaagagcctccttagccagctggacgca aagtacctcaccatcgagctcgatgaagagagcgatggcagcgccatccaggacgccctc gtggaaatcagcggccagcgcaccgtccccaacattttcatcaagcagaagcatatcggt ggaaactcggatctgcaggctcgcaaaagcgagcttcctgccttgctgaaggacgctggt gctctttag |
| A9NKR9 | UniRef cluster | ------------------------MEKITRLSVLFVAMSALCMSASAATRQEKFVKTTISNNKIAIFSKSYCPYCARAKNVFKELNVT--PYVVE----LDLRD---DGGEIQQALSILVGRRTVPQVFIDGKHIGGSDDTLEAYQSGQLAKLVGQTDEDELR----------------------------- | atggagaagataactcgtctaagcgtgttgtttgtagcgatgtctgcactgtgcatgagc gcttccgcagcaacaaggcaagaaaagttcgtgaagaccaccatctccaacaacaagatc gctattttctccaaatcatattgtccgtattgtgcccgtgcaaagaatgttttcaaggag ttgaatgtgacaccatatgttgtggagcttgatttgagagatgatggaggagaaattcaa caggctttaagcatcttggtgggacgacgcactgtgccacaagtcttcatagacggcaaa catattggtggctctgatgatactttggaggcatatcaaagtggtcagctcgctaagctt gtaggccagacagatgaagacgagctgcgttaa |
| A1ECK0 | UniRef cluster | -------------------------------------------------MALPKAQETVSSNSVVVFSKTFCPFCVSVKELFQQLGVTFKAIELN----KESD-----GSDIQSALAEWTGQKTVPNVFIGGKHIGGCDSTTALHREGKLVPLLTEAGAVAKTAA--------------------------- | atggctttgcccaaggcccaggaaaccgtttcctccaattccgtcgtcgttttcagcaaa acgttttgtccgttctgcgtgagtgtcaaagagctgtttcagcaactcggagtcactttc aaggccattgaactgaacaaagaaagtgatggaagtgacattcaatcagctctagctgag tggactggacagaaaactgttccaaatgtcttcatcggagggaaacacattggtggctgc gattcaactactgcactgcacagggaagggaagctggttcctctgctcactgaagctgga gctgttgccaagacagctgcctga |
| Q6BT54 | UniRef cluster | ---------------------------------MFEYIKSWFVSPPVSPVIKAEVQNLIDSNKILIFLKSYCPYCDSTKDLIKSITSD--------FKVVELNTSA-NGRTIQDALREMTGQNTVPNIFINRKHIGGNSDLQALQG----AGKLKSLVN--------------------------------- | atgttcgaatatattaagtcatggttcgtttcacctccagtatcgcctgtaataaaggct gaagttcaaaatttaattgatagtaataaaatcttaattttcctgaagtcatattgtcca tactgtgattccaccaaagacttaatcaagtccataacgtccgactttaaagtggttgag ttgaatacttcggctaatggtagaacgatacaagatgctttacgtgaaatgacaggacaa aacactgttccaaatatttttatcaataggaaacatatcggtggcaattcagatctccag gctttacaaggagccggcaaacttaagtcattagttaactaa |
| Q0ZR58 | UniRef cluster | ---------------MGSQHRRRLAIAAVPVLLLLVVVSELSDSAGAANSVSAFVQNAILSNKIVIFSKSYCPYCLRSKRIFSELKEQ--PFVVE----LDQRE---DGDQIQYELLEFVGRRTVPQVFVNGKHIGGSDDLGAAVENGQLQKLLAAS----------------------------------- | atgggtagccagcatcgtcgccgtcttgccatcgcggcggtgccggtactcctattactc gtggttgtcagcgagctgtcagattcagccggcgctgcgaattcggtatcggctttcgtt cagaacgccatcttgtccaacaagattgtcatcttctccaagtcctactgcccgtattgc ttgcgttcgaaacgtattttcagcgaacttaaggagcagccatttgttgtggagcttgat cagagagaggacggagatcaaatccagtacgagcttctggaattcgttggtcgccgaact gtcccgcaagtttttgtaaacggcaagcacattggtggatcagatgatcttggagctgct gtggagaatggtcagttgcaaaagcttcttgctgcaagttga |
| Q2F5R3 | UniRef cluster | -------------------------------MGSQSGKITRSSKMAGSIDIQQFIKEAISKDKVVVFSKSYCPYCKLAKDVFEKVKQ--PIKVIE----LNERD---DGNTIQDNLAQLTGFRTVPQVFINGNCVGGGSDVKALYESGKLEPMLIG------------------------------------ | atgggttctcagtctggaaaaattacgcgttcatctaaaatggccggatctatagacatt caacagtttatcaaggaagctatctccaaagacaaagttgtagtgttctccaaatcttac tgtccttactgtaagctggcaaaagatgtttttgagaaagtgaagcaaccaattaaagtt attgagttgaatgaacgtgatgatggaaacaccattcaagataatctcgcacaactgact ggtttcagaactgtacctcaagtctttataaatggcaactgtgtgggaggtggctctgat gttaaagcattgtatgaatctggaaaattagaacctatgttaataggataa |
| A5DVU3 | UniRef cluster | ---------------------------------------------MVSQQTKQKVQKLIKEKPIFIAAKSFCSNSDQVKRTIEEITHTSTTEDDDQVYSINLDLVD-DGQEIQDALTELTGQTTVPNVFIGGEHIGGNTDVQKLKA----LGVLDSKINAVLL----------------------------- | atggtttcacaacagactaaacaaaaggttcaaaagctcattaaagaaaaaccaatcttt atcgctgcaaagtccttttgctcaaacagcgatcaagtaaagaggacaattgaagagatt acccacactagtacaactgaagatgacgatcaagtttactcgatcaacttggaccttgtg gatgatggacaagagattcaggatgcattgacagaattgactggccaaactacggtgccc aatgtctttattggcggcgaacatattggaggaaacactgatgtccaaaaattgaaggct ctgggcgtgttggattcaaagattaatgccgttttgttgtaa |
| A5DVV1 | UniRef cluster | ---------------------------------------------MSDAQAKQKVQKWIKEKPIFIAAKSWCPHCAQAEKTIDHITKD--------AFVVDMDLED-DGDAIQEAVTELTGQKTIPNIFIGGEHIGGNDDLQKLKR----EGKLQEKIDAALK----------------------------- | atgtctgacgcacaagcaaagcaaaaagtccagaaatggatcaaagaaaagcccattttc atagctgccaagtcatggtgtcctcattgtgcacaggcagagaagacaattgatcatatt acaaaggatgcatttgttgtggatatggatttggaagacgatggtgatgctattcaagag gccgtgactgaattaactggacagaaaaccattccaaacatttttattggcggagagcat attggaggaaacgatgatttgcagaaattgaaaagagagggcaagttgcaagagaagatc gatgctgcattgaagtaa |
| A9SVS0 | UniRef cluster | ---------------------------------------------------MQEIEKLVQENAVVVFSQSGCCMCHVVKRLFCSLGVGPTVHELD----ERKEG----GDMEKALLRLNN-KVALPTVFVGGKLVGGVDAVMAAHVSGNLVPRLKEAGALWL------------------------------ | atgcaggagatagagaagctggtgcaggaaaatgctgtggttgtgttcagccagagcggg tgctgcatgtgtcatgtggtaaagcgtctcttctgcagtctgggagtggggccaactgtg cacgaactcgatgaacggaaggaaggtggcgacatggagaaggcattgctgcgcctcaac aacaaagttgcgcttcctaccgtgtttgtgggcggcaaactggtggggggcgtcgatgct gtcatggctgcccacgtgagtgggaaccttgtcccccgcttgaaggaagccggagctctt tggctgtag |
| A9SBI3 | UniRef cluster | ---------------------------------------------------MQEIEKLVQENAVVVFSQSRCCMCHVVKRLFCNLGVGPTVHELD----ERKEG----VDMEKALLRLNN-TVVLPTVFVGGKLVGGVDAVMAAHVSGNLVPRLKEAGALWL------------------------------ | atgcaggaaatagagaagctagtccaagaaaatgctgtagtggtgttcagccagagcagg tgctgcatgtgtcatgtggtgaagcggctcttctgcaacttaggagtggggcctacagtg cacgagctcgatgagcgtaaggaaggtgttgacatggagaaagcattactgcgcctcaac aacacagttgtgcttcccaccgtgtttgtgggggggaaacttgtagggggagtcgatgct gtcatggctgcccatgtgagcggcaacctcgttccccgcttgaaggaagctggagccctg tggttgtag |
| Q05926 | UniRef cluster | -----------------------------------------------MSAFVTKAEEMIKSHPYFQLSASWCPDCVYANSIWNKLNVQDKVFVFDIG---SLPRNEQE--KWRIAFQKVVGSRNLPTIVVNGKFWGTESQLHRFEAKGTLEEELTKIGLLP------------------------------- | atgtctgcctttgttactaaagctgaagagatgatcaaatctcatccatatttccagtta tccgccagctggtgccccgactgcgtctatgctaattccatttggaataagttgaatgta caggacaaagttttcgtttttgatattggttcacttccaagaaacgaacaggaaaaatgg agaattgcgttccaaaaagttgttggtagcagaaacttaccaacgatagttgtcaatggt aaattctggggtactgagagtcaattgcatagatttgaagcaaaaggcactcttgaggag gaattgactaaaatcgggcttctgccttga |
| A7A1P3 | UniRef cluster | -----------------------------------------------MSAFVTKAEEMIKSHPYFQLSASWCPDCVYANSIWNKLNVQDKVFVFDIG---SLPRNEQE--KWRIAFQKVVGSRNLPTIVVNGKFWGTESQLHRFEAKGTLEEELTKIGLLP------------------------------- | atgtctgcctttgttactaaagctgaagagatgatcaaatctcatccatatttccagtta tccgccagctggtgccccgactgcgtctatgctaattccatttggaataagttgaatgta caggacaaagttttcgtttttgatattggttcacttccaagaaacgaacaggaaaaatgg agaattgcgttccaaaaagttgttggtagcagaaacttaccaacgatagttgtcaatggt aaattctggggtactgagagtcaattgcatagatttgaagcaaaaggcactcttgaggag gaattgactaaaatcgggcttctgccttga |
| A7TT26 | UniRef cluster | -----------------------------------------------MSDYNTKAKEIINSHQFVQFSANWCPDCVYANSVWSKFGVSDKVHVFDIG---NLSKDEQA--QWRDAFESVSKIRNLPTIFVNGKAWGTESELHKYESKGTLKDELSKIGLIN------------------------------- | atgtctgattacaatactaaggcaaaagaaattataaattctcatcaatttgttcagttt tctgcaaattggtgtcctgattgcgtttatgcaaattcagtatggtcaaaatttggtgta tctgacaaagttcatgtctttgatattggtaacctttccaaagatgaacaagcccaatgg agggatgcttttgaatctgtttccaagattagaaacctaccaactatctttgttaatggc aaagcttggggtaccgaatctgagttacataaatatgagtcaaaaggaactctcaaagac gaattatcaaaaataggcttgataaattaa |
| Q6FT68 | UniRef cluster | -----------------------------------------------MTDYVKEAKDMVANNRLFQFSASWCPDCVYARSIWNKYHVENQIHVFDIG---SLDKEIQA--KWRDAFEEVLGVRNLPTIVVDGKVWGTETRLHEVEDNGSLSDELTKMGFKL------------------------------- | atgactgattatgtgaaagaggcaaaggatatggttgccaataataggctattccagttt tctgcaagctggtgtcctgattgtgtctacgctagatcaatctggaataaatatcatgtc gagaatcaaatccatgtgtttgacatcggctccctagacaaggagattcaagcaaagtgg agagatgcctttgaagaagtattaggtgttagaaacctaccaacaattgtcgttgacggt aaagtttggggaactgaaaccagacttcatgaggtggaagacaatggttctttgagtgat gaattgactaagatgggattcaaattatga |
| Q6CW04 | UniRef cluster | -----------------------------------------------MSKYIAIAREVVSSHKFVQLSAGWCPDCVYSNSIWKKFGVTDKIFNYDIA---EVTNSRSEWNEIRDAFQKATGSRNLPTLYVDGKVWGTESELARFERNGTLKEELQKIGLVD------------------------------- | atgtctaagtatattgctatcgcccgtgaagtagtttcttctcacaagtttgttcaattg agtgcgggatggtgtcctgattgtgtatattccaattccatatggaagaaattcggcgtt acggacaagatcttcaattatgatattgctgaagtgactaacagtagatccgaatggaat gaaatcagagatgcatttcaaaaggccactggctcaagaaacttgccaactttgtacgtt gatggtaaggtttggggtactgaatcagagttagcgagatttgaaaggaatggtactttg aaagaagagttacagaagatcggactggtagattag |
| Q752J7 | UniRef cluster | --------------------------------------MCCRAAESRMSEYEEKARELVSQHKYLQLSASWCPDCVYANGVWERLGLQQQIVVFDIA---QEAKDKQEEGAWRDGFERATGSRNLPTLYVDGQVWGTERELHALEDAGTLRAELAKIGLDAK------------------------------ | atgtgctgtcgggcagcagagagcaggatgagcgagtacgaggaaaaggccagggagctg gtatcgcagcacaagtacctgcagctgtctgcgtcgtggtgtcccgactgcgtgtacgcc aacggggtgtgggagcggctggggctgcagcagcagattgttgtgttcgacatagcgcag gaggcgaaggacaagcaggaggagggagcgtggcgggacggcttcgagcgcgcgacgggg tcgcgtaacctgccgacgctgtacgtggacgggcaggtatgggggacggagcgggagctg cacgcgctcgaggacgcggggacgctgcgggcggaattggccaagatcgggctggatgca aaatag |
| Q3Y400 | UniRef cluster | -----------------------------------MGGSASTPPKPTLSEHSKKIVEEVKEHAVVLYTKDGCGYCVKAKNELYEDGIHYTEKNLN-----TVSKVIPNPQEYIQGLMDLTRQRTVPQIFICGKFVGGYTELNALRPNLAKILETCSVDNGETLRREYASKI--------------------- | atgggaggatctgcatcaacaccacccaagccaacactatcggaacattcgaaaaaaatt gttgaggaagtcaaggaacatgcggttgttctctacacaaaagatggatgtggttattgt gtgaaagctaagaatgagctttacgaggatggaattcattatacagaaaagaatctgaat acggtgtcaaaagtgattccaaatccacaagaatacattcaagggctcatggatttgaca cgtcagcgaactgttccacagattttcatttgtgggaaattcgttggtggatacacagaa ctcaacgctcttagaccgaatctcgctaaaattctggaaacttgctcagtggacaatggg gagacactccggagagaatatgcatcgaaaatttga |
| A7Q168 | UniRef cluster | ---------------------------MKESGMIEEQLGMDGEAHEKPETTYEAVRRLGSSNAVVVFSLSGCCMCHVMKQLLFGLGVGPTIVELD----KEKYG----SEMQSVLYQLAGGQHSVPAVFVGGKFLGGIETLMSCHINGTLVPLLKAAGALWL------------------------------ | atgaaggaatcaggcatgatagaggaacagcttgggatggacggtgaagcccatgagaag ccggagaccacgtatgaggcggtgaggaggctgggctccagcaatgcggtggtggtgttc agcctcagcggctgctgcatgtgccacgtgatgaaacagctgcttttcggccttggagta ggacccactatcgtggagctcgacaaggagaagtatggctccgaaatgcaatccgtcctg taccagctcgccggcggccagcattctgtcccggctgtctttgtgggtggcaagttcttg ggcggtatcgaaactctcatgtcttgtcacattaacggcaccttggtccctctcctgaaa gctgccggagctctctggctataa |
| A7PTY3 | UniRef cluster | ---------------------------------------------------MDRVEELARKNAAVIFTKSSCCMCHSIKTLFYDLGASPAIHELD----KDARG----REMEWALRRIG-CNPSVPAVFVGGKFVGSAKDVITSHVDGSLKQMLIAARAIWF------------------------------ | atggatagggtggaggagttggcgaggaagaatgctgcagtgattttcaccaagagctca tgctgcatgtgccacagcatcaagacactgttctacgatctgggtgcgagccctgcgatt catgagctcgataaggacgctagaggaagggaaatggagtgggctttgcggcggataggg tgcaacccctccgtccctgctgtgtttgtaggcggaaaatttgttgggtctgctaaagat gtgattaccagccatgttgatgggtctctaaagcaaatgctcattgcagccagagccatc tggttctag |
| O82255 | UniRef cluster | ---------------------------------------------------MDKVMRMSSEKGVVIFTKSSCCLCYAVQILFRDLRVQPTIHEID----NDPDC----REIEKALLRLG-CSTAVPAVFVGGKLVGSTNEVMSLHLSGSLVPLIKPYQSILY------------------------------ | atggacaaagtgatgagaatgtcttcagagaaaggagtggtgatcttcacgaagagctca tgttgtctctgctacgccgttcaaatcctgttccgtgaccttagggttcaaccaaccatc cacgagatcgacaacgacccggactgccgtgagatcgagaaggctcttctccggctcggc tgttccacggcggttccagctgtctttgtcggaggcaagcttgttggctccaccaatgaa gtcatgtcccttcaccttagtggctctcttgtcccattgatcaaaccctatcagtccatc ctttactag |
| Q1EPG2 | UniRef cluster | ---------------------------------------------------MDKVKKLVSQRAVVVFSISSCCMCHTVKSLLHDLGVNAAVHELD----EEPRG----REMETALAVLVRRNPLVPLVFIGGKLVGSTDRIMSLHLGGELVPLLHEAGALWV------------------------------ | atggacaaggtgaagaagttggtgtcccaacgagcggtggtagtcttcagcatcagctca tgctgcatgtgccatactgtgaagagcctcctccatgacctcggtgtcaatgctgcagta catgaacttgacgaggaaccaaggggaagagagatggagacggcgctagccgtgcttgtg aggcgtaatcctctggtgccgcttgtcttcatcggaggcaagctggttggatctactgat aggatcatgtcactgcaccttggtggtgaattggtgccacttcttcacgaggcaggtgct ctttgggtctga |
