# Supplementary material for: Evolution based on domain combinations: the case of glutaredoxins
Source: BMC Evol Biol. 2009 Mar 25;9:66. doi: 10.1186/1471-2148-9-66 (PMC2679010; doi:10.1186/1471-2148-9-66)
Supplement: Additional File 4 — Analysis of CGFS-GRXs sequences from archaea. Contains the supplementary table I, with compiled information about CGFS class GRXs in archaea. [file 1471-2148-9-66-S4.htm]

Supplementary file 5: Multidomain alignments


#### Alignment for Multidomain GRXs

|  |  |  |  |
| --- | --- | --- | --- |
| UNIPROT accession number | UNIPROT 90\% similarity cluster | Aligned Protein Sequence | DNA Sequence |
| Q9V2B3 | UniRef cluster | -------------------------------------------EEFFSKMVNPVKLIVFIG----KEHCQYCD-QLKQLVQELSELT---DK---LSYEIVDFDTPEGKELAEKY----RID----------RAPATTITQDGKDFG-VRYFGIPAGH-----EFAAFLEDIVDVSRAETDLMAESKEEVAKI- | atgggattgataagtgaggaggacaagaggataattaaggaggagttcttctcgaaaatg gtgaacccagtcaagctcatcgtgttcataggaaaggagcactgccagtactgtgatcag cttaagcaactggttcaagagctttcagagctaacagataagctaagctatgagattgta gactttgacaccccagaggggaaggagcttgcggagaagtacaggatagacagggcaccg gcaactacgataacccaggatgggaaggacttcggggttaggtacttcggaattccagcg ggacacgagtttgcggcattccttgaggatatagtggatgtaagtagggccgagactgac ctaatggcagagagcaaggaagaagttgcgaaaatagacaagaacgttaggatactagtt ttcgtcacgccaacgtgcccatactgtccactggccgttagaatggctcacaagttcgca atagagaacactaaggccgggaagggcaagattctaggggacatggtcgaggccattgag tacccagagtgggccgaccagtacaacgttatggccgttccaaagatagttatacaagtg gatggcgaggacaaggttcagttcgagggggcttatccagagaagatgttcctcgagaag ctacttgcagcacttagctag |
| Q9V2B3 | UniRef cluster | -------------------------------------------KEEVAKIDKNVRILVFVT-----PTCPYCP-LAVRMAHKFAIENTKAGK---GKILGDMVEAIEYPEWADQY----NVM----------AVPKIVIQVD--GEDKVQFEGAYPEK-----MFLEKLLAALS-------------------- | atgggattgataagtgaggaggacaagaggataattaaggaggagttcttctcgaaaatg gtgaacccagtcaagctcatcgtgttcataggaaaggagcactgccagtactgtgatcag cttaagcaactggttcaagagctttcagagctaacagataagctaagctatgagattgta gactttgacaccccagaggggaaggagcttgcggagaagtacaggatagacagggcaccg gcaactacgataacccaggatgggaaggacttcggggttaggtacttcggaattccagcg ggacacgagtttgcggcattccttgaggatatagtggatgtaagtagggccgagactgac ctaatggcagagagcaaggaagaagttgcgaaaatagacaagaacgttaggatactagtt ttcgtcacgccaacgtgcccatactgtccactggccgttagaatggctcacaagttcgca atagagaacactaaggccgggaagggcaagattctaggggacatggtcgaggccattgag tacccagagtgggccgaccagtacaacgttatggccgttccaaagatagttatacaagtg gatggcgaggacaaggttcagttcgagggggcttatccagagaagatgttcctcgagaag ctacttgcagcacttagctag |
| Q980T5 | UniRef cluster | -------------------------------------------EAIKSKLNGKVKIETVVT-----PSCPYCP-YAALMAHMVAFEACRAGK---CNVISEVIEAYENQDIAEKY----QVM----------SVPAIAINES------IEFIGVPYEE-----NFINAILEKQKIS------------------ | atggaacaagaatatgccgaactatttagtgatgaagttaagaatgcgttacaagacgca ttaaaagatatgaaaaatccagtagatgtttatgttttcgtagacaatacagactctaat tgccaatattgcggtgttactactaaatttctggaattcatggctagtgctgcaccaaaa gatagtaatggtagtttattaaaggttcatatagtggatagagcaaatgaaaatagcaga aaaattttcgaagagtttaacgtggaaagagttcctactgtggcattttataatgggtat attagatggactggagctcctttaggtgaggaaataagagcattagttgagactatagta aggctttcacaaggtgaaagtggtctaagtcaagaaaccgtagaagctataaaatctaaa ctgaatggtaaggtaaagatagaaacggtagtcacaccatcttgtccatactgtccatat gcagcgttaatggcgcatatggttgcttttgaggcttgcagagcagggaagtgcaatgtt atatctgaagtgatagaagcttatgaaaatcaagatattgcggagaaatatcaagtaatg tcagtaccagctatagctataaatgaatctatagaatttataggagtaccctacgaggag aactttataaatgcaattttagagaagcaaaagataagctaa |
| Q97CA1 | UniRef cluster | -------------------------------------------GEFDKYLKNDVDLVVFTSN---DENCRYCK-ETVQLATEVSEIN---PK---IHLKVYNFD--EDKDQVKAY----GVE----------KYPATIVSKAGVEDGRIVYYGLPSGY-----EFGSLIEDLKNVS------------------ | atggcaactttaataagaaaggaagacagagaatatttgaagggtgagtttgacaaatac ctgaaaaatgatgtggatttagtagttttcacatctaacgatgaaaactgcagatactgc aaagaaactgtacagcttgctactgaagtttctgagataaacccgaagatccacctcaaa gtatacaacttcgatgaggacaaggatcaggtaaaggcatatggtgttgagaaatatcct gcaactatcgtttctaaggctggagtcgaagatggaagaatagtctattacggattgcct tcaggatatgaattcgggtctctgatcgaagacctaaagaacgtttctgttggtgatgct gatgtttcttcaaaggcagcagaacttatatcaaagatcgataagcctataacgataaag gtctacgtaacccctacatgcccatattgcccaagggctgtaggtaccgcccataagttt gcattaatgaatccaaacataaagggtgaaatgatcgaggccttggagttcgaaaatgaa gcagaagaagtaggggtatcaagcgtaccacatatagtaataaacaatgatgtgacgttc ataggcgcatatcctgacgaccagttcgctgaatacgtaatggaagcttacgatcaccag taa |
| Q97CA1 | UniRef cluster | -------------------------------------------AELISKIDKPITIKVYVT-----PTCPYCP-RAVGTAHKFALMN--------PNIKGEMIEALEFENEAEEV----GVS----------SVPHIVIN------NDVTFIGAYPDD-----QFAEYVMEAYDHQ------------------ | atggcaactttaataagaaaggaagacagagaatatttgaagggtgagtttgacaaatac ctgaaaaatgatgtggatttagtagttttcacatctaacgatgaaaactgcagatactgc aaagaaactgtacagcttgctactgaagtttctgagataaacccgaagatccacctcaaa gtatacaacttcgatgaggacaaggatcaggtaaaggcatatggtgttgagaaatatcct gcaactatcgtttctaaggctggagtcgaagatggaagaatagtctattacggattgcct tcaggatatgaattcgggtctctgatcgaagacctaaagaacgtttctgttggtgatgct gatgtttcttcaaaggcagcagaacttatatcaaagatcgataagcctataacgataaag gtctacgtaacccctacatgcccatattgcccaagggctgtaggtaccgcccataagttt gcattaatgaatccaaacataaagggtgaaatgatcgaggccttggagttcgaaaatgaa gcagaagaagtaggggtatcaagcgtaccacatatagtaataaacaatgatgtgacgttc ataggcgcatatcctgacgaccagttcgctgaatacgtaatggaagcttacgatcaccag taa |
| Q8ZVB4 | UniRef cluster | -----------------------------------RQKTRAELSTLAQGAPKRVYILTVVT-----PSCPYCP-YAVLMANMFAYES----K---GKVVSVVVEAYENPDIADMY----GVT----------GVPTVILQAEDAAVGDVEFVGVPPEH-----ELLARVKNHMGLS------------------ | atggctgtgcctataggagggccggaagaggtgcctcatattgaagtcgacgaggagact aaagaaattataaaggaaatgttatcgcaaatggaaaacccagtaaatattaacttcttc actagtcccaactgcgccgggagggagactaattggtgcatccccacggaggagttgctg gatctcctcgtacagctggccccacagggaaaactcatagttaacaaatacaacgcagaa aaagacgtagaggcctttaagaaattcggcgtagagccccagagagtgcccgtgatttac ttcggcgaggggtttattagatatttaggcgcccctatgggggaggaggtaagggcgttt atagagactgtggttaggctaagcactgggaagacaggccttaggcaaaagacaagggct gagctctcaacgctggctcaaggcgcgccaaaaagagtatacatattgactgttgtcact cccagctgtccctactgtccctacgcagtgcttatggcgaatatgtttgcatatgaaagt aagggaaaggtcgtgtctgtagtagtagaggcgtacgagaacccagatattgccgacatg tatggcgtcactggcgtgcccacagtgatactacaagcggaagatgccgccgttggagat gtggagttcgtcggcgtgccgcctgagcacgagttattagcccgtgttaaaaaccacatg ggtctctcttaa |
| Q8U2V4 | UniRef cluster | -------------------------------------------PRIEAEEFESSKVHFYMYG---AYWCPHCRNVKESIKEHFGEET--LTY---YEIEGVEHNKNKFEELYLLT----GIS----------GIPATAIFYEGEIYAVIEGEFDVSKT----WQIITEAKNQGGVILVTSGKAYILKW------ | atgaagctgaaagccttatttttattgctggctttgggtagtttaatcgttcccaggata gaggcagaagaatttgagtcatctaaggtgcacttctacatgtacggtgcctactggtgt ccccactgccgtaacgttaaggaaagtataaaggaacacttcggagaggaaacactaacg tattatgaaatagagggggtggagcacaacaaaaacaagtttgaagaactctatctgcta acgggtataagcggtattcctgctacggctatattctatgagggggaaatctatgcggtg atagagggagaatttgacgtctccaaaacatggcagattataacagaggccaaaaaccaa ggtggagtgattctagtgaccagcggtaaagcatacattttgaaatgggacagtcaggaa gtgcagaagttaaaggaaatctttttgagtggaaaagtgaatgaaacacagaaaaaagca gaagaaaaaagcatatgtgggcctgcgagcgtgcttctcctatccatgggtgcgatctac cttagaaagcgtaattattcaacagaagtgtccagtaatgtataa |
| Q74MG6 | UniRef cluster | -------------------------------------------HKTEPKHYKHYNIYVVYS-----PYCPHCH-NLLKTLDELGIKAITIDY----REFP---KTPYYKFVAKYF----N------------GVPLVFAKTKN----QLIIISGYPSEIQDNNGYYYGKEYEIELCKKLGGKP----------- | atgaaggcgaagaagttattaatattcgtgccattgttgttactgcctttattaacttta ttaatgcacaaaaccgaacctaagcattataagcattataatatatatgtggtttactct ccttattgtccacattgccataatttgcttaaaactttagatgaattaggaattaaagct ataacaattgattatagggaatttccgaaaactccttattacaaatttgttgccaaatat tttaatggtgttcccttggtttttgctaaaaccaaaaaccagctaattataataagcggg tatccatcggaaatacaagataataatggttactattatggaaaagaatatgaaattgaa ttatgcaaaaaattgggaggcaaacccgtatatattaataatagttattatttctgtgaa ataaataacacaatattgggaaacagaaaagctattgaatggctaataaatatatgtaaa agccaaggatgcgaaaatctaactatcataaaatag |
| Q6L248 | UniRef cluster | -------------------------------------------DEFNGKLKEPVDLVVFTSN---KSDCKYCK-ETVQLAEELSSIN---EK---INLIKYVYE--DNKDAVNDY----GVE----------KYPATIVAKHGQKDGRIVYYGIPSGY-----EFGSLIEDIENVS------------------ | atggcactgataagagatgaggacaggaaatatttagaggatgaattcaatggcaagtta aaggaaccagttgaccttgttgttttcacatcaaacaagagtgactgcaagtactgcaag gaaacagtccagcttgcagaggaattatcatcaataaatgaaaagataaatttaataaag tacgtttacgaggataacaaggacgctgtaaatgattatggtgttgagaaatatcctgca acaatagtcgcaaagcatggccagaaggacggaaggatagtatactatggaattccatct ggctacgaattcggatcattaatagaggacattgaaaacgtctcaatgaacgaggcagat gtatcaaagcacgcaatggagttaatatcaaaggttgacaggccattaacaataaaggtc tatgtaacaccaacatgccagtactgcccaagggccgttggcacagcacataagttcgca ctattaaacaagaatataaaagccgaaatgatagaatcactggaattcgacaaggaggct gaggaggtcggggtttcagcggtaccacacgttgttataaacgacgatgtaacctttgtt ggtgcacagccggatgatcagttcgcagaatttataatggaagcatacaaccaccaggat taa |
| Q6L248 | UniRef cluster | -------------------------------------------MELISKVDRPLTIKVYVT-----PTCQYCP-RAVGTAHKFALLN--------KNIKAEMIESLEFDKEAEEV----GVS----------AVPHVVIN------DDVTFVGAQPDD-----QFAEFIMEAYNHQD----------------- | atggcactgataagagatgaggacaggaaatatttagaggatgaattcaatggcaagtta aaggaaccagttgaccttgttgttttcacatcaaacaagagtgactgcaagtactgcaag gaaacagtccagcttgcagaggaattatcatcaataaatgaaaagataaatttaataaag tacgtttacgaggataacaaggacgctgtaaatgattatggtgttgagaaatatcctgca acaatagtcgcaaagcatggccagaaggacggaaggatagtatactatggaattccatct ggctacgaattcggatcattaatagaggacattgaaaacgtctcaatgaacgaggcagat gtatcaaagcacgcaatggagttaatatcaaaggttgacaggccattaacaataaaggtc tatgtaacaccaacatgccagtactgcccaagggccgttggcacagcacataagttcgca ctattaaacaagaatataaaagccgaaatgatagaatcactggaattcgacaaggaggct gaggaggtcggggtttcagcggtaccacacgttgttataaacgacgatgtaacctttgtt ggtgcacagccggatgatcagttcgcagaatttataatggaagcatacaaccaccaggat taa |
| Q5JGG9 | UniRef cluster | -------------------------------------------VDVNGTRIYLDQIHFYMYG---MKTCPHCRHMKEWIPEEFGADS--LTY---YELVNDETNSELFGQLAQLT----GIT----------GVPAIAITYNGTIQAIFEGEFNVSAT----PEIVATAMNANGVILFIGGEWYLLA------- | gtgaaaaaaactggaatactgctgatgctcctggtcttggtggggtttgcctccgcctgt ctgagttctgattcaaatacctccagccagacgtccacagaatctccaaaatacgtggac gtgaatggaacgagaatttacctggatcagatacatttttacatgtacggtatgaagacc tgtcctcactgcaggcatatgaaagagtggattcctgaagaatttggggcagattcactc acgtattatgagcttgtaaatgacgagactaactcagagctttttggacagcttgcacag ctaacaggcataaccggtgttcccgctatcgcaataacttacaacggtactatacaggca atatttgagggggagttcaacgtctctgcaacacctgagattgttgcaactgctatgaat gcaaatggagttatccttttcattgggggtgaatggtatcttttggcccgggataatcca aggtcagagacgctcatcgatgcccttcagacgattttcgtagagcacgaaagcgtcgat gttcagagcgttctgacaaagctgaagtctaactcaacgggttga |
| Q5JE30 | UniRef cluster | -------------------------------------------EEFFSKMTNPVKLIVFVG----KEHCQYCD-QLKQLVQELSELT---DK---LSYEVVDFDTEEGKKLAEQY----RID----------RAPATTITQDGKDMG-VRYFGLPAGH-----EFGAFLEDIVDVSNGTTDLAPDTKEAIHGV- | atgggactcattagcgatgctgacaagaaggtaattaaggaggagttcttctcaaagatg actaacccggtcaagctcatagtattcgtcgggaaggagcactgccagtactgcgaccag ctcaagcagctcgtccaggagctttccgagctcaccgacaagctgagctacgaggtcgtt gacttcgacactgaggagggcaagaagctggccgagcagtacaggatcgaccgcgctccc gccaccaccataacccaggacggcaaggacatgggcgtcaggtacttcggccttccagcc ggccacgagttcggagccttccttgaggacatcgttgacgtcagcaacggcacaaccgac ctcgctccggacaccaaggaggcaatccacggtgttgataaggacgtcaggatactcgtc ttcgttactccgacctgcccgtactgcccgctcgcggtcaggatggcccacaagttcgcc atcgagaacacccttgccggaaagggcaagatactcggcgacatggtcgaggcgatagag taccccgagtgggccgaccagtacagcgtcatggccgtcccgaagatagtcattcaggtg gacggcgaggacaaggttcagtttgagggagcttatcccgagaagatgttccttgagaag ctcctcgctgccctcgagtga |
| Q5JE30 | UniRef cluster | -------------------------------------------KEAIHGVDKDVRILVFVT-----PTCPYCP-LAVRMAHKFAIENTLAGK---GKILGDMVEAIEYPEWADQY----SVM----------AVPKIVIQVD--GEDKVQFEGAYPEK-----MFLEKLLAALE-------------------- | atgggactcattagcgatgctgacaagaaggtaattaaggaggagttcttctcaaagatg actaacccggtcaagctcatagtattcgtcgggaaggagcactgccagtactgcgaccag ctcaagcagctcgtccaggagctttccgagctcaccgacaagctgagctacgaggtcgtt gacttcgacactgaggagggcaagaagctggccgagcagtacaggatcgaccgcgctccc gccaccaccataacccaggacggcaaggacatgggcgtcaggtacttcggccttccagcc ggccacgagttcggagccttccttgaggacatcgttgacgtcagcaacggcacaaccgac ctcgctccggacaccaaggaggcaatccacggtgttgataaggacgtcaggatactcgtc ttcgttactccgacctgcccgtactgcccgctcgcggtcaggatggcccacaagttcgcc atcgagaacacccttgccggaaagggcaagatactcggcgacatggtcgaggcgatagag taccccgagtgggccgaccagtacagcgtcatggccgtcccgaagatagtcattcaggtg gacggcgaggacaaggttcagtttgagggagcttatcccgagaagatgttccttgagaag ctcctcgctgccctcgagtga |
| Q51760 | UniRef cluster | -------------------------------------------EEFFSKMVNPVKLIVFVR----KDHCQYCD-QLKQLVQELSELT---DK---LSYEIVDFDTPEGKELAKRY----RID----------RAPATTITQDGKDFG-VRYFGLPAGH-----EFAAFLEDIVDVSREETNLMDETKQAIRNI- | atgggattgattagtgacgctgacaagaaggtaattaaggaagagttcttttcaaaaatg gtcaatccagttaagctaatcgtttttgtgagaaaggaccactgtcaatactgtgaccag ctaaaacaactagttcaagagctttctgagctaacagacaagctcagttatgaaattgtt gacttcgacacaccagaaggcaaagagctagccaagaggtatagaatagacagagctcca gcaacaacaataacccaagatggaaaggactttggagtaaggtactttggattaccggca ggtcatgagttcgcagcattcttagaggacattgtggatgttagtagagaagaaacaaac cttatggacgagacaaaacaggccatcagaaacatagaccaggatgtaagaatattggtg tttgtaactccaacatgcccatactgtccacttgccgttagaatggctcacaagtttgcc attgaaaacacaaaagctgggaaaggtaagatacttggggatatggtcgaggccattgag tatccagagtgggctgaccagtacaatgtaatggcagtaccaaaaattgttattcaggtc aacggagaagacagagtagaatttgaaggagcttatccagagaaaatgttcttagagaag ttactctcagctctcagctga |
| Q51760 | UniRef cluster | -------------------------------------------KQAIRNIDQDVRILVFVT-----PTCPYCP-LAVRMAHKFAIENTKAGK---GKILGDMVEAIEYPEWADQY----NVM----------AVPKIVIQVN--GEDRVEFEGAYPEK-----MFLEKLLSALS-------------------- | atgggattgattagtgacgctgacaagaaggtaattaaggaagagttcttttcaaaaatg gtcaatccagttaagctaatcgtttttgtgagaaaggaccactgtcaatactgtgaccag ctaaaacaactagttcaagagctttctgagctaacagacaagctcagttatgaaattgtt gacttcgacacaccagaaggcaaagagctagccaagaggtatagaatagacagagctcca gcaacaacaataacccaagatggaaaggactttggagtaaggtactttggattaccggca ggtcatgagttcgcagcattcttagaggacattgtggatgttagtagagaagaaacaaac cttatggacgagacaaaacaggccatcagaaacatagaccaggatgtaagaatattggtg tttgtaactccaacatgcccatactgtccacttgccgttagaatggctcacaagtttgcc attgaaaacacaaaagctgggaaaggtaagatacttggggatatggtcgaggccattgag tatccagagtgggctgaccagtacaatgtaatggcagtaccaaaaattgttattcaggtc aacggagaagacagagtagaatttgaaggagcttatccagagaaaatgttcttagagaag ttactctcagctctcagctga |
| Q2FT67 | UniRef cluster | --------------------------------------------MTDTYLSHMPEVTVYSTQN-----CPYCR-LAKAFLDRNNI---PYRS---VDVGI---DRKAAKEMVELS----GQY----------GVPVIVA-GEE-------VIVGFDTDK--LRALFTTGKKPDMFDVIIVGA------------ | gtgaccgacacatacctatctcatatgccagaggtcacggtctattcaactcagaactgt ccatactgccggcttgcaaaagcgtttttagacagaaataatattccataccgctcggta gatgtgggaattgaccggaaagcagccaaggagatggttgaactctcaggacaatatggg gttccggtcatcgttgcaggtgaggaggtcatcgtcggattcgatacagacaaactcagg gccctgtttaccaccggaaaaaagccggatatgtttgatgtgattattgtcggtgcaggc cctgcaggcctgactgctgcgttatactgtgtcaggaagaatctgaaaacgcttatgata tcaccggacatcggaggtcaggctcttgagagctggaatattgagaattacatgggatac cggatgatcaccggtgatgacctgatggcgaagtttgaagagcagatccgggaactggag atcaggattgaacttgatcaggtcaactctcttctcccgaccagtggcggattctcggtg aaaaccgtatcagaccaggaatataaaggaaaaacaataatcctttcccagggaaaaaag ccaagaaaactcggtgtggcaagagaagaggagttcatcggaaggggtctttcagtctgt gcaacatgtgacgggccgatctttaaagagaaggtggttggagttgtcggtggggggaac tcagcgcttacgactgcacttgagatgagtggtattgcaaaagaagtgcatctcattgtc cggagcagcattcgggctgatgcagtgtacacctcacaatacgcgcaaaagcagaacatc atcacccataccggatatgaagtgaccgaacttatcggagatgaccggctttcaggcatt attattacaaaccgggagaccggtgagaagaagaccttaaagcttgacggattgttcacg gagataggatggattccaaatacttcctttgttgagggattgctcaaattaaatgatcag aaagagattgtgatagatattaactgccggaccagtgctcctggtatatttgctgccggg gatgtcacctcaattgcaggaaaacagatcatcattgcctgtggtgaaggggcaaaagca gctctttcagcatttgactatctgatgacacagtag |
| O57917 | UniRef cluster | -------------------------------------------EEFFSKMVNPVKLIVFIG----KEHCQYCD-QLKQLVQELSELT---DK---LSYEIVDFDTPEGKELAEKY----RID----------RAPATTITQDGKDFG-VRYFGIPAGH-----EFAAFLEDIVDVSKGDTDLMQDSKEEVSKI- | atgggactaataagtgaggaggacaagaggataattaaggaagagttcttctcaaagatg gtgaacccagtcaagctcatcgtcttcataggaaaagaacactgccaatactgtgatcag cttaagcaattagttcaggagctctcagagctgacagataagctgagctatgagatagtt gacttcgacactcccgagggaaaggagctagctgagaagtacaggatcgacagggcccca gcaactacaataacccaggatggaaaggacttcggcgttagatacttcggaattccagct ggacacgagtttgcagcatttcttgaggatatagttgatgtaagcaagggagacaccgat ttaatgcaggatagcaaggaggaggtttcaaagatagacaaagacgtcaggatattgatc ttcgtaacgccaacctgcccatactgtccattagccgttagaatggcccacaagttcgca atcgagaacacaaaagctggaaaaggaaagatccttggagacatggtggaagctatagag tatccagaatgggccgatcagtacaacgtcatggccgttccaaagatagtaatacaggta aatggagaggataaagtccaattcgagggggcttacccagagaaaatgttcctggaaaag ctactttcagccctaagctag |
| O57917 | UniRef cluster | -------------------------------------------KEEVSKIDKDVRILIFVT-----PTCPYCP-LAVRMAHKFAIENTKAGK---GKILGDMVEAIEYPEWADQY----NVM----------AVPKIVIQVN--GEDKVQFEGAYPEK-----MFLEKLLSALS-------------------- | atgggactaataagtgaggaggacaagaggataattaaggaagagttcttctcaaagatg gtgaacccagtcaagctcatcgtcttcataggaaaagaacactgccaatactgtgatcag cttaagcaattagttcaggagctctcagagctgacagataagctgagctatgagatagtt gacttcgacactcccgagggaaaggagctagctgagaagtacaggatcgacagggcccca gcaactacaataacccaggatggaaaggacttcggcgttagatacttcggaattccagct ggacacgagtttgcagcatttcttgaggatatagttgatgtaagcaagggagacaccgat ttaatgcaggatagcaaggaggaggtttcaaagatagacaaagacgtcaggatattgatc ttcgtaacgccaacctgcccatactgtccattagccgttagaatggcccacaagttcgca atcgagaacacaaaagctggaaaaggaaagatccttggagacatggtggaagctatagag tatccagaatgggccgatcagtacaacgtcatggccgttccaaagatagtaatacaggta aatggagaggataaagtccaattcgagggggcttacccagagaaaatgttcctggaaaag ctactttcagccctaagctag |
| B1Y9K2 | UniRef cluster | -------------------------------------------AALAQNAPKRVNVITVVT-----PSCPYCP-YAVLLANMFAYES----K---GKVVSVVVEAHENPDIADMY----GVT----------GVPTVILQAEDAAVGDVEFVGVPPEH-----ELLARVKNHMGLS------------------ | atgcatgtcggctgtgttgacatggcggttccaataggcggccctgaagaggtgccccat atcgaagttgatgaggagacgaaggaaatcatcaaggagatgctgtcgcagatggaaaac gtggtggaggtcaacttcttcaccagcgccaactgcggaggtagagagacaaactggtgc gtccctaccgaggagcttatcgatctgctcgcccagcttgcgccgcctggtaaactcaag gtgaacaagtacagctacgacaaggatcaagaggtcttcaagaaattcggaatagaaccc cagagggtccccgcggtaatatttggcgacgggtttgtcagatacctcggctcgcctata ggcgaggaggtgagggcctttatagagactgtggtgaggttgagcacggggaagacaggc ctgcgccagaagaccagatcggagctggcagcgctagctcagaacgccccgaagcgggtt aacgtgattaccgtggtgacgcccagctgtccctactgcccatacgcggtgctcttggcc aacatgttcgcgtatgagagtaagggcaaggtggtgtctgtcgtcgtcgaggcacacgag aaccctgacatagcggatatgtacggagtcacaggcgtgcccacggtgatactccaagca gaagacgccgccgtgggagacgtggagttcgtgggggtgccccccgagcacgagctactg gcccgtgtgaagaaccacatgggtctctcctag |
| A8MAS4 | UniRef cluster | -----------------------------------------------------MEVEVFVH-----PTCSTCH-SLIRLLKQWG----FIDK---VKIYD----TSIDPHAALER----GVR----------SVPSIFIDGD------LVFAGVVDFKR--LKSILDTGLHTERVMLSDEELIE---------- | atggaggtagaggttttcgtgcatccaacgtgctcaacatgccattccctaataaggcta ctgaagcagtggggattcatagataaggttaaaatctacgacacatccattgaccctcac gcagccttggagaggggtgttaggtcagtaccaagcatattcatagatggtgacttagtg tttgcgggtgttgttgactttaagaggcttaagagtattcttgatactgggctacatact gagagagtcatgttaagtgatgaggagttaattgagagattccttaggggtgttttggat tcagtggccactgcactatggctctacattaatgttaactgcagggccttcaccaatgat gtgaagttccttaaggcaataactaacttatccacctacagtgatggtaatttaaatagg ctagttgatattcttaagaatgacggtaactgcaggagtattgtggagaataataaggag aggttccttagggtaatagccactaacttcactagggaggtttactggcttaacggtaag gttaatggtactgatgtgttaggtaactataatcttcaatcaattgcccactgggccatg gttaggggttcagttagtagggttggtttagtgcctcatagccttaatgatgatgaattc aggggtaagattgaggagctttggtcatacattaaggctcatttcgatgaattaatgagt agggtcgcctcagagcaggaggagcttaggggtgattcggattgggtactgaggagttga |
| A8M8V0 | UniRef cluster | -------------------------------------------ASMINSDAKTVEVVTVVT-----PSCPYCP-YAVLLANMFAYES----K---GKVRSVVVEAYEEPDIADMY----GVT----------AVPTVVIRNE-GSTGDVEFVGVPPEA-----DLLKKVLSYSGFNP----------------- | atgtcagctacaccactagcaccacctactggtcctgaggaggttcatatagaggttgat gaggagactaagggtataataagggagatgctttcacaaatggttaacccagtgaccttc gatctcttcacaacaggcaattgcgcaggcagggacactaactggtgcacaccaacggag gagctccttgatctattatccagtttagcacctgagggtaagttaatagttaataagcat aggcttgatcagaataaggatgatggggctaagttcaatgttgaggagaatagggttccc gtaatatacattaatggaggcataataaggtactttggcgcaccactgggtgaggaggtt agggcattcatagagactataacaaggatatcaaccggtaaaacagggttaagggctagg acaaagagtaccttagcctcaatgattaactcagatgcgaagacagttgaagtagtaaca gtggttaccccatcatgcccatactgcccctacgccgtactgttagccaacatgttcgcc tatgaaagtaagggtaaggttaggtcagtggttgttgaggcctatgaggaaccggacata gcggacatgtacggtgtaacagccgtaccaactgtggtaattaggaatgagggctcaacc ggtgatgttgagttcgttggagtaccacctgaggctgacttacttaagaaggtactatca tacagtggttttaaccctcctggccagtag |
| A8M8V0 | UniRef cluster | -----------------------------------------MSATPLAPPTGPEEVHIEVD----EETKGIIREMLSQMVNPVTFDLFTTGN---CAGRDTNWCTPTEELLDLLS----SLA----------PEGKLIVNKHR-------LDQNKDDG---AKFNVEENRVPVIYINGGIIRYFGAPL------ | atgtcagctacaccactagcaccacctactggtcctgaggaggttcatatagaggttgat gaggagactaagggtataataagggagatgctttcacaaatggttaacccagtgaccttc gatctcttcacaacaggcaattgcgcaggcagggacactaactggtgcacaccaacggag gagctccttgatctattatccagtttagcacctgagggtaagttaatagttaataagcat aggcttgatcagaataaggatgatggggctaagttcaatgttgaggagaatagggttccc gtaatatacattaatggaggcataataaggtactttggcgcaccactgggtgaggaggtt agggcattcatagagactataacaaggatatcaaccggtaaaacagggttaagggctagg acaaagagtaccttagcctcaatgattaactcagatgcgaagacagttgaagtagtaaca gtggttaccccatcatgcccatactgcccctacgccgtactgttagccaacatgttcgcc tatgaaagtaagggtaaggttaggtcagtggttgttgaggcctatgaggaaccggacata gcggacatgtacggtgtaacagccgtaccaactgtggtaattaggaatgagggctcaacc ggtgatgttgagttcgttggagtaccacctgaggctgacttacttaagaaggtactatca tacagtggttttaaccctcctggccagtag |
| A8AAY4 | UniRef cluster | -------------------------------------------MSFGMEWTPEARAGLREA------LADMKGPVEALVFVEEG-----CEP-----------CEATIEMMQVMK---EESP---------KVNGKPYFDYK-------VFWRGKDDE---AFDKYKVDRVPSVLLLDGYIRY----------- | atgtcgttcggtatggagtggacgccggaggcgagagctgggctcagggaagctctggcg gacatgaaaggtccggtggaggccctagtcttcgtagaggaaggctgtgagccgtgcgag gctacgatagaaatgatgcaagtaatgaaggaagagagccctaaggtgaacgggaagccc tacttcgactacaaagtcttctggagggggaaggacgacgaggcgttcgacaagtacaag gtagatagggtgccctcggtcttgttgttggacggctacatccgctacaccggcatcccc gcgggagaggaagtgaagggcttcgtagaaaccgtaataaggatctctgagaatgagagc ggcttggacgaggagaccaaggaggggttgaagaaactcaagggctgttactacatcgag aacgtggtgaccccccagtgcccttattgcccctacgccgccttactaattaatatgttc gccttcgaggccaagaagcaaggcaacccgtgcgtggtggcagacacagtagaggcctac gagaacgaggacatagctgacaaatacaacgtcatgagcgttccggcgatagcgataaac gggaacgtggagttcgtgggagtcccctacgaagacgacttgttagccaagctgttcgag gtagagcccaaggggccttgtgaggacgaggtgtgcttcgtcaggccctaa |
| A8AAY4 | UniRef cluster | -------------------------------------------KEGLKKLKGCYYIENVVT-----PQCPYCP-YAALLINMFAFEAKKQGN---PCVVADTVEAYENEDIADKY----NVM----------SVPAIAINGN------VEFVGVPYED-----DLLAKLFEVEPKGPCEDEVC----------- | atgtcgttcggtatggagtggacgccggaggcgagagctgggctcagggaagctctggcg gacatgaaaggtccggtggaggccctagtcttcgtagaggaaggctgtgagccgtgcgag gctacgatagaaatgatgcaagtaatgaaggaagagagccctaaggtgaacgggaagccc tacttcgactacaaagtcttctggagggggaaggacgacgaggcgttcgacaagtacaag gtagatagggtgccctcggtcttgttgttggacggctacatccgctacaccggcatcccc gcgggagaggaagtgaagggcttcgtagaaaccgtaataaggatctctgagaatgagagc ggcttggacgaggagaccaaggaggggttgaagaaactcaagggctgttactacatcgag aacgtggtgaccccccagtgcccttattgcccctacgccgccttactaattaatatgttc gccttcgaggccaagaagcaaggcaacccgtgcgtggtggcagacacagtagaggcctac gagaacgaggacatagctgacaaatacaacgtcatgagcgttccggcgatagcgataaac gggaacgtggagttcgtgggagtcccctacgaagacgacttgttagccaagctgttcgag gtagagcccaaggggccttgtgaggacgaggtgtgcttcgtcaggccctaa |
| A7I8I7 | UniRef cluster | ------------------------------------------ESFIRKLWGKPRVVRVYAT-----PGCSGCR-AVKEYLKSKNV---EFTE---IDIAA---DERARTLVMEKT----GHL----------GSPYVQIDDT--------FIFGFDRKK--LDQLLQGT------------------------- | atggatgaaaaacagggaacgggaaaaaacgtaaaaaaagcgacctttgccggcggatgc ttctggtgcatgcagccgccgttccgggcgctcaacggtgtgatcgatgcggtatcggga tacgcaggcggaaaaaaggagaacccgacctacgaggaggtttcgggcgggacgaccggg cacctcgaatcggtgcaggtgacctatgacgaggataggatcccgtatgatactctcctt gacacgttctggaagcagatagacccgaccgatccggcagggcagttcgcggacaagggg tcgcagtacaaaaccgccatattctaccacgacgacgaacaaaaacggcaggcagaagaa tcgaaaaagaaggtcgaggcttcaggaaagtttgcacaccctgttgcaacagagatccgg ccgtacacgaatttctacccggccgaggaataccaccaggactatgataagaaaaatccc gggagataccagcagtacaaggcgttgtccggccgcgaatcattcatcaggaaactctgg ggtaaaccccgcgttgtccgggtctacgcgacaccgggatgttccggctgccgtgcggtt aaggaatacttaaaatcgaagaatgtggaattcaccgagatcgatatcgccgcagatgag agggcaaggacgcttgtcatggaaaaaaccggccacctcggttcgccctatgtccagatc gatgataccttcatcttcgggtttgaccggaaaaaactggaccagctcctgcagggaact taa |
| A7I4E7 | UniRef cluster | ---------------------------------------------------MMSTVTVYSTKN-----CPYCR-MAKAFLEKYGV---PYTA---IDVGA---DTAAAHKMIALS----GQR----------GVPVITV-DDE-------VIVGFDSQR--LNELFGTHAAGETYDVIIVGAGPGG-------- | atgatgagcacggtcaccgtttattccacgaaaaattgcccgtactgccggatggccaag gcattcctggagaagtacggcgttccctacacagccattgatgtcggcgccgataccgct gcggctcataagatgatcgcgctttcaggccagcgcggcgtaccggtaattaccgttgat gacgaggtgattgtcggctttgactcccagaggctcaacgagcttttcgggacccatgca gcgggagagacctacgatgtcatcatcgttggtgccggcccgggaggacttactgccgcg gtctactgcgcccgcaaactcttaaaaacgctcgttgtttccgagagtattggcgggcag gcgctggaatcgtgggccattgagaattacatgggctaccggatgatcaccggtgaagac ttgatgaagaagttcgaggagcaggtccggacccttgacgtgcgcctggagctcgaccgg gtaaacggcgttacaatggaggagaacctgtttgtggtaacaaccgtttccggcctttcc gtgaaggcaaagagcctgatcctcaccccgggaaaacagccccggaaactaggggttgaa aacgaggagaagtacctcgggcgcggtttgtccatctgctcgacctgcgatggcccgctt tttaaggataagaagatcgctgttgtgggcggcggaaactcggcgctccagaccgccgtt gagatgagcagcatcgcctcctccgtgagcctgcttgtccggagcacaatcaaggcggat ccggtgtacgcaaaaaagcttgaagggctaaagaacattaccgtgcacctccacacgcag atcaccgcgcttgccggcgatccgttcctccagcatatcacgattgcaaccgagggccag ggggaacagcgcattgatgttgatggcgtctttatcgaggttggctggacgccgaatacg gagatctttgatggcctggtggagctcaacgggaaaaaggagatcgtggtggatgtggac ggccacacgagccggcccggggtatttgcggcaggcgatgtgacatcggtaaagagcaag cagatcattatagctgccggtgaaggggcaaaggccgcgctcgaagcgtacgattacctg atgagcctggcctga |
| A6UTS1 | UniRef cluster | --------------------------------------------------MANNLLKLYVN-----STCPLCN-ILKNILEYNN---IKFELIN-IEDIE---ESDLNEEFKVFN----NIG-----DIKNIAFPSIVLGNE--------IIVGYNIEK--LEKLLNKKLLKPPVTEKSFID------------ | atggccaataatttattaaaattatatgtaaattcaacatgcccactatgcaatatttta aagaatattttggaatataataatattaaatttgaattaataaatatagaagatattgaa gaatctgatttaaatgaggaatttaaagtcttcaataatattggagatattaaaaatatt gcatttccatccattgtactgggcaatgagataattgtgggctacaacatagaaaaatta gaaaaactattaaataaaaaattgttaaagccacctgttaccgaaaaatcatttattgat tttgcaaaacataacaagcataataacgatagtaatgataaaaataatctgatagaatat tataataaagttgaattattcagtgaatatagtggctattatttaaatcctgataaagat tatgttttaaatgtgcttaatacccagttaaagaatcaagaaaacggaataaaatactgc ccctgcaagctcggaaatgtgccctgcccctgccccgatgtaaaacatgagattaaaaca tacgggcattgtttttgcggactttatgtatcgaatgaatatatagaaaattggaaaaaa atggatactccattaatgctttctaacagaaacattaaaaaatatgaagatattccaaat aatatcaatagccattttttaaaaattcatatattatttaacaaaggtatgattgaagga aattctaaatcttctgatttaattattgaggagctccttgataaaacatatagattaaaa aatagcggatatgggattaaaaatgatatattgattaaattcaaagaagatgaggcacat aaaaaccttcaattgtggtataatgattttaatgactattataacaataagaataaggta ggggacatttttagcgattatgaatttgatattgcaataaatgtgccaatagaagttcca gatggagaaaaaatagaatataacgggctaactataaccacaaactacaacactacacaa tatcgtagaagatatttaattgaaggaaataatttaaatattatcaacggaattgacaat ataattgtgttggaagttgttggacaaatgaatgaaataatgaatgatttatacttagaa tattcaaaaaaagtagaatcattacaacaagatataaatcagcttgtaatagatgtaaat attgataaagaacattcatttgaagaatatttgtcgatgctatcgataagtgttaaaaat atatctaaccttcaagagaaacttacaaaaaaatatagtctatttgaatattatatggcg aaaaaattagacgaccaaaaatttaaatcctttttagattcagcaggatatagtatggag tatagggagctcctcaatgatttcacacatttattgaatgagctcagtattatgaaaaat accgtaaatgaattaataggaatacttgaaacaaatgtaaatatctcccaaaataataaa accataaatttacaaaatgaaatacataaaacggcaaaaactcagttaaatatgcatagg gcggtggagggattatatacaatctttgcagcattttattttacagaactcgccttgata gtttttgaagggctaaatcatgcccatattgtaaaatatgatgcctatgttttagcatca tttttcgttccagttgccatgcttcttggagttgtagtaagtaaaaaattaaaaaataaa tacggttaa |
| A4YD28 | UniRef cluster | -------------------------------------------NAIREKLNGKVKVEVMVT-----PSCPYCP-YAALLAHMVAYEACKAEK---CNVISDVVESYENPDIAEKY----QVM----------SVPTVAVNES------VEFIGVPQEE-----NFLNTLLEKQM-------------------- | atggaggaagaatatgctgagttattcacagaggaagtgaagaatgcccttgtggacgct ttaagggacatgaagggggaagttgaggctcatgtatttatagactcgagtaatcccaat tgccagtattgtggtgttacggagaagtttatggaattcgtgagacaagcctcaccaaaa ggtcaagacggagattacttgcttaaggtaaaggtacacgatctagcgaaggataaggag tcatttaaaaaatatgaggtatctagggttcccacagtcgccttcctcgagggcaagata aggtggacaggtgcgccacttggggaggaaataagggcactagtggagacaatagtcaga ttatctcaaggcgaaagcggtttaagtcctgagactgtgaacgcaataagggagaaacta aacggaaaggtaaaggttgaggtaatggttactccctcttgtccatactgcccatatgcc gcccttctagctcacatggtagcatacgaagcttgcaaggcagaaaagtgcaacgtaata tctgatgtggttgagtcctacgagaatcccgacatagcagagaagtatcaggttatgtca gttccgacggttgccgtgaatgaatccgtggaattcataggagttccgcaagaggaaaac ttcttgaatactctcctagaaaagcaaatgtag |
| A4WL56 | UniRef cluster | ---------------------------------------RNELSNLAQSAPKRVYVMTVVT-----PSCPYCP-YAVLLANMFAYES----K---GKVVSVAVEAYENPDIADMY----GVT----------GVPTVILQAEDAAVGDVEFVGVPPEH-----ELLARVKNHMGLS------------------ | atggctgttcctataggagagcctggagaagtaccacacatcgaagtagacgaggagact aaggagataatcaaagagatgctctcacaaatggagaacccggtagagataaacttcttc acaagcgggagttgcggaggaagggagactaattggtgcgtccctacagaggagttgctg gatctactccaccagctggcgccagccggcaagctcgtgataaataagtacgacgcagat aaaaacgccgacgtttttaggaaattcggcgtagagccgcagagggttcccgtagtatat tttggcgacggctttattagatacctcggcgcaccaatgggggaggaggtcagggcattt atagagacagtggtgcgcctgagcaccgggaaaacagggctgaggcagaagacccgcaac gagctctcaaacctggcgcagagcgcccccaagagagtatatgtgatgacagtggtcacg ccgagttgcccctactgcccatacgcagtactcctcgcaaacatgttcgcatatgaaagc aagggaaaggtagtatctgtagccgtggaggcttacgagaatcccgacatagctgacatg tacggagtgacaggagtccctacggtgattctgcaagcagaggacgcggcggtgggcgat gtggaatttgtcggtgttccgccagagcacgagcttcttgcacgggttaaaaaccacatg ggcctatcttag |
| A3MWU7 | UniRef cluster | -------------MGEEVRAFIETVVRLSTGKTGLRQRTRGELSALAQNAPKRVYVLTVVT-----PSCPYCP-YAVLLANMFAYES----K---GKVVSVVVEAYENPDIADMY----GVT----------GVPTVILQAEDAAVGEVEFVGVPPEH-----ELLAKVKNHMGL------------------- | atgtctaccgttccaataggtccgcccgaggaggttccccacatagaggtagacgaggag accaaggagataattaaggagatgttgtctcaaatggagaacccggtagaagtgaacttt ttcacgagtaaggggtgcggaggcagagagaccaactggtgtgtacccaccgaggagctc ctagacctactagtgcaacttgcccccgccggcaaattgagagtcaacaaatatgcgtat gaacaaaacgccgatgtgttcaacaagtacggggttgagccgcacagagtgcctgttgta tacttcggcgatggcttcattaggtaccttggcgcgcctatgggcgaggaggtcagagcg ttcatagaaaccgtggtgcggttgagcactggcaagaccggccttaggcagaggaccaga ggcgagctttctgctctcgcgcaaaatgccccaaagcgcgtatacgtcttaacggtggtc actcccagctgcccctactgtccctacgccgtccttttggcaaacatgttcgcgtatgag agcaagggcaaggttgtctccgtggtggtggaggcctatgagaacccagacatagcagac atgtacggagtcaccggcgtccccacagttatcctacaagccgaagacgctgcggtggga gaggtggagtttgtgggagttccaccagagcacgagctgttggcaaaagtgaaaaatcac atgggtctttaa |
| A3DKZ8 | UniRef cluster | -------------------------------------------IEEAKKIDVPLHIKIFVT-----PECPYCP-LTVDAFNQLALIN--------DKILVETIEAIELPLEADMY----NVA----------YVPDVIITD----PDKMDEYGVEPVERINGYMPIEEAINIVKYAAEKLKEMKKQG------- | atgagcgctgatgaattatttgatgaggaaactaagcaggcgttaaaacaaatattccag caattccagcgaaaagttgttgattacttagtggtagagaagggttcagagagcaatcct gatcctaatgatcctgatgatgaacacgagcatacagcaactcatattcacccccatcct catgttcatcatcaccacggatgcccaacttgcggagaagcagagatcttagcaaaagcg cttatggagcttgctgatggtaaactagaatttaagatcatagataagaatagtgaggaa gcaaaacagcttcatacaagatatgttccagcattcatatatggatcgccaaagaagaat attagatattatggattgccaagtggacaagaatttgctccattcatatatgttcatcta tacattgctaataatgatataaaacttccagaaaacgttatagaggaagctaagaaaata gatgtaccgcttcatatcaaaatatttgttacaccagaatgcccctattgcccattaaca gtagatgctttcaatcaattagcacttataaacgataagatattagttgaaacaatagaa gcaattgaattacctctagaagcagatatgtataatgtagcatatgttccagacgtaata ataactgatccagacaaaatggatgagtacggagtagaacctgttgagaggataaatggt tatatgcctatagaagaagcaataaacattgtaaaatatgcagccgaaaaacttaaagaa atgaagaagcaaggctag |
| A1RS60 | UniRef cluster | YFGNGFIRYLGAPLGEEVRAFIETVIRLSTGKTGLRQKTRAELSTLAQSASKRVYILTVVT-----PSCPYCP-YAVLLANMFAYES----K---GKVVSVVVEAHENPDIADMY----GVT----------GVPAVILQAEDVSVGDVEFVGVPPEH-----ELLARVKNHMGLS------------------ | atggctacaccaataggcggacctgaagaggaagtgcctcatatagaagtagacgaagag actagggagatcattagggagatgttatcccaaatggagaaccctgtggagataaacttc tttacaagtcagaattgtgcaggccgtgagactaattggtgtatacctaccgaagagttt ctagatctactaatacaactggcgccgcaggagaaactacgtcttaataaatacttgtat gaaaaagacgcagagagttttaaaaaatttggcgtggaacctcagcgtgtgccagtggta tactttggcaacggctttattaggtatcttggcgcacctcttggagaagaggtcagagcg tttatagagactgttattagacttagtacgggtaagacagggcttaggcaaaaaactagg gctgagctttctacacttgcacagagtgcttcaaaacgtgtctacatcttaactgtagtt acgccaagctgcccatattgtccatatgctgtcctcttggcaaatatgtttgcatatgaa agcaaagggaaagttgtgtctgttgtagtagaggcacacgaaaacccagatatagcagat atgtacggcgtgacaggagttccagctgtaattttacaagccgaagacgtttcagttgga gatgtagaatttgtaggcgtacccccagagcacgagcttttggcgcgagttaaaaaccac atgggtctctcttaa |
| A1RX05 | UniRef cluster | -------------------------------------------EILLKYVTKPTRIMIFVT-----PTCPYCP-IAVRAAHRFAMVN----K---N-IYGDMIEALEFSDLADRY----GVY----------AVPKNVIQVN--GEDKNEFEGAAPDP-----YFVAKILEAYGVEIPRSLQEAIAGI------ | atgcctgttgaatacgatgaggaactcgtaaacgacttgaggcagatgtttcaggctctc gagaacccagtgagcatcaagtggttcgtggacccgggctccgagtgtgtttactgcgat gacacagagcagatactaaaccttgtgcaacaggtatccggtgggaaagtccgcgtcact aggtatacgagtaaagaccctgaggcaaagaagtacagcatagacatgtttccggctata ctcatccatggtgtggacgagtggaatgtgaggttcttcggtataccggctggctacgag ttcggcgcgtttgttgaagacatcatcgatgcgtctacgggtagggttaacatttctccg gagatcagggagatactcttgaagtacgtgactaagcctacccggataatgatattcgtg acacccacttgcccctattgcccgatagctgtccgtgccgctcacaggtttgccatggtg aacaagaacatctacggagacatgatagaggctctagagttctcggatttagcggacagg tatggcgtgtacgctgtcccgaagaacgttatacaggtaaacggcgaggataagaacgag ttcgaaggggcggctcccgacccctacttcgtggcgaaaatactcgaagcctacggagtc gagatacccagaagtcttcaggaagcgatagccgggatagaagcccagtatactgaggag acttacgaagacgaggaactacaccatcaccaccatcaccatcattaa |
| A2STK9 | UniRef cluster | ---------------------------------------------------MLHEVVVYSLSG-----CPHCK-ALKTFLDNQNI---TYTN---IDVGE---DEKAAAEMIKIS----GQR----------GVPVTVIDGEK-------IVIGDDLKK--VMEYLDAPVAVKKTPDVSADH------------ | atgttacatgaagtggttgtgtactcgctttcaggttgtcctcactgtaaagcattgaaa acattccttgataaccagaatataacgtacaccaatatcgatgtaggcgaagatgaaaaa gccgctgctgaaatgataaagatctccggccagaggggggttccggtcacggtcatcgac ggcgaaaaaatcgttattggcgatgatttgaaaaaagtcatggagtatcttgacgctccg gttgcggtaaaaaagactccggatgtttctgcagatcatgatctcgtagtgatcggtgcc ggagccggcggtctgtctgcagccatgtacggcgcccgaaaaggcatcgatatggtcgtg atcaccggtgctattggaggaatggtgaatcagagttatgttgttgaaaactatcccggc atccctgatatatccggtgctgatctgatgaaaaaaatctatgatcatacggtaaattca ggtgggattttcgttgaggatgttgtgaccggcatttccaaagccggggatgttttttcc attgacacgcttaacggagcccattatactgcaaaagccgtcatagcggccactggaagg tctccacgtctctccggagcgaaaggcgaaacagactatctcggaaaaggcgttgcgatc tgtacgacctgtgacgggccgttgtacaaaaataaagtggtcgggatccttggggggggt aacacggcagttgatatggcgatcgaactgagcgatattgcatcaaaaatccatctgatc gtccgcagtaagctgaaagccgacaaggttctgatcgaccggctcaaaacgaagaagaac attgttctgcacaaaggatatactatcgaagaatttggcggcggtcagtttctggaatat gtgattttgaaaaaacagggcgggatcaccagcctccttggaaaaggtgaagaaaagatc cctgtcgacggcgtcttccttgggatcggtcttgatccaaacacctccatctttgagggt cttggagtaacaatgaatccaaataaagagatcatcgtcgatatcgactgcaatacgaat gttcccggattctatgcggccggagatgcgacctccatcaaagccaagcagatcgcatca tccgttggtgaaggagtaaaggctcttctttcagcatatgattacctgaaaaggtaa |
| A3CRV4 | UniRef cluster | -------------------------------------------NTVPEIFPFMAGVKVYTTEN-----CPYCR-MVKAFLRKHDI---EHEI---VDVGK---DREAAREMIEIS----GQR----------GVPVTVS-GDE-------VVVGFDAKR--LRELFGTALEETVYDAVIVG------------- | atgttaaatactgtccccgagatattccccttcatggcaggcgttaaggtctacacgacg gagaactgcccctactgccggatggtcaaggcgttcctccggaagcacgatatcgaacac gagatcgtcgacgtcgggaaagaccgcgaggcggcccgggagatgatcgagatctcgggg caacggggtgtgccggtcacggtatccggcgacgaggtggtcgtcgggttcgatgcaaag aggctccgggagctcttcgggacggcgctggaggagaccgtctacgacgcggtcatcgtg ggcgctggtccggcagggctgacggcggcggtctactgcgcccggaaactcatgaagacc gtcgtcatagcggagaacatcggtgggcaggcggcctggagctgggctatcgagaactac atggggttctcgacgatatcgggcgaggaactggtccggaagttcgaggagcaggtccgg gggttcgacgtccgtctcgagctcgagagcgtcggggacgttcgcaaagaggacgatacg atcctcgtccggacggcctcggggaccgtctaccgttcccggacgctcatcctcgctccc gggaaggagccccggcggctggggcttcccggtgaggaccggctgatgggaaagggtatc tcggtctgcgctatctgcgacgccccgctctaccgcgataaaccggttgccgtcgtcggc ggcggaaacgccgccctccagaccgcgatcgagatgacgaagttcgcgagttcggtgacc ctgatcgcccggagggatctccggtgcgacgaggtctacgtcgaccgggcaaagaaggcg ggggtgcggactttctcccaccacgaggtgacggcgctccacggggacgcgggcctgacc ggggtcacggtccgcgaccgcgagaccggggaggagacgggtctcgatgtggaggggctc ttcctcgcgatcgggcttgcgccgaatacgggcttcctcaaggatctcgtggtgctgaac gagcagggcgagatcgatatcgacgaaaatggccatacgagcgttcccggggtcttcgcg gccggggacgcgacctgcgtcaaggccaagcagatcatcgtcgccgccggcgacggggcg aaggcggcgctcgaggcgcacgagtatttcgaaggcgagccggaagaagagcagatcgtc tgcacgtga |
| Q3BPF6 | UniRef cluster | -------------------------------------------RSRIDTLLQSSRVVLFMKGQPGMPQCGFSA-KAVGVLDGLGI---DYAH---VNVLA---DQEIREGIKAYG----DWP----------TIPQLYVDGE--------LIGGSDI----IVQMADSGELSSMLGLQAPDR------------ | atgtctctcgatcccgccctgcgttcccgtatcgatacgctgctgcaatccagccgcgtg gtgctcttcatgaaaggccagcccggcatgccgcagtgcggtttttcggccaaggcggtc ggcgtgctggacggtctgggcatcgactatgcccacgtcaacgtgctggccgaccaggaa atccgcgagggcatcaaggcctatggcgactggccgaccatcccgcagctgtacgtggac ggcgagctgatcggcggcagcgacatcatcgtgcagatggccgacagcggagagctgagc agcatgctcggcctgcaggcgcccgaccgcagcccgcccaagatcaccatcacccccgcc gcggtcgagatgctcaaaagtgcgctggccgatgcgccggatgcctcgctgacgctggcc atcgatgccaatttccagcccaatttccagctggcgccgaccaacccgaacgccattgcg gctgaatccaacgggctgcgcgtgcagttcgacctggccagcgcgcgtcgtgccgacggc atcaccatcgactgggtcgacgacatccgcggccgtggcctggccatcgataaccccaat gcgcccaagccggtgcaggagctgtcggtgcgcgatgccgacgaccgtctcaaggccggc agcctgaccctggtggacgtgcgcccggccgacgagcgcgcactggccacggtggccgca ccgttccgcacgctggatgcgcacgagcgcgccgccatcgaacagttgcccaaggacacc ccgctggcgttcctgtgccaccgcggcggccgcagccttcaggccgccgaacatttccgt gggctgggcttcaccaatgtctacaacgtcaccggcggcatcgatgcgtggtcggacgag gtggacaacggcgtggcgaaatactga |
| Q5H3X7 | UniRef cluster | -------------------------------------------RSRIDTLLQSSRVVLFMKGQPGMPQCGFSA-KAVGVLDGLGI---DYAH---VNVLA---DQEIREGIKVYG----DWP----------TIPQLYVDGE--------LIGGSDI----ILQMADSGELSSMLGLQAPDR------------ | atgtccctcgatcccgccctgcgttcccgtatcgatacgctgctgcaatccagccgcgtg gtgctcttcatgaaaggccagcccggcatgccgcagtgcggtttttcggccaaggcggtc ggcgtgctggacggtctgggcatcgactatgcccatgtcaacgtactggccgaccaggaa atccgcgagggcatcaaggtctatggcgattggccgaccattccgcagctgtatgtggac ggcgagctgatcggcggtagcgacatcatcctgcagatggccgacagcggcgagctgagc agcatgctcggcctgcaagcacccgaccgcaccccgcccaagatcaccattacccccgct gcggtcgagatgctcaagggtgcgctggccgacgcgccggatgcctccttgacgctgtcc atcgatgccaacttccagcccaacttccagctggcaccgaccaatccgaacgccattgcc gctgaatccaacggcctgcgcgtgcagttcgacctggccagcgcgcgccgcgccgacggc atcaccatcgattgggtggacgacatccgcggtcgtggcctggccatcgacaaccccaac gcgcccaagccggtgcaggagctgtcggtgcgcgatgccgacgaccgcctcaaggccggc agcctgaccctggtggacgtgcgcccggccgatgagcgtgcgctggcctccgtggccgcg ccgttccgcaccctggatgcgcacgagcgcgctgcgatcgaacagctgcccaaggacacc gcgctggcgttcctgtgccaccgcggcggacgcagcctgcaggccgccgaacatttccgc agcctgggctttaccaacgtctacaacgtcaccggcggcatcgatgcgtggtcggacgac gtggacaatggcgtggcgaaatactga |
| Q2P6T4 | UniRef cluster | -------------------------------------------RSRIDTLLQSSRVVLFMKGQPGMPQCGFSA-KAVGVLDGLGI---DYAH---VNVLA---DQEIREGIKVYG----DWP----------TIPQLYVDGE--------LIGGSDI----ILQMADSGELSSMLGLQAPDR------------ | atgtccctcgatcccgccctgcgttcccgtatcgatacgctgctgcaatccagccgcgtg gtgctcttcatgaaaggccagcccggcatgccgcagtgcggtttttcggccaaggcggtc ggcgtgctggacggtctgggcatcgactatgcccatgtcaacgtactggccgaccaggaa atccgcgagggcatcaaggtctatggcgattggccgaccattccgcagctgtatgtggac ggcgagctgatcggcggtagcgacatcatcctgcagatggccgacagcggcgagctgagc agcatgctcggcctgcaagcacccgaccgcaccccgcccaagatcaccattacccccgct gcggtcgagatgctcaagggtgcgctggccgacgcgccggatgcctccttgacgctgtcc atcgatgccaacttccagcccaacttccagctggcaccgaccaatccgaacgccattgcc gctgaatccaacggcctgcgcgtgcagttcgacctggccagcgcgcgccgcgccgacggc atcaccatcgattgggtggacgacatccgcggtcgtggcctggccatcgacaaccccaac gcgcccaagccggtgcaggagctgtcggtgcgcgatgccgacgaccgcctcaaggccggc agcctgaccctggtggacgtgcgcccggccgatgagcgtgcgctggcctccgtggccgcg ccgttccgcaccctggatgcgcacgagcgcgctgcgatcgaacagctgcccaaggacacc gcgctggcgttcctgtgccaccgcggcggacgcagcctgcaggccgccgaacatttccgc agcctgggctttaccaacgtctacaacgtcaccggcggcatcgatgcgtggtcggacgac gtggacaatggcgtggcgaaatactga |
| Q5H3X7 | UniRef cluster | -------------------------------------------RSRIDTLLQSSRVVLFMKGQPGMPQCGFSA-KAVGVLDGLGI---DYAH---VNVLA---DQEIREGIKVYG----DWP----------TIPQLYVDGE--------LIGGSDI----ILQMADSGELSSMLGLQAPDR------------ | atgtccctcgatcccgccctgcgttcccgtatcgatacgctgctgcaatccagccgcgtg gtgctcttcatgaaaggccagcccggcatgccgcagtgcggtttttcggccaaggcggtc ggcgtgctggacggtctgggcatcgactatgcccatgtcaacgtactggccgaccaggaa atccgcgagggcatcaaggtctatggcgattggccgaccattccgcagctgtatgtggac ggcgagctgatcggcggtagcgacatcatcctgcagatggccgacagcggcgagctgagc agcatgctcggcctgcaagcacccgaccgcaccccgcccaagatcaccattacccccgct gcggtcgagatgctcaagggtgcgctggccgacgcgccggatgcctccttgacgctgtcc atcgatgccaacttccagcccaacttccagctggcaccgaccaatccgaacgccattgcc gctgaatccaacggcctgcgcgtgcagttcgacctggccagcgcgcgccgcgccgacggc atcaccatcgattgggtggacgacatccgcggtcgtggcctggccatcgacaaccccaac gcgcccaagccggtgcaggagctgtcggtgcgcgatgccgacgaccgcctcaaggccggc agcctgaccctggtggacgtgcgcccggccgatgagcgtgcgctggcctccgtggccgcg ccgttccgcaccctggatgcgcacgagcgcgctgcgatcgaacagctgcccaaggacacc gcgctggcgttcctgtgccaccgcggcggacgcagcctgcaggccgccgaacatttccgc agcctgggctttaccaacgtctacaacgtcaccggcggcatcgatgcgtggtcggacgac gtggacaatggcgtggcgaaatactga |
| Q2P6T4 | UniRef cluster | -------------------------------------------RSRIDTLLQSSRVVLFMKGQPGMPQCGFSA-KAVGVLDGLGI---DYAH---VNVLA---DQEIREGIKVYG----DWP----------TIPQLYVDGE--------LIGGSDI----ILQMADSGELSSMLGLQAPDR------------ | atgtccctcgatcccgccctgcgttcccgtatcgatacgctgctgcaatccagccgcgtg gtgctcttcatgaaaggccagcccggcatgccgcagtgcggtttttcggccaaggcggtc ggcgtgctggacggtctgggcatcgactatgcccatgtcaacgtactggccgaccaggaa atccgcgagggcatcaaggtctatggcgattggccgaccattccgcagctgtatgtggac ggcgagctgatcggcggtagcgacatcatcctgcagatggccgacagcggcgagctgagc agcatgctcggcctgcaagcacccgaccgcaccccgcccaagatcaccattacccccgct gcggtcgagatgctcaagggtgcgctggccgacgcgccggatgcctccttgacgctgtcc atcgatgccaacttccagcccaacttccagctggcaccgaccaatccgaacgccattgcc gctgaatccaacggcctgcgcgtgcagttcgacctggccagcgcgcgccgcgccgacggc atcaccatcgattgggtggacgacatccgcggtcgtggcctggccatcgacaaccccaac gcgcccaagccggtgcaggagctgtcggtgcgcgatgccgacgaccgcctcaaggccggc agcctgaccctggtggacgtgcgcccggccgatgagcgtgcgctggcctccgtggccgcg ccgttccgcaccctggatgcgcacgagcgcgctgcgatcgaacagctgcccaaggacacc gcgctggcgttcctgtgccaccgcggcggacgcagcctgcaggccgccgaacatttccgc agcctgggctttaccaacgtctacaacgtcaccggcggcatcgatgcgtggtcggacgac gtggacaatggcgtggcgaaatactga |
| B0RNY7 | UniRef cluster | -------------------------------------------RSRIDTLLQSNRVVLFMKGQPGMPQCGFSA-KAIGVLDGLGI---DYAH---VNVLA---DQEIREGIKAYG----DWP----------TIPQLYVDGE--------LIGGSDI----IVQMADSGELSSMLGLQAPDR------------ | atgtccctcgatcccgccctgcgttcccgcatcgatacgctgctgcaatccaaccgcgtg gtcctcttcatgaaaggccagcccggcatgccgcagtgcggcttctcggccaaggccatc ggcgtgctggacggcctgggcatcgactatgcccacgtcaacgtgctggccgaccaggaa atccgcgaaggcatcaaggcctacggcgactggccgaccattccgcagctgtatgtggat ggcgagctgatcggcggcagcgacatcatcgtgcagatggccgacagcggcgagctgagc agcatgctcggcctgcaggcaccggaccgcagcccgcccaagatcaccatcaccccggct gcggtcgagatgctcaagggtgcgttggccgatgcaccggatgcctcgctgacgctggcc atcgatgccaatttccagccgaacttccagctggcgccaaccaacccgaacgctatcgcc gccgagtccaacgggctgcgcgtgcagttcgacctggccagcgcgcgccgcgccgacggc atcaccatcgactgggtggacgacatccgtggccgcggcctggccatcgacaaccccaac gcgcccaagccggtgcaggagctggcggtgcgcgatgccgacgaccgtctcaaggccggt acgctgactgtggtggacgtgcgcccggccgatgagcgtgcgctggctacggtggcagcg ccgttccgcaccctggacgcacacgagcgcctggagatcgagcaactgcccaaggacacc ccgctggcgttcctgtgccaccgcggcgggcgtagcctgcaggcggccgagcatttccgc ggcctgggctttaccaacgtctacaacgtcaccggcggcatcgatgcctggtcggacgcg gtggacaacggcgtgcccaagtactga |
| A1G226 | UniRef cluster | -------------------------------------------RSRIETILNDNRVVLFMKGQPSMPQCGFSA-KAVGALQDLGV---EFAH---VNVLA---DQEIREGIKAYG----DWP----------TIPQLYIDGE--------LVGGSDI----VLQMAASGELSSVLGLAAPDR------------ | atgttcctgtgttgctcatcccccatgatgccatgggggttagaattagcgattcgaatt cccggaaccccgacgtccatgtccctcgatcccgccctgcgttcgcgcatcgaaaccatc ctcaacgacaaccgcgtcgtgctgttcatgaagggccagccgtcgatgccgcagtgtggt ttctcggccaaggccgtgggcgccctgcaggacctcggcgtcgagttcgcccatgtcaac gtgctggccgaccaggaaatccgtgagggcatcaaggcctatggcgactggccgacgatc ccgcagctgtacatcgacggcgaactggtcggcggcagcgacatcgtgctgcagatggcc gccagtggcgagctgagcagcgtgctcggcctggccgcaccggaccgcaccccgccgagc atcaccgtcaccccggccgccgtggaaatgctcaagggcgcgttggccgacgccccgggc gccgccctgcagctgagcatcgacgccggcttccagccgaacttccagctggccccgcac gacgaaggcgcgatcgccgccgagtccaacggcctgcgcgtgcagttcgacctggccagc gcccgtcgcgccaatggcatcaccatcgactgggtggacgacatccgcggcaagggcctg gcgatcgacaacccgaacgcgcccaagccggtacaggaaatcagcgtgcgtgatgccgac gacctggtgcgtgccggcaacatcaccctggtggacgtgcgcccggccgacgagcgcgcc atcgccgcggtcggcgtgccgttcaagagcttcgacggcaacggccgcgccgagctggaa gccctgcccaaggacaccgcgctggccttcatgtgccaccacggtggccgcagtgcgcag gccgccgagcagttccgcgccctgggcttcaccaaggtgttcaacgtcaccggcggcatc aacgcctggtcggaagacgtggacaacggcgtgccgaagtactga |
| Q87BN9 | UniRef cluster | -------------------------------------------RSRIETLLHSNRVVLFMKGRPGMPQCGFSA-KAAGILQALGV---EYAH---VNVLD---DQEIREGIKRYG----DWP----------TIPQLYIDGE--------LIGGSDI----VSQMYENGELSTLLGVAAPDL------------ | atgtctcttgattctgcattgcgttcgcgcatcgaaacgttgttgcattccaaccgtgtt gttttgtttatgaaggggcgtccggggatgccgcagtgcggtttttctgctaaggcggca ggcattctccaggcacttggtgttgagtacgcgcatgtcaatgtattggacgaccaagag atacgagaggggatcaaacgctatggcgattggccaacaattccgcagttgtacattgat ggcgagttgatcggtggcagtgacatcgtgtcgcagatgtatgagaacggggagttgagc actttgcttggtgtggctgctccggaccttacgccgccatccatcacgatcactccaact gctgtggagatgctcaagggtacattagccaatgcacccgggagtacgttggcactgtca atcgatagtcgtttccaacctacttttgagctggcgccaattaatacgcaggcaattgct gcagaatataacggtttacgggtgcagtttgatcttgccagtgcgcgtcgtgctgagggc atcacgattgattgggttgatgatatccgtggccaggggttagtgattgacaatccaaac gcgcctcagccgattcaggagttatcgcccggtgatgcggcggctcaggttgatgcgggt gttctgacgttggtggatgtgcgccctgctgatgagcgtgcggttgcttcggtcgctgtg ccgttccgcaccatggacggcggcgaacgtgctgttctggagcagcttcccaaagggacg ccgctggcattcttgtgtcatcacggcgggcgtagtctccaggccgccgagcatttccgt agtctggggtttacctctatctacaatatcggtggtggcattgatgcttggtccacccag gttgatcctggcgtgccgaagtattga |
| Q3RDN3 | UniRef cluster | -------------------------------------------RSRIETLLHSNRVVLFMKGRPGMPQCGFSA-KAAGILQALGV---EYAH---VNVLD---DQEIREGIKRYG----DWP----------TIPQLYIDGE--------LIGGSDI----VSQMYENGELSTLLGVAAPDL------------ | atgtctcttgattctgcattgcgttcgcgcatcgaaacgttgttgcattccaaccgtgtt gttttgtttatgaaggggcgtccggggatgccgcagtgcggtttttctgctaaggcggca ggcattctccaggcacttggtgtcgagtacgcgcatgtcaatgtattggacgaccaagag attcgagaggggatcaaacgctatggcgattggccaacaattccgcagttgtacattgat ggcgagttgatcggtggcagtgacatcgtgtcgcagatgtatgagaacggggagttgagc actttgcttggtgtggctgctccggatcttacgccgccatccatcacgatcactccaact gctgtggagatgctcaagggtacattagccaatgcacccgggagtacgttggcgctgtca atcgatagtcgtttccaacctacttttgagctggcgccaattaatacgcaggcaattgct gcagaatctaacggtttacgggtgcagtttgatcttgccagtgcgcgtcgtgctgagggc atcacgattgattgggttgatgatatccgtggccaggggttagtgattgacaatccaaac gtgcctcagccgattcaggagttatcgcccggtgatgcggcggctcaggttgatgcgggt gttctgacgttggtggatgtgcgccctgctgatgagcgtgcggttgcttcggtcgctgtg ccgttccgcaccatggacggcggcgaacgtgctgttctggagcagcttcccaaagggacg ccgctggcattcttgtgtcatcacggcgggcgtagtctccaggccgccgagcatttccgt agtctggggtttacctctatctacaatatcggtggtggcattgatgcttggtccacccag gttgatcctggcgtgccgaagtattga |
| Q3R9I2 | UniRef cluster | -------------------------------------------RSRIETLLHSNRVVLFMKGRPGMPQCGFSA-KAAGILQALGV---EYAH---VNVLD---DQEIREGIKRYG----DWP----------TIPQLYIDGE--------LIGGSDI----VSQMYENGELSTLLGVAAPDL------------ | atgtctcttgattctgcattgcgttcgcgcatcgaaacgttgttgcattccaaccgtgtt gttttgtttatgaaggggcgtccggggatgccgcagtgcggtttttctgctaaggcggca ggcattctccaggcacttggtgttgagtacgcgcatgtcaatgtattggacgaccaagag atacgagaggggatcaaacgctatggcgattggccaacaattccgcagttgtacattgat ggcgagttgatcggtggcagtgacatcgtgtcgcagatgtatgagaacggggagttgagc actttgcttggtgtggctgctccggaccttacgccgccatccatcacgatcactccaact gctgtggagatgctcaagggtacattagccaatgcacccgggagtacgttggcactgtca atcgatagtcgtttccaacctacttttgagctggcgccaattaatacgcaggcaattgct gcagaatctaacggtttacgggtgcagtttgatcttgccagtgcgcgtcgtgctgagggc atcacgattgattgggttgatgatatccgtggccaggggttagtgattgacaatccaaac gcgcctcagccgattcaggagttatcgcccggtgatgcggcggctcaggttgatgcgggt gttctgacgttggtggatgtgcgccctgctgatgagcgtgcggttgcttcggtcgctgtg ccgttccgcaccatggacggcggcgaacgtgctgttctggagcagcttcccaaagggacg ccgctggcatttttgtgtcatcacggcgggcgtagtctccaggccgccgagcatttccgt agtctggggtttacctctatctacaatatcggtggtggcattgatgcttggtccacccag gttgatcctggcgtgccgaagtattga |
| Q3R4U9 | UniRef cluster | -------------------------------------------RSRIETLLHSNRVVLFMKGRPGMPQCGFSA-KAAGILQALGV---EYAH---VNVLD---DQEIREGIKRYG----DWP----------TIPQLYIDGE--------LIGGSDI----VSQMYENGELSTLLGVAAPDL------------ | atgtctcttgattctgcattgcgttcgcgcatcgaaacgttgttgcattccaaccgtgtt gttttgtttatgaaggggcgtccggggatgccgcagtgcggtttttctgctaaggcggca ggcattctccaggcacttggtgtcgagtacgcgcatgtcaatgtattggacgaccaagag attcgagaggggatcaaacgctatggcgattggccaacaattccgcagttgtacattgat ggcgagttgatcggtggcagtgacatcgtgtcgcagatgtatgagaacggggagttgagc actttgcttggtgtggctgctccggatcttacgccgccatccatcacgatcactccaact gctgtggagatgctcaagggtacattagccaatgcacccgggagtacgttggcgctgtca atcgatagtcgtttccaacctacttttgagctggcgccaattaatacgcaggcaattgct gcagaatctaacggtttacgggtgcagtttgatcttgccagtgcgcgtcgtgctgagggc atcacgattgattgggttgatgatatccgtggccaggggttagtgattgacaatccaaac gtgcctcagccgattcaggagttatcgcccggtgatgcggcggctcaggttgatgcgggt gttctgacgttggtggatgtgcgccctgctgatgagcgtgcggttgcttcggtcgctgtg ccgttccgcaccatggacggcggcgaacgtgctgttctggagcagcttcccaaagggacg ccgctggcattcttgtgtcatcacggcgggcgtagtctccaggccgccgagcatttccgt agtctggggtttacctctatctacaatatcggtggtggcattgatgcttggtccacccag gttgatcctggcgtgccgaagtattga |
| B0U3N8 | UniRef cluster | -------------------------------------------RSRIETLLHSNRVVLFMKGRPGMPQCGFSA-KAAGILQALGV---EYAH---VNVLD---DQEIREGIKRYG----DWP----------TIPQLYIDGE--------LIGGSDI----VSQMYENGELSTLLGVAAPDL------------ | atgtctcttgattctgcattgcgttcgcgcatcgaaacgttgttgcattccaaccgtgtt gttttgtttatgaaggggcgtccggggatgccgcagtgcggtttttctgctaaggcggca ggcattctccaggcacttggtgtcgagtacgcgcatgtcaatgtattggacgaccaagag attcgagaggggatcaaacgctatggcgattggccaacaattccgcagttgtacattgat ggcgagttgatcggtggcagtgacatcgtgtcgcagatgtatgagaacggggagttgagc actttgcttggtgtggctgctccggatcttacgccgccatccatcacgatcactccaact gctgtggagatgctcaagggtacattagccaatgcacccgggagtacgttggcgctgtca atcgatagtcgtttccaacctacttttgagctggcgccaattaatacgcaggcaattgct gcagaatctaacggtttacgggtgcagtttgatcttgccagtgcgcgtcgtgctgagggc atcacgattgattgggttgatgatatccgtggccaggggttagtgattgacaatccaaac gtgcctcagccgattcaggagttatcgcccggtgatgcggcggctcaggttgatgcgggt gttctgacgttggtggatgtgcgccctgctgatgagcgtgcggttgcttcggtcgctgtg ccgttccgcaccatggacggcggcgaacgtgctgttctggagcagcttcccaaagggacg ccgctggcattcttgtgtcatcacggcgggcgtagtctccaggccgccgagcatttccgt agtctggggtttacctctatctacaatatcggtggtggcattgatgcttggtccacccag gttgatcctggcgtgccgaagtattga |
| Q87BN9 | UniRef cluster | -------------------------------------------RSRIETLLHSNRVVLFMKGRPGMPQCGFSA-KAAGILQALGV---EYAH---VNVLD---DQEIREGIKRYG----DWP----------TIPQLYIDGE--------LIGGSDI----VSQMYENGELSTLLGVAAPDL------------ | atgtctcttgattctgcattgcgttcgcgcatcgaaacgttgttgcattccaaccgtgtt gttttgtttatgaaggggcgtccggggatgccgcagtgcggtttttctgctaaggcggca ggcattctccaggcacttggtgttgagtacgcgcatgtcaatgtattggacgaccaagag atacgagaggggatcaaacgctatggcgattggccaacaattccgcagttgtacattgat ggcgagttgatcggtggcagtgacatcgtgtcgcagatgtatgagaacggggagttgagc actttgcttggtgtggctgctccggaccttacgccgccatccatcacgatcactccaact gctgtggagatgctcaagggtacattagccaatgcacccgggagtacgttggcactgtca atcgatagtcgtttccaacctacttttgagctggcgccaattaatacgcaggcaattgct gcagaatataacggtttacgggtgcagtttgatcttgccagtgcgcgtcgtgctgagggc atcacgattgattgggttgatgatatccgtggccaggggttagtgattgacaatccaaac gcgcctcagccgattcaggagttatcgcccggtgatgcggcggctcaggttgatgcgggt gttctgacgttggtggatgtgcgccctgctgatgagcgtgcggttgcttcggtcgctgtg ccgttccgcaccatggacggcggcgaacgtgctgttctggagcagcttcccaaagggacg ccgctggcattcttgtgtcatcacggcgggcgtagtctccaggccgccgagcatttccgt agtctggggtttacctctatctacaatatcggtggtggcattgatgcttggtccacccag gttgatcctggcgtgccgaagtattga |
| Q3RDN3 | UniRef cluster | -------------------------------------------RSRIETLLHSNRVVLFMKGRPGMPQCGFSA-KAAGILQALGV---EYAH---VNVLD---DQEIREGIKRYG----DWP----------TIPQLYIDGE--------LIGGSDI----VSQMYENGELSTLLGVAAPDL------------ | atgtctcttgattctgcattgcgttcgcgcatcgaaacgttgttgcattccaaccgtgtt gttttgtttatgaaggggcgtccggggatgccgcagtgcggtttttctgctaaggcggca ggcattctccaggcacttggtgtcgagtacgcgcatgtcaatgtattggacgaccaagag attcgagaggggatcaaacgctatggcgattggccaacaattccgcagttgtacattgat ggcgagttgatcggtggcagtgacatcgtgtcgcagatgtatgagaacggggagttgagc actttgcttggtgtggctgctccggatcttacgccgccatccatcacgatcactccaact gctgtggagatgctcaagggtacattagccaatgcacccgggagtacgttggcgctgtca atcgatagtcgtttccaacctacttttgagctggcgccaattaatacgcaggcaattgct gcagaatctaacggtttacgggtgcagtttgatcttgccagtgcgcgtcgtgctgagggc atcacgattgattgggttgatgatatccgtggccaggggttagtgattgacaatccaaac gtgcctcagccgattcaggagttatcgcccggtgatgcggcggctcaggttgatgcgggt gttctgacgttggtggatgtgcgccctgctgatgagcgtgcggttgcttcggtcgctgtg ccgttccgcaccatggacggcggcgaacgtgctgttctggagcagcttcccaaagggacg ccgctggcattcttgtgtcatcacggcgggcgtagtctccaggccgccgagcatttccgt agtctggggtttacctctatctacaatatcggtggtggcattgatgcttggtccacccag gttgatcctggcgtgccgaagtattga |
| Q3R9I2 | UniRef cluster | -------------------------------------------RSRIETLLHSNRVVLFMKGRPGMPQCGFSA-KAAGILQALGV---EYAH---VNVLD---DQEIREGIKRYG----DWP----------TIPQLYIDGE--------LIGGSDI----VSQMYENGELSTLLGVAAPDL------------ | atgtctcttgattctgcattgcgttcgcgcatcgaaacgttgttgcattccaaccgtgtt gttttgtttatgaaggggcgtccggggatgccgcagtgcggtttttctgctaaggcggca ggcattctccaggcacttggtgttgagtacgcgcatgtcaatgtattggacgaccaagag atacgagaggggatcaaacgctatggcgattggccaacaattccgcagttgtacattgat ggcgagttgatcggtggcagtgacatcgtgtcgcagatgtatgagaacggggagttgagc actttgcttggtgtggctgctccggaccttacgccgccatccatcacgatcactccaact gctgtggagatgctcaagggtacattagccaatgcacccgggagtacgttggcactgtca atcgatagtcgtttccaacctacttttgagctggcgccaattaatacgcaggcaattgct gcagaatctaacggtttacgggtgcagtttgatcttgccagtgcgcgtcgtgctgagggc atcacgattgattgggttgatgatatccgtggccaggggttagtgattgacaatccaaac gcgcctcagccgattcaggagttatcgcccggtgatgcggcggctcaggttgatgcgggt gttctgacgttggtggatgtgcgccctgctgatgagcgtgcggttgcttcggtcgctgtg ccgttccgcaccatggacggcggcgaacgtgctgttctggagcagcttcccaaagggacg ccgctggcatttttgtgtcatcacggcgggcgtagtctccaggccgccgagcatttccgt agtctggggtttacctctatctacaatatcggtggtggcattgatgcttggtccacccag gttgatcctggcgtgccgaagtattga |
| Q3R4U9 | UniRef cluster | -------------------------------------------RSRIETLLHSNRVVLFMKGRPGMPQCGFSA-KAAGILQALGV---EYAH---VNVLD---DQEIREGIKRYG----DWP----------TIPQLYIDGE--------LIGGSDI----VSQMYENGELSTLLGVAAPDL------------ | atgtctcttgattctgcattgcgttcgcgcatcgaaacgttgttgcattccaaccgtgtt gttttgtttatgaaggggcgtccggggatgccgcagtgcggtttttctgctaaggcggca ggcattctccaggcacttggtgtcgagtacgcgcatgtcaatgtattggacgaccaagag attcgagaggggatcaaacgctatggcgattggccaacaattccgcagttgtacattgat ggcgagttgatcggtggcagtgacatcgtgtcgcagatgtatgagaacggggagttgagc actttgcttggtgtggctgctccggatcttacgccgccatccatcacgatcactccaact gctgtggagatgctcaagggtacattagccaatgcacccgggagtacgttggcgctgtca atcgatagtcgtttccaacctacttttgagctggcgccaattaatacgcaggcaattgct gcagaatctaacggtttacgggtgcagtttgatcttgccagtgcgcgtcgtgctgagggc atcacgattgattgggttgatgatatccgtggccaggggttagtgattgacaatccaaac gtgcctcagccgattcaggagttatcgcccggtgatgcggcggctcaggttgatgcgggt gttctgacgttggtggatgtgcgccctgctgatgagcgtgcggttgcttcggtcgctgtg ccgttccgcaccatggacggcggcgaacgtgctgttctggagcagcttcccaaagggacg ccgctggcattcttgtgtcatcacggcgggcgtagtctccaggccgccgagcatttccgt agtctggggtttacctctatctacaatatcggtggtggcattgatgcttggtccacccag gttgatcctggcgtgccgaagtattga |
| B0U3N8 | UniRef cluster | -------------------------------------------RSRIETLLHSNRVVLFMKGRPGMPQCGFSA-KAAGILQALGV---EYAH---VNVLD---DQEIREGIKRYG----DWP----------TIPQLYIDGE--------LIGGSDI----VSQMYENGELSTLLGVAAPDL------------ | atgtctcttgattctgcattgcgttcgcgcatcgaaacgttgttgcattccaaccgtgtt gttttgtttatgaaggggcgtccggggatgccgcagtgcggtttttctgctaaggcggca ggcattctccaggcacttggtgtcgagtacgcgcatgtcaatgtattggacgaccaagag attcgagaggggatcaaacgctatggcgattggccaacaattccgcagttgtacattgat ggcgagttgatcggtggcagtgacatcgtgtcgcagatgtatgagaacggggagttgagc actttgcttggtgtggctgctccggatcttacgccgccatccatcacgatcactccaact gctgtggagatgctcaagggtacattagccaatgcacccgggagtacgttggcgctgtca atcgatagtcgtttccaacctacttttgagctggcgccaattaatacgcaggcaattgct gcagaatctaacggtttacgggtgcagtttgatcttgccagtgcgcgtcgtgctgagggc atcacgattgattgggttgatgatatccgtggccaggggttagtgattgacaatccaaac gtgcctcagccgattcaggagttatcgcccggtgatgcggcggctcaggttgatgcgggt gttctgacgttggtggatgtgcgccctgctgatgagcgtgcggttgcttcggtcgctgtg ccgttccgcaccatggacggcggcgaacgtgctgttctggagcagcttcccaaagggacg ccgctggcattcttgtgtcatcacggcgggcgtagtctccaggccgccgagcatttccgt agtctggggtttacctctatctacaatatcggtggtggcattgatgcttggtccacccag gttgatcctggcgtgccgaagtattga |
| Q87BN9 | UniRef cluster | -------------------------------------------RSRIETLLHSNRVVLFMKGRPGMPQCGFSA-KAAGILQALGV---EYAH---VNVLD---DQEIREGIKRYG----DWP----------TIPQLYIDGE--------LIGGSDI----VSQMYENGELSTLLGVAAPDL------------ | atgtctcttgattctgcattgcgttcgcgcatcgaaacgttgttgcattccaaccgtgtt gttttgtttatgaaggggcgtccggggatgccgcagtgcggtttttctgctaaggcggca ggcattctccaggcacttggtgttgagtacgcgcatgtcaatgtattggacgaccaagag atacgagaggggatcaaacgctatggcgattggccaacaattccgcagttgtacattgat ggcgagttgatcggtggcagtgacatcgtgtcgcagatgtatgagaacggggagttgagc actttgcttggtgtggctgctccggaccttacgccgccatccatcacgatcactccaact gctgtggagatgctcaagggtacattagccaatgcacccgggagtacgttggcactgtca atcgatagtcgtttccaacctacttttgagctggcgccaattaatacgcaggcaattgct gcagaatataacggtttacgggtgcagtttgatcttgccagtgcgcgtcgtgctgagggc atcacgattgattgggttgatgatatccgtggccaggggttagtgattgacaatccaaac gcgcctcagccgattcaggagttatcgcccggtgatgcggcggctcaggttgatgcgggt gttctgacgttggtggatgtgcgccctgctgatgagcgtgcggttgcttcggtcgctgtg ccgttccgcaccatggacggcggcgaacgtgctgttctggagcagcttcccaaagggacg ccgctggcattcttgtgtcatcacggcgggcgtagtctccaggccgccgagcatttccgt agtctggggtttacctctatctacaatatcggtggtggcattgatgcttggtccacccag gttgatcctggcgtgccgaagtattga |
| Q3RDN3 | UniRef cluster | -------------------------------------------RSRIETLLHSNRVVLFMKGRPGMPQCGFSA-KAAGILQALGV---EYAH---VNVLD---DQEIREGIKRYG----DWP----------TIPQLYIDGE--------LIGGSDI----VSQMYENGELSTLLGVAAPDL------------ | atgtctcttgattctgcattgcgttcgcgcatcgaaacgttgttgcattccaaccgtgtt gttttgtttatgaaggggcgtccggggatgccgcagtgcggtttttctgctaaggcggca ggcattctccaggcacttggtgtcgagtacgcgcatgtcaatgtattggacgaccaagag attcgagaggggatcaaacgctatggcgattggccaacaattccgcagttgtacattgat ggcgagttgatcggtggcagtgacatcgtgtcgcagatgtatgagaacggggagttgagc actttgcttggtgtggctgctccggatcttacgccgccatccatcacgatcactccaact gctgtggagatgctcaagggtacattagccaatgcacccgggagtacgttggcgctgtca atcgatagtcgtttccaacctacttttgagctggcgccaattaatacgcaggcaattgct gcagaatctaacggtttacgggtgcagtttgatcttgccagtgcgcgtcgtgctgagggc atcacgattgattgggttgatgatatccgtggccaggggttagtgattgacaatccaaac gtgcctcagccgattcaggagttatcgcccggtgatgcggcggctcaggttgatgcgggt gttctgacgttggtggatgtgcgccctgctgatgagcgtgcggttgcttcggtcgctgtg ccgttccgcaccatggacggcggcgaacgtgctgttctggagcagcttcccaaagggacg ccgctggcattcttgtgtcatcacggcgggcgtagtctccaggccgccgagcatttccgt agtctggggtttacctctatctacaatatcggtggtggcattgatgcttggtccacccag gttgatcctggcgtgccgaagtattga |
| Q3R9I2 | UniRef cluster | -------------------------------------------RSRIETLLHSNRVVLFMKGRPGMPQCGFSA-KAAGILQALGV---EYAH---VNVLD---DQEIREGIKRYG----DWP----------TIPQLYIDGE--------LIGGSDI----VSQMYENGELSTLLGVAAPDL------------ | atgtctcttgattctgcattgcgttcgcgcatcgaaacgttgttgcattccaaccgtgtt gttttgtttatgaaggggcgtccggggatgccgcagtgcggtttttctgctaaggcggca ggcattctccaggcacttggtgttgagtacgcgcatgtcaatgtattggacgaccaagag atacgagaggggatcaaacgctatggcgattggccaacaattccgcagttgtacattgat ggcgagttgatcggtggcagtgacatcgtgtcgcagatgtatgagaacggggagttgagc actttgcttggtgtggctgctccggaccttacgccgccatccatcacgatcactccaact gctgtggagatgctcaagggtacattagccaatgcacccgggagtacgttggcactgtca atcgatagtcgtttccaacctacttttgagctggcgccaattaatacgcaggcaattgct gcagaatctaacggtttacgggtgcagtttgatcttgccagtgcgcgtcgtgctgagggc atcacgattgattgggttgatgatatccgtggccaggggttagtgattgacaatccaaac gcgcctcagccgattcaggagttatcgcccggtgatgcggcggctcaggttgatgcgggt gttctgacgttggtggatgtgcgccctgctgatgagcgtgcggttgcttcggtcgctgtg ccgttccgcaccatggacggcggcgaacgtgctgttctggagcagcttcccaaagggacg ccgctggcatttttgtgtcatcacggcgggcgtagtctccaggccgccgagcatttccgt agtctggggtttacctctatctacaatatcggtggtggcattgatgcttggtccacccag gttgatcctggcgtgccgaagtattga |
| Q3R4U9 | UniRef cluster | -------------------------------------------RSRIETLLHSNRVVLFMKGRPGMPQCGFSA-KAAGILQALGV---EYAH---VNVLD---DQEIREGIKRYG----DWP----------TIPQLYIDGE--------LIGGSDI----VSQMYENGELSTLLGVAAPDL------------ | atgtctcttgattctgcattgcgttcgcgcatcgaaacgttgttgcattccaaccgtgtt gttttgtttatgaaggggcgtccggggatgccgcagtgcggtttttctgctaaggcggca ggcattctccaggcacttggtgtcgagtacgcgcatgtcaatgtattggacgaccaagag attcgagaggggatcaaacgctatggcgattggccaacaattccgcagttgtacattgat ggcgagttgatcggtggcagtgacatcgtgtcgcagatgtatgagaacggggagttgagc actttgcttggtgtggctgctccggatcttacgccgccatccatcacgatcactccaact gctgtggagatgctcaagggtacattagccaatgcacccgggagtacgttggcgctgtca atcgatagtcgtttccaacctacttttgagctggcgccaattaatacgcaggcaattgct gcagaatctaacggtttacgggtgcagtttgatcttgccagtgcgcgtcgtgctgagggc atcacgattgattgggttgatgatatccgtggccaggggttagtgattgacaatccaaac gtgcctcagccgattcaggagttatcgcccggtgatgcggcggctcaggttgatgcgggt gttctgacgttggtggatgtgcgccctgctgatgagcgtgcggttgcttcggtcgctgtg ccgttccgcaccatggacggcggcgaacgtgctgttctggagcagcttcccaaagggacg ccgctggcattcttgtgtcatcacggcgggcgtagtctccaggccgccgagcatttccgt agtctggggtttacctctatctacaatatcggtggtggcattgatgcttggtccacccag gttgatcctggcgtgccgaagtattga |
| B0U3N8 | UniRef cluster | -------------------------------------------RSRIETLLHSNRVVLFMKGRPGMPQCGFSA-KAAGILQALGV---EYAH---VNVLD---DQEIREGIKRYG----DWP----------TIPQLYIDGE--------LIGGSDI----VSQMYENGELSTLLGVAAPDL------------ | atgtctcttgattctgcattgcgttcgcgcatcgaaacgttgttgcattccaaccgtgtt gttttgtttatgaaggggcgtccggggatgccgcagtgcggtttttctgctaaggcggca ggcattctccaggcacttggtgtcgagtacgcgcatgtcaatgtattggacgaccaagag attcgagaggggatcaaacgctatggcgattggccaacaattccgcagttgtacattgat ggcgagttgatcggtggcagtgacatcgtgtcgcagatgtatgagaacggggagttgagc actttgcttggtgtggctgctccggatcttacgccgccatccatcacgatcactccaact gctgtggagatgctcaagggtacattagccaatgcacccgggagtacgttggcgctgtca atcgatagtcgtttccaacctacttttgagctggcgccaattaatacgcaggcaattgct gcagaatctaacggtttacgggtgcagtttgatcttgccagtgcgcgtcgtgctgagggc atcacgattgattgggttgatgatatccgtggccaggggttagtgattgacaatccaaac gtgcctcagccgattcaggagttatcgcccggtgatgcggcggctcaggttgatgcgggt gttctgacgttggtggatgtgcgccctgctgatgagcgtgcggttgcttcggtcgctgtg ccgttccgcaccatggacggcggcgaacgtgctgttctggagcagcttcccaaagggacg ccgctggcattcttgtgtcatcacggcgggcgtagtctccaggccgccgagcatttccgt agtctggggtttacctctatctacaatatcggtggtggcattgatgcttggtccacccag gttgatcctggcgtgccgaagtattga |
| Q87BN9 | UniRef cluster | -------------------------------------------RSRIETLLHSNRVVLFMKGRPGMPQCGFSA-KAAGILQALGV---EYAH---VNVLD---DQEIREGIKRYG----DWP----------TIPQLYIDGE--------LIGGSDI----VSQMYENGELSTLLGVAAPDL------------ | atgtctcttgattctgcattgcgttcgcgcatcgaaacgttgttgcattccaaccgtgtt gttttgtttatgaaggggcgtccggggatgccgcagtgcggtttttctgctaaggcggca ggcattctccaggcacttggtgttgagtacgcgcatgtcaatgtattggacgaccaagag atacgagaggggatcaaacgctatggcgattggccaacaattccgcagttgtacattgat ggcgagttgatcggtggcagtgacatcgtgtcgcagatgtatgagaacggggagttgagc actttgcttggtgtggctgctccggaccttacgccgccatccatcacgatcactccaact gctgtggagatgctcaagggtacattagccaatgcacccgggagtacgttggcactgtca atcgatagtcgtttccaacctacttttgagctggcgccaattaatacgcaggcaattgct gcagaatataacggtttacgggtgcagtttgatcttgccagtgcgcgtcgtgctgagggc atcacgattgattgggttgatgatatccgtggccaggggttagtgattgacaatccaaac gcgcctcagccgattcaggagttatcgcccggtgatgcggcggctcaggttgatgcgggt gttctgacgttggtggatgtgcgccctgctgatgagcgtgcggttgcttcggtcgctgtg ccgttccgcaccatggacggcggcgaacgtgctgttctggagcagcttcccaaagggacg ccgctggcattcttgtgtcatcacggcgggcgtagtctccaggccgccgagcatttccgt agtctggggtttacctctatctacaatatcggtggtggcattgatgcttggtccacccag gttgatcctggcgtgccgaagtattga |
| Q3RDN3 | UniRef cluster | -------------------------------------------RSRIETLLHSNRVVLFMKGRPGMPQCGFSA-KAAGILQALGV---EYAH---VNVLD---DQEIREGIKRYG----DWP----------TIPQLYIDGE--------LIGGSDI----VSQMYENGELSTLLGVAAPDL------------ | atgtctcttgattctgcattgcgttcgcgcatcgaaacgttgttgcattccaaccgtgtt gttttgtttatgaaggggcgtccggggatgccgcagtgcggtttttctgctaaggcggca ggcattctccaggcacttggtgtcgagtacgcgcatgtcaatgtattggacgaccaagag attcgagaggggatcaaacgctatggcgattggccaacaattccgcagttgtacattgat ggcgagttgatcggtggcagtgacatcgtgtcgcagatgtatgagaacggggagttgagc actttgcttggtgtggctgctccggatcttacgccgccatccatcacgatcactccaact gctgtggagatgctcaagggtacattagccaatgcacccgggagtacgttggcgctgtca atcgatagtcgtttccaacctacttttgagctggcgccaattaatacgcaggcaattgct gcagaatctaacggtttacgggtgcagtttgatcttgccagtgcgcgtcgtgctgagggc atcacgattgattgggttgatgatatccgtggccaggggttagtgattgacaatccaaac gtgcctcagccgattcaggagttatcgcccggtgatgcggcggctcaggttgatgcgggt gttctgacgttggtggatgtgcgccctgctgatgagcgtgcggttgcttcggtcgctgtg ccgttccgcaccatggacggcggcgaacgtgctgttctggagcagcttcccaaagggacg ccgctggcattcttgtgtcatcacggcgggcgtagtctccaggccgccgagcatttccgt agtctggggtttacctctatctacaatatcggtggtggcattgatgcttggtccacccag gttgatcctggcgtgccgaagtattga |
| Q3R9I2 | UniRef cluster | -------------------------------------------RSRIETLLHSNRVVLFMKGRPGMPQCGFSA-KAAGILQALGV---EYAH---VNVLD---DQEIREGIKRYG----DWP----------TIPQLYIDGE--------LIGGSDI----VSQMYENGELSTLLGVAAPDL------------ | atgtctcttgattctgcattgcgttcgcgcatcgaaacgttgttgcattccaaccgtgtt gttttgtttatgaaggggcgtccggggatgccgcagtgcggtttttctgctaaggcggca ggcattctccaggcacttggtgttgagtacgcgcatgtcaatgtattggacgaccaagag atacgagaggggatcaaacgctatggcgattggccaacaattccgcagttgtacattgat ggcgagttgatcggtggcagtgacatcgtgtcgcagatgtatgagaacggggagttgagc actttgcttggtgtggctgctccggaccttacgccgccatccatcacgatcactccaact gctgtggagatgctcaagggtacattagccaatgcacccgggagtacgttggcactgtca atcgatagtcgtttccaacctacttttgagctggcgccaattaatacgcaggcaattgct gcagaatctaacggtttacgggtgcagtttgatcttgccagtgcgcgtcgtgctgagggc atcacgattgattgggttgatgatatccgtggccaggggttagtgattgacaatccaaac gcgcctcagccgattcaggagttatcgcccggtgatgcggcggctcaggttgatgcgggt gttctgacgttggtggatgtgcgccctgctgatgagcgtgcggttgcttcggtcgctgtg ccgttccgcaccatggacggcggcgaacgtgctgttctggagcagcttcccaaagggacg ccgctggcatttttgtgtcatcacggcgggcgtagtctccaggccgccgagcatttccgt agtctggggtttacctctatctacaatatcggtggtggcattgatgcttggtccacccag gttgatcctggcgtgccgaagtattga |
| Q3R4U9 | UniRef cluster | -------------------------------------------RSRIETLLHSNRVVLFMKGRPGMPQCGFSA-KAAGILQALGV---EYAH---VNVLD---DQEIREGIKRYG----DWP----------TIPQLYIDGE--------LIGGSDI----VSQMYENGELSTLLGVAAPDL------------ | atgtctcttgattctgcattgcgttcgcgcatcgaaacgttgttgcattccaaccgtgtt gttttgtttatgaaggggcgtccggggatgccgcagtgcggtttttctgctaaggcggca ggcattctccaggcacttggtgtcgagtacgcgcatgtcaatgtattggacgaccaagag attcgagaggggatcaaacgctatggcgattggccaacaattccgcagttgtacattgat ggcgagttgatcggtggcagtgacatcgtgtcgcagatgtatgagaacggggagttgagc actttgcttggtgtggctgctccggatcttacgccgccatccatcacgatcactccaact gctgtggagatgctcaagggtacattagccaatgcacccgggagtacgttggcgctgtca atcgatagtcgtttccaacctacttttgagctggcgccaattaatacgcaggcaattgct gcagaatctaacggtttacgggtgcagtttgatcttgccagtgcgcgtcgtgctgagggc atcacgattgattgggttgatgatatccgtggccaggggttagtgattgacaatccaaac gtgcctcagccgattcaggagttatcgcccggtgatgcggcggctcaggttgatgcgggt gttctgacgttggtggatgtgcgccctgctgatgagcgtgcggttgcttcggtcgctgtg ccgttccgcaccatggacggcggcgaacgtgctgttctggagcagcttcccaaagggacg ccgctggcattcttgtgtcatcacggcgggcgtagtctccaggccgccgagcatttccgt agtctggggtttacctctatctacaatatcggtggtggcattgatgcttggtccacccag gttgatcctggcgtgccgaagtattga |
| B0U3N8 | UniRef cluster | -------------------------------------------RSRIETLLHSNRVVLFMKGRPGMPQCGFSA-KAAGILQALGV---EYAH---VNVLD---DQEIREGIKRYG----DWP----------TIPQLYIDGE--------LIGGSDI----VSQMYENGELSTLLGVAAPDL------------ | atgtctcttgattctgcattgcgttcgcgcatcgaaacgttgttgcattccaaccgtgtt gttttgtttatgaaggggcgtccggggatgccgcagtgcggtttttctgctaaggcggca ggcattctccaggcacttggtgtcgagtacgcgcatgtcaatgtattggacgaccaagag attcgagaggggatcaaacgctatggcgattggccaacaattccgcagttgtacattgat ggcgagttgatcggtggcagtgacatcgtgtcgcagatgtatgagaacggggagttgagc actttgcttggtgtggctgctccggatcttacgccgccatccatcacgatcactccaact gctgtggagatgctcaagggtacattagccaatgcacccgggagtacgttggcgctgtca atcgatagtcgtttccaacctacttttgagctggcgccaattaatacgcaggcaattgct gcagaatctaacggtttacgggtgcagtttgatcttgccagtgcgcgtcgtgctgagggc atcacgattgattgggttgatgatatccgtggccaggggttagtgattgacaatccaaac gtgcctcagccgattcaggagttatcgcccggtgatgcggcggctcaggttgatgcgggt gttctgacgttggtggatgtgcgccctgctgatgagcgtgcggttgcttcggtcgctgtg ccgttccgcaccatggacggcggcgaacgtgctgttctggagcagcttcccaaagggacg ccgctggcattcttgtgtcatcacggcgggcgtagtctccaggccgccgagcatttccgt agtctggggtttacctctatctacaatatcggtggtggcattgatgcttggtccacccag gttgatcctggcgtgccgaagtattga |
| Q87BN9 | UniRef cluster | -------------------------------------------RSRIETLLHSNRVVLFMKGRPGMPQCGFSA-KAAGILQALGV---EYAH---VNVLD---DQEIREGIKRYG----DWP----------TIPQLYIDGE--------LIGGSDI----VSQMYENGELSTLLGVAAPDL------------ | atgtctcttgattctgcattgcgttcgcgcatcgaaacgttgttgcattccaaccgtgtt gttttgtttatgaaggggcgtccggggatgccgcagtgcggtttttctgctaaggcggca ggcattctccaggcacttggtgttgagtacgcgcatgtcaatgtattggacgaccaagag atacgagaggggatcaaacgctatggcgattggccaacaattccgcagttgtacattgat ggcgagttgatcggtggcagtgacatcgtgtcgcagatgtatgagaacggggagttgagc actttgcttggtgtggctgctccggaccttacgccgccatccatcacgatcactccaact gctgtggagatgctcaagggtacattagccaatgcacccgggagtacgttggcactgtca atcgatagtcgtttccaacctacttttgagctggcgccaattaatacgcaggcaattgct gcagaatataacggtttacgggtgcagtttgatcttgccagtgcgcgtcgtgctgagggc atcacgattgattgggttgatgatatccgtggccaggggttagtgattgacaatccaaac gcgcctcagccgattcaggagttatcgcccggtgatgcggcggctcaggttgatgcgggt gttctgacgttggtggatgtgcgccctgctgatgagcgtgcggttgcttcggtcgctgtg ccgttccgcaccatggacggcggcgaacgtgctgttctggagcagcttcccaaagggacg ccgctggcattcttgtgtcatcacggcgggcgtagtctccaggccgccgagcatttccgt agtctggggtttacctctatctacaatatcggtggtggcattgatgcttggtccacccag gttgatcctggcgtgccgaagtattga |
| Q3RDN3 | UniRef cluster | -------------------------------------------RSRIETLLHSNRVVLFMKGRPGMPQCGFSA-KAAGILQALGV---EYAH---VNVLD---DQEIREGIKRYG----DWP----------TIPQLYIDGE--------LIGGSDI----VSQMYENGELSTLLGVAAPDL------------ | atgtctcttgattctgcattgcgttcgcgcatcgaaacgttgttgcattccaaccgtgtt gttttgtttatgaaggggcgtccggggatgccgcagtgcggtttttctgctaaggcggca ggcattctccaggcacttggtgtcgagtacgcgcatgtcaatgtattggacgaccaagag attcgagaggggatcaaacgctatggcgattggccaacaattccgcagttgtacattgat ggcgagttgatcggtggcagtgacatcgtgtcgcagatgtatgagaacggggagttgagc actttgcttggtgtggctgctccggatcttacgccgccatccatcacgatcactccaact gctgtggagatgctcaagggtacattagccaatgcacccgggagtacgttggcgctgtca atcgatagtcgtttccaacctacttttgagctggcgccaattaatacgcaggcaattgct gcagaatctaacggtttacgggtgcagtttgatcttgccagtgcgcgtcgtgctgagggc atcacgattgattgggttgatgatatccgtggccaggggttagtgattgacaatccaaac gtgcctcagccgattcaggagttatcgcccggtgatgcggcggctcaggttgatgcgggt gttctgacgttggtggatgtgcgccctgctgatgagcgtgcggttgcttcggtcgctgtg ccgttccgcaccatggacggcggcgaacgtgctgttctggagcagcttcccaaagggacg ccgctggcattcttgtgtcatcacggcgggcgtagtctccaggccgccgagcatttccgt agtctggggtttacctctatctacaatatcggtggtggcattgatgcttggtccacccag gttgatcctggcgtgccgaagtattga |
| Q3R9I2 | UniRef cluster | -------------------------------------------RSRIETLLHSNRVVLFMKGRPGMPQCGFSA-KAAGILQALGV---EYAH---VNVLD---DQEIREGIKRYG----DWP----------TIPQLYIDGE--------LIGGSDI----VSQMYENGELSTLLGVAAPDL------------ | atgtctcttgattctgcattgcgttcgcgcatcgaaacgttgttgcattccaaccgtgtt gttttgtttatgaaggggcgtccggggatgccgcagtgcggtttttctgctaaggcggca ggcattctccaggcacttggtgttgagtacgcgcatgtcaatgtattggacgaccaagag atacgagaggggatcaaacgctatggcgattggccaacaattccgcagttgtacattgat ggcgagttgatcggtggcagtgacatcgtgtcgcagatgtatgagaacggggagttgagc actttgcttggtgtggctgctccggaccttacgccgccatccatcacgatcactccaact gctgtggagatgctcaagggtacattagccaatgcacccgggagtacgttggcactgtca atcgatagtcgtttccaacctacttttgagctggcgccaattaatacgcaggcaattgct gcagaatctaacggtttacgggtgcagtttgatcttgccagtgcgcgtcgtgctgagggc atcacgattgattgggttgatgatatccgtggccaggggttagtgattgacaatccaaac gcgcctcagccgattcaggagttatcgcccggtgatgcggcggctcaggttgatgcgggt gttctgacgttggtggatgtgcgccctgctgatgagcgtgcggttgcttcggtcgctgtg ccgttccgcaccatggacggcggcgaacgtgctgttctggagcagcttcccaaagggacg ccgctggcatttttgtgtcatcacggcgggcgtagtctccaggccgccgagcatttccgt agtctggggtttacctctatctacaatatcggtggtggcattgatgcttggtccacccag gttgatcctggcgtgccgaagtattga |
| Q3R4U9 | UniRef cluster | -------------------------------------------RSRIETLLHSNRVVLFMKGRPGMPQCGFSA-KAAGILQALGV---EYAH---VNVLD---DQEIREGIKRYG----DWP----------TIPQLYIDGE--------LIGGSDI----VSQMYENGELSTLLGVAAPDL------------ | atgtctcttgattctgcattgcgttcgcgcatcgaaacgttgttgcattccaaccgtgtt gttttgtttatgaaggggcgtccggggatgccgcagtgcggtttttctgctaaggcggca ggcattctccaggcacttggtgtcgagtacgcgcatgtcaatgtattggacgaccaagag attcgagaggggatcaaacgctatggcgattggccaacaattccgcagttgtacattgat ggcgagttgatcggtggcagtgacatcgtgtcgcagatgtatgagaacggggagttgagc actttgcttggtgtggctgctccggatcttacgccgccatccatcacgatcactccaact gctgtggagatgctcaagggtacattagccaatgcacccgggagtacgttggcgctgtca atcgatagtcgtttccaacctacttttgagctggcgccaattaatacgcaggcaattgct gcagaatctaacggtttacgggtgcagtttgatcttgccagtgcgcgtcgtgctgagggc atcacgattgattgggttgatgatatccgtggccaggggttagtgattgacaatccaaac gtgcctcagccgattcaggagttatcgcccggtgatgcggcggctcaggttgatgcgggt gttctgacgttggtggatgtgcgccctgctgatgagcgtgcggttgcttcggtcgctgtg ccgttccgcaccatggacggcggcgaacgtgctgttctggagcagcttcccaaagggacg ccgctggcattcttgtgtcatcacggcgggcgtagtctccaggccgccgagcatttccgt agtctggggtttacctctatctacaatatcggtggtggcattgatgcttggtccacccag gttgatcctggcgtgccgaagtattga |
| B0U3N8 | UniRef cluster | -------------------------------------------RSRIETLLHSNRVVLFMKGRPGMPQCGFSA-KAAGILQALGV---EYAH---VNVLD---DQEIREGIKRYG----DWP----------TIPQLYIDGE--------LIGGSDI----VSQMYENGELSTLLGVAAPDL------------ | atgtctcttgattctgcattgcgttcgcgcatcgaaacgttgttgcattccaaccgtgtt gttttgtttatgaaggggcgtccggggatgccgcagtgcggtttttctgctaaggcggca ggcattctccaggcacttggtgtcgagtacgcgcatgtcaatgtattggacgaccaagag attcgagaggggatcaaacgctatggcgattggccaacaattccgcagttgtacattgat ggcgagttgatcggtggcagtgacatcgtgtcgcagatgtatgagaacggggagttgagc actttgcttggtgtggctgctccggatcttacgccgccatccatcacgatcactccaact gctgtggagatgctcaagggtacattagccaatgcacccgggagtacgttggcgctgtca atcgatagtcgtttccaacctacttttgagctggcgccaattaatacgcaggcaattgct gcagaatctaacggtttacgggtgcagtttgatcttgccagtgcgcgtcgtgctgagggc atcacgattgattgggttgatgatatccgtggccaggggttagtgattgacaatccaaac gtgcctcagccgattcaggagttatcgcccggtgatgcggcggctcaggttgatgcgggt gttctgacgttggtggatgtgcgccctgctgatgagcgtgcggttgcttcggtcgctgtg ccgttccgcaccatggacggcggcgaacgtgctgttctggagcagcttcccaaagggacg ccgctggcattcttgtgtcatcacggcgggcgtagtctccaggccgccgagcatttccgt agtctggggtttacctctatctacaatatcggtggtggcattgatgcttggtccacccag gttgatcctggcgtgccgaagtattga |
| A9GKU4 | UniRef cluster | -------------------------------------------RARIATIIRESDVVLFMKGTRTVPQCGFSA-TVVNILDEHLP---EYQT---VDVLS---DPALRDGIKEFS----SWP----------TIPQLYVRGE--------FIGGCDI----VKELHATGELVKALGLAQGEA------------ | atgagcctcagcgagcccatgcgggccaggatcgccaccatcatccgcgagagcgatgtc gtcctgttcatgaaaggcacgcgcaccgtgccgcagtgcgggttttccgccacggtggtg aacatcctcgacgagcacctccccgagtaccagacggtggacgtgctctcggatcccgcg ctgcgcgacggcatcaaggagttctcgagctggccgacgatcccgcagctctacgtccgc ggcgagttcatcggcggctgcgacatcgtgaaggagctgcacgcgaccggggagctcgtg aaggcgctcgggctcgcgcagggcgaggcgcaggcgccgcccacgctccgcgtgaccgcg gcggcgctcgcggcgttcgcggccgcgaaagagtccgaggccgacttcgtccacatcgag atcgacccgtcctacaactacggcctttacttcggcccgcggcaggcaggcgacgtcgag gcccaggcgggcggggcggtgttcctgctcgaccgcgcgagcgcgcggcgcgccgagggg ctgtcgatcgacttcgtcgagggcccctccggcggcggcttcaagctggagagcccgaac gagccgccgaaggtcaagcagatctcgtcgaccgcgctcaaggcccttctgaacagcggc gaggcgatcgagctgttcgacgtccggaccgagcaggagcgcaagatcgccacgatcgag ggggcccgtcacctcgatcaggcagggcagcggtacctggagacgctcgcgaaggacgcg cggatcgtcttccactgccaccacggcggccgcagccaggcagccgccgagcactacctc acgaagggctacaggaacctttacaacctccagggcggaatcgatgcgtggtcgcaagat gtcgacccctcggtgccgcggtactga |
| A6GID3 | UniRef cluster | -------------------------------------------RKTLQDLVTRERVVLFMKGNRRSPQCGFSA-AVVETLDLWLD---SYHT---VDVLA---DEAIREGVKLFS----EWP----------TIPQLYVGGE--------FLGGADI----IRELEDTGELPKALGVPDTLE------------ | gtggccagcaccctcgacgacgcgacccgcaaaaccctccaagacctcgtgacccgcgag cgcgtggtcctgttcatgaagggcaaccgccgctcgccgcagtgcgggttctcggccgcg gtggtcgagaccctcgacctgtggctcgacagctaccacaccgtcgacgtgctcgccgac gaggcgatccgcgagggggtcaagctgttctccgagtggccgacgatcccccagctctac gtcggcggggagttcctcggcggggcggacatcatccgcgagctcgaggacaccggcgag ctgcccaaggccctgggcgtgcccgacaccctcgagcccccgcagatcaccatctgcgcc ggggcggcggcgcagatcaaggcggccttcgactcccccgaggtcgacgaggccgaccag ctgcgcctggccatcgacgcgcgctaccacaacgacctgtccatcggcccgcgccggccc ggggacgtggccgtcgagagccagggcctgaccctgctcctcgatcgccgcagcgcccgg cgggcccgcggcctgagcatcgacttcgtcgacggacccgacggcacgggcttcaagatc gacaaccccaacgcgccggcgagcgtgcagtcgatctcggccaaggagctgcaggcgcgc atggacgcggccgaggacgaaggcatggagttccacctcctcgacgtgcgcaccccggcc gagcgcgagctggccgtggtcgagggctcggtgctcctcgacggcgagcgcgcggccgcc ctcgaggatctgccccgggacacgccgctgtacttcatgtgccaccacgggatgcgcagc atgcgagcggcggagcacttcgccagcgtcggctttcgccaggtgttcaacgtgaccggc gggatcgcggcgtggtcgagcgaggtcgacccgaaggtgccgcagtactaa |
| B0RNY7 | UniRef cluster | -----------------------------------------------------------------MPQCGFSA-KAIGVLDGLGI---DYAH---VNVLA---DQEIREGIKAYG----DWP----------TIPQLYVDGE--------LIGGSDI----IVQMADSGELSSMLGLQAPDR------------ | atgtccctcgatcccgccctgcgttcccgcatcgatacgctgctgcaatccaaccgcgtg gtcctcttcatgaaaggccagcccggcatgccgcagtgcggcttctcggccaaggccatc ggcgtgctggacggcctgggcatcgactatgcccacgtcaacgtgctggccgaccaggaa atccgcgaaggcatcaaggcctacggcgactggccgaccattccgcagctgtatgtggat ggcgagctgatcggcggcagcgacatcatcgtgcagatggccgacagcggcgagctgagc agcatgctcggcctgcaggcaccggaccgcagcccgcccaagatcaccatcaccccggct gcggtcgagatgctcaagggtgcgttggccgatgcaccggatgcctcgctgacgctggcc atcgatgccaatttccagccgaacttccagctggcgccaaccaacccgaacgctatcgcc gccgagtccaacgggctgcgcgtgcagttcgacctggccagcgcgcgccgcgccgacggc atcaccatcgactgggtggacgacatccgtggccgcggcctggccatcgacaaccccaac gcgcccaagccggtgcaggagctggcggtgcgcgatgccgacgaccgtctcaaggccggt acgctgactgtggtggacgtgcgcccggccgatgagcgtgcgctggctacggtggcagcg ccgttccgcaccctggacgcacacgagcgcctggagatcgagcaactgcccaaggacacc ccgctggcgttcctgtgccaccgcggcgggcgtagcctgcaggcggccgagcatttccgc ggcctgggctttaccaacgtctacaacgtcaccggcggcatcgatgcctggtcggacgcg gtggacaacggcgtgcccaagtactga |
| Q8P5H3 | UniRef cluster | -----------------------------------------------------------------MPQCGFSA-KAIGVLDGLGI---DYAH---VNVLA---DQEIREGIKAYG----DWP----------TIPQLYVDGE--------LIGGSDI----IVQMADSGELSSMLGLQAPDR------------ | atgccgcagtgcggcttctcggccaaggccatcggcgtgctggacggcctgggcatcgac tatgcccacgtcaacgtgctggccgaccaggaaatccgcgaaggcatcaaggcctacggc gactggccgaccattccgcagctgtatgtggatggcgagctgatcggcggcagcgacatc atcgtgcagatggccgacagcggcgagctgagcagcatgctcggcctgcaggcaccggac cgcagcccgcccaagatcaccatcaccccggctgcggtcgagatgctcaagggtgcgttg gccgatgcaccggatgcctcgctgacgctggccatcgatgccaatttccagccgaacttc cagctggcgccaaccaacccgaacgctatcgccgccgagtccaacgggctgcgcgtgcag ttcgacctggccagcgcgcgccgcgccgacggcatcaccatcgactgggtggacgacatc cgtggccgcggcctggccatcgacaaccccaacgcgcccaagccggtgcaggagctggcg gtgcgcgatgccgacgaccgtctcaaggccggtacgctgactgtggtggacgtgcgcccg gccgatgagcgtgcgctggctacggtggcagcgccgttccgcaccctggacgcacacgag cgcctggagatcgagcaactgcccaaggacaccccgctggcgttcctgtgccaccgcggc gggcgtagcctgcaggcggccgagcatttccgcggcctgggctttaccaacgtctacaac gtcaccggcggcatcgatgcctggtcggacgcggtggacaacggcgtgcccaagtactga |
| Q4UYK2 | UniRef cluster | -----------------------------------------------------------------MPQCGFSA-KAIGVLDGLGI---DYAH---VNVLA---DQEIREGIKAYG----DWP----------TIPQLYVDGE--------LIGGSDI----IVQMADSGELSSMLGLQAPDR------------ | atgccgcagtgcggcttctcggccaaggccatcggcgtgctggacggcctgggcatcgac tatgcccacgtcaacgtgctggccgaccaggaaatccgcgaaggcatcaaggcctacggc gactggccgaccattccgcagctgtatgtggatggcgagctgatcggcggcagcgacatc atcgtgcagatggccgacagcggcgagctgagcagcatgctcggcctgcaggcaccggac cgcagcccgcccaagatcaccatcaccccggctgcggtcgagatgctcaagggtgcgttg gccgatgcaccggatgcctcgctgacgctggccatcgatgccaatttccagccgaacttc cagctggcgccaaccaacccgaacgctatcgccgccgagtccaacgggctgcgcgtgcag ttcgacctggccagcgcgcgccgcgccgacggcatcaccatcgactgggtggacgacatc cgtggccgcggcctggccatcgacaaccccaacgcgcccaagccggtgcaggagctggcg gtgcgcgatgccgacgaccgtctcaaggccggtacgctgactgtggtggacgtgcgcccg gccgatgagcgtgcgctggctacggtggcagcgccgttccgcaccctggacgcacacgag cgcctggagatcgagcaactgcccaaggacaccccgctggcgttcctgtgccaccgcggc gggcgtagcctgcaggcggccgagcatttccgcggcctgggctttaccaacgtctacaac gtcaccggcggcatcgatgcctggtcggacgcggtggacaacggcgtgcccaagtactga |
| Q9PAV1 | UniRef cluster | -----------------------------------------------------------------MPQCGFSA-KAAGILQALGV---EYAH---VNVLD---DQEIREGIKRYG----DWP----------TIPQLYIDGE--------LIGGSDI----VSQMYENGELSALLGVAAPDL------------ | atgccgcagtgcggtttttctgctaaggctgcaggcatcctccaggcacttggtgtcgag tacgcgcatgtcaatgtattggacgaccaagagattcgagaggggatcaaacgctatggc gattggccaacaattcctcagttgtacattgatggtgagttgatcggtggcagtgacatc gtgtcgcagatgtatgagaacggggagttgagcgctttgcttggtgtggctgctccggat cttacgccgccatccatcacgatcactccaactgctgtggagatgctcaagagtacatta gccaatgcgcccgggagcgcgttggcactgtcaatcgatagtcgtttccaacctactttt gagctgacgccaattaatacgcaggcgattgctgcagaatctaacggtttacgggtgcag tttgatcttgccagtgcacgtcgtgctgagggcatcacgattgattgggttgatgatatc cgtggccaggggttagtgattgacaatccaaacgcgcctcagccgattcaggagttatcg ccaggtgatgcggcggctcaggttggtgcgggtgctctgacgttggtggatgtgcgccct gctgatgagcgtgcgattgcttcggtcgctgtgccgtttcgcaccatggacggtggcgaa cgtgctgctctggagcagcttcccaaagagacgccgctagcattcttgtgtcatcacggc gggcgtagtctccaggccgcggagcatttccgtagtctggggtttacctctatctacaat atcggtggtggcattgatgcttggtccatccaggttgatgcgagtgtgccgaagtattga |
| Q3IDU3 | UniRef cluster | -------------------------------------------DYINPKQAKPEPVTVFTK-----PGCPFCS-KAKALLTEKG---FAFEE----IVMG---SGASLTSLKAVS----GRE----------TVPQVFIGGK--------HIGGSDD----LEKYFA--------------------------- | atgttaaacaatattgaaggcaaaacaatcccaagcgttacatttgcaactcgtcaaaac gatgaatggaaatcagtaacaaccgacgacatttttaaaggtaaaactgttgttgtattt tcactaccgggtgcatttacaccaacgtgttcgtctactcatttaccacgttacaacgaa ctagctggcgtacttaagcaaaacggcgttgacgaaattgtatgtttatcggtaaacgac acctttgtaatgaacgcatgggctgagcatcaagaagcgcaaaacattactttactacca gacggtaacggcgaatttactgacggcatgggcatgttagtagataaaaacgacctaggt tttggtaagcgcagctggagatactctatgcttgtaaaagatggcgtgatcgacaaaatg ttcatcgaaccagatttaccaggcgacccgtttgaagtatctgacgcagatacaatgctt gactacatcaaccctaagcaagctaaaccagagccagtaactgtatttactaaacctggt tgtccgttctgttcaaaagctaaagcgcttcttacagaaaaaggctttgcatttgaagaa attgttatgggctcaggcgcatcactaacaagccttaaagcagtatctggccgtgaaacc gttccacaagtatttattggcggtaagcacattggtggctcagacgatttagaaaaatac tttgcttaa |
| Q9KNU3 | UniRef cluster | -------------------------------------------KYIAPQYKVQESVTIFTK-----PGCPYCA-KAKQALIDAG---LQYEE----LILG---KDATTVSLRAVS----GRT----------TVPQVFIGGK--------HIGGSDD----LEVYLNQ-------------------------- | atgaggaacacaatgtttacatctaaagaaggtcaaaccattccacaggttacttttcct actcgccaaggtgacgcttgggtcaatgtgactagcgatgaactgttcaaaggcaaaacc gttatcgtgtttagcttgccgggtgcctttactccaacctgttcatccactcacctaccg cgctacaacgagctgtttcctgtctttaaagagcatggtgtcgacagcattctgtgcgta tcggtcaacgatactttcgtgatgaatgcttggaaagatgaccaaaatgccgacaacatc accttcattcctgatggtaatggtgaatttaccgatggtatgggcatgctggtggataaa aatgaccttggctttggtaaacgctcatggcgctacagcatgctggttaaagacggtgtg gtagaaaaaatgtttatcgaaccgaatgagccgggcgacccgttcaaagtatcggacgcc gataccatgctcaaatacattgcccctcaatacaaggtgcaagaatcagtgactattttc actaagccaggctgtccttattgcgccaaggcgaaacaagcgctgattgatgccggtcta cagtatgaagagctgattttaggtaaagacgctaccacagtgagtctgcgcgccgtttct ggccgtaccacggtgccgcaagtgtttatcggtggtaaacacattggtggtagcgacgac ttagaagtctacctaaatcaataa |
| A6Y1K9 | UniRef cluster | -------------------------------------------KYIAPQYKVQESVTIFTK-----PGCPYCA-KAKQALIDAG---LQYEE----LILG---KDATTVSLRAVS----GRT----------TVPQVFIGGK--------HIGGSDD----LEVYLNQ-------------------------- | atgaggaacacaatgtttacatctaaagaaggtcaaaccattccacaggttacttttcct actcgccaaggtgacgcttgggtcaatgtgactagcgatgagctgttcaaaggcaaaacc gttatcgtgtttagcttgccgggtgcctttactccaacctgttcatccactcacctaccg cgctacaacgagctgtttcctgtctttaaagagcatggtgtcgacagcattctgtgcgta tcggtcaacgatactttcgtgatgaatgcttggaaagatgaccaaaatgccgacaacatc acctttattcctgatggtaatggtgaatttaccgatggtatggggatgctggtggataaa aatgaccttggctttggtaaacgctcatggcgctacagcatgctggttaaagacggtgtg gtagaaaaaatgtttatcgaaccgaatgagccgggcgacccgttcaaagtatcggacgcc gataccatgctcaaatacattgcccctcaatacaaggtgcaagaatcagtgactattttc actaagcctggatgtccttattgcgccaaggcgaaacaagcgctgattgatgccggtcta cagtatgaagagctgattttaggtaaagacgccaccacagtgagcctgcgcgctgtttct ggccgtaccacggtgccgcaagtgtttatcggtggtaaacacattggcggcagcgacgac ttagaagtctacctaaatcaataa |
| A6XU96 | UniRef cluster | -------------------------------------------KYIAPQYKVQESVTIFTK-----PGCPYCA-KAKQALIDAG---LQYEE----LILG---KDATTVSLRAVS----GRT----------TVPQVFIGGK--------HIGGSDD----LEVYLNQ-------------------------- | atgaggaacacaatgtttacatctaaagaaggtcaaaccattccacaggttacttttcct actcgccaaggtgacgcttgggtcaatgtgactagcgatgaactgttcaaaggcaaaacc gttatcgtgtttagcttgccgggtgcctttactccaacctgttcatccactcacctaccg cgctacaacgagctgtttcctgtctttaaagagcatggtgtcgacagcattctgtgcgta tcggtcaacgatactttcgtgatgaatgcttggaaagatgaccaaaatgccgacaacatc accttcattcctgatggtaatggtgaatttaccgatggtatgggcatgttggtggataaa aatgaccttggctttggtaaacgctcatggcgctacagcatgctggttaaagatggtgtg gtagaaaaaatgtttatcgaaccgaatgagccgggcgacccgttcaaagtatcggacgcc gataccatgctcaaatacattgcccctcaatacaaggtgcaagaatcagtgactattttc actaagcctggatgtccttattgcgccaaggcgaaacaagcgctgattgatgccggtcta cagtatgaagagctgattttaggtaaagacgccaccacagtgagcctgcgcgctgtttct ggccgtaccacggtgccgcaagtgtttatcggtggtaaacacattggtggcagcgacgac ttagaagtctacctaaatcaataa |
| A6AFP4 | UniRef cluster | -------------------------------------------KYIAPQYKVQESVTIFTK-----PGCPYCA-KAKQALIDAG---LQYEE----LILG---KDATTVSLRAVS----GRT----------TVPQVFIGGK--------HIGGSDD----LEVYLNQ-------------------------- | atgaggaacacaatgtttacatctaaagaaggtcaaaccattccacaggttacttttcct actcgccaaggtgacgcttgggtcaatgtgactagcgatgaactgttcaaaggcaaaacc gttatcgtgtttagcttgccgggtgcctttactccaacctgttcatccactcacctaccg cgctacaacgagctgtatcctgtctttaaagagaatggtgtcgacagcattctgtgcgta tcggtcaacgatactttcgtgatgaatgcttggaaagatgaccaaaatgccgacaacatc accttcattcctgatggtaatggtgaatttaccgatggtatgggcatgctggtggataaa aatgaccttggctttggtaaacgctcatggcgctacagcatgctggttaaagacggtgtg gtagaaaaaatgtttatcgaaccgaatgagccgggcgacccgttcaaagtatcggacgcc gataccatgctcaaatacattgcccctcaatacaaggtgcaagaatcagtgactattttc actaagccaggctgtccttattgcgccaaggcgaaacaagcgctgattgatgccggtcta cagtatgaagagctgattttaggtaaagacgccaccacagtgagcctgcgcgctgtttct ggccgtaccacggtgccgcaagtgtttatcggtggtaaacacattggtggcagcgacgac ttagaagtctacctaaatcaataa |
| A6A149 | UniRef cluster | -------------------------------------------KYIAPQYKVQESVTIFTK-----PGCPYCA-KAKQALIDAG---LQYEE----LILG---KDATTVSLRAVS----GRT----------TVPQVFIGGK--------HIGGSDD----LEVYLNQ-------------------------- | atgaggaacacaatgtttacatctaaagaaggtcaaaccattccacaggttacttttcct actcgccaaggtgacgcttgggtcaatgtgactagcgatgagctgttcaaaggcaaaacc gttatcgtgtttagcttgccgggtgcctttactccaacctgttcatccactcacctaccg cgctacaacgagctgtttcctgtctttaaagagcatggtgtcgacagcattctgtgcgta tcggtcaacgatactttcgtgatgaatgcttggaaagatgaccaaaacgccgacaacatc accttcattcctgatggtaatggtgaatttaccgatggtatgggcatgttggtggataaa aatgaccttggctttggtaaacgctcatggcgctacagcatgctggttaaagacggtgtg gtagaaaaaatgtttatcgaaccgaatgagccgggcgacccgttcaaagtatcggacgcc gataccatgctcaaatacattgcccctcaatacaaggtgcaagaatcagtgactattttc actaagcctggctgtccttattgcgccaaggcgaaacaagcgctgattgatgccggtcta cagtatgaagagctgattttaggtaaagacgccaccacagtgagcctgcgcgctgtttct ggccgtaccacggtgccgcaagtgtttatcggtggtaaacacattggtggcagcgacgac ttagaagtctacctaaatcaataa |
| A5F510 | UniRef cluster | -------------------------------------------KYIAPQYKVQESVTIFTK-----PGCPYCA-KAKQALIDAG---LQYEE----LILG---KDATTVSLRAVS----GRT----------TVPQVFIGGK--------HIGGSDD----LEVYLNQ-------------------------- | atgaggaacacaatgtttacatctaaagaaggtcaaaccattccacaggttacttttcct actcgccaaggtgacgcttgggtcaatgtgactagcgatgaactgttcaaaggcaaaacc gttatcgtgtttagcttgccgggtgcctttactccaacctgttcatccactcacctaccg cgctacaacgagctgtttcctgtctttaaagagcatggtgtcgacagcattctgtgcgta tcggtcaacgatactttcgtgatgaatgcttggaaagatgaccaaaatgccgacaacatc accttcattcctgatggtaatggtgaatttaccgatggtatgggcatgctggtggataaa aatgaccttggctttggtaaacgctcatggcgctacagcatgctggttaaagacggtgtg gtagaaaaaatgtttatcgaaccgaatgagccgggcgacccgttcaaagtatcggacgcc gataccatgctcaaatacattgcccctcaatacaaggtgcaagaatcagtgactattttc actaagccaggctgtccttattgcgccaaggcgaaacaagcgctgattgatgccggtcta cagtatgaagagctgattttaggtaaagacgctaccacagtgagtctgcgcgccgtttct ggccgtaccacggtgccgcaagtgtttatcggtggtaaacacattggtggtagcgacgac ttagaagtctacctaaatcaataa |
| A3GXC3 | UniRef cluster | -------------------------------------------KYIAPQYKVQESVTIFTK-----PGCPYCA-KAKQALIDAG---LQYEE----LILG---KDATTVSLRAVS----GRT----------TVPQVFIGGK--------HIGGSDD----LEVYLNQ-------------------------- | atgaggaacacaatgtttacatctaaagaaggtcaaaccattccacaggttacttttcct actcgccaaggtgacgcttgggtcaatgtgactagcgatgaactgttcaaaggcaaaacc gttatcgtgtttagcttgccgggtgcctttactccaacctgttcatccactcacctaccg cgctacaacgagctgtttcctgtctttaaagagcatggtgtcgacagcattctgtgcgta tcggtcaacgatactttcgtgatgaatgcttggaaagatgaccaaaatgccgacaacatc accttcattcctgatggtaatggtgaatttaccgatggtatgggcatgctggtggataaa aatgaccttggctttggtaaacgctcatggcgctacagcatgctggttaaagacggtgtg gtagaaaaaatgtttatcgaaccgaatgagccgggcgacccgttcaaagtatcggacgcc gataccatgctcaaatacattgcccctcaatacaaggtgcaagaatcagtgactattttc actaagccaggctgtccttattgcgccaaggcgaaacaagcgctgattgatgccggtcta cagtatgaagagctgattttaggtaaagacgctaccacagtgagtctgcgcgccgtttct ggccgtaccacggtgccgcaagtgtttatcggtggtaaacacattggtggtagcgacgac ttagaagtctacctaaatcaataa |
| A3GRD5 | UniRef cluster | -------------------------------------------KYIAPQYKVQESVTIFTK-----PGCPYCA-KAKQALIDAG---LQYEE----LILG---KDATTVSLRAVS----GRT----------TVPQVFIGGK--------HIGGSDD----LEVYLNQ-------------------------- | atgaggaacacaatgtttacatctaaagaaggtcaaaccattccacaggttacttttcct actcgccaaggtgacgcttgggtcaatgtgactagcgatgaactgttcaaaggcaaaacc gttatcgtgtttagcttgccgggtgcctttactccaacctgttcatccactcacctaccg cgctacaacgagctgtttcctgtctttaaagagcatggtgtcgacagcattctgtgcgta tcggtcaacgatactttcgtgatgaatgcttggaaagatgaccaaaatgccgacaacatc accttcattcctgatggtaatggtgaatttaccgatggtatgggcatgctggtggataaa aatgaccttggctttggtaaacgctcatggcgctacagcatgctggttaaagacggtgtg gtagaaaaaatgtttatcgaaccgaatgagccgggcgacccgttcaaagtatcggacgcc gataccatgctcaaatacattgcccctcaatacaaggtgcaagaatcagtgactattttc actaagccaggctgtccttattgcgccaaggcgaaacaagcgctgattgatgccggtcta cagtatgaagagctgattttaggtaaagacgctaccacagtgagtctgcgcgccgtttct ggccgtaccacggtgccgcaagtgtttatcggtggtaaacacattggtggtagcgacgac ttagaagtctacctaaatcaataa |
| A3EJ81 | UniRef cluster | -------------------------------------------KYIAPQYKVQESVTIFTK-----PGCPYCA-KAKQALIDAG---LQYEE----LILG---KDATTVSLRAVS----GRT----------TVPQVFIGGK--------HIGGSDD----LEVYLNQ-------------------------- | atgaggaacacaatgtttacatctaaagaaggtcaaaccattccacaggttacttttcct actcgccaaggtgacgcttgggtcaatgtgactagcgatgaactgttcaaaggcaaaacc gttatcgtgtttagcttgccgggtgcctttactccaacctgttcatccactcacctaccg cgctacaacgagctgtttcctgtctttaaagagcatggtgtcgacagcattctgtgcgta tcggtcaacgatactttcgtgatgaatgcttggaaagatgaccaaaatgccgacaacatc accttcattcctgatggtaatggtgaatttaccgatggtatgggcatgctggtggataaa aatgaccttggctttggtaaacgctcatggcgctacagcatgctggttaaagacggtgtg gtagaaaaaatgtttatcgaaccgaatgagccgggcgacccgttcaaagtatcggacgcc gataccatgctcaaatacattgcccctcaatacaaggtgcaagaatcagtgactattttc actaagcctggctgtccttattgcgccaaggcgaaacaagcgctgattgatgccggtcta cagtatgaagagctgattttaggtaaagacgccaccacagtgagcctgcgcgctgtttct ggccgtaccacggtgccgcaagtgtttatcggtggtaaacacattggtggcagcgacgac ttagaagtctacctaaatcaataa |
| A3EB97 | UniRef cluster | -------------------------------------------KYIAPQYKVQESVTIFTK-----PGCPYCA-KAKQALIDAG---LQYEE----LILG---KDATTVSLRAVS----GRT----------TVPQVFIGGK--------HIGGSDD----LEVYLNQ-------------------------- | atgaggaacacaatgtttacatctaaagaaggtcaaaccattccacaggttacttttcct actcgccaaggtgacgcttgggtcaatgtgactagcgatgaactgttcaaaggcaaaacc gttatcgtgtttagcttgccgggtgcctttactccaacctgttcatccactcacctaccg cgctacaacgagctgtttcctgtctttaaagagcatggtgtcgacagcattctgtgcgta tcggtcaacgatactttcgtgatgaatgcttggaaagatgaccaaaatgccgacaacatc accttcattcctgatggtaatggtgaatttaccgatggtatgggcatgctggtggataaa aatgaccttggctttggtaaacgctcatggcgctacagcatgctggttaaagacggtgtg gtagaaaaaatgtttatcgaaccgaatgagccgggcgacccgttcaaagtatcggacgcc gataccatgctcaaatacattgcccctcaatacaaggtgcaagaatcagtgactattttc actaagccaggctgtccttattgcgccaaggcgaaacaagcgctgattgatgccggtcta cagtatgaagagctgattttaggtaaagacgctaccacagtgagtctgcgcgccgtttct ggccgtaccacggtgccgcaagtgtttatcggtggtaaacacattggtggtagcgacgac ttagaagtctacctaaatcaataa |
| A2PVZ9 | UniRef cluster | -------------------------------------------KYIAPQYKVQESVTIFTK-----PGCPYCA-KAKQALIDAG---LQYEE----LILG---KDATTVSLRAVS----GRT----------TVPQVFIGGK--------HIGGSDD----LEVYLNQ-------------------------- | atgaggaacacaatgtttacatctaaagaaggtcaaaccattccacaggttacttttcct actcgccaaggtgacgcttgggtcaatgtgactaccgatgaactgttcaaaggcaaaacc gttatcgtgtttagcttgccgggtgcctttactccaacctgttcatccactcacctaccg cgctacaacgagctgtttcctgtctttaaagagcatggtgtcgacagcattctgtgcgta tcggtcaacgatactttcgtgatgaatgcttggaaagatgaccaaaacgccgacaacatc accttcattcctgatggtaatggtgaatttaccgatggtatgggcatgttggtggataaa aatgaccttggctttggtaaacgctcatggcgctacagcatgctggttaaagacggtgtg gtagaaaaaatgtttatcgaaccgaatgagccgggcgacccgttcaaagtatcggacgcc gataccatgctcaaatacattgcccctcaatacaaggtgcaagaatcagtgactattttc actaagccaggctgcccttattgcgccaaggcgaaacaagcgctgattgatgccggtcta cagtatgaagagctgattttaggtaaagacgccaccacagtgagcctgcgcgctgtttct ggccgtaccacggtgccgcaagtgtttatcggtggtaaacacattggtggcagcgacgac ttagaagtctacctaaatcaataa |
| A2PLL7 | UniRef cluster | -------------------------------------------KYIAPQYKVQESVTIFTK-----PGCPYCA-KAKQALIDAG---LQYEE----LILG---KDATTVSLRAVS----GRT----------TVPQVFIGGK--------HIGGSDD----LEVYLNQ-------------------------- | atgaggaacacaatgtttacatctaaagaaggtcaaaccattccacaggttacttttcct actcgccaaggtgacgcttgggtcaatgtgactagcgatgaactgttcaaaggcaaaacc gttatcgtgtttagcttgccgggtgcctttactccaacctgttcatccactcacctaccg cgctacaacgagctgtttcctgtctttaaagagcatggtgtcgacagcattctgtgcgta tcggtcaacgatactttcgtgatgaatgcttggaaagatgaccaaaatgccgacaacatc accttcattcctgatggtaatggtgaatttaccgatggtatgggcatgctggtggataaa aatgaccttggctttggtaaacgctcatggcgctacagcatgctggttaaagacggtgtg gtagaaaaaatgtttatcgaaccgaatgagccgggcgacccgttcaaagtatcggacgcc gataccatgctcaaatacattgcccctcaatacaaggtgcaagaatcagtgactattttc actaagccaggctgtccttattgcgccaaggcgaaacaagcgctgattgatgccggtcta cagtatgaagagctgattttaggtaaagacgctaccacagtgagtctgcgcgccgtttct ggccgtaccacggtgccgcaagtgtttatcggtggtaaacacattggtggtagcgacgac ttagaagtctacctaaatcaataa |
| A2P6Y2 | UniRef cluster | -------------------------------------------KYIAPQYKVQESVTIFTK-----PGCPYCA-KAKQALIDAG---LQYEE----LILG---KDATTVSLRAVS----GRT----------TVPQVFIGGK--------HIGGSDD----LEVYLNQ-------------------------- | atgaggaacacaatgtttacatctaaagaaggtcaaaccattccacaggttacttttcct actcgccaaggtgacgcttgggtcaatgtgactagcgatgagcttttcaaaggcaaaacc gttatcgtgtttagcttgccgggtgcctttactccaacctgttcatccactcacctaccg cgctacaacgagctgtttcctgtctttaaagagcatggtgtcgacagcattctgtgcgta tcggtcaacgatactttcgtgatgaatgcttggaaagatgaccaaaacgccgacaacatc acctttattcctgatggtaatggtgaatttaccgatggtatgggcatgctggtggataaa aatgaccttggctttggtaaacgctcatggcgctacagcatgctggttaaagatggtgtg gtagaaaaaatgtttatcgaaccgaatgagccgggcgacccgttcaaagtatcggacgcc gataccatgctcaaatacattgcccctcaatacaaggtgcaagaatcagtgactattttc actaagcctggatgtccttattgcgccaaggcgaaacaagcgctgattgatgccggtcta cagtatgaagagctgattttaggtaaagacgccaccacagtgagcctgcgcgctgtttct ggccgtaccacggtgccgcaagtgtttatcggtggtaaacacattggtggcagcgacgac ttagaagtctacctaaatcaataa |
| A1F753 | UniRef cluster | -------------------------------------------KYIAPQYKVQESVTIFTK-----PGCPYCA-KAKQALIDAG---LQYEE----LILG---KDATTVSLRAVS----GRT----------TVPQVFIGGK--------HIGGSDD----LEVYLNQ-------------------------- | atgaggaacacaatgtttacatctaaagaaggtcaaaccattccacaggttacttttcct actcgccaaggtgacgcttgggtcaatgtgactagcgatgaactgttcaaaggcaaaacc gttatcgtgtttagcttgccgggtgcctttactccaacctgttcatccactcacctaccg cgctacaacgagctgtttcctgtctttaaagagcatggtgtcgacagcattctgtgcgta tcggtcaacgatactttcgtgatgaatgcttggaaagatgaccaaaatgccgacaacatc accttcattcctgatggtaatggtgaatttaccgatggtatgggcatgctggtggataaa aatgaccttggctttggtaaacgctcatggcgctacagcatgctggttaaagacggtgtg gtagaaaaaatgtttatcgaaccgaatgagccgggcgacccgttcaaagtatcggacgcc gataccatgctcaaatacattgcccctcaatacaaggtgcaagaatcagtgactattttc actaagccaggctgtccttattgcgccaaggcgaaacaagcgctgattgatgccggtcta cagtatgaagagctgattttaggtaaagacgctaccacagtgagtctgcgcgccgtttct ggccgtaccacggtgccgcaagtgtttatcggtggtaaacacattggtggtagcgacgac ttagaagtctacctaaatcaataa |
| A1EMB6 | UniRef cluster | -------------------------------------------KYIAPQYKVQESVTIFTK-----PGCPYCA-KAKQALIDAG---LQYEE----LILG---KDATTVSLRAVS----GRT----------TVPQVFIGGK--------HIGGSDD----LEVYLNQ-------------------------- | atgaggaacacaatgtttacatctaaagaaggtcaaaccattccacaggttacttttcct actcgccaaggtgacgcttgggtcaatgtgactagcgatgaactgttcaaaggcaaaacc gttatcgtgtttagcttgccgggtgcctttactccaacctgttcatccactcacctaccg cgctacaacgagctgtttcctgtctttaaagagcatggtgtcgacagcattctgtgcgta tcggtcaacgatactttcgtgatgaatgcttggaaagatgaccaaaatgccgacaacatc accttcattcctgatggtaatggtgaatttaccgatggtatgggcatgctggtggataaa aatgaccttggctttggtaaacgctcatggcgctacagcatgctggttaaagatggtgtg gtagaaaaaatgtttatcgaaccgaatgagccgggcgacccgttcaaagtatcggacgcc gataccatgctcaaatacattgcccctcaatacaaggtgcaagaatcagtgactattttc actaagcctggatgtccttattgcgccaaggcgaaacaagcgctgattgatgccggtcta cagtatgaagagctgattttaggtaaagacgccaccacagtgagcctgcgcgctgtttct ggccgtaccacggtgccgcaagtgtttatcggtggtaaacacattggtggcagcgacgac ttagaagtctacctaaatcaataa |
| Q9KNU3 | UniRef cluster | -------------------------------------------KYIAPQYKVQESVTIFTK-----PGCPYCA-KAKQALIDAG---LQYEE----LILG---KDATTVSLRAVS----GRT----------TVPQVFIGGK--------HIGGSDD----LEVYLNQ-------------------------- | atgaggaacacaatgtttacatctaaagaaggtcaaaccattccacaggttacttttcct actcgccaaggtgacgcttgggtcaatgtgactagcgatgaactgttcaaaggcaaaacc gttatcgtgtttagcttgccgggtgcctttactccaacctgttcatccactcacctaccg cgctacaacgagctgtttcctgtctttaaagagcatggtgtcgacagcattctgtgcgta tcggtcaacgatactttcgtgatgaatgcttggaaagatgaccaaaatgccgacaacatc accttcattcctgatggtaatggtgaatttaccgatggtatgggcatgctggtggataaa aatgaccttggctttggtaaacgctcatggcgctacagcatgctggttaaagacggtgtg gtagaaaaaatgtttatcgaaccgaatgagccgggcgacccgttcaaagtatcggacgcc gataccatgctcaaatacattgcccctcaatacaaggtgcaagaatcagtgactattttc actaagccaggctgtccttattgcgccaaggcgaaacaagcgctgattgatgccggtcta cagtatgaagagctgattttaggtaaagacgctaccacagtgagtctgcgcgccgtttct ggccgtaccacggtgccgcaagtgtttatcggtggtaaacacattggtggtagcgacgac ttagaagtctacctaaatcaataa |
| A6Y1K9 | UniRef cluster | -------------------------------------------KYIAPQYKVQESVTIFTK-----PGCPYCA-KAKQALIDAG---LQYEE----LILG---KDATTVSLRAVS----GRT----------TVPQVFIGGK--------HIGGSDD----LEVYLNQ-------------------------- | atgaggaacacaatgtttacatctaaagaaggtcaaaccattccacaggttacttttcct actcgccaaggtgacgcttgggtcaatgtgactagcgatgagctgttcaaaggcaaaacc gttatcgtgtttagcttgccgggtgcctttactccaacctgttcatccactcacctaccg cgctacaacgagctgtttcctgtctttaaagagcatggtgtcgacagcattctgtgcgta tcggtcaacgatactttcgtgatgaatgcttggaaagatgaccaaaatgccgacaacatc acctttattcctgatggtaatggtgaatttaccgatggtatggggatgctggtggataaa aatgaccttggctttggtaaacgctcatggcgctacagcatgctggttaaagacggtgtg gtagaaaaaatgtttatcgaaccgaatgagccgggcgacccgttcaaagtatcggacgcc gataccatgctcaaatacattgcccctcaatacaaggtgcaagaatcagtgactattttc actaagcctggatgtccttattgcgccaaggcgaaacaagcgctgattgatgccggtcta cagtatgaagagctgattttaggtaaagacgccaccacagtgagcctgcgcgctgtttct ggccgtaccacggtgccgcaagtgtttatcggtggtaaacacattggcggcagcgacgac ttagaagtctacctaaatcaataa |
| A6XU96 | UniRef cluster | -------------------------------------------KYIAPQYKVQESVTIFTK-----PGCPYCA-KAKQALIDAG---LQYEE----LILG---KDATTVSLRAVS----GRT----------TVPQVFIGGK--------HIGGSDD----LEVYLNQ-------------------------- | atgaggaacacaatgtttacatctaaagaaggtcaaaccattccacaggttacttttcct actcgccaaggtgacgcttgggtcaatgtgactagcgatgaactgttcaaaggcaaaacc gttatcgtgtttagcttgccgggtgcctttactccaacctgttcatccactcacctaccg cgctacaacgagctgtttcctgtctttaaagagcatggtgtcgacagcattctgtgcgta tcggtcaacgatactttcgtgatgaatgcttggaaagatgaccaaaatgccgacaacatc accttcattcctgatggtaatggtgaatttaccgatggtatgggcatgttggtggataaa aatgaccttggctttggtaaacgctcatggcgctacagcatgctggttaaagatggtgtg gtagaaaaaatgtttatcgaaccgaatgagccgggcgacccgttcaaagtatcggacgcc gataccatgctcaaatacattgcccctcaatacaaggtgcaagaatcagtgactattttc actaagcctggatgtccttattgcgccaaggcgaaacaagcgctgattgatgccggtcta cagtatgaagagctgattttaggtaaagacgccaccacagtgagcctgcgcgctgtttct ggccgtaccacggtgccgcaagtgtttatcggtggtaaacacattggtggcagcgacgac ttagaagtctacctaaatcaataa |
| A6AFP4 | UniRef cluster | -------------------------------------------KYIAPQYKVQESVTIFTK-----PGCPYCA-KAKQALIDAG---LQYEE----LILG---KDATTVSLRAVS----GRT----------TVPQVFIGGK--------HIGGSDD----LEVYLNQ-------------------------- | atgaggaacacaatgtttacatctaaagaaggtcaaaccattccacaggttacttttcct actcgccaaggtgacgcttgggtcaatgtgactagcgatgaactgttcaaaggcaaaacc gttatcgtgtttagcttgccgggtgcctttactccaacctgttcatccactcacctaccg cgctacaacgagctgtatcctgtctttaaagagaatggtgtcgacagcattctgtgcgta tcggtcaacgatactttcgtgatgaatgcttggaaagatgaccaaaatgccgacaacatc accttcattcctgatggtaatggtgaatttaccgatggtatgggcatgctggtggataaa aatgaccttggctttggtaaacgctcatggcgctacagcatgctggttaaagacggtgtg gtagaaaaaatgtttatcgaaccgaatgagccgggcgacccgttcaaagtatcggacgcc gataccatgctcaaatacattgcccctcaatacaaggtgcaagaatcagtgactattttc actaagccaggctgtccttattgcgccaaggcgaaacaagcgctgattgatgccggtcta cagtatgaagagctgattttaggtaaagacgccaccacagtgagcctgcgcgctgtttct ggccgtaccacggtgccgcaagtgtttatcggtggtaaacacattggtggcagcgacgac ttagaagtctacctaaatcaataa |
| A6A149 | UniRef cluster | -------------------------------------------KYIAPQYKVQESVTIFTK-----PGCPYCA-KAKQALIDAG---LQYEE----LILG---KDATTVSLRAVS----GRT----------TVPQVFIGGK--------HIGGSDD----LEVYLNQ-------------------------- | atgaggaacacaatgtttacatctaaagaaggtcaaaccattccacaggttacttttcct actcgccaaggtgacgcttgggtcaatgtgactagcgatgagctgttcaaaggcaaaacc gttatcgtgtttagcttgccgggtgcctttactccaacctgttcatccactcacctaccg cgctacaacgagctgtttcctgtctttaaagagcatggtgtcgacagcattctgtgcgta tcggtcaacgatactttcgtgatgaatgcttggaaagatgaccaaaacgccgacaacatc accttcattcctgatggtaatggtgaatttaccgatggtatgggcatgttggtggataaa aatgaccttggctttggtaaacgctcatggcgctacagcatgctggttaaagacggtgtg gtagaaaaaatgtttatcgaaccgaatgagccgggcgacccgttcaaagtatcggacgcc gataccatgctcaaatacattgcccctcaatacaaggtgcaagaatcagtgactattttc actaagcctggctgtccttattgcgccaaggcgaaacaagcgctgattgatgccggtcta cagtatgaagagctgattttaggtaaagacgccaccacagtgagcctgcgcgctgtttct ggccgtaccacggtgccgcaagtgtttatcggtggtaaacacattggtggcagcgacgac ttagaagtctacctaaatcaataa |
| A5F510 | UniRef cluster | -------------------------------------------KYIAPQYKVQESVTIFTK-----PGCPYCA-KAKQALIDAG---LQYEE----LILG---KDATTVSLRAVS----GRT----------TVPQVFIGGK--------HIGGSDD----LEVYLNQ-------------------------- | atgaggaacacaatgtttacatctaaagaaggtcaaaccattccacaggttacttttcct actcgccaaggtgacgcttgggtcaatgtgactagcgatgaactgttcaaaggcaaaacc gttatcgtgtttagcttgccgggtgcctttactccaacctgttcatccactcacctaccg cgctacaacgagctgtttcctgtctttaaagagcatggtgtcgacagcattctgtgcgta tcggtcaacgatactttcgtgatgaatgcttggaaagatgaccaaaatgccgacaacatc accttcattcctgatggtaatggtgaatttaccgatggtatgggcatgctggtggataaa aatgaccttggctttggtaaacgctcatggcgctacagcatgctggttaaagacggtgtg gtagaaaaaatgtttatcgaaccgaatgagccgggcgacccgttcaaagtatcggacgcc gataccatgctcaaatacattgcccctcaatacaaggtgcaagaatcagtgactattttc actaagccaggctgtccttattgcgccaaggcgaaacaagcgctgattgatgccggtcta cagtatgaagagctgattttaggtaaagacgctaccacagtgagtctgcgcgccgtttct ggccgtaccacggtgccgcaagtgtttatcggtggtaaacacattggtggtagcgacgac ttagaagtctacctaaatcaataa |
| A3GXC3 | UniRef cluster | -------------------------------------------KYIAPQYKVQESVTIFTK-----PGCPYCA-KAKQALIDAG---LQYEE----LILG---KDATTVSLRAVS----GRT----------TVPQVFIGGK--------HIGGSDD----LEVYLNQ-------------------------- | atgaggaacacaatgtttacatctaaagaaggtcaaaccattccacaggttacttttcct actcgccaaggtgacgcttgggtcaatgtgactagcgatgaactgttcaaaggcaaaacc gttatcgtgtttagcttgccgggtgcctttactccaacctgttcatccactcacctaccg cgctacaacgagctgtttcctgtctttaaagagcatggtgtcgacagcattctgtgcgta tcggtcaacgatactttcgtgatgaatgcttggaaagatgaccaaaatgccgacaacatc accttcattcctgatggtaatggtgaatttaccgatggtatgggcatgctggtggataaa aatgaccttggctttggtaaacgctcatggcgctacagcatgctggttaaagacggtgtg gtagaaaaaatgtttatcgaaccgaatgagccgggcgacccgttcaaagtatcggacgcc gataccatgctcaaatacattgcccctcaatacaaggtgcaagaatcagtgactattttc actaagccaggctgtccttattgcgccaaggcgaaacaagcgctgattgatgccggtcta cagtatgaagagctgattttaggtaaagacgctaccacagtgagtctgcgcgccgtttct ggccgtaccacggtgccgcaagtgtttatcggtggtaaacacattggtggtagcgacgac ttagaagtctacctaaatcaataa |
| A3GRD5 | UniRef cluster | -------------------------------------------KYIAPQYKVQESVTIFTK-----PGCPYCA-KAKQALIDAG---LQYEE----LILG---KDATTVSLRAVS----GRT----------TVPQVFIGGK--------HIGGSDD----LEVYLNQ-------------------------- | atgaggaacacaatgtttacatctaaagaaggtcaaaccattccacaggttacttttcct actcgccaaggtgacgcttgggtcaatgtgactagcgatgaactgttcaaaggcaaaacc gttatcgtgtttagcttgccgggtgcctttactccaacctgttcatccactcacctaccg cgctacaacgagctgtttcctgtctttaaagagcatggtgtcgacagcattctgtgcgta tcggtcaacgatactttcgtgatgaatgcttggaaagatgaccaaaatgccgacaacatc accttcattcctgatggtaatggtgaatttaccgatggtatgggcatgctggtggataaa aatgaccttggctttggtaaacgctcatggcgctacagcatgctggttaaagacggtgtg gtagaaaaaatgtttatcgaaccgaatgagccgggcgacccgttcaaagtatcggacgcc gataccatgctcaaatacattgcccctcaatacaaggtgcaagaatcagtgactattttc actaagccaggctgtccttattgcgccaaggcgaaacaagcgctgattgatgccggtcta cagtatgaagagctgattttaggtaaagacgctaccacagtgagtctgcgcgccgtttct ggccgtaccacggtgccgcaagtgtttatcggtggtaaacacattggtggtagcgacgac ttagaagtctacctaaatcaataa |
| A3EJ81 | UniRef cluster | -------------------------------------------KYIAPQYKVQESVTIFTK-----PGCPYCA-KAKQALIDAG---LQYEE----LILG---KDATTVSLRAVS----GRT----------TVPQVFIGGK--------HIGGSDD----LEVYLNQ-------------------------- | atgaggaacacaatgtttacatctaaagaaggtcaaaccattccacaggttacttttcct actcgccaaggtgacgcttgggtcaatgtgactagcgatgaactgttcaaaggcaaaacc gttatcgtgtttagcttgccgggtgcctttactccaacctgttcatccactcacctaccg cgctacaacgagctgtttcctgtctttaaagagcatggtgtcgacagcattctgtgcgta tcggtcaacgatactttcgtgatgaatgcttggaaagatgaccaaaatgccgacaacatc accttcattcctgatggtaatggtgaatttaccgatggtatgggcatgctggtggataaa aatgaccttggctttggtaaacgctcatggcgctacagcatgctggttaaagacggtgtg gtagaaaaaatgtttatcgaaccgaatgagccgggcgacccgttcaaagtatcggacgcc gataccatgctcaaatacattgcccctcaatacaaggtgcaagaatcagtgactattttc actaagcctggctgtccttattgcgccaaggcgaaacaagcgctgattgatgccggtcta cagtatgaagagctgattttaggtaaagacgccaccacagtgagcctgcgcgctgtttct ggccgtaccacggtgccgcaagtgtttatcggtggtaaacacattggtggcagcgacgac ttagaagtctacctaaatcaataa |
| A3EB97 | UniRef cluster | -------------------------------------------KYIAPQYKVQESVTIFTK-----PGCPYCA-KAKQALIDAG---LQYEE----LILG---KDATTVSLRAVS----GRT----------TVPQVFIGGK--------HIGGSDD----LEVYLNQ-------------------------- | atgaggaacacaatgtttacatctaaagaaggtcaaaccattccacaggttacttttcct actcgccaaggtgacgcttgggtcaatgtgactagcgatgaactgttcaaaggcaaaacc gttatcgtgtttagcttgccgggtgcctttactccaacctgttcatccactcacctaccg cgctacaacgagctgtttcctgtctttaaagagcatggtgtcgacagcattctgtgcgta tcggtcaacgatactttcgtgatgaatgcttggaaagatgaccaaaatgccgacaacatc accttcattcctgatggtaatggtgaatttaccgatggtatgggcatgctggtggataaa aatgaccttggctttggtaaacgctcatggcgctacagcatgctggttaaagacggtgtg gtagaaaaaatgtttatcgaaccgaatgagccgggcgacccgttcaaagtatcggacgcc gataccatgctcaaatacattgcccctcaatacaaggtgcaagaatcagtgactattttc actaagccaggctgtccttattgcgccaaggcgaaacaagcgctgattgatgccggtcta cagtatgaagagctgattttaggtaaagacgctaccacagtgagtctgcgcgccgtttct ggccgtaccacggtgccgcaagtgtttatcggtggtaaacacattggtggtagcgacgac ttagaagtctacctaaatcaataa |
| A2PVZ9 | UniRef cluster | -------------------------------------------KYIAPQYKVQESVTIFTK-----PGCPYCA-KAKQALIDAG---LQYEE----LILG---KDATTVSLRAVS----GRT----------TVPQVFIGGK--------HIGGSDD----LEVYLNQ-------------------------- | atgaggaacacaatgtttacatctaaagaaggtcaaaccattccacaggttacttttcct actcgccaaggtgacgcttgggtcaatgtgactaccgatgaactgttcaaaggcaaaacc gttatcgtgtttagcttgccgggtgcctttactccaacctgttcatccactcacctaccg cgctacaacgagctgtttcctgtctttaaagagcatggtgtcgacagcattctgtgcgta tcggtcaacgatactttcgtgatgaatgcttggaaagatgaccaaaacgccgacaacatc accttcattcctgatggtaatggtgaatttaccgatggtatgggcatgttggtggataaa aatgaccttggctttggtaaacgctcatggcgctacagcatgctggttaaagacggtgtg gtagaaaaaatgtttatcgaaccgaatgagccgggcgacccgttcaaagtatcggacgcc gataccatgctcaaatacattgcccctcaatacaaggtgcaagaatcagtgactattttc actaagccaggctgcccttattgcgccaaggcgaaacaagcgctgattgatgccggtcta cagtatgaagagctgattttaggtaaagacgccaccacagtgagcctgcgcgctgtttct ggccgtaccacggtgccgcaagtgtttatcggtggtaaacacattggtggcagcgacgac ttagaagtctacctaaatcaataa |
| A2PLL7 | UniRef cluster | -------------------------------------------KYIAPQYKVQESVTIFTK-----PGCPYCA-KAKQALIDAG---LQYEE----LILG---KDATTVSLRAVS----GRT----------TVPQVFIGGK--------HIGGSDD----LEVYLNQ-------------------------- | atgaggaacacaatgtttacatctaaagaaggtcaaaccattccacaggttacttttcct actcgccaaggtgacgcttgggtcaatgtgactagcgatgaactgttcaaaggcaaaacc gttatcgtgtttagcttgccgggtgcctttactccaacctgttcatccactcacctaccg cgctacaacgagctgtttcctgtctttaaagagcatggtgtcgacagcattctgtgcgta tcggtcaacgatactttcgtgatgaatgcttggaaagatgaccaaaatgccgacaacatc accttcattcctgatggtaatggtgaatttaccgatggtatgggcatgctggtggataaa aatgaccttggctttggtaaacgctcatggcgctacagcatgctggttaaagacggtgtg gtagaaaaaatgtttatcgaaccgaatgagccgggcgacccgttcaaagtatcggacgcc gataccatgctcaaatacattgcccctcaatacaaggtgcaagaatcagtgactattttc actaagccaggctgtccttattgcgccaaggcgaaacaagcgctgattgatgccggtcta cagtatgaagagctgattttaggtaaagacgctaccacagtgagtctgcgcgccgtttct ggccgtaccacggtgccgcaagtgtttatcggtggtaaacacattggtggtagcgacgac ttagaagtctacctaaatcaataa |
| A2P6Y2 | UniRef cluster | -------------------------------------------KYIAPQYKVQESVTIFTK-----PGCPYCA-KAKQALIDAG---LQYEE----LILG---KDATTVSLRAVS----GRT----------TVPQVFIGGK--------HIGGSDD----LEVYLNQ-------------------------- | atgaggaacacaatgtttacatctaaagaaggtcaaaccattccacaggttacttttcct actcgccaaggtgacgcttgggtcaatgtgactagcgatgagcttttcaaaggcaaaacc gttatcgtgtttagcttgccgggtgcctttactccaacctgttcatccactcacctaccg cgctacaacgagctgtttcctgtctttaaagagcatggtgtcgacagcattctgtgcgta tcggtcaacgatactttcgtgatgaatgcttggaaagatgaccaaaacgccgacaacatc acctttattcctgatggtaatggtgaatttaccgatggtatgggcatgctggtggataaa aatgaccttggctttggtaaacgctcatggcgctacagcatgctggttaaagatggtgtg gtagaaaaaatgtttatcgaaccgaatgagccgggcgacccgttcaaagtatcggacgcc gataccatgctcaaatacattgcccctcaatacaaggtgcaagaatcagtgactattttc actaagcctggatgtccttattgcgccaaggcgaaacaagcgctgattgatgccggtcta cagtatgaagagctgattttaggtaaagacgccaccacagtgagcctgcgcgctgtttct ggccgtaccacggtgccgcaagtgtttatcggtggtaaacacattggtggcagcgacgac ttagaagtctacctaaatcaataa |
| A1F753 | UniRef cluster | -------------------------------------------KYIAPQYKVQESVTIFTK-----PGCPYCA-KAKQALIDAG---LQYEE----LILG---KDATTVSLRAVS----GRT----------TVPQVFIGGK--------HIGGSDD----LEVYLNQ-------------------------- | atgaggaacacaatgtttacatctaaagaaggtcaaaccattccacaggttacttttcct actcgccaaggtgacgcttgggtcaatgtgactagcgatgaactgttcaaaggcaaaacc gttatcgtgtttagcttgccgggtgcctttactccaacctgttcatccactcacctaccg cgctacaacgagctgtttcctgtctttaaagagcatggtgtcgacagcattctgtgcgta tcggtcaacgatactttcgtgatgaatgcttggaaagatgaccaaaatgccgacaacatc accttcattcctgatggtaatggtgaatttaccgatggtatgggcatgctggtggataaa aatgaccttggctttggtaaacgctcatggcgctacagcatgctggttaaagacggtgtg gtagaaaaaatgtttatcgaaccgaatgagccgggcgacccgttcaaagtatcggacgcc gataccatgctcaaatacattgcccctcaatacaaggtgcaagaatcagtgactattttc actaagccaggctgtccttattgcgccaaggcgaaacaagcgctgattgatgccggtcta cagtatgaagagctgattttaggtaaagacgctaccacagtgagtctgcgcgccgtttct ggccgtaccacggtgccgcaagtgtttatcggtggtaaacacattggtggtagcgacgac ttagaagtctacctaaatcaataa |
| A1EMB6 | UniRef cluster | -------------------------------------------KYIAPQYKVQESVTIFTK-----PGCPYCA-KAKQALIDAG---LQYEE----LILG---KDATTVSLRAVS----GRT----------TVPQVFIGGK--------HIGGSDD----LEVYLNQ-------------------------- | atgaggaacacaatgtttacatctaaagaaggtcaaaccattccacaggttacttttcct actcgccaaggtgacgcttgggtcaatgtgactagcgatgaactgttcaaaggcaaaacc gttatcgtgtttagcttgccgggtgcctttactccaacctgttcatccactcacctaccg cgctacaacgagctgtttcctgtctttaaagagcatggtgtcgacagcattctgtgcgta tcggtcaacgatactttcgtgatgaatgcttggaaagatgaccaaaatgccgacaacatc accttcattcctgatggtaatggtgaatttaccgatggtatgggcatgctggtggataaa aatgaccttggctttggtaaacgctcatggcgctacagcatgctggttaaagatggtgtg gtagaaaaaatgtttatcgaaccgaatgagccgggcgacccgttcaaagtatcggacgcc gataccatgctcaaatacattgcccctcaatacaaggtgcaagaatcagtgactattttc actaagcctggatgtccttattgcgccaaggcgaaacaagcgctgattgatgccggtcta cagtatgaagagctgattttaggtaaagacgccaccacagtgagcctgcgcgctgtttct ggccgtaccacggtgccgcaagtgtttatcggtggtaaacacattggtggcagcgacgac ttagaagtctacctaaatcaataa |
| Q9KNU3 | UniRef cluster | -------------------------------------------KYIAPQYKVQESVTIFTK-----PGCPYCA-KAKQALIDAG---LQYEE----LILG---KDATTVSLRAVS----GRT----------TVPQVFIGGK--------HIGGSDD----LEVYLNQ-------------------------- | atgaggaacacaatgtttacatctaaagaaggtcaaaccattccacaggttacttttcct actcgccaaggtgacgcttgggtcaatgtgactagcgatgaactgttcaaaggcaaaacc gttatcgtgtttagcttgccgggtgcctttactccaacctgttcatccactcacctaccg cgctacaacgagctgtttcctgtctttaaagagcatggtgtcgacagcattctgtgcgta tcggtcaacgatactttcgtgatgaatgcttggaaagatgaccaaaatgccgacaacatc accttcattcctgatggtaatggtgaatttaccgatggtatgggcatgctggtggataaa aatgaccttggctttggtaaacgctcatggcgctacagcatgctggttaaagacggtgtg gtagaaaaaatgtttatcgaaccgaatgagccgggcgacccgttcaaagtatcggacgcc gataccatgctcaaatacattgcccctcaatacaaggtgcaagaatcagtgactattttc actaagccaggctgtccttattgcgccaaggcgaaacaagcgctgattgatgccggtcta cagtatgaagagctgattttaggtaaagacgctaccacagtgagtctgcgcgccgtttct ggccgtaccacggtgccgcaagtgtttatcggtggtaaacacattggtggtagcgacgac ttagaagtctacctaaatcaataa |
| A6Y1K9 | UniRef cluster | -------------------------------------------KYIAPQYKVQESVTIFTK-----PGCPYCA-KAKQALIDAG---LQYEE----LILG---KDATTVSLRAVS----GRT----------TVPQVFIGGK--------HIGGSDD----LEVYLNQ-------------------------- | atgaggaacacaatgtttacatctaaagaaggtcaaaccattccacaggttacttttcct actcgccaaggtgacgcttgggtcaatgtgactagcgatgagctgttcaaaggcaaaacc gttatcgtgtttagcttgccgggtgcctttactccaacctgttcatccactcacctaccg cgctacaacgagctgtttcctgtctttaaagagcatggtgtcgacagcattctgtgcgta tcggtcaacgatactttcgtgatgaatgcttggaaagatgaccaaaatgccgacaacatc acctttattcctgatggtaatggtgaatttaccgatggtatggggatgctggtggataaa aatgaccttggctttggtaaacgctcatggcgctacagcatgctggttaaagacggtgtg gtagaaaaaatgtttatcgaaccgaatgagccgggcgacccgttcaaagtatcggacgcc gataccatgctcaaatacattgcccctcaatacaaggtgcaagaatcagtgactattttc actaagcctggatgtccttattgcgccaaggcgaaacaagcgctgattgatgccggtcta cagtatgaagagctgattttaggtaaagacgccaccacagtgagcctgcgcgctgtttct ggccgtaccacggtgccgcaagtgtttatcggtggtaaacacattggcggcagcgacgac ttagaagtctacctaaatcaataa |
| A6XU96 | UniRef cluster | -------------------------------------------KYIAPQYKVQESVTIFTK-----PGCPYCA-KAKQALIDAG---LQYEE----LILG---KDATTVSLRAVS----GRT----------TVPQVFIGGK--------HIGGSDD----LEVYLNQ-------------------------- | atgaggaacacaatgtttacatctaaagaaggtcaaaccattccacaggttacttttcct actcgccaaggtgacgcttgggtcaatgtgactagcgatgaactgttcaaaggcaaaacc gttatcgtgtttagcttgccgggtgcctttactccaacctgttcatccactcacctaccg cgctacaacgagctgtttcctgtctttaaagagcatggtgtcgacagcattctgtgcgta tcggtcaacgatactttcgtgatgaatgcttggaaagatgaccaaaatgccgacaacatc accttcattcctgatggtaatggtgaatttaccgatggtatgggcatgttggtggataaa aatgaccttggctttggtaaacgctcatggcgctacagcatgctggttaaagatggtgtg gtagaaaaaatgtttatcgaaccgaatgagccgggcgacccgttcaaagtatcggacgcc gataccatgctcaaatacattgcccctcaatacaaggtgcaagaatcagtgactattttc actaagcctggatgtccttattgcgccaaggcgaaacaagcgctgattgatgccggtcta cagtatgaagagctgattttaggtaaagacgccaccacagtgagcctgcgcgctgtttct ggccgtaccacggtgccgcaagtgtttatcggtggtaaacacattggtggcagcgacgac ttagaagtctacctaaatcaataa |
| A6AFP4 | UniRef cluster | -------------------------------------------KYIAPQYKVQESVTIFTK-----PGCPYCA-KAKQALIDAG---LQYEE----LILG---KDATTVSLRAVS----GRT----------TVPQVFIGGK--------HIGGSDD----LEVYLNQ-------------------------- | atgaggaacacaatgtttacatctaaagaaggtcaaaccattccacaggttacttttcct actcgccaaggtgacgcttgggtcaatgtgactagcgatgaactgttcaaaggcaaaacc gttatcgtgtttagcttgccgggtgcctttactccaacctgttcatccactcacctaccg cgctacaacgagctgtatcctgtctttaaagagaatggtgtcgacagcattctgtgcgta tcggtcaacgatactttcgtgatgaatgcttggaaagatgaccaaaatgccgacaacatc accttcattcctgatggtaatggtgaatttaccgatggtatgggcatgctggtggataaa aatgaccttggctttggtaaacgctcatggcgctacagcatgctggttaaagacggtgtg gtagaaaaaatgtttatcgaaccgaatgagccgggcgacccgttcaaagtatcggacgcc gataccatgctcaaatacattgcccctcaatacaaggtgcaagaatcagtgactattttc actaagccaggctgtccttattgcgccaaggcgaaacaagcgctgattgatgccggtcta cagtatgaagagctgattttaggtaaagacgccaccacagtgagcctgcgcgctgtttct ggccgtaccacggtgccgcaagtgtttatcggtggtaaacacattggtggcagcgacgac ttagaagtctacctaaatcaataa |
| A6A149 | UniRef cluster | -------------------------------------------KYIAPQYKVQESVTIFTK-----PGCPYCA-KAKQALIDAG---LQYEE----LILG---KDATTVSLRAVS----GRT----------TVPQVFIGGK--------HIGGSDD----LEVYLNQ-------------------------- | atgaggaacacaatgtttacatctaaagaaggtcaaaccattccacaggttacttttcct actcgccaaggtgacgcttgggtcaatgtgactagcgatgagctgttcaaaggcaaaacc gttatcgtgtttagcttgccgggtgcctttactccaacctgttcatccactcacctaccg cgctacaacgagctgtttcctgtctttaaagagcatggtgtcgacagcattctgtgcgta tcggtcaacgatactttcgtgatgaatgcttggaaagatgaccaaaacgccgacaacatc accttcattcctgatggtaatggtgaatttaccgatggtatgggcatgttggtggataaa aatgaccttggctttggtaaacgctcatggcgctacagcatgctggttaaagacggtgtg gtagaaaaaatgtttatcgaaccgaatgagccgggcgacccgttcaaagtatcggacgcc gataccatgctcaaatacattgcccctcaatacaaggtgcaagaatcagtgactattttc actaagcctggctgtccttattgcgccaaggcgaaacaagcgctgattgatgccggtcta cagtatgaagagctgattttaggtaaagacgccaccacagtgagcctgcgcgctgtttct ggccgtaccacggtgccgcaagtgtttatcggtggtaaacacattggtggcagcgacgac ttagaagtctacctaaatcaataa |
| A5F510 | UniRef cluster | -------------------------------------------KYIAPQYKVQESVTIFTK-----PGCPYCA-KAKQALIDAG---LQYEE----LILG---KDATTVSLRAVS----GRT----------TVPQVFIGGK--------HIGGSDD----LEVYLNQ-------------------------- | atgaggaacacaatgtttacatctaaagaaggtcaaaccattccacaggttacttttcct actcgccaaggtgacgcttgggtcaatgtgactagcgatgaactgttcaaaggcaaaacc gttatcgtgtttagcttgccgggtgcctttactccaacctgttcatccactcacctaccg cgctacaacgagctgtttcctgtctttaaagagcatggtgtcgacagcattctgtgcgta tcggtcaacgatactttcgtgatgaatgcttggaaagatgaccaaaatgccgacaacatc accttcattcctgatggtaatggtgaatttaccgatggtatgggcatgctggtggataaa aatgaccttggctttggtaaacgctcatggcgctacagcatgctggttaaagacggtgtg gtagaaaaaatgtttatcgaaccgaatgagccgggcgacccgttcaaagtatcggacgcc gataccatgctcaaatacattgcccctcaatacaaggtgcaagaatcagtgactattttc actaagccaggctgtccttattgcgccaaggcgaaacaagcgctgattgatgccggtcta cagtatgaagagctgattttaggtaaagacgctaccacagtgagtctgcgcgccgtttct ggccgtaccacggtgccgcaagtgtttatcggtggtaaacacattggtggtagcgacgac ttagaagtctacctaaatcaataa |
| A3GXC3 | UniRef cluster | -------------------------------------------KYIAPQYKVQESVTIFTK-----PGCPYCA-KAKQALIDAG---LQYEE----LILG---KDATTVSLRAVS----GRT----------TVPQVFIGGK--------HIGGSDD----LEVYLNQ-------------------------- | atgaggaacacaatgtttacatctaaagaaggtcaaaccattccacaggttacttttcct actcgccaaggtgacgcttgggtcaatgtgactagcgatgaactgttcaaaggcaaaacc gttatcgtgtttagcttgccgggtgcctttactccaacctgttcatccactcacctaccg cgctacaacgagctgtttcctgtctttaaagagcatggtgtcgacagcattctgtgcgta tcggtcaacgatactttcgtgatgaatgcttggaaagatgaccaaaatgccgacaacatc accttcattcctgatggtaatggtgaatttaccgatggtatgggcatgctggtggataaa aatgaccttggctttggtaaacgctcatggcgctacagcatgctggttaaagacggtgtg gtagaaaaaatgtttatcgaaccgaatgagccgggcgacccgttcaaagtatcggacgcc gataccatgctcaaatacattgcccctcaatacaaggtgcaagaatcagtgactattttc actaagccaggctgtccttattgcgccaaggcgaaacaagcgctgattgatgccggtcta cagtatgaagagctgattttaggtaaagacgctaccacagtgagtctgcgcgccgtttct ggccgtaccacggtgccgcaagtgtttatcggtggtaaacacattggtggtagcgacgac ttagaagtctacctaaatcaataa |
| A3GRD5 | UniRef cluster | -------------------------------------------KYIAPQYKVQESVTIFTK-----PGCPYCA-KAKQALIDAG---LQYEE----LILG---KDATTVSLRAVS----GRT----------TVPQVFIGGK--------HIGGSDD----LEVYLNQ-------------------------- | atgaggaacacaatgtttacatctaaagaaggtcaaaccattccacaggttacttttcct actcgccaaggtgacgcttgggtcaatgtgactagcgatgaactgttcaaaggcaaaacc gttatcgtgtttagcttgccgggtgcctttactccaacctgttcatccactcacctaccg cgctacaacgagctgtttcctgtctttaaagagcatggtgtcgacagcattctgtgcgta tcggtcaacgatactttcgtgatgaatgcttggaaagatgaccaaaatgccgacaacatc accttcattcctgatggtaatggtgaatttaccgatggtatgggcatgctggtggataaa aatgaccttggctttggtaaacgctcatggcgctacagcatgctggttaaagacggtgtg gtagaaaaaatgtttatcgaaccgaatgagccgggcgacccgttcaaagtatcggacgcc gataccatgctcaaatacattgcccctcaatacaaggtgcaagaatcagtgactattttc actaagccaggctgtccttattgcgccaaggcgaaacaagcgctgattgatgccggtcta cagtatgaagagctgattttaggtaaagacgctaccacagtgagtctgcgcgccgtttct ggccgtaccacggtgccgcaagtgtttatcggtggtaaacacattggtggtagcgacgac ttagaagtctacctaaatcaataa |
| A3EJ81 | UniRef cluster | -------------------------------------------KYIAPQYKVQESVTIFTK-----PGCPYCA-KAKQALIDAG---LQYEE----LILG---KDATTVSLRAVS----GRT----------TVPQVFIGGK--------HIGGSDD----LEVYLNQ-------------------------- | atgaggaacacaatgtttacatctaaagaaggtcaaaccattccacaggttacttttcct actcgccaaggtgacgcttgggtcaatgtgactagcgatgaactgttcaaaggcaaaacc gttatcgtgtttagcttgccgggtgcctttactccaacctgttcatccactcacctaccg cgctacaacgagctgtttcctgtctttaaagagcatggtgtcgacagcattctgtgcgta tcggtcaacgatactttcgtgatgaatgcttggaaagatgaccaaaatgccgacaacatc accttcattcctgatggtaatggtgaatttaccgatggtatgggcatgctggtggataaa aatgaccttggctttggtaaacgctcatggcgctacagcatgctggttaaagacggtgtg gtagaaaaaatgtttatcgaaccgaatgagccgggcgacccgttcaaagtatcggacgcc gataccatgctcaaatacattgcccctcaatacaaggtgcaagaatcagtgactattttc actaagcctggctgtccttattgcgccaaggcgaaacaagcgctgattgatgccggtcta cagtatgaagagctgattttaggtaaagacgccaccacagtgagcctgcgcgctgtttct ggccgtaccacggtgccgcaagtgtttatcggtggtaaacacattggtggcagcgacgac ttagaagtctacctaaatcaataa |
| A3EB97 | UniRef cluster | -------------------------------------------KYIAPQYKVQESVTIFTK-----PGCPYCA-KAKQALIDAG---LQYEE----LILG---KDATTVSLRAVS----GRT----------TVPQVFIGGK--------HIGGSDD----LEVYLNQ-------------------------- | atgaggaacacaatgtttacatctaaagaaggtcaaaccattccacaggttacttttcct actcgccaaggtgacgcttgggtcaatgtgactagcgatgaactgttcaaaggcaaaacc gttatcgtgtttagcttgccgggtgcctttactccaacctgttcatccactcacctaccg cgctacaacgagctgtttcctgtctttaaagagcatggtgtcgacagcattctgtgcgta tcggtcaacgatactttcgtgatgaatgcttggaaagatgaccaaaatgccgacaacatc accttcattcctgatggtaatggtgaatttaccgatggtatgggcatgctggtggataaa aatgaccttggctttggtaaacgctcatggcgctacagcatgctggttaaagacggtgtg gtagaaaaaatgtttatcgaaccgaatgagccgggcgacccgttcaaagtatcggacgcc gataccatgctcaaatacattgcccctcaatacaaggtgcaagaatcagtgactattttc actaagccaggctgtccttattgcgccaaggcgaaacaagcgctgattgatgccggtcta cagtatgaagagctgattttaggtaaagacgctaccacagtgagtctgcgcgccgtttct ggccgtaccacggtgccgcaagtgtttatcggtggtaaacacattggtggtagcgacgac ttagaagtctacctaaatcaataa |
| A2PVZ9 | UniRef cluster | -------------------------------------------KYIAPQYKVQESVTIFTK-----PGCPYCA-KAKQALIDAG---LQYEE----LILG---KDATTVSLRAVS----GRT----------TVPQVFIGGK--------HIGGSDD----LEVYLNQ-------------------------- | atgaggaacacaatgtttacatctaaagaaggtcaaaccattccacaggttacttttcct actcgccaaggtgacgcttgggtcaatgtgactaccgatgaactgttcaaaggcaaaacc gttatcgtgtttagcttgccgggtgcctttactccaacctgttcatccactcacctaccg cgctacaacgagctgtttcctgtctttaaagagcatggtgtcgacagcattctgtgcgta tcggtcaacgatactttcgtgatgaatgcttggaaagatgaccaaaacgccgacaacatc accttcattcctgatggtaatggtgaatttaccgatggtatgggcatgttggtggataaa aatgaccttggctttggtaaacgctcatggcgctacagcatgctggttaaagacggtgtg gtagaaaaaatgtttatcgaaccgaatgagccgggcgacccgttcaaagtatcggacgcc gataccatgctcaaatacattgcccctcaatacaaggtgcaagaatcagtgactattttc actaagccaggctgcccttattgcgccaaggcgaaacaagcgctgattgatgccggtcta cagtatgaagagctgattttaggtaaagacgccaccacagtgagcctgcgcgctgtttct ggccgtaccacggtgccgcaagtgtttatcggtggtaaacacattggtggcagcgacgac ttagaagtctacctaaatcaataa |
| A2PLL7 | UniRef cluster | -------------------------------------------KYIAPQYKVQESVTIFTK-----PGCPYCA-KAKQALIDAG---LQYEE----LILG---KDATTVSLRAVS----GRT----------TVPQVFIGGK--------HIGGSDD----LEVYLNQ-------------------------- | atgaggaacacaatgtttacatctaaagaaggtcaaaccattccacaggttacttttcct actcgccaaggtgacgcttgggtcaatgtgactagcgatgaactgttcaaaggcaaaacc gttatcgtgtttagcttgccgggtgcctttactccaacctgttcatccactcacctaccg cgctacaacgagctgtttcctgtctttaaagagcatggtgtcgacagcattctgtgcgta tcggtcaacgatactttcgtgatgaatgcttggaaagatgaccaaaatgccgacaacatc accttcattcctgatggtaatggtgaatttaccgatggtatgggcatgctggtggataaa aatgaccttggctttggtaaacgctcatggcgctacagcatgctggttaaagacggtgtg gtagaaaaaatgtttatcgaaccgaatgagccgggcgacccgttcaaagtatcggacgcc gataccatgctcaaatacattgcccctcaatacaaggtgcaagaatcagtgactattttc actaagccaggctgtccttattgcgccaaggcgaaacaagcgctgattgatgccggtcta cagtatgaagagctgattttaggtaaagacgctaccacagtgagtctgcgcgccgtttct ggccgtaccacggtgccgcaagtgtttatcggtggtaaacacattggtggtagcgacgac ttagaagtctacctaaatcaataa |
| A2P6Y2 | UniRef cluster | -------------------------------------------KYIAPQYKVQESVTIFTK-----PGCPYCA-KAKQALIDAG---LQYEE----LILG---KDATTVSLRAVS----GRT----------TVPQVFIGGK--------HIGGSDD----LEVYLNQ-------------------------- | atgaggaacacaatgtttacatctaaagaaggtcaaaccattccacaggttacttttcct actcgccaaggtgacgcttgggtcaatgtgactagcgatgagcttttcaaaggcaaaacc gttatcgtgtttagcttgccgggtgcctttactccaacctgttcatccactcacctaccg cgctacaacgagctgtttcctgtctttaaagagcatggtgtcgacagcattctgtgcgta tcggtcaacgatactttcgtgatgaatgcttggaaagatgaccaaaacgccgacaacatc acctttattcctgatggtaatggtgaatttaccgatggtatgggcatgctggtggataaa aatgaccttggctttggtaaacgctcatggcgctacagcatgctggttaaagatggtgtg gtagaaaaaatgtttatcgaaccgaatgagccgggcgacccgttcaaagtatcggacgcc gataccatgctcaaatacattgcccctcaatacaaggtgcaagaatcagtgactattttc actaagcctggatgtccttattgcgccaaggcgaaacaagcgctgattgatgccggtcta cagtatgaagagctgattttaggtaaagacgccaccacagtgagcctgcgcgctgtttct ggccgtaccacggtgccgcaagtgtttatcggtggtaaacacattggtggcagcgacgac ttagaagtctacctaaatcaataa |
| A1F753 | UniRef cluster | -------------------------------------------KYIAPQYKVQESVTIFTK-----PGCPYCA-KAKQALIDAG---LQYEE----LILG---KDATTVSLRAVS----GRT----------TVPQVFIGGK--------HIGGSDD----LEVYLNQ-------------------------- | atgaggaacacaatgtttacatctaaagaaggtcaaaccattccacaggttacttttcct actcgccaaggtgacgcttgggtcaatgtgactagcgatgaactgttcaaaggcaaaacc gttatcgtgtttagcttgccgggtgcctttactccaacctgttcatccactcacctaccg cgctacaacgagctgtttcctgtctttaaagagcatggtgtcgacagcattctgtgcgta tcggtcaacgatactttcgtgatgaatgcttggaaagatgaccaaaatgccgacaacatc accttcattcctgatggtaatggtgaatttaccgatggtatgggcatgctggtggataaa aatgaccttggctttggtaaacgctcatggcgctacagcatgctggttaaagacggtgtg gtagaaaaaatgtttatcgaaccgaatgagccgggcgacccgttcaaagtatcggacgcc gataccatgctcaaatacattgcccctcaatacaaggtgcaagaatcagtgactattttc actaagccaggctgtccttattgcgccaaggcgaaacaagcgctgattgatgccggtcta cagtatgaagagctgattttaggtaaagacgctaccacagtgagtctgcgcgccgtttct ggccgtaccacggtgccgcaagtgtttatcggtggtaaacacattggtggtagcgacgac ttagaagtctacctaaatcaataa |
| A1EMB6 | UniRef cluster | -------------------------------------------KYIAPQYKVQESVTIFTK-----PGCPYCA-KAKQALIDAG---LQYEE----LILG---KDATTVSLRAVS----GRT----------TVPQVFIGGK--------HIGGSDD----LEVYLNQ-------------------------- | atgaggaacacaatgtttacatctaaagaaggtcaaaccattccacaggttacttttcct actcgccaaggtgacgcttgggtcaatgtgactagcgatgaactgttcaaaggcaaaacc gttatcgtgtttagcttgccgggtgcctttactccaacctgttcatccactcacctaccg cgctacaacgagctgtttcctgtctttaaagagcatggtgtcgacagcattctgtgcgta tcggtcaacgatactttcgtgatgaatgcttggaaagatgaccaaaatgccgacaacatc accttcattcctgatggtaatggtgaatttaccgatggtatgggcatgctggtggataaa aatgaccttggctttggtaaacgctcatggcgctacagcatgctggttaaagatggtgtg gtagaaaaaatgtttatcgaaccgaatgagccgggcgacccgttcaaagtatcggacgcc gataccatgctcaaatacattgcccctcaatacaaggtgcaagaatcagtgactattttc actaagcctggatgtccttattgcgccaaggcgaaacaagcgctgattgatgccggtcta cagtatgaagagctgattttaggtaaagacgccaccacagtgagcctgcgcgctgtttct ggccgtaccacggtgccgcaagtgtttatcggtggtaaacacattggtggcagcgacgac ttagaagtctacctaaatcaataa |
| Q9KNU3 | UniRef cluster | -------------------------------------------KYIAPQYKVQESVTIFTK-----PGCPYCA-KAKQALIDAG---LQYEE----LILG---KDATTVSLRAVS----GRT----------TVPQVFIGGK--------HIGGSDD----LEVYLNQ-------------------------- | atgaggaacacaatgtttacatctaaagaaggtcaaaccattccacaggttacttttcct actcgccaaggtgacgcttgggtcaatgtgactagcgatgaactgttcaaaggcaaaacc gttatcgtgtttagcttgccgggtgcctttactccaacctgttcatccactcacctaccg cgctacaacgagctgtttcctgtctttaaagagcatggtgtcgacagcattctgtgcgta tcggtcaacgatactttcgtgatgaatgcttggaaagatgaccaaaatgccgacaacatc accttcattcctgatggtaatggtgaatttaccgatggtatgggcatgctggtggataaa aatgaccttggctttggtaaacgctcatggcgctacagcatgctggttaaagacggtgtg gtagaaaaaatgtttatcgaaccgaatgagccgggcgacccgttcaaagtatcggacgcc gataccatgctcaaatacattgcccctcaatacaaggtgcaagaatcagtgactattttc actaagccaggctgtccttattgcgccaaggcgaaacaagcgctgattgatgccggtcta cagtatgaagagctgattttaggtaaagacgctaccacagtgagtctgcgcgccgtttct ggccgtaccacggtgccgcaagtgtttatcggtggtaaacacattggtggtagcgacgac ttagaagtctacctaaatcaataa |
| A6Y1K9 | UniRef cluster | -------------------------------------------KYIAPQYKVQESVTIFTK-----PGCPYCA-KAKQALIDAG---LQYEE----LILG---KDATTVSLRAVS----GRT----------TVPQVFIGGK--------HIGGSDD----LEVYLNQ-------------------------- | atgaggaacacaatgtttacatctaaagaaggtcaaaccattccacaggttacttttcct actcgccaaggtgacgcttgggtcaatgtgactagcgatgagctgttcaaaggcaaaacc gttatcgtgtttagcttgccgggtgcctttactccaacctgttcatccactcacctaccg cgctacaacgagctgtttcctgtctttaaagagcatggtgtcgacagcattctgtgcgta tcggtcaacgatactttcgtgatgaatgcttggaaagatgaccaaaatgccgacaacatc acctttattcctgatggtaatggtgaatttaccgatggtatggggatgctggtggataaa aatgaccttggctttggtaaacgctcatggcgctacagcatgctggttaaagacggtgtg gtagaaaaaatgtttatcgaaccgaatgagccgggcgacccgttcaaagtatcggacgcc gataccatgctcaaatacattgcccctcaatacaaggtgcaagaatcagtgactattttc actaagcctggatgtccttattgcgccaaggcgaaacaagcgctgattgatgccggtcta cagtatgaagagctgattttaggtaaagacgccaccacagtgagcctgcgcgctgtttct ggccgtaccacggtgccgcaagtgtttatcggtggtaaacacattggcggcagcgacgac ttagaagtctacctaaatcaataa |
| A6XU96 | UniRef cluster | -------------------------------------------KYIAPQYKVQESVTIFTK-----PGCPYCA-KAKQALIDAG---LQYEE----LILG---KDATTVSLRAVS----GRT----------TVPQVFIGGK--------HIGGSDD----LEVYLNQ-------------------------- | atgaggaacacaatgtttacatctaaagaaggtcaaaccattccacaggttacttttcct actcgccaaggtgacgcttgggtcaatgtgactagcgatgaactgttcaaaggcaaaacc gttatcgtgtttagcttgccgggtgcctttactccaacctgttcatccactcacctaccg cgctacaacgagctgtttcctgtctttaaagagcatggtgtcgacagcattctgtgcgta tcggtcaacgatactttcgtgatgaatgcttggaaagatgaccaaaatgccgacaacatc accttcattcctgatggtaatggtgaatttaccgatggtatgggcatgttggtggataaa aatgaccttggctttggtaaacgctcatggcgctacagcatgctggttaaagatggtgtg gtagaaaaaatgtttatcgaaccgaatgagccgggcgacccgttcaaagtatcggacgcc gataccatgctcaaatacattgcccctcaatacaaggtgcaagaatcagtgactattttc actaagcctggatgtccttattgcgccaaggcgaaacaagcgctgattgatgccggtcta cagtatgaagagctgattttaggtaaagacgccaccacagtgagcctgcgcgctgtttct ggccgtaccacggtgccgcaagtgtttatcggtggtaaacacattggtggcagcgacgac ttagaagtctacctaaatcaataa |
| A6AFP4 | UniRef cluster | -------------------------------------------KYIAPQYKVQESVTIFTK-----PGCPYCA-KAKQALIDAG---LQYEE----LILG---KDATTVSLRAVS----GRT----------TVPQVFIGGK--------HIGGSDD----LEVYLNQ-------------------------- | atgaggaacacaatgtttacatctaaagaaggtcaaaccattccacaggttacttttcct actcgccaaggtgacgcttgggtcaatgtgactagcgatgaactgttcaaaggcaaaacc gttatcgtgtttagcttgccgggtgcctttactccaacctgttcatccactcacctaccg cgctacaacgagctgtatcctgtctttaaagagaatggtgtcgacagcattctgtgcgta tcggtcaacgatactttcgtgatgaatgcttggaaagatgaccaaaatgccgacaacatc accttcattcctgatggtaatggtgaatttaccgatggtatgggcatgctggtggataaa aatgaccttggctttggtaaacgctcatggcgctacagcatgctggttaaagacggtgtg gtagaaaaaatgtttatcgaaccgaatgagccgggcgacccgttcaaagtatcggacgcc gataccatgctcaaatacattgcccctcaatacaaggtgcaagaatcagtgactattttc actaagccaggctgtccttattgcgccaaggcgaaacaagcgctgattgatgccggtcta cagtatgaagagctgattttaggtaaagacgccaccacagtgagcctgcgcgctgtttct ggccgtaccacggtgccgcaagtgtttatcggtggtaaacacattggtggcagcgacgac ttagaagtctacctaaatcaataa |
| A6A149 | UniRef cluster | -------------------------------------------KYIAPQYKVQESVTIFTK-----PGCPYCA-KAKQALIDAG---LQYEE----LILG---KDATTVSLRAVS----GRT----------TVPQVFIGGK--------HIGGSDD----LEVYLNQ-------------------------- | atgaggaacacaatgtttacatctaaagaaggtcaaaccattccacaggttacttttcct actcgccaaggtgacgcttgggtcaatgtgactagcgatgagctgttcaaaggcaaaacc gttatcgtgtttagcttgccgggtgcctttactccaacctgttcatccactcacctaccg cgctacaacgagctgtttcctgtctttaaagagcatggtgtcgacagcattctgtgcgta tcggtcaacgatactttcgtgatgaatgcttggaaagatgaccaaaacgccgacaacatc accttcattcctgatggtaatggtgaatttaccgatggtatgggcatgttggtggataaa aatgaccttggctttggtaaacgctcatggcgctacagcatgctggttaaagacggtgtg gtagaaaaaatgtttatcgaaccgaatgagccgggcgacccgttcaaagtatcggacgcc gataccatgctcaaatacattgcccctcaatacaaggtgcaagaatcagtgactattttc actaagcctggctgtccttattgcgccaaggcgaaacaagcgctgattgatgccggtcta cagtatgaagagctgattttaggtaaagacgccaccacagtgagcctgcgcgctgtttct ggccgtaccacggtgccgcaagtgtttatcggtggtaaacacattggtggcagcgacgac ttagaagtctacctaaatcaataa |
| A5F510 | UniRef cluster | -------------------------------------------KYIAPQYKVQESVTIFTK-----PGCPYCA-KAKQALIDAG---LQYEE----LILG---KDATTVSLRAVS----GRT----------TVPQVFIGGK--------HIGGSDD----LEVYLNQ-------------------------- | atgaggaacacaatgtttacatctaaagaaggtcaaaccattccacaggttacttttcct actcgccaaggtgacgcttgggtcaatgtgactagcgatgaactgttcaaaggcaaaacc gttatcgtgtttagcttgccgggtgcctttactccaacctgttcatccactcacctaccg cgctacaacgagctgtttcctgtctttaaagagcatggtgtcgacagcattctgtgcgta tcggtcaacgatactttcgtgatgaatgcttggaaagatgaccaaaatgccgacaacatc accttcattcctgatggtaatggtgaatttaccgatggtatgggcatgctggtggataaa aatgaccttggctttggtaaacgctcatggcgctacagcatgctggttaaagacggtgtg gtagaaaaaatgtttatcgaaccgaatgagccgggcgacccgttcaaagtatcggacgcc gataccatgctcaaatacattgcccctcaatacaaggtgcaagaatcagtgactattttc actaagccaggctgtccttattgcgccaaggcgaaacaagcgctgattgatgccggtcta cagtatgaagagctgattttaggtaaagacgctaccacagtgagtctgcgcgccgtttct ggccgtaccacggtgccgcaagtgtttatcggtggtaaacacattggtggtagcgacgac ttagaagtctacctaaatcaataa |
| A3GXC3 | UniRef cluster | -------------------------------------------KYIAPQYKVQESVTIFTK-----PGCPYCA-KAKQALIDAG---LQYEE----LILG---KDATTVSLRAVS----GRT----------TVPQVFIGGK--------HIGGSDD----LEVYLNQ-------------------------- | atgaggaacacaatgtttacatctaaagaaggtcaaaccattccacaggttacttttcct actcgccaaggtgacgcttgggtcaatgtgactagcgatgaactgttcaaaggcaaaacc gttatcgtgtttagcttgccgggtgcctttactccaacctgttcatccactcacctaccg cgctacaacgagctgtttcctgtctttaaagagcatggtgtcgacagcattctgtgcgta tcggtcaacgatactttcgtgatgaatgcttggaaagatgaccaaaatgccgacaacatc accttcattcctgatggtaatggtgaatttaccgatggtatgggcatgctggtggataaa aatgaccttggctttggtaaacgctcatggcgctacagcatgctggttaaagacggtgtg gtagaaaaaatgtttatcgaaccgaatgagccgggcgacccgttcaaagtatcggacgcc gataccatgctcaaatacattgcccctcaatacaaggtgcaagaatcagtgactattttc actaagccaggctgtccttattgcgccaaggcgaaacaagcgctgattgatgccggtcta cagtatgaagagctgattttaggtaaagacgctaccacagtgagtctgcgcgccgtttct ggccgtaccacggtgccgcaagtgtttatcggtggtaaacacattggtggtagcgacgac ttagaagtctacctaaatcaataa |
| A3GRD5 | UniRef cluster | -------------------------------------------KYIAPQYKVQESVTIFTK-----PGCPYCA-KAKQALIDAG---LQYEE----LILG---KDATTVSLRAVS----GRT----------TVPQVFIGGK--------HIGGSDD----LEVYLNQ-------------------------- | atgaggaacacaatgtttacatctaaagaaggtcaaaccattccacaggttacttttcct actcgccaaggtgacgcttgggtcaatgtgactagcgatgaactgttcaaaggcaaaacc gttatcgtgtttagcttgccgggtgcctttactccaacctgttcatccactcacctaccg cgctacaacgagctgtttcctgtctttaaagagcatggtgtcgacagcattctgtgcgta tcggtcaacgatactttcgtgatgaatgcttggaaagatgaccaaaatgccgacaacatc accttcattcctgatggtaatggtgaatttaccgatggtatgggcatgctggtggataaa aatgaccttggctttggtaaacgctcatggcgctacagcatgctggttaaagacggtgtg gtagaaaaaatgtttatcgaaccgaatgagccgggcgacccgttcaaagtatcggacgcc gataccatgctcaaatacattgcccctcaatacaaggtgcaagaatcagtgactattttc actaagccaggctgtccttattgcgccaaggcgaaacaagcgctgattgatgccggtcta cagtatgaagagctgattttaggtaaagacgctaccacagtgagtctgcgcgccgtttct ggccgtaccacggtgccgcaagtgtttatcggtggtaaacacattggtggtagcgacgac ttagaagtctacctaaatcaataa |
| A3EJ81 | UniRef cluster | -------------------------------------------KYIAPQYKVQESVTIFTK-----PGCPYCA-KAKQALIDAG---LQYEE----LILG---KDATTVSLRAVS----GRT----------TVPQVFIGGK--------HIGGSDD----LEVYLNQ-------------------------- | atgaggaacacaatgtttacatctaaagaaggtcaaaccattccacaggttacttttcct actcgccaaggtgacgcttgggtcaatgtgactagcgatgaactgttcaaaggcaaaacc gttatcgtgtttagcttgccgggtgcctttactccaacctgttcatccactcacctaccg cgctacaacgagctgtttcctgtctttaaagagcatggtgtcgacagcattctgtgcgta tcggtcaacgatactttcgtgatgaatgcttggaaagatgaccaaaatgccgacaacatc accttcattcctgatggtaatggtgaatttaccgatggtatgggcatgctggtggataaa aatgaccttggctttggtaaacgctcatggcgctacagcatgctggttaaagacggtgtg gtagaaaaaatgtttatcgaaccgaatgagccgggcgacccgttcaaagtatcggacgcc gataccatgctcaaatacattgcccctcaatacaaggtgcaagaatcagtgactattttc actaagcctggctgtccttattgcgccaaggcgaaacaagcgctgattgatgccggtcta cagtatgaagagctgattttaggtaaagacgccaccacagtgagcctgcgcgctgtttct ggccgtaccacggtgccgcaagtgtttatcggtggtaaacacattggtggcagcgacgac ttagaagtctacctaaatcaataa |
| A3EB97 | UniRef cluster | -------------------------------------------KYIAPQYKVQESVTIFTK-----PGCPYCA-KAKQALIDAG---LQYEE----LILG---KDATTVSLRAVS----GRT----------TVPQVFIGGK--------HIGGSDD----LEVYLNQ-------------------------- | atgaggaacacaatgtttacatctaaagaaggtcaaaccattccacaggttacttttcct actcgccaaggtgacgcttgggtcaatgtgactagcgatgaactgttcaaaggcaaaacc gttatcgtgtttagcttgccgggtgcctttactccaacctgttcatccactcacctaccg cgctacaacgagctgtttcctgtctttaaagagcatggtgtcgacagcattctgtgcgta tcggtcaacgatactttcgtgatgaatgcttggaaagatgaccaaaatgccgacaacatc accttcattcctgatggtaatggtgaatttaccgatggtatgggcatgctggtggataaa aatgaccttggctttggtaaacgctcatggcgctacagcatgctggttaaagacggtgtg gtagaaaaaatgtttatcgaaccgaatgagccgggcgacccgttcaaagtatcggacgcc gataccatgctcaaatacattgcccctcaatacaaggtgcaagaatcagtgactattttc actaagccaggctgtccttattgcgccaaggcgaaacaagcgctgattgatgccggtcta cagtatgaagagctgattttaggtaaagacgctaccacagtgagtctgcgcgccgtttct ggccgtaccacggtgccgcaagtgtttatcggtggtaaacacattggtggtagcgacgac ttagaagtctacctaaatcaataa |
| A2PVZ9 | UniRef cluster | -------------------------------------------KYIAPQYKVQESVTIFTK-----PGCPYCA-KAKQALIDAG---LQYEE----LILG---KDATTVSLRAVS----GRT----------TVPQVFIGGK--------HIGGSDD----LEVYLNQ-------------------------- | atgaggaacacaatgtttacatctaaagaaggtcaaaccattccacaggttacttttcct actcgccaaggtgacgcttgggtcaatgtgactaccgatgaactgttcaaaggcaaaacc gttatcgtgtttagcttgccgggtgcctttactccaacctgttcatccactcacctaccg cgctacaacgagctgtttcctgtctttaaagagcatggtgtcgacagcattctgtgcgta tcggtcaacgatactttcgtgatgaatgcttggaaagatgaccaaaacgccgacaacatc accttcattcctgatggtaatggtgaatttaccgatggtatgggcatgttggtggataaa aatgaccttggctttggtaaacgctcatggcgctacagcatgctggttaaagacggtgtg gtagaaaaaatgtttatcgaaccgaatgagccgggcgacccgttcaaagtatcggacgcc gataccatgctcaaatacattgcccctcaatacaaggtgcaagaatcagtgactattttc actaagccaggctgcccttattgcgccaaggcgaaacaagcgctgattgatgccggtcta cagtatgaagagctgattttaggtaaagacgccaccacagtgagcctgcgcgctgtttct ggccgtaccacggtgccgcaagtgtttatcggtggtaaacacattggtggcagcgacgac ttagaagtctacctaaatcaataa |
| A2PLL7 | UniRef cluster | -------------------------------------------KYIAPQYKVQESVTIFTK-----PGCPYCA-KAKQALIDAG---LQYEE----LILG---KDATTVSLRAVS----GRT----------TVPQVFIGGK--------HIGGSDD----LEVYLNQ-------------------------- | atgaggaacacaatgtttacatctaaagaaggtcaaaccattccacaggttacttttcct actcgccaaggtgacgcttgggtcaatgtgactagcgatgaactgttcaaaggcaaaacc gttatcgtgtttagcttgccgggtgcctttactccaacctgttcatccactcacctaccg cgctacaacgagctgtttcctgtctttaaagagcatggtgtcgacagcattctgtgcgta tcggtcaacgatactttcgtgatgaatgcttggaaagatgaccaaaatgccgacaacatc accttcattcctgatggtaatggtgaatttaccgatggtatgggcatgctggtggataaa aatgaccttggctttggtaaacgctcatggcgctacagcatgctggttaaagacggtgtg gtagaaaaaatgtttatcgaaccgaatgagccgggcgacccgttcaaagtatcggacgcc gataccatgctcaaatacattgcccctcaatacaaggtgcaagaatcagtgactattttc actaagccaggctgtccttattgcgccaaggcgaaacaagcgctgattgatgccggtcta cagtatgaagagctgattttaggtaaagacgctaccacagtgagtctgcgcgccgtttct ggccgtaccacggtgccgcaagtgtttatcggtggtaaacacattggtggtagcgacgac ttagaagtctacctaaatcaataa |
| A2P6Y2 | UniRef cluster | -------------------------------------------KYIAPQYKVQESVTIFTK-----PGCPYCA-KAKQALIDAG---LQYEE----LILG---KDATTVSLRAVS----GRT----------TVPQVFIGGK--------HIGGSDD----LEVYLNQ-------------------------- | atgaggaacacaatgtttacatctaaagaaggtcaaaccattccacaggttacttttcct actcgccaaggtgacgcttgggtcaatgtgactagcgatgagcttttcaaaggcaaaacc gttatcgtgtttagcttgccgggtgcctttactccaacctgttcatccactcacctaccg cgctacaacgagctgtttcctgtctttaaagagcatggtgtcgacagcattctgtgcgta tcggtcaacgatactttcgtgatgaatgcttggaaagatgaccaaaacgccgacaacatc acctttattcctgatggtaatggtgaatttaccgatggtatgggcatgctggtggataaa aatgaccttggctttggtaaacgctcatggcgctacagcatgctggttaaagatggtgtg gtagaaaaaatgtttatcgaaccgaatgagccgggcgacccgttcaaagtatcggacgcc gataccatgctcaaatacattgcccctcaatacaaggtgcaagaatcagtgactattttc actaagcctggatgtccttattgcgccaaggcgaaacaagcgctgattgatgccggtcta cagtatgaagagctgattttaggtaaagacgccaccacagtgagcctgcgcgctgtttct ggccgtaccacggtgccgcaagtgtttatcggtggtaaacacattggtggcagcgacgac ttagaagtctacctaaatcaataa |
| A1F753 | UniRef cluster | -------------------------------------------KYIAPQYKVQESVTIFTK-----PGCPYCA-KAKQALIDAG---LQYEE----LILG---KDATTVSLRAVS----GRT----------TVPQVFIGGK--------HIGGSDD----LEVYLNQ-------------------------- | atgaggaacacaatgtttacatctaaagaaggtcaaaccattccacaggttacttttcct actcgccaaggtgacgcttgggtcaatgtgactagcgatgaactgttcaaaggcaaaacc gttatcgtgtttagcttgccgggtgcctttactccaacctgttcatccactcacctaccg cgctacaacgagctgtttcctgtctttaaagagcatggtgtcgacagcattctgtgcgta tcggtcaacgatactttcgtgatgaatgcttggaaagatgaccaaaatgccgacaacatc accttcattcctgatggtaatggtgaatttaccgatggtatgggcatgctggtggataaa aatgaccttggctttggtaaacgctcatggcgctacagcatgctggttaaagacggtgtg gtagaaaaaatgtttatcgaaccgaatgagccgggcgacccgttcaaagtatcggacgcc gataccatgctcaaatacattgcccctcaatacaaggtgcaagaatcagtgactattttc actaagccaggctgtccttattgcgccaaggcgaaacaagcgctgattgatgccggtcta cagtatgaagagctgattttaggtaaagacgctaccacagtgagtctgcgcgccgtttct ggccgtaccacggtgccgcaagtgtttatcggtggtaaacacattggtggtagcgacgac ttagaagtctacctaaatcaataa |
| A1EMB6 | UniRef cluster | -------------------------------------------KYIAPQYKVQESVTIFTK-----PGCPYCA-KAKQALIDAG---LQYEE----LILG---KDATTVSLRAVS----GRT----------TVPQVFIGGK--------HIGGSDD----LEVYLNQ-------------------------- | atgaggaacacaatgtttacatctaaagaaggtcaaaccattccacaggttacttttcct actcgccaaggtgacgcttgggtcaatgtgactagcgatgaactgttcaaaggcaaaacc gttatcgtgtttagcttgccgggtgcctttactccaacctgttcatccactcacctaccg cgctacaacgagctgtttcctgtctttaaagagcatggtgtcgacagcattctgtgcgta tcggtcaacgatactttcgtgatgaatgcttggaaagatgaccaaaatgccgacaacatc accttcattcctgatggtaatggtgaatttaccgatggtatgggcatgctggtggataaa aatgaccttggctttggtaaacgctcatggcgctacagcatgctggttaaagatggtgtg gtagaaaaaatgtttatcgaaccgaatgagccgggcgacccgttcaaagtatcggacgcc gataccatgctcaaatacattgcccctcaatacaaggtgcaagaatcagtgactattttc actaagcctggatgtccttattgcgccaaggcgaaacaagcgctgattgatgccggtcta cagtatgaagagctgattttaggtaaagacgccaccacagtgagcctgcgcgctgtttct ggccgtaccacggtgccgcaagtgtttatcggtggtaaacacattggtggcagcgacgac ttagaagtctacctaaatcaataa |
| Q9KNU3 | UniRef cluster | -------------------------------------------KYIAPQYKVQESVTIFTK-----PGCPYCA-KAKQALIDAG---LQYEE----LILG---KDATTVSLRAVS----GRT----------TVPQVFIGGK--------HIGGSDD----LEVYLNQ-------------------------- | atgaggaacacaatgtttacatctaaagaaggtcaaaccattccacaggttacttttcct actcgccaaggtgacgcttgggtcaatgtgactagcgatgaactgttcaaaggcaaaacc gttatcgtgtttagcttgccgggtgcctttactccaacctgttcatccactcacctaccg cgctacaacgagctgtttcctgtctttaaagagcatggtgtcgacagcattctgtgcgta tcggtcaacgatactttcgtgatgaatgcttggaaagatgaccaaaatgccgacaacatc accttcattcctgatggtaatggtgaatttaccgatggtatgggcatgctggtggataaa aatgaccttggctttggtaaacgctcatggcgctacagcatgctggttaaagacggtgtg gtagaaaaaatgtttatcgaaccgaatgagccgggcgacccgttcaaagtatcggacgcc gataccatgctcaaatacattgcccctcaatacaaggtgcaagaatcagtgactattttc actaagccaggctgtccttattgcgccaaggcgaaacaagcgctgattgatgccggtcta cagtatgaagagctgattttaggtaaagacgctaccacagtgagtctgcgcgccgtttct ggccgtaccacggtgccgcaagtgtttatcggtggtaaacacattggtggtagcgacgac ttagaagtctacctaaatcaataa |
| A6Y1K9 | UniRef cluster | -------------------------------------------KYIAPQYKVQESVTIFTK-----PGCPYCA-KAKQALIDAG---LQYEE----LILG---KDATTVSLRAVS----GRT----------TVPQVFIGGK--------HIGGSDD----LEVYLNQ-------------------------- | atgaggaacacaatgtttacatctaaagaaggtcaaaccattccacaggttacttttcct actcgccaaggtgacgcttgggtcaatgtgactagcgatgagctgttcaaaggcaaaacc gttatcgtgtttagcttgccgggtgcctttactccaacctgttcatccactcacctaccg cgctacaacgagctgtttcctgtctttaaagagcatggtgtcgacagcattctgtgcgta tcggtcaacgatactttcgtgatgaatgcttggaaagatgaccaaaatgccgacaacatc acctttattcctgatggtaatggtgaatttaccgatggtatggggatgctggtggataaa aatgaccttggctttggtaaacgctcatggcgctacagcatgctggttaaagacggtgtg gtagaaaaaatgtttatcgaaccgaatgagccgggcgacccgttcaaagtatcggacgcc gataccatgctcaaatacattgcccctcaatacaaggtgcaagaatcagtgactattttc actaagcctggatgtccttattgcgccaaggcgaaacaagcgctgattgatgccggtcta cagtatgaagagctgattttaggtaaagacgccaccacagtgagcctgcgcgctgtttct ggccgtaccacggtgccgcaagtgtttatcggtggtaaacacattggcggcagcgacgac ttagaagtctacctaaatcaataa |
| A6XU96 | UniRef cluster | -------------------------------------------KYIAPQYKVQESVTIFTK-----PGCPYCA-KAKQALIDAG---LQYEE----LILG---KDATTVSLRAVS----GRT----------TVPQVFIGGK--------HIGGSDD----LEVYLNQ-------------------------- | atgaggaacacaatgtttacatctaaagaaggtcaaaccattccacaggttacttttcct actcgccaaggtgacgcttgggtcaatgtgactagcgatgaactgttcaaaggcaaaacc gttatcgtgtttagcttgccgggtgcctttactccaacctgttcatccactcacctaccg cgctacaacgagctgtttcctgtctttaaagagcatggtgtcgacagcattctgtgcgta tcggtcaacgatactttcgtgatgaatgcttggaaagatgaccaaaatgccgacaacatc accttcattcctgatggtaatggtgaatttaccgatggtatgggcatgttggtggataaa aatgaccttggctttggtaaacgctcatggcgctacagcatgctggttaaagatggtgtg gtagaaaaaatgtttatcgaaccgaatgagccgggcgacccgttcaaagtatcggacgcc gataccatgctcaaatacattgcccctcaatacaaggtgcaagaatcagtgactattttc actaagcctggatgtccttattgcgccaaggcgaaacaagcgctgattgatgccggtcta cagtatgaagagctgattttaggtaaagacgccaccacagtgagcctgcgcgctgtttct ggccgtaccacggtgccgcaagtgtttatcggtggtaaacacattggtggcagcgacgac ttagaagtctacctaaatcaataa |
| A6AFP4 | UniRef cluster | -------------------------------------------KYIAPQYKVQESVTIFTK-----PGCPYCA-KAKQALIDAG---LQYEE----LILG---KDATTVSLRAVS----GRT----------TVPQVFIGGK--------HIGGSDD----LEVYLNQ-------------------------- | atgaggaacacaatgtttacatctaaagaaggtcaaaccattccacaggttacttttcct actcgccaaggtgacgcttgggtcaatgtgactagcgatgaactgttcaaaggcaaaacc gttatcgtgtttagcttgccgggtgcctttactccaacctgttcatccactcacctaccg cgctacaacgagctgtatcctgtctttaaagagaatggtgtcgacagcattctgtgcgta tcggtcaacgatactttcgtgatgaatgcttggaaagatgaccaaaatgccgacaacatc accttcattcctgatggtaatggtgaatttaccgatggtatgggcatgctggtggataaa aatgaccttggctttggtaaacgctcatggcgctacagcatgctggttaaagacggtgtg gtagaaaaaatgtttatcgaaccgaatgagccgggcgacccgttcaaagtatcggacgcc gataccatgctcaaatacattgcccctcaatacaaggtgcaagaatcagtgactattttc actaagccaggctgtccttattgcgccaaggcgaaacaagcgctgattgatgccggtcta cagtatgaagagctgattttaggtaaagacgccaccacagtgagcctgcgcgctgtttct ggccgtaccacggtgccgcaagtgtttatcggtggtaaacacattggtggcagcgacgac ttagaagtctacctaaatcaataa |
| A6A149 | UniRef cluster | -------------------------------------------KYIAPQYKVQESVTIFTK-----PGCPYCA-KAKQALIDAG---LQYEE----LILG---KDATTVSLRAVS----GRT----------TVPQVFIGGK--------HIGGSDD----LEVYLNQ-------------------------- | atgaggaacacaatgtttacatctaaagaaggtcaaaccattccacaggttacttttcct actcgccaaggtgacgcttgggtcaatgtgactagcgatgagctgttcaaaggcaaaacc gttatcgtgtttagcttgccgggtgcctttactccaacctgttcatccactcacctaccg cgctacaacgagctgtttcctgtctttaaagagcatggtgtcgacagcattctgtgcgta tcggtcaacgatactttcgtgatgaatgcttggaaagatgaccaaaacgccgacaacatc accttcattcctgatggtaatggtgaatttaccgatggtatgggcatgttggtggataaa aatgaccttggctttggtaaacgctcatggcgctacagcatgctggttaaagacggtgtg gtagaaaaaatgtttatcgaaccgaatgagccgggcgacccgttcaaagtatcggacgcc gataccatgctcaaatacattgcccctcaatacaaggtgcaagaatcagtgactattttc actaagcctggctgtccttattgcgccaaggcgaaacaagcgctgattgatgccggtcta cagtatgaagagctgattttaggtaaagacgccaccacagtgagcctgcgcgctgtttct ggccgtaccacggtgccgcaagtgtttatcggtggtaaacacattggtggcagcgacgac ttagaagtctacctaaatcaataa |
| A5F510 | UniRef cluster | -------------------------------------------KYIAPQYKVQESVTIFTK-----PGCPYCA-KAKQALIDAG---LQYEE----LILG---KDATTVSLRAVS----GRT----------TVPQVFIGGK--------HIGGSDD----LEVYLNQ-------------------------- | atgaggaacacaatgtttacatctaaagaaggtcaaaccattccacaggttacttttcct actcgccaaggtgacgcttgggtcaatgtgactagcgatgaactgttcaaaggcaaaacc gttatcgtgtttagcttgccgggtgcctttactccaacctgttcatccactcacctaccg cgctacaacgagctgtttcctgtctttaaagagcatggtgtcgacagcattctgtgcgta tcggtcaacgatactttcgtgatgaatgcttggaaagatgaccaaaatgccgacaacatc accttcattcctgatggtaatggtgaatttaccgatggtatgggcatgctggtggataaa aatgaccttggctttggtaaacgctcatggcgctacagcatgctggttaaagacggtgtg gtagaaaaaatgtttatcgaaccgaatgagccgggcgacccgttcaaagtatcggacgcc gataccatgctcaaatacattgcccctcaatacaaggtgcaagaatcagtgactattttc actaagccaggctgtccttattgcgccaaggcgaaacaagcgctgattgatgccggtcta cagtatgaagagctgattttaggtaaagacgctaccacagtgagtctgcgcgccgtttct ggccgtaccacggtgccgcaagtgtttatcggtggtaaacacattggtggtagcgacgac ttagaagtctacctaaatcaataa |
| A3GXC3 | UniRef cluster | -------------------------------------------KYIAPQYKVQESVTIFTK-----PGCPYCA-KAKQALIDAG---LQYEE----LILG---KDATTVSLRAVS----GRT----------TVPQVFIGGK--------HIGGSDD----LEVYLNQ-------------------------- | atgaggaacacaatgtttacatctaaagaaggtcaaaccattccacaggttacttttcct actcgccaaggtgacgcttgggtcaatgtgactagcgatgaactgttcaaaggcaaaacc gttatcgtgtttagcttgccgggtgcctttactccaacctgttcatccactcacctaccg cgctacaacgagctgtttcctgtctttaaagagcatggtgtcgacagcattctgtgcgta tcggtcaacgatactttcgtgatgaatgcttggaaagatgaccaaaatgccgacaacatc accttcattcctgatggtaatggtgaatttaccgatggtatgggcatgctggtggataaa aatgaccttggctttggtaaacgctcatggcgctacagcatgctggttaaagacggtgtg gtagaaaaaatgtttatcgaaccgaatgagccgggcgacccgttcaaagtatcggacgcc gataccatgctcaaatacattgcccctcaatacaaggtgcaagaatcagtgactattttc actaagccaggctgtccttattgcgccaaggcgaaacaagcgctgattgatgccggtcta cagtatgaagagctgattttaggtaaagacgctaccacagtgagtctgcgcgccgtttct ggccgtaccacggtgccgcaagtgtttatcggtggtaaacacattggtggtagcgacgac ttagaagtctacctaaatcaataa |
| A3GRD5 | UniRef cluster | -------------------------------------------KYIAPQYKVQESVTIFTK-----PGCPYCA-KAKQALIDAG---LQYEE----LILG---KDATTVSLRAVS----GRT----------TVPQVFIGGK--------HIGGSDD----LEVYLNQ-------------------------- | atgaggaacacaatgtttacatctaaagaaggtcaaaccattccacaggttacttttcct actcgccaaggtgacgcttgggtcaatgtgactagcgatgaactgttcaaaggcaaaacc gttatcgtgtttagcttgccgggtgcctttactccaacctgttcatccactcacctaccg cgctacaacgagctgtttcctgtctttaaagagcatggtgtcgacagcattctgtgcgta tcggtcaacgatactttcgtgatgaatgcttggaaagatgaccaaaatgccgacaacatc accttcattcctgatggtaatggtgaatttaccgatggtatgggcatgctggtggataaa aatgaccttggctttggtaaacgctcatggcgctacagcatgctggttaaagacggtgtg gtagaaaaaatgtttatcgaaccgaatgagccgggcgacccgttcaaagtatcggacgcc gataccatgctcaaatacattgcccctcaatacaaggtgcaagaatcagtgactattttc actaagccaggctgtccttattgcgccaaggcgaaacaagcgctgattgatgccggtcta cagtatgaagagctgattttaggtaaagacgctaccacagtgagtctgcgcgccgtttct ggccgtaccacggtgccgcaagtgtttatcggtggtaaacacattggtggtagcgacgac ttagaagtctacctaaatcaataa |
| A3EJ81 | UniRef cluster | -------------------------------------------KYIAPQYKVQESVTIFTK-----PGCPYCA-KAKQALIDAG---LQYEE----LILG---KDATTVSLRAVS----GRT----------TVPQVFIGGK--------HIGGSDD----LEVYLNQ-------------------------- | atgaggaacacaatgtttacatctaaagaaggtcaaaccattccacaggttacttttcct actcgccaaggtgacgcttgggtcaatgtgactagcgatgaactgttcaaaggcaaaacc gttatcgtgtttagcttgccgggtgcctttactccaacctgttcatccactcacctaccg cgctacaacgagctgtttcctgtctttaaagagcatggtgtcgacagcattctgtgcgta tcggtcaacgatactttcgtgatgaatgcttggaaagatgaccaaaatgccgacaacatc accttcattcctgatggtaatggtgaatttaccgatggtatgggcatgctggtggataaa aatgaccttggctttggtaaacgctcatggcgctacagcatgctggttaaagacggtgtg gtagaaaaaatgtttatcgaaccgaatgagccgggcgacccgttcaaagtatcggacgcc gataccatgctcaaatacattgcccctcaatacaaggtgcaagaatcagtgactattttc actaagcctggctgtccttattgcgccaaggcgaaacaagcgctgattgatgccggtcta cagtatgaagagctgattttaggtaaagacgccaccacagtgagcctgcgcgctgtttct ggccgtaccacggtgccgcaagtgtttatcggtggtaaacacattggtggcagcgacgac ttagaagtctacctaaatcaataa |
| A3EB97 | UniRef cluster | -------------------------------------------KYIAPQYKVQESVTIFTK-----PGCPYCA-KAKQALIDAG---LQYEE----LILG---KDATTVSLRAVS----GRT----------TVPQVFIGGK--------HIGGSDD----LEVYLNQ-------------------------- | atgaggaacacaatgtttacatctaaagaaggtcaaaccattccacaggttacttttcct actcgccaaggtgacgcttgggtcaatgtgactagcgatgaactgttcaaaggcaaaacc gttatcgtgtttagcttgccgggtgcctttactccaacctgttcatccactcacctaccg cgctacaacgagctgtttcctgtctttaaagagcatggtgtcgacagcattctgtgcgta tcggtcaacgatactttcgtgatgaatgcttggaaagatgaccaaaatgccgacaacatc accttcattcctgatggtaatggtgaatttaccgatggtatgggcatgctggtggataaa aatgaccttggctttggtaaacgctcatggcgctacagcatgctggttaaagacggtgtg gtagaaaaaatgtttatcgaaccgaatgagccgggcgacccgttcaaagtatcggacgcc gataccatgctcaaatacattgcccctcaatacaaggtgcaagaatcagtgactattttc actaagccaggctgtccttattgcgccaaggcgaaacaagcgctgattgatgccggtcta cagtatgaagagctgattttaggtaaagacgctaccacagtgagtctgcgcgccgtttct ggccgtaccacggtgccgcaagtgtttatcggtggtaaacacattggtggtagcgacgac ttagaagtctacctaaatcaataa |
| A2PVZ9 | UniRef cluster | -------------------------------------------KYIAPQYKVQESVTIFTK-----PGCPYCA-KAKQALIDAG---LQYEE----LILG---KDATTVSLRAVS----GRT----------TVPQVFIGGK--------HIGGSDD----LEVYLNQ-------------------------- | atgaggaacacaatgtttacatctaaagaaggtcaaaccattccacaggttacttttcct actcgccaaggtgacgcttgggtcaatgtgactaccgatgaactgttcaaaggcaaaacc gttatcgtgtttagcttgccgggtgcctttactccaacctgttcatccactcacctaccg cgctacaacgagctgtttcctgtctttaaagagcatggtgtcgacagcattctgtgcgta tcggtcaacgatactttcgtgatgaatgcttggaaagatgaccaaaacgccgacaacatc accttcattcctgatggtaatggtgaatttaccgatggtatgggcatgttggtggataaa aatgaccttggctttggtaaacgctcatggcgctacagcatgctggttaaagacggtgtg gtagaaaaaatgtttatcgaaccgaatgagccgggcgacccgttcaaagtatcggacgcc gataccatgctcaaatacattgcccctcaatacaaggtgcaagaatcagtgactattttc actaagccaggctgcccttattgcgccaaggcgaaacaagcgctgattgatgccggtcta cagtatgaagagctgattttaggtaaagacgccaccacagtgagcctgcgcgctgtttct ggccgtaccacggtgccgcaagtgtttatcggtggtaaacacattggtggcagcgacgac ttagaagtctacctaaatcaataa |
| A2PLL7 | UniRef cluster | -------------------------------------------KYIAPQYKVQESVTIFTK-----PGCPYCA-KAKQALIDAG---LQYEE----LILG---KDATTVSLRAVS----GRT----------TVPQVFIGGK--------HIGGSDD----LEVYLNQ-------------------------- | atgaggaacacaatgtttacatctaaagaaggtcaaaccattccacaggttacttttcct actcgccaaggtgacgcttgggtcaatgtgactagcgatgaactgttcaaaggcaaaacc gttatcgtgtttagcttgccgggtgcctttactccaacctgttcatccactcacctaccg cgctacaacgagctgtttcctgtctttaaagagcatggtgtcgacagcattctgtgcgta tcggtcaacgatactttcgtgatgaatgcttggaaagatgaccaaaatgccgacaacatc accttcattcctgatggtaatggtgaatttaccgatggtatgggcatgctggtggataaa aatgaccttggctttggtaaacgctcatggcgctacagcatgctggttaaagacggtgtg gtagaaaaaatgtttatcgaaccgaatgagccgggcgacccgttcaaagtatcggacgcc gataccatgctcaaatacattgcccctcaatacaaggtgcaagaatcagtgactattttc actaagccaggctgtccttattgcgccaaggcgaaacaagcgctgattgatgccggtcta cagtatgaagagctgattttaggtaaagacgctaccacagtgagtctgcgcgccgtttct ggccgtaccacggtgccgcaagtgtttatcggtggtaaacacattggtggtagcgacgac ttagaagtctacctaaatcaataa |
| A2P6Y2 | UniRef cluster | -------------------------------------------KYIAPQYKVQESVTIFTK-----PGCPYCA-KAKQALIDAG---LQYEE----LILG---KDATTVSLRAVS----GRT----------TVPQVFIGGK--------HIGGSDD----LEVYLNQ-------------------------- | atgaggaacacaatgtttacatctaaagaaggtcaaaccattccacaggttacttttcct actcgccaaggtgacgcttgggtcaatgtgactagcgatgagcttttcaaaggcaaaacc gttatcgtgtttagcttgccgggtgcctttactccaacctgttcatccactcacctaccg cgctacaacgagctgtttcctgtctttaaagagcatggtgtcgacagcattctgtgcgta tcggtcaacgatactttcgtgatgaatgcttggaaagatgaccaaaacgccgacaacatc acctttattcctgatggtaatggtgaatttaccgatggtatgggcatgctggtggataaa aatgaccttggctttggtaaacgctcatggcgctacagcatgctggttaaagatggtgtg gtagaaaaaatgtttatcgaaccgaatgagccgggcgacccgttcaaagtatcggacgcc gataccatgctcaaatacattgcccctcaatacaaggtgcaagaatcagtgactattttc actaagcctggatgtccttattgcgccaaggcgaaacaagcgctgattgatgccggtcta cagtatgaagagctgattttaggtaaagacgccaccacagtgagcctgcgcgctgtttct ggccgtaccacggtgccgcaagtgtttatcggtggtaaacacattggtggcagcgacgac ttagaagtctacctaaatcaataa |
| A1F753 | UniRef cluster | -------------------------------------------KYIAPQYKVQESVTIFTK-----PGCPYCA-KAKQALIDAG---LQYEE----LILG---KDATTVSLRAVS----GRT----------TVPQVFIGGK--------HIGGSDD----LEVYLNQ-------------------------- | atgaggaacacaatgtttacatctaaagaaggtcaaaccattccacaggttacttttcct actcgccaaggtgacgcttgggtcaatgtgactagcgatgaactgttcaaaggcaaaacc gttatcgtgtttagcttgccgggtgcctttactccaacctgttcatccactcacctaccg cgctacaacgagctgtttcctgtctttaaagagcatggtgtcgacagcattctgtgcgta tcggtcaacgatactttcgtgatgaatgcttggaaagatgaccaaaatgccgacaacatc accttcattcctgatggtaatggtgaatttaccgatggtatgggcatgctggtggataaa aatgaccttggctttggtaaacgctcatggcgctacagcatgctggttaaagacggtgtg gtagaaaaaatgtttatcgaaccgaatgagccgggcgacccgttcaaagtatcggacgcc gataccatgctcaaatacattgcccctcaatacaaggtgcaagaatcagtgactattttc actaagccaggctgtccttattgcgccaaggcgaaacaagcgctgattgatgccggtcta cagtatgaagagctgattttaggtaaagacgctaccacagtgagtctgcgcgccgtttct ggccgtaccacggtgccgcaagtgtttatcggtggtaaacacattggtggtagcgacgac ttagaagtctacctaaatcaataa |
| A1EMB6 | UniRef cluster | -------------------------------------------KYIAPQYKVQESVTIFTK-----PGCPYCA-KAKQALIDAG---LQYEE----LILG---KDATTVSLRAVS----GRT----------TVPQVFIGGK--------HIGGSDD----LEVYLNQ-------------------------- | atgaggaacacaatgtttacatctaaagaaggtcaaaccattccacaggttacttttcct actcgccaaggtgacgcttgggtcaatgtgactagcgatgaactgttcaaaggcaaaacc gttatcgtgtttagcttgccgggtgcctttactccaacctgttcatccactcacctaccg cgctacaacgagctgtttcctgtctttaaagagcatggtgtcgacagcattctgtgcgta tcggtcaacgatactttcgtgatgaatgcttggaaagatgaccaaaatgccgacaacatc accttcattcctgatggtaatggtgaatttaccgatggtatgggcatgctggtggataaa aatgaccttggctttggtaaacgctcatggcgctacagcatgctggttaaagatggtgtg gtagaaaaaatgtttatcgaaccgaatgagccgggcgacccgttcaaagtatcggacgcc gataccatgctcaaatacattgcccctcaatacaaggtgcaagaatcagtgactattttc actaagcctggatgtccttattgcgccaaggcgaaacaagcgctgattgatgccggtcta cagtatgaagagctgattttaggtaaagacgccaccacagtgagcctgcgcgctgtttct ggccgtaccacggtgccgcaagtgtttatcggtggtaaacacattggtggcagcgacgac ttagaagtctacctaaatcaataa |
| Q4QMX3 | UniRef cluster | -------------------------------------------KYLAPQHQVQESISIFTK-----PGCPFCA-KAKQLLHDKG---LSFEE----IILG---HDATIVSVRAVS----GRA----------TVPQVFIGGK--------HIGGSDD----LEKYFA--------------------------- | atgtctagtatggaaggaaaaaaagtccctcaagtgacattccgcactcgtcagggtgat aaatgggttgatgtaactacctcagagttatttgataacaaaacagtgatcgtgttctca ttaccgggcgcattcactccaacttgctcatcatcacacttaccacgttacaacgaatta gcgccagtattcaaaaaatacggtgtagacgatattcttgttgtatctgtaaatgatact ttcgtaatgaacgcatggaaagaagatgaaaaatctgaaaacatcactttcattccagat ggtaatggtgaatttaccgaaggcatgggtatgttagttggtaaagaagatttaggcttc ggtaaacgttcatggcgttattctatgcttgtgaaaaacggcgtagttgaaaaaatgttt atcgaaccaaacgaaccaggcgatccgttcaaagtatccgatgctgacactatgttgaaa taccttgcaccacaacaccaagtgcaagagtctatttcaatctttacaaaacctggctgt cctttctgtgcaaaagcaaaacaacttttacacgataaaggcttaagctttgaagaaatc atattaggtcacgatgcaacaatcgtgagcgtacgtgcagtttcaggtcgtgctactgtt ccacaagtgtttatcggtggtaaacacattggcggtagcgacgatttggaaaaatacttt gcataa |
| A5U9R8 | UniRef cluster | -------------------------------------------KYLAPQHQVQESISIFTK-----PGCPFCA-KAKQLLHDKG---LSFEE----IILG---HDATIVSVRAVS----GRA----------TVPQVFIGGK--------HIGGSDD----LEKYFA--------------------------- | atgtctaatatggaaggaaaaaaagtccctcaaatgacattccgcactcgtcagggtgat aaatgggttgatgtaactacctcagagttatttgataacaaaacagtgatcgtgttctca ttaccgggagcattcactccaacttgctcatcatcacacttaccacgttacaacgaatta gcgccagtattcaaaaaatacggtgtagacgatattcttgttgtatctgtaaatgatact ttcgtaatgaacgcatggaaagaagatgaaaaatctgaaaacatcactttcattccagat ggtaatggtgaatttaccgaaggcatgggtatgttagttggtaaagaagatttaggcttc ggtaaacgttcatggcgttattctatgcttgtgaaaaacggcgtagttgaaaaaatgttt atcgaaccaaacgaaccaggcgatccgttcaaagtatccgatgctgacactatgttgaaa taccttgcaccacaacaccaagtgcaagagtctatttcaatcttcacaaaacctggctgt cctttctgtgcaaaagcaaaacaacttttacacgataaaggcttaagctttgaagaaatc atattaggtcacgatgcaacaatcgtgagcgtacgtgcagtttcaggtcgtgctactgtt ccacaagtgtttatcggtggtaaacacattggcggtagcgacgatttggaaaaatacttt gcataa |
| A4NUZ4 | UniRef cluster | -------------------------------------------KYLAPQHQVQESISIFTK-----PGCPFCA-KAKQLLHDKG---LSFEE----IILG---HDATIVSVRAVS----GRA----------TVPQVFIGGK--------HIGGSDD----LEKYFA--------------------------- | atgtctagtatggaaggaaaaaaagtccctcaagtgacattccgcactcgtcagggtgat aaatgggttgatgtaactacctcagagttatttgataacaaaacagtgatcgtgttctca ttaccgggcgcattcactccaacttgctcatcatcacacttaccacgttacaacgaatta gcgccagtattcaaaaaatacggtgtagatgatattcttgttgtatctgtaaatgatact ttcgtaatgaacgcatggaaagaagatgaaaaatctgaaaacatcactttcattccagat ggtaatggtgaatttaccgaaggcatgggtatgttagttggtaaagaagatttaggcttc ggtaaacgttcatggcgttattctatgcttgtgaaaaacggcgtagttgaaaaaatgttt atcgaaccaaacgaaccaggcgatccgttcaaagtatccgatgctgacactatgttgaaa taccttgcaccacaacaccaagtgcaagagtctatttcaatcttcacaaaacctggctgt cctttctgtgcaaaagcaaaacaacttttacacgataaaggcttaagctttgaagaaatc atattaggtcacgatgcaacaatcgtgagcgtacgtgcagtttcaggtcgtgctactgtt ccacaagtgtttatcggtggtaaacacattggcggtagcgacgatttggaaaaatacttt gcataa |
| A4NQK7 | UniRef cluster | -------------------------------------------KYLAPQHQVQESISIFTK-----PGCPFCA-KAKQLLHDKG---LSFEE----IILG---HDATIVSVRAVS----GRA----------TVPQVFIGGK--------HIGGSDD----LEKYFA--------------------------- | atgtctagtatggaaggaaaaaaagtccctcaagtgacattccgcactcgtcagggtgat aaatgggttgatgtaactacctcagagttatttgataacaaaacagtgatcgtgttctca ttaccgggcgcattcactccaacttgctcatcatcacacttaccacgttacaacgaatta gcgccagtattcaaaaaatacggtgtagacgatattcttgttgtatctgtaaatgatact ttcgtaatgaacgcatggaaagaagatgaaaaatctgaaaacatcactttcattccagat ggtaatggtgaatttaccgaaggcatgggtatgttagttggtaaagaagatttaggcttc ggtaaacgttcatggcgttattctatgcttgtgaaaaacggcgtagttgaaaaaatgttt atcgaaccaaacgaaccaggcgatccgttcaaagtatccgatgctgacactatgttgaaa taccttgcaccacaacaccaagtgcaagagtctatttcaatcttcacaaaacctggctgt cctttctgtgcaaaagcaaaacaacttttacacgataaaggcttaagctttgaagaaatc atattaggtcacgatgcaacaatcgtgagcgtacgtgcagtttcaggtcgtgctactgtt ccacaagtgtttatcggtggtaaacacattggcggtagcgacgatttggaaaaatacttt gcataa |
| A4NNT9 | UniRef cluster | -------------------------------------------KYLAPQHQVQESISIFTK-----PGCPFCA-KAKQLLHDKG---LSFEE----IILG---HDATIVSVRAVS----GRA----------TVPQVFIGGK--------HIGGSDD----LEKYFA--------------------------- | atgtctaatatggaaggaaaaaaagtccctcaagtgacattccgcactcgtcagggtgat aaatgggttgatgtaactacctcagagttatttgataacaaaacagtgatcgtgttctca ttaccgggagcattcactccaacttgctcatcatcacacttaccacgttacaacgaatta gcgccagtattcaaaaaatacggtgtagatgatattcttgttgtatctgtaaatgatact ttcgtaatgaacgcatggaaagaagatgaaaaatctgaaaacatcactttcattccagat ggtaatggtgaatttaccgaaggcatgggtatgttagttggtaaagaagatttaggcttc ggtaaacgttcatggcgttattctatgcttgtgaaaaacggcgtagttgaaaaaatgttt atcgaaccaaacgaaccaggcgatccgttcaaagtatccgatgctgacactatgttgaaa taccttgcaccacaacaccaagtgcaagagtctatttcaatcttcacaaaacctggctgt cctttctgtgcaaaagcaaaacaacttttacacgataaaggcttaagctttgaagaaatc atattaggtcacgatgcaacaatcgtgagcgtacgtgcagtttcaggtcgtgctactgtt ccacaagtgtttatcggtggtaaacacattggcggtagcgacgatttggaaaaatacttt gcataa |
| A4NGR7 | UniRef cluster | -------------------------------------------KYLAPQHQVQESISIFTK-----PGCPFCA-KAKQLLHDKG---LSFEE----IILG---HDATIVSVRAVS----GRA----------TVPQVFIGGK--------HIGGSDD----LEKYFA--------------------------- | atgtctagtatggaaggaaaaaaagtccctcaagtgacattccgcactcgtcagggtgat aaatgggttgatgtaactacctcagagttatttgataacaaaacagtgatcgtgttctca ttaccgggcgcattcactccaacttgctcatcatcacacttaccacgttacaacgaatta gtgccagtattcaaaaaatacggtgtagacgatattcttgttgtatctgtaaatgatact ttcgtaatgaacgcatggaaagaagatgaaaaatctgaaaacatcactttcattccagat ggtaatggtgaatttaccgaaggcatgggtatgttagttggtaaagaagatttaggcttc ggtaaacgttcatggcgttattctatgcttgtgaaaaacggcgtagttgaaaaaatgttt atcgaaccaaacgaaccaggcgatccgttcaaagtatccgatgctgacactatgttgaaa taccttgcaccacaacaccaagtgcaagagtctatttcaatctttacaaaacctggctgt cctttctgtgcaaaagcaaaacaacttttacacgataaaggcttaagctttgaagaaatc atattaggtcacgatgcaacaatcgtgagcgtacgtgcagtttcaggtcgtgctactgtt ccacaagtgtttatcggtggtaaacacattggcggtagcgacgatttggaaaaatacttt gcataa |
| A4NBB5 | UniRef cluster | -------------------------------------------KYLAPQHQVQESISIFTK-----PGCPFCA-KAKQLLHDKG---LSFEE----IILG---HDATIVSVRAVS----GRA----------TVPQVFIGGK--------HIGGSDD----LEKYFA--------------------------- | atgtctaatatggaaggaaaaaaagtccctcaagtgacattccgcactcgtcagggtgat aaatgggttgatgtaactacctcagagttatttgataacaaaacagtgatcgtgttctca ttaccgggcgcattcactccaacttgctcatcatcacacttaccacgttacaacgaatta gcgccagtattcaaaaaatacggtgtagacgatattctcgttgtatctgtaaatgatact ttcgtaatgaacgcatggaaagaagatgaaaaatctgaaaacatcactttcattccagat ggtaatggtgaatttaccgaaggcatgggtatgttagttggtaaagaagatttaggcttc ggtaaacgttcatggcgttattctatgcttgtgaaaaacggcgtagttgaaaaaatgttt atcgaaccaaacgaaccaggcgatccgttcaaagtatccgatgctgacactatgttgaaa taccttgcaccacaacaccaagtgcaagagtctatttcaatcttcacaaaacctggctgt cctttctgtgcaaaagcaaaacaacttttacacgataaaggcttaagctttgaagaaatc atattaggtcacgatgcaacaatcgtaagcgtacgtgcagtttcaggtcgtgctactgtt ccacaagtgtttatcggtggtaaacacattggcggtagcgacgatttggaaaaatacttt gcataa |
| A4N6H3 | UniRef cluster | -------------------------------------------KYLAPQHQVQESISIFTK-----PGCPFCA-KAKQLLHDKG---LSFEE----IILG---HDATIVSVRAVS----GRA----------TVPQVFIGGK--------HIGGSDD----LEKYFA--------------------------- | atgtctagtatggaaggaaaaaaagtccctcaagtgacattccgcactcgtcagggtgat aaatgggttgatgtaactacctcagagttatttgataacaaaacagtgatcgtgttctca ttaccgggcgcattcactccaacttgctcatcatcacacttaccacgttacaacgaatta gcgccagtattcaaaaaatacggtgtagatgatattcttgttgtatctgtaaatgatact ttcgtaatgaacgcatggaaagaagatgaaaaatctgaaaacatcactttcattccagat ggtaatggtgaatttaccgaaggcatgggtatgttagttggtaaagaagatttaggcttc ggtaaacgttcatggcgttattctatgcttgtgaaaaacggcgtagttgaaaaaatgttt atcgaaccaaacgaaccaggcgatccgttcaaagtatccgatgctgacactatgttgaaa taccttgcaccacaacaccaagtgcaagagtctatttcaatcttcacaaaacctggctgt cctttctgtgcaaaagcaaaacaacttttacacgataaaggcttaagctttgaagaaatc atattaggtcacgatgcaacaatcgtgagcgtacgtgcagtttcaggtcgtgctactgtt ccacaagtgtttatcggtggtaaacacattggcggtagcgacgatttggaaaaatacttt gcataa |
| A4MYL9 | UniRef cluster | -------------------------------------------KYLAPQHQVQESISIFTK-----PGCPFCA-KAKQLLHDKG---LSFEE----IILG---HDATIVSVRAVS----GRA----------TVPQVFIGGK--------HIGGSDD----LEKYFA--------------------------- | atgtctagtatggaaggaaaaaaagtccctcaagtgacattccgcactcgtcagggtgat aaatgggttgatgtaactacctcagagttatttgataacaaaacagtgatcgtgttctca ttaccgggcgcattcactccaacttgctcatcatcacacttaccacgttacaacgaatta gcaccagtattcaaaaaatacggtgtagacgatattcttgttgtatctgtaaatgatact ttcgtaatgaacgcatggaaagaagatgaaaaatctgaaaacatcactttcattccagat ggtaatggtgaatttaccgaaggcatgggtatgttagttggtaaagaagatttaggcttc ggtaaacgttcatggcgttattctatgcttgtgaaaaacggcgtagttgaaaaaatgttt atcgaaccaaacgaaccaggcgatccgttcaaagtatccgatgctgacactatgttgaaa taccttgcaccacaacaccaagtgcaagagtctatttcaatcttcacaaaacctggctgt cctttctgtgcaaaagcaaaacaacttttacacgataaaggcttaagctttgaagaaatc atattaggtcacgatgcaacaatcgtgagcgtacgtgcagtttcaggtcgtgctactgtt ccacaagtgtttatcggtggtaaacacattggcggtagcgacgatttggaaaaatacttt gcataa |
| Q4QMX3 | UniRef cluster | -------------------------------------------KYLAPQHQVQESISIFTK-----PGCPFCA-KAKQLLHDKG---LSFEE----IILG---HDATIVSVRAVS----GRA----------TVPQVFIGGK--------HIGGSDD----LEKYFA--------------------------- | atgtctagtatggaaggaaaaaaagtccctcaagtgacattccgcactcgtcagggtgat aaatgggttgatgtaactacctcagagttatttgataacaaaacagtgatcgtgttctca ttaccgggcgcattcactccaacttgctcatcatcacacttaccacgttacaacgaatta gcgccagtattcaaaaaatacggtgtagacgatattcttgttgtatctgtaaatgatact ttcgtaatgaacgcatggaaagaagatgaaaaatctgaaaacatcactttcattccagat ggtaatggtgaatttaccgaaggcatgggtatgttagttggtaaagaagatttaggcttc ggtaaacgttcatggcgttattctatgcttgtgaaaaacggcgtagttgaaaaaatgttt atcgaaccaaacgaaccaggcgatccgttcaaagtatccgatgctgacactatgttgaaa taccttgcaccacaacaccaagtgcaagagtctatttcaatctttacaaaacctggctgt cctttctgtgcaaaagcaaaacaacttttacacgataaaggcttaagctttgaagaaatc atattaggtcacgatgcaacaatcgtgagcgtacgtgcagtttcaggtcgtgctactgtt ccacaagtgtttatcggtggtaaacacattggcggtagcgacgatttggaaaaatacttt gcataa |
| A5U9R8 | UniRef cluster | -------------------------------------------KYLAPQHQVQESISIFTK-----PGCPFCA-KAKQLLHDKG---LSFEE----IILG---HDATIVSVRAVS----GRA----------TVPQVFIGGK--------HIGGSDD----LEKYFA--------------------------- | atgtctaatatggaaggaaaaaaagtccctcaaatgacattccgcactcgtcagggtgat aaatgggttgatgtaactacctcagagttatttgataacaaaacagtgatcgtgttctca ttaccgggagcattcactccaacttgctcatcatcacacttaccacgttacaacgaatta gcgccagtattcaaaaaatacggtgtagacgatattcttgttgtatctgtaaatgatact ttcgtaatgaacgcatggaaagaagatgaaaaatctgaaaacatcactttcattccagat ggtaatggtgaatttaccgaaggcatgggtatgttagttggtaaagaagatttaggcttc ggtaaacgttcatggcgttattctatgcttgtgaaaaacggcgtagttgaaaaaatgttt atcgaaccaaacgaaccaggcgatccgttcaaagtatccgatgctgacactatgttgaaa taccttgcaccacaacaccaagtgcaagagtctatttcaatcttcacaaaacctggctgt cctttctgtgcaaaagcaaaacaacttttacacgataaaggcttaagctttgaagaaatc atattaggtcacgatgcaacaatcgtgagcgtacgtgcagtttcaggtcgtgctactgtt ccacaagtgtttatcggtggtaaacacattggcggtagcgacgatttggaaaaatacttt gcataa |
| A4NUZ4 | UniRef cluster | -------------------------------------------KYLAPQHQVQESISIFTK-----PGCPFCA-KAKQLLHDKG---LSFEE----IILG---HDATIVSVRAVS----GRA----------TVPQVFIGGK--------HIGGSDD----LEKYFA--------------------------- | atgtctagtatggaaggaaaaaaagtccctcaagtgacattccgcactcgtcagggtgat aaatgggttgatgtaactacctcagagttatttgataacaaaacagtgatcgtgttctca ttaccgggcgcattcactccaacttgctcatcatcacacttaccacgttacaacgaatta gcgccagtattcaaaaaatacggtgtagatgatattcttgttgtatctgtaaatgatact ttcgtaatgaacgcatggaaagaagatgaaaaatctgaaaacatcactttcattccagat ggtaatggtgaatttaccgaaggcatgggtatgttagttggtaaagaagatttaggcttc ggtaaacgttcatggcgttattctatgcttgtgaaaaacggcgtagttgaaaaaatgttt atcgaaccaaacgaaccaggcgatccgttcaaagtatccgatgctgacactatgttgaaa taccttgcaccacaacaccaagtgcaagagtctatttcaatcttcacaaaacctggctgt cctttctgtgcaaaagcaaaacaacttttacacgataaaggcttaagctttgaagaaatc atattaggtcacgatgcaacaatcgtgagcgtacgtgcagtttcaggtcgtgctactgtt ccacaagtgtttatcggtggtaaacacattggcggtagcgacgatttggaaaaatacttt gcataa |
| A4NQK7 | UniRef cluster | -------------------------------------------KYLAPQHQVQESISIFTK-----PGCPFCA-KAKQLLHDKG---LSFEE----IILG---HDATIVSVRAVS----GRA----------TVPQVFIGGK--------HIGGSDD----LEKYFA--------------------------- | atgtctagtatggaaggaaaaaaagtccctcaagtgacattccgcactcgtcagggtgat aaatgggttgatgtaactacctcagagttatttgataacaaaacagtgatcgtgttctca ttaccgggcgcattcactccaacttgctcatcatcacacttaccacgttacaacgaatta gcgccagtattcaaaaaatacggtgtagacgatattcttgttgtatctgtaaatgatact ttcgtaatgaacgcatggaaagaagatgaaaaatctgaaaacatcactttcattccagat ggtaatggtgaatttaccgaaggcatgggtatgttagttggtaaagaagatttaggcttc ggtaaacgttcatggcgttattctatgcttgtgaaaaacggcgtagttgaaaaaatgttt atcgaaccaaacgaaccaggcgatccgttcaaagtatccgatgctgacactatgttgaaa taccttgcaccacaacaccaagtgcaagagtctatttcaatcttcacaaaacctggctgt cctttctgtgcaaaagcaaaacaacttttacacgataaaggcttaagctttgaagaaatc atattaggtcacgatgcaacaatcgtgagcgtacgtgcagtttcaggtcgtgctactgtt ccacaagtgtttatcggtggtaaacacattggcggtagcgacgatttggaaaaatacttt gcataa |
| A4NNT9 | UniRef cluster | -------------------------------------------KYLAPQHQVQESISIFTK-----PGCPFCA-KAKQLLHDKG---LSFEE----IILG---HDATIVSVRAVS----GRA----------TVPQVFIGGK--------HIGGSDD----LEKYFA--------------------------- | atgtctaatatggaaggaaaaaaagtccctcaagtgacattccgcactcgtcagggtgat aaatgggttgatgtaactacctcagagttatttgataacaaaacagtgatcgtgttctca ttaccgggagcattcactccaacttgctcatcatcacacttaccacgttacaacgaatta gcgccagtattcaaaaaatacggtgtagatgatattcttgttgtatctgtaaatgatact ttcgtaatgaacgcatggaaagaagatgaaaaatctgaaaacatcactttcattccagat ggtaatggtgaatttaccgaaggcatgggtatgttagttggtaaagaagatttaggcttc ggtaaacgttcatggcgttattctatgcttgtgaaaaacggcgtagttgaaaaaatgttt atcgaaccaaacgaaccaggcgatccgttcaaagtatccgatgctgacactatgttgaaa taccttgcaccacaacaccaagtgcaagagtctatttcaatcttcacaaaacctggctgt cctttctgtgcaaaagcaaaacaacttttacacgataaaggcttaagctttgaagaaatc atattaggtcacgatgcaacaatcgtgagcgtacgtgcagtttcaggtcgtgctactgtt ccacaagtgtttatcggtggtaaacacattggcggtagcgacgatttggaaaaatacttt gcataa |
| A4NGR7 | UniRef cluster | -------------------------------------------KYLAPQHQVQESISIFTK-----PGCPFCA-KAKQLLHDKG---LSFEE----IILG---HDATIVSVRAVS----GRA----------TVPQVFIGGK--------HIGGSDD----LEKYFA--------------------------- | atgtctagtatggaaggaaaaaaagtccctcaagtgacattccgcactcgtcagggtgat aaatgggttgatgtaactacctcagagttatttgataacaaaacagtgatcgtgttctca ttaccgggcgcattcactccaacttgctcatcatcacacttaccacgttacaacgaatta gtgccagtattcaaaaaatacggtgtagacgatattcttgttgtatctgtaaatgatact ttcgtaatgaacgcatggaaagaagatgaaaaatctgaaaacatcactttcattccagat ggtaatggtgaatttaccgaaggcatgggtatgttagttggtaaagaagatttaggcttc ggtaaacgttcatggcgttattctatgcttgtgaaaaacggcgtagttgaaaaaatgttt atcgaaccaaacgaaccaggcgatccgttcaaagtatccgatgctgacactatgttgaaa taccttgcaccacaacaccaagtgcaagagtctatttcaatctttacaaaacctggctgt cctttctgtgcaaaagcaaaacaacttttacacgataaaggcttaagctttgaagaaatc atattaggtcacgatgcaacaatcgtgagcgtacgtgcagtttcaggtcgtgctactgtt ccacaagtgtttatcggtggtaaacacattggcggtagcgacgatttggaaaaatacttt gcataa |
| A4NBB5 | UniRef cluster | -------------------------------------------KYLAPQHQVQESISIFTK-----PGCPFCA-KAKQLLHDKG---LSFEE----IILG---HDATIVSVRAVS----GRA----------TVPQVFIGGK--------HIGGSDD----LEKYFA--------------------------- | atgtctaatatggaaggaaaaaaagtccctcaagtgacattccgcactcgtcagggtgat aaatgggttgatgtaactacctcagagttatttgataacaaaacagtgatcgtgttctca ttaccgggcgcattcactccaacttgctcatcatcacacttaccacgttacaacgaatta gcgccagtattcaaaaaatacggtgtagacgatattctcgttgtatctgtaaatgatact ttcgtaatgaacgcatggaaagaagatgaaaaatctgaaaacatcactttcattccagat ggtaatggtgaatttaccgaaggcatgggtatgttagttggtaaagaagatttaggcttc ggtaaacgttcatggcgttattctatgcttgtgaaaaacggcgtagttgaaaaaatgttt atcgaaccaaacgaaccaggcgatccgttcaaagtatccgatgctgacactatgttgaaa taccttgcaccacaacaccaagtgcaagagtctatttcaatcttcacaaaacctggctgt cctttctgtgcaaaagcaaaacaacttttacacgataaaggcttaagctttgaagaaatc atattaggtcacgatgcaacaatcgtaagcgtacgtgcagtttcaggtcgtgctactgtt ccacaagtgtttatcggtggtaaacacattggcggtagcgacgatttggaaaaatacttt gcataa |
| A4N6H3 | UniRef cluster | -------------------------------------------KYLAPQHQVQESISIFTK-----PGCPFCA-KAKQLLHDKG---LSFEE----IILG---HDATIVSVRAVS----GRA----------TVPQVFIGGK--------HIGGSDD----LEKYFA--------------------------- | atgtctagtatggaaggaaaaaaagtccctcaagtgacattccgcactcgtcagggtgat aaatgggttgatgtaactacctcagagttatttgataacaaaacagtgatcgtgttctca ttaccgggcgcattcactccaacttgctcatcatcacacttaccacgttacaacgaatta gcgccagtattcaaaaaatacggtgtagatgatattcttgttgtatctgtaaatgatact ttcgtaatgaacgcatggaaagaagatgaaaaatctgaaaacatcactttcattccagat ggtaatggtgaatttaccgaaggcatgggtatgttagttggtaaagaagatttaggcttc ggtaaacgttcatggcgttattctatgcttgtgaaaaacggcgtagttgaaaaaatgttt atcgaaccaaacgaaccaggcgatccgttcaaagtatccgatgctgacactatgttgaaa taccttgcaccacaacaccaagtgcaagagtctatttcaatcttcacaaaacctggctgt cctttctgtgcaaaagcaaaacaacttttacacgataaaggcttaagctttgaagaaatc atattaggtcacgatgcaacaatcgtgagcgtacgtgcagtttcaggtcgtgctactgtt ccacaagtgtttatcggtggtaaacacattggcggtagcgacgatttggaaaaatacttt gcataa |
| A4MYL9 | UniRef cluster | -------------------------------------------KYLAPQHQVQESISIFTK-----PGCPFCA-KAKQLLHDKG---LSFEE----IILG---HDATIVSVRAVS----GRA----------TVPQVFIGGK--------HIGGSDD----LEKYFA--------------------------- | atgtctagtatggaaggaaaaaaagtccctcaagtgacattccgcactcgtcagggtgat aaatgggttgatgtaactacctcagagttatttgataacaaaacagtgatcgtgttctca ttaccgggcgcattcactccaacttgctcatcatcacacttaccacgttacaacgaatta gcaccagtattcaaaaaatacggtgtagacgatattcttgttgtatctgtaaatgatact ttcgtaatgaacgcatggaaagaagatgaaaaatctgaaaacatcactttcattccagat ggtaatggtgaatttaccgaaggcatgggtatgttagttggtaaagaagatttaggcttc ggtaaacgttcatggcgttattctatgcttgtgaaaaacggcgtagttgaaaaaatgttt atcgaaccaaacgaaccaggcgatccgttcaaagtatccgatgctgacactatgttgaaa taccttgcaccacaacaccaagtgcaagagtctatttcaatcttcacaaaacctggctgt cctttctgtgcaaaagcaaaacaacttttacacgataaaggcttaagctttgaagaaatc atattaggtcacgatgcaacaatcgtgagcgtacgtgcagtttcaggtcgtgctactgtt ccacaagtgtttatcggtggtaaacacattggcggtagcgacgatttggaaaaatacttt gcataa |
| Q4QMX3 | UniRef cluster | -------------------------------------------KYLAPQHQVQESISIFTK-----PGCPFCA-KAKQLLHDKG---LSFEE----IILG---HDATIVSVRAVS----GRA----------TVPQVFIGGK--------HIGGSDD----LEKYFA--------------------------- | atgtctagtatggaaggaaaaaaagtccctcaagtgacattccgcactcgtcagggtgat aaatgggttgatgtaactacctcagagttatttgataacaaaacagtgatcgtgttctca ttaccgggcgcattcactccaacttgctcatcatcacacttaccacgttacaacgaatta gcgccagtattcaaaaaatacggtgtagacgatattcttgttgtatctgtaaatgatact ttcgtaatgaacgcatggaaagaagatgaaaaatctgaaaacatcactttcattccagat ggtaatggtgaatttaccgaaggcatgggtatgttagttggtaaagaagatttaggcttc ggtaaacgttcatggcgttattctatgcttgtgaaaaacggcgtagttgaaaaaatgttt atcgaaccaaacgaaccaggcgatccgttcaaagtatccgatgctgacactatgttgaaa taccttgcaccacaacaccaagtgcaagagtctatttcaatctttacaaaacctggctgt cctttctgtgcaaaagcaaaacaacttttacacgataaaggcttaagctttgaagaaatc atattaggtcacgatgcaacaatcgtgagcgtacgtgcagtttcaggtcgtgctactgtt ccacaagtgtttatcggtggtaaacacattggcggtagcgacgatttggaaaaatacttt gcataa |
| A5U9R8 | UniRef cluster | -------------------------------------------KYLAPQHQVQESISIFTK-----PGCPFCA-KAKQLLHDKG---LSFEE----IILG---HDATIVSVRAVS----GRA----------TVPQVFIGGK--------HIGGSDD----LEKYFA--------------------------- | atgtctaatatggaaggaaaaaaagtccctcaaatgacattccgcactcgtcagggtgat aaatgggttgatgtaactacctcagagttatttgataacaaaacagtgatcgtgttctca ttaccgggagcattcactccaacttgctcatcatcacacttaccacgttacaacgaatta gcgccagtattcaaaaaatacggtgtagacgatattcttgttgtatctgtaaatgatact ttcgtaatgaacgcatggaaagaagatgaaaaatctgaaaacatcactttcattccagat ggtaatggtgaatttaccgaaggcatgggtatgttagttggtaaagaagatttaggcttc ggtaaacgttcatggcgttattctatgcttgtgaaaaacggcgtagttgaaaaaatgttt atcgaaccaaacgaaccaggcgatccgttcaaagtatccgatgctgacactatgttgaaa taccttgcaccacaacaccaagtgcaagagtctatttcaatcttcacaaaacctggctgt cctttctgtgcaaaagcaaaacaacttttacacgataaaggcttaagctttgaagaaatc atattaggtcacgatgcaacaatcgtgagcgtacgtgcagtttcaggtcgtgctactgtt ccacaagtgtttatcggtggtaaacacattggcggtagcgacgatttggaaaaatacttt gcataa |
| A4NUZ4 | UniRef cluster | -------------------------------------------KYLAPQHQVQESISIFTK-----PGCPFCA-KAKQLLHDKG---LSFEE----IILG---HDATIVSVRAVS----GRA----------TVPQVFIGGK--------HIGGSDD----LEKYFA--------------------------- | atgtctagtatggaaggaaaaaaagtccctcaagtgacattccgcactcgtcagggtgat aaatgggttgatgtaactacctcagagttatttgataacaaaacagtgatcgtgttctca ttaccgggcgcattcactccaacttgctcatcatcacacttaccacgttacaacgaatta gcgccagtattcaaaaaatacggtgtagatgatattcttgttgtatctgtaaatgatact ttcgtaatgaacgcatggaaagaagatgaaaaatctgaaaacatcactttcattccagat ggtaatggtgaatttaccgaaggcatgggtatgttagttggtaaagaagatttaggcttc ggtaaacgttcatggcgttattctatgcttgtgaaaaacggcgtagttgaaaaaatgttt atcgaaccaaacgaaccaggcgatccgttcaaagtatccgatgctgacactatgttgaaa taccttgcaccacaacaccaagtgcaagagtctatttcaatcttcacaaaacctggctgt cctttctgtgcaaaagcaaaacaacttttacacgataaaggcttaagctttgaagaaatc atattaggtcacgatgcaacaatcgtgagcgtacgtgcagtttcaggtcgtgctactgtt ccacaagtgtttatcggtggtaaacacattggcggtagcgacgatttggaaaaatacttt gcataa |
| A4NQK7 | UniRef cluster | -------------------------------------------KYLAPQHQVQESISIFTK-----PGCPFCA-KAKQLLHDKG---LSFEE----IILG---HDATIVSVRAVS----GRA----------TVPQVFIGGK--------HIGGSDD----LEKYFA--------------------------- | atgtctagtatggaaggaaaaaaagtccctcaagtgacattccgcactcgtcagggtgat aaatgggttgatgtaactacctcagagttatttgataacaaaacagtgatcgtgttctca ttaccgggcgcattcactccaacttgctcatcatcacacttaccacgttacaacgaatta gcgccagtattcaaaaaatacggtgtagacgatattcttgttgtatctgtaaatgatact ttcgtaatgaacgcatggaaagaagatgaaaaatctgaaaacatcactttcattccagat ggtaatggtgaatttaccgaaggcatgggtatgttagttggtaaagaagatttaggcttc ggtaaacgttcatggcgttattctatgcttgtgaaaaacggcgtagttgaaaaaatgttt atcgaaccaaacgaaccaggcgatccgttcaaagtatccgatgctgacactatgttgaaa taccttgcaccacaacaccaagtgcaagagtctatttcaatcttcacaaaacctggctgt cctttctgtgcaaaagcaaaacaacttttacacgataaaggcttaagctttgaagaaatc atattaggtcacgatgcaacaatcgtgagcgtacgtgcagtttcaggtcgtgctactgtt ccacaagtgtttatcggtggtaaacacattggcggtagcgacgatttggaaaaatacttt gcataa |
| A4NNT9 | UniRef cluster | -------------------------------------------KYLAPQHQVQESISIFTK-----PGCPFCA-KAKQLLHDKG---LSFEE----IILG---HDATIVSVRAVS----GRA----------TVPQVFIGGK--------HIGGSDD----LEKYFA--------------------------- | atgtctaatatggaaggaaaaaaagtccctcaagtgacattccgcactcgtcagggtgat aaatgggttgatgtaactacctcagagttatttgataacaaaacagtgatcgtgttctca ttaccgggagcattcactccaacttgctcatcatcacacttaccacgttacaacgaatta gcgccagtattcaaaaaatacggtgtagatgatattcttgttgtatctgtaaatgatact ttcgtaatgaacgcatggaaagaagatgaaaaatctgaaaacatcactttcattccagat ggtaatggtgaatttaccgaaggcatgggtatgttagttggtaaagaagatttaggcttc ggtaaacgttcatggcgttattctatgcttgtgaaaaacggcgtagttgaaaaaatgttt atcgaaccaaacgaaccaggcgatccgttcaaagtatccgatgctgacactatgttgaaa taccttgcaccacaacaccaagtgcaagagtctatttcaatcttcacaaaacctggctgt cctttctgtgcaaaagcaaaacaacttttacacgataaaggcttaagctttgaagaaatc atattaggtcacgatgcaacaatcgtgagcgtacgtgcagtttcaggtcgtgctactgtt ccacaagtgtttatcggtggtaaacacattggcggtagcgacgatttggaaaaatacttt gcataa |
| A4NGR7 | UniRef cluster | -------------------------------------------KYLAPQHQVQESISIFTK-----PGCPFCA-KAKQLLHDKG---LSFEE----IILG---HDATIVSVRAVS----GRA----------TVPQVFIGGK--------HIGGSDD----LEKYFA--------------------------- | atgtctagtatggaaggaaaaaaagtccctcaagtgacattccgcactcgtcagggtgat aaatgggttgatgtaactacctcagagttatttgataacaaaacagtgatcgtgttctca ttaccgggcgcattcactccaacttgctcatcatcacacttaccacgttacaacgaatta gtgccagtattcaaaaaatacggtgtagacgatattcttgttgtatctgtaaatgatact ttcgtaatgaacgcatggaaagaagatgaaaaatctgaaaacatcactttcattccagat ggtaatggtgaatttaccgaaggcatgggtatgttagttggtaaagaagatttaggcttc ggtaaacgttcatggcgttattctatgcttgtgaaaaacggcgtagttgaaaaaatgttt atcgaaccaaacgaaccaggcgatccgttcaaagtatccgatgctgacactatgttgaaa taccttgcaccacaacaccaagtgcaagagtctatttcaatctttacaaaacctggctgt cctttctgtgcaaaagcaaaacaacttttacacgataaaggcttaagctttgaagaaatc atattaggtcacgatgcaacaatcgtgagcgtacgtgcagtttcaggtcgtgctactgtt ccacaagtgtttatcggtggtaaacacattggcggtagcgacgatttggaaaaatacttt gcataa |
| A4NBB5 | UniRef cluster | -------------------------------------------KYLAPQHQVQESISIFTK-----PGCPFCA-KAKQLLHDKG---LSFEE----IILG---HDATIVSVRAVS----GRA----------TVPQVFIGGK--------HIGGSDD----LEKYFA--------------------------- | atgtctaatatggaaggaaaaaaagtccctcaagtgacattccgcactcgtcagggtgat aaatgggttgatgtaactacctcagagttatttgataacaaaacagtgatcgtgttctca ttaccgggcgcattcactccaacttgctcatcatcacacttaccacgttacaacgaatta gcgccagtattcaaaaaatacggtgtagacgatattctcgttgtatctgtaaatgatact ttcgtaatgaacgcatggaaagaagatgaaaaatctgaaaacatcactttcattccagat ggtaatggtgaatttaccgaaggcatgggtatgttagttggtaaagaagatttaggcttc ggtaaacgttcatggcgttattctatgcttgtgaaaaacggcgtagttgaaaaaatgttt atcgaaccaaacgaaccaggcgatccgttcaaagtatccgatgctgacactatgttgaaa taccttgcaccacaacaccaagtgcaagagtctatttcaatcttcacaaaacctggctgt cctttctgtgcaaaagcaaaacaacttttacacgataaaggcttaagctttgaagaaatc atattaggtcacgatgcaacaatcgtaagcgtacgtgcagtttcaggtcgtgctactgtt ccacaagtgtttatcggtggtaaacacattggcggtagcgacgatttggaaaaatacttt gcataa |
| A4N6H3 | UniRef cluster | -------------------------------------------KYLAPQHQVQESISIFTK-----PGCPFCA-KAKQLLHDKG---LSFEE----IILG---HDATIVSVRAVS----GRA----------TVPQVFIGGK--------HIGGSDD----LEKYFA--------------------------- | atgtctagtatggaaggaaaaaaagtccctcaagtgacattccgcactcgtcagggtgat aaatgggttgatgtaactacctcagagttatttgataacaaaacagtgatcgtgttctca ttaccgggcgcattcactccaacttgctcatcatcacacttaccacgttacaacgaatta gcgccagtattcaaaaaatacggtgtagatgatattcttgttgtatctgtaaatgatact ttcgtaatgaacgcatggaaagaagatgaaaaatctgaaaacatcactttcattccagat ggtaatggtgaatttaccgaaggcatgggtatgttagttggtaaagaagatttaggcttc ggtaaacgttcatggcgttattctatgcttgtgaaaaacggcgtagttgaaaaaatgttt atcgaaccaaacgaaccaggcgatccgttcaaagtatccgatgctgacactatgttgaaa taccttgcaccacaacaccaagtgcaagagtctatttcaatcttcacaaaacctggctgt cctttctgtgcaaaagcaaaacaacttttacacgataaaggcttaagctttgaagaaatc atattaggtcacgatgcaacaatcgtgagcgtacgtgcagtttcaggtcgtgctactgtt ccacaagtgtttatcggtggtaaacacattggcggtagcgacgatttggaaaaatacttt gcataa |
| A4MYL9 | UniRef cluster | -------------------------------------------KYLAPQHQVQESISIFTK-----PGCPFCA-KAKQLLHDKG---LSFEE----IILG---HDATIVSVRAVS----GRA----------TVPQVFIGGK--------HIGGSDD----LEKYFA--------------------------- | atgtctagtatggaaggaaaaaaagtccctcaagtgacattccgcactcgtcagggtgat aaatgggttgatgtaactacctcagagttatttgataacaaaacagtgatcgtgttctca ttaccgggcgcattcactccaacttgctcatcatcacacttaccacgttacaacgaatta gcaccagtattcaaaaaatacggtgtagacgatattcttgttgtatctgtaaatgatact ttcgtaatgaacgcatggaaagaagatgaaaaatctgaaaacatcactttcattccagat ggtaatggtgaatttaccgaaggcatgggtatgttagttggtaaagaagatttaggcttc ggtaaacgttcatggcgttattctatgcttgtgaaaaacggcgtagttgaaaaaatgttt atcgaaccaaacgaaccaggcgatccgttcaaagtatccgatgctgacactatgttgaaa taccttgcaccacaacaccaagtgcaagagtctatttcaatcttcacaaaacctggctgt cctttctgtgcaaaagcaaaacaacttttacacgataaaggcttaagctttgaagaaatc atattaggtcacgatgcaacaatcgtgagcgtacgtgcagtttcaggtcgtgctactgtt ccacaagtgtttatcggtggtaaacacattggcggtagcgacgatttggaaaaatacttt gcataa |
| Q9KNU3 | UniRef cluster | -------------------------------------------KYIAPQYKVQESVTIFTK-----PGCPYCA-KAKQALIDAG---LQYEE----LILG---KDATTVSLRAVS----GRT----------TVPQVFIGGK--------HIGGSDD----LEVYLNQ-------------------------- | atgaggaacacaatgtttacatctaaagaaggtcaaaccattccacaggttacttttcct actcgccaaggtgacgcttgggtcaatgtgactagcgatgaactgttcaaaggcaaaacc gttatcgtgtttagcttgccgggtgcctttactccaacctgttcatccactcacctaccg cgctacaacgagctgtttcctgtctttaaagagcatggtgtcgacagcattctgtgcgta tcggtcaacgatactttcgtgatgaatgcttggaaagatgaccaaaatgccgacaacatc accttcattcctgatggtaatggtgaatttaccgatggtatgggcatgctggtggataaa aatgaccttggctttggtaaacgctcatggcgctacagcatgctggttaaagacggtgtg gtagaaaaaatgtttatcgaaccgaatgagccgggcgacccgttcaaagtatcggacgcc gataccatgctcaaatacattgcccctcaatacaaggtgcaagaatcagtgactattttc actaagccaggctgtccttattgcgccaaggcgaaacaagcgctgattgatgccggtcta cagtatgaagagctgattttaggtaaagacgctaccacagtgagtctgcgcgccgtttct ggccgtaccacggtgccgcaagtgtttatcggtggtaaacacattggtggtagcgacgac ttagaagtctacctaaatcaataa |
| A6Y1K9 | UniRef cluster | -------------------------------------------KYIAPQYKVQESVTIFTK-----PGCPYCA-KAKQALIDAG---LQYEE----LILG---KDATTVSLRAVS----GRT----------TVPQVFIGGK--------HIGGSDD----LEVYLNQ-------------------------- | atgaggaacacaatgtttacatctaaagaaggtcaaaccattccacaggttacttttcct actcgccaaggtgacgcttgggtcaatgtgactagcgatgagctgttcaaaggcaaaacc gttatcgtgtttagcttgccgggtgcctttactccaacctgttcatccactcacctaccg cgctacaacgagctgtttcctgtctttaaagagcatggtgtcgacagcattctgtgcgta tcggtcaacgatactttcgtgatgaatgcttggaaagatgaccaaaatgccgacaacatc acctttattcctgatggtaatggtgaatttaccgatggtatggggatgctggtggataaa aatgaccttggctttggtaaacgctcatggcgctacagcatgctggttaaagacggtgtg gtagaaaaaatgtttatcgaaccgaatgagccgggcgacccgttcaaagtatcggacgcc gataccatgctcaaatacattgcccctcaatacaaggtgcaagaatcagtgactattttc actaagcctggatgtccttattgcgccaaggcgaaacaagcgctgattgatgccggtcta cagtatgaagagctgattttaggtaaagacgccaccacagtgagcctgcgcgctgtttct ggccgtaccacggtgccgcaagtgtttatcggtggtaaacacattggcggcagcgacgac ttagaagtctacctaaatcaataa |
| A6XU96 | UniRef cluster | -------------------------------------------KYIAPQYKVQESVTIFTK-----PGCPYCA-KAKQALIDAG---LQYEE----LILG---KDATTVSLRAVS----GRT----------TVPQVFIGGK--------HIGGSDD----LEVYLNQ-------------------------- | atgaggaacacaatgtttacatctaaagaaggtcaaaccattccacaggttacttttcct actcgccaaggtgacgcttgggtcaatgtgactagcgatgaactgttcaaaggcaaaacc gttatcgtgtttagcttgccgggtgcctttactccaacctgttcatccactcacctaccg cgctacaacgagctgtttcctgtctttaaagagcatggtgtcgacagcattctgtgcgta tcggtcaacgatactttcgtgatgaatgcttggaaagatgaccaaaatgccgacaacatc accttcattcctgatggtaatggtgaatttaccgatggtatgggcatgttggtggataaa aatgaccttggctttggtaaacgctcatggcgctacagcatgctggttaaagatggtgtg gtagaaaaaatgtttatcgaaccgaatgagccgggcgacccgttcaaagtatcggacgcc gataccatgctcaaatacattgcccctcaatacaaggtgcaagaatcagtgactattttc actaagcctggatgtccttattgcgccaaggcgaaacaagcgctgattgatgccggtcta cagtatgaagagctgattttaggtaaagacgccaccacagtgagcctgcgcgctgtttct ggccgtaccacggtgccgcaagtgtttatcggtggtaaacacattggtggcagcgacgac ttagaagtctacctaaatcaataa |
| A6AFP4 | UniRef cluster | -------------------------------------------KYIAPQYKVQESVTIFTK-----PGCPYCA-KAKQALIDAG---LQYEE----LILG---KDATTVSLRAVS----GRT----------TVPQVFIGGK--------HIGGSDD----LEVYLNQ-------------------------- | atgaggaacacaatgtttacatctaaagaaggtcaaaccattccacaggttacttttcct actcgccaaggtgacgcttgggtcaatgtgactagcgatgaactgttcaaaggcaaaacc gttatcgtgtttagcttgccgggtgcctttactccaacctgttcatccactcacctaccg cgctacaacgagctgtatcctgtctttaaagagaatggtgtcgacagcattctgtgcgta tcggtcaacgatactttcgtgatgaatgcttggaaagatgaccaaaatgccgacaacatc accttcattcctgatggtaatggtgaatttaccgatggtatgggcatgctggtggataaa aatgaccttggctttggtaaacgctcatggcgctacagcatgctggttaaagacggtgtg gtagaaaaaatgtttatcgaaccgaatgagccgggcgacccgttcaaagtatcggacgcc gataccatgctcaaatacattgcccctcaatacaaggtgcaagaatcagtgactattttc actaagccaggctgtccttattgcgccaaggcgaaacaagcgctgattgatgccggtcta cagtatgaagagctgattttaggtaaagacgccaccacagtgagcctgcgcgctgtttct ggccgtaccacggtgccgcaagtgtttatcggtggtaaacacattggtggcagcgacgac ttagaagtctacctaaatcaataa |
| A6A149 | UniRef cluster | -------------------------------------------KYIAPQYKVQESVTIFTK-----PGCPYCA-KAKQALIDAG---LQYEE----LILG---KDATTVSLRAVS----GRT----------TVPQVFIGGK--------HIGGSDD----LEVYLNQ-------------------------- | atgaggaacacaatgtttacatctaaagaaggtcaaaccattccacaggttacttttcct actcgccaaggtgacgcttgggtcaatgtgactagcgatgagctgttcaaaggcaaaacc gttatcgtgtttagcttgccgggtgcctttactccaacctgttcatccactcacctaccg cgctacaacgagctgtttcctgtctttaaagagcatggtgtcgacagcattctgtgcgta tcggtcaacgatactttcgtgatgaatgcttggaaagatgaccaaaacgccgacaacatc accttcattcctgatggtaatggtgaatttaccgatggtatgggcatgttggtggataaa aatgaccttggctttggtaaacgctcatggcgctacagcatgctggttaaagacggtgtg gtagaaaaaatgtttatcgaaccgaatgagccgggcgacccgttcaaagtatcggacgcc gataccatgctcaaatacattgcccctcaatacaaggtgcaagaatcagtgactattttc actaagcctggctgtccttattgcgccaaggcgaaacaagcgctgattgatgccggtcta cagtatgaagagctgattttaggtaaagacgccaccacagtgagcctgcgcgctgtttct ggccgtaccacggtgccgcaagtgtttatcggtggtaaacacattggtggcagcgacgac ttagaagtctacctaaatcaataa |
| A5F510 | UniRef cluster | -------------------------------------------KYIAPQYKVQESVTIFTK-----PGCPYCA-KAKQALIDAG---LQYEE----LILG---KDATTVSLRAVS----GRT----------TVPQVFIGGK--------HIGGSDD----LEVYLNQ-------------------------- | atgaggaacacaatgtttacatctaaagaaggtcaaaccattccacaggttacttttcct actcgccaaggtgacgcttgggtcaatgtgactagcgatgaactgttcaaaggcaaaacc gttatcgtgtttagcttgccgggtgcctttactccaacctgttcatccactcacctaccg cgctacaacgagctgtttcctgtctttaaagagcatggtgtcgacagcattctgtgcgta tcggtcaacgatactttcgtgatgaatgcttggaaagatgaccaaaatgccgacaacatc accttcattcctgatggtaatggtgaatttaccgatggtatgggcatgctggtggataaa aatgaccttggctttggtaaacgctcatggcgctacagcatgctggttaaagacggtgtg gtagaaaaaatgtttatcgaaccgaatgagccgggcgacccgttcaaagtatcggacgcc gataccatgctcaaatacattgcccctcaatacaaggtgcaagaatcagtgactattttc actaagccaggctgtccttattgcgccaaggcgaaacaagcgctgattgatgccggtcta cagtatgaagagctgattttaggtaaagacgctaccacagtgagtctgcgcgccgtttct ggccgtaccacggtgccgcaagtgtttatcggtggtaaacacattggtggtagcgacgac ttagaagtctacctaaatcaataa |
| A3GXC3 | UniRef cluster | -------------------------------------------KYIAPQYKVQESVTIFTK-----PGCPYCA-KAKQALIDAG---LQYEE----LILG---KDATTVSLRAVS----GRT----------TVPQVFIGGK--------HIGGSDD----LEVYLNQ-------------------------- | atgaggaacacaatgtttacatctaaagaaggtcaaaccattccacaggttacttttcct actcgccaaggtgacgcttgggtcaatgtgactagcgatgaactgttcaaaggcaaaacc gttatcgtgtttagcttgccgggtgcctttactccaacctgttcatccactcacctaccg cgctacaacgagctgtttcctgtctttaaagagcatggtgtcgacagcattctgtgcgta tcggtcaacgatactttcgtgatgaatgcttggaaagatgaccaaaatgccgacaacatc accttcattcctgatggtaatggtgaatttaccgatggtatgggcatgctggtggataaa aatgaccttggctttggtaaacgctcatggcgctacagcatgctggttaaagacggtgtg gtagaaaaaatgtttatcgaaccgaatgagccgggcgacccgttcaaagtatcggacgcc gataccatgctcaaatacattgcccctcaatacaaggtgcaagaatcagtgactattttc actaagccaggctgtccttattgcgccaaggcgaaacaagcgctgattgatgccggtcta cagtatgaagagctgattttaggtaaagacgctaccacagtgagtctgcgcgccgtttct ggccgtaccacggtgccgcaagtgtttatcggtggtaaacacattggtggtagcgacgac ttagaagtctacctaaatcaataa |
| A3GRD5 | UniRef cluster | -------------------------------------------KYIAPQYKVQESVTIFTK-----PGCPYCA-KAKQALIDAG---LQYEE----LILG---KDATTVSLRAVS----GRT----------TVPQVFIGGK--------HIGGSDD----LEVYLNQ-------------------------- | atgaggaacacaatgtttacatctaaagaaggtcaaaccattccacaggttacttttcct actcgccaaggtgacgcttgggtcaatgtgactagcgatgaactgttcaaaggcaaaacc gttatcgtgtttagcttgccgggtgcctttactccaacctgttcatccactcacctaccg cgctacaacgagctgtttcctgtctttaaagagcatggtgtcgacagcattctgtgcgta tcggtcaacgatactttcgtgatgaatgcttggaaagatgaccaaaatgccgacaacatc accttcattcctgatggtaatggtgaatttaccgatggtatgggcatgctggtggataaa aatgaccttggctttggtaaacgctcatggcgctacagcatgctggttaaagacggtgtg gtagaaaaaatgtttatcgaaccgaatgagccgggcgacccgttcaaagtatcggacgcc gataccatgctcaaatacattgcccctcaatacaaggtgcaagaatcagtgactattttc actaagccaggctgtccttattgcgccaaggcgaaacaagcgctgattgatgccggtcta cagtatgaagagctgattttaggtaaagacgctaccacagtgagtctgcgcgccgtttct ggccgtaccacggtgccgcaagtgtttatcggtggtaaacacattggtggtagcgacgac ttagaagtctacctaaatcaataa |
| A3EJ81 | UniRef cluster | -------------------------------------------KYIAPQYKVQESVTIFTK-----PGCPYCA-KAKQALIDAG---LQYEE----LILG---KDATTVSLRAVS----GRT----------TVPQVFIGGK--------HIGGSDD----LEVYLNQ-------------------------- | atgaggaacacaatgtttacatctaaagaaggtcaaaccattccacaggttacttttcct actcgccaaggtgacgcttgggtcaatgtgactagcgatgaactgttcaaaggcaaaacc gttatcgtgtttagcttgccgggtgcctttactccaacctgttcatccactcacctaccg cgctacaacgagctgtttcctgtctttaaagagcatggtgtcgacagcattctgtgcgta tcggtcaacgatactttcgtgatgaatgcttggaaagatgaccaaaatgccgacaacatc accttcattcctgatggtaatggtgaatttaccgatggtatgggcatgctggtggataaa aatgaccttggctttggtaaacgctcatggcgctacagcatgctggttaaagacggtgtg gtagaaaaaatgtttatcgaaccgaatgagccgggcgacccgttcaaagtatcggacgcc gataccatgctcaaatacattgcccctcaatacaaggtgcaagaatcagtgactattttc actaagcctggctgtccttattgcgccaaggcgaaacaagcgctgattgatgccggtcta cagtatgaagagctgattttaggtaaagacgccaccacagtgagcctgcgcgctgtttct ggccgtaccacggtgccgcaagtgtttatcggtggtaaacacattggtggcagcgacgac ttagaagtctacctaaatcaataa |
| A3EB97 | UniRef cluster | -------------------------------------------KYIAPQYKVQESVTIFTK-----PGCPYCA-KAKQALIDAG---LQYEE----LILG---KDATTVSLRAVS----GRT----------TVPQVFIGGK--------HIGGSDD----LEVYLNQ-------------------------- | atgaggaacacaatgtttacatctaaagaaggtcaaaccattccacaggttacttttcct actcgccaaggtgacgcttgggtcaatgtgactagcgatgaactgttcaaaggcaaaacc gttatcgtgtttagcttgccgggtgcctttactccaacctgttcatccactcacctaccg cgctacaacgagctgtttcctgtctttaaagagcatggtgtcgacagcattctgtgcgta tcggtcaacgatactttcgtgatgaatgcttggaaagatgaccaaaatgccgacaacatc accttcattcctgatggtaatggtgaatttaccgatggtatgggcatgctggtggataaa aatgaccttggctttggtaaacgctcatggcgctacagcatgctggttaaagacggtgtg gtagaaaaaatgtttatcgaaccgaatgagccgggcgacccgttcaaagtatcggacgcc gataccatgctcaaatacattgcccctcaatacaaggtgcaagaatcagtgactattttc actaagccaggctgtccttattgcgccaaggcgaaacaagcgctgattgatgccggtcta cagtatgaagagctgattttaggtaaagacgctaccacagtgagtctgcgcgccgtttct ggccgtaccacggtgccgcaagtgtttatcggtggtaaacacattggtggtagcgacgac ttagaagtctacctaaatcaataa |
| A2PVZ9 | UniRef cluster | -------------------------------------------KYIAPQYKVQESVTIFTK-----PGCPYCA-KAKQALIDAG---LQYEE----LILG---KDATTVSLRAVS----GRT----------TVPQVFIGGK--------HIGGSDD----LEVYLNQ-------------------------- | atgaggaacacaatgtttacatctaaagaaggtcaaaccattccacaggttacttttcct actcgccaaggtgacgcttgggtcaatgtgactaccgatgaactgttcaaaggcaaaacc gttatcgtgtttagcttgccgggtgcctttactccaacctgttcatccactcacctaccg cgctacaacgagctgtttcctgtctttaaagagcatggtgtcgacagcattctgtgcgta tcggtcaacgatactttcgtgatgaatgcttggaaagatgaccaaaacgccgacaacatc accttcattcctgatggtaatggtgaatttaccgatggtatgggcatgttggtggataaa aatgaccttggctttggtaaacgctcatggcgctacagcatgctggttaaagacggtgtg gtagaaaaaatgtttatcgaaccgaatgagccgggcgacccgttcaaagtatcggacgcc gataccatgctcaaatacattgcccctcaatacaaggtgcaagaatcagtgactattttc actaagccaggctgcccttattgcgccaaggcgaaacaagcgctgattgatgccggtcta cagtatgaagagctgattttaggtaaagacgccaccacagtgagcctgcgcgctgtttct ggccgtaccacggtgccgcaagtgtttatcggtggtaaacacattggtggcagcgacgac ttagaagtctacctaaatcaataa |
| A2PLL7 | UniRef cluster | -------------------------------------------KYIAPQYKVQESVTIFTK-----PGCPYCA-KAKQALIDAG---LQYEE----LILG---KDATTVSLRAVS----GRT----------TVPQVFIGGK--------HIGGSDD----LEVYLNQ-------------------------- | atgaggaacacaatgtttacatctaaagaaggtcaaaccattccacaggttacttttcct actcgccaaggtgacgcttgggtcaatgtgactagcgatgaactgttcaaaggcaaaacc gttatcgtgtttagcttgccgggtgcctttactccaacctgttcatccactcacctaccg cgctacaacgagctgtttcctgtctttaaagagcatggtgtcgacagcattctgtgcgta tcggtcaacgatactttcgtgatgaatgcttggaaagatgaccaaaatgccgacaacatc accttcattcctgatggtaatggtgaatttaccgatggtatgggcatgctggtggataaa aatgaccttggctttggtaaacgctcatggcgctacagcatgctggttaaagacggtgtg gtagaaaaaatgtttatcgaaccgaatgagccgggcgacccgttcaaagtatcggacgcc gataccatgctcaaatacattgcccctcaatacaaggtgcaagaatcagtgactattttc actaagccaggctgtccttattgcgccaaggcgaaacaagcgctgattgatgccggtcta cagtatgaagagctgattttaggtaaagacgctaccacagtgagtctgcgcgccgtttct ggccgtaccacggtgccgcaagtgtttatcggtggtaaacacattggtggtagcgacgac ttagaagtctacctaaatcaataa |
| A2P6Y2 | UniRef cluster | -------------------------------------------KYIAPQYKVQESVTIFTK-----PGCPYCA-KAKQALIDAG---LQYEE----LILG---KDATTVSLRAVS----GRT----------TVPQVFIGGK--------HIGGSDD----LEVYLNQ-------------------------- | atgaggaacacaatgtttacatctaaagaaggtcaaaccattccacaggttacttttcct actcgccaaggtgacgcttgggtcaatgtgactagcgatgagcttttcaaaggcaaaacc gttatcgtgtttagcttgccgggtgcctttactccaacctgttcatccactcacctaccg cgctacaacgagctgtttcctgtctttaaagagcatggtgtcgacagcattctgtgcgta tcggtcaacgatactttcgtgatgaatgcttggaaagatgaccaaaacgccgacaacatc acctttattcctgatggtaatggtgaatttaccgatggtatgggcatgctggtggataaa aatgaccttggctttggtaaacgctcatggcgctacagcatgctggttaaagatggtgtg gtagaaaaaatgtttatcgaaccgaatgagccgggcgacccgttcaaagtatcggacgcc gataccatgctcaaatacattgcccctcaatacaaggtgcaagaatcagtgactattttc actaagcctggatgtccttattgcgccaaggcgaaacaagcgctgattgatgccggtcta cagtatgaagagctgattttaggtaaagacgccaccacagtgagcctgcgcgctgtttct ggccgtaccacggtgccgcaagtgtttatcggtggtaaacacattggtggcagcgacgac ttagaagtctacctaaatcaataa |
| A1F753 | UniRef cluster | -------------------------------------------KYIAPQYKVQESVTIFTK-----PGCPYCA-KAKQALIDAG---LQYEE----LILG---KDATTVSLRAVS----GRT----------TVPQVFIGGK--------HIGGSDD----LEVYLNQ-------------------------- | atgaggaacacaatgtttacatctaaagaaggtcaaaccattccacaggttacttttcct actcgccaaggtgacgcttgggtcaatgtgactagcgatgaactgttcaaaggcaaaacc gttatcgtgtttagcttgccgggtgcctttactccaacctgttcatccactcacctaccg cgctacaacgagctgtttcctgtctttaaagagcatggtgtcgacagcattctgtgcgta tcggtcaacgatactttcgtgatgaatgcttggaaagatgaccaaaatgccgacaacatc accttcattcctgatggtaatggtgaatttaccgatggtatgggcatgctggtggataaa aatgaccttggctttggtaaacgctcatggcgctacagcatgctggttaaagacggtgtg gtagaaaaaatgtttatcgaaccgaatgagccgggcgacccgttcaaagtatcggacgcc gataccatgctcaaatacattgcccctcaatacaaggtgcaagaatcagtgactattttc actaagccaggctgtccttattgcgccaaggcgaaacaagcgctgattgatgccggtcta cagtatgaagagctgattttaggtaaagacgctaccacagtgagtctgcgcgccgtttct ggccgtaccacggtgccgcaagtgtttatcggtggtaaacacattggtggtagcgacgac ttagaagtctacctaaatcaataa |
| A1EMB6 | UniRef cluster | -------------------------------------------KYIAPQYKVQESVTIFTK-----PGCPYCA-KAKQALIDAG---LQYEE----LILG---KDATTVSLRAVS----GRT----------TVPQVFIGGK--------HIGGSDD----LEVYLNQ-------------------------- | atgaggaacacaatgtttacatctaaagaaggtcaaaccattccacaggttacttttcct actcgccaaggtgacgcttgggtcaatgtgactagcgatgaactgttcaaaggcaaaacc gttatcgtgtttagcttgccgggtgcctttactccaacctgttcatccactcacctaccg cgctacaacgagctgtttcctgtctttaaagagcatggtgtcgacagcattctgtgcgta tcggtcaacgatactttcgtgatgaatgcttggaaagatgaccaaaatgccgacaacatc accttcattcctgatggtaatggtgaatttaccgatggtatgggcatgctggtggataaa aatgaccttggctttggtaaacgctcatggcgctacagcatgctggttaaagatggtgtg gtagaaaaaatgtttatcgaaccgaatgagccgggcgacccgttcaaagtatcggacgcc gataccatgctcaaatacattgcccctcaatacaaggtgcaagaatcagtgactattttc actaagcctggatgtccttattgcgccaaggcgaaacaagcgctgattgatgccggtcta cagtatgaagagctgattttaggtaaagacgccaccacagtgagcctgcgcgctgtttct ggccgtaccacggtgccgcaagtgtttatcggtggtaaacacattggtggcagcgacgac ttagaagtctacctaaatcaataa |
| Q9KNU3 | UniRef cluster | -------------------------------------------KYIAPQYKVQESVTIFTK-----PGCPYCA-KAKQALIDAG---LQYEE----LILG---KDATTVSLRAVS----GRT----------TVPQVFIGGK--------HIGGSDD----LEVYLNQ-------------------------- | atgaggaacacaatgtttacatctaaagaaggtcaaaccattccacaggttacttttcct actcgccaaggtgacgcttgggtcaatgtgactagcgatgaactgttcaaaggcaaaacc gttatcgtgtttagcttgccgggtgcctttactccaacctgttcatccactcacctaccg cgctacaacgagctgtttcctgtctttaaagagcatggtgtcgacagcattctgtgcgta tcggtcaacgatactttcgtgatgaatgcttggaaagatgaccaaaatgccgacaacatc accttcattcctgatggtaatggtgaatttaccgatggtatgggcatgctggtggataaa aatgaccttggctttggtaaacgctcatggcgctacagcatgctggttaaagacggtgtg gtagaaaaaatgtttatcgaaccgaatgagccgggcgacccgttcaaagtatcggacgcc gataccatgctcaaatacattgcccctcaatacaaggtgcaagaatcagtgactattttc actaagccaggctgtccttattgcgccaaggcgaaacaagcgctgattgatgccggtcta cagtatgaagagctgattttaggtaaagacgctaccacagtgagtctgcgcgccgtttct ggccgtaccacggtgccgcaagtgtttatcggtggtaaacacattggtggtagcgacgac ttagaagtctacctaaatcaataa |
| A6Y1K9 | UniRef cluster | -------------------------------------------KYIAPQYKVQESVTIFTK-----PGCPYCA-KAKQALIDAG---LQYEE----LILG---KDATTVSLRAVS----GRT----------TVPQVFIGGK--------HIGGSDD----LEVYLNQ-------------------------- | atgaggaacacaatgtttacatctaaagaaggtcaaaccattccacaggttacttttcct actcgccaaggtgacgcttgggtcaatgtgactagcgatgagctgttcaaaggcaaaacc gttatcgtgtttagcttgccgggtgcctttactccaacctgttcatccactcacctaccg cgctacaacgagctgtttcctgtctttaaagagcatggtgtcgacagcattctgtgcgta tcggtcaacgatactttcgtgatgaatgcttggaaagatgaccaaaatgccgacaacatc acctttattcctgatggtaatggtgaatttaccgatggtatggggatgctggtggataaa aatgaccttggctttggtaaacgctcatggcgctacagcatgctggttaaagacggtgtg gtagaaaaaatgtttatcgaaccgaatgagccgggcgacccgttcaaagtatcggacgcc gataccatgctcaaatacattgcccctcaatacaaggtgcaagaatcagtgactattttc actaagcctggatgtccttattgcgccaaggcgaaacaagcgctgattgatgccggtcta cagtatgaagagctgattttaggtaaagacgccaccacagtgagcctgcgcgctgtttct ggccgtaccacggtgccgcaagtgtttatcggtggtaaacacattggcggcagcgacgac ttagaagtctacctaaatcaataa |
| A6XU96 | UniRef cluster | -------------------------------------------KYIAPQYKVQESVTIFTK-----PGCPYCA-KAKQALIDAG---LQYEE----LILG---KDATTVSLRAVS----GRT----------TVPQVFIGGK--------HIGGSDD----LEVYLNQ-------------------------- | atgaggaacacaatgtttacatctaaagaaggtcaaaccattccacaggttacttttcct actcgccaaggtgacgcttgggtcaatgtgactagcgatgaactgttcaaaggcaaaacc gttatcgtgtttagcttgccgggtgcctttactccaacctgttcatccactcacctaccg cgctacaacgagctgtttcctgtctttaaagagcatggtgtcgacagcattctgtgcgta tcggtcaacgatactttcgtgatgaatgcttggaaagatgaccaaaatgccgacaacatc accttcattcctgatggtaatggtgaatttaccgatggtatgggcatgttggtggataaa aatgaccttggctttggtaaacgctcatggcgctacagcatgctggttaaagatggtgtg gtagaaaaaatgtttatcgaaccgaatgagccgggcgacccgttcaaagtatcggacgcc gataccatgctcaaatacattgcccctcaatacaaggtgcaagaatcagtgactattttc actaagcctggatgtccttattgcgccaaggcgaaacaagcgctgattgatgccggtcta cagtatgaagagctgattttaggtaaagacgccaccacagtgagcctgcgcgctgtttct ggccgtaccacggtgccgcaagtgtttatcggtggtaaacacattggtggcagcgacgac ttagaagtctacctaaatcaataa |
| A6AFP4 | UniRef cluster | -------------------------------------------KYIAPQYKVQESVTIFTK-----PGCPYCA-KAKQALIDAG---LQYEE----LILG---KDATTVSLRAVS----GRT----------TVPQVFIGGK--------HIGGSDD----LEVYLNQ-------------------------- | atgaggaacacaatgtttacatctaaagaaggtcaaaccattccacaggttacttttcct actcgccaaggtgacgcttgggtcaatgtgactagcgatgaactgttcaaaggcaaaacc gttatcgtgtttagcttgccgggtgcctttactccaacctgttcatccactcacctaccg cgctacaacgagctgtatcctgtctttaaagagaatggtgtcgacagcattctgtgcgta tcggtcaacgatactttcgtgatgaatgcttggaaagatgaccaaaatgccgacaacatc accttcattcctgatggtaatggtgaatttaccgatggtatgggcatgctggtggataaa aatgaccttggctttggtaaacgctcatggcgctacagcatgctggttaaagacggtgtg gtagaaaaaatgtttatcgaaccgaatgagccgggcgacccgttcaaagtatcggacgcc gataccatgctcaaatacattgcccctcaatacaaggtgcaagaatcagtgactattttc actaagccaggctgtccttattgcgccaaggcgaaacaagcgctgattgatgccggtcta cagtatgaagagctgattttaggtaaagacgccaccacagtgagcctgcgcgctgtttct ggccgtaccacggtgccgcaagtgtttatcggtggtaaacacattggtggcagcgacgac ttagaagtctacctaaatcaataa |
| A6A149 | UniRef cluster | -------------------------------------------KYIAPQYKVQESVTIFTK-----PGCPYCA-KAKQALIDAG---LQYEE----LILG---KDATTVSLRAVS----GRT----------TVPQVFIGGK--------HIGGSDD----LEVYLNQ-------------------------- | atgaggaacacaatgtttacatctaaagaaggtcaaaccattccacaggttacttttcct actcgccaaggtgacgcttgggtcaatgtgactagcgatgagctgttcaaaggcaaaacc gttatcgtgtttagcttgccgggtgcctttactccaacctgttcatccactcacctaccg cgctacaacgagctgtttcctgtctttaaagagcatggtgtcgacagcattctgtgcgta tcggtcaacgatactttcgtgatgaatgcttggaaagatgaccaaaacgccgacaacatc accttcattcctgatggtaatggtgaatttaccgatggtatgggcatgttggtggataaa aatgaccttggctttggtaaacgctcatggcgctacagcatgctggttaaagacggtgtg gtagaaaaaatgtttatcgaaccgaatgagccgggcgacccgttcaaagtatcggacgcc gataccatgctcaaatacattgcccctcaatacaaggtgcaagaatcagtgactattttc actaagcctggctgtccttattgcgccaaggcgaaacaagcgctgattgatgccggtcta cagtatgaagagctgattttaggtaaagacgccaccacagtgagcctgcgcgctgtttct ggccgtaccacggtgccgcaagtgtttatcggtggtaaacacattggtggcagcgacgac ttagaagtctacctaaatcaataa |
| A5F510 | UniRef cluster | -------------------------------------------KYIAPQYKVQESVTIFTK-----PGCPYCA-KAKQALIDAG---LQYEE----LILG---KDATTVSLRAVS----GRT----------TVPQVFIGGK--------HIGGSDD----LEVYLNQ-------------------------- | atgaggaacacaatgtttacatctaaagaaggtcaaaccattccacaggttacttttcct actcgccaaggtgacgcttgggtcaatgtgactagcgatgaactgttcaaaggcaaaacc gttatcgtgtttagcttgccgggtgcctttactccaacctgttcatccactcacctaccg cgctacaacgagctgtttcctgtctttaaagagcatggtgtcgacagcattctgtgcgta tcggtcaacgatactttcgtgatgaatgcttggaaagatgaccaaaatgccgacaacatc accttcattcctgatggtaatggtgaatttaccgatggtatgggcatgctggtggataaa aatgaccttggctttggtaaacgctcatggcgctacagcatgctggttaaagacggtgtg gtagaaaaaatgtttatcgaaccgaatgagccgggcgacccgttcaaagtatcggacgcc gataccatgctcaaatacattgcccctcaatacaaggtgcaagaatcagtgactattttc actaagccaggctgtccttattgcgccaaggcgaaacaagcgctgattgatgccggtcta cagtatgaagagctgattttaggtaaagacgctaccacagtgagtctgcgcgccgtttct ggccgtaccacggtgccgcaagtgtttatcggtggtaaacacattggtggtagcgacgac ttagaagtctacctaaatcaataa |
| A3GXC3 | UniRef cluster | -------------------------------------------KYIAPQYKVQESVTIFTK-----PGCPYCA-KAKQALIDAG---LQYEE----LILG---KDATTVSLRAVS----GRT----------TVPQVFIGGK--------HIGGSDD----LEVYLNQ-------------------------- | atgaggaacacaatgtttacatctaaagaaggtcaaaccattccacaggttacttttcct actcgccaaggtgacgcttgggtcaatgtgactagcgatgaactgttcaaaggcaaaacc gttatcgtgtttagcttgccgggtgcctttactccaacctgttcatccactcacctaccg cgctacaacgagctgtttcctgtctttaaagagcatggtgtcgacagcattctgtgcgta tcggtcaacgatactttcgtgatgaatgcttggaaagatgaccaaaatgccgacaacatc accttcattcctgatggtaatggtgaatttaccgatggtatgggcatgctggtggataaa aatgaccttggctttggtaaacgctcatggcgctacagcatgctggttaaagacggtgtg gtagaaaaaatgtttatcgaaccgaatgagccgggcgacccgttcaaagtatcggacgcc gataccatgctcaaatacattgcccctcaatacaaggtgcaagaatcagtgactattttc actaagccaggctgtccttattgcgccaaggcgaaacaagcgctgattgatgccggtcta cagtatgaagagctgattttaggtaaagacgctaccacagtgagtctgcgcgccgtttct ggccgtaccacggtgccgcaagtgtttatcggtggtaaacacattggtggtagcgacgac ttagaagtctacctaaatcaataa |
| A3GRD5 | UniRef cluster | -------------------------------------------KYIAPQYKVQESVTIFTK-----PGCPYCA-KAKQALIDAG---LQYEE----LILG---KDATTVSLRAVS----GRT----------TVPQVFIGGK--------HIGGSDD----LEVYLNQ-------------------------- | atgaggaacacaatgtttacatctaaagaaggtcaaaccattccacaggttacttttcct actcgccaaggtgacgcttgggtcaatgtgactagcgatgaactgttcaaaggcaaaacc gttatcgtgtttagcttgccgggtgcctttactccaacctgttcatccactcacctaccg cgctacaacgagctgtttcctgtctttaaagagcatggtgtcgacagcattctgtgcgta tcggtcaacgatactttcgtgatgaatgcttggaaagatgaccaaaatgccgacaacatc accttcattcctgatggtaatggtgaatttaccgatggtatgggcatgctggtggataaa aatgaccttggctttggtaaacgctcatggcgctacagcatgctggttaaagacggtgtg gtagaaaaaatgtttatcgaaccgaatgagccgggcgacccgttcaaagtatcggacgcc gataccatgctcaaatacattgcccctcaatacaaggtgcaagaatcagtgactattttc actaagccaggctgtccttattgcgccaaggcgaaacaagcgctgattgatgccggtcta cagtatgaagagctgattttaggtaaagacgctaccacagtgagtctgcgcgccgtttct ggccgtaccacggtgccgcaagtgtttatcggtggtaaacacattggtggtagcgacgac ttagaagtctacctaaatcaataa |
| A3EJ81 | UniRef cluster | -------------------------------------------KYIAPQYKVQESVTIFTK-----PGCPYCA-KAKQALIDAG---LQYEE----LILG---KDATTVSLRAVS----GRT----------TVPQVFIGGK--------HIGGSDD----LEVYLNQ-------------------------- | atgaggaacacaatgtttacatctaaagaaggtcaaaccattccacaggttacttttcct actcgccaaggtgacgcttgggtcaatgtgactagcgatgaactgttcaaaggcaaaacc gttatcgtgtttagcttgccgggtgcctttactccaacctgttcatccactcacctaccg cgctacaacgagctgtttcctgtctttaaagagcatggtgtcgacagcattctgtgcgta tcggtcaacgatactttcgtgatgaatgcttggaaagatgaccaaaatgccgacaacatc accttcattcctgatggtaatggtgaatttaccgatggtatgggcatgctggtggataaa aatgaccttggctttggtaaacgctcatggcgctacagcatgctggttaaagacggtgtg gtagaaaaaatgtttatcgaaccgaatgagccgggcgacccgttcaaagtatcggacgcc gataccatgctcaaatacattgcccctcaatacaaggtgcaagaatcagtgactattttc actaagcctggctgtccttattgcgccaaggcgaaacaagcgctgattgatgccggtcta cagtatgaagagctgattttaggtaaagacgccaccacagtgagcctgcgcgctgtttct ggccgtaccacggtgccgcaagtgtttatcggtggtaaacacattggtggcagcgacgac ttagaagtctacctaaatcaataa |
| A3EB97 | UniRef cluster | -------------------------------------------KYIAPQYKVQESVTIFTK-----PGCPYCA-KAKQALIDAG---LQYEE----LILG---KDATTVSLRAVS----GRT----------TVPQVFIGGK--------HIGGSDD----LEVYLNQ-------------------------- | atgaggaacacaatgtttacatctaaagaaggtcaaaccattccacaggttacttttcct actcgccaaggtgacgcttgggtcaatgtgactagcgatgaactgttcaaaggcaaaacc gttatcgtgtttagcttgccgggtgcctttactccaacctgttcatccactcacctaccg cgctacaacgagctgtttcctgtctttaaagagcatggtgtcgacagcattctgtgcgta tcggtcaacgatactttcgtgatgaatgcttggaaagatgaccaaaatgccgacaacatc accttcattcctgatggtaatggtgaatttaccgatggtatgggcatgctggtggataaa aatgaccttggctttggtaaacgctcatggcgctacagcatgctggttaaagacggtgtg gtagaaaaaatgtttatcgaaccgaatgagccgggcgacccgttcaaagtatcggacgcc gataccatgctcaaatacattgcccctcaatacaaggtgcaagaatcagtgactattttc actaagccaggctgtccttattgcgccaaggcgaaacaagcgctgattgatgccggtcta cagtatgaagagctgattttaggtaaagacgctaccacagtgagtctgcgcgccgtttct ggccgtaccacggtgccgcaagtgtttatcggtggtaaacacattggtggtagcgacgac ttagaagtctacctaaatcaataa |
| A2PVZ9 | UniRef cluster | -------------------------------------------KYIAPQYKVQESVTIFTK-----PGCPYCA-KAKQALIDAG---LQYEE----LILG---KDATTVSLRAVS----GRT----------TVPQVFIGGK--------HIGGSDD----LEVYLNQ-------------------------- | atgaggaacacaatgtttacatctaaagaaggtcaaaccattccacaggttacttttcct actcgccaaggtgacgcttgggtcaatgtgactaccgatgaactgttcaaaggcaaaacc gttatcgtgtttagcttgccgggtgcctttactccaacctgttcatccactcacctaccg cgctacaacgagctgtttcctgtctttaaagagcatggtgtcgacagcattctgtgcgta tcggtcaacgatactttcgtgatgaatgcttggaaagatgaccaaaacgccgacaacatc accttcattcctgatggtaatggtgaatttaccgatggtatgggcatgttggtggataaa aatgaccttggctttggtaaacgctcatggcgctacagcatgctggttaaagacggtgtg gtagaaaaaatgtttatcgaaccgaatgagccgggcgacccgttcaaagtatcggacgcc gataccatgctcaaatacattgcccctcaatacaaggtgcaagaatcagtgactattttc actaagccaggctgcccttattgcgccaaggcgaaacaagcgctgattgatgccggtcta cagtatgaagagctgattttaggtaaagacgccaccacagtgagcctgcgcgctgtttct ggccgtaccacggtgccgcaagtgtttatcggtggtaaacacattggtggcagcgacgac ttagaagtctacctaaatcaataa |
| A2PLL7 | UniRef cluster | -------------------------------------------KYIAPQYKVQESVTIFTK-----PGCPYCA-KAKQALIDAG---LQYEE----LILG---KDATTVSLRAVS----GRT----------TVPQVFIGGK--------HIGGSDD----LEVYLNQ-------------------------- | atgaggaacacaatgtttacatctaaagaaggtcaaaccattccacaggttacttttcct actcgccaaggtgacgcttgggtcaatgtgactagcgatgaactgttcaaaggcaaaacc gttatcgtgtttagcttgccgggtgcctttactccaacctgttcatccactcacctaccg cgctacaacgagctgtttcctgtctttaaagagcatggtgtcgacagcattctgtgcgta tcggtcaacgatactttcgtgatgaatgcttggaaagatgaccaaaatgccgacaacatc accttcattcctgatggtaatggtgaatttaccgatggtatgggcatgctggtggataaa aatgaccttggctttggtaaacgctcatggcgctacagcatgctggttaaagacggtgtg gtagaaaaaatgtttatcgaaccgaatgagccgggcgacccgttcaaagtatcggacgcc gataccatgctcaaatacattgcccctcaatacaaggtgcaagaatcagtgactattttc actaagccaggctgtccttattgcgccaaggcgaaacaagcgctgattgatgccggtcta cagtatgaagagctgattttaggtaaagacgctaccacagtgagtctgcgcgccgtttct ggccgtaccacggtgccgcaagtgtttatcggtggtaaacacattggtggtagcgacgac ttagaagtctacctaaatcaataa |
| A2P6Y2 | UniRef cluster | -------------------------------------------KYIAPQYKVQESVTIFTK-----PGCPYCA-KAKQALIDAG---LQYEE----LILG---KDATTVSLRAVS----GRT----------TVPQVFIGGK--------HIGGSDD----LEVYLNQ-------------------------- | atgaggaacacaatgtttacatctaaagaaggtcaaaccattccacaggttacttttcct actcgccaaggtgacgcttgggtcaatgtgactagcgatgagcttttcaaaggcaaaacc gttatcgtgtttagcttgccgggtgcctttactccaacctgttcatccactcacctaccg cgctacaacgagctgtttcctgtctttaaagagcatggtgtcgacagcattctgtgcgta tcggtcaacgatactttcgtgatgaatgcttggaaagatgaccaaaacgccgacaacatc acctttattcctgatggtaatggtgaatttaccgatggtatgggcatgctggtggataaa aatgaccttggctttggtaaacgctcatggcgctacagcatgctggttaaagatggtgtg gtagaaaaaatgtttatcgaaccgaatgagccgggcgacccgttcaaagtatcggacgcc gataccatgctcaaatacattgcccctcaatacaaggtgcaagaatcagtgactattttc actaagcctggatgtccttattgcgccaaggcgaaacaagcgctgattgatgccggtcta cagtatgaagagctgattttaggtaaagacgccaccacagtgagcctgcgcgctgtttct ggccgtaccacggtgccgcaagtgtttatcggtggtaaacacattggtggcagcgacgac ttagaagtctacctaaatcaataa |
| A1F753 | UniRef cluster | -------------------------------------------KYIAPQYKVQESVTIFTK-----PGCPYCA-KAKQALIDAG---LQYEE----LILG---KDATTVSLRAVS----GRT----------TVPQVFIGGK--------HIGGSDD----LEVYLNQ-------------------------- | atgaggaacacaatgtttacatctaaagaaggtcaaaccattccacaggttacttttcct actcgccaaggtgacgcttgggtcaatgtgactagcgatgaactgttcaaaggcaaaacc gttatcgtgtttagcttgccgggtgcctttactccaacctgttcatccactcacctaccg cgctacaacgagctgtttcctgtctttaaagagcatggtgtcgacagcattctgtgcgta tcggtcaacgatactttcgtgatgaatgcttggaaagatgaccaaaatgccgacaacatc accttcattcctgatggtaatggtgaatttaccgatggtatgggcatgctggtggataaa aatgaccttggctttggtaaacgctcatggcgctacagcatgctggttaaagacggtgtg gtagaaaaaatgtttatcgaaccgaatgagccgggcgacccgttcaaagtatcggacgcc gataccatgctcaaatacattgcccctcaatacaaggtgcaagaatcagtgactattttc actaagccaggctgtccttattgcgccaaggcgaaacaagcgctgattgatgccggtcta cagtatgaagagctgattttaggtaaagacgctaccacagtgagtctgcgcgccgtttct ggccgtaccacggtgccgcaagtgtttatcggtggtaaacacattggtggtagcgacgac ttagaagtctacctaaatcaataa |
| A1EMB6 | UniRef cluster | -------------------------------------------KYIAPQYKVQESVTIFTK-----PGCPYCA-KAKQALIDAG---LQYEE----LILG---KDATTVSLRAVS----GRT----------TVPQVFIGGK--------HIGGSDD----LEVYLNQ-------------------------- | atgaggaacacaatgtttacatctaaagaaggtcaaaccattccacaggttacttttcct actcgccaaggtgacgcttgggtcaatgtgactagcgatgaactgttcaaaggcaaaacc gttatcgtgtttagcttgccgggtgcctttactccaacctgttcatccactcacctaccg cgctacaacgagctgtttcctgtctttaaagagcatggtgtcgacagcattctgtgcgta tcggtcaacgatactttcgtgatgaatgcttggaaagatgaccaaaatgccgacaacatc accttcattcctgatggtaatggtgaatttaccgatggtatgggcatgctggtggataaa aatgaccttggctttggtaaacgctcatggcgctacagcatgctggttaaagatggtgtg gtagaaaaaatgtttatcgaaccgaatgagccgggcgacccgttcaaagtatcggacgcc gataccatgctcaaatacattgcccctcaatacaaggtgcaagaatcagtgactattttc actaagcctggatgtccttattgcgccaaggcgaaacaagcgctgattgatgccggtcta cagtatgaagagctgattttaggtaaagacgccaccacagtgagcctgcgcgctgtttct ggccgtaccacggtgccgcaagtgtttatcggtggtaaacacattggtggcagcgacgac ttagaagtctacctaaatcaataa |
| Q9KNU3 | UniRef cluster | -------------------------------------------KYIAPQYKVQESVTIFTK-----PGCPYCA-KAKQALIDAG---LQYEE----LILG---KDATTVSLRAVS----GRT----------TVPQVFIGGK--------HIGGSDD----LEVYLNQ-------------------------- | atgaggaacacaatgtttacatctaaagaaggtcaaaccattccacaggttacttttcct actcgccaaggtgacgcttgggtcaatgtgactagcgatgaactgttcaaaggcaaaacc gttatcgtgtttagcttgccgggtgcctttactccaacctgttcatccactcacctaccg cgctacaacgagctgtttcctgtctttaaagagcatggtgtcgacagcattctgtgcgta tcggtcaacgatactttcgtgatgaatgcttggaaagatgaccaaaatgccgacaacatc accttcattcctgatggtaatggtgaatttaccgatggtatgggcatgctggtggataaa aatgaccttggctttggtaaacgctcatggcgctacagcatgctggttaaagacggtgtg gtagaaaaaatgtttatcgaaccgaatgagccgggcgacccgttcaaagtatcggacgcc gataccatgctcaaatacattgcccctcaatacaaggtgcaagaatcagtgactattttc actaagccaggctgtccttattgcgccaaggcgaaacaagcgctgattgatgccggtcta cagtatgaagagctgattttaggtaaagacgctaccacagtgagtctgcgcgccgtttct ggccgtaccacggtgccgcaagtgtttatcggtggtaaacacattggtggtagcgacgac ttagaagtctacctaaatcaataa |
| A6Y1K9 | UniRef cluster | -------------------------------------------KYIAPQYKVQESVTIFTK-----PGCPYCA-KAKQALIDAG---LQYEE----LILG---KDATTVSLRAVS----GRT----------TVPQVFIGGK--------HIGGSDD----LEVYLNQ-------------------------- | atgaggaacacaatgtttacatctaaagaaggtcaaaccattccacaggttacttttcct actcgccaaggtgacgcttgggtcaatgtgactagcgatgagctgttcaaaggcaaaacc gttatcgtgtttagcttgccgggtgcctttactccaacctgttcatccactcacctaccg cgctacaacgagctgtttcctgtctttaaagagcatggtgtcgacagcattctgtgcgta tcggtcaacgatactttcgtgatgaatgcttggaaagatgaccaaaatgccgacaacatc acctttattcctgatggtaatggtgaatttaccgatggtatggggatgctggtggataaa aatgaccttggctttggtaaacgctcatggcgctacagcatgctggttaaagacggtgtg gtagaaaaaatgtttatcgaaccgaatgagccgggcgacccgttcaaagtatcggacgcc gataccatgctcaaatacattgcccctcaatacaaggtgcaagaatcagtgactattttc actaagcctggatgtccttattgcgccaaggcgaaacaagcgctgattgatgccggtcta cagtatgaagagctgattttaggtaaagacgccaccacagtgagcctgcgcgctgtttct ggccgtaccacggtgccgcaagtgtttatcggtggtaaacacattggcggcagcgacgac ttagaagtctacctaaatcaataa |
| A6XU96 | UniRef cluster | -------------------------------------------KYIAPQYKVQESVTIFTK-----PGCPYCA-KAKQALIDAG---LQYEE----LILG---KDATTVSLRAVS----GRT----------TVPQVFIGGK--------HIGGSDD----LEVYLNQ-------------------------- | atgaggaacacaatgtttacatctaaagaaggtcaaaccattccacaggttacttttcct actcgccaaggtgacgcttgggtcaatgtgactagcgatgaactgttcaaaggcaaaacc gttatcgtgtttagcttgccgggtgcctttactccaacctgttcatccactcacctaccg cgctacaacgagctgtttcctgtctttaaagagcatggtgtcgacagcattctgtgcgta tcggtcaacgatactttcgtgatgaatgcttggaaagatgaccaaaatgccgacaacatc accttcattcctgatggtaatggtgaatttaccgatggtatgggcatgttggtggataaa aatgaccttggctttggtaaacgctcatggcgctacagcatgctggttaaagatggtgtg gtagaaaaaatgtttatcgaaccgaatgagccgggcgacccgttcaaagtatcggacgcc gataccatgctcaaatacattgcccctcaatacaaggtgcaagaatcagtgactattttc actaagcctggatgtccttattgcgccaaggcgaaacaagcgctgattgatgccggtcta cagtatgaagagctgattttaggtaaagacgccaccacagtgagcctgcgcgctgtttct ggccgtaccacggtgccgcaagtgtttatcggtggtaaacacattggtggcagcgacgac ttagaagtctacctaaatcaataa |
| A6AFP4 | UniRef cluster | -------------------------------------------KYIAPQYKVQESVTIFTK-----PGCPYCA-KAKQALIDAG---LQYEE----LILG---KDATTVSLRAVS----GRT----------TVPQVFIGGK--------HIGGSDD----LEVYLNQ-------------------------- | atgaggaacacaatgtttacatctaaagaaggtcaaaccattccacaggttacttttcct actcgccaaggtgacgcttgggtcaatgtgactagcgatgaactgttcaaaggcaaaacc gttatcgtgtttagcttgccgggtgcctttactccaacctgttcatccactcacctaccg cgctacaacgagctgtatcctgtctttaaagagaatggtgtcgacagcattctgtgcgta tcggtcaacgatactttcgtgatgaatgcttggaaagatgaccaaaatgccgacaacatc accttcattcctgatggtaatggtgaatttaccgatggtatgggcatgctggtggataaa aatgaccttggctttggtaaacgctcatggcgctacagcatgctggttaaagacggtgtg gtagaaaaaatgtttatcgaaccgaatgagccgggcgacccgttcaaagtatcggacgcc gataccatgctcaaatacattgcccctcaatacaaggtgcaagaatcagtgactattttc actaagccaggctgtccttattgcgccaaggcgaaacaagcgctgattgatgccggtcta cagtatgaagagctgattttaggtaaagacgccaccacagtgagcctgcgcgctgtttct ggccgtaccacggtgccgcaagtgtttatcggtggtaaacacattggtggcagcgacgac ttagaagtctacctaaatcaataa |
| A6A149 | UniRef cluster | -------------------------------------------KYIAPQYKVQESVTIFTK-----PGCPYCA-KAKQALIDAG---LQYEE----LILG---KDATTVSLRAVS----GRT----------TVPQVFIGGK--------HIGGSDD----LEVYLNQ-------------------------- | atgaggaacacaatgtttacatctaaagaaggtcaaaccattccacaggttacttttcct actcgccaaggtgacgcttgggtcaatgtgactagcgatgagctgttcaaaggcaaaacc gttatcgtgtttagcttgccgggtgcctttactccaacctgttcatccactcacctaccg cgctacaacgagctgtttcctgtctttaaagagcatggtgtcgacagcattctgtgcgta tcggtcaacgatactttcgtgatgaatgcttggaaagatgaccaaaacgccgacaacatc accttcattcctgatggtaatggtgaatttaccgatggtatgggcatgttggtggataaa aatgaccttggctttggtaaacgctcatggcgctacagcatgctggttaaagacggtgtg gtagaaaaaatgtttatcgaaccgaatgagccgggcgacccgttcaaagtatcggacgcc gataccatgctcaaatacattgcccctcaatacaaggtgcaagaatcagtgactattttc actaagcctggctgtccttattgcgccaaggcgaaacaagcgctgattgatgccggtcta cagtatgaagagctgattttaggtaaagacgccaccacagtgagcctgcgcgctgtttct ggccgtaccacggtgccgcaagtgtttatcggtggtaaacacattggtggcagcgacgac ttagaagtctacctaaatcaataa |
| A5F510 | UniRef cluster | -------------------------------------------KYIAPQYKVQESVTIFTK-----PGCPYCA-KAKQALIDAG---LQYEE----LILG---KDATTVSLRAVS----GRT----------TVPQVFIGGK--------HIGGSDD----LEVYLNQ-------------------------- | atgaggaacacaatgtttacatctaaagaaggtcaaaccattccacaggttacttttcct actcgccaaggtgacgcttgggtcaatgtgactagcgatgaactgttcaaaggcaaaacc gttatcgtgtttagcttgccgggtgcctttactccaacctgttcatccactcacctaccg cgctacaacgagctgtttcctgtctttaaagagcatggtgtcgacagcattctgtgcgta tcggtcaacgatactttcgtgatgaatgcttggaaagatgaccaaaatgccgacaacatc accttcattcctgatggtaatggtgaatttaccgatggtatgggcatgctggtggataaa aatgaccttggctttggtaaacgctcatggcgctacagcatgctggttaaagacggtgtg gtagaaaaaatgtttatcgaaccgaatgagccgggcgacccgttcaaagtatcggacgcc gataccatgctcaaatacattgcccctcaatacaaggtgcaagaatcagtgactattttc actaagccaggctgtccttattgcgccaaggcgaaacaagcgctgattgatgccggtcta cagtatgaagagctgattttaggtaaagacgctaccacagtgagtctgcgcgccgtttct ggccgtaccacggtgccgcaagtgtttatcggtggtaaacacattggtggtagcgacgac ttagaagtctacctaaatcaataa |
| A3GXC3 | UniRef cluster | -------------------------------------------KYIAPQYKVQESVTIFTK-----PGCPYCA-KAKQALIDAG---LQYEE----LILG---KDATTVSLRAVS----GRT----------TVPQVFIGGK--------HIGGSDD----LEVYLNQ-------------------------- | atgaggaacacaatgtttacatctaaagaaggtcaaaccattccacaggttacttttcct actcgccaaggtgacgcttgggtcaatgtgactagcgatgaactgttcaaaggcaaaacc gttatcgtgtttagcttgccgggtgcctttactccaacctgttcatccactcacctaccg cgctacaacgagctgtttcctgtctttaaagagcatggtgtcgacagcattctgtgcgta tcggtcaacgatactttcgtgatgaatgcttggaaagatgaccaaaatgccgacaacatc accttcattcctgatggtaatggtgaatttaccgatggtatgggcatgctggtggataaa aatgaccttggctttggtaaacgctcatggcgctacagcatgctggttaaagacggtgtg gtagaaaaaatgtttatcgaaccgaatgagccgggcgacccgttcaaagtatcggacgcc gataccatgctcaaatacattgcccctcaatacaaggtgcaagaatcagtgactattttc actaagccaggctgtccttattgcgccaaggcgaaacaagcgctgattgatgccggtcta cagtatgaagagctgattttaggtaaagacgctaccacagtgagtctgcgcgccgtttct ggccgtaccacggtgccgcaagtgtttatcggtggtaaacacattggtggtagcgacgac ttagaagtctacctaaatcaataa |
| A3GRD5 | UniRef cluster | -------------------------------------------KYIAPQYKVQESVTIFTK-----PGCPYCA-KAKQALIDAG---LQYEE----LILG---KDATTVSLRAVS----GRT----------TVPQVFIGGK--------HIGGSDD----LEVYLNQ-------------------------- | atgaggaacacaatgtttacatctaaagaaggtcaaaccattccacaggttacttttcct actcgccaaggtgacgcttgggtcaatgtgactagcgatgaactgttcaaaggcaaaacc gttatcgtgtttagcttgccgggtgcctttactccaacctgttcatccactcacctaccg cgctacaacgagctgtttcctgtctttaaagagcatggtgtcgacagcattctgtgcgta tcggtcaacgatactttcgtgatgaatgcttggaaagatgaccaaaatgccgacaacatc accttcattcctgatggtaatggtgaatttaccgatggtatgggcatgctggtggataaa aatgaccttggctttggtaaacgctcatggcgctacagcatgctggttaaagacggtgtg gtagaaaaaatgtttatcgaaccgaatgagccgggcgacccgttcaaagtatcggacgcc gataccatgctcaaatacattgcccctcaatacaaggtgcaagaatcagtgactattttc actaagccaggctgtccttattgcgccaaggcgaaacaagcgctgattgatgccggtcta cagtatgaagagctgattttaggtaaagacgctaccacagtgagtctgcgcgccgtttct ggccgtaccacggtgccgcaagtgtttatcggtggtaaacacattggtggtagcgacgac ttagaagtctacctaaatcaataa |
| A3EJ81 | UniRef cluster | -------------------------------------------KYIAPQYKVQESVTIFTK-----PGCPYCA-KAKQALIDAG---LQYEE----LILG---KDATTVSLRAVS----GRT----------TVPQVFIGGK--------HIGGSDD----LEVYLNQ-------------------------- | atgaggaacacaatgtttacatctaaagaaggtcaaaccattccacaggttacttttcct actcgccaaggtgacgcttgggtcaatgtgactagcgatgaactgttcaaaggcaaaacc gttatcgtgtttagcttgccgggtgcctttactccaacctgttcatccactcacctaccg cgctacaacgagctgtttcctgtctttaaagagcatggtgtcgacagcattctgtgcgta tcggtcaacgatactttcgtgatgaatgcttggaaagatgaccaaaatgccgacaacatc accttcattcctgatggtaatggtgaatttaccgatggtatgggcatgctggtggataaa aatgaccttggctttggtaaacgctcatggcgctacagcatgctggttaaagacggtgtg gtagaaaaaatgtttatcgaaccgaatgagccgggcgacccgttcaaagtatcggacgcc gataccatgctcaaatacattgcccctcaatacaaggtgcaagaatcagtgactattttc actaagcctggctgtccttattgcgccaaggcgaaacaagcgctgattgatgccggtcta cagtatgaagagctgattttaggtaaagacgccaccacagtgagcctgcgcgctgtttct ggccgtaccacggtgccgcaagtgtttatcggtggtaaacacattggtggcagcgacgac ttagaagtctacctaaatcaataa |
| A3EB97 | UniRef cluster | -------------------------------------------KYIAPQYKVQESVTIFTK-----PGCPYCA-KAKQALIDAG---LQYEE----LILG---KDATTVSLRAVS----GRT----------TVPQVFIGGK--------HIGGSDD----LEVYLNQ-------------------------- | atgaggaacacaatgtttacatctaaagaaggtcaaaccattccacaggttacttttcct actcgccaaggtgacgcttgggtcaatgtgactagcgatgaactgttcaaaggcaaaacc gttatcgtgtttagcttgccgggtgcctttactccaacctgttcatccactcacctaccg cgctacaacgagctgtttcctgtctttaaagagcatggtgtcgacagcattctgtgcgta tcggtcaacgatactttcgtgatgaatgcttggaaagatgaccaaaatgccgacaacatc accttcattcctgatggtaatggtgaatttaccgatggtatgggcatgctggtggataaa aatgaccttggctttggtaaacgctcatggcgctacagcatgctggttaaagacggtgtg gtagaaaaaatgtttatcgaaccgaatgagccgggcgacccgttcaaagtatcggacgcc gataccatgctcaaatacattgcccctcaatacaaggtgcaagaatcagtgactattttc actaagccaggctgtccttattgcgccaaggcgaaacaagcgctgattgatgccggtcta cagtatgaagagctgattttaggtaaagacgctaccacagtgagtctgcgcgccgtttct ggccgtaccacggtgccgcaagtgtttatcggtggtaaacacattggtggtagcgacgac ttagaagtctacctaaatcaataa |
| A2PVZ9 | UniRef cluster | -------------------------------------------KYIAPQYKVQESVTIFTK-----PGCPYCA-KAKQALIDAG---LQYEE----LILG---KDATTVSLRAVS----GRT----------TVPQVFIGGK--------HIGGSDD----LEVYLNQ-------------------------- | atgaggaacacaatgtttacatctaaagaaggtcaaaccattccacaggttacttttcct actcgccaaggtgacgcttgggtcaatgtgactaccgatgaactgttcaaaggcaaaacc gttatcgtgtttagcttgccgggtgcctttactccaacctgttcatccactcacctaccg cgctacaacgagctgtttcctgtctttaaagagcatggtgtcgacagcattctgtgcgta tcggtcaacgatactttcgtgatgaatgcttggaaagatgaccaaaacgccgacaacatc accttcattcctgatggtaatggtgaatttaccgatggtatgggcatgttggtggataaa aatgaccttggctttggtaaacgctcatggcgctacagcatgctggttaaagacggtgtg gtagaaaaaatgtttatcgaaccgaatgagccgggcgacccgttcaaagtatcggacgcc gataccatgctcaaatacattgcccctcaatacaaggtgcaagaatcagtgactattttc actaagccaggctgcccttattgcgccaaggcgaaacaagcgctgattgatgccggtcta cagtatgaagagctgattttaggtaaagacgccaccacagtgagcctgcgcgctgtttct ggccgtaccacggtgccgcaagtgtttatcggtggtaaacacattggtggcagcgacgac ttagaagtctacctaaatcaataa |
| A2PLL7 | UniRef cluster | -------------------------------------------KYIAPQYKVQESVTIFTK-----PGCPYCA-KAKQALIDAG---LQYEE----LILG---KDATTVSLRAVS----GRT----------TVPQVFIGGK--------HIGGSDD----LEVYLNQ-------------------------- | atgaggaacacaatgtttacatctaaagaaggtcaaaccattccacaggttacttttcct actcgccaaggtgacgcttgggtcaatgtgactagcgatgaactgttcaaaggcaaaacc gttatcgtgtttagcttgccgggtgcctttactccaacctgttcatccactcacctaccg cgctacaacgagctgtttcctgtctttaaagagcatggtgtcgacagcattctgtgcgta tcggtcaacgatactttcgtgatgaatgcttggaaagatgaccaaaatgccgacaacatc accttcattcctgatggtaatggtgaatttaccgatggtatgggcatgctggtggataaa aatgaccttggctttggtaaacgctcatggcgctacagcatgctggttaaagacggtgtg gtagaaaaaatgtttatcgaaccgaatgagccgggcgacccgttcaaagtatcggacgcc gataccatgctcaaatacattgcccctcaatacaaggtgcaagaatcagtgactattttc actaagccaggctgtccttattgcgccaaggcgaaacaagcgctgattgatgccggtcta cagtatgaagagctgattttaggtaaagacgctaccacagtgagtctgcgcgccgtttct ggccgtaccacggtgccgcaagtgtttatcggtggtaaacacattggtggtagcgacgac ttagaagtctacctaaatcaataa |
| A2P6Y2 | UniRef cluster | -------------------------------------------KYIAPQYKVQESVTIFTK-----PGCPYCA-KAKQALIDAG---LQYEE----LILG---KDATTVSLRAVS----GRT----------TVPQVFIGGK--------HIGGSDD----LEVYLNQ-------------------------- | atgaggaacacaatgtttacatctaaagaaggtcaaaccattccacaggttacttttcct actcgccaaggtgacgcttgggtcaatgtgactagcgatgagcttttcaaaggcaaaacc gttatcgtgtttagcttgccgggtgcctttactccaacctgttcatccactcacctaccg cgctacaacgagctgtttcctgtctttaaagagcatggtgtcgacagcattctgtgcgta tcggtcaacgatactttcgtgatgaatgcttggaaagatgaccaaaacgccgacaacatc acctttattcctgatggtaatggtgaatttaccgatggtatgggcatgctggtggataaa aatgaccttggctttggtaaacgctcatggcgctacagcatgctggttaaagatggtgtg gtagaaaaaatgtttatcgaaccgaatgagccgggcgacccgttcaaagtatcggacgcc gataccatgctcaaatacattgcccctcaatacaaggtgcaagaatcagtgactattttc actaagcctggatgtccttattgcgccaaggcgaaacaagcgctgattgatgccggtcta cagtatgaagagctgattttaggtaaagacgccaccacagtgagcctgcgcgctgtttct ggccgtaccacggtgccgcaagtgtttatcggtggtaaacacattggtggcagcgacgac ttagaagtctacctaaatcaataa |
| A1F753 | UniRef cluster | -------------------------------------------KYIAPQYKVQESVTIFTK-----PGCPYCA-KAKQALIDAG---LQYEE----LILG---KDATTVSLRAVS----GRT----------TVPQVFIGGK--------HIGGSDD----LEVYLNQ-------------------------- | atgaggaacacaatgtttacatctaaagaaggtcaaaccattccacaggttacttttcct actcgccaaggtgacgcttgggtcaatgtgactagcgatgaactgttcaaaggcaaaacc gttatcgtgtttagcttgccgggtgcctttactccaacctgttcatccactcacctaccg cgctacaacgagctgtttcctgtctttaaagagcatggtgtcgacagcattctgtgcgta tcggtcaacgatactttcgtgatgaatgcttggaaagatgaccaaaatgccgacaacatc accttcattcctgatggtaatggtgaatttaccgatggtatgggcatgctggtggataaa aatgaccttggctttggtaaacgctcatggcgctacagcatgctggttaaagacggtgtg gtagaaaaaatgtttatcgaaccgaatgagccgggcgacccgttcaaagtatcggacgcc gataccatgctcaaatacattgcccctcaatacaaggtgcaagaatcagtgactattttc actaagccaggctgtccttattgcgccaaggcgaaacaagcgctgattgatgccggtcta cagtatgaagagctgattttaggtaaagacgctaccacagtgagtctgcgcgccgtttct ggccgtaccacggtgccgcaagtgtttatcggtggtaaacacattggtggtagcgacgac ttagaagtctacctaaatcaataa |
| A1EMB6 | UniRef cluster | -------------------------------------------KYIAPQYKVQESVTIFTK-----PGCPYCA-KAKQALIDAG---LQYEE----LILG---KDATTVSLRAVS----GRT----------TVPQVFIGGK--------HIGGSDD----LEVYLNQ-------------------------- | atgaggaacacaatgtttacatctaaagaaggtcaaaccattccacaggttacttttcct actcgccaaggtgacgcttgggtcaatgtgactagcgatgaactgttcaaaggcaaaacc gttatcgtgtttagcttgccgggtgcctttactccaacctgttcatccactcacctaccg cgctacaacgagctgtttcctgtctttaaagagcatggtgtcgacagcattctgtgcgta tcggtcaacgatactttcgtgatgaatgcttggaaagatgaccaaaatgccgacaacatc accttcattcctgatggtaatggtgaatttaccgatggtatgggcatgctggtggataaa aatgaccttggctttggtaaacgctcatggcgctacagcatgctggttaaagatggtgtg gtagaaaaaatgtttatcgaaccgaatgagccgggcgacccgttcaaagtatcggacgcc gataccatgctcaaatacattgcccctcaatacaaggtgcaagaatcagtgactattttc actaagcctggatgtccttattgcgccaaggcgaaacaagcgctgattgatgccggtcta cagtatgaagagctgattttaggtaaagacgccaccacagtgagcctgcgcgctgtttct ggccgtaccacggtgccgcaagtgtttatcggtggtaaacacattggtggcagcgacgac ttagaagtctacctaaatcaataa |
| Q9KNU3 | UniRef cluster | -------------------------------------------KYIAPQYKVQESVTIFTK-----PGCPYCA-KAKQALIDAG---LQYEE----LILG---KDATTVSLRAVS----GRT----------TVPQVFIGGK--------HIGGSDD----LEVYLNQ-------------------------- | atgaggaacacaatgtttacatctaaagaaggtcaaaccattccacaggttacttttcct actcgccaaggtgacgcttgggtcaatgtgactagcgatgaactgttcaaaggcaaaacc gttatcgtgtttagcttgccgggtgcctttactccaacctgttcatccactcacctaccg cgctacaacgagctgtttcctgtctttaaagagcatggtgtcgacagcattctgtgcgta tcggtcaacgatactttcgtgatgaatgcttggaaagatgaccaaaatgccgacaacatc accttcattcctgatggtaatggtgaatttaccgatggtatgggcatgctggtggataaa aatgaccttggctttggtaaacgctcatggcgctacagcatgctggttaaagacggtgtg gtagaaaaaatgtttatcgaaccgaatgagccgggcgacccgttcaaagtatcggacgcc gataccatgctcaaatacattgcccctcaatacaaggtgcaagaatcagtgactattttc actaagccaggctgtccttattgcgccaaggcgaaacaagcgctgattgatgccggtcta cagtatgaagagctgattttaggtaaagacgctaccacagtgagtctgcgcgccgtttct ggccgtaccacggtgccgcaagtgtttatcggtggtaaacacattggtggtagcgacgac ttagaagtctacctaaatcaataa |
| A6Y1K9 | UniRef cluster | -------------------------------------------KYIAPQYKVQESVTIFTK-----PGCPYCA-KAKQALIDAG---LQYEE----LILG---KDATTVSLRAVS----GRT----------TVPQVFIGGK--------HIGGSDD----LEVYLNQ-------------------------- | atgaggaacacaatgtttacatctaaagaaggtcaaaccattccacaggttacttttcct actcgccaaggtgacgcttgggtcaatgtgactagcgatgagctgttcaaaggcaaaacc gttatcgtgtttagcttgccgggtgcctttactccaacctgttcatccactcacctaccg cgctacaacgagctgtttcctgtctttaaagagcatggtgtcgacagcattctgtgcgta tcggtcaacgatactttcgtgatgaatgcttggaaagatgaccaaaatgccgacaacatc acctttattcctgatggtaatggtgaatttaccgatggtatggggatgctggtggataaa aatgaccttggctttggtaaacgctcatggcgctacagcatgctggttaaagacggtgtg gtagaaaaaatgtttatcgaaccgaatgagccgggcgacccgttcaaagtatcggacgcc gataccatgctcaaatacattgcccctcaatacaaggtgcaagaatcagtgactattttc actaagcctggatgtccttattgcgccaaggcgaaacaagcgctgattgatgccggtcta cagtatgaagagctgattttaggtaaagacgccaccacagtgagcctgcgcgctgtttct ggccgtaccacggtgccgcaagtgtttatcggtggtaaacacattggcggcagcgacgac ttagaagtctacctaaatcaataa |
| A6XU96 | UniRef cluster | -------------------------------------------KYIAPQYKVQESVTIFTK-----PGCPYCA-KAKQALIDAG---LQYEE----LILG---KDATTVSLRAVS----GRT----------TVPQVFIGGK--------HIGGSDD----LEVYLNQ-------------------------- | atgaggaacacaatgtttacatctaaagaaggtcaaaccattccacaggttacttttcct actcgccaaggtgacgcttgggtcaatgtgactagcgatgaactgttcaaaggcaaaacc gttatcgtgtttagcttgccgggtgcctttactccaacctgttcatccactcacctaccg cgctacaacgagctgtttcctgtctttaaagagcatggtgtcgacagcattctgtgcgta tcggtcaacgatactttcgtgatgaatgcttggaaagatgaccaaaatgccgacaacatc accttcattcctgatggtaatggtgaatttaccgatggtatgggcatgttggtggataaa aatgaccttggctttggtaaacgctcatggcgctacagcatgctggttaaagatggtgtg gtagaaaaaatgtttatcgaaccgaatgagccgggcgacccgttcaaagtatcggacgcc gataccatgctcaaatacattgcccctcaatacaaggtgcaagaatcagtgactattttc actaagcctggatgtccttattgcgccaaggcgaaacaagcgctgattgatgccggtcta cagtatgaagagctgattttaggtaaagacgccaccacagtgagcctgcgcgctgtttct ggccgtaccacggtgccgcaagtgtttatcggtggtaaacacattggtggcagcgacgac ttagaagtctacctaaatcaataa |
| A6AFP4 | UniRef cluster | -------------------------------------------KYIAPQYKVQESVTIFTK-----PGCPYCA-KAKQALIDAG---LQYEE----LILG---KDATTVSLRAVS----GRT----------TVPQVFIGGK--------HIGGSDD----LEVYLNQ-------------------------- | atgaggaacacaatgtttacatctaaagaaggtcaaaccattccacaggttacttttcct actcgccaaggtgacgcttgggtcaatgtgactagcgatgaactgttcaaaggcaaaacc gttatcgtgtttagcttgccgggtgcctttactccaacctgttcatccactcacctaccg cgctacaacgagctgtatcctgtctttaaagagaatggtgtcgacagcattctgtgcgta tcggtcaacgatactttcgtgatgaatgcttggaaagatgaccaaaatgccgacaacatc accttcattcctgatggtaatggtgaatttaccgatggtatgggcatgctggtggataaa aatgaccttggctttggtaaacgctcatggcgctacagcatgctggttaaagacggtgtg gtagaaaaaatgtttatcgaaccgaatgagccgggcgacccgttcaaagtatcggacgcc gataccatgctcaaatacattgcccctcaatacaaggtgcaagaatcagtgactattttc actaagccaggctgtccttattgcgccaaggcgaaacaagcgctgattgatgccggtcta cagtatgaagagctgattttaggtaaagacgccaccacagtgagcctgcgcgctgtttct ggccgtaccacggtgccgcaagtgtttatcggtggtaaacacattggtggcagcgacgac ttagaagtctacctaaatcaataa |
| A6A149 | UniRef cluster | -------------------------------------------KYIAPQYKVQESVTIFTK-----PGCPYCA-KAKQALIDAG---LQYEE----LILG---KDATTVSLRAVS----GRT----------TVPQVFIGGK--------HIGGSDD----LEVYLNQ-------------------------- | atgaggaacacaatgtttacatctaaagaaggtcaaaccattccacaggttacttttcct actcgccaaggtgacgcttgggtcaatgtgactagcgatgagctgttcaaaggcaaaacc gttatcgtgtttagcttgccgggtgcctttactccaacctgttcatccactcacctaccg cgctacaacgagctgtttcctgtctttaaagagcatggtgtcgacagcattctgtgcgta tcggtcaacgatactttcgtgatgaatgcttggaaagatgaccaaaacgccgacaacatc accttcattcctgatggtaatggtgaatttaccgatggtatgggcatgttggtggataaa aatgaccttggctttggtaaacgctcatggcgctacagcatgctggttaaagacggtgtg gtagaaaaaatgtttatcgaaccgaatgagccgggcgacccgttcaaagtatcggacgcc gataccatgctcaaatacattgcccctcaatacaaggtgcaagaatcagtgactattttc actaagcctggctgtccttattgcgccaaggcgaaacaagcgctgattgatgccggtcta cagtatgaagagctgattttaggtaaagacgccaccacagtgagcctgcgcgctgtttct ggccgtaccacggtgccgcaagtgtttatcggtggtaaacacattggtggcagcgacgac ttagaagtctacctaaatcaataa |
| A5F510 | UniRef cluster | -------------------------------------------KYIAPQYKVQESVTIFTK-----PGCPYCA-KAKQALIDAG---LQYEE----LILG---KDATTVSLRAVS----GRT----------TVPQVFIGGK--------HIGGSDD----LEVYLNQ-------------------------- | atgaggaacacaatgtttacatctaaagaaggtcaaaccattccacaggttacttttcct actcgccaaggtgacgcttgggtcaatgtgactagcgatgaactgttcaaaggcaaaacc gttatcgtgtttagcttgccgggtgcctttactccaacctgttcatccactcacctaccg cgctacaacgagctgtttcctgtctttaaagagcatggtgtcgacagcattctgtgcgta tcggtcaacgatactttcgtgatgaatgcttggaaagatgaccaaaatgccgacaacatc accttcattcctgatggtaatggtgaatttaccgatggtatgggcatgctggtggataaa aatgaccttggctttggtaaacgctcatggcgctacagcatgctggttaaagacggtgtg gtagaaaaaatgtttatcgaaccgaatgagccgggcgacccgttcaaagtatcggacgcc gataccatgctcaaatacattgcccctcaatacaaggtgcaagaatcagtgactattttc actaagccaggctgtccttattgcgccaaggcgaaacaagcgctgattgatgccggtcta cagtatgaagagctgattttaggtaaagacgctaccacagtgagtctgcgcgccgtttct ggccgtaccacggtgccgcaagtgtttatcggtggtaaacacattggtggtagcgacgac ttagaagtctacctaaatcaataa |
| A3GXC3 | UniRef cluster | -------------------------------------------KYIAPQYKVQESVTIFTK-----PGCPYCA-KAKQALIDAG---LQYEE----LILG---KDATTVSLRAVS----GRT----------TVPQVFIGGK--------HIGGSDD----LEVYLNQ-------------------------- | atgaggaacacaatgtttacatctaaagaaggtcaaaccattccacaggttacttttcct actcgccaaggtgacgcttgggtcaatgtgactagcgatgaactgttcaaaggcaaaacc gttatcgtgtttagcttgccgggtgcctttactccaacctgttcatccactcacctaccg cgctacaacgagctgtttcctgtctttaaagagcatggtgtcgacagcattctgtgcgta tcggtcaacgatactttcgtgatgaatgcttggaaagatgaccaaaatgccgacaacatc accttcattcctgatggtaatggtgaatttaccgatggtatgggcatgctggtggataaa aatgaccttggctttggtaaacgctcatggcgctacagcatgctggttaaagacggtgtg gtagaaaaaatgtttatcgaaccgaatgagccgggcgacccgttcaaagtatcggacgcc gataccatgctcaaatacattgcccctcaatacaaggtgcaagaatcagtgactattttc actaagccaggctgtccttattgcgccaaggcgaaacaagcgctgattgatgccggtcta cagtatgaagagctgattttaggtaaagacgctaccacagtgagtctgcgcgccgtttct ggccgtaccacggtgccgcaagtgtttatcggtggtaaacacattggtggtagcgacgac ttagaagtctacctaaatcaataa |
| A3GRD5 | UniRef cluster | -------------------------------------------KYIAPQYKVQESVTIFTK-----PGCPYCA-KAKQALIDAG---LQYEE----LILG---KDATTVSLRAVS----GRT----------TVPQVFIGGK--------HIGGSDD----LEVYLNQ-------------------------- | atgaggaacacaatgtttacatctaaagaaggtcaaaccattccacaggttacttttcct actcgccaaggtgacgcttgggtcaatgtgactagcgatgaactgttcaaaggcaaaacc gttatcgtgtttagcttgccgggtgcctttactccaacctgttcatccactcacctaccg cgctacaacgagctgtttcctgtctttaaagagcatggtgtcgacagcattctgtgcgta tcggtcaacgatactttcgtgatgaatgcttggaaagatgaccaaaatgccgacaacatc accttcattcctgatggtaatggtgaatttaccgatggtatgggcatgctggtggataaa aatgaccttggctttggtaaacgctcatggcgctacagcatgctggttaaagacggtgtg gtagaaaaaatgtttatcgaaccgaatgagccgggcgacccgttcaaagtatcggacgcc gataccatgctcaaatacattgcccctcaatacaaggtgcaagaatcagtgactattttc actaagccaggctgtccttattgcgccaaggcgaaacaagcgctgattgatgccggtcta cagtatgaagagctgattttaggtaaagacgctaccacagtgagtctgcgcgccgtttct ggccgtaccacggtgccgcaagtgtttatcggtggtaaacacattggtggtagcgacgac ttagaagtctacctaaatcaataa |
| A3EJ81 | UniRef cluster | -------------------------------------------KYIAPQYKVQESVTIFTK-----PGCPYCA-KAKQALIDAG---LQYEE----LILG---KDATTVSLRAVS----GRT----------TVPQVFIGGK--------HIGGSDD----LEVYLNQ-------------------------- | atgaggaacacaatgtttacatctaaagaaggtcaaaccattccacaggttacttttcct actcgccaaggtgacgcttgggtcaatgtgactagcgatgaactgttcaaaggcaaaacc gttatcgtgtttagcttgccgggtgcctttactccaacctgttcatccactcacctaccg cgctacaacgagctgtttcctgtctttaaagagcatggtgtcgacagcattctgtgcgta tcggtcaacgatactttcgtgatgaatgcttggaaagatgaccaaaatgccgacaacatc accttcattcctgatggtaatggtgaatttaccgatggtatgggcatgctggtggataaa aatgaccttggctttggtaaacgctcatggcgctacagcatgctggttaaagacggtgtg gtagaaaaaatgtttatcgaaccgaatgagccgggcgacccgttcaaagtatcggacgcc gataccatgctcaaatacattgcccctcaatacaaggtgcaagaatcagtgactattttc actaagcctggctgtccttattgcgccaaggcgaaacaagcgctgattgatgccggtcta cagtatgaagagctgattttaggtaaagacgccaccacagtgagcctgcgcgctgtttct ggccgtaccacggtgccgcaagtgtttatcggtggtaaacacattggtggcagcgacgac ttagaagtctacctaaatcaataa |
| A3EB97 | UniRef cluster | -------------------------------------------KYIAPQYKVQESVTIFTK-----PGCPYCA-KAKQALIDAG---LQYEE----LILG---KDATTVSLRAVS----GRT----------TVPQVFIGGK--------HIGGSDD----LEVYLNQ-------------------------- | atgaggaacacaatgtttacatctaaagaaggtcaaaccattccacaggttacttttcct actcgccaaggtgacgcttgggtcaatgtgactagcgatgaactgttcaaaggcaaaacc gttatcgtgtttagcttgccgggtgcctttactccaacctgttcatccactcacctaccg cgctacaacgagctgtttcctgtctttaaagagcatggtgtcgacagcattctgtgcgta tcggtcaacgatactttcgtgatgaatgcttggaaagatgaccaaaatgccgacaacatc accttcattcctgatggtaatggtgaatttaccgatggtatgggcatgctggtggataaa aatgaccttggctttggtaaacgctcatggcgctacagcatgctggttaaagacggtgtg gtagaaaaaatgtttatcgaaccgaatgagccgggcgacccgttcaaagtatcggacgcc gataccatgctcaaatacattgcccctcaatacaaggtgcaagaatcagtgactattttc actaagccaggctgtccttattgcgccaaggcgaaacaagcgctgattgatgccggtcta cagtatgaagagctgattttaggtaaagacgctaccacagtgagtctgcgcgccgtttct ggccgtaccacggtgccgcaagtgtttatcggtggtaaacacattggtggtagcgacgac ttagaagtctacctaaatcaataa |
| A2PVZ9 | UniRef cluster | -------------------------------------------KYIAPQYKVQESVTIFTK-----PGCPYCA-KAKQALIDAG---LQYEE----LILG---KDATTVSLRAVS----GRT----------TVPQVFIGGK--------HIGGSDD----LEVYLNQ-------------------------- | atgaggaacacaatgtttacatctaaagaaggtcaaaccattccacaggttacttttcct actcgccaaggtgacgcttgggtcaatgtgactaccgatgaactgttcaaaggcaaaacc gttatcgtgtttagcttgccgggtgcctttactccaacctgttcatccactcacctaccg cgctacaacgagctgtttcctgtctttaaagagcatggtgtcgacagcattctgtgcgta tcggtcaacgatactttcgtgatgaatgcttggaaagatgaccaaaacgccgacaacatc accttcattcctgatggtaatggtgaatttaccgatggtatgggcatgttggtggataaa aatgaccttggctttggtaaacgctcatggcgctacagcatgctggttaaagacggtgtg gtagaaaaaatgtttatcgaaccgaatgagccgggcgacccgttcaaagtatcggacgcc gataccatgctcaaatacattgcccctcaatacaaggtgcaagaatcagtgactattttc actaagccaggctgcccttattgcgccaaggcgaaacaagcgctgattgatgccggtcta cagtatgaagagctgattttaggtaaagacgccaccacagtgagcctgcgcgctgtttct ggccgtaccacggtgccgcaagtgtttatcggtggtaaacacattggtggcagcgacgac ttagaagtctacctaaatcaataa |
| A2PLL7 | UniRef cluster | -------------------------------------------KYIAPQYKVQESVTIFTK-----PGCPYCA-KAKQALIDAG---LQYEE----LILG---KDATTVSLRAVS----GRT----------TVPQVFIGGK--------HIGGSDD----LEVYLNQ-------------------------- | atgaggaacacaatgtttacatctaaagaaggtcaaaccattccacaggttacttttcct actcgccaaggtgacgcttgggtcaatgtgactagcgatgaactgttcaaaggcaaaacc gttatcgtgtttagcttgccgggtgcctttactccaacctgttcatccactcacctaccg cgctacaacgagctgtttcctgtctttaaagagcatggtgtcgacagcattctgtgcgta tcggtcaacgatactttcgtgatgaatgcttggaaagatgaccaaaatgccgacaacatc accttcattcctgatggtaatggtgaatttaccgatggtatgggcatgctggtggataaa aatgaccttggctttggtaaacgctcatggcgctacagcatgctggttaaagacggtgtg gtagaaaaaatgtttatcgaaccgaatgagccgggcgacccgttcaaagtatcggacgcc gataccatgctcaaatacattgcccctcaatacaaggtgcaagaatcagtgactattttc actaagccaggctgtccttattgcgccaaggcgaaacaagcgctgattgatgccggtcta cagtatgaagagctgattttaggtaaagacgctaccacagtgagtctgcgcgccgtttct ggccgtaccacggtgccgcaagtgtttatcggtggtaaacacattggtggtagcgacgac ttagaagtctacctaaatcaataa |
| A2P6Y2 | UniRef cluster | -------------------------------------------KYIAPQYKVQESVTIFTK-----PGCPYCA-KAKQALIDAG---LQYEE----LILG---KDATTVSLRAVS----GRT----------TVPQVFIGGK--------HIGGSDD----LEVYLNQ-------------------------- | atgaggaacacaatgtttacatctaaagaaggtcaaaccattccacaggttacttttcct actcgccaaggtgacgcttgggtcaatgtgactagcgatgagcttttcaaaggcaaaacc gttatcgtgtttagcttgccgggtgcctttactccaacctgttcatccactcacctaccg cgctacaacgagctgtttcctgtctttaaagagcatggtgtcgacagcattctgtgcgta tcggtcaacgatactttcgtgatgaatgcttggaaagatgaccaaaacgccgacaacatc acctttattcctgatggtaatggtgaatttaccgatggtatgggcatgctggtggataaa aatgaccttggctttggtaaacgctcatggcgctacagcatgctggttaaagatggtgtg gtagaaaaaatgtttatcgaaccgaatgagccgggcgacccgttcaaagtatcggacgcc gataccatgctcaaatacattgcccctcaatacaaggtgcaagaatcagtgactattttc actaagcctggatgtccttattgcgccaaggcgaaacaagcgctgattgatgccggtcta cagtatgaagagctgattttaggtaaagacgccaccacagtgagcctgcgcgctgtttct ggccgtaccacggtgccgcaagtgtttatcggtggtaaacacattggtggcagcgacgac ttagaagtctacctaaatcaataa |
| A1F753 | UniRef cluster | -------------------------------------------KYIAPQYKVQESVTIFTK-----PGCPYCA-KAKQALIDAG---LQYEE----LILG---KDATTVSLRAVS----GRT----------TVPQVFIGGK--------HIGGSDD----LEVYLNQ-------------------------- | atgaggaacacaatgtttacatctaaagaaggtcaaaccattccacaggttacttttcct actcgccaaggtgacgcttgggtcaatgtgactagcgatgaactgttcaaaggcaaaacc gttatcgtgtttagcttgccgggtgcctttactccaacctgttcatccactcacctaccg cgctacaacgagctgtttcctgtctttaaagagcatggtgtcgacagcattctgtgcgta tcggtcaacgatactttcgtgatgaatgcttggaaagatgaccaaaatgccgacaacatc accttcattcctgatggtaatggtgaatttaccgatggtatgggcatgctggtggataaa aatgaccttggctttggtaaacgctcatggcgctacagcatgctggttaaagacggtgtg gtagaaaaaatgtttatcgaaccgaatgagccgggcgacccgttcaaagtatcggacgcc gataccatgctcaaatacattgcccctcaatacaaggtgcaagaatcagtgactattttc actaagccaggctgtccttattgcgccaaggcgaaacaagcgctgattgatgccggtcta cagtatgaagagctgattttaggtaaagacgctaccacagtgagtctgcgcgccgtttct ggccgtaccacggtgccgcaagtgtttatcggtggtaaacacattggtggtagcgacgac ttagaagtctacctaaatcaataa |
| A1EMB6 | UniRef cluster | -------------------------------------------KYIAPQYKVQESVTIFTK-----PGCPYCA-KAKQALIDAG---LQYEE----LILG---KDATTVSLRAVS----GRT----------TVPQVFIGGK--------HIGGSDD----LEVYLNQ-------------------------- | atgaggaacacaatgtttacatctaaagaaggtcaaaccattccacaggttacttttcct actcgccaaggtgacgcttgggtcaatgtgactagcgatgaactgttcaaaggcaaaacc gttatcgtgtttagcttgccgggtgcctttactccaacctgttcatccactcacctaccg cgctacaacgagctgtttcctgtctttaaagagcatggtgtcgacagcattctgtgcgta tcggtcaacgatactttcgtgatgaatgcttggaaagatgaccaaaatgccgacaacatc accttcattcctgatggtaatggtgaatttaccgatggtatgggcatgctggtggataaa aatgaccttggctttggtaaacgctcatggcgctacagcatgctggttaaagatggtgtg gtagaaaaaatgtttatcgaaccgaatgagccgggcgacccgttcaaagtatcggacgcc gataccatgctcaaatacattgcccctcaatacaaggtgcaagaatcagtgactattttc actaagcctggatgtccttattgcgccaaggcgaaacaagcgctgattgatgccggtcta cagtatgaagagctgattttaggtaaagacgccaccacagtgagcctgcgcgctgtttct ggccgtaccacggtgccgcaagtgtttatcggtggtaaacacattggtggcagcgacgac ttagaagtctacctaaatcaataa |
| Q9KNU3 | UniRef cluster | -------------------------------------------KYIAPQYKVQESVTIFTK-----PGCPYCA-KAKQALIDAG---LQYEE----LILG---KDATTVSLRAVS----GRT----------TVPQVFIGGK--------HIGGSDD----LEVYLNQ-------------------------- | atgaggaacacaatgtttacatctaaagaaggtcaaaccattccacaggttacttttcct actcgccaaggtgacgcttgggtcaatgtgactagcgatgaactgttcaaaggcaaaacc gttatcgtgtttagcttgccgggtgcctttactccaacctgttcatccactcacctaccg cgctacaacgagctgtttcctgtctttaaagagcatggtgtcgacagcattctgtgcgta tcggtcaacgatactttcgtgatgaatgcttggaaagatgaccaaaatgccgacaacatc accttcattcctgatggtaatggtgaatttaccgatggtatgggcatgctggtggataaa aatgaccttggctttggtaaacgctcatggcgctacagcatgctggttaaagacggtgtg gtagaaaaaatgtttatcgaaccgaatgagccgggcgacccgttcaaagtatcggacgcc gataccatgctcaaatacattgcccctcaatacaaggtgcaagaatcagtgactattttc actaagccaggctgtccttattgcgccaaggcgaaacaagcgctgattgatgccggtcta cagtatgaagagctgattttaggtaaagacgctaccacagtgagtctgcgcgccgtttct ggccgtaccacggtgccgcaagtgtttatcggtggtaaacacattggtggtagcgacgac ttagaagtctacctaaatcaataa |
| A6Y1K9 | UniRef cluster | -------------------------------------------KYIAPQYKVQESVTIFTK-----PGCPYCA-KAKQALIDAG---LQYEE----LILG---KDATTVSLRAVS----GRT----------TVPQVFIGGK--------HIGGSDD----LEVYLNQ-------------------------- | atgaggaacacaatgtttacatctaaagaaggtcaaaccattccacaggttacttttcct actcgccaaggtgacgcttgggtcaatgtgactagcgatgagctgttcaaaggcaaaacc gttatcgtgtttagcttgccgggtgcctttactccaacctgttcatccactcacctaccg cgctacaacgagctgtttcctgtctttaaagagcatggtgtcgacagcattctgtgcgta tcggtcaacgatactttcgtgatgaatgcttggaaagatgaccaaaatgccgacaacatc acctttattcctgatggtaatggtgaatttaccgatggtatggggatgctggtggataaa aatgaccttggctttggtaaacgctcatggcgctacagcatgctggttaaagacggtgtg gtagaaaaaatgtttatcgaaccgaatgagccgggcgacccgttcaaagtatcggacgcc gataccatgctcaaatacattgcccctcaatacaaggtgcaagaatcagtgactattttc actaagcctggatgtccttattgcgccaaggcgaaacaagcgctgattgatgccggtcta cagtatgaagagctgattttaggtaaagacgccaccacagtgagcctgcgcgctgtttct ggccgtaccacggtgccgcaagtgtttatcggtggtaaacacattggcggcagcgacgac ttagaagtctacctaaatcaataa |
| A6XU96 | UniRef cluster | -------------------------------------------KYIAPQYKVQESVTIFTK-----PGCPYCA-KAKQALIDAG---LQYEE----LILG---KDATTVSLRAVS----GRT----------TVPQVFIGGK--------HIGGSDD----LEVYLNQ-------------------------- | atgaggaacacaatgtttacatctaaagaaggtcaaaccattccacaggttacttttcct actcgccaaggtgacgcttgggtcaatgtgactagcgatgaactgttcaaaggcaaaacc gttatcgtgtttagcttgccgggtgcctttactccaacctgttcatccactcacctaccg cgctacaacgagctgtttcctgtctttaaagagcatggtgtcgacagcattctgtgcgta tcggtcaacgatactttcgtgatgaatgcttggaaagatgaccaaaatgccgacaacatc accttcattcctgatggtaatggtgaatttaccgatggtatgggcatgttggtggataaa aatgaccttggctttggtaaacgctcatggcgctacagcatgctggttaaagatggtgtg gtagaaaaaatgtttatcgaaccgaatgagccgggcgacccgttcaaagtatcggacgcc gataccatgctcaaatacattgcccctcaatacaaggtgcaagaatcagtgactattttc actaagcctggatgtccttattgcgccaaggcgaaacaagcgctgattgatgccggtcta cagtatgaagagctgattttaggtaaagacgccaccacagtgagcctgcgcgctgtttct ggccgtaccacggtgccgcaagtgtttatcggtggtaaacacattggtggcagcgacgac ttagaagtctacctaaatcaataa |
| A6AFP4 | UniRef cluster | -------------------------------------------KYIAPQYKVQESVTIFTK-----PGCPYCA-KAKQALIDAG---LQYEE----LILG---KDATTVSLRAVS----GRT----------TVPQVFIGGK--------HIGGSDD----LEVYLNQ-------------------------- | atgaggaacacaatgtttacatctaaagaaggtcaaaccattccacaggttacttttcct actcgccaaggtgacgcttgggtcaatgtgactagcgatgaactgttcaaaggcaaaacc gttatcgtgtttagcttgccgggtgcctttactccaacctgttcatccactcacctaccg cgctacaacgagctgtatcctgtctttaaagagaatggtgtcgacagcattctgtgcgta tcggtcaacgatactttcgtgatgaatgcttggaaagatgaccaaaatgccgacaacatc accttcattcctgatggtaatggtgaatttaccgatggtatgggcatgctggtggataaa aatgaccttggctttggtaaacgctcatggcgctacagcatgctggttaaagacggtgtg gtagaaaaaatgtttatcgaaccgaatgagccgggcgacccgttcaaagtatcggacgcc gataccatgctcaaatacattgcccctcaatacaaggtgcaagaatcagtgactattttc actaagccaggctgtccttattgcgccaaggcgaaacaagcgctgattgatgccggtcta cagtatgaagagctgattttaggtaaagacgccaccacagtgagcctgcgcgctgtttct ggccgtaccacggtgccgcaagtgtttatcggtggtaaacacattggtggcagcgacgac ttagaagtctacctaaatcaataa |
| A6A149 | UniRef cluster | -------------------------------------------KYIAPQYKVQESVTIFTK-----PGCPYCA-KAKQALIDAG---LQYEE----LILG---KDATTVSLRAVS----GRT----------TVPQVFIGGK--------HIGGSDD----LEVYLNQ-------------------------- | atgaggaacacaatgtttacatctaaagaaggtcaaaccattccacaggttacttttcct actcgccaaggtgacgcttgggtcaatgtgactagcgatgagctgttcaaaggcaaaacc gttatcgtgtttagcttgccgggtgcctttactccaacctgttcatccactcacctaccg cgctacaacgagctgtttcctgtctttaaagagcatggtgtcgacagcattctgtgcgta tcggtcaacgatactttcgtgatgaatgcttggaaagatgaccaaaacgccgacaacatc accttcattcctgatggtaatggtgaatttaccgatggtatgggcatgttggtggataaa aatgaccttggctttggtaaacgctcatggcgctacagcatgctggttaaagacggtgtg gtagaaaaaatgtttatcgaaccgaatgagccgggcgacccgttcaaagtatcggacgcc gataccatgctcaaatacattgcccctcaatacaaggtgcaagaatcagtgactattttc actaagcctggctgtccttattgcgccaaggcgaaacaagcgctgattgatgccggtcta cagtatgaagagctgattttaggtaaagacgccaccacagtgagcctgcgcgctgtttct ggccgtaccacggtgccgcaagtgtttatcggtggtaaacacattggtggcagcgacgac ttagaagtctacctaaatcaataa |
| A5F510 | UniRef cluster | -------------------------------------------KYIAPQYKVQESVTIFTK-----PGCPYCA-KAKQALIDAG---LQYEE----LILG---KDATTVSLRAVS----GRT----------TVPQVFIGGK--------HIGGSDD----LEVYLNQ-------------------------- | atgaggaacacaatgtttacatctaaagaaggtcaaaccattccacaggttacttttcct actcgccaaggtgacgcttgggtcaatgtgactagcgatgaactgttcaaaggcaaaacc gttatcgtgtttagcttgccgggtgcctttactccaacctgttcatccactcacctaccg cgctacaacgagctgtttcctgtctttaaagagcatggtgtcgacagcattctgtgcgta tcggtcaacgatactttcgtgatgaatgcttggaaagatgaccaaaatgccgacaacatc accttcattcctgatggtaatggtgaatttaccgatggtatgggcatgctggtggataaa aatgaccttggctttggtaaacgctcatggcgctacagcatgctggttaaagacggtgtg gtagaaaaaatgtttatcgaaccgaatgagccgggcgacccgttcaaagtatcggacgcc gataccatgctcaaatacattgcccctcaatacaaggtgcaagaatcagtgactattttc actaagccaggctgtccttattgcgccaaggcgaaacaagcgctgattgatgccggtcta cagtatgaagagctgattttaggtaaagacgctaccacagtgagtctgcgcgccgtttct ggccgtaccacggtgccgcaagtgtttatcggtggtaaacacattggtggtagcgacgac ttagaagtctacctaaatcaataa |
| A3GXC3 | UniRef cluster | -------------------------------------------KYIAPQYKVQESVTIFTK-----PGCPYCA-KAKQALIDAG---LQYEE----LILG---KDATTVSLRAVS----GRT----------TVPQVFIGGK--------HIGGSDD----LEVYLNQ-------------------------- | atgaggaacacaatgtttacatctaaagaaggtcaaaccattccacaggttacttttcct actcgccaaggtgacgcttgggtcaatgtgactagcgatgaactgttcaaaggcaaaacc gttatcgtgtttagcttgccgggtgcctttactccaacctgttcatccactcacctaccg cgctacaacgagctgtttcctgtctttaaagagcatggtgtcgacagcattctgtgcgta tcggtcaacgatactttcgtgatgaatgcttggaaagatgaccaaaatgccgacaacatc accttcattcctgatggtaatggtgaatttaccgatggtatgggcatgctggtggataaa aatgaccttggctttggtaaacgctcatggcgctacagcatgctggttaaagacggtgtg gtagaaaaaatgtttatcgaaccgaatgagccgggcgacccgttcaaagtatcggacgcc gataccatgctcaaatacattgcccctcaatacaaggtgcaagaatcagtgactattttc actaagccaggctgtccttattgcgccaaggcgaaacaagcgctgattgatgccggtcta cagtatgaagagctgattttaggtaaagacgctaccacagtgagtctgcgcgccgtttct ggccgtaccacggtgccgcaagtgtttatcggtggtaaacacattggtggtagcgacgac ttagaagtctacctaaatcaataa |
| A3GRD5 | UniRef cluster | -------------------------------------------KYIAPQYKVQESVTIFTK-----PGCPYCA-KAKQALIDAG---LQYEE----LILG---KDATTVSLRAVS----GRT----------TVPQVFIGGK--------HIGGSDD----LEVYLNQ-------------------------- | atgaggaacacaatgtttacatctaaagaaggtcaaaccattccacaggttacttttcct actcgccaaggtgacgcttgggtcaatgtgactagcgatgaactgttcaaaggcaaaacc gttatcgtgtttagcttgccgggtgcctttactccaacctgttcatccactcacctaccg cgctacaacgagctgtttcctgtctttaaagagcatggtgtcgacagcattctgtgcgta tcggtcaacgatactttcgtgatgaatgcttggaaagatgaccaaaatgccgacaacatc accttcattcctgatggtaatggtgaatttaccgatggtatgggcatgctggtggataaa aatgaccttggctttggtaaacgctcatggcgctacagcatgctggttaaagacggtgtg gtagaaaaaatgtttatcgaaccgaatgagccgggcgacccgttcaaagtatcggacgcc gataccatgctcaaatacattgcccctcaatacaaggtgcaagaatcagtgactattttc actaagccaggctgtccttattgcgccaaggcgaaacaagcgctgattgatgccggtcta cagtatgaagagctgattttaggtaaagacgctaccacagtgagtctgcgcgccgtttct ggccgtaccacggtgccgcaagtgtttatcggtggtaaacacattggtggtagcgacgac ttagaagtctacctaaatcaataa |
| A3EJ81 | UniRef cluster | -------------------------------------------KYIAPQYKVQESVTIFTK-----PGCPYCA-KAKQALIDAG---LQYEE----LILG---KDATTVSLRAVS----GRT----------TVPQVFIGGK--------HIGGSDD----LEVYLNQ-------------------------- | atgaggaacacaatgtttacatctaaagaaggtcaaaccattccacaggttacttttcct actcgccaaggtgacgcttgggtcaatgtgactagcgatgaactgttcaaaggcaaaacc gttatcgtgtttagcttgccgggtgcctttactccaacctgttcatccactcacctaccg cgctacaacgagctgtttcctgtctttaaagagcatggtgtcgacagcattctgtgcgta tcggtcaacgatactttcgtgatgaatgcttggaaagatgaccaaaatgccgacaacatc accttcattcctgatggtaatggtgaatttaccgatggtatgggcatgctggtggataaa aatgaccttggctttggtaaacgctcatggcgctacagcatgctggttaaagacggtgtg gtagaaaaaatgtttatcgaaccgaatgagccgggcgacccgttcaaagtatcggacgcc gataccatgctcaaatacattgcccctcaatacaaggtgcaagaatcagtgactattttc actaagcctggctgtccttattgcgccaaggcgaaacaagcgctgattgatgccggtcta cagtatgaagagctgattttaggtaaagacgccaccacagtgagcctgcgcgctgtttct ggccgtaccacggtgccgcaagtgtttatcggtggtaaacacattggtggcagcgacgac ttagaagtctacctaaatcaataa |
| A3EB97 | UniRef cluster | -------------------------------------------KYIAPQYKVQESVTIFTK-----PGCPYCA-KAKQALIDAG---LQYEE----LILG---KDATTVSLRAVS----GRT----------TVPQVFIGGK--------HIGGSDD----LEVYLNQ-------------------------- | atgaggaacacaatgtttacatctaaagaaggtcaaaccattccacaggttacttttcct actcgccaaggtgacgcttgggtcaatgtgactagcgatgaactgttcaaaggcaaaacc gttatcgtgtttagcttgccgggtgcctttactccaacctgttcatccactcacctaccg cgctacaacgagctgtttcctgtctttaaagagcatggtgtcgacagcattctgtgcgta tcggtcaacgatactttcgtgatgaatgcttggaaagatgaccaaaatgccgacaacatc accttcattcctgatggtaatggtgaatttaccgatggtatgggcatgctggtggataaa aatgaccttggctttggtaaacgctcatggcgctacagcatgctggttaaagacggtgtg gtagaaaaaatgtttatcgaaccgaatgagccgggcgacccgttcaaagtatcggacgcc gataccatgctcaaatacattgcccctcaatacaaggtgcaagaatcagtgactattttc actaagccaggctgtccttattgcgccaaggcgaaacaagcgctgattgatgccggtcta cagtatgaagagctgattttaggtaaagacgctaccacagtgagtctgcgcgccgtttct ggccgtaccacggtgccgcaagtgtttatcggtggtaaacacattggtggtagcgacgac ttagaagtctacctaaatcaataa |
| A2PVZ9 | UniRef cluster | -------------------------------------------KYIAPQYKVQESVTIFTK-----PGCPYCA-KAKQALIDAG---LQYEE----LILG---KDATTVSLRAVS----GRT----------TVPQVFIGGK--------HIGGSDD----LEVYLNQ-------------------------- | atgaggaacacaatgtttacatctaaagaaggtcaaaccattccacaggttacttttcct actcgccaaggtgacgcttgggtcaatgtgactaccgatgaactgttcaaaggcaaaacc gttatcgtgtttagcttgccgggtgcctttactccaacctgttcatccactcacctaccg cgctacaacgagctgtttcctgtctttaaagagcatggtgtcgacagcattctgtgcgta tcggtcaacgatactttcgtgatgaatgcttggaaagatgaccaaaacgccgacaacatc accttcattcctgatggtaatggtgaatttaccgatggtatgggcatgttggtggataaa aatgaccttggctttggtaaacgctcatggcgctacagcatgctggttaaagacggtgtg gtagaaaaaatgtttatcgaaccgaatgagccgggcgacccgttcaaagtatcggacgcc gataccatgctcaaatacattgcccctcaatacaaggtgcaagaatcagtgactattttc actaagccaggctgcccttattgcgccaaggcgaaacaagcgctgattgatgccggtcta cagtatgaagagctgattttaggtaaagacgccaccacagtgagcctgcgcgctgtttct ggccgtaccacggtgccgcaagtgtttatcggtggtaaacacattggtggcagcgacgac ttagaagtctacctaaatcaataa |
| A2PLL7 | UniRef cluster | -------------------------------------------KYIAPQYKVQESVTIFTK-----PGCPYCA-KAKQALIDAG---LQYEE----LILG---KDATTVSLRAVS----GRT----------TVPQVFIGGK--------HIGGSDD----LEVYLNQ-------------------------- | atgaggaacacaatgtttacatctaaagaaggtcaaaccattccacaggttacttttcct actcgccaaggtgacgcttgggtcaatgtgactagcgatgaactgttcaaaggcaaaacc gttatcgtgtttagcttgccgggtgcctttactccaacctgttcatccactcacctaccg cgctacaacgagctgtttcctgtctttaaagagcatggtgtcgacagcattctgtgcgta tcggtcaacgatactttcgtgatgaatgcttggaaagatgaccaaaatgccgacaacatc accttcattcctgatggtaatggtgaatttaccgatggtatgggcatgctggtggataaa aatgaccttggctttggtaaacgctcatggcgctacagcatgctggttaaagacggtgtg gtagaaaaaatgtttatcgaaccgaatgagccgggcgacccgttcaaagtatcggacgcc gataccatgctcaaatacattgcccctcaatacaaggtgcaagaatcagtgactattttc actaagccaggctgtccttattgcgccaaggcgaaacaagcgctgattgatgccggtcta cagtatgaagagctgattttaggtaaagacgctaccacagtgagtctgcgcgccgtttct ggccgtaccacggtgccgcaagtgtttatcggtggtaaacacattggtggtagcgacgac ttagaagtctacctaaatcaataa |
| A2P6Y2 | UniRef cluster | -------------------------------------------KYIAPQYKVQESVTIFTK-----PGCPYCA-KAKQALIDAG---LQYEE----LILG---KDATTVSLRAVS----GRT----------TVPQVFIGGK--------HIGGSDD----LEVYLNQ-------------------------- | atgaggaacacaatgtttacatctaaagaaggtcaaaccattccacaggttacttttcct actcgccaaggtgacgcttgggtcaatgtgactagcgatgagcttttcaaaggcaaaacc gttatcgtgtttagcttgccgggtgcctttactccaacctgttcatccactcacctaccg cgctacaacgagctgtttcctgtctttaaagagcatggtgtcgacagcattctgtgcgta tcggtcaacgatactttcgtgatgaatgcttggaaagatgaccaaaacgccgacaacatc acctttattcctgatggtaatggtgaatttaccgatggtatgggcatgctggtggataaa aatgaccttggctttggtaaacgctcatggcgctacagcatgctggttaaagatggtgtg gtagaaaaaatgtttatcgaaccgaatgagccgggcgacccgttcaaagtatcggacgcc gataccatgctcaaatacattgcccctcaatacaaggtgcaagaatcagtgactattttc actaagcctggatgtccttattgcgccaaggcgaaacaagcgctgattgatgccggtcta cagtatgaagagctgattttaggtaaagacgccaccacagtgagcctgcgcgctgtttct ggccgtaccacggtgccgcaagtgtttatcggtggtaaacacattggtggcagcgacgac ttagaagtctacctaaatcaataa |
| A1F753 | UniRef cluster | -------------------------------------------KYIAPQYKVQESVTIFTK-----PGCPYCA-KAKQALIDAG---LQYEE----LILG---KDATTVSLRAVS----GRT----------TVPQVFIGGK--------HIGGSDD----LEVYLNQ-------------------------- | atgaggaacacaatgtttacatctaaagaaggtcaaaccattccacaggttacttttcct actcgccaaggtgacgcttgggtcaatgtgactagcgatgaactgttcaaaggcaaaacc gttatcgtgtttagcttgccgggtgcctttactccaacctgttcatccactcacctaccg cgctacaacgagctgtttcctgtctttaaagagcatggtgtcgacagcattctgtgcgta tcggtcaacgatactttcgtgatgaatgcttggaaagatgaccaaaatgccgacaacatc accttcattcctgatggtaatggtgaatttaccgatggtatgggcatgctggtggataaa aatgaccttggctttggtaaacgctcatggcgctacagcatgctggttaaagacggtgtg gtagaaaaaatgtttatcgaaccgaatgagccgggcgacccgttcaaagtatcggacgcc gataccatgctcaaatacattgcccctcaatacaaggtgcaagaatcagtgactattttc actaagccaggctgtccttattgcgccaaggcgaaacaagcgctgattgatgccggtcta cagtatgaagagctgattttaggtaaagacgctaccacagtgagtctgcgcgccgtttct ggccgtaccacggtgccgcaagtgtttatcggtggtaaacacattggtggtagcgacgac ttagaagtctacctaaatcaataa |
| A1EMB6 | UniRef cluster | -------------------------------------------KYIAPQYKVQESVTIFTK-----PGCPYCA-KAKQALIDAG---LQYEE----LILG---KDATTVSLRAVS----GRT----------TVPQVFIGGK--------HIGGSDD----LEVYLNQ-------------------------- | atgaggaacacaatgtttacatctaaagaaggtcaaaccattccacaggttacttttcct actcgccaaggtgacgcttgggtcaatgtgactagcgatgaactgttcaaaggcaaaacc gttatcgtgtttagcttgccgggtgcctttactccaacctgttcatccactcacctaccg cgctacaacgagctgtttcctgtctttaaagagcatggtgtcgacagcattctgtgcgta tcggtcaacgatactttcgtgatgaatgcttggaaagatgaccaaaatgccgacaacatc accttcattcctgatggtaatggtgaatttaccgatggtatgggcatgctggtggataaa aatgaccttggctttggtaaacgctcatggcgctacagcatgctggttaaagatggtgtg gtagaaaaaatgtttatcgaaccgaatgagccgggcgacccgttcaaagtatcggacgcc gataccatgctcaaatacattgcccctcaatacaaggtgcaagaatcagtgactattttc actaagcctggatgtccttattgcgccaaggcgaaacaagcgctgattgatgccggtcta cagtatgaagagctgattttaggtaaagacgccaccacagtgagcctgcgcgctgtttct ggccgtaccacggtgccgcaagtgtttatcggtggtaaacacattggtggcagcgacgac ttagaagtctacctaaatcaataa |
| Q6LVG4 | UniRef cluster | -------------------------------------------NYIAPNHKLQESITVFSK-----PGCPFCA-KAKQNLIDKG---LQYEE----VVLG---KDATTVSLRAIT----GRS----------TVPQVFIGGK--------HIGGSEE----LETYLA--------------------------- | atgttcgcatctaaagaaggtcaggctgtaccacaagtaacattccatactcgccaaggc gatgcatgggtagatgttacaacagaagagctattcgcaaacaagactgtggttttattt agcctaccaggtgcatttacgccgacttgttcttcaagccacctacctcgctataacgag ctagcatcagtatttgcagagcatggcgttgatgagattgtgtgtgtgtcagttaacgat acgtttgtaatgaacgcatggaaagcagaccaagaagcagaaaacatcacattcattcca gatggtaacggtgaattctcgaaaggcatggacatgctggttgataaagaagacttaggt tttggacctcgttcatggcgttacagcatgctggttaaaaacggcgttgttgaaaaaatg tttgttgagcaagaagaaccaggcgacccgttcaaagtctctgatgctgatactatgctt aactacattgcaccaaaccacaagctacaagaatcaatcaccgtattctctaagcctggt tgtccattctgtgcaaaagcaaagcagaacctaatcgacaaaggccttcagtacgaagaa gtggtattaggtaaggatgcaacaactgtcagcctacgtgctatcacaggtcgcagcacc gttcctcaggtattcatcggtggtaagcacatcggtggcagtgaagaactagaaacatac ttagcttaa |
| Q1Z007 | UniRef cluster | -------------------------------------------NYVAPNHKLQESITVFSK-----PGCPFCA-KAKQNLIDKG---LQYEE----VVLG---KDATTVSLRAIT----GRS----------TVPQVFIGGK--------HIGGSEE----LETYLA--------------------------- | atgttcgcatctaaagaaggtcaggctgtaccacaagtaacgttccacactcgtcaaggc gatgcatgggtcgatgttacaacagaagaactattcgcaaacaagacagtggttttattt agcctaccaggtgcttttacaccgacgtgttcttcaagccacttacctcgctacaacgag ctagcatcagtatttgcagaacacggcgttgatgagattgtgtgtgtgtcagtgaacgat acgtttgtaatgaacgcatggaaagcagaccaagaagcagaaaacatcacattcattcca gatggtaacggtgaattctcgaaaggcatgaacatgctggttgataaagaagacttaggt tttggacctcgttcatggcgttacagcatgctagttaaaaacggcgtggttgaaaaaatg tttgttgagcaagaagaaccaggcgacccgttcaaagtctctgatgctgatactatgctt aactacgttgcaccaaaccacaaactacaagaatcaatcaccgtattctctaagcctggt tgtccattctgtgcaaaagcaaagcagaacctaatcgacaaaggccttcagtacgaagaa gtggtattaggtaaagatgcaacaactgtcagcctacgtgctatcacaggtcgcagcaca gttcctcaggtattcatcggtggtaagcacatcggcggcagtgaagaattagaaacatac ttagcttaa |
| A8T9N1 | UniRef cluster | -------------------------------------------NYVAPEYKTQESITVFTK-----PGCPFCM-KAKQNLIDHG---LQYEE----VVLG---KDATTVSLRAIS----GRA----------TVPQVFIGGK--------HIGGSEE----LETYLS--------------------------- | atgtttgcatcaaaagaaggtcaagctgtaccacaagttacctttccaactcgtgagggt gacgcgtgggtgaatgtcaccacggatgaactatttaaagacaaaacggttatcgtattc agcttgccaggtgcatttactccaacatgttcgtcaagccacctacctcgttacaacgag ttgttctccgtatttaaagagcatggtgtggatgaaattgtctgtgtgtcagtcaatgat acgtttgtaatgaacgcgtggaaagctgaccaagaagcagaaaacatcaccttcatcccc gatggtaacggagagttcacggacggcatgggtatgctggtggacaaaaacgacatcggc tttggcaagcgttcgtggcgttacagcatgctagtgaaaaatggcgtggttgaaaaaatg tttattgaaccaaacgagccaggcgacccgttcaaagtctcggatgcagacaccatgatg aattacgtggcacctgagtacaaaactcaagaatcgatcaccgtgttcaccaaaccaggt tgtcctttctgcatgaaggcgaaacaaaacctaattgaccatggccttcaatatgaagag gtcgttttgggtaaagatgcaacaaccgtgagtctgcgggcaatctctggccgtgcgacg gttcctcaagtcttcatcggtggtaaacatatcggtggtagcgaagagctagaaacttac ctaagctaa |
| GLRX4 | UniRef cluster | -------------------------------------------NARLVKLVQAAPVMLFMKGSPSEPKCGFSR-QLVGILREHQI---RFGF---FDILR---DENVRQSLKKFS----DWP----------TFPQLYINGE--------FQGGLDI----IKESIEEDPEYFQHALQ---------------- |  |
| A6ZRD0 | UniRef cluster | -------------------------------------------NARLVKLVQAAPVMLFMKGSPSEPKCGFSR-QLVGILREHQI---RFGF---FDILR---DENVRQSLKKFS----DWP----------TFPQLYINGE--------FQGGLDI----IKESIEEDPEYFQHALQ---------------- | atgactgtggttgaaataaaaagccaggaccaatttacgcaactaaccactacaaacgct gctaataaactcattgtcttatattttaaagctcaatgggctgatccttgcaaaagtatg agccaggtgctagaagctgttagtgaaaaagttaggcaagaggatgtccggtttttatca atagatgcagacaaacatccagaaatatcagatctttttgagattgcagccgtaccatac ttcgtcttcattcaaaatggtactattgtgaaagaaatatcaggcgcagatcctaaggag tttgtgaaaagcttagaaattctttcgaatgcttctgcctcactagcgaacaatgccaag ggtcctaaatctacgtctgatgaggaaagcagcgggtcttccgatgatgaagaggacgaa actgaagaagaaataaatgctaggctggtgaagctagtacaagctgcacctgtgatgcta ttcatgaaaggaagcccatcagaacctaaatgcggattttctagacagttagttggtatc ctcagagaacaccaaataaggttcggattttttgatatattaagagacgaaaacgttaga caaagcttgaagaagttttctgattggcctacttttcctcagttatatatcaatggggag ttccagggaggtttggatattatcaaggaatctatagaagaagatcctgaatatttccaa catgctctacagtaa |
| GLRX4 | UniRef cluster | -------------------------------------------NARLVKLVQAAPVMLFMKGSPSEPKCGFSR-QLVGILREHQI---RFGF---FDILR---DENVRQSLKKFS----DWP----------TFPQLYINGE--------FQGGLDI----IKESIEEDPEYFQHALQ---------------- |  |
| A6ZRD0 | UniRef cluster | -------------------------------------------NARLVKLVQAAPVMLFMKGSPSEPKCGFSR-QLVGILREHQI---RFGF---FDILR---DENVRQSLKKFS----DWP----------TFPQLYINGE--------FQGGLDI----IKESIEEDPEYFQHALQ---------------- | atgactgtggttgaaataaaaagccaggaccaatttacgcaactaaccactacaaacgct gctaataaactcattgtcttatattttaaagctcaatgggctgatccttgcaaaagtatg agccaggtgctagaagctgttagtgaaaaagttaggcaagaggatgtccggtttttatca atagatgcagacaaacatccagaaatatcagatctttttgagattgcagccgtaccatac ttcgtcttcattcaaaatggtactattgtgaaagaaatatcaggcgcagatcctaaggag tttgtgaaaagcttagaaattctttcgaatgcttctgcctcactagcgaacaatgccaag ggtcctaaatctacgtctgatgaggaaagcagcgggtcttccgatgatgaagaggacgaa actgaagaagaaataaatgctaggctggtgaagctagtacaagctgcacctgtgatgcta ttcatgaaaggaagcccatcagaacctaaatgcggattttctagacagttagttggtatc ctcagagaacaccaaataaggttcggattttttgatatattaagagacgaaaacgttaga caaagcttgaagaagttttctgattggcctacttttcctcagttatatatcaatggggag ttccagggaggtttggatattatcaaggaatctatagaagaagatcctgaatatttccaa catgctctacagtaa |
| GLRX3 | UniRef cluster | -------------------------------------------NARLTKLVNAAPVMLFMKGSPSEPKCGFSR-QLVGILREHQV---RFGF---FDILR---DESVRQNLKKFS----EWP----------TFPQLYINGE--------FQGGLDI----IKESLEEDPDFLQHALQS--------------- |  |
| A6ZY62 | UniRef cluster | -------------------------------------------NARLTKLVNAAPVMLFMKGSPSEPKCGFSR-QLVGILREHQV---RFGF---FDILR---DESVRQNLKKFS----EWP----------TFPQLYINGE--------FQGGLDI----IKESLEEDPDFLQHALQS--------------- | atgtgttcttttcaggttccatctgcattttcttttaactacacctcgtactgttataaa cgccaccaagcaagatattacacagcagcaaaactttttcaggaaatgcctgttattgaa attaacgatcaagagcaatttacttacctaactaccactgcggccggcgacaagttaatc gtgctttatttccataccagttgggcagaaccatgcaaagcattaaagcaggtttttgag gccattagtaatgagccttccaattccaacgtctctttcttatccattgatgcggacgaa aactcggaaatttcagaactttttgaaatctcagctgttccatattttatcataattcac aaagggacaatcttaaaagaattatccggcgcggatccaaaggagtttgtgtccttatta gaagactgcaagaactcagtcaattccggatcatcacaaactcatactatggaaaatgca aacgtaaatgaggggagtcataacgatgaagacgatgacgacgaagaagaggaagaagaa actgaggagcaaataaacgctagattgactaaattggtcaatgccgcgccggtaatgtta tttatgaaggggagcccctctgaacctaaatgcgggttttcgagacaacttgtgggtatt ttgagagaacatcaagtaagatttggcttctttgatatattaagagacgaatctgttaga caaaacttgaaaaagttttctgaatggccaactttccctcaactttatataaatggggag tttcaaggcggtttagacattatcaaggaatccttggaggaagaccctgattttttgcag catgctctccaatcttaa |
| GLRX3 | UniRef cluster | -------------------------------------------NARLTKLVNAAPVMLFMKGSPSEPKCGFSR-QLVGILREHQV---RFGF---FDILR---DESVRQNLKKFS----EWP----------TFPQLYINGE--------FQGGLDI----IKESLEEDPDFLQHALQS--------------- |  |
| A6ZY62 | UniRef cluster | -------------------------------------------NARLTKLVNAAPVMLFMKGSPSEPKCGFSR-QLVGILREHQV---RFGF---FDILR---DESVRQNLKKFS----EWP----------TFPQLYINGE--------FQGGLDI----IKESLEEDPDFLQHALQS--------------- | atgtgttcttttcaggttccatctgcattttcttttaactacacctcgtactgttataaa cgccaccaagcaagatattacacagcagcaaaactttttcaggaaatgcctgttattgaa attaacgatcaagagcaatttacttacctaactaccactgcggccggcgacaagttaatc gtgctttatttccataccagttgggcagaaccatgcaaagcattaaagcaggtttttgag gccattagtaatgagccttccaattccaacgtctctttcttatccattgatgcggacgaa aactcggaaatttcagaactttttgaaatctcagctgttccatattttatcataattcac aaagggacaatcttaaaagaattatccggcgcggatccaaaggagtttgtgtccttatta gaagactgcaagaactcagtcaattccggatcatcacaaactcatactatggaaaatgca aacgtaaatgaggggagtcataacgatgaagacgatgacgacgaagaagaggaagaagaa actgaggagcaaataaacgctagattgactaaattggtcaatgccgcgccggtaatgtta tttatgaaggggagcccctctgaacctaaatgcgggttttcgagacaacttgtgggtatt ttgagagaacatcaagtaagatttggcttctttgatatattaagagacgaatctgttaga caaaacttgaaaaagttttctgaatggccaactttccctcaactttatataaatggggag tttcaaggcggtttagacattatcaaggaatccttggaggaagaccctgattttttgcag catgctctccaatcttaa |
| Q6CSU2 | UniRef cluster | -------------------------------------------GERLKKLTQAAPVMLFMKGTPSEPKCGFSR-QMVGILREHQI---RFGF---FDILK---DENVRQGLKKFS----DWP----------TFPQLYINGE--------FQGGLDI----IKESLEEDPEFFQHTLSA--------------- | atgggtgttattgatattacttctcaggatcaatttactgagttgactactactaaggcg ggtgacaagctattggtattgtatttctataccagttgggccgagccatgtgttgccgtt ggaaaagttgtggaagctctaagtgaagaacgttgcaataaggatgttgtattcttgagt atttctgctgaggaacaagttgaaatctctgagcttttcgaagtttcatctgtgccttat ttcatcttcattaaaagcggaacgatattgagagaaatgtcgggtgctgatccaaaggaa tttgttgctattttgaaccaattgaacgcttctggtactgacgataacgaatctggtgcg gctaattcttctgctgataatggagctggttccaatgtcgcttcttctagaacggaaaac gtttctaatgttggcccagggtcaccattgggtgaagataacgaagagcaagaagaaaca gaagaacagttgggcgaaaggctaaaaaaacttacacaagctgcacctgttatgctgttt atgaagggtacaccatccgagccaaagtgtggcttttctagacagatggttggaatattg agagaacatcaaatcagatttgggttcttcgatattttgaaggatgagaacgttagacaa ggtttgaagaaattttctgactggccaactttcccacagctatatatcaatggtgaattc caaggaggtttggatatcatcaaggagtcattagaggaagatccagagttttttcaacac actctgtcagcataa |
| Q6FSS7 | UniRef cluster | -------------------------------------------NMRLTKLVNAAPVMLFMKGNPSEPKCGFSR-QIVGILREHQI---RFGF---FDILR---DDTVRTNLKKFS----DWP----------TFPQLYINGE--------FQGGLDI----IKESLEEDPDFFQHTIRVNN------------- | atggaggggggaaggagggaaatcgaaggcaggagacactttttgaagaggaatatggtt gctgagattacgaaccaggaccagtttacggagctgactacgcagaatgtgcaggacaag ctgattgctatatatttccacacctcgtgggcggagccctgcaaggccatcagcgagttg taccgggctataagcgaagacgagtcgaacaaggacgtgtcgttcttggcggtggatgcg gacgagcaggctgagatagctgagctgtttgagataagcgcggtgccatacttcgtgctg atcaggaacgggaccatcctgaaggagctctccggtgccaaccccaaggagttccttgct gcgctggaggagtgcaggagagcgggcacagagtcgaagtcgaaggaaagcaacggcgcg gtagaggacgacgaagacagcgaggacagcgaggaagagaccgaggaagagatgaatatg aggctgacaaagctggtgaacgcagcacccgtgatgctgttcatgaagggtaacccatcc gagcctaagtgcgggttctccagacaaatcgtgggaattctaagggaacaccagatcaga ttcgggttcttcgacatattaagagacgacaccgtcagaaccaacttgaagaagttctca gactggcctacgttccctcaattatatatcaacggtgagttccaaggtggcctagatatc ataaaggaatccctagaggaagaccctgacttcttccaacacaccatacgagtaaataac tag |
| A7TRJ2 | UniRef cluster | -------------------------------------------LERLTKLTQAAPVMLFMKGSPSEPKCGFSR-QIVGILREHQV---RFGF---FDILK---DDSVRQGLKKFS----DWP----------TFPQLYINGE--------FQGGLDI----IKESLEEDSEFFQNALQS--------------- | atgccactaattaatattgttgatcaagatcaattctctagattgactactaccgaagct aatggtaagattgttgtattgtatttttatgctacttgggctgaaccttgtaaagctatt aatgatgttgtaaaggcattaagtgatgagccaacaaaccataatgttcaattcttatct attgatgcagatgaacattctgaaattgctgaactgttcaatgtttctgcagtacctttt ttcgtcatcattcaaaatgaaactgttttgaaagaattgtcaggagctgatccaaaggaa tttgtttctgctttaaatgagtgtaagacagctgctttaggtgcagggtcatcttctact tctgctgctttaaatgatgaagctagagttagtgatgatgaggatgaggatgaagaagaa gaaacagaagaagaacttttggaaaggttgactaaattgactcaagccgcacctgtcatg ttattcatgaagggcagtccttctgaacctaaatgtgggttttcaagacaaatagttggt atcctaagagaacatcaagttaggtttgggttctttgatatattgaaggatgactctgtc agacaaggtttgaaaaagttctccgattggccaactttcccacaattatatattaacggt gaatttcaaggtggtttggatattattaaagaatcgttggaagaagattcagaattcttt caaaatgctcttcaatcttaa |
| Q74ZT7 | UniRef cluster | -------------------------------------------QERLKKLTSAAPVMLFMKGTPSEPKCGFSR-QMVGILREHQV---RFGF---FDILK---DESVRQGLKTFS----DWP----------TFPQLYINGE--------FQGGLDI----IKESLEEDPEFFQNTLSS--------------- | atgacagtcatcgatatcacatccaaggaccagttcacgcaacagactacggtggctgcc ggctctaagcttgtggcgctgtacttccacacagcctgggccgagccatgcaaggccatg agcgccgtgttttctgctgtgagtgaggaaccggcgcacaaagatgtcctattccttgcg attgatgcagacgagcatgcggaaatctctgagctgtttgaggtggcggcggttccgtac ttcgtcttgatcaaggagggggccatcgtaaaagagatttctgggtcggaccccaaagac ttcgtcagcgcattaaatgaatacaccgccggtagcaactccacaggcgctgccgccact gcatctgcacctgctgaagagtatggtgaggaggaaacagaggaacaactacaggaacgg ctcaagaagctgaccagcgctgctcctgtaatgttattcatgaaaggaacaccatctgaa ccgaaatgtggtttctccaggcagatggttggtatcttacgtgagcaccaagtgaggttt ggcttttttgacattcttaaggacgagagtgtgagacaggggctgaagacgttttcagat tggcccacgttcccgcaattgtacatcaatggtgagttccaaggtggcctagatatcatc aaggagtccttggaggaagaccctgagtttttccaaaacactctaagctcctga |
| Q5AF81 | UniRef cluster | -------------------------------------------NERLNKLTKAAPIMLFMKGSPSSPQCGFSR-QLVAILREHQV---RFGF---FDILK---DDSVRQGLKKFS----DWP----------TFPQLYINGE--------FQGGLDI----IKESIEDDEKFFEHALEA--------------- | atgggagttattgaaatcgagtcagaacaacagttcactgaattaaccaaagccgatcca tccaagttgattgcactttatttccatacaccatgggcaggaccttgtaaaaccatgaat caagtattcaaaaccttagctgattccaaagaatcagataattcaataatctttttaagt ataaatgctgatgagttaccggaaatcagtgaaatctttgaagttagtgcagttccttat ttcatattgattcgtaatcaaaccatattgaaagaattatcaggagctgatcctaaagaa tttattcaagcattaaaccaattttccaataacaccaacagcactactaccagtaataac gataacgtccaggcatcaattaacagcacaactgccaataccaatagtaataataccacc actaatgctcctgaagtggaggaatcagaggaagcattaaatgaacgattaaataaatta actaaagctgctccaatcatgttgtttatgaaaggttcaccatcttcaccacaatgtgga ttttctcgtcaattagtggcaattttaagagaacatcaagttagatttggatttttcgat attttaaaagatgattcagtaagacaaggtttaaaaaaattcagtgattggccaactttc cctcaattatacattaatggtgaattccaaggtggattggatatcatcaaagaaagtatt gaagatgatgagaaattcttcgaacatgctttagaagcttga |
| A5E3H6 | UniRef cluster | -------------------------------------------NERLSKLTSAAPIMLFMKGSPSSPQCGFSR-QLVAILREHQV---RFGF---FDILK---DDSVRQGLKKFS----DWP----------TFPQLYINGE--------FQGGLDI----IKENIEEDEQFFEHALNSNE------------- | atgggcgttatagagattgaaagtgaagaacagttcacagagattactcagtcatccgac tcaaagcttcttgctttgtacttccacaccccctgggctggtccttgtaagaccatgaac caagtattcaaaaccttggctgactctaaagcagacgatagctcaatagtatttctttca ataaatgcagacgacttgtctgaaatcagcgagttgtttgatgttagtgctgttccatat ttcatcttgattagaaagcaaacaatcttgaaagaattatccggcgcagacccaaaggaa tttatcgcagcattaaaccaattttctggatcaagtgattccaactctgcagcatcagcc acctcagtcgcaaccaatacaaacaacacagcttcaggttcagcagcagatccagcacaa gcagaacccgtggaggagtccgaggaggcgcttaacgaaaggttatcaaaactcacatcg gcagctcctataatgttgtttatgaaaggatctccatcgagtccacaatgtggattttcg cgacagttagtggcaatattgagagagcaccaagtgagatttgggttctttgatatttta aaggacgattcagttaggcaaggattgaagaaatttagtgactggccaacgtttcctcaa ctatacatcaatggagaattccaaggtgggttggacatcattaaagagaatatagaagag gatgaacaatttttcgagcatgctttaaattcaaatgaatga |
| A3GGH5 | UniRef cluster | -------------------------------------------DERLKKLTAAAPIMLFMKGSPSSPQCGFSR-QMVAILREHQV---RFGF---FDILK---DDSVRQGLKKFS----DWP----------TFPQLYINGE--------FQGGLDI----IKESIEEDDQFFERALAA--------------- | atggccgtcatcgagatccagtcggaagctcaattttccgagttgaccaagtcggaccct tccaagcttattgcgctctacttccacacaccatgggctgggccatgtaagacaatgaac tctgtttttaagactttggctgattctaaagcggccgatccaactatcctctttctcagc attaatgccgacgacctctcggaaataagtgaggtttttgaagtttccgctgttccatac ttcattttgatccgcaactcgaccatattgaaagagttatcgggcgctgaccccaaggaa tttataaacgctttgaaccagttttcaaactcagctccggctgctgttgctgccgccgca aaggaagaagctcctattaataatggtgtaggaaatggagctggagatgtttctgaatca gaagaagcccttgatgaaagattaaagaagttgactgcagccgctccaataatgttgttc atgaaaggttcgccttcctcgcctcaatgtggcttttccagacaaatggttgctatcttg agagaacaccaggtcagatttggattttttgatatcttgaaggacgactccgtcagacaa ggcttgaagaagtttagcgactggcccactttccctcaattgtatatcaacggagagttc cagggaggtttggacatcattaaggaaagtattgaggaagatgaccagttcttcgagaga gctttggctgcgtaa |
| Q6CES4 | UniRef cluster | -------------------------------------------NARLAKLVKAAPVMLFMKGTPAAPQCGFSR-QLVAILREHHV---RFGF---FDILK---DDAVRQGLKKFS----DWP----------TFPQLYIGGE--------LQGGLDI----VKESIQEDPEFFEKAVAE--------------- | atgtcagtcgtggaaatcaccagcgactcgcacttctccgagctcaccagctcgctagct cccaccactctggtggccgtctacttccacactccttgggccgctccctgtgcccagatg aacagcgttttcaagtcgctgtcgaccctccattcgtcggtgctttttctgtccgtcaac gccgatgagctgccagagatttccgaatctttcgacatctcggccgtaccctactttgtc atgctcagggacggcaccattctcaaggagttgagtggagccgaccccaaggagctggcc gccactatcagcgctctgagcgagagcgacgccaagcctgccgagtctgagtcggccgcc gccgctgcccccacttcttctactgctgacgcccctgccagctcatccacagacgctccc gaacccgcagaggagaccgaagaagaactcaacgcccgtctggccaaactcgtgaaggcc gcaccagtcatgctcttcatgaagggcacgccggcagctccacagtgcggcttctcacga caattggtcgccattctcagagaacatcacgtgcggttcggctttttcgacattctcaaa gatgacgccgtgcgacagggcctgaaaaagttttccgactggcccaccttcccccagctg tacattggaggcgagctgcagggcggtctggatatcgtcaaggagagcattcaggaggac cccgagtttttcgagaaggctgttgctgaatga |
| A5DIU5 | UniRef cluster | -------------------------------------------NERLKKLTSAAPVMLFMKGSPSAPQCGFSR-QLVAILREHQV---KFGF---FDILK---DDTVRQGLKTFS----DWP----------TFPQLYVNGE--------FQGGLDI----IKESIEEDPNFFASATAA--------------- | atgtcagttgtggaaatcacctccgaggcccagtttacggacttgacgcggtcggacccc gataagctcattgcgctctatttccacacaccatgggcccaaccatgtcaaactatgaac tcggtatttaagactttggctaaagaaaatccatcagttttattcatttcgattaatgcc gacgaccacgccgagatcagcgagctttttgaggtttctgcggttccctacttcattctc atcaagaactctaccataatcaaggagctttccggagcagatcccaaggaatttattgct gcattgaatcagctgtcaggaaagactgtaactccggcacccgagacatctgaaaagcct tcagtagagactgaggaatcaccagaggcactcaacgaaagattgaaaaaattgacgtct gctgcgcccgtaatgttgttcatgaaaggctcaccatcggctcctcagtgtggcttttcc agacagctagttgccatattgagagaacatcaagttaaatttggattctttgatatcttg aaggatgatactgtgagacagggcttgaagacgttttctgattggccaacattcccccaa ttgtatgttaatggagagttccagggtgggttggatatcataaaggagtctatcgaagag gaccccaatttctttgcttctgctaccgcagcctga |
| Q6BP74 | UniRef cluster | -------------------------------------------NERLKKLTGAAPVMLFMKGSPSAPQCGFSR-QLVAILREHQV---RFGF---FDILK---DDSVRQGLKNFS----DWP----------TFPQLYISGE--------FQGGLDI----VKESIEEDSSFFENAVKSA-------------- | atgtcagtgatagaaatagagtctgagtcgcagttcacagagttaactaaatcggacgct aataagttgattgcattgtatttccatacaccatgggcaggaccatgccaaacgatgaac tcagtgtttaagacgttagcagaggccaacaagtcggttttgttcatatcgatcaacgca gacgaccacccggacataagcgagcttttcgaggtgtcggcggtgccatattttatattg attcgtaactcgactattttgaaggagttgtcgggtgcagaccctagggaattcataagt gcgttgaaccagttcacagagaagaaggacacggctgctccagcagcagccgctgctgct gctccggcggcaccggcacaatccccaatcgaggagtcgccggaagcgttgaacgagagg ttgaagaagttgacgggtgcagcaccagtgatgcttttcatgaaggggtcgccatcggct ccgcaatgtgggttttcgagacaactcgtggcgattttgagagaacaccaggtcagattt gggttctttgacatcttgaaggacgattctgtcagacagggtctcaagaacttttcggac tggccaacgttcccccaattatacatcagcggagaattccaagggggacttgatatcgtt aaggaatcgatcgaggaagattcgagcttcttcgagaatgcggttaaaagtgcttag |
| Q6FKF5 | UniRef cluster | -------------------------------------------TESLDKLVNAAPVMVFIKGSPSDPKCGFSR-QMVNILRSHQI---RFGF---FDILK---DKITRQQLKDYS----DWP----------TFPQLYINGE--------FEGGLDI----VKESLLEDPDFFNKKLSL--------------- | atgtctgttgttgatatagataatgtggagcagtttctggatttggccaggacgaaagct ggtaataagctagtggtgttgtttttttacatgccttggatgaagtcatgtagagtcttg aaggaagtggtgctggcgttgagcagatcagtaggtaagaaacattccacatttttacag attaatatagaaggtaatagagacattgtgtctcttctgggtatcacacaggttccagct ttcttcctggtgagaaaaggtgttgtgataaaagcactaagcggtgttgatcccagagag tttttagcagcataccatgaatgtgtagataattccaactgctatgaccatgaccttgac gaagactcccaagatatcaaatcggatgatacattacaggagcccgaagatttctactct gaagataccgaaagtttagataaactggtaaacgctgcaccagtgatggtgttcataaaa ggaagcccctctgatccaaaatgtggtttctctagacaaatggtaaatatactgagatcc caccagatacggtttggtttttttgatatactaaaggataagattacaagacaacagcta aaggattactcagactggcccacattcccacaattatatattaatggcgaatttgaggga ggcctggatatagtaaaggaatcgttattggaagaccccgatttctttaacaagaagtta tcactgtaa |
| A2R5A2 | UniRef cluster | -------------------------------------------FARLAELVKAAPVMLFMKGSPSAPQCGFSR-QIVGILRERSV---KYGF---FNILA---DEDVRQGLKEFA----DWP----------TFPQLWVEGE--------LVGGLDI----VKEELENDPEFLDRFSVNKPAA----------- | atgtcctccctcgttgaagtcgcctctgaggccgaattccctaacatcatctcctccatt cccccgtcatgcctacaggttctctacttccacgccccctgggctgctccttgcgcccaa atgcgcgccgtcatctccgccctcgcctcccagtaccccgcgaccaaccctccctccatc gccttcctcagcgttaacgccgaggagctccctgatatctcggaagagtacaatgtcacc gctgttcccttcgtagtcctcgtccgggacaacaagattctcgaaaccattagcggcagt gaagcaatccaagtgcgcgatgccgttgagcgccacgccggtgccgctgccgccgcctct gcagacggtgctcccaagaccgtgatccccccaccattgacagccgtgcctcgagagaac gttcccgccaccgctacacaggcgcccgccacgaacggcaacgcagctgcccccgcattg acccccgaacagtcgaaggaggcgctgtttgcgcgtctcgcggagttggtgaaggctgcg ccggtgatgttgttcatgaagggatctcccagtgcgcctcagtgtggattcagtcggcag atcgtgggcattctgcgtgagcggagcgtcaagtacggattcttcaacatcttggccgat gaggatgtgagacagggcttgaaggagtttgcggattggcctaccttcccgcagttgtgg gtggagggtgagttggttggtggattggatattgtcaaggaggaacttgagaacgatccc gaattcctggaccgcttctccgttaacaagcccgctgcctag |
| Q5AVW3 | UniRef cluster | -------------------------------------------FARLSELVKAAPVMLFMKGTPSAPQCGFSR-QLVGILRERSV---KYGF---FNILA---DEDVRQGLKEFA----DWP----------TFPQLWVNGE--------LVGGLDI----VKEELENDPNFLDSFSINK-------------- | atgtcgaccctcttcgaagtcacctcagaggagagcttcgggcctcatcttgcctccatt ccgtccgatactctcatcgtcctctatttccacgcgccatgggccgccccctgcgcgcaa atgagagctgtgctctccgccctcgcctcccaatacccagccactacccctccaaccgtt tccttcatcagtgtcaacgctgaagaactccctgacatctccgaagaatacgacgtcacc gccgtcccttacgttgtcctcctccgcaacggccaagtcctcgaaacaatctccggcagt gaggcgacaaaggtgcgcgatgctgttgaacggtatgccggtgctggctcagcaggtgca agcgccaatggcgccgcatctgcaattccacctgcattgactgctgtgccgagagaggat gtgaatacgccaacgacggctacgcaagcacctgtggccggtgctgcttcgggtgctggc gccgcaccggccttgacgcccgagcagagcaaggaggcgctatttgctcgactttcggag ctggtgaaggctgcgcccgtcatgctattcatgaaggggacaccgagtgcaccgcagtgt gggttcagtcgacagttggttggtatcctgcgtgaacggagtgtcaaatacgggttcttc aacattcttgccgatgaggatgtgcgacagggtttgaaagagtttgcggactggcctact ttcccccagttatgggttaacggggagttagttggtgggctagatattgtcaaggaagaa ctcgagaatgatcccaatttcctggacagcttctccattaacaaataa |
| Q0CEP1 | UniRef cluster | -------------------------------------------FARLAELVKAAPVMLFMKGTPSAPQCGFSR-QLVGILRERSV---KYGF---FNILA---DEDVRQGLKEFA----DWP----------TFPQLWVNGE--------LVGGLDI----VKEEINNDPGFLAEFSVNKAPA----------- | atgtccagcctggtcgaggtcacatcggaggccgagttcgtctcgggcctgcaatccatc ccggattccaccctggtggtagtctacttccacgcgccctgggccgcgccttgcgcccaa atgcgcgccgttctctccgccctcgcctcgcagtaccccgtcacctccccgccttccatc gccttcctcagcgtcaacgccgaagagctcccggacatctcggaggaatacgacgtcacc gccgtgccctttgtcgtcctgctgcgcgccggaaaggtcctcgaatccatcagcggcagc gaggccgcccgcgtgcgtgacgccgtcgagcgtcacgccggcgtgggcgccggcgctccc tcctccgcctccgcgaccaccatccctccgccgctggaggctgtcccccgcgagaacggc ccggccaccgccacccagccgcccgtcaatggcgccgccacccccgaacagtccaaggag gcgctgtttgcccgtctcgccgagctggtcaaggccgcccccgtcatgctgttcatgaag ggcacgcccagtgcgccgcagtgcggattcagtcggcagttggtgggtatcctgcgcgag cgcagcgtcaaatacgggttctttaatatcttggccgatgaggatgtccggcagggcttg aaggagtttgccgactggccgactttcccgcagttgtgggtgaacggcgaattggttggt ggactggatatcgtcaaggaagagatcaacaacgaccccgggttcctcgcggaattttcg gtcaacaaggcgcccgctgcggcttaa |
| A1C748 | UniRef cluster | -------------------------------------------FARLAELVKAAPVMLFMKGTPSAPECGFSR-QLVAILRERSV---KYGF---FNILA---DEDVRQGLKEFA----DWP----------TFPQLWVGGE--------LVGGLDI----VKEEISNDPDFLGAYSVNKTSTAA--------- | atgtcgaccctcaacgaaatcacgtccgacgccgagttctcgacattcatttcctccctc ccatcttcggcactggcagtcctctacttccatgcaccatgggctgctccctgcgcccaa atgcgcgccgttctaaccgccctcgcctcccaataccccgtcaccaccccgccctcgatc tccttcgtgagcatcaacgcagaagagctccctgacatctcagaagactacgatgtcacc gccgttccctacgtcgtcctgcttcgtgacggcaagatcctcgaatccataagtggcggc gaggcagtcaaggtgcgcgacgcggtggagcgccatgccggtgccgcctcgggaaccggc gcaggcgccaacaaggccgctatccccccgccgctgacggctgtcccgcgcgagaacgtg ccttcgacggctacgcaggcgcccgccccgtcggcgcagcagctgtcgcctgaggagtcc aaggaggcgttgtttgcccgcttggcggagctggtgaaggcggcgccggttatgctattc atgaagggaaccccgagtgcgccggagtgcggtttcagtcgacagctcgtggcgattctc cgggagcggagcgttaagtatggattcttcaatattctggccgatgaggatgttagacag ggtttgaaagagtttgcggattggccgacatttcctcagctctgggttgggggagaattg gttggtggattggatattgtcaaggaagagatcagcaacgaccctgatttcctcggcgcg tactcagtcaacaagacctccactgcggcttga |
| Q4X024 | UniRef cluster | -------------------------------------------FARLAELVKAAPVMLFMKGTPSAPQCGFSR-QLVAILREKSV---KYGF---FNILA---DEDVRQGLKEFA----DWP----------TFPQLWVEGE--------LVGGLDI----VKEEINNDPDFLSQYSINKAPASA--------- | atgtcgacgctcaacgaaatcacctcggacgccgacttctcggcacacacctcctctctc cctccctccaccttactagtcctctacttccatgcgccgtgggcagctccctgcgcccag atgcgcgccgttctctccgccctcgcctcccagcacccggttactaccccgcccaccatc tcctttgtgagcgtcaacgctgaagaactccccgacatctccgaagagtacaacgtcaca gcagtcccctacgtggtccttatccgcaacggccagatcctcgaatccatcagcggcagc gacgccgtcaaggtgcgcgatgcggtcgagcgccacgcgggcgctggatcgggcgcgggt gcagacggcgcgaacaagacggctatccctcctcctttgacggctacgcctcgcgagaat gcccctgcggctgctgcgcaaccgcccgctccctcgacccaggcattgacgcccgagcag tccaaggaggcgctgtttgcacggttggcggagctggtgaaggccgcgccggttatgctc ttcatgaagggtacacctagtgcaccacagtgcggattcagtcgccagttggttgccatc ctgcgggagaagagcgtcaagtacgggttcttcaatattctggccgatgaggacgtaagg caggggttgaaggagtttgcggactggcctacatttcctcagttgtgggttgagggcgag ctggttggtggactagatatcgtcaaggaagagatcaacaacgaccctgatttcctgagc cagtactccattaacaaggctcctgccagcgcttga |
| B0XU77 | UniRef cluster | -------------------------------------------FARLAELVKAAPVMLFMKGTPSAPQCGFSR-QLVAILREKSV---KYGF---FNILA---DEDVRQGLKEFA----DWP----------TFPQLWVEGE--------LVGGLDI----VKEEINNDPDFLSQYSINKAPASA--------- | atgtcgacgctcaacgaaatcacctcggacgccgacttctcggcacacacctcctctctc cctccctccaccttactagtcctctacttccatgcgccgtgggcagctccctgcgcccag atgcgcgccgttctctccgccctcgcctcccagcacccggttactaccccgcccaccatc tcctttgtgagcgtcaacgctgaagaactccccgacatctccgaagagtacaacgtcaca gcagtcccctacgtggtccttatccgcaacggccagatcctcgaatccatcagcggcagc gacgccgtcaaggtgcgcgatgcggtcgagcgccacgcgggcgctggatcgggcgcgggt gcagacggcgcgaacaagacggctatccctcctcctttgacggctacgcctcgcgagaat gcccctgcggctgctgcgcaaccacccgctccctcgacccaggcattgacgcccgagcag tccaaggaggcgctgtttgcacggttggcggagctggtgaaggccgcgccggttatgctc ttcatgaagggtacacctagtgcaccacagtgcggattcagtcgccagttggttgccatc ctgcgggagaagagcgtcaagtacgggttcttcaatattctggccgatgaggacgtaagg caggggttgaaggagtttgcggactggcctacatttcctcagttgtgggttgagggcgag ctggttggtggactagatatcgtcaaggaagagatcaacaacgaccctgatttcctgagc cagtactccattaacaaggctcctgccagcgcttga |
| A6RF75 | UniRef cluster | -------------------------------------------FARLDELVKAAPVMLFMKGTPSAPQCGFSR-QLVGILRENGV---KYGF---FNILA---DEDVRQGLKEYA----DWP----------TFPQLWVKGE--------LVGGLDI----VKEEIATNPDFFKDYSVAPRT------------ | atgtcaaccctgaccgaaattgcttcggaagaagccttcaataatcatgtctcctccatc cctccatccgccctcttagttctctatttccacactccctgggctgcgccgtgcacccaa atgcgcactgtgctctccaccctcgcgtccacctatcccgccactactcctcccggcatc tcctttgtcagcgtaaacgctgaagagctcccagagatctctgaagaatatggcgttacg gcggtcccttttgttgtccttgtccgcgacggcaaaaccctccaagctctttccggctcc gacgcagtcaaagcccgcgtcgccatcgaacagcatgccggcgtggatgcaacaggcaac actaccggtgcgggaaagcaatccatccctcctcctcttcatgcagaaccaagaaaagat atctccacttccactcccagcacctctgatacccctctcgctaccacaaacggtgcagtc tcagccgccgccgacccatcgctgccacccacgaaagacgagcttttcgcccgtctcgat gagcttgtaaaggccgcgccagtcatgttatttatgaagggaacaccaagtgccccacag tgcggattcagcaggcagctcgtcggcattcttcgcgaaaatggtgtgaaatatggcttc ttcaatattttggcggacgaggacgtgaggcagggtttgaaggaatatgccgattggcct acgttcccccagctgtgggtgaagggagaattggtgggtggacttgatattgtcaaggaa gaaatcgccacaaatccagactttttcaaagactattcggtagcaccccgaacataa |
| A1DI55 | UniRef cluster | -------------------------------------------FARLAELVKAAPVMLFMKGTPSAPQCGFSR-QLVAILREKSV---KYGF---FNILA---DEDVRQGLKEFA----DWP----------TFPQLWVEGE--------LVGGLDI----VKEEINNDPDFLSQFSVNKAPA----------- | atgtcgacgctcaacgaaattacctcggacgccgacttctcggcacacacctcctctctt cctccctcagccttactagtcctctacttccatgcgccgtgggcagctccctgcgcccag atgcgcgccgttctctccgccctcgcctcccagcacccggttaccaccccgcccaccatc tccttcgtgagcatcaacgccgaagaactccccgacatctccgaagagtacaacgtcaca gcagtcccctacgtggtccttatccgcaacgtccagatcctcgaatccgttagcggcagc gacgccgtcaaggtgcgcgatgcggtcgagcggcacgcgggcgctggatcgggcgcgggc gcagacggcgcgaacaagacggctatccctcctcctttgacggctacgcctcgcgaaaat gcccctgcggctgctacgcaaccgcccgctccctcgacccaggcattgacgcccgagcag tccaaggaggcgctgtttgcacggttggcggagctggtgaaggccgcgccggttatgctc ttcatgaagggtacacctagtgcgccgcagtgcggattcagtcgccagttggttgccatc ctgcgggagaagagtgtcaagtacgggttcttcaatattctggccgatgaggacgtaagg caggggttgaaggagtttgcggactggcctacatttcctcagttgtgggttgagggggag ctggttggtggattagatatcgtcaaggaagagatcaacaacgaccctgatttcctgagc cagttctcagttaacaaagctcctgctaccgcttga |
| Q1E7N6 | UniRef cluster | -------------------------------------------FARLSELVKAAPVMLFMKGTPSAPQCGFSR-QIVSILRENGV---KYGF---FNILA---DEDVRQGLKEFA----DWP----------TFPQLWVKGE--------LVGGLDI----VKEEISANPDFFCDYSVSKPTS----------- | atgtccacccttcacgaaatcacttccgaagcggactttacgacgcagctctcttccctc ccatccacatctctagctgtcctctcatttcacactccctgggctgcgccatgcacgcaa atgcgcaacgtactgtccaccctcgcctccacctaccccgccactacccctccatccatt cacttcttgagcatcaacgccgaagacctccccgacatctcagagcaatacgacgtatcc gctgtgccatacctagttctccttcgagacaacaagattgttgagactgtgtccggctcc gatccagtcagggtccgggaggcgattgagaaacatgtggggcaagacgggcagacagat cgaccttccattccgccacctctggtcgcagtaccgagagcgacagcggcgcaggataca tccgacgacgctactgcttcagacgcgcagttagagcctcccgtgcccacgaaagaagag ctctttgcgcgcctttcagagcttgtaaaagccgccccggttatgttattcatgaaaggg actcccagcgccccacagtgtggattcagtaggcagattgtgagcattctgcgcgagaac ggggtgaaatatggattctttaatattctggcggatgaggacgttcggcaggggttgaaa gagtttgcggactggccgactttcccgcagctctgggtcaagggggagctggttggcgga ttggacattgtgaaagaagagatctccgcgaaccctgattttttctgcgactattcggtc tctaaacctacaagcgcgccctcagcttga |
| B2AYZ1 | UniRef cluster | -------------------------------------------FKRLGDLVKAAPVMLFMKGTPSEPKCGFSR-QLVAILRENAV---KYGF---FNILA---DDEVRQGLKEFA----DWP----------TYPQLWVDGE--------LVGGLDI----VKEELSNDADFFKAYSIKSNGE----------- | aacattgcacaccgacagccacgccttgcgagtcttggtctccccgtcacccgctcgaat atctaccaatacaacaacatgtcgactattcgcgaaatcaccagtctttcgaactgggaa catcacgtcacctcactaccgccttccactctcctcgtggtctctttccatgcgccatgg gccgctccatgcgcgcaaatggcgaccgttctgtcgacgctggcgagcgagtatcccgtc actgaaccaccgtccacatcatgggtgtcaatcaacgcagaggatctttcggatattagc gaaacctacaacgtgacagccgttccatttcttgtcctgattcgcaacggacaggtgctg gaaaccgtcagtggaagcagcgccgtcaaggtgcggaatgccatcgaagcccatgccgcc aaggccggcgccccagttttgaatggcgctgcgactgcgactgacggccatgatggtgag gtggcaaccgaggaggatccagagaagaaaaaggaggagctcttcaagcgtctgggagat cttgtcaaggctgctcccgtgatgctcttcatgaagggcactcctagcgagcccaagtgc ggcttttctcggcagctagtagctattctgcgggaaaacgccgtcaagtatggcttcttc aacatcctggccgacgatgaagtgcgccagggcctcaaagagtttgccgactggccaact tatccccagctctgggttgacggcgagctggtcggagggcttgatattgtcaaggaggaa ctgtctaatgacgccgacttcttcaaggcatatagcatcaagtccaacggcgagactgca gccgcctcgtga |
| Q7SDJ8 | UniRef cluster | -------------------------------------------FRRLGDLVKAAPVMLFMKGTPSEPKCGFSR-QLVAILRENAV---KYGF---FNILA---DDEVRQGLKEFA----DWP----------TYPQLWVDGE--------LVGGLDI----VKEELANDADFFKPYSVKANGDA---------- | atgtcgaccatcaaggacattaccagtatcgaggcttggaacaatcatgtgtcgtcattg ccggcgtccaccctcctgatcgtctccttccacgcgccctgggctgcgccctgcgcccag atggcgacagtgctcaagacattggcgagcgaatatcccgttactgaaccgctttccacg tcctgggtctcgatcgacgccgaagaactgtcggaaatcagcgaaacgtacaacgtgacg gccgttccctttctcgtgctcgcccggaataaccaggttttggagacggttagcggaagc agtgccgtcaaggttcgaaacgcgattgagacccacgccaaaaaatcggcccagttcggc actgaagctagtgagaagacaccggcgatcgcaaatggcgccgcaactgctgagggccag gccggcgacgttgtacctcaggaccccaagaagcaaaaggaggagttgttcagacgtctg ggtgacctggtcaaggccgccccggtcatgctgttcatgaagggtactcccagtgagccc aagtgcggcttctcgcgacagctggttgctattctccgagaaaatgccgttaaatacggt ttctttaacattctagcggacgacgaagtacgacagggccttaaggagtttgctgattgg cctacttatccacaactttgggtcgacggtgagctagtggggggactcgatatcgtcaag gaggagttggcgaacgacgccgacttcttcaaaccatatagtgtcaaggcgaacggcgat gcgtccgccgggcaatcatag |
| A6RRT7 | UniRef cluster | -------------------------------------------HARLSNLVKAAPVMLFMKGTPSAPQCGFSR-QLVALLREKSV---KYGF---FNILA---DDEVRQGLKEFA----DWP----------TFPQLWMDGE--------LVGGLDI----VKEEAANDPDFFTAYSVAKPAA----------- | atgtcaactcttatcgatattacaacagaggaagaatggcagaagcacactgaatctctg ccatcctcaactctgcagatcatcaactttcacgcaccatgggcggcaccatgcgcgcaa atgacaaccgtcctccgtaccctcgccctctcctaccccccaactacacctccaaccacg tcatgggtttccatgaatgccgaagaagttatttccgtctccgatgcctttgacgtaacc gcggttccctacctcgtcctcacccgcaacaatgttgtcctcgagaccgtctccggcagc gacgccaccaaagtccgcaacgccattgaaaaacacgccaactctccctcctcctccaca aatggtacatcagccaagcccgtccccagtataagccatgcctctaccgcaccatcaaca accgcacctaccacttctaccgaatccgaagaaccaatgtctaaagaagatcttcacgct cgtttatctaaccttgttaaggcggctcctgtgatgttattcatgaaaggaacaccttca gcaccccaatgcggattttcccgtcaactcgtcgctttactaagagagaagtcggtgaaa tatggattctttaatattttggccgatgatgaggtaaggcagggtcttaaggaatttgcg gattggcctactttcccacaattatggatggatggtgaacttgttggaggtctggatatt gtcaaggaagaagcagcaaatgatccggatttcttcactgcatatagtgttgctaaacca gcagcagcgtag |
| A4R8W2 | UniRef cluster | -------------------------------------------FKRLADLVKAAPVMLFMKGTPSAPQCGFSR-QLVALLRENSV---KYGF---FNILA---DDEVRQGLKEFA----DWP----------TYPQLWMDGE--------LVGGLDI----VKEEIGNDPDFFKAYSVKASTD----------- | atgtcaacgctcagagatatcaccactttggaggaatgggaggctcaccaggcctcactc ccggcctcaactcttcagattatatactttcatgcaccatgggcgccaccttgcgctcag atggcaacagtgcttaagaccctgtcatccgaatatccagtaaccgaccctcttgcgacc tcatgggtatccctcgacgccgaggacctgagcgacgtgagcgagacctacgacgtcacg gcggttccgttcgtcgttctttcgcgcggcggcaaggtcctcgagtctatcagcggtaac agcgcccagcgcgtgcgcactgcgatcgaaacgcataccagcagctccggccccggcgcg tcttcagcaggtgccccagtggcggctagcgacgaggccgccaaggccgagggtgcacct caagacccggagaagcagaaggaggagctgttcaagcggctggcggatttggtcaaggcg gcaccggtcatgctgttcatgaagggcacgcctagcgcgccgcagtgcggcttctcgagg cagctggtcgctctgctgagggaaaactcagtcaagtacggtttcttcaacatcctggcg gacgacgaggtcaggcaaggcctcaaagaattcgccgactggccgacatacccacagctc tggatggatggcgagctggttggcggactcgatattgtcaaggaggagatcggaaacgac ccggactttttcaaggcctatagcgtcaaggccagcacagatgctgcgggcacatcatag |
| A7EQN8 | UniRef cluster | -------------------------------------------HERLSNLVKAAPVMLFMKGTPSAPQCGFSR-QLVALLRENSV---KYGF---FNILA---DDEVRQGLKEFA----DWP----------TFPQLWMDGE--------LVGGLDI----VKEEAGNDPDFFKAYSVAKPATAA--------- | atgtcaagtcttatagaaattacaacggaggaagaatggcagaagcatattgaatctttg ccaccttcaactctgcagataatcagctttcatgcaccatgggcagcaccatgtgcgcaa atgtcggtcgtcctccgtaccctcgcgctctcctaccccgcaaccacaccacctaccacc tcctgggtttcgatcaacgccgaagaggtaatctccgtctctgatgccttcgacgtaacc gcagtccctttcctcgtcctcacccgcaacaacgctgtccttgaaaccgtctccggcagt gacgccgccaaagtgcgcagtaccattgaaaaacacgccaattctacctcttccactaac ggcacatctctgagacccgtaccaagcataagccatacttctcccgaaccatcaactcca cctactacttctaccgaatcccaagagccaacatctaaagaggatctccacgaacgctta tccaatcttgtaaaggcggctcctgtgatgctgtttatgaaaggaacaccttcagcacct cagtgtggtttctctcgtcaactcgtcgctttactaagagaaaactcggtgaaatatggc tttttcaatattttggccgatgatgaggttaggcagggtcttaaggaattcgcagattgg cctactttcccacaattgtggatggacggagaacttgtaggaggtctagatattgtcaaa gaagaagccggaaatgatccagatttcttcaaggcatatagcgtcgctaaaccggcgaca gcagcttag |
| Q0UVX2 | UniRef cluster | -------------------------------------------MKRLGELVKAAPVMLFMKGTPSAPQCGFSR-QTVSVLREKGV---RYGF---FNILA---DDEVRQGLKEYA----DWP----------TFPQLWVDGE--------LVGGLDI----VKEEFENDPEFLSQYSRSTKQR----------- | atggcgaccattcaagacgttgccgacgagcaggccttcctgtccaccatctccacgctg cccgcctccaccctcgccgtcatatacttccacgcgccatgggccaagccatgcgagcaa atgtccatcatcctcaagacgctcgccagcacataccccgccgacgcgcccatatcgttc ctcgcgctcaacgccgaagaagtcccagaggtttccgaagagtacgacgtcacagctgtt ccatacattgtgctgcagaaagacggcaagacgctagagacagtttcggggtccgatgcc gccaaggtacgagcggcggtggagaaatatgctggtgcgggctcaggctcaggtgactcc aaagcaaatctgccaccagcgcagacagttacacgaccagcgcagacgaatggcacggac agcgcgggcaagaacttggccggctatgcgccaggcgcccaagatccgaacactgcgccc gagtacagcgcgagcgagcaccgcgagggcgagcagcagacaaacaaggaggagctgatg aagaggctaggcgagcttgtcaaggcggcacccgtcatgctgtttatgaagggcacacca agcgcaccgcagtgtggcttcagcaggcagacggtcagcgtactgcgcgagaagggcgta cggtacggcttcttcaacatcctggccgacgacgaggttcggcagggtctgaaggagtat gcggattggccaactttcccacaattgtgggtggatggtgagctggttggcggtcttgat attgtcaaggaagaatttgagaacgaccccgagttcctctcacagtattcacgttcaaca aagcagcggcttagagacctcgacccacataccatctga |
| Q2HGB4 | UniRef cluster | -------------------------------------------FRRLGDLVKAAPVMLFMKGTPSEPKCGFSR-QLVAILRDNAV---KYGF---FNILA---DDEVRQGLKEFA----DWP----------TFPQLWVDGE--------LVGGLDI----VKEELDSNADFLKPYSVKANGDTSAVAA----- | atgtctaatatcgaagagatcaccagcgtggtggcttgggaaaagcacatatcgtcgttg ccgacatccacgcttctaatcgtctcgtttcatgcaccatgggcggcgccgtgtgcgcaa atggcaaccgtgctctcgacgctggctagcgaataccccgccaccgaaccgcggtcgact tcatgggtgaagatcaacgccgaggagctatcagatattagcgaagcctacgacgtgact gctgttccattcctcgtcctcatccggaacggcgaggtgctcgagacggtgagcgggagc agtgcggtcaaagtgcgagaggctattgaaaagcacgcgagcaaggcgggggcagcaacc actaccaacaatggtgccgcgccggcacccgggctcggcgaagcggaggtcaacacagag gaagacccagaaaagaagaaacaggaactctttaggcgcctcggtgatctcgtgaaagcg gcacccgtcatgctcttcatgaaagggacaccgagcgagcccaaatgcggcttttcgcgg caactggtggcgatcctgcgcgacaacgccgtcaagtacggcttctttaatatcttggcc gacgatgaggtgcggcaaggtttgaaagagtttgccgactggccgaccttcccgcagttg tgggtggacggcgaacttgtcggtgggcttgatatcgtcaaggaggagctggacagtaac gcggatttcctgaagccgtatagtgtcaaggcaaatggcgatacctcggccgtagcggct taa |
| Q5M993 | UniRef cluster | -------------------------------------------NGRLKKLINAAPCMLFIKGSPQEPRCGFSR-QIVDILNQHKI---QFSS---FDILS---DEEVRQGLKTFS----DWP----------TYPQFYVNGE--------LLGGLDI----VKEMVASGELDQ-MCPKAQNLE----------- | atggcggcggtgttagaggccggctctcagagccagtttgaggagctgctgcagaaaagt gccaagtctctaacagttgtgcatttttgggcaccatgggctccacagtgcacccaaatg aacgaagtcatggccgaactggcgaaggagcagcctcaggtcatgtttgtaaagcttgaa gctgaagccgttccagaagtctctgaaaaatacgaaattacctccgtgccgacctttttg ttttttaagaactcacagaagattgacagattggacggtgcccacgcgccggagctcaca aagagggttcagcgccatgcctcaagcacctcttttccagctacccccaacagtgctccc aaagaagacctcaatggcagactgaaaaagttgataaatgctgctccatgcatgctcttt attaagggatctccccaggagccaagatgtgggttcagcaggcagattgtggatatcctt aaccagcacaagatccagttcagcagctttgatatactgtcggacgaagaagtccgccag ggcctaaaaaccttttccgactggcccacttatccgcagttttacgtcaatggcgaactg ttgggaggactggatatcgtgaaggaaatggtggcatcaggagaattggaccaaatgtgc cccaaagcccagaatctagaggagaggctgaaggtgcttgtgaacaaagccccagtaatg ctgtttatgaaagggaacaaagagatggcaaaatgtggcttcagcaggcaaattctagaa ataatgaacaacaccggtgtcaattttgaaacgtttgatatactggaagatgaagaggtg cgacaaggtttaaaagcctactccaactggccgacctatcctcagctttacgtcaaagga gaacttgtcggggggctggacatcgttaaggagttaaaagaaaatggcgaatttgtttca gttctgaagggagatcagtga |
| Q5M993 | UniRef cluster | -------------------------------------------EERLKVLVNKAPVMLFMKGNKEMAKCGFSR-QILEIMNNTGV---NFET---FDILE---DEEVRQGLKAYS----NWP----------TYPQLYVKGE--------LVGGLDI----VKELKENGEFVS-VLKGDQ-------------- | atggcggcggtgttagaggccggctctcagagccagtttgaggagctgctgcagaaaagt gccaagtctctaacagttgtgcatttttgggcaccatgggctccacagtgcacccaaatg aacgaagtcatggccgaactggcgaaggagcagcctcaggtcatgtttgtaaagcttgaa gctgaagccgttccagaagtctctgaaaaatacgaaattacctccgtgccgacctttttg ttttttaagaactcacagaagattgacagattggacggtgcccacgcgccggagctcaca aagagggttcagcgccatgcctcaagcacctcttttccagctacccccaacagtgctccc aaagaagacctcaatggcagactgaaaaagttgataaatgctgctccatgcatgctcttt attaagggatctccccaggagccaagatgtgggttcagcaggcagattgtggatatcctt aaccagcacaagatccagttcagcagctttgatatactgtcggacgaagaagtccgccag ggcctaaaaaccttttccgactggcccacttatccgcagttttacgtcaatggcgaactg ttgggaggactggatatcgtgaaggaaatggtggcatcaggagaattggaccaaatgtgc cccaaagcccagaatctagaggagaggctgaaggtgcttgtgaacaaagccccagtaatg ctgtttatgaaagggaacaaagagatggcaaaatgtggcttcagcaggcaaattctagaa ataatgaacaacaccggtgtcaattttgaaacgtttgatatactggaagatgaagaggtg cgacaaggtttaaaagcctactccaactggccgacctatcctcagctttacgtcaaagga gaacttgtcggggggctggacatcgttaaggagttaaaagaaaatggcgaatttgtttca gttctgaagggagatcagtga |
| GLRX5 | UniRef cluster | -------------------------------------------NERLSTLTNAHNVMLFLKGTPSEPACGFSR-KLVGLLREQNV---QYGF---FNILA---DDSVRQGLKVFS----DWP----------TFPQLYIKGE--------FVGGLDI----VSEMIENGE--LQEMLPN--------------- |  |
| Q4S9D6 | UniRef cluster | -------------------------------------------NQRLKKLINAAPCMLFMKGSPQEPRCGFSR-QIVALLSEHNI---QFSS---FDILS---DEEVRQGLKTFS----NWP----------TYPQLYANGK--------LLGGLDI----VRELAESGELAN-TCPKAQSLE----------- | atgggctccgcagtgcagccaaatgaacgacgtgatggcggagctcgccaaggggcacgc acacgcaacgttcgtcaagctggaggccgaagcggttcccgagcgagccaggtggaccgc ctggacggggcctatgctccggagctcacgaagaaggtagagcggctggcagccagcggg agcccgggcgggtccgcgggaagccccgccgcggacctgaaccagcggctgaagaagctg atcaacgcagccccctgcatgctcttcatgaaggggtcccctcaggagccccgctgtggc ttcagccggcagatcgtcgccctcctctccgaacacaacatccaatttagcagcttcgac atcttatccgacgaggaggtgcggcaggggctgaagaccttctccaactggcccacctac cctcagctgtacgccaacgggaagctgctgggcgggctggacattgtgagagagctggct gagtctggagagcttgcaaacacctgccccaaggcccagagcctggagcaccgcctgaag accttgatcaaccgcagcccagtgatgctgttcatgaagggcaacaaggaggcggccaaa tgtggcttcagcaggcaaacgctgagcatcctgaacaacgccggagtggattacgacact tttgatatcctgcaggacgaggaagtccgacaggggctcaagacctactctaactggccc acctacccccagctgtacgtcaaaggcgagctggttggcggtctggacataatcaaggag ctggacgagagcggagagctggggtccacgctgaagggagagtcgtag |
| Q4S9D6 | UniRef cluster | -------------------------------------------EHRLKTLINRSPVMLFMKGNKEAAKCGFSR-QTLSILNNAGV---DYDT---FDILQ---DEEVRQGLKTYS----NWP----------TYPQLYVKGE--------LVGGLDI----IKELDESGELGS-TLKGES-------------- | atgggctccgcagtgcagccaaatgaacgacgtgatggcggagctcgccaaggggcacgc acacgcaacgttcgtcaagctggaggccgaagcggttcccgagcgagccaggtggaccgc ctggacggggcctatgctccggagctcacgaagaaggtagagcggctggcagccagcggg agcccgggcgggtccgcgggaagccccgccgcggacctgaaccagcggctgaagaagctg atcaacgcagccccctgcatgctcttcatgaaggggtcccctcaggagccccgctgtggc ttcagccggcagatcgtcgccctcctctccgaacacaacatccaatttagcagcttcgac atcttatccgacgaggaggtgcggcaggggctgaagaccttctccaactggcccacctac cctcagctgtacgccaacgggaagctgctgggcgggctggacattgtgagagagctggct gagtctggagagcttgcaaacacctgccccaaggcccagagcctggagcaccgcctgaag accttgatcaaccgcagcccagtgatgctgttcatgaagggcaacaaggaggcggccaaa tgtggcttcagcaggcaaacgctgagcatcctgaacaacgccggagtggattacgacact tttgatatcctgcaggacgaggaagtccgacaggggctcaagacctactctaactggccc acctacccccagctgtacgtcaaaggcgagctggttggcggtctggacataatcaaggag ctggacgagagcggagagctggggtccacgctgaagggagagtcgtag |
| Q5XGR5 | UniRef cluster | -------------------------------------------NSRLKKLITAAPCMLFIKGSPQEPRCGFSR-QIVDILNQHKI---QFSS---FDILS---DEEVRQGLKTFS----NWP----------TYPQFYSNGE--------LLGGLDI----VKEMVASGELDQ-MCPKAQSLE----------- | gggggcttttcctgggcgcaaggcagtatggcggctgtggtggaggccggctctcagagc cagtttgaggagctgctacagaaaagcgcaaagtctctaacagttgtgcatttttgggca ccatgggctccgcagtgcacccaaatgaacgaagtcatggccgaactggcgaaggagcag ccacaggtcatgtttgtaaagcttgaagccgaagccgttccagaagtgtcggaaaagtac gaaattacctccgtgccaacctttttgttttttaagaactctcagaagattgacagattg gatggtgcccacgctccggagctcacaaagagggttcagcgtcatgcctcaagcacctct tttccagcgacccccaacagtgctcccaaagaagacctcaatagcagactgaaaaagctg attacggctgctccgtgcatgctgttcataaagggatctccccaggagccaagatgtggg ttcagccggcagattgtggatatcctcaaccagcacaagatccagttcagcagctttgat atactatcagatgaagaagtccgtcagggcctcaaaaccttttccaattggcccacttac ccgcagttttactccaatggcgaactgctaggcggactggatatcgtaaaggaaatggtg gcgtctggagaattggaccaaatgtgccccaaagcccagagtctagaggagagactaaag gcattggtgaacaaagccccagtaatgctgtttatgaaagggaacaaagagatggcaaaa tgtggattcagcaggcagattctagaattaatgaacaacacgggtgtcggctatgaaaca tttgatattctggaagatgaagaggtgcgacaaggtttaaaaacctattccaactggccc acctaccctcagctttacgtgaaaggagaacttgtcggggggctggacatccttaaggag ttaaaagaaagcggcgaattggtttcagttctgaaaggagatcagtga |
| Q5XGR5 | UniRef cluster | -------------------------------------------EERLKALVNKAPVMLFMKGNKEMAKCGFSR-QILELMNNTGV---GYET---FDILE---DEEVRQGLKTYS----NWP----------TYPQLYVKGE--------LVGGLDI----LKELKESGELVS-VLKGDQ-------------- | gggggcttttcctgggcgcaaggcagtatggcggctgtggtggaggccggctctcagagc cagtttgaggagctgctacagaaaagcgcaaagtctctaacagttgtgcatttttgggca ccatgggctccgcagtgcacccaaatgaacgaagtcatggccgaactggcgaaggagcag ccacaggtcatgtttgtaaagcttgaagccgaagccgttccagaagtgtcggaaaagtac gaaattacctccgtgccaacctttttgttttttaagaactctcagaagattgacagattg gatggtgcccacgctccggagctcacaaagagggttcagcgtcatgcctcaagcacctct tttccagcgacccccaacagtgctcccaaagaagacctcaatagcagactgaaaaagctg attacggctgctccgtgcatgctgttcataaagggatctccccaggagccaagatgtggg ttcagccggcagattgtggatatcctcaaccagcacaagatccagttcagcagctttgat atactatcagatgaagaagtccgtcagggcctcaaaaccttttccaattggcccacttac ccgcagttttactccaatggcgaactgctaggcggactggatatcgtaaaggaaatggtg gcgtctggagaattggaccaaatgtgccccaaagcccagagtctagaggagagactaaag gcattggtgaacaaagccccagtaatgctgtttatgaaagggaacaaagagatggcaaaa tgtggattcagcaggcagattctagaattaatgaacaacacgggtgtcggctatgaaaca tttgatattctggaagatgaagaggtgcgacaaggtttaaaaacctattccaactggccc acctaccctcagctttacgtgaaaggagaacttgtcggggggctggacatccttaaggag ttaaaagaaagcggcgaattggtttcagttctgaaaggagatcagtga |
| Q2UCX0 | UniRef cluster | -------------------------------------------FARLRELVKAAPVMLFMKGTPSAPQCGFSR-QLVAILRERSV---KYGF---FNILA---DEDVRQGLKEFA----DWP----------TFPQLWVGGE--------LVGGLDI----VSLYFLFFF-FLCLVSLPWLNGLVL-------- | atgtcaaccctaattgaaatttcctccgaggcagaattcgactcccacatcaagtccctg ccttcaacaaccttatcgatcctctacttccatgccccctgggccgcgccctgcgcgcaa atgcgcaccgtgctcgccgccctcgcatcccagtaccccgccacacagcccccaacaacc tccttcattagcatcaacgccgaagaactccccgacatttctgaaacttacgaggtcacc gccgtgcccttcgtagtcctcactcgcgacggcaaaatccttgaatccatctccggcagc gacgccgtccgcgtccgtgaagccatcgaacgccacgccggctcgaaagcctcgaccggc gcacctgccacgatccccccgcccttggccgctgtgccccgcgagacgggccccacgacg gctacgcagccgccggccggagccgcgaacggggatgcgctgacgccggagcagtcgaag gaggcgctgtttgcgcgactcagagagctggtcaaggcggcgccggtgatgctgtttatg aaggggacgccgagtgcgccgcagtgtgggttcagtcggcagctcgtggcaattctgcgg gagaggagcgtcaagtatgggttctttaatatcttggccgatgaggatgtgaggcaggga ttgaaggagtttgcggattggccgacgttcccgcagttgtgggttggtggggagttggtt ggggggttggatattgtaagtctttatttcctcttttttttcttcctctgtttggtttcc ttaccgtggttgaatggacttgtgctaattggtgaacaggtcaaggacgagattgaaaat gatccggatttcttgcgtgagcactctgtcaacaaggctcccgttgcggcttga |
| Q5XJ54 | UniRef cluster | -------------------------------------------NQRLKRLINAAPCMLFMKGSPQEPRCGFSR-QIIQILKDHNV---QYSS---FDILS---DEEVRQGLKTYS----NWP----------TYPQVYVSGE--------LIGGLDI----VKELVESGELEN-TFPKTVSLE----------- | atggcgaacttcacggacgcggcatcactgcagcagtttgacgaattattaaaaaataac agcaagtccctcaccgttgtccattttcatgcgccatgggcaccgcagtgttcgcagatg aacgatgtgatggcggaattggccaaagagcacaaacacaccatgtttgttaagctggag gcagaggcagtcccggaggtttcggagaagtatgagatcacttcagttcccaccttcctc ttcttcaagggcggtgagaagattgacaggttggatggtgcgcatgcccctgagctgacc aataaggtgcaacggctggggtccggtgggggcggagctgtcggggcgggggacgtccct aaagaggatctcaaccagagactgaagaggctgatcaacgccgcaccctgcatgctcttc atgaagggatcgccccaggagcctcgctgtggttttagtcgtcaaattattcagattttg aaggaccacaatgtgcagtacagcagtttcgatatcctctcagacgaagaggtcagacag ggactcaaaacctactccaactggcccacttacccacaggtctacgtcagcggagagctc atcggcggattggacattgtaaaggagctggtcgagtctggtgaacttgagaatactttc cccaagaccgtctctctggaaaacaggctgaaatctctaatcaataaatcacccgtcatg ctcttcatgaaaggcaataaagaggctgccaaatgcggattcagccgtcagatacttgaa ataatgaacaacacgggggttgaatatgatacgtttgacatattggaagatgaagaggta agacaaggactgaagacgtactccaactggccaacgttcccacagctgtacgttaaagga gatctaatcggaggactggatattgtgaaggagctattagaaggcggtgagctggtgtct gtgttgaaaggggaaaactga |
| Q5XJ54 | UniRef cluster | -------------------------------------------ENRLKSLINKSPVMLFMKGNKEAAKCGFSR-QILEIMNNTGV---EYDT---FDILE---DEEVRQGLKTYS----NWP----------TFPQLYVKGD--------LIGGLDI----VKELLEGGELVS-VLKGEN-------------- | atggcgaacttcacggacgcggcatcactgcagcagtttgacgaattattaaaaaataac agcaagtccctcaccgttgtccattttcatgcgccatgggcaccgcagtgttcgcagatg aacgatgtgatggcggaattggccaaagagcacaaacacaccatgtttgttaagctggag gcagaggcagtcccggaggtttcggagaagtatgagatcacttcagttcccaccttcctc ttcttcaagggcggtgagaagattgacaggttggatggtgcgcatgcccctgagctgacc aataaggtgcaacggctggggtccggtgggggcggagctgtcggggcgggggacgtccct aaagaggatctcaaccagagactgaagaggctgatcaacgccgcaccctgcatgctcttc atgaagggatcgccccaggagcctcgctgtggttttagtcgtcaaattattcagattttg aaggaccacaatgtgcagtacagcagtttcgatatcctctcagacgaagaggtcagacag ggactcaaaacctactccaactggcccacttacccacaggtctacgtcagcggagagctc atcggcggattggacattgtaaaggagctggtcgagtctggtgaacttgagaatactttc cccaagaccgtctctctggaaaacaggctgaaatctctaatcaataaatcacccgtcatg ctcttcatgaaaggcaataaagaggctgccaaatgcggattcagccgtcagatacttgaa ataatgaacaacacgggggttgaatatgatacgtttgacatattggaagatgaagaggta agacaaggactgaagacgtactccaactggccaacgttcccacagctgtacgttaaagga gatctaatcggaggactggatattgtgaaggagctattagaaggcggtgagctggtgtct gtgttgaaaggggaaaactga |
| Q28ID3 | UniRef cluster | -------------------------------------------NGRLKKLINAAPCMLFMKGSPQEPRCGFSR-QIVALLNDQKV---QFSS---FDILS---DEEVRQGLKTFS----NWP----------TYPQFYVKGE--------LVGGLDI----VKEMVASGELDQ-MCPKAQSLE----------- | cagcatggcggcggtgttggaggccggctctgcgggccagtttgagcagctgatacagaa cagcgcaaagtctctcacagttgttcatttttgggcgccatgggctccacagtgcaccca aatgaacgaagtcatggctgaactggcgaaggagcagcctcaggttatgtttgtaaagct tgaagccgaagccgttccagaagtgtcggaaaagtatgaagttacctccgtgccaacctt tttgttttttaagaactctcagaagattgacagactggatggtgcccatgcgccggagct tacaaagagggttcaacgccatgcttcaagcacctcttttccagctacccccaacagtgc tcccaaagaagacctcaatggcagactgaaaaagttgattaacgctgctccatgcatgct gttcatgaaaggatctccccaggagccaagatgcgggttcagcaggcagattgtggctct ccttaacgatcaaaaggtccagttcagcagttttgatatcctgtcagacgaggaagtccg ccagggcctaaaaaccttttccaactggcccacttatccccagttttatgtcaagggtga actggtgggaggactcgatattgtgaaggaaatggtggcatcaggagaactggaccaaat gtgccccaaagcccagagtctagaggagaggctgaaggcacttgtgaacaaagccccagt catgctgtttatgaaagggaacaaagagatggcaaagtgtggattcagcaggcagattct agaaataatgaacaacacgggtgttacctacgaaacatttgatatactggaagatgaaga agtgcgtcaaggtttaaaagcctattccaattggcccacctatcctcaactttacgttaa aggagaacttgtcggggggctggacatcattaaggagttaaaagaaagtggcgaattggt ttcagttctgaaaggagatcaataa |
| A9JSB2 | UniRef cluster | -------------------------------------------NGRLKKLINAAPCMLFMKGSPQEPRCGFSR-QIVALLNDQKV---QFSS---FDILS---DEEVRQGLKTFS----NWP----------TYPQFYVKGE--------LVGGLDI----VKEMVASGELDQ-MCPKAQSLE----------- | ggaggccggctctgcgggccagtttgagcagctgatacagaacagcgcaaagtctctcac agttgttcatttttgggcgccatgggctccacagtgcacccaaatgaacgaagtcatggc tgaactggcgaaggagcagcctcaggttatgtttgtaaagcttgaagccgaagccgttcc agaagtgtcggaaaagtatgaagttacctccgtgccaacctttttgttttttaagaactc tcagaagattgacagactggatggtgcccatgcgccggagcttacaaagagggttcaacg ccatgcttcaagcacctcttttccagctacccccaacagtgctcccaaagaagacctcaa tggcagactgaaaaagttgattaacgctgctccatgcatgctgttcatgaaaggatctcc ccaggagccaagatgcgggttcagcaggcagattgtggctctccttaacgatcaaaaggt ccagttcagcagttttgatatcctgtcagacgaggaagtccgccagggcctaaaaacctt ttccaactggcccacttatccccagttttatgtcaagggtgaactggtgggaggactcga tattgtgaaggaaatggtggcatcaggagaactggaccaaatgtgccccaaagcccagag tctagaggagaggctgaaggcacttgtgaacaaagccccagtcatgctgtttatgaaagg gaacaaagagatggcaaagtgtggattcagcaggcagattctagaaataatgaacaacac gggtgttacctacgaaacatttgatatactggaagatgaagaagtgcgtcaaggtttaaa agcctattccaattggcccacctatcctcaactttacgttaaaggagaacttgtcggggg gctggacatcattaaggagttaaaagaaagtggcgaattggtttcagttctgaaaggaga tcaataa |
| Q28ID3 | UniRef cluster | -------------------------------------------EERLKALVNKAPVMLFMKGNKEMAKCGFSR-QILEIMNNTGV---TYET---FDILE---DEEVRQGLKAYS----NWP----------TYPQLYVKGE--------LVGGLDI----IKELKESGELVS-VLKGDQ-------------- | cagcatggcggcggtgttggaggccggctctgcgggccagtttgagcagctgatacagaa cagcgcaaagtctctcacagttgttcatttttgggcgccatgggctccacagtgcaccca aatgaacgaagtcatggctgaactggcgaaggagcagcctcaggttatgtttgtaaagct tgaagccgaagccgttccagaagtgtcggaaaagtatgaagttacctccgtgccaacctt tttgttttttaagaactctcagaagattgacagactggatggtgcccatgcgccggagct tacaaagagggttcaacgccatgcttcaagcacctcttttccagctacccccaacagtgc tcccaaagaagacctcaatggcagactgaaaaagttgattaacgctgctccatgcatgct gttcatgaaaggatctccccaggagccaagatgcgggttcagcaggcagattgtggctct ccttaacgatcaaaaggtccagttcagcagttttgatatcctgtcagacgaggaagtccg ccagggcctaaaaaccttttccaactggcccacttatccccagttttatgtcaagggtga actggtgggaggactcgatattgtgaaggaaatggtggcatcaggagaactggaccaaat gtgccccaaagcccagagtctagaggagaggctgaaggcacttgtgaacaaagccccagt catgctgtttatgaaagggaacaaagagatggcaaagtgtggattcagcaggcagattct agaaataatgaacaacacgggtgttacctacgaaacatttgatatactggaagatgaaga agtgcgtcaaggtttaaaagcctattccaattggcccacctatcctcaactttacgttaa aggagaacttgtcggggggctggacatcattaaggagttaaaagaaagtggcgaattggt ttcagttctgaaaggagatcaataa |
| A9JSB2 | UniRef cluster | -------------------------------------------EERLKALVNKAPVMLFMKGNKEMAKCGFSR-QILEIMNNTGV---TYET---FDILE---DEEVRQGLKAYS----NWP----------TYPQLYVKGE--------LVGGLDI----IKELKESGELVS-VLKGDQ-------------- | ggaggccggctctgcgggccagtttgagcagctgatacagaacagcgcaaagtctctcac agttgttcatttttgggcgccatgggctccacagtgcacccaaatgaacgaagtcatggc tgaactggcgaaggagcagcctcaggttatgtttgtaaagcttgaagccgaagccgttcc agaagtgtcggaaaagtatgaagttacctccgtgccaacctttttgttttttaagaactc tcagaagattgacagactggatggtgcccatgcgccggagcttacaaagagggttcaacg ccatgcttcaagcacctcttttccagctacccccaacagtgctcccaaagaagacctcaa tggcagactgaaaaagttgattaacgctgctccatgcatgctgttcatgaaaggatctcc ccaggagccaagatgcgggttcagcaggcagattgtggctctccttaacgatcaaaaggt ccagttcagcagttttgatatcctgtcagacgaggaagtccgccagggcctaaaaacctt ttccaactggcccacttatccccagttttatgtcaagggtgaactggtgggaggactcga tattgtgaaggaaatggtggcatcaggagaactggaccaaatgtgccccaaagcccagag tctagaggagaggctgaaggcacttgtgaacaaagccccagtcatgctgtttatgaaagg gaacaaagagatggcaaagtgtggattcagcaggcagattctagaaataatgaacaacac gggtgttacctacgaaacatttgatatactggaagatgaagaagtgcgtcaaggtttaaa agcctattccaattggcccacctatcctcaactttacgttaaaggagaacttgtcggggg gctggacatcattaaggagttaaaagaaagtggcgaattggtttcagttctgaaaggaga tcaataa |
| Q28ID3 | UniRef cluster | -------------------------------------------NGRLKKLINAAPCMLFMKGSPQEPRCGFSR-QIVALLNDQKV---QFSS---FDILS---DEEVRQGLKTFS----NWP----------TYPQFYVKGE--------LVGGLDI----VKEMVASGELDQ-MCPKAQSLE----------- | cagcatggcggcggtgttggaggccggctctgcgggccagtttgagcagctgatacagaa cagcgcaaagtctctcacagttgttcatttttgggcgccatgggctccacagtgcaccca aatgaacgaagtcatggctgaactggcgaaggagcagcctcaggttatgtttgtaaagct tgaagccgaagccgttccagaagtgtcggaaaagtatgaagttacctccgtgccaacctt tttgttttttaagaactctcagaagattgacagactggatggtgcccatgcgccggagct tacaaagagggttcaacgccatgcttcaagcacctcttttccagctacccccaacagtgc tcccaaagaagacctcaatggcagactgaaaaagttgattaacgctgctccatgcatgct gttcatgaaaggatctccccaggagccaagatgcgggttcagcaggcagattgtggctct ccttaacgatcaaaaggtccagttcagcagttttgatatcctgtcagacgaggaagtccg ccagggcctaaaaaccttttccaactggcccacttatccccagttttatgtcaagggtga actggtgggaggactcgatattgtgaaggaaatggtggcatcaggagaactggaccaaat gtgccccaaagcccagagtctagaggagaggctgaaggcacttgtgaacaaagccccagt catgctgtttatgaaagggaacaaagagatggcaaagtgtggattcagcaggcagattct agaaataatgaacaacacgggtgttacctacgaaacatttgatatactggaagatgaaga agtgcgtcaaggtttaaaagcctattccaattggcccacctatcctcaactttacgttaa aggagaacttgtcggggggctggacatcattaaggagttaaaagaaagtggcgaattggt ttcagttctgaaaggagatcaataa |
| A9JSB2 | UniRef cluster | -------------------------------------------NGRLKKLINAAPCMLFMKGSPQEPRCGFSR-QIVALLNDQKV---QFSS---FDILS---DEEVRQGLKTFS----NWP----------TYPQFYVKGE--------LVGGLDI----VKEMVASGELDQ-MCPKAQSLE----------- | ggaggccggctctgcgggccagtttgagcagctgatacagaacagcgcaaagtctctcac agttgttcatttttgggcgccatgggctccacagtgcacccaaatgaacgaagtcatggc tgaactggcgaaggagcagcctcaggttatgtttgtaaagcttgaagccgaagccgttcc agaagtgtcggaaaagtatgaagttacctccgtgccaacctttttgttttttaagaactc tcagaagattgacagactggatggtgcccatgcgccggagcttacaaagagggttcaacg ccatgcttcaagcacctcttttccagctacccccaacagtgctcccaaagaagacctcaa tggcagactgaaaaagttgattaacgctgctccatgcatgctgttcatgaaaggatctcc ccaggagccaagatgcgggttcagcaggcagattgtggctctccttaacgatcaaaaggt ccagttcagcagttttgatatcctgtcagacgaggaagtccgccagggcctaaaaacctt ttccaactggcccacttatccccagttttatgtcaagggtgaactggtgggaggactcga tattgtgaaggaaatggtggcatcaggagaactggaccaaatgtgccccaaagcccagag tctagaggagaggctgaaggcacttgtgaacaaagccccagtcatgctgtttatgaaagg gaacaaagagatggcaaagtgtggattcagcaggcagattctagaaataatgaacaacac gggtgttacctacgaaacatttgatatactggaagatgaagaagtgcgtcaaggtttaaa agcctattccaattggcccacctatcctcaactttacgttaaaggagaacttgtcggggg gctggacatcattaaggagttaaaagaaagtggcgaattggtttcagttctgaaaggaga tcaataa |
| Q28ID3 | UniRef cluster | -------------------------------------------EERLKALVNKAPVMLFMKGNKEMAKCGFSR-QILEIMNNTGV---TYET---FDILE---DEEVRQGLKAYS----NWP----------TYPQLYVKGE--------LVGGLDI----IKELKESGELVS-VLKGDQ-------------- | cagcatggcggcggtgttggaggccggctctgcgggccagtttgagcagctgatacagaa cagcgcaaagtctctcacagttgttcatttttgggcgccatgggctccacagtgcaccca aatgaacgaagtcatggctgaactggcgaaggagcagcctcaggttatgtttgtaaagct tgaagccgaagccgttccagaagtgtcggaaaagtatgaagttacctccgtgccaacctt tttgttttttaagaactctcagaagattgacagactggatggtgcccatgcgccggagct tacaaagagggttcaacgccatgcttcaagcacctcttttccagctacccccaacagtgc tcccaaagaagacctcaatggcagactgaaaaagttgattaacgctgctccatgcatgct gttcatgaaaggatctccccaggagccaagatgcgggttcagcaggcagattgtggctct ccttaacgatcaaaaggtccagttcagcagttttgatatcctgtcagacgaggaagtccg ccagggcctaaaaaccttttccaactggcccacttatccccagttttatgtcaagggtga actggtgggaggactcgatattgtgaaggaaatggtggcatcaggagaactggaccaaat gtgccccaaagcccagagtctagaggagaggctgaaggcacttgtgaacaaagccccagt catgctgtttatgaaagggaacaaagagatggcaaagtgtggattcagcaggcagattct agaaataatgaacaacacgggtgttacctacgaaacatttgatatactggaagatgaaga agtgcgtcaaggtttaaaagcctattccaattggcccacctatcctcaactttacgttaa aggagaacttgtcggggggctggacatcattaaggagttaaaagaaagtggcgaattggt ttcagttctgaaaggagatcaataa |
| A9JSB2 | UniRef cluster | -------------------------------------------EERLKALVNKAPVMLFMKGNKEMAKCGFSR-QILEIMNNTGV---TYET---FDILE---DEEVRQGLKAYS----NWP----------TYPQLYVKGE--------LVGGLDI----IKELKESGELVS-VLKGDQ-------------- | ggaggccggctctgcgggccagtttgagcagctgatacagaacagcgcaaagtctctcac agttgttcatttttgggcgccatgggctccacagtgcacccaaatgaacgaagtcatggc tgaactggcgaaggagcagcctcaggttatgtttgtaaagcttgaagccgaagccgttcc agaagtgtcggaaaagtatgaagttacctccgtgccaacctttttgttttttaagaactc tcagaagattgacagactggatggtgcccatgcgccggagcttacaaagagggttcaacg ccatgcttcaagcacctcttttccagctacccccaacagtgctcccaaagaagacctcaa tggcagactgaaaaagttgattaacgctgctccatgcatgctgttcatgaaaggatctcc ccaggagccaagatgcgggttcagcaggcagattgtggctctccttaacgatcaaaaggt ccagttcagcagttttgatatcctgtcagacgaggaagtccgccagggcctaaaaacctt ttccaactggcccacttatccccagttttatgtcaagggtgaactggtgggaggactcga tattgtgaaggaaatggtggcatcaggagaactggaccaaatgtgccccaaagcccagag tctagaggagaggctgaaggcacttgtgaacaaagccccagtcatgctgtttatgaaagg gaacaaagagatggcaaagtgtggattcagcaggcagattctagaaataatgaacaacac gggtgttacctacgaaacatttgatatactggaagatgaagaagtgcgtcaaggtttaaa agcctattccaattggcccacctatcctcaactttacgttaaaggagaacttgtcggggg gctggacatcattaaggagttaaaagaaagtggcgaattggtttcagttctgaaaggaga tcaataa |
| A7RQK5 | UniRef cluster | -------------------------------------------TSRLKKLVNSSPCMLFMKGTPQEPKCGFSR-QVVGILAGVGA---QYST---FDILK---DEEVRQGLKKYS----DWP----------TYPQLYINGE--------LVGGLDI----IKELATSGELAS-MLPPKQDLK----------- | ttggctgtagtgcacttttttgcaccatgggccccacattgtaaccaaatgaatgatgta cttgaagagcttgccaaggaaaatcctcatgtaaactttatcaaggttgaagctgaaaaa ctacctgaagtctcgtataaaaataatataaatgctgtaccaactttattactttttaag aatcagaaggttgttgatcggattgatggcgcaaatgcacctgagctaaccaaaaaagtc gagcaccatgccagcataatattaccaccagaaccatgtgaacagcccgagactcaggac ttgacttcaagactcaagaagctggtcaatagctcaccttgtatgttattcatgaaaggc acccctcaggaacccaaatgtggtttcagtcgtcaagttgttggaattctggctggagtt ggtgcacagtatagcacatttgacatcctaaaggatgaggaagtcagacagggtctgaaa aaatactctgactggcctacatatcctcagctgtacataaatggtgagctagttggtgga ttggacatcattaaggaattggctaccagcggtgagctggcatctatgttgccgccaaaa caggatctaaaaacaaggtgtgtcaatctactaaaatctgtgaatgttttgctgtttatg aaaggaagtcctgagaatcctcgatgtgggtttagtcgacaaatttgtgagattctttcg cactacagtcaaagctacaagtcttttgatatccttgaggatattgaggttcgagaaggt ctcaagaagtactctaattggccgacctaccctcagttgtacgtcaagggggagcttatc ggaggactggacatagttagggaactccatgggaatggcgagttggaagatgctctaaaa agctcccagtaa |
| A7RQK5 | UniRef cluster | -------------------------------------------KTRCVNLLKSVNVLLFMKGSPENPRCGFSR-QICEILSHYSQ---SYKS---FDILE---DIEVREGLKKYS----NWP----------TYPQLYVKGE--------LIGGLDI----VRELHGNGELEDALKSSQ--------------- | ttggctgtagtgcacttttttgcaccatgggccccacattgtaaccaaatgaatgatgta cttgaagagcttgccaaggaaaatcctcatgtaaactttatcaaggttgaagctgaaaaa ctacctgaagtctcgtataaaaataatataaatgctgtaccaactttattactttttaag aatcagaaggttgttgatcggattgatggcgcaaatgcacctgagctaaccaaaaaagtc gagcaccatgccagcataatattaccaccagaaccatgtgaacagcccgagactcaggac ttgacttcaagactcaagaagctggtcaatagctcaccttgtatgttattcatgaaaggc acccctcaggaacccaaatgtggtttcagtcgtcaagttgttggaattctggctggagtt ggtgcacagtatagcacatttgacatcctaaaggatgaggaagtcagacagggtctgaaa aaatactctgactggcctacatatcctcagctgtacataaatggtgagctagttggtgga ttggacatcattaaggaattggctaccagcggtgagctggcatctatgttgccgccaaaa caggatctaaaaacaaggtgtgtcaatctactaaaatctgtgaatgttttgctgtttatg aaaggaagtcctgagaatcctcgatgtgggtttagtcgacaaatttgtgagattctttcg cactacagtcaaagctacaagtcttttgatatccttgaggatattgaggttcgagaaggt ctcaagaagtactctaattggccgacctaccctcagttgtacgtcaagggggagcttatc ggaggactggacatagttagggaactccatgggaatggcgagttggaagatgctctaaaa agctcccagtaa |
| GLRX3 | UniRef cluster | -------------------------------------------NLRLKKLTHAAPCMLFMKGTPQEPRCGFSK-QMVEILHKHNI---QFSS---FDIFS---DEEVRQGLKTYS----NWP----------TYPQLYVSGE--------LIGGLDI----IKELEASEELDT-ICPKAPKLE----------- |  |
| GLRX3 | UniRef cluster | -------------------------------------------EERLKVLTNKASVMLFMKGNKQEAKCGFSK-QILEILNSTGV---EYET---FDILE---DEEVRQGLKTFS----NWP----------TYPQLYVRGD--------LVGGLDI----VKELKDNGELLP-ILKGEN-------------- |  |
| GLRX3 | UniRef cluster | -------------------------------------------EERLKVLTNKASVMLFMKGNKQEAKCGFSK-QILEILNSTGV---EYET---FDILE---DEEVRQGLKTFS----NWP----------TYPQLYVRGD--------LVGGLDI----VKELKDNGELLP-ILKGEN-------------- |  |
| Q5I0V8 | UniRef cluster | -------------------------------------------EERLKVLTNKASVMLFMKGNKQEAKCGFSK-QILEILNSTGV---EYET---FDILE---DEEVRQGLKTFS----NWP----------TYPQLYVRGD--------LVGGLDI----VKELKDNGELLP-ILKGEN-------------- | atggcggcgggggcggccgaggcaggcgaggcagccgtggcagtggtggaagtcggctct gcccagcagtttgaagagctactgcgcctcaaaaccaagtcactccttgtggttcatttc tgggcaccatgggctccacagtgtgtacagatgaacgatgtcatggcagagttagctaaa gaacaccctcatgtttcatttgtgaagctggaagccgaagctgttcctgaagtatctgaa aaatatgaaattagctctgtccccaccttcctgttcttcaagaattctcagaaagtcgac cggttagatggtgcacatgccccagagttgaccaaaaaagtccagcgacacgtgtctagc ggagcctttcctcctagtactaatgaacatcttaaagaagacctcagccttcgcctgaaa aagctgactcacgctgccccctgcatgctgttcatgaagggaacacctcaagaaccacgc tgtggtttcagcaagcagatggtggaaatccttcacaaacacaatattcagttcagcagc tttgatatcttctcagatgaagaagttcgacaggggctcaaaacgtactctaattggccc acctatcctcagctctatgtttctggagagctaataggaggacttgacataattaaggag ctggaagcatcagaagagctggacacgatctgtcccaaagcacccaagttagaggaaagg ctcaaagtgctgaccaataaagcttccgtgatgctctttatgaaaggaaacaaacaggaa gcaaaatgtggattcagcaaacaaattctggaaatactgaatagtactggggttgaatat gaaacttttgatatactggaagatgaagaagtgcgtcagggattaaaaacgttctcaaat tggccaacctaccctcagctgtatgtgagaggggatcttgttggaggattggatattgtc aaggaactgaaagacaacggtgaattgctgcctatactgaaaggagaaaattag |
| Q9JLZ1-2 | UniRef cluster | -------------------------------------------EERLKVLTNKASVMLFMKGNKQEAKCGFSK-QILEILNSTGV---EYET---FDILE---DEEVRQGLKTFS----NWP----------TYPQLYVRGD--------LVGGLDI----VKELKDNGELLP-ILKGEN-------------- |  |
| GLRX3 | UniRef cluster | -------------------------------------------NLRLKKLTHAAPCMLFMKGTPQEPRCGFSK-QMVEILHKHNI---QFSS---FDIFS---DEEVRQGLKAYS----SWP----------TYPQLYVSGE--------LIGGLDI----IKELEASEELDT-ICPKAPKLE----------- |  |
| Q5JV01 | UniRef cluster | -------------------------------------------NLRLKKLTHAAPCMLFMKGTPQEPRCGFSK-QMVEILHKHNI---QFSS---FDIFS---DEEVRQGLKAYS----SWP----------TYPQLYVSGE--------LIGGLDI----IKELEASEELDT-ICPKAPKLE----------- | atggcggcgggggcggctgaggcagctgtagcggccgtggaggaggtcggctcagccggg cagtttgaggagctgctgcgcctcaaagccaagtccctccttgtggtccatttctgggca ccatgggctccacagtgtgcacagatgaacgaagttatggcagagttagctaaagaactc cctcaagtttcatttgtgaagttggaagctgaaggtgttcctgaagtatctgaaaaatat gaaattagctctgttcccacttttctgtttttcaagaattctcagaaaatcgaccgatta gatggtgcacatgccccagagttgaccaaaaaagttcagcgacatgcatctagtggctcc ttcctacccagcgctaatgaacatcttaaagaagatctcaaccttcgcttgaagaaattg actcatgctgccccctgcatgctgtttatgaaaggaactcctcaagaaccacgctgtggt ttcagcaagcagatggtggaaattcttcacaaacataatattcagtttagcagttttgat atcttctcagatgaagaggttcgacagggactcaaagcctattccagttggcctacctat cctcagctctatgtttctggagagctcataggaggacttgatataattaaggagctagaa gcatctgaagaactagatacaatttgtcccaaagctcccaaattagaggaaaggctcaaa gtgctgacaaataaagcttctgtgatgctctttatgaaaggaaacaaacaggaagcaaaa tgtggattcagcaaacaaattctggaaatactaaatagtactggtgttgaatatgaaaca ttcgatatattggaggatgaagaagttcggcaaggattaaaagcttactcaaattggcca acataccctcagctgtatgtgaaaggggagctggtgggaggattggatattgtgaaggaa ctgaaagaaaatggtgaattgctgcctatactgagaggagaaaattaa |
| GLRX3 | UniRef cluster | -------------------------------------------EERLKVLTNKASVMLFMKGNKQEAKCGFSK-QILEILNSTGV---EYET---FDILE---DEEVRQGLKAYS----NWP----------TYPQLYVKGE--------LVGGLDI----VKELKENGELLP-ILRGEN-------------- |  |
| Q5JV01 | UniRef cluster | -------------------------------------------EERLKVLTNKASVMLFMKGNKQEAKCGFSK-QILEILNSTGV---EYET---FDILE---DEEVRQGLKAYS----NWP----------TYPQLYVKGE--------LVGGLDI----VKELKENGELLP-ILRGEN-------------- | atggcggcgggggcggctgaggcagctgtagcggccgtggaggaggtcggctcagccggg cagtttgaggagctgctgcgcctcaaagccaagtccctccttgtggtccatttctgggca ccatgggctccacagtgtgcacagatgaacgaagttatggcagagttagctaaagaactc cctcaagtttcatttgtgaagttggaagctgaaggtgttcctgaagtatctgaaaaatat gaaattagctctgttcccacttttctgtttttcaagaattctcagaaaatcgaccgatta gatggtgcacatgccccagagttgaccaaaaaagttcagcgacatgcatctagtggctcc ttcctacccagcgctaatgaacatcttaaagaagatctcaaccttcgcttgaagaaattg actcatgctgccccctgcatgctgtttatgaaaggaactcctcaagaaccacgctgtggt ttcagcaagcagatggtggaaattcttcacaaacataatattcagtttagcagttttgat atcttctcagatgaagaggttcgacagggactcaaagcctattccagttggcctacctat cctcagctctatgtttctggagagctcataggaggacttgatataattaaggagctagaa gcatctgaagaactagatacaatttgtcccaaagctcccaaattagaggaaaggctcaaa gtgctgacaaataaagcttctgtgatgctctttatgaaaggaaacaaacaggaagcaaaa tgtggattcagcaaacaaattctggaaatactaaatagtactggtgttgaatatgaaaca ttcgatatattggaggatgaagaagttcggcaaggattaaaagcttactcaaattggcca acataccctcagctgtatgtgaaaggggagctggtgggaggattggatattgtgaaggaa ctgaaagaaaatggtgaattgctgcctatactgagaggagaaaattaa |
| GLRX3 | UniRef cluster | -------------------------------------------NLRLKKLTHAAPCMLFMKGTPQEPRCGFSK-QMVEILHKHNI---QFSS---FDIFS---DEEVRQGLKAYS----SWP----------TYPQLYVSGE--------LIGGLDI----IKELEASEELDT-ICPKAPKLE----------- |  |
| Q5JV01 | UniRef cluster | -------------------------------------------NLRLKKLTHAAPCMLFMKGTPQEPRCGFSK-QMVEILHKHNI---QFSS---FDIFS---DEEVRQGLKAYS----SWP----------TYPQLYVSGE--------LIGGLDI----IKELEASEELDT-ICPKAPKLE----------- | atggcggcgggggcggctgaggcagctgtagcggccgtggaggaggtcggctcagccggg cagtttgaggagctgctgcgcctcaaagccaagtccctccttgtggtccatttctgggca ccatgggctccacagtgtgcacagatgaacgaagttatggcagagttagctaaagaactc cctcaagtttcatttgtgaagttggaagctgaaggtgttcctgaagtatctgaaaaatat gaaattagctctgttcccacttttctgtttttcaagaattctcagaaaatcgaccgatta gatggtgcacatgccccagagttgaccaaaaaagttcagcgacatgcatctagtggctcc ttcctacccagcgctaatgaacatcttaaagaagatctcaaccttcgcttgaagaaattg actcatgctgccccctgcatgctgtttatgaaaggaactcctcaagaaccacgctgtggt ttcagcaagcagatggtggaaattcttcacaaacataatattcagtttagcagttttgat atcttctcagatgaagaggttcgacagggactcaaagcctattccagttggcctacctat cctcagctctatgtttctggagagctcataggaggacttgatataattaaggagctagaa gcatctgaagaactagatacaatttgtcccaaagctcccaaattagaggaaaggctcaaa gtgctgacaaataaagcttctgtgatgctctttatgaaaggaaacaaacaggaagcaaaa tgtggattcagcaaacaaattctggaaatactaaatagtactggtgttgaatatgaaaca ttcgatatattggaggatgaagaagttcggcaaggattaaaagcttactcaaattggcca acataccctcagctgtatgtgaaaggggagctggtgggaggattggatattgtgaaggaa ctgaaagaaaatggtgaattgctgcctatactgagaggagaaaattaa |
| GLRX3 | UniRef cluster | -------------------------------------------SLRLKKLTHAAPCMLFMKGTPQEPRCGFSK-QMVEILHKHNI---QFSS---FDIFS---DEEVRQGLKTYS----NWP----------TYPQLYVSGE--------LIGGLDI----IKELEASEELDT-ICPKAPKLE----------- |  |
| Q5I0V8 | UniRef cluster | -------------------------------------------SLRLKKLTHAAPCMLFMKGTPQEPRCGFSK-QMVEILHKHNI---QFSS---FDIFS---DEEVRQGLKTYS----NWP----------TYPQLYVSGE--------LIGGLDI----IKELEASEELDT-ICPKAPKLE----------- | atggcggcgggggcggccgaggcaggcgaggcagccgtggcagtggtggaagtcggctct gcccagcagtttgaagagctactgcgcctcaaaaccaagtcactccttgtggttcatttc tgggcaccatgggctccacagtgtgtacagatgaacgatgtcatggcagagttagctaaa gaacaccctcatgtttcatttgtgaagctggaagccgaagctgttcctgaagtatctgaa aaatatgaaattagctctgtccccaccttcctgttcttcaagaattctcagaaagtcgac cggttagatggtgcacatgccccagagttgaccaaaaaagtccagcgacacgtgtctagc ggagcctttcctcctagtactaatgaacatcttaaagaagacctcagccttcgcctgaaa aagctgactcacgctgccccctgcatgctgttcatgaagggaacacctcaagaaccacgc tgtggtttcagcaagcagatggtggaaatccttcacaaacacaatattcagttcagcagc tttgatatcttctcagatgaagaagttcgacaggggctcaaaacgtactctaattggccc acctatcctcagctctatgtttctggagagctaataggaggacttgacataattaaggag ctggaagcatcagaagagctggacacgatctgtcccaaagcacccaagttagaggaaagg ctcaaagtgctgaccaataaagcttccgtgatgctctttatgaaaggaaacaaacaggaa gcaaaatgtggattcagcaaacaaattctggaaatactgaatagtactggggttgaatat gaaacttttgatatactggaagatgaagaagtgcgtcagggattaaaaacgttctcaaat tggccaacctaccctcagctgtatgtgagaggggatcttgttggaggattggatattgtc aaggaactgaaagacaacggtgaattgctgcctatactgaaaggagaaaattag |
| GLRX3 | UniRef cluster | -------------------------------------------EERLKVLTNKASVMLFMKGNKQEAKCGFSK-QILEILNSTGV---EYET---FDILE---DEEVRQGLKTFS----NWP----------TYPQLYVRGD--------LVGGLDI----VKELKDNGELLP-ILKGEN-------------- |  |
| GLRX3 | UniRef cluster | -------------------------------------------EERLKVLTNKASVMLFMKGNKQEAKCGFSK-QILEILNSTGV---EYET---FDILE---DEEVRQGLKTFS----NWP----------TYPQLYVRGD--------LVGGLDI----VKELKDNGELLP-ILKGEN-------------- |  |
| Q5I0V8 | UniRef cluster | -------------------------------------------EERLKVLTNKASVMLFMKGNKQEAKCGFSK-QILEILNSTGV---EYET---FDILE---DEEVRQGLKTFS----NWP----------TYPQLYVRGD--------LVGGLDI----VKELKDNGELLP-ILKGEN-------------- | atggcggcgggggcggccgaggcaggcgaggcagccgtggcagtggtggaagtcggctct gcccagcagtttgaagagctactgcgcctcaaaaccaagtcactccttgtggttcatttc tgggcaccatgggctccacagtgtgtacagatgaacgatgtcatggcagagttagctaaa gaacaccctcatgtttcatttgtgaagctggaagccgaagctgttcctgaagtatctgaa aaatatgaaattagctctgtccccaccttcctgttcttcaagaattctcagaaagtcgac cggttagatggtgcacatgccccagagttgaccaaaaaagtccagcgacacgtgtctagc ggagcctttcctcctagtactaatgaacatcttaaagaagacctcagccttcgcctgaaa aagctgactcacgctgccccctgcatgctgttcatgaagggaacacctcaagaaccacgc tgtggtttcagcaagcagatggtggaaatccttcacaaacacaatattcagttcagcagc tttgatatcttctcagatgaagaagttcgacaggggctcaaaacgtactctaattggccc acctatcctcagctctatgtttctggagagctaataggaggacttgacataattaaggag ctggaagcatcagaagagctggacacgatctgtcccaaagcacccaagttagaggaaagg ctcaaagtgctgaccaataaagcttccgtgatgctctttatgaaaggaaacaaacaggaa gcaaaatgtggattcagcaaacaaattctggaaatactgaatagtactggggttgaatat gaaacttttgatatactggaagatgaagaagtgcgtcagggattaaaaacgttctcaaat tggccaacctaccctcagctgtatgtgagaggggatcttgttggaggattggatattgtc aaggaactgaaagacaacggtgaattgctgcctatactgaaaggagaaaattag |
| Q9JLZ1-2 | UniRef cluster | -------------------------------------------EERLKVLTNKASVMLFMKGNKQEAKCGFSK-QILEILNSTGV---EYET---FDILE---DEEVRQGLKTFS----NWP----------TYPQLYVRGD--------LVGGLDI----VKELKDNGELLP-ILKGEN-------------- |  |
| GLRX3 | UniRef cluster | -------------------------------------------SLRLKKLTHAAPCMLFMKGTPQEPRCGFSK-QMVEILHKHNI---QFSS---FDIFS---DEEVRQGLKTYS----NWP----------TYPQLYVSGE--------LIGGLDI----IKELEASEELDT-ICPKAPKLE----------- |  |
| Q5I0V8 | UniRef cluster | -------------------------------------------SLRLKKLTHAAPCMLFMKGTPQEPRCGFSK-QMVEILHKHNI---QFSS---FDIFS---DEEVRQGLKTYS----NWP----------TYPQLYVSGE--------LIGGLDI----IKELEASEELDT-ICPKAPKLE----------- | atggcggcgggggcggccgaggcaggcgaggcagccgtggcagtggtggaagtcggctct gcccagcagtttgaagagctactgcgcctcaaaaccaagtcactccttgtggttcatttc tgggcaccatgggctccacagtgtgtacagatgaacgatgtcatggcagagttagctaaa gaacaccctcatgtttcatttgtgaagctggaagccgaagctgttcctgaagtatctgaa aaatatgaaattagctctgtccccaccttcctgttcttcaagaattctcagaaagtcgac cggttagatggtgcacatgccccagagttgaccaaaaaagtccagcgacacgtgtctagc ggagcctttcctcctagtactaatgaacatcttaaagaagacctcagccttcgcctgaaa aagctgactcacgctgccccctgcatgctgttcatgaagggaacacctcaagaaccacgc tgtggtttcagcaagcagatggtggaaatccttcacaaacacaatattcagttcagcagc tttgatatcttctcagatgaagaagttcgacaggggctcaaaacgtactctaattggccc acctatcctcagctctatgtttctggagagctaataggaggacttgacataattaaggag ctggaagcatcagaagagctggacacgatctgtcccaaagcacccaagttagaggaaagg ctcaaagtgctgaccaataaagcttccgtgatgctctttatgaaaggaaacaaacaggaa gcaaaatgtggattcagcaaacaaattctggaaatactgaatagtactggggttgaatat gaaacttttgatatactggaagatgaagaagtgcgtcagggattaaaaacgttctcaaat tggccaacctaccctcagctgtatgtgagaggggatcttgttggaggattggatattgtc aaggaactgaaagacaacggtgaattgctgcctatactgaaaggagaaaattag |
| GLRX3 | UniRef cluster | -------------------------------------------EERLKVLTNKASVMLFMKGNKQEAKCGFSK-QILEILNSTGV---EYET---FDILE---DEEVRQGLKTFS----NWP----------TYPQLYVRGD--------LVGGLDI----VKELKDNGELLP-ILKGEN-------------- |  |
| GLRX3 | UniRef cluster | -------------------------------------------EERLKVLTNKASVMLFMKGNKQEAKCGFSK-QILEILNSTGV---EYET---FDILE---DEEVRQGLKTFS----NWP----------TYPQLYVRGD--------LVGGLDI----VKELKDNGELLP-ILKGEN-------------- |  |
| Q5I0V8 | UniRef cluster | -------------------------------------------EERLKVLTNKASVMLFMKGNKQEAKCGFSK-QILEILNSTGV---EYET---FDILE---DEEVRQGLKTFS----NWP----------TYPQLYVRGD--------LVGGLDI----VKELKDNGELLP-ILKGEN-------------- | atggcggcgggggcggccgaggcaggcgaggcagccgtggcagtggtggaagtcggctct gcccagcagtttgaagagctactgcgcctcaaaaccaagtcactccttgtggttcatttc tgggcaccatgggctccacagtgtgtacagatgaacgatgtcatggcagagttagctaaa gaacaccctcatgtttcatttgtgaagctggaagccgaagctgttcctgaagtatctgaa aaatatgaaattagctctgtccccaccttcctgttcttcaagaattctcagaaagtcgac cggttagatggtgcacatgccccagagttgaccaaaaaagtccagcgacacgtgtctagc ggagcctttcctcctagtactaatgaacatcttaaagaagacctcagccttcgcctgaaa aagctgactcacgctgccccctgcatgctgttcatgaagggaacacctcaagaaccacgc tgtggtttcagcaagcagatggtggaaatccttcacaaacacaatattcagttcagcagc tttgatatcttctcagatgaagaagttcgacaggggctcaaaacgtactctaattggccc acctatcctcagctctatgtttctggagagctaataggaggacttgacataattaaggag ctggaagcatcagaagagctggacacgatctgtcccaaagcacccaagttagaggaaagg ctcaaagtgctgaccaataaagcttccgtgatgctctttatgaaaggaaacaaacaggaa gcaaaatgtggattcagcaaacaaattctggaaatactgaatagtactggggttgaatat gaaacttttgatatactggaagatgaagaagtgcgtcagggattaaaaacgttctcaaat tggccaacctaccctcagctgtatgtgagaggggatcttgttggaggattggatattgtc aaggaactgaaagacaacggtgaattgctgcctatactgaaaggagaaaattag |
| Q9JLZ1-2 | UniRef cluster | -------------------------------------------EERLKVLTNKASVMLFMKGNKQEAKCGFSK-QILEILNSTGV---EYET---FDILE---DEEVRQGLKTFS----NWP----------TYPQLYVRGD--------LVGGLDI----VKELKDNGELLP-ILKGEN-------------- |  |
| GLRX3 | UniRef cluster | -------------------------------------------SLRLKKLTHAAPCMLFMKGTPQEPRCGFSK-QMVEILNKHNI---QFSS---FDIFS---DEEVRQGLKTYS----SWP----------TYPQLYVSGE--------LIGGLDI----IKELEASKELDT-ICPKAPKLE----------- |  |
| GLRX3 | UniRef cluster | -------------------------------------------EERLKVLTNKASVMLFMKGNKQEAKCGFSR-QILEILNSTGI---EYET---FDILE---DEEVRQGLKAYS----NWP----------TYPQLYVKGE--------LVGGLDI----VKELKENGELLP-ILKGEN-------------- |  |
| Q4P6P0 | UniRef cluster | -------------------------------------------FARCKKLMEQSKVMLFMKGDPDTPRCGFSQ-KTVNLLRQEKV---DFGH---YDILK---DENVRQGLKKLN----EWP----------TFPQIIVNGE--------LIGGLDI----LKESIESGE--FQEMLHA--------------- | atgtctgccgaagcaggacccagcaacctcgtcgaggtcacctcgcccgagcactttacc gagatcatgcagaaggacttgactcggatctcgctgctcaactttcatgctccgtgggcc gagccatgcaaacagatgaacgaagtggtccgcgagatcgccatcaagtatcctcaagtg ctgtgtctcgaaatcgaggcagagtctcttccggatgtctctgaatccttcgacatcgag gcagtaccctcgtttgtattgcttcgtggtcacacgctcctctctcgcatcagcggtgcc aacgcttctgccctctcggcagctgtcgcaacacacgctgcttccgctcgatccaacggc actggcagtgtctccaagacctcggccgccccacgtgctgcgtcagacgtctacgacgct tcaagttccaaacgtaacggcgcttccacctacgctgacgaggaagacgaggaaaatgtt gtgcccgagacagaagaggaaatctttgcgaggtgcaaaaagctcatggaacagagcaag gtgatgcttttcatgaagggcgacccggacacaccgcgttgcggtttcagccagaaaacg gtcaaccttttgcgacaagaaaaggttgatttcggccactacgacattctcaaggacgaa aatgtacgacagggtcttaagaagctcaatgaatggcctactttcccacagatcattgtc aatggcgagcttatcggtggtcttgacatcctcaaggagagcatcgagagcggagagttc caagagatgctccatgcttga |
| Q95X14 | UniRef cluster | -------------------------------------------NARLGALVNSQKVMVFMKGDPSAPRCGFSR-TIVELLNSHKI---KFGS---FDIFS---DEAVRQGLKEYS----NWP----------TYPQLYFDGE--------LIGGLDV----VKEEFSDPQFIK-QLPKVGENS----------- | atgccaattcaagaaatcaagtcaggagaggaggttgctgcgttcattaaggaaccatct ccagctgttcttcatttctacgcatcgtgggctccaagctgtgagcaagttaatcagcta ctggatgatttgctcgctgaaattgctctgccgcttcgtgccgccttcattgacgctgaa gctttgcccggaatttcgttgaatttcaagattactgctgcgccgacgcttgtatttttt agtaatggcaaagaagtcgacagaatcgatggcttcgtgccaaaagagatccaatcaaaa gttgttcttgttgccagccgatccctttcccagagctcatcggatgcttcatctacaacc tcttccacaccttcactgactccacaacaagaaaaggaagctctaaatgccagattgggt gcgctggtcaattctcaaaaagtcatggttttcatgaaaggagatccatcggcgccacgt tgtggtttctcgagaactattgtcgaacttttaaattcacataaaatcaaattcggatcc ttcgacattttctcggatgaagcagttcgccaaggattaaaggaatactcgaattggcca acttatcctcaactctatttcgatggagaattgattggagggcttgatgtcgtcaaggag gagttttcagatccacaatttatcaagcaacttccgaaagttggagaaaatagtgagggg ggatcacttgaagatcgactcaaaaagcttgtctcatcgcaaagattgatgctatttatg aaaggagatcgggagacaccaaagtgcggattttcacgaactatcgttgatttgttgaac aaggctcgtgctgattatcatacttttgatatcttggaggatgaggaagtcaggcaaggg cttaagaaattctccaattggccaacttatccacagctctatctcgacggagagctcgtc ggagggcttgatgtcgtcaaggaagagctcttggatacgcatttccttcgtcagattcca agaattagaaatgattaa |
| Q6EZG4 | UniRef cluster | -------------------------------------------NARLGALVNSQKVMVFMKGDPSAPRCGFSR-TIVELLNSHKI---KFGS---FDIFS---DEAVRQGLKEYS----NWP----------TYPQLYFDGE--------LIGGLDV----VKEEFSDPQFIK-QLPKVGENS----------- | atgccaattcaagaaatcaagtcaggagaggaggttgctgcgttcattaaggaaccatct ccagctgttcttcatttctacgcatcgtgggctccaagctgtgagcaagttaatcagcta ctggatgatttgctcgctgaaattgctctgccgcttcgtgccgccttcattgacgctgaa gctttgcccggaatttcgttgaatttcaagattactgctgcgccgacgcttgtatttttt agtaatggcaaagaagtcgacagaatcgatggcttcgtgccaaaagagatccaatcaaaa gttgttcttgttgccagccgatccctttcccagagctcatcggatgcttcatctacaacc tcttccacaccttcactgactccacaacaagaaaaggaagctctaaatgccagattgggt gcgctggtcaattctcaaaaagtcatggttttcatgaaaggagatccatcggcgccacgt tgtggtttctcgagaactattgtcgaacttttaaattcacataaaatcaaattcggatcc ttcgacattttctcggatgaagcagttcgccaaggattaaaggaatactcgaattggcca acttatcctcaactctatttcgatggagaattgattggagggcttgatgtcgtcaaggag gagttttcagatccacaatttatcaagcaacttccgaaagttggagaaaatagtgagggg ggatcacttgaagatcgactcaaaaagcttgtctcatcgcaaagattgatgctatttatg aaaggagatcgggagacaccaaagtgcggattttcacgaactatcgttgatttgttgaac aaggctcgtgctgattatcatacttttgatatcttggaggatgaggaagtcagaaattct ccaattggccaacttatccacagctctatctcgacggagagctcgtcggagggcttgatg tcgtcaaggaagagctcttggatacgcatttccttcgtcagattccaagaattagaaatg attaattaa |
| Q95X14 | UniRef cluster | -------------------------------------------NARLGALVNSQKVMVFMKGDPSAPRCGFSR-TIVELLNSHKI---KFGS---FDIFS---DEAVRQGLKEYS----NWP----------TYPQLYFDGE--------LIGGLDV----VKEEFSDPQFIK-QLPKVGENS----------- | atgccaattcaagaaatcaagtcaggagaggaggttgctgcgttcattaaggaaccatct ccagctgttcttcatttctacgcatcgtgggctccaagctgtgagcaagttaatcagcta ctggatgatttgctcgctgaaattgctctgccgcttcgtgccgccttcattgacgctgaa gctttgcccggaatttcgttgaatttcaagattactgctgcgccgacgcttgtatttttt agtaatggcaaagaagtcgacagaatcgatggcttcgtgccaaaagagatccaatcaaaa gttgttcttgttgccagccgatccctttcccagagctcatcggatgcttcatctacaacc tcttccacaccttcactgactccacaacaagaaaaggaagctctaaatgccagattgggt gcgctggtcaattctcaaaaagtcatggttttcatgaaaggagatccatcggcgccacgt tgtggtttctcgagaactattgtcgaacttttaaattcacataaaatcaaattcggatcc ttcgacattttctcggatgaagcagttcgccaaggattaaaggaatactcgaattggcca acttatcctcaactctatttcgatggagaattgattggagggcttgatgtcgtcaaggag gagttttcagatccacaatttatcaagcaacttccgaaagttggagaaaatagtgagggg ggatcacttgaagatcgactcaaaaagcttgtctcatcgcaaagattgatgctatttatg aaaggagatcgggagacaccaaagtgcggattttcacgaactatcgttgatttgttgaac aaggctcgtgctgattatcatacttttgatatcttggaggatgaggaagtcaggcaaggg cttaagaaattctccaattggccaacttatccacagctctatctcgacggagagctcgtc ggagggcttgatgtcgtcaaggaagagctcttggatacgcatttccttcgtcagattcca agaattagaaatgattaa |
| Q6EZG4 | UniRef cluster | -------------------------------------------NARLGALVNSQKVMVFMKGDPSAPRCGFSR-TIVELLNSHKI---KFGS---FDIFS---DEAVRQGLKEYS----NWP----------TYPQLYFDGE--------LIGGLDV----VKEEFSDPQFIK-QLPKVGENS----------- | atgccaattcaagaaatcaagtcaggagaggaggttgctgcgttcattaaggaaccatct ccagctgttcttcatttctacgcatcgtgggctccaagctgtgagcaagttaatcagcta ctggatgatttgctcgctgaaattgctctgccgcttcgtgccgccttcattgacgctgaa gctttgcccggaatttcgttgaatttcaagattactgctgcgccgacgcttgtatttttt agtaatggcaaagaagtcgacagaatcgatggcttcgtgccaaaagagatccaatcaaaa gttgttcttgttgccagccgatccctttcccagagctcatcggatgcttcatctacaacc tcttccacaccttcactgactccacaacaagaaaaggaagctctaaatgccagattgggt gcgctggtcaattctcaaaaagtcatggttttcatgaaaggagatccatcggcgccacgt tgtggtttctcgagaactattgtcgaacttttaaattcacataaaatcaaattcggatcc ttcgacattttctcggatgaagcagttcgccaaggattaaaggaatactcgaattggcca acttatcctcaactctatttcgatggagaattgattggagggcttgatgtcgtcaaggag gagttttcagatccacaatttatcaagcaacttccgaaagttggagaaaatagtgagggg ggatcacttgaagatcgactcaaaaagcttgtctcatcgcaaagattgatgctatttatg aaaggagatcgggagacaccaaagtgcggattttcacgaactatcgttgatttgttgaac aaggctcgtgctgattatcatacttttgatatcttggaggatgaggaagtcagaaattct ccaattggccaacttatccacagctctatctcgacggagagctcgtcggagggcttgatg tcgtcaaggaagagctcttggatacgcatttccttcgtcagattccaagaattagaaatg attaattaa |
| Q95X14 | UniRef cluster | -------------------------------------------EDRLKKLVSSQRLMLFMKGDRETPKCGFSR-TIVDLLNKARA---DYHT---FDILE---DEEVRQGLKKFS----NWP----------TYPQLYLDGE--------LVGGLDV----VKEELLDTHFLR-QIPRIRND------------ | atgccaattcaagaaatcaagtcaggagaggaggttgctgcgttcattaaggaaccatct ccagctgttcttcatttctacgcatcgtgggctccaagctgtgagcaagttaatcagcta ctggatgatttgctcgctgaaattgctctgccgcttcgtgccgccttcattgacgctgaa gctttgcccggaatttcgttgaatttcaagattactgctgcgccgacgcttgtatttttt agtaatggcaaagaagtcgacagaatcgatggcttcgtgccaaaagagatccaatcaaaa gttgttcttgttgccagccgatccctttcccagagctcatcggatgcttcatctacaacc tcttccacaccttcactgactccacaacaagaaaaggaagctctaaatgccagattgggt gcgctggtcaattctcaaaaagtcatggttttcatgaaaggagatccatcggcgccacgt tgtggtttctcgagaactattgtcgaacttttaaattcacataaaatcaaattcggatcc ttcgacattttctcggatgaagcagttcgccaaggattaaaggaatactcgaattggcca acttatcctcaactctatttcgatggagaattgattggagggcttgatgtcgtcaaggag gagttttcagatccacaatttatcaagcaacttccgaaagttggagaaaatagtgagggg ggatcacttgaagatcgactcaaaaagcttgtctcatcgcaaagattgatgctatttatg aaaggagatcgggagacaccaaagtgcggattttcacgaactatcgttgatttgttgaac aaggctcgtgctgattatcatacttttgatatcttggaggatgaggaagtcaggcaaggg cttaagaaattctccaattggccaacttatccacagctctatctcgacggagagctcgtc ggagggcttgatgtcgtcaaggaagagctcttggatacgcatttccttcgtcagattcca agaattagaaatgattaa |
| Q95X14 | UniRef cluster | -------------------------------------------EDRLKKLVSSQRLMLFMKGDRETPKCGFSR-TIVDLLNKARA---DYHT---FDILE---DEEVRQGLKKFS----NWP----------TYPQLYLDGE--------LVGGLDV----VKEELLDTHFLR-QIPRIRND------------ | atgccaattcaagaaatcaagtcaggagaggaggttgctgcgttcattaaggaaccatct ccagctgttcttcatttctacgcatcgtgggctccaagctgtgagcaagttaatcagcta ctggatgatttgctcgctgaaattgctctgccgcttcgtgccgccttcattgacgctgaa gctttgcccggaatttcgttgaatttcaagattactgctgcgccgacgcttgtatttttt agtaatggcaaagaagtcgacagaatcgatggcttcgtgccaaaagagatccaatcaaaa gttgttcttgttgccagccgatccctttcccagagctcatcggatgcttcatctacaacc tcttccacaccttcactgactccacaacaagaaaaggaagctctaaatgccagattgggt gcgctggtcaattctcaaaaagtcatggttttcatgaaaggagatccatcggcgccacgt tgtggtttctcgagaactattgtcgaacttttaaattcacataaaatcaaattcggatcc ttcgacattttctcggatgaagcagttcgccaaggattaaaggaatactcgaattggcca acttatcctcaactctatttcgatggagaattgattggagggcttgatgtcgtcaaggag gagttttcagatccacaatttatcaagcaacttccgaaagttggagaaaatagtgagggg ggatcacttgaagatcgactcaaaaagcttgtctcatcgcaaagattgatgctatttatg aaaggagatcgggagacaccaaagtgcggattttcacgaactatcgttgatttgttgaac aaggctcgtgctgattatcatacttttgatatcttggaggatgaggaagtcaggcaaggg cttaagaaattctccaattggccaacttatccacagctctatctcgacggagagctcgtc ggagggcttgatgtcgtcaaggaagagctcttggatacgcatttccttcgtcagattcca agaattagaaatgattaa |
| Q95X14 | UniRef cluster | -------------------------------------------NARLGALVNSQKVMVFMKGDPSAPRCGFSR-TIVELLNSHKI---KFGS---FDIFS---DEAVRQGLKEYS----NWP----------TYPQLYFDGE--------LIGGLDV----VKEEFSDPQFIK-QLPKVGENS----------- | atgccaattcaagaaatcaagtcaggagaggaggttgctgcgttcattaaggaaccatct ccagctgttcttcatttctacgcatcgtgggctccaagctgtgagcaagttaatcagcta ctggatgatttgctcgctgaaattgctctgccgcttcgtgccgccttcattgacgctgaa gctttgcccggaatttcgttgaatttcaagattactgctgcgccgacgcttgtatttttt agtaatggcaaagaagtcgacagaatcgatggcttcgtgccaaaagagatccaatcaaaa gttgttcttgttgccagccgatccctttcccagagctcatcggatgcttcatctacaacc tcttccacaccttcactgactccacaacaagaaaaggaagctctaaatgccagattgggt gcgctggtcaattctcaaaaagtcatggttttcatgaaaggagatccatcggcgccacgt tgtggtttctcgagaactattgtcgaacttttaaattcacataaaatcaaattcggatcc ttcgacattttctcggatgaagcagttcgccaaggattaaaggaatactcgaattggcca acttatcctcaactctatttcgatggagaattgattggagggcttgatgtcgtcaaggag gagttttcagatccacaatttatcaagcaacttccgaaagttggagaaaatagtgagggg ggatcacttgaagatcgactcaaaaagcttgtctcatcgcaaagattgatgctatttatg aaaggagatcgggagacaccaaagtgcggattttcacgaactatcgttgatttgttgaac aaggctcgtgctgattatcatacttttgatatcttggaggatgaggaagtcaggcaaggg cttaagaaattctccaattggccaacttatccacagctctatctcgacggagagctcgtc ggagggcttgatgtcgtcaaggaagagctcttggatacgcatttccttcgtcagattcca agaattagaaatgattaa |
| Q6EZG4 | UniRef cluster | -------------------------------------------NARLGALVNSQKVMVFMKGDPSAPRCGFSR-TIVELLNSHKI---KFGS---FDIFS---DEAVRQGLKEYS----NWP----------TYPQLYFDGE--------LIGGLDV----VKEEFSDPQFIK-QLPKVGENS----------- | atgccaattcaagaaatcaagtcaggagaggaggttgctgcgttcattaaggaaccatct ccagctgttcttcatttctacgcatcgtgggctccaagctgtgagcaagttaatcagcta ctggatgatttgctcgctgaaattgctctgccgcttcgtgccgccttcattgacgctgaa gctttgcccggaatttcgttgaatttcaagattactgctgcgccgacgcttgtatttttt agtaatggcaaagaagtcgacagaatcgatggcttcgtgccaaaagagatccaatcaaaa gttgttcttgttgccagccgatccctttcccagagctcatcggatgcttcatctacaacc tcttccacaccttcactgactccacaacaagaaaaggaagctctaaatgccagattgggt gcgctggtcaattctcaaaaagtcatggttttcatgaaaggagatccatcggcgccacgt tgtggtttctcgagaactattgtcgaacttttaaattcacataaaatcaaattcggatcc ttcgacattttctcggatgaagcagttcgccaaggattaaaggaatactcgaattggcca acttatcctcaactctatttcgatggagaattgattggagggcttgatgtcgtcaaggag gagttttcagatccacaatttatcaagcaacttccgaaagttggagaaaatagtgagggg ggatcacttgaagatcgactcaaaaagcttgtctcatcgcaaagattgatgctatttatg aaaggagatcgggagacaccaaagtgcggattttcacgaactatcgttgatttgttgaac aaggctcgtgctgattatcatacttttgatatcttggaggatgaggaagtcagaaattct ccaattggccaacttatccacagctctatctcgacggagagctcgtcggagggcttgatg tcgtcaaggaagagctcttggatacgcatttccttcgtcagattccaagaattagaaatg attaattaa |
| Q95X14 | UniRef cluster | -------------------------------------------NARLGALVNSQKVMVFMKGDPSAPRCGFSR-TIVELLNSHKI---KFGS---FDIFS---DEAVRQGLKEYS----NWP----------TYPQLYFDGE--------LIGGLDV----VKEEFSDPQFIK-QLPKVGENS----------- | atgccaattcaagaaatcaagtcaggagaggaggttgctgcgttcattaaggaaccatct ccagctgttcttcatttctacgcatcgtgggctccaagctgtgagcaagttaatcagcta ctggatgatttgctcgctgaaattgctctgccgcttcgtgccgccttcattgacgctgaa gctttgcccggaatttcgttgaatttcaagattactgctgcgccgacgcttgtatttttt agtaatggcaaagaagtcgacagaatcgatggcttcgtgccaaaagagatccaatcaaaa gttgttcttgttgccagccgatccctttcccagagctcatcggatgcttcatctacaacc tcttccacaccttcactgactccacaacaagaaaaggaagctctaaatgccagattgggt gcgctggtcaattctcaaaaagtcatggttttcatgaaaggagatccatcggcgccacgt tgtggtttctcgagaactattgtcgaacttttaaattcacataaaatcaaattcggatcc ttcgacattttctcggatgaagcagttcgccaaggattaaaggaatactcgaattggcca acttatcctcaactctatttcgatggagaattgattggagggcttgatgtcgtcaaggag gagttttcagatccacaatttatcaagcaacttccgaaagttggagaaaatagtgagggg ggatcacttgaagatcgactcaaaaagcttgtctcatcgcaaagattgatgctatttatg aaaggagatcgggagacaccaaagtgcggattttcacgaactatcgttgatttgttgaac aaggctcgtgctgattatcatacttttgatatcttggaggatgaggaagtcaggcaaggg cttaagaaattctccaattggccaacttatccacagctctatctcgacggagagctcgtc ggagggcttgatgtcgtcaaggaagagctcttggatacgcatttccttcgtcagattcca agaattagaaatgattaa |
| Q6EZG4 | UniRef cluster | -------------------------------------------NARLGALVNSQKVMVFMKGDPSAPRCGFSR-TIVELLNSHKI---KFGS---FDIFS---DEAVRQGLKEYS----NWP----------TYPQLYFDGE--------LIGGLDV----VKEEFSDPQFIK-QLPKVGENS----------- | atgccaattcaagaaatcaagtcaggagaggaggttgctgcgttcattaaggaaccatct ccagctgttcttcatttctacgcatcgtgggctccaagctgtgagcaagttaatcagcta ctggatgatttgctcgctgaaattgctctgccgcttcgtgccgccttcattgacgctgaa gctttgcccggaatttcgttgaatttcaagattactgctgcgccgacgcttgtatttttt agtaatggcaaagaagtcgacagaatcgatggcttcgtgccaaaagagatccaatcaaaa gttgttcttgttgccagccgatccctttcccagagctcatcggatgcttcatctacaacc tcttccacaccttcactgactccacaacaagaaaaggaagctctaaatgccagattgggt gcgctggtcaattctcaaaaagtcatggttttcatgaaaggagatccatcggcgccacgt tgtggtttctcgagaactattgtcgaacttttaaattcacataaaatcaaattcggatcc ttcgacattttctcggatgaagcagttcgccaaggattaaaggaatactcgaattggcca acttatcctcaactctatttcgatggagaattgattggagggcttgatgtcgtcaaggag gagttttcagatccacaatttatcaagcaacttccgaaagttggagaaaatagtgagggg ggatcacttgaagatcgactcaaaaagcttgtctcatcgcaaagattgatgctatttatg aaaggagatcgggagacaccaaagtgcggattttcacgaactatcgttgatttgttgaac aaggctcgtgctgattatcatacttttgatatcttggaggatgaggaagtcagaaattct ccaattggccaacttatccacagctctatctcgacggagagctcgtcggagggcttgatg tcgtcaaggaagagctcttggatacgcatttccttcgtcagattccaagaattagaaatg attaattaa |
| Q6EZG4 | UniRef cluster | -------------------------------------------EDRLKKLVSSQRLMLFMKGDRETPKCGFSR-TIVDLLNKARA---DYHT---FDILE---DEEVRN---SPI----GQL----------IHSSISTESS--------SEGLMSS----RKSSWIRISFVRFQELEMIN------------- | atgccaattcaagaaatcaagtcaggagaggaggttgctgcgttcattaaggaaccatct ccagctgttcttcatttctacgcatcgtgggctccaagctgtgagcaagttaatcagcta ctggatgatttgctcgctgaaattgctctgccgcttcgtgccgccttcattgacgctgaa gctttgcccggaatttcgttgaatttcaagattactgctgcgccgacgcttgtatttttt agtaatggcaaagaagtcgacagaatcgatggcttcgtgccaaaagagatccaatcaaaa gttgttcttgttgccagccgatccctttcccagagctcatcggatgcttcatctacaacc tcttccacaccttcactgactccacaacaagaaaaggaagctctaaatgccagattgggt gcgctggtcaattctcaaaaagtcatggttttcatgaaaggagatccatcggcgccacgt tgtggtttctcgagaactattgtcgaacttttaaattcacataaaatcaaattcggatcc ttcgacattttctcggatgaagcagttcgccaaggattaaaggaatactcgaattggcca acttatcctcaactctatttcgatggagaattgattggagggcttgatgtcgtcaaggag gagttttcagatccacaatttatcaagcaacttccgaaagttggagaaaatagtgagggg ggatcacttgaagatcgactcaaaaagcttgtctcatcgcaaagattgatgctatttatg aaaggagatcgggagacaccaaagtgcggattttcacgaactatcgttgatttgttgaac aaggctcgtgctgattatcatacttttgatatcttggaggatgaggaagtcagaaattct ccaattggccaacttatccacagctctatctcgacggagagctcgtcggagggcttgatg tcgtcaaggaagagctcttggatacgcatttccttcgtcagattccaagaattagaaatg attaattaa |
| Q6EZG4 | UniRef cluster | -------------------------------------------EDRLKKLVSSQRLMLFMKGDRETPKCGFSR-TIVDLLNKARA---DYHT---FDILE---DEEVRN---SPI----GQL----------IHSSISTESS--------SEGLMSS----RKSSWIRISFVRFQELEMIN------------- | atgccaattcaagaaatcaagtcaggagaggaggttgctgcgttcattaaggaaccatct ccagctgttcttcatttctacgcatcgtgggctccaagctgtgagcaagttaatcagcta ctggatgatttgctcgctgaaattgctctgccgcttcgtgccgccttcattgacgctgaa gctttgcccggaatttcgttgaatttcaagattactgctgcgccgacgcttgtatttttt agtaatggcaaagaagtcgacagaatcgatggcttcgtgccaaaagagatccaatcaaaa gttgttcttgttgccagccgatccctttcccagagctcatcggatgcttcatctacaacc tcttccacaccttcactgactccacaacaagaaaaggaagctctaaatgccagattgggt gcgctggtcaattctcaaaaagtcatggttttcatgaaaggagatccatcggcgccacgt tgtggtttctcgagaactattgtcgaacttttaaattcacataaaatcaaattcggatcc ttcgacattttctcggatgaagcagttcgccaaggattaaaggaatactcgaattggcca acttatcctcaactctatttcgatggagaattgattggagggcttgatgtcgtcaaggag gagttttcagatccacaatttatcaagcaacttccgaaagttggagaaaatagtgagggg ggatcacttgaagatcgactcaaaaagcttgtctcatcgcaaagattgatgctatttatg aaaggagatcgggagacaccaaagtgcggattttcacgaactatcgttgatttgttgaac aaggctcgtgctgattatcatacttttgatatcttggaggatgaggaagtcagaaattct ccaattggccaacttatccacagctctatctcgacggagagctcgtcggagggcttgatg tcgtcaaggaagagctcttggatacgcatttccttcgtcagattccaagaattagaaatg attaattaa |
| A7R2K3 | UniRef cluster | -------------------------------------------KIHLQKVIETQPVMLFMKGSPEEPKCGFSR-KVVEILREEKV---KFGS---FDILL---DTEVREGLKKFS----NWP----------TFPQLYCKGE--------LLGGCDI----AIAMHESGELKE-VFRDHGIET----------- | atggagggtgcagatccatctagtttggccaacaaagttgctaaagttgctgggtcaatt aaccctggggaagctgcagcccctgccagccttgggatggctgctgggcccactgtcctt gaaacagtcaaagagtttgccaaagaaaatggtgcttcccaagtggaaagtcaattgcca tcaggtctgagtgatacactaaaaatacatctccagaaggtaatcgagacacaaccagtc atgcttttcatgaaaggaagcccagaagagcccaagtgtggatttagccgaaaagttgtt gaaattttgagggaagagaaggtcaaatttgggagctttgatattctgctggacactgag gtacgcgaggggttgaagaagttctccaactggccaacatttcctcagctctactgcaag ggagagcttcttggcgggtgtgatatagcaatagcaatgcatgagagtggtgaattgaaa gaggttttcagagatcatgggattgaaacttctgacttgaacgaggcaaaagaaactaaa cctggaagtggcaagggtggcatctctgagtccactggcttgagtgtgaccttaacctcc aggcttgaaagtttgatcaattcaagcccagttatcctgtttatgaagggaaaaccagat gaacctaggtgtggtttcagccgaaaggtggttgaaattcttcaacaagagaaggtggat tttgggagctttgacattctttctgatgatgaagttcggcaagggctcaaagttcactca aactggtccagttaccctcagctttacataaagggtgaacttattggtggatcagacatt gtattagagatgcagaaaagtggggagcttgagagggttttagctgagaaagggattact cagaaagagactcttgaggatcgtgtaaggaatttaataaattcttcaccgacgatgctc tttatgaagggtacccctgatgctcccaagtgtggtttcagctctaaggttgtggatgct ttgagggcagaaaatgtgagttttggctccttcgatattctaactgatgaggaggtaaga cagggtttaaaggtcttctcaaactggccgacatttcctcagctttattacaagggtgag ttgataggaggttgtgatatcataatggagttgcgaaacaatggagagctcaaatctacc ctatctgagtag |
| A7R2K3 | UniRef cluster | -------------------------------------------TSRLESLINSSPVILFMKGKPDEPRCGFSR-KVVEILQQEKV---DFGS---FDILS---DDEVRQGLKVHS----NWS----------SYPQLYIKGE--------LIGGSDI----VLEMQKSGELER-VLAEKGITQ----------- | atggagggtgcagatccatctagtttggccaacaaagttgctaaagttgctgggtcaatt aaccctggggaagctgcagcccctgccagccttgggatggctgctgggcccactgtcctt gaaacagtcaaagagtttgccaaagaaaatggtgcttcccaagtggaaagtcaattgcca tcaggtctgagtgatacactaaaaatacatctccagaaggtaatcgagacacaaccagtc atgcttttcatgaaaggaagcccagaagagcccaagtgtggatttagccgaaaagttgtt gaaattttgagggaagagaaggtcaaatttgggagctttgatattctgctggacactgag gtacgcgaggggttgaagaagttctccaactggccaacatttcctcagctctactgcaag ggagagcttcttggcgggtgtgatatagcaatagcaatgcatgagagtggtgaattgaaa gaggttttcagagatcatgggattgaaacttctgacttgaacgaggcaaaagaaactaaa cctggaagtggcaagggtggcatctctgagtccactggcttgagtgtgaccttaacctcc aggcttgaaagtttgatcaattcaagcccagttatcctgtttatgaagggaaaaccagat gaacctaggtgtggtttcagccgaaaggtggttgaaattcttcaacaagagaaggtggat tttgggagctttgacattctttctgatgatgaagttcggcaagggctcaaagttcactca aactggtccagttaccctcagctttacataaagggtgaacttattggtggatcagacatt gtattagagatgcagaaaagtggggagcttgagagggttttagctgagaaagggattact cagaaagagactcttgaggatcgtgtaaggaatttaataaattcttcaccgacgatgctc tttatgaagggtacccctgatgctcccaagtgtggtttcagctctaaggttgtggatgct ttgagggcagaaaatgtgagttttggctccttcgatattctaactgatgaggaggtaaga cagggtttaaaggtcttctcaaactggccgacatttcctcagctttattacaagggtgag ttgataggaggttgtgatatcataatggagttgcgaaacaatggagagctcaaatctacc ctatctgagtag |
| A7R2K3 | UniRef cluster | -------------------------------------------EDRVRNLINSSPTMLFMKGTPDAPKCGFSS-KVVDALRAENV---SFGS---FDILT---DEEVRQGLKVFS----NWP----------TFPQLYYKGE--------LIGGCDI----IMELRNNGELKS-TLSE---------------- | atggagggtgcagatccatctagtttggccaacaaagttgctaaagttgctgggtcaatt aaccctggggaagctgcagcccctgccagccttgggatggctgctgggcccactgtcctt gaaacagtcaaagagtttgccaaagaaaatggtgcttcccaagtggaaagtcaattgcca tcaggtctgagtgatacactaaaaatacatctccagaaggtaatcgagacacaaccagtc atgcttttcatgaaaggaagcccagaagagcccaagtgtggatttagccgaaaagttgtt gaaattttgagggaagagaaggtcaaatttgggagctttgatattctgctggacactgag gtacgcgaggggttgaagaagttctccaactggccaacatttcctcagctctactgcaag ggagagcttcttggcgggtgtgatatagcaatagcaatgcatgagagtggtgaattgaaa gaggttttcagagatcatgggattgaaacttctgacttgaacgaggcaaaagaaactaaa cctggaagtggcaagggtggcatctctgagtccactggcttgagtgtgaccttaacctcc aggcttgaaagtttgatcaattcaagcccagttatcctgtttatgaagggaaaaccagat gaacctaggtgtggtttcagccgaaaggtggttgaaattcttcaacaagagaaggtggat tttgggagctttgacattctttctgatgatgaagttcggcaagggctcaaagttcactca aactggtccagttaccctcagctttacataaagggtgaacttattggtggatcagacatt gtattagagatgcagaaaagtggggagcttgagagggttttagctgagaaagggattact cagaaagagactcttgaggatcgtgtaaggaatttaataaattcttcaccgacgatgctc tttatgaagggtacccctgatgctcccaagtgtggtttcagctctaaggttgtggatgct ttgagggcagaaaatgtgagttttggctccttcgatattctaactgatgaggaggtaaga cagggtttaaaggtcttctcaaactggccgacatttcctcagctttattacaagggtgag ttgataggaggttgtgatatcataatggagttgcgaaacaatggagagctcaaatctacc ctatctgagtag |
| Q86H62 | UniRef cluster | -------------------------------------------NERLEKLVNQSPVMLFMKGNPEKPQCGFSN-KTVTILKENGF---EFGS---FDILQ---DQAVRNGLKEYS----NWP----------TYPQLYINGK--------LVGGYDI----IKDLNEEGELID-LKP----------------- | atggcaaatacaattaatacaaatgaagaatttgaaaatattttaaaagataataaattt ttagttgttatgttttgggcagattggtcaaaaccatcaactcaaatgagagatgttttt gatcaattagctaaacaagctacaaatcaagcaaacaataaacttttattcttaaaagtt gaagcagagaaagttcatcaaatttctggtagatataatgttaaatctgtaccaacttgt atatttttaaatcaaggtaaattagttcaatcagtcgttggtgctaatccatcagaatta gcattacaaacaaataatttctcaaaaacttgtgatacattaccattggaagagcaacaa caacaacaacaaagtgaagaaatcataaataaaattaaattagatcaagaacaagagaaa aaattattaaatgaaagattagaaaaacttgtaaatcaatcaccagttatgttatttatg aaaggtaatccagagaaaccacaatgtggtttctcaaataaaactgttacaatcttaaaa gagaatggttttgaatttggttcattcgatatcttacaagatcaagcagttcgtaatggt ttaaaggaatattcgaattggccaacttatcctcaattatatatcaatggtaaattagtt ggtggttatgatattattaaagatttaaatgaagaaggtgaattaattgatcttaagcca tag |
| Q86H62 | UniRef cluster | -------------------------------------------NERLEKLVNQSPVMLFMKGNPEKPQCGFSN-KTVTILKENGF---EFGS---FDILQ---DQAVRNGLKEYS----NWP----------TYPQLYINGK--------LVGGYDI----IKDLNEEGELID-LKP----------------- | atggcaaatacaattaatacaaatgaagaatttgaaaatattttaaaagataataaattt ttagttgttatgttttgggcagattggtcaaaaccatcaactcaaatgagagatgttttt gatcaattagctaaacaagctacaaatcaagcaaacaataaacttttattcttaaaagtt gaagcagagaaagttcatcaaatttctggtagatataatgttaaatctgtaccaacttgt atatttttaaatcaaggtaaattagttcaatcagtcgttggtgctaatccatcagaatta gcattacaaacaaataatttctcaaaaacttgtgatacattaccattggaagagcaacaa caacaacaacaaagtgaagaaatcataaataaaattaaattagatcaagaacaagagaaa aaattattaaatgaaagattagaaaaacttgtaaatcaatcaccagttatgttatttatg aaaggtaatccagagaaaccacaatgtggtttctcaaataaaactgttacaatcttaaaa gagaatggttttgaatttggttcattcgatatcttacaagatcaagcagttcgtaatggt ttaaaggaatattcgaattggccaacttatcctcaattatatatcaatggtaaattagtt ggtggttatgatattattaaagatttaaatgaagaaggtgaattaattgatcttaagcca tag |
| A8Y105 | UniRef cluster | -------------------------------------------NNRLKSLINSHRVMLFMKGNPSSPRCGFSR-TIVDLLNTHNV---EFGS---FDIFS---DEAVRQGLKEYS----NWP----------TYPQLYLDGE--------LVGGLDV----VKEEFQDQGFID-GLPKVGGSG----------- | atgccgattcaagagctcaaaacgggcgaagacgtcgccgcgttcatcaaggacccgtcg ccggcggtgcttcatttctttgccacgtgggcgccgagctgtgagcaagtgaatcagctg ctcgacgatcttctggccgaaatcgcgttgccacttcgcgcggcgtacattgatgcggaa gcgctgccaggaatctcgctgaatttcaaaattacagccgcaccgacgctggttttcttt agtaatggcaaagaagtggaccgtgtcgatggcttcattccgaaagagatccaatccaaa gtggtccttgtcgccagccgctctctctcccaatcctctttagactccaactccaccatc tcctcctcaacgccttcattgactccagaacaggagaaggatgctctcaacaatcgtctc aaatctctgatcaactctcaccgagttatgctttttatgaaaggaaatccatcgtcgcca cgttgcggattctccagaaccattgtggatcttttgaacacccataacgtcgagtttgga tcctttgacatcttctccgatgaagctgtacgtcagggtttgaaggagtactccaactgg ccaacgtatccacagttgtatctcgatggagagcttgtcggaggactggatgtcgttaag gaggagttccaagatcaaggattcattgatggactgccgaaagttggaggatcaggaaag gaagatctggagaagagactcaaggatctcgtttcatcgcatcggttgatgttgttcatg aaggggaataaggaaatgccgaagtgcggattctcgaggactattgtggagttgttgaac aatgcacgcgccgattttcacacttttgatattctggaggatgaggaagttagacaggga ttgaaggaattctccaactggccaacctacccacaactctatctcgatggcgagctcatc ggcggactcgacgttgtcaaggaggaacttctggacacccatttcttgcgtcagattccc agaattcggaatgaatga |
| A8Y105 | UniRef cluster | -------------------------------------------NNRLKSLINSHRVMLFMKGNPSSPRCGFSR-TIVDLLNTHNV---EFGS---FDIFS---DEAVRQGLKEYS----NWP----------TYPQLYLDGE--------LVGGLDV----VKEEFQDQGFID-GLPKVGGSG----------- | atgccgattcaagagctcaaaacgggcgaagacgtcgccgcgttcatcaaggacccgtcg ccggcggtgcttcatttctttgccacgtgggcgccgagctgtgagcaagtgaatcagctg ctcgacgatcttctggccgaaatcgcgttgccacttcgcgcggcgtacattgatgcggaa gcgctgccaggaatctcgctgaatttcaaaattacagccgcaccgacgctggttttcttt agtaatggcaaagaagtggaccgtgtcgatggcttcattccgaaagagatccaatccaaa gtggtccttgtcgccagccgctctctctcccaatcctctttagactccaactccaccatc tcctcctcaacgccttcattgactccagaacaggagaaggatgctctcaacaatcgtctc aaatctctgatcaactctcaccgagttatgctttttatgaaaggaaatccatcgtcgcca cgttgcggattctccagaaccattgtggatcttttgaacacccataacgtcgagtttgga tcctttgacatcttctccgatgaagctgtacgtcagggtttgaaggagtactccaactgg ccaacgtatccacagttgtatctcgatggagagcttgtcggaggactggatgtcgttaag gaggagttccaagatcaaggattcattgatggactgccgaaagttggaggatcaggaaag gaagatctggagaagagactcaaggatctcgtttcatcgcatcggttgatgttgttcatg aaggggaataaggaaatgccgaagtgcggattctcgaggactattgtggagttgttgaac aatgcacgcgccgattttcacacttttgatattctggaggatgaggaagttagacaggga ttgaaggaattctccaactggccaacctacccacaactctatctcgatggcgagctcatc ggcggactcgacgttgtcaaggaggaacttctggacacccatttcttgcgtcagattccc agaattcggaatgaatga |
| A8Y105 | UniRef cluster | -------------------------------------------EKRLKDLVSSHRLMLFMKGNKEMPKCGFSR-TIVELLNNARA---DFHT---FDILE---DEEVRQGLKEFS----NWP----------TYPQLYLDGE--------LIGGLDV----VKEELLDTHFLR-QIPRIRNE------------ | atgccgattcaagagctcaaaacgggcgaagacgtcgccgcgttcatcaaggacccgtcg ccggcggtgcttcatttctttgccacgtgggcgccgagctgtgagcaagtgaatcagctg ctcgacgatcttctggccgaaatcgcgttgccacttcgcgcggcgtacattgatgcggaa gcgctgccaggaatctcgctgaatttcaaaattacagccgcaccgacgctggttttcttt agtaatggcaaagaagtggaccgtgtcgatggcttcattccgaaagagatccaatccaaa gtggtccttgtcgccagccgctctctctcccaatcctctttagactccaactccaccatc tcctcctcaacgccttcattgactccagaacaggagaaggatgctctcaacaatcgtctc aaatctctgatcaactctcaccgagttatgctttttatgaaaggaaatccatcgtcgcca cgttgcggattctccagaaccattgtggatcttttgaacacccataacgtcgagtttgga tcctttgacatcttctccgatgaagctgtacgtcagggtttgaaggagtactccaactgg ccaacgtatccacagttgtatctcgatggagagcttgtcggaggactggatgtcgttaag gaggagttccaagatcaaggattcattgatggactgccgaaagttggaggatcaggaaag gaagatctggagaagagactcaaggatctcgtttcatcgcatcggttgatgttgttcatg aaggggaataaggaaatgccgaagtgcggattctcgaggactattgtggagttgttgaac aatgcacgcgccgattttcacacttttgatattctggaggatgaggaagttagacaggga ttgaaggaattctccaactggccaacctacccacaactctatctcgatggcgagctcatc ggcggactcgacgttgtcaaggaggaacttctggacacccatttcttgcgtcagattccc agaattcggaatgaatga |
| A8Y105 | UniRef cluster | -------------------------------------------EKRLKDLVSSHRLMLFMKGNKEMPKCGFSR-TIVELLNNARA---DFHT---FDILE---DEEVRQGLKEFS----NWP----------TYPQLYLDGE--------LIGGLDV----VKEELLDTHFLR-QIPRIRNE------------ | atgccgattcaagagctcaaaacgggcgaagacgtcgccgcgttcatcaaggacccgtcg ccggcggtgcttcatttctttgccacgtgggcgccgagctgtgagcaagtgaatcagctg ctcgacgatcttctggccgaaatcgcgttgccacttcgcgcggcgtacattgatgcggaa gcgctgccaggaatctcgctgaatttcaaaattacagccgcaccgacgctggttttcttt agtaatggcaaagaagtggaccgtgtcgatggcttcattccgaaagagatccaatccaaa gtggtccttgtcgccagccgctctctctcccaatcctctttagactccaactccaccatc tcctcctcaacgccttcattgactccagaacaggagaaggatgctctcaacaatcgtctc aaatctctgatcaactctcaccgagttatgctttttatgaaaggaaatccatcgtcgcca cgttgcggattctccagaaccattgtggatcttttgaacacccataacgtcgagtttgga tcctttgacatcttctccgatgaagctgtacgtcagggtttgaaggagtactccaactgg ccaacgtatccacagttgtatctcgatggagagcttgtcggaggactggatgtcgttaag gaggagttccaagatcaaggattcattgatggactgccgaaagttggaggatcaggaaag gaagatctggagaagagactcaaggatctcgtttcatcgcatcggttgatgttgttcatg aaggggaataaggaaatgccgaagtgcggattctcgaggactattgtggagttgttgaac aatgcacgcgccgattttcacacttttgatattctggaggatgaggaagttagacaggga ttgaaggaattctccaactggccaacctacccacaactctatctcgatggcgagctcatc ggcggactcgacgttgtcaaggaggaacttctggacacccatttcttgcgtcagattccc agaattcggaatgaatga |
| Q5KJR8 | UniRef cluster | -------------------------------------------VARCHELMNKHKVVLFMKGNPTAPKCGFSR-QTVGLLREQGV---EFAW---FDIFS---DEDVRQGLKKVN----DWP----------TFPQIIVNGE--------LVGGLDI----LREMIENGE--WQELMDSIEEGKAE-------- | atgtccgcaagcaacctcgtccaagtcaattcccccgaacacttcagggaactcctctct gcagacctcaacagggtttcgtgcctcaatttctgggcaccttgggcagagccatgtgtc gctttcaacaacgctgtagagcaagaggcggcccaatttccttcagttctcttcttaaac attgaagctgaacaattggccgacatctccgaatcatttgacattgaagcagtcccttcc ttccttgtccttcgtgggcacactctcctggctcgacactctggcgccgacgcttccctc cttcgttctctcctcactcaacacgcctccccctctgcccccctctctacctcttccgcc caaccccaagcccctgccgccgcccaacgtccccggacagaagcagaaattgtcgcccgc tgccacgaactgatgaacaagcacaaggttgtgttattcatgaagggaaaccctacagct cctaagtgtgggttctcaaggcagactgtgggattattgagggagcaaggagtggaattt gcttggtttgatatctttagtgatgaggatgtaaggcaaggattgaagaaggttaatgac tggcctacattcccgcaaatcattgtgaatggcgagttggtaggcggtcttgatattttg cgggagatgattgagaatggagaatggcaagagttgatggattctatcgaggagggcaag gcggaataa |
| Q5KJR7 | UniRef cluster | -------------------------------------------VARCHELMNKHKVVLFMKGNPTAPKCGFSR-QTVGLLREQGV---EFAW---FDIFS---DEDVRQGLKKVN----DWP----------TFPQIIVNGE--------LVGGLDI----LREMIENGE--WQELMDSIEEGKAE-------- | atgaattctacacctacgcctacacctcaaccggatacggataacccccattctaatgac gccaatcatgctgatgatcggcgacctttggctgctcatatccctgactatgctgggaaa tcgcctttccctgccccgccctcgcgttttactcctcgtacacctttacaattcctatcc aattcacctaccaacaccgacgcctccgcgatatcttgcccttcttatattctccccact cttccttcctctgccgcctctctcccctctctcgaccctccatcgtccaacgaccgcttg gcgatgatcaaccgtatcaaatcactgggttcctacaccaattcacctgctaccgtcaaa attcctctccccaccccctccccgtccgtatctttatccctctcttctcgcggtcccgca acaccttcatcctctatcatccgcaacaaaccccacatgccatctccacttaacaaactc gattacctatacgacatcccaccttcccctcccatctccgccgatgctgacccgggtcaa ttactcgtcatgccgcgaatgccaaatcccgatgtcgaatcgcccatcgcgtcacctttt gtcaacctcaaccttcaaacgtcgacttcatcgaaccggaaacccggtgggcacactctc ctggctcgacactctggcgccgacgcttccctccttcgttctctcctcactcaacacgcc tccccctctgcccccctctctacctcttccgcccaaccccaagcccctgccgccgcccaa cgtccccggacagaagcagaaattgtcgcccgctgccacgaactgatgaacaagcacaag gttgtgttattcatgaagggaaaccctacagctcctaagtgtgggttctcaaggcagact gtgggattattgagggagcaaggagtggaatttgcttggtttgatatctttagtgatgag gatgtaaggcaaggattgaagaaggttaatgactggcctacattcccgcaaatcattgtg aatggcgagttggtaggcggtcttgatattttgcgggagatgattgagaatggagaatgg caagagttgatggattctatcgaggagggcaaggcggaataa |
| Q55WH9 | UniRef cluster | -------------------------------------------VARCHELMNKHKVVLFMKGNPTAPKCGFSR-QTVGLLREQGV---EFAW---FDIFS---DEDVRQGLKKVN----DWP----------TFPQIIVNGE--------LVGGLDI----LREMIENGE--WQELMDSIEEGKAE-------- | atgaattctacacctacgcctacacctcaaccggatacggataacccccattctaatgac gccaatcatgctgatgatcggcgacctttggctgctcatatccctgactatgctgggaaa tcgcctttccctgccccgccctcgcgttttactcctcgtacacctttacaattcctatcc aattcacctaccaacaccgacgcctccgcgatatcttgcccttcttatattctccccact cttccttcctctgccgcctctctcccctctctcgaccctccatcgtccaacgaccgcttg gcgatgatcaaccgtatcaaatcactgggttcctacaccaattcacctgctaccgtcaaa attcctctccccaccccctccccgtccgtatctttatccctctcttctcgcggtcccgca acaccttcatcctctatcatccgcaacaaaccccacatgccatctccacttaacaaactc gattacctatacgacatcccaccttcccctcccatctccgccgatgctgacccgggtcaa ttactcgtcatgccgcgaatgccaaatcccgatgtcgaatcgcccatcgcgtcacctttt gtcaacctcaaccttcaaacgtcgacttcatcgaaccggaaacccggtgtgggtgttgtg atttttgactcacctcctcgtcgacggatatcgggtaattctcatggtccgcgacgcggt tcggctctggctaatgaatgggggcacactctcctggctcgacactctggcgccgacgct tccctccttcgttctctcctcactcaacacgcctccccctctgcccccctctctacctct tccgcccaaccccaagcccctgccgccgcccaacgtccccggacagaagcagaaattgtc gcccgctgccacgaactgatgaacaagcacaaggttgtgttattcatgaagggaaaccct acagctcctaagtgtgggttctcaaggcagactgtgggattattgagggagcaaggagtg gaatttgcttggtttgatatctttagtgatgaggatgtaaggcaaggattgaagaaggtt aatgactggcctacattcccgcaaatcattgtgaatggcgagttggtaggcggtcttgat attttgcgggagatgattgagaatggagaatggcaagagttgatggattctatcgaggag ggcaaggcggaataa |
| Q5KJR8 | UniRef cluster | -------------------------------------------VARCHELMNKHKVVLFMKGNPTAPKCGFSR-QTVGLLREQGV---EFAW---FDIFS---DEDVRQGLKKVN----DWP----------TFPQIIVNGE--------LVGGLDI----LREMIENGE--WQELMDSIEEGKAE-------- | atgtccgcaagcaacctcgtccaagtcaattcccccgaacacttcagggaactcctctct gcagacctcaacagggtttcgtgcctcaatttctgggcaccttgggcagagccatgtgtc gctttcaacaacgctgtagagcaagaggcggcccaatttccttcagttctcttcttaaac attgaagctgaacaattggccgacatctccgaatcatttgacattgaagcagtcccttcc ttccttgtccttcgtgggcacactctcctggctcgacactctggcgccgacgcttccctc cttcgttctctcctcactcaacacgcctccccctctgcccccctctctacctcttccgcc caaccccaagcccctgccgccgcccaacgtccccggacagaagcagaaattgtcgcccgc tgccacgaactgatgaacaagcacaaggttgtgttattcatgaagggaaaccctacagct cctaagtgtgggttctcaaggcagactgtgggattattgagggagcaaggagtggaattt gcttggtttgatatctttagtgatgaggatgtaaggcaaggattgaagaaggttaatgac tggcctacattcccgcaaatcattgtgaatggcgagttggtaggcggtcttgatattttg cgggagatgattgagaatggagaatggcaagagttgatggattctatcgaggagggcaag gcggaataa |
| Q5KJR7 | UniRef cluster | -------------------------------------------VARCHELMNKHKVVLFMKGNPTAPKCGFSR-QTVGLLREQGV---EFAW---FDIFS---DEDVRQGLKKVN----DWP----------TFPQIIVNGE--------LVGGLDI----LREMIENGE--WQELMDSIEEGKAE-------- | atgaattctacacctacgcctacacctcaaccggatacggataacccccattctaatgac gccaatcatgctgatgatcggcgacctttggctgctcatatccctgactatgctgggaaa tcgcctttccctgccccgccctcgcgttttactcctcgtacacctttacaattcctatcc aattcacctaccaacaccgacgcctccgcgatatcttgcccttcttatattctccccact cttccttcctctgccgcctctctcccctctctcgaccctccatcgtccaacgaccgcttg gcgatgatcaaccgtatcaaatcactgggttcctacaccaattcacctgctaccgtcaaa attcctctccccaccccctccccgtccgtatctttatccctctcttctcgcggtcccgca acaccttcatcctctatcatccgcaacaaaccccacatgccatctccacttaacaaactc gattacctatacgacatcccaccttcccctcccatctccgccgatgctgacccgggtcaa ttactcgtcatgccgcgaatgccaaatcccgatgtcgaatcgcccatcgcgtcacctttt gtcaacctcaaccttcaaacgtcgacttcatcgaaccggaaacccggtgggcacactctc ctggctcgacactctggcgccgacgcttccctccttcgttctctcctcactcaacacgcc tccccctctgcccccctctctacctcttccgcccaaccccaagcccctgccgccgcccaa cgtccccggacagaagcagaaattgtcgcccgctgccacgaactgatgaacaagcacaag gttgtgttattcatgaagggaaaccctacagctcctaagtgtgggttctcaaggcagact gtgggattattgagggagcaaggagtggaatttgcttggtttgatatctttagtgatgag gatgtaaggcaaggattgaagaaggttaatgactggcctacattcccgcaaatcattgtg aatggcgagttggtaggcggtcttgatattttgcgggagatgattgagaatggagaatgg caagagttgatggattctatcgaggagggcaaggcggaataa |
| Q55WH9 | UniRef cluster | -------------------------------------------VARCHELMNKHKVVLFMKGNPTAPKCGFSR-QTVGLLREQGV---EFAW---FDIFS---DEDVRQGLKKVN----DWP----------TFPQIIVNGE--------LVGGLDI----LREMIENGE--WQELMDSIEEGKAE-------- | atgaattctacacctacgcctacacctcaaccggatacggataacccccattctaatgac gccaatcatgctgatgatcggcgacctttggctgctcatatccctgactatgctgggaaa tcgcctttccctgccccgccctcgcgttttactcctcgtacacctttacaattcctatcc aattcacctaccaacaccgacgcctccgcgatatcttgcccttcttatattctccccact cttccttcctctgccgcctctctcccctctctcgaccctccatcgtccaacgaccgcttg gcgatgatcaaccgtatcaaatcactgggttcctacaccaattcacctgctaccgtcaaa attcctctccccaccccctccccgtccgtatctttatccctctcttctcgcggtcccgca acaccttcatcctctatcatccgcaacaaaccccacatgccatctccacttaacaaactc gattacctatacgacatcccaccttcccctcccatctccgccgatgctgacccgggtcaa ttactcgtcatgccgcgaatgccaaatcccgatgtcgaatcgcccatcgcgtcacctttt gtcaacctcaaccttcaaacgtcgacttcatcgaaccggaaacccggtgtgggtgttgtg atttttgactcacctcctcgtcgacggatatcgggtaattctcatggtccgcgacgcggt tcggctctggctaatgaatgggggcacactctcctggctcgacactctggcgccgacgct tccctccttcgttctctcctcactcaacacgcctccccctctgcccccctctctacctct tccgcccaaccccaagcccctgccgccgcccaacgtccccggacagaagcagaaattgtc gcccgctgccacgaactgatgaacaagcacaaggttgtgttattcatgaagggaaaccct acagctcctaagtgtgggttctcaaggcagactgtgggattattgagggagcaaggagtg gaatttgcttggtttgatatctttagtgatgaggatgtaaggcaaggattgaagaaggtt aatgactggcctacattcccgcaaatcattgtgaatggcgagttggtaggcggtcttgat attttgcgggagatgattgagaatggagaatggcaagagttgatggattctatcgaggag ggcaaggcggaataa |
| Q5KJR8 | UniRef cluster | -------------------------------------------VARCHELMNKHKVVLFMKGNPTAPKCGFSR-QTVGLLREQGV---EFAW---FDIFS---DEDVRQGLKKVN----DWP----------TFPQIIVNGE--------LVGGLDI----LREMIENGE--WQELMDSIEEGKAE-------- | atgtccgcaagcaacctcgtccaagtcaattcccccgaacacttcagggaactcctctct gcagacctcaacagggtttcgtgcctcaatttctgggcaccttgggcagagccatgtgtc gctttcaacaacgctgtagagcaagaggcggcccaatttccttcagttctcttcttaaac attgaagctgaacaattggccgacatctccgaatcatttgacattgaagcagtcccttcc ttccttgtccttcgtgggcacactctcctggctcgacactctggcgccgacgcttccctc cttcgttctctcctcactcaacacgcctccccctctgcccccctctctacctcttccgcc caaccccaagcccctgccgccgcccaacgtccccggacagaagcagaaattgtcgcccgc tgccacgaactgatgaacaagcacaaggttgtgttattcatgaagggaaaccctacagct cctaagtgtgggttctcaaggcagactgtgggattattgagggagcaaggagtggaattt gcttggtttgatatctttagtgatgaggatgtaaggcaaggattgaagaaggttaatgac tggcctacattcccgcaaatcattgtgaatggcgagttggtaggcggtcttgatattttg cgggagatgattgagaatggagaatggcaagagttgatggattctatcgaggagggcaag gcggaataa |
| Q5KJR7 | UniRef cluster | -------------------------------------------VARCHELMNKHKVVLFMKGNPTAPKCGFSR-QTVGLLREQGV---EFAW---FDIFS---DEDVRQGLKKVN----DWP----------TFPQIIVNGE--------LVGGLDI----LREMIENGE--WQELMDSIEEGKAE-------- | atgaattctacacctacgcctacacctcaaccggatacggataacccccattctaatgac gccaatcatgctgatgatcggcgacctttggctgctcatatccctgactatgctgggaaa tcgcctttccctgccccgccctcgcgttttactcctcgtacacctttacaattcctatcc aattcacctaccaacaccgacgcctccgcgatatcttgcccttcttatattctccccact cttccttcctctgccgcctctctcccctctctcgaccctccatcgtccaacgaccgcttg gcgatgatcaaccgtatcaaatcactgggttcctacaccaattcacctgctaccgtcaaa attcctctccccaccccctccccgtccgtatctttatccctctcttctcgcggtcccgca acaccttcatcctctatcatccgcaacaaaccccacatgccatctccacttaacaaactc gattacctatacgacatcccaccttcccctcccatctccgccgatgctgacccgggtcaa ttactcgtcatgccgcgaatgccaaatcccgatgtcgaatcgcccatcgcgtcacctttt gtcaacctcaaccttcaaacgtcgacttcatcgaaccggaaacccggtgggcacactctc ctggctcgacactctggcgccgacgcttccctccttcgttctctcctcactcaacacgcc tccccctctgcccccctctctacctcttccgcccaaccccaagcccctgccgccgcccaa cgtccccggacagaagcagaaattgtcgcccgctgccacgaactgatgaacaagcacaag gttgtgttattcatgaagggaaaccctacagctcctaagtgtgggttctcaaggcagact gtgggattattgagggagcaaggagtggaatttgcttggtttgatatctttagtgatgag gatgtaaggcaaggattgaagaaggttaatgactggcctacattcccgcaaatcattgtg aatggcgagttggtaggcggtcttgatattttgcgggagatgattgagaatggagaatgg caagagttgatggattctatcgaggagggcaaggcggaataa |
| Q55WH9 | UniRef cluster | -------------------------------------------VARCHELMNKHKVVLFMKGNPTAPKCGFSR-QTVGLLREQGV---EFAW---FDIFS---DEDVRQGLKKVN----DWP----------TFPQIIVNGE--------LVGGLDI----LREMIENGE--WQELMDSIEEGKAE-------- | atgaattctacacctacgcctacacctcaaccggatacggataacccccattctaatgac gccaatcatgctgatgatcggcgacctttggctgctcatatccctgactatgctgggaaa tcgcctttccctgccccgccctcgcgttttactcctcgtacacctttacaattcctatcc aattcacctaccaacaccgacgcctccgcgatatcttgcccttcttatattctccccact cttccttcctctgccgcctctctcccctctctcgaccctccatcgtccaacgaccgcttg gcgatgatcaaccgtatcaaatcactgggttcctacaccaattcacctgctaccgtcaaa attcctctccccaccccctccccgtccgtatctttatccctctcttctcgcggtcccgca acaccttcatcctctatcatccgcaacaaaccccacatgccatctccacttaacaaactc gattacctatacgacatcccaccttcccctcccatctccgccgatgctgacccgggtcaa ttactcgtcatgccgcgaatgccaaatcccgatgtcgaatcgcccatcgcgtcacctttt gtcaacctcaaccttcaaacgtcgacttcatcgaaccggaaacccggtgtgggtgttgtg atttttgactcacctcctcgtcgacggatatcgggtaattctcatggtccgcgacgcggt tcggctctggctaatgaatgggggcacactctcctggctcgacactctggcgccgacgct tccctccttcgttctctcctcactcaacacgcctccccctctgcccccctctctacctct tccgcccaaccccaagcccctgccgccgcccaacgtccccggacagaagcagaaattgtc gcccgctgccacgaactgatgaacaagcacaaggttgtgttattcatgaagggaaaccct acagctcctaagtgtgggttctcaaggcagactgtgggattattgagggagcaaggagtg gaatttgcttggtttgatatctttagtgatgaggatgtaaggcaaggattgaagaaggtt aatgactggcctacattcccgcaaatcattgtgaatggcgagttggtaggcggtcttgat attttgcgggagatgattgagaatggagaatggcaagagttgatggattctatcgaggag ggcaaggcggaataa |
| GRS17 | UniRef cluster | -------------------------------------------KSRLEKLTNSHPVMLFMKGIPEEPRCGFSR-KVVDILKEVNV---DFGS---FDILS---DNEVREGLKKFS----NWP----------TFPQLYCNGE--------LLGGADI----AIAMHESGELKD-AFKDLGITT----------- |  |
| GRS17 | UniRef cluster | -------------------------------------------RARLEGLVNSKPVMLFMKGRPEEPKCGFSG-KVVEILNQEKI---EFGS---FDILL---DDEVRQGLKVYS----NWS----------SYPQLYVKGE--------LMGGSDI----VLEMQKSGELKK-VLTEKGITG----------- |  |
| GRS17 | UniRef cluster | -------------------------------------------EDRLKALINSSEVMLFMKGSPDEPKCGFSS-KVVKALRGENV---SFGS---FDILT---DEEVRQGIKNFS----NWP----------TFPQLYYKGE--------LIGGCDI----IMELSESGDLKA-TLSE---------------- |  |
| A8QEG2 | UniRef cluster | -------------------------------------------NNRMKSLVHHSPLTLFMKGTPDNPKCGFSS-QIVNLLRAVNA---DFSS---FDVLE---DDEVRQGLKEYS----HWP----------TFPQLYLNGE--------LIGGLDI----LKEELNDPDFRS-KLPKLKNSN----------- | atgaaatgggggaggcaaataacttggggacaacatgaaaatagttcgaaaggagatgag gcaatgcagacgaagagagtggcaaatgctactttgaaagaggaaaggaagaccgggtgg gttggaacattctcttggggtcaagatgatcgtgcatgtctggatcaagatatggcaaaa ctattaaaaaacgaggatgaattcaatcaattcatcagaatgactgaatatgctctagtg catttctctgcaaaatggtgtgaaatatgtgagcaactcaataatttattattagaattt aaagatgaactaaagtgttttgattttgctgtagtggaagcagaagaagttgccggaata tcagttgcgaataaaattgtcgcagctccaaccgttctgttttttaagaaagggaaggaa gtcgatcgtttacaaggattcgatccggctaaattaagaataaaaatcattaagcacaat tttgttgaaggcgttrctaacatgacaacaaaaccaatcgatgatgagaaaaatgatgtg aataataggatgaaatcactggtccatcactctcctcttacgctttttatgaagggaaca ccagataacccaaaatgcggattcagcagtcaaattgttaatcttttacgtgcggtaaat gcagatttttcaagttttgatgtcttggaagacgatgaggtgagacaaggattaaaagaa tatagccattggcctacatttccacagctttatttgaatggcgaacttattggtggccta gatattctcaaagaagagttaaatgatcctgactttcgcagtaaattgccgaaactgaag aatagcaatgaaagattaaaagcattaatcaatcaggctcctctaatgcttttcatgaaa ggaagtcctaaagcacctcaatgcaaattcagcaagaagattattgaactgctagctggg attaacgctgagtattcatattttgatatccttaaagatgatgagatacgagaaggattg aaagaatatagcaactggccaacttatccgcagctgtatttgaatggtgaactaatcggt ggtttggatgtggttacagaggagttgaagaatcctgattttgtggagaaaatacctaaa tcgagctga |
| A8QEG2 | UniRef cluster | -------------------------------------------NNRMKSLVHHSPLTLFMKGTPDNPKCGFSS-QIVNLLRAVNA---DFSS---FDVLE---DDEVRQGLKEYS----HWP----------TFPQLYLNGE--------LIGGLDI----LKEELNDPDFRS-KLPKLKNSN----------- | atgaaatgggggaggcaaataacttggggacaacatgaaaatagttcgaaaggagatgag gcaatgcagacgaagagagtggcaaatgctactttgaaagaggaaaggaagaccgggtgg gttggaacattctcttggggtcaagatgatcgtgcatgtctggatcaagatatggcaaaa ctattaaaaaacgaggatgaattcaatcaattcatcagaatgactgaatatgctctagtg catttctctgcaaaatggtgtgaaatatgtgagcaactcaataatttattattagaattt aaagatgaactaaagtgttttgattttgctgtagtggaagcagaagaagttgccggaata tcagttgcgaataaaattgtcgcagctccaaccgttctgttttttaagaaagggaaggaa gtcgatcgtttacaaggattcgatccggctaaattaagaataaaaatcattaagcacaat tttgttgaaggcgttrctaacatgacaacaaaaccaatcgatgatgagaaaaatgatgtg aataataggatgaaatcactggtccatcactctcctcttacgctttttatgaagggaaca ccagataacccaaaatgcggattcagcagtcaaattgttaatcttttacgtgcggtaaat gcagatttttcaagttttgatgtcttggaagacgatgaggtgagacaaggattaaaagaa tatagccattggcctacatttccacagctttatttgaatggcgaacttattggtggccta gatattctcaaagaagagttaaatgatcctgactttcgcagtaaattgccgaaactgaag aatagcaatgaaagattaaaagcattaatcaatcaggctcctctaatgcttttcatgaaa ggaagtcctaaagcacctcaatgcaaattcagcaagaagattattgaactgctagctggg attaacgctgagtattcatattttgatatccttaaagatgatgagatacgagaaggattg aaagaatatagcaactggccaacttatccgcagctgtatttgaatggtgaactaatcggt ggtttggatgtggttacagaggagttgaagaatcctgattttgtggagaaaatacctaaa tcgagctga |
| A8QEG2 | UniRef cluster | -------------------------------------------NERLKALINQAPLMLFMKGSPKAPQCKFSK-KIIELLAGINA---EYSY---FDILK---DDEIREGLKEYS----NWP----------TYPQLYLNGE--------LIGGLDV----VTEELKNPDFVE-KIP--KSS------------ | atgaaatgggggaggcaaataacttggggacaacatgaaaatagttcgaaaggagatgag gcaatgcagacgaagagagtggcaaatgctactttgaaagaggaaaggaagaccgggtgg gttggaacattctcttggggtcaagatgatcgtgcatgtctggatcaagatatggcaaaa ctattaaaaaacgaggatgaattcaatcaattcatcagaatgactgaatatgctctagtg catttctctgcaaaatggtgtgaaatatgtgagcaactcaataatttattattagaattt aaagatgaactaaagtgttttgattttgctgtagtggaagcagaagaagttgccggaata tcagttgcgaataaaattgtcgcagctccaaccgttctgttttttaagaaagggaaggaa gtcgatcgtttacaaggattcgatccggctaaattaagaataaaaatcattaagcacaat tttgttgaaggcgttrctaacatgacaacaaaaccaatcgatgatgagaaaaatgatgtg aataataggatgaaatcactggtccatcactctcctcttacgctttttatgaagggaaca ccagataacccaaaatgcggattcagcagtcaaattgttaatcttttacgtgcggtaaat gcagatttttcaagttttgatgtcttggaagacgatgaggtgagacaaggattaaaagaa tatagccattggcctacatttccacagctttatttgaatggcgaacttattggtggccta gatattctcaaagaagagttaaatgatcctgactttcgcagtaaattgccgaaactgaag aatagcaatgaaagattaaaagcattaatcaatcaggctcctctaatgcttttcatgaaa ggaagtcctaaagcacctcaatgcaaattcagcaagaagattattgaactgctagctggg attaacgctgagtattcatattttgatatccttaaagatgatgagatacgagaaggattg aaagaatatagcaactggccaacttatccgcagctgtatttgaatggtgaactaatcggt ggtttggatgtggttacagaggagttgaagaatcctgattttgtggagaaaatacctaaa tcgagctga |
| A8QEG2 | UniRef cluster | -------------------------------------------NERLKALINQAPLMLFMKGSPKAPQCKFSK-KIIELLAGINA---EYSY---FDILK---DDEIREGLKEYS----NWP----------TYPQLYLNGE--------LIGGLDV----VTEELKNPDFVE-KIP--KSS------------ | atgaaatgggggaggcaaataacttggggacaacatgaaaatagttcgaaaggagatgag gcaatgcagacgaagagagtggcaaatgctactttgaaagaggaaaggaagaccgggtgg gttggaacattctcttggggtcaagatgatcgtgcatgtctggatcaagatatggcaaaa ctattaaaaaacgaggatgaattcaatcaattcatcagaatgactgaatatgctctagtg catttctctgcaaaatggtgtgaaatatgtgagcaactcaataatttattattagaattt aaagatgaactaaagtgttttgattttgctgtagtggaagcagaagaagttgccggaata tcagttgcgaataaaattgtcgcagctccaaccgttctgttttttaagaaagggaaggaa gtcgatcgtttacaaggattcgatccggctaaattaagaataaaaatcattaagcacaat tttgttgaaggcgttrctaacatgacaacaaaaccaatcgatgatgagaaaaatgatgtg aataataggatgaaatcactggtccatcactctcctcttacgctttttatgaagggaaca ccagataacccaaaatgcggattcagcagtcaaattgttaatcttttacgtgcggtaaat gcagatttttcaagttttgatgtcttggaagacgatgaggtgagacaaggattaaaagaa tatagccattggcctacatttccacagctttatttgaatggcgaacttattggtggccta gatattctcaaagaagagttaaatgatcctgactttcgcagtaaattgccgaaactgaag aatagcaatgaaagattaaaagcattaatcaatcaggctcctctaatgcttttcatgaaa ggaagtcctaaagcacctcaatgcaaattcagcaagaagattattgaactgctagctggg attaacgctgagtattcatattttgatatccttaaagatgatgagatacgagaaggattg aaagaatatagcaactggccaacttatccgcagctgtatttgaatggtgaactaatcggt ggtttggatgtggttacagaggagttgaagaatcctgattttgtggagaaaatacctaaa tcgagctga |
| B0DE05 | UniRef cluster | -------------------------------------------NERLRGLMNQSKVVVFIKGSPQEPRCGFSR-KIVGLLKDKGV---EYKH---FDILT---DESVRQGLKKLN----DWP----------TFPQLIINGE--------LVGGLDI----VQEMAENGE--LEQALA---------------- | atgtcgaaaacaaacttctacgacgtcacttcgcccacggaattccaggaacttctctcc gcagacctcaaccgcgtatcagtgatcaatttttgggcgccatgggccgagccatgcaag caaatgaacgaagtcgtcaaggaactctcaaagaagtatcaacagactctattcctgcaa gtcgaggcggaggaacaagctgacatcgccgaatctttcgatatcgaggctgttccgacg ttcatcatccttcggggacatctcctgctcgatcgagtcgcaggcgccgacgcagcggca ctcacaaagtccgttgaaaaacacaccgcagggccatcctacaacccccaatcgcggaca gacaaagccccagccccagcccccaccaccgtcccttcttcactccaagacggggattcc aagcaacctgagagcgaggcgcagctgaacgagcgcctccgtgggctgatgaaccagagc aaggtggtggtgttcataaagggatccccgcaggaaccacgatgtgggttctcccgcaag attgtagggttgttgaaggataagggtgttgagtacaagcactttgatatcctcacggac gagagcgtcagacagggtctgaagaagctgaacgactggccgaccttcccgcagctgatc atcaacggcgagttggtgggtgggttggacatagtgcaagagatggcggagaacggagag cttgagcaggccctggcgtag |
| Q011R3 | UniRef cluster | -------------------------------------------QARLKSLIESQPVVLFMKGHPDEPKCGFSR-KVVDALNGAGI---KFGS---FNILA---DEDVRQGLKEYS----NWP----------TYPQLYVDGE--------LLGGCDI----ILEMAESGELAE-ACSEASGDG----------- | atgtcggtcgcgttgacggacgcggtggcgtacgacgcgttcaccgccgcgcgcgatcgc gcgatcgtgcacttcaccgcgagctggtgcgagccgtgtgcggcgatggagattgtcctg gaaaagattcgagagaaacacgcgggcgtcgtcacggcgacgtgcgacgccgaggcggtc gaggacgtcgccgagcgcgagggtgtgagcgcggtgccgtattttgtgttttatcgcgac ggtaagcgcgtggacgcggtggagggggcggacgcggcgacggtgacgaacaagacgcgc atgtactttccggcgacggcgacgcagacgaccgcgacgacggcgaaaggcgcggacgcg gtgaagtcgacggaggcgagcgggaagcaggcgcttcaggcgcggttgaaatcgctgatc gagagccaaccggtggtgctgttcatgaagggacacccggacgaaccgaagtgcgggttc agccgaaaggtggtcgacgcgctcaacggcgccggaatcaagtttggttcgttcaacatt ctcgccgacgaggacgtgcgtcagggtttgaaggaatatagcaattggccgacgtacccg cagttgtacgtcgacggtgagctcttgggcgggtgcgacatcatactcgagatggctgag tccggagagctcgccgaggcgtgctcggaagcgtcgggcgacggtaaggtaaagaaggcg ctcaacgagcgaatcaagcgtatgcttgatgcgcaagacgtgattttgttcatgaagggt gaccgaaacgtcccgcgatgcggtttctctgggaaagtcgtcaaggctctggacgccaca ggagtcgagtacgcgacgttcgacatcctcggcgacgagccgattcgccaaggactgaag gagtactcaaactggccgacgtacccgcagctgtactacaagggcgagctcatcggcgga tgcgacatcatcttggagctcgccgaggcgggcgagctcgcgacggaactcggcgccgcg tga |
| Q011R3 | UniRef cluster | -------------------------------------------NERIKRMLDAQDVILFMKGDRNVPRCGFSG-KVVKALDATGV---EYAT---FDILG---DEPIRQGLKEYS----NWP----------TYPQLYYKGE--------LIGGCDI----ILELAEAGELAT-ELGAA--------------- | atgtcggtcgcgttgacggacgcggtggcgtacgacgcgttcaccgccgcgcgcgatcgc gcgatcgtgcacttcaccgcgagctggtgcgagccgtgtgcggcgatggagattgtcctg gaaaagattcgagagaaacacgcgggcgtcgtcacggcgacgtgcgacgccgaggcggtc gaggacgtcgccgagcgcgagggtgtgagcgcggtgccgtattttgtgttttatcgcgac ggtaagcgcgtggacgcggtggagggggcggacgcggcgacggtgacgaacaagacgcgc atgtactttccggcgacggcgacgcagacgaccgcgacgacggcgaaaggcgcggacgcg gtgaagtcgacggaggcgagcgggaagcaggcgcttcaggcgcggttgaaatcgctgatc gagagccaaccggtggtgctgttcatgaagggacacccggacgaaccgaagtgcgggttc agccgaaaggtggtcgacgcgctcaacggcgccggaatcaagtttggttcgttcaacatt ctcgccgacgaggacgtgcgtcagggtttgaaggaatatagcaattggccgacgtacccg cagttgtacgtcgacggtgagctcttgggcgggtgcgacatcatactcgagatggctgag tccggagagctcgccgaggcgtgctcggaagcgtcgggcgacggtaaggtaaagaaggcg ctcaacgagcgaatcaagcgtatgcttgatgcgcaagacgtgattttgttcatgaagggt gaccgaaacgtcccgcgatgcggtttctctgggaaagtcgtcaaggctctggacgccaca ggagtcgagtacgcgacgttcgacatcctcggcgacgagccgattcgccaaggactgaag gagtactcaaactggccgacgtacccgcagctgtactacaagggcgagctcatcggcgga tgcgacatcatcttggagctcgccgaggcgggcgagctcgcgacggaactcggcgccgcg tga |
| Q9UVH0 | UniRef cluster | -------------------------------------------NARLKELTSSSSVMAFIKGTPTAPRCQFSR-QLLEILTAQNI---RFSS---FNILA---DDEVRQAMKTFS----DWP----------TFPQVYVKGE--------FVGGLDV----VKELV-ASGEFQALVPAEKDLK----------- | atggtctccaacaactatatagacatcaccagcgaagacgactttgcgcaagtcttccag ccctcctcatccactgtctatgctctgaacttttgggcggcatgggcacctccttgcgtc caaatgaatgaggtctttgaagaactggccgccaagaacgctaatgtgaactttctcaag attgaggcagaaaagtttcccgacatttccgaagactatgagatcgctgctgttccaagc ttcgttatcgtgaaggagggtactgtcgtcgatcgcgtggagggagctaacgcacccgag ctggccaaagtcatcgccaagtactcaaagtcaacgtcatcgcctttaccaacacagagt tcgaccatggcggccgcaggacacgcagcgccttctgttgcccctccaacaatgtctccc gaagagatgaacgctcggctcaaggagctgaccagcagttcgagtgtcatggcttttatc aaaggaacacctacagctcccagatgccagttctctcgccagctcctggaaatcttgact gctcaaaacatccgattcagctccttcaacattcttgctgacgatgaggtccgacaggcc atgaagaccttttcggactggccaacattccctcaggtgtatgtgaagggtgagtttgtc ggaggactggatgttgtcaaggagctggttgcatctggcgagttccaggccctggtgcct gctgaaaaggatctgaagactcgcatggatgagctgattcgcaaggcacctgtgatgatc tttatcaaaggcagccccgagacgcctcgttgcggcttttcgaagtaa |
| Q9UVH0 | UniRef cluster | -------------------------------------------KTRMDELIRKAPVMIFIKGSPETPRCGFSK------------------------------------------------------------------------------------------------------------------------- | atggtctccaacaactatatagacatcaccagcgaagacgactttgcgcaagtcttccag ccctcctcatccactgtctatgctctgaacttttgggcggcatgggcacctccttgcgtc caaatgaatgaggtctttgaagaactggccgccaagaacgctaatgtgaactttctcaag attgaggcagaaaagtttcccgacatttccgaagactatgagatcgctgctgttccaagc ttcgttatcgtgaaggagggtactgtcgtcgatcgcgtggagggagctaacgcacccgag ctggccaaagtcatcgccaagtactcaaagtcaacgtcatcgcctttaccaacacagagt tcgaccatggcggccgcaggacacgcagcgccttctgttgcccctccaacaatgtctccc gaagagatgaacgctcggctcaaggagctgaccagcagttcgagtgtcatggcttttatc aaaggaacacctacagctcccagatgccagttctctcgccagctcctggaaatcttgact gctcaaaacatccgattcagctccttcaacattcttgctgacgatgaggtccgacaggcc atgaagaccttttcggactggccaacattccctcaggtgtatgtgaagggtgagtttgtc ggaggactggatgttgtcaaggagctggttgcatctggcgagttccaggccctggtgcct gctgaaaaggatctgaagactcgcatggatgagctgattcgcaaggcacctgtgatgatc tttatcaaaggcagccccgagacgcctcgttgcggcttttcgaagtaa |
| A9S689 | UniRef cluster | -------------------------------------------KGRLHELVNSKKVMLFMKGSPEEPRCGFSR-KVVNVLNDQGV---EFGS---FDILS---DETVRQGMKTYA----NWP----------TFPQLYVEGE--------LLGGCDI----ILEMNENGELKE-VFAEKGLLP----------- | atgggagtagcggagatggccggagttgtaagggatgcgaattccaagggagatttggac aatgcagtgaagaaacaaggcagtctggttgtagtgcacttttgggcttcctggtgtgaa ccgtctaaggctatggagcccgtttttactcagatcgctatcgaaacacctaatgcccaa tttttccgtgtggaggctgaggaacagtccgatatatctgagacttatgaggttgatgca gtgccgctgttcatttggattaaggatggagtggtagttgacaaaatgcaaggtgcgaat gctcctgaactggccagtaaagtagccaagtgggtaaaggatacacctgctccgttcaat gaggtgaaaaaagaggtcccctctgctggcgtagctgttgtaggccctgctgtttcaagt cagggcgaaaccaaagctaacctcgccgaagccgagaagggtaggcttcatgaacttgtg aattcgaagaaagtcatgcttttcatgaaaggatcaccagaggaaccccgctgcggtttc agccgcaaggtggtcaatgttttgaatgaccagggcgtggagtttggcagctttgacatc ttgagcgatgagacggtgaggcagggtatgaagacatacgctaattggcccacatttccc cagctctacgttgagggcgagcttctgggtggatgtgatataattttggagatgaatgag aacggcgagctcaaagaggtttttgctgagaaggggctactaccaaaagagactttggag actcgactgaagaatgtcatcaaccaatccgctactatgctgttcatgaaaggaacgcca gatgctccacgttgtggctttagcacaaaagttgtcaacgcgttgaaagaagaaggaatt gaatttggcagctttaacattttggaggacgaagaggttcgtcagggacttaagacatac tcaaactggcctacgtacccacagttgtattacaagggcgagcttctcggtggctgcgat attatcttagaaatgaaagcttctggagagttaaagtcagctctcaccgagtaa |
| A9S689 | UniRef cluster | -------------------------------------------ETRLKNVINQSATMLFMKGTPDAPRCGFST-KVVNALKEEGI---EFGS---FNILE---DEEVRQGLKTYS----NWP----------TYPQLYYKGE--------LLGGCDI----ILEMKASGELKS-ALTE---------------- | atgggagtagcggagatggccggagttgtaagggatgcgaattccaagggagatttggac aatgcagtgaagaaacaaggcagtctggttgtagtgcacttttgggcttcctggtgtgaa ccgtctaaggctatggagcccgtttttactcagatcgctatcgaaacacctaatgcccaa tttttccgtgtggaggctgaggaacagtccgatatatctgagacttatgaggttgatgca gtgccgctgttcatttggattaaggatggagtggtagttgacaaaatgcaaggtgcgaat gctcctgaactggccagtaaagtagccaagtgggtaaaggatacacctgctccgttcaat gaggtgaaaaaagaggtcccctctgctggcgtagctgttgtaggccctgctgtttcaagt cagggcgaaaccaaagctaacctcgccgaagccgagaagggtaggcttcatgaacttgtg aattcgaagaaagtcatgcttttcatgaaaggatcaccagaggaaccccgctgcggtttc agccgcaaggtggtcaatgttttgaatgaccagggcgtggagtttggcagctttgacatc ttgagcgatgagacggtgaggcagggtatgaagacatacgctaattggcccacatttccc cagctctacgttgagggcgagcttctgggtggatgtgatataattttggagatgaatgag aacggcgagctcaaagaggtttttgctgagaaggggctactaccaaaagagactttggag actcgactgaagaatgtcatcaaccaatccgctactatgctgttcatgaaaggaacgcca gatgctccacgttgtggctttagcacaaaagttgtcaacgcgttgaaagaagaaggaatt gaatttggcagctttaacattttggaggacgaagaggttcgtcagggacttaagacatac tcaaactggcctacgtacccacagttgtattacaagggcgagcttctcggtggctgcgat attatcttagaaatgaaagcttctggagagttaaagtcagctctcaccgagtaa |
| GRS11 | UniRef cluster | -------------------------------------------NKRLEQLVNSHPVFLFMKGTPEQPRCGFSR-KVVDVLKQEGV---EFGS---FDILT---DNDVREGMKKFS----NWP----------TFPQLYCKGE--------LLGGCDI----VIAMHESGELKD-VFKEHNIPL----------- |  |
| A3C671 | UniRef cluster | -------------------------------------------NKRLEQLVNSHPVFLFMKGTPEQPRCGFSR-KVVDVLKQEGV---EFGS---FDILT---DNDVREGMKKFS----NWP----------TFPQLYCKGE--------LLGGCDI----VIAMHESGELKD-VFKEHNIPL----------- | atggcggcggtgagggaggtggggtcgaaggcggagctggaggcggcggcgggaggggcg cgggccgccgcggtgcacttctgggcggcgtggtgcgaggcgtccaagcagatggacgag gtcttcgcgcacctcgccgtcgacttctcccacgccgtcttcctccgggttgaagctgag gaacaacccgaaatttcagaggcatatggagttacagcagtgccatattttgttttcttg aaggaaggtaaaactgttgatactctggagggtgcaaatccagccagcttggccaataag gttgcaaagttagctgggcctgccagcgttgctgagtctgctgtgcctgctagcctgggt gtggctgctgggcctgctgtacttgaaaaggttcaagagatggcacagcaaaatggagct tctgccactagtagtgcagaagatgcattgaacaagagattggagcagcttgtcaattcc catcccgtcttcttatttatgaagggaacccctgagcaaccaaggtgtggtttcagtcga aaagtagttgacgttttgaagcaggaaggagttgaatttgggagctttgacatcctaaca gataacgatgtacgtgaaggaatgaaaaagttctcaaactggccgacttttcctcagctc tactgcaaaggtgagctgcttggtggatgtgatattgtgattgctatgcatgaaagcggt gaactgaaggatgtttttaaggagcacaacattccgctgcagccacagggaagcaaaaac gaggaggcagtgaaagccaagcctgatactgagaagagtggtgcagtttctgaaccagct ttgcttactgcagctcagaaggaacgcttggaaagccttgttaatttcagcacagtgatg gcatttataaaaggtacacctgaggagcccaagtgtggattcagtggaaaactagtgcat attcttaagcaagagaagatccctttctcaagttttgacattcttacggatgatgaggtt aggcagggtctaaagcttctctcaaactggcctagttaccctcaactgtacataaacggt gaactggttggcggatcagacattgttatggagatgcataagagtggggagcttaagaag gttctatctgagaaagggatcgttgcgaaagaaagtctagaagaccgcctgaaggccctg atttcctctgccccagtgatgctcttcatgaagggcaccccagatgccccccgctgcggc ttcagttcgaaggttgtgaatgcactgaagcaagcaggagtcagcttcggagcgttcgac atcctatccgacgaggaggttaggcaaggcttgaagacgtactccaactggcccacgttc cctcagctgtactacaaatcagaactgattggaggctgtgacatcgttcttgagctggag aagagtggagagctgaagtccacgctttcggagtga |
| A2Z916 | UniRef cluster | -------------------------------------------NKRLEQLVNSHPVFLFMKGTPEQPRCGFSR-KVVDVLKQEGV---EFGS---FDILT---DNDVREGMKKFS----NWP----------TFPQLYCKGE--------LLGGCDI----VIAMHESGELKD-VFKEHNIPL----------- | atggcgccggtgagggaggtggggtcgaaggcggagctggaggcggcggcgggaggggcg cgggccgccgcggtgcacttctgggcggcgtggtgcgaggcgtccaagcagatggacgag gtcttcgcgcacctcgccgtcgacttcccccacgccgtcttcctccgggttgaagctgag gaacaacccgaaatttcagaggcatatggagttacagcagtgccatattttgttttcttg aaggaaggtaaaactgttgatactctggagggtgcaaatccagccagcttggccaataag gttgcaaagttagctgggcctgccagcgttgctgagtctgctgtgcctgctagcctgggt gtggctgctgggcctgctgtacttgaaaaggttcaagagatggcacagcaaaatggagct tcttccactagtagtgcagaagatgcattgaacaagagattggagcagcttgtcaattcc catcccgtcttcttatttatgaagggaacccctgagcaaccaaggtgtggtttcagtcga aaagtagttgacgttttgaagcaggaaggagttgaatttgggagctttgacatcctaaca gataacgatgtacgtgaaggaatgaaaaagttctcaaactggccgacttttcctcagctc tactgcaaaggtgagctgcttggtggatgtgatattgtgattgctatgcatgaaagcggt gaactgaaggatgtttttaaggagcacaacattccgctgcagccacagggaagcaaaaac gaggaggcagtgaaagccaagcctgatactgagaagagtggtgcagtttctgaaccagct ttgcttactgcagctcagaaggaacgcttggaaagccttgttaattccagcacagtgatg gcaattataaaaggtacacctgaggagcccaagtgtggattcagtggaaaactagtgcat attcttaagcaagagaagatccctttctcaagttttgacattcttacggatgatgaggtt aggcagggtctaaagcttctctcaaactggcctagttaccctcaactgtacataaacggt gaactggttggcggatcagacattgttatggagatgcataagagtggggagcttaagaag gttctatctgagaaagggatcgttgcgaaagaaagtctagaagaccgcctgaaggccctg atttcctctgccccagtgatgctcttcatgaagggcaccccagatgccccccgctgcggc ttcagttcgaaggttgtgaatgcactgaagcaagcaggagtcagcttcggagcgttcgac atcctatccgacgaggaggttaggcaaggcttgaagacgtactccaactggcccacgttc cctcagctgtactacaaatcagaactgattggaggctgtgacatcgttcttgagctggag aagagtggagagctgaagtccacgctttcggagtga |
| GRS11 | UniRef cluster | -------------------------------------------KERLESLVNFSTVMAFIKGTPEEPKCGFSG-KLVHILKQEKI---PFSS---FDILT---DDEVRQGLKLLS----NWP----------SYPQLYINGE--------LVGGSDI----VMEMHKSGELKK-VLSEKGIVA----------- |  |
| A3C671 | UniRef cluster | -------------------------------------------KERLESLVNFSTVMAFIKGTPEEPKCGFSG-KLVHILKQEKI---PFSS---FDILT---DDEVRQGLKLLS----NWP----------SYPQLYINGE--------LVGGSDI----VMEMHKSGELKK-VLSEKGIVA----------- | atggcggcggtgagggaggtggggtcgaaggcggagctggaggcggcggcgggaggggcg cgggccgccgcggtgcacttctgggcggcgtggtgcgaggcgtccaagcagatggacgag gtcttcgcgcacctcgccgtcgacttctcccacgccgtcttcctccgggttgaagctgag gaacaacccgaaatttcagaggcatatggagttacagcagtgccatattttgttttcttg aaggaaggtaaaactgttgatactctggagggtgcaaatccagccagcttggccaataag gttgcaaagttagctgggcctgccagcgttgctgagtctgctgtgcctgctagcctgggt gtggctgctgggcctgctgtacttgaaaaggttcaagagatggcacagcaaaatggagct tctgccactagtagtgcagaagatgcattgaacaagagattggagcagcttgtcaattcc catcccgtcttcttatttatgaagggaacccctgagcaaccaaggtgtggtttcagtcga aaagtagttgacgttttgaagcaggaaggagttgaatttgggagctttgacatcctaaca gataacgatgtacgtgaaggaatgaaaaagttctcaaactggccgacttttcctcagctc tactgcaaaggtgagctgcttggtggatgtgatattgtgattgctatgcatgaaagcggt gaactgaaggatgtttttaaggagcacaacattccgctgcagccacagggaagcaaaaac gaggaggcagtgaaagccaagcctgatactgagaagagtggtgcagtttctgaaccagct ttgcttactgcagctcagaaggaacgcttggaaagccttgttaatttcagcacagtgatg gcatttataaaaggtacacctgaggagcccaagtgtggattcagtggaaaactagtgcat attcttaagcaagagaagatccctttctcaagttttgacattcttacggatgatgaggtt aggcagggtctaaagcttctctcaaactggcctagttaccctcaactgtacataaacggt gaactggttggcggatcagacattgttatggagatgcataagagtggggagcttaagaag gttctatctgagaaagggatcgttgcgaaagaaagtctagaagaccgcctgaaggccctg atttcctctgccccagtgatgctcttcatgaagggcaccccagatgccccccgctgcggc ttcagttcgaaggttgtgaatgcactgaagcaagcaggagtcagcttcggagcgttcgac atcctatccgacgaggaggttaggcaaggcttgaagacgtactccaactggcccacgttc cctcagctgtactacaaatcagaactgattggaggctgtgacatcgttcttgagctggag aagagtggagagctgaagtccacgctttcggagtga |
| GRS11 | UniRef cluster | -------------------------------------------EDRLKALISSAPVMLFMKGTPDAPRCGFSS-KVVNALKQAGV---SFGA---FDILS---DEEVRQGLKTYS----NWP----------TFPQLYYKSE--------LIGGCDI----VLELEKSGELKS-TLSE---------------- |  |
| A3C671 | UniRef cluster | -------------------------------------------EDRLKALISSAPVMLFMKGTPDAPRCGFSS-KVVNALKQAGV---SFGA---FDILS---DEEVRQGLKTYS----NWP----------TFPQLYYKSE--------LIGGCDI----VLELEKSGELKS-TLSE---------------- | atggcggcggtgagggaggtggggtcgaaggcggagctggaggcggcggcgggaggggcg cgggccgccgcggtgcacttctgggcggcgtggtgcgaggcgtccaagcagatggacgag gtcttcgcgcacctcgccgtcgacttctcccacgccgtcttcctccgggttgaagctgag gaacaacccgaaatttcagaggcatatggagttacagcagtgccatattttgttttcttg aaggaaggtaaaactgttgatactctggagggtgcaaatccagccagcttggccaataag gttgcaaagttagctgggcctgccagcgttgctgagtctgctgtgcctgctagcctgggt gtggctgctgggcctgctgtacttgaaaaggttcaagagatggcacagcaaaatggagct tctgccactagtagtgcagaagatgcattgaacaagagattggagcagcttgtcaattcc catcccgtcttcttatttatgaagggaacccctgagcaaccaaggtgtggtttcagtcga aaagtagttgacgttttgaagcaggaaggagttgaatttgggagctttgacatcctaaca gataacgatgtacgtgaaggaatgaaaaagttctcaaactggccgacttttcctcagctc tactgcaaaggtgagctgcttggtggatgtgatattgtgattgctatgcatgaaagcggt gaactgaaggatgtttttaaggagcacaacattccgctgcagccacagggaagcaaaaac gaggaggcagtgaaagccaagcctgatactgagaagagtggtgcagtttctgaaccagct ttgcttactgcagctcagaaggaacgcttggaaagccttgttaatttcagcacagtgatg gcatttataaaaggtacacctgaggagcccaagtgtggattcagtggaaaactagtgcat attcttaagcaagagaagatccctttctcaagttttgacattcttacggatgatgaggtt aggcagggtctaaagcttctctcaaactggcctagttaccctcaactgtacataaacggt gaactggttggcggatcagacattgttatggagatgcataagagtggggagcttaagaag gttctatctgagaaagggatcgttgcgaaagaaagtctagaagaccgcctgaaggccctg atttcctctgccccagtgatgctcttcatgaagggcaccccagatgccccccgctgcggc ttcagttcgaaggttgtgaatgcactgaagcaagcaggagtcagcttcggagcgttcgac atcctatccgacgaggaggttaggcaaggcttgaagacgtactccaactggcccacgttc cctcagctgtactacaaatcagaactgattggaggctgtgacatcgttcttgagctggag aagagtggagagctgaagtccacgctttcggagtga |
| A2Z916 | UniRef cluster | -------------------------------------------EDRLKALISSAPVMLFMKGTPDAPRCGFSS-KVVNALKQAGV---SFGA---FDILS---DEEVRQGLKTYS----NWP----------TFPQLYYKSE--------LIGGCDI----VLELEKSGELKS-TLSE---------------- | atggcgccggtgagggaggtggggtcgaaggcggagctggaggcggcggcgggaggggcg cgggccgccgcggtgcacttctgggcggcgtggtgcgaggcgtccaagcagatggacgag gtcttcgcgcacctcgccgtcgacttcccccacgccgtcttcctccgggttgaagctgag gaacaacccgaaatttcagaggcatatggagttacagcagtgccatattttgttttcttg aaggaaggtaaaactgttgatactctggagggtgcaaatccagccagcttggccaataag gttgcaaagttagctgggcctgccagcgttgctgagtctgctgtgcctgctagcctgggt gtggctgctgggcctgctgtacttgaaaaggttcaagagatggcacagcaaaatggagct tcttccactagtagtgcagaagatgcattgaacaagagattggagcagcttgtcaattcc catcccgtcttcttatttatgaagggaacccctgagcaaccaaggtgtggtttcagtcga aaagtagttgacgttttgaagcaggaaggagttgaatttgggagctttgacatcctaaca gataacgatgtacgtgaaggaatgaaaaagttctcaaactggccgacttttcctcagctc tactgcaaaggtgagctgcttggtggatgtgatattgtgattgctatgcatgaaagcggt gaactgaaggatgtttttaaggagcacaacattccgctgcagccacagggaagcaaaaac gaggaggcagtgaaagccaagcctgatactgagaagagtggtgcagtttctgaaccagct ttgcttactgcagctcagaaggaacgcttggaaagccttgttaattccagcacagtgatg gcaattataaaaggtacacctgaggagcccaagtgtggattcagtggaaaactagtgcat attcttaagcaagagaagatccctttctcaagttttgacattcttacggatgatgaggtt aggcagggtctaaagcttctctcaaactggcctagttaccctcaactgtacataaacggt gaactggttggcggatcagacattgttatggagatgcataagagtggggagcttaagaag gttctatctgagaaagggatcgttgcgaaagaaagtctagaagaccgcctgaaggccctg atttcctctgccccagtgatgctcttcatgaagggcaccccagatgccccccgctgcggc ttcagttcgaaggttgtgaatgcactgaagcaagcaggagtcagcttcggagcgttcgac atcctatccgacgaggaggttaggcaaggcttgaagacgtactccaactggcccacgttc cctcagctgtactacaaatcagaactgattggaggctgtgacatcgttcttgagctggag aagagtggagagctgaagtccacgctttcggagtga |
| GRS11 | UniRef cluster | -------------------------------------------NKRLEQLVNSHPVFLFMKGTPEQPRCGFSR-KVVDVLKQEGV---EFGS---FDILT---DNDVREGMKKFS----NWP----------TFPQLYCKGE--------LLGGCDI----VIAMHESGELKD-VFKEHNIPL----------- |  |
| A3C671 | UniRef cluster | -------------------------------------------NKRLEQLVNSHPVFLFMKGTPEQPRCGFSR-KVVDVLKQEGV---EFGS---FDILT---DNDVREGMKKFS----NWP----------TFPQLYCKGE--------LLGGCDI----VIAMHESGELKD-VFKEHNIPL----------- | atggcggcggtgagggaggtggggtcgaaggcggagctggaggcggcggcgggaggggcg cgggccgccgcggtgcacttctgggcggcgtggtgcgaggcgtccaagcagatggacgag gtcttcgcgcacctcgccgtcgacttctcccacgccgtcttcctccgggttgaagctgag gaacaacccgaaatttcagaggcatatggagttacagcagtgccatattttgttttcttg aaggaaggtaaaactgttgatactctggagggtgcaaatccagccagcttggccaataag gttgcaaagttagctgggcctgccagcgttgctgagtctgctgtgcctgctagcctgggt gtggctgctgggcctgctgtacttgaaaaggttcaagagatggcacagcaaaatggagct tctgccactagtagtgcagaagatgcattgaacaagagattggagcagcttgtcaattcc catcccgtcttcttatttatgaagggaacccctgagcaaccaaggtgtggtttcagtcga aaagtagttgacgttttgaagcaggaaggagttgaatttgggagctttgacatcctaaca gataacgatgtacgtgaaggaatgaaaaagttctcaaactggccgacttttcctcagctc tactgcaaaggtgagctgcttggtggatgtgatattgtgattgctatgcatgaaagcggt gaactgaaggatgtttttaaggagcacaacattccgctgcagccacagggaagcaaaaac gaggaggcagtgaaagccaagcctgatactgagaagagtggtgcagtttctgaaccagct ttgcttactgcagctcagaaggaacgcttggaaagccttgttaatttcagcacagtgatg gcatttataaaaggtacacctgaggagcccaagtgtggattcagtggaaaactagtgcat attcttaagcaagagaagatccctttctcaagttttgacattcttacggatgatgaggtt aggcagggtctaaagcttctctcaaactggcctagttaccctcaactgtacataaacggt gaactggttggcggatcagacattgttatggagatgcataagagtggggagcttaagaag gttctatctgagaaagggatcgttgcgaaagaaagtctagaagaccgcctgaaggccctg atttcctctgccccagtgatgctcttcatgaagggcaccccagatgccccccgctgcggc ttcagttcgaaggttgtgaatgcactgaagcaagcaggagtcagcttcggagcgttcgac atcctatccgacgaggaggttaggcaaggcttgaagacgtactccaactggcccacgttc cctcagctgtactacaaatcagaactgattggaggctgtgacatcgttcttgagctggag aagagtggagagctgaagtccacgctttcggagtga |
| A2Z916 | UniRef cluster | -------------------------------------------NKRLEQLVNSHPVFLFMKGTPEQPRCGFSR-KVVDVLKQEGV---EFGS---FDILT---DNDVREGMKKFS----NWP----------TFPQLYCKGE--------LLGGCDI----VIAMHESGELKD-VFKEHNIPL----------- | atggcgccggtgagggaggtggggtcgaaggcggagctggaggcggcggcgggaggggcg cgggccgccgcggtgcacttctgggcggcgtggtgcgaggcgtccaagcagatggacgag gtcttcgcgcacctcgccgtcgacttcccccacgccgtcttcctccgggttgaagctgag gaacaacccgaaatttcagaggcatatggagttacagcagtgccatattttgttttcttg aaggaaggtaaaactgttgatactctggagggtgcaaatccagccagcttggccaataag gttgcaaagttagctgggcctgccagcgttgctgagtctgctgtgcctgctagcctgggt gtggctgctgggcctgctgtacttgaaaaggttcaagagatggcacagcaaaatggagct tcttccactagtagtgcagaagatgcattgaacaagagattggagcagcttgtcaattcc catcccgtcttcttatttatgaagggaacccctgagcaaccaaggtgtggtttcagtcga aaagtagttgacgttttgaagcaggaaggagttgaatttgggagctttgacatcctaaca gataacgatgtacgtgaaggaatgaaaaagttctcaaactggccgacttttcctcagctc tactgcaaaggtgagctgcttggtggatgtgatattgtgattgctatgcatgaaagcggt gaactgaaggatgtttttaaggagcacaacattccgctgcagccacagggaagcaaaaac gaggaggcagtgaaagccaagcctgatactgagaagagtggtgcagtttctgaaccagct ttgcttactgcagctcagaaggaacgcttggaaagccttgttaattccagcacagtgatg gcaattataaaaggtacacctgaggagcccaagtgtggattcagtggaaaactagtgcat attcttaagcaagagaagatccctttctcaagttttgacattcttacggatgatgaggtt aggcagggtctaaagcttctctcaaactggcctagttaccctcaactgtacataaacggt gaactggttggcggatcagacattgttatggagatgcataagagtggggagcttaagaag gttctatctgagaaagggatcgttgcgaaagaaagtctagaagaccgcctgaaggccctg atttcctctgccccagtgatgctcttcatgaagggcaccccagatgccccccgctgcggc ttcagttcgaaggttgtgaatgcactgaagcaagcaggagtcagcttcggagcgttcgac atcctatccgacgaggaggttaggcaaggcttgaagacgtactccaactggcccacgttc cctcagctgtactacaaatcagaactgattggaggctgtgacatcgttcttgagctggag aagagtggagagctgaagtccacgctttcggagtga |
| GRS11 | UniRef cluster | -------------------------------------------KERLESLVNFSTVMAFIKGTPEEPKCGFSG-KLVHILKQEKI---PFSS---FDILT---DDEVRQGLKLLS----NWP----------SYPQLYINGE--------LVGGSDI----VMEMHKSGELKK-VLSEKGIVA----------- |  |
| A3C671 | UniRef cluster | -------------------------------------------KERLESLVNFSTVMAFIKGTPEEPKCGFSG-KLVHILKQEKI---PFSS---FDILT---DDEVRQGLKLLS----NWP----------SYPQLYINGE--------LVGGSDI----VMEMHKSGELKK-VLSEKGIVA----------- | atggcggcggtgagggaggtggggtcgaaggcggagctggaggcggcggcgggaggggcg cgggccgccgcggtgcacttctgggcggcgtggtgcgaggcgtccaagcagatggacgag gtcttcgcgcacctcgccgtcgacttctcccacgccgtcttcctccgggttgaagctgag gaacaacccgaaatttcagaggcatatggagttacagcagtgccatattttgttttcttg aaggaaggtaaaactgttgatactctggagggtgcaaatccagccagcttggccaataag gttgcaaagttagctgggcctgccagcgttgctgagtctgctgtgcctgctagcctgggt gtggctgctgggcctgctgtacttgaaaaggttcaagagatggcacagcaaaatggagct tctgccactagtagtgcagaagatgcattgaacaagagattggagcagcttgtcaattcc catcccgtcttcttatttatgaagggaacccctgagcaaccaaggtgtggtttcagtcga aaagtagttgacgttttgaagcaggaaggagttgaatttgggagctttgacatcctaaca gataacgatgtacgtgaaggaatgaaaaagttctcaaactggccgacttttcctcagctc tactgcaaaggtgagctgcttggtggatgtgatattgtgattgctatgcatgaaagcggt gaactgaaggatgtttttaaggagcacaacattccgctgcagccacagggaagcaaaaac gaggaggcagtgaaagccaagcctgatactgagaagagtggtgcagtttctgaaccagct ttgcttactgcagctcagaaggaacgcttggaaagccttgttaatttcagcacagtgatg gcatttataaaaggtacacctgaggagcccaagtgtggattcagtggaaaactagtgcat attcttaagcaagagaagatccctttctcaagttttgacattcttacggatgatgaggtt aggcagggtctaaagcttctctcaaactggcctagttaccctcaactgtacataaacggt gaactggttggcggatcagacattgttatggagatgcataagagtggggagcttaagaag gttctatctgagaaagggatcgttgcgaaagaaagtctagaagaccgcctgaaggccctg atttcctctgccccagtgatgctcttcatgaagggcaccccagatgccccccgctgcggc ttcagttcgaaggttgtgaatgcactgaagcaagcaggagtcagcttcggagcgttcgac atcctatccgacgaggaggttaggcaaggcttgaagacgtactccaactggcccacgttc cctcagctgtactacaaatcagaactgattggaggctgtgacatcgttcttgagctggag aagagtggagagctgaagtccacgctttcggagtga |
| GRS11 | UniRef cluster | -------------------------------------------NKRLEQLVNSHPVFLFMKGTPEQPRCGFSR-KVVDVLKQEGV---EFGS---FDILT---DNDVREGMKKFS----NWP----------TFPQLYCKGE--------LLGGCDI----VIAMHESGELKD-VFKEHNIPL----------- |  |
| A3C671 | UniRef cluster | -------------------------------------------NKRLEQLVNSHPVFLFMKGTPEQPRCGFSR-KVVDVLKQEGV---EFGS---FDILT---DNDVREGMKKFS----NWP----------TFPQLYCKGE--------LLGGCDI----VIAMHESGELKD-VFKEHNIPL----------- | atggcggcggtgagggaggtggggtcgaaggcggagctggaggcggcggcgggaggggcg cgggccgccgcggtgcacttctgggcggcgtggtgcgaggcgtccaagcagatggacgag gtcttcgcgcacctcgccgtcgacttctcccacgccgtcttcctccgggttgaagctgag gaacaacccgaaatttcagaggcatatggagttacagcagtgccatattttgttttcttg aaggaaggtaaaactgttgatactctggagggtgcaaatccagccagcttggccaataag gttgcaaagttagctgggcctgccagcgttgctgagtctgctgtgcctgctagcctgggt gtggctgctgggcctgctgtacttgaaaaggttcaagagatggcacagcaaaatggagct tctgccactagtagtgcagaagatgcattgaacaagagattggagcagcttgtcaattcc catcccgtcttcttatttatgaagggaacccctgagcaaccaaggtgtggtttcagtcga aaagtagttgacgttttgaagcaggaaggagttgaatttgggagctttgacatcctaaca gataacgatgtacgtgaaggaatgaaaaagttctcaaactggccgacttttcctcagctc tactgcaaaggtgagctgcttggtggatgtgatattgtgattgctatgcatgaaagcggt gaactgaaggatgtttttaaggagcacaacattccgctgcagccacagggaagcaaaaac gaggaggcagtgaaagccaagcctgatactgagaagagtggtgcagtttctgaaccagct ttgcttactgcagctcagaaggaacgcttggaaagccttgttaatttcagcacagtgatg gcatttataaaaggtacacctgaggagcccaagtgtggattcagtggaaaactagtgcat attcttaagcaagagaagatccctttctcaagttttgacattcttacggatgatgaggtt aggcagggtctaaagcttctctcaaactggcctagttaccctcaactgtacataaacggt gaactggttggcggatcagacattgttatggagatgcataagagtggggagcttaagaag gttctatctgagaaagggatcgttgcgaaagaaagtctagaagaccgcctgaaggccctg atttcctctgccccagtgatgctcttcatgaagggcaccccagatgccccccgctgcggc ttcagttcgaaggttgtgaatgcactgaagcaagcaggagtcagcttcggagcgttcgac atcctatccgacgaggaggttaggcaaggcttgaagacgtactccaactggcccacgttc cctcagctgtactacaaatcagaactgattggaggctgtgacatcgttcttgagctggag aagagtggagagctgaagtccacgctttcggagtga |
| A2Z916 | UniRef cluster | -------------------------------------------NKRLEQLVNSHPVFLFMKGTPEQPRCGFSR-KVVDVLKQEGV---EFGS---FDILT---DNDVREGMKKFS----NWP----------TFPQLYCKGE--------LLGGCDI----VIAMHESGELKD-VFKEHNIPL----------- | atggcgccggtgagggaggtggggtcgaaggcggagctggaggcggcggcgggaggggcg cgggccgccgcggtgcacttctgggcggcgtggtgcgaggcgtccaagcagatggacgag gtcttcgcgcacctcgccgtcgacttcccccacgccgtcttcctccgggttgaagctgag gaacaacccgaaatttcagaggcatatggagttacagcagtgccatattttgttttcttg aaggaaggtaaaactgttgatactctggagggtgcaaatccagccagcttggccaataag gttgcaaagttagctgggcctgccagcgttgctgagtctgctgtgcctgctagcctgggt gtggctgctgggcctgctgtacttgaaaaggttcaagagatggcacagcaaaatggagct tcttccactagtagtgcagaagatgcattgaacaagagattggagcagcttgtcaattcc catcccgtcttcttatttatgaagggaacccctgagcaaccaaggtgtggtttcagtcga aaagtagttgacgttttgaagcaggaaggagttgaatttgggagctttgacatcctaaca gataacgatgtacgtgaaggaatgaaaaagttctcaaactggccgacttttcctcagctc tactgcaaaggtgagctgcttggtggatgtgatattgtgattgctatgcatgaaagcggt gaactgaaggatgtttttaaggagcacaacattccgctgcagccacagggaagcaaaaac gaggaggcagtgaaagccaagcctgatactgagaagagtggtgcagtttctgaaccagct ttgcttactgcagctcagaaggaacgcttggaaagccttgttaattccagcacagtgatg gcaattataaaaggtacacctgaggagcccaagtgtggattcagtggaaaactagtgcat attcttaagcaagagaagatccctttctcaagttttgacattcttacggatgatgaggtt aggcagggtctaaagcttctctcaaactggcctagttaccctcaactgtacataaacggt gaactggttggcggatcagacattgttatggagatgcataagagtggggagcttaagaag gttctatctgagaaagggatcgttgcgaaagaaagtctagaagaccgcctgaaggccctg atttcctctgccccagtgatgctcttcatgaagggcaccccagatgccccccgctgcggc ttcagttcgaaggttgtgaatgcactgaagcaagcaggagtcagcttcggagcgttcgac atcctatccgacgaggaggttaggcaaggcttgaagacgtactccaactggcccacgttc cctcagctgtactacaaatcagaactgattggaggctgtgacatcgttcttgagctggag aagagtggagagctgaagtccacgctttcggagtga |
[truncated: 734,312 more chars]
