# Supplementary material for: Evolution based on domain combinations: the case of glutaredoxins
Source: BMC Evol Biol. 2009 Mar 25;9:66. doi: 10.1186/1471-2148-9-66 (PMC2679010; doi:10.1186/1471-2148-9-66)
Supplement: Additional File 7 — Aligned sequences of CGFS-class GRX domains from multidomain proteins. Alignment used to build the phylogenetic trees. [file 1471-2148-9-66-S7.doc]

Supplementary Table I: Analysis of CGFS-class GRX genes in Archaea. The phylogenetic trees were made using NCBI’s tree-viewing applets. We show the 2o residues immediately bfore and after each gene in the corresponding genomes.

| **Organism** | **Accession numbers** | **Homology** | **Flanking Regions (20 residues)** | **Homology of flanking regions** |
| --- | --- | --- | --- | --- |
| *Halobacterium sp. NRC-1* | NCBI-GI: [15789689](http://www.ncbi.nih.gov/entrez/query.fcgi?cmd=Retrieve&db=Protein&list_uids=15789689&dopt=GenPept) NCBI-GeneID: [1447297](http://www.ncbi.nih.gov/entrez/query.fcgi?db=gene&cmd=Retrieve&dopt=Graphics&list_uids=1447297) UniProt: [Q9HS15](http://www.expasy.org/uniprot/Q9HS15) | **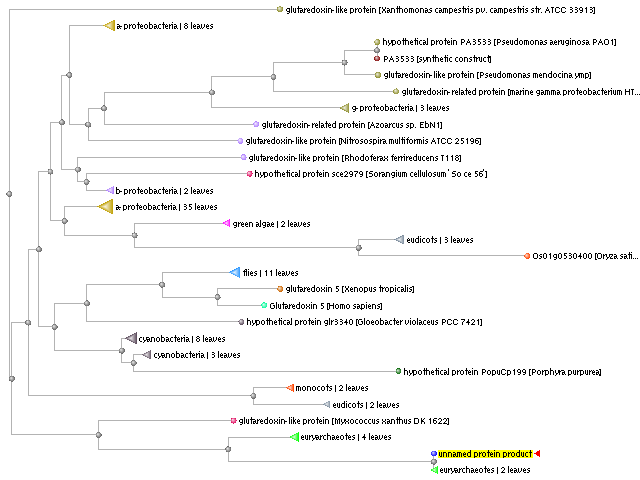** | **Upstream:**  TCGATCCGCTCGACTCGTCG  **Downstream:**  CGGGGCCGTCGCTAGTAGAT  **Match**  TCGATCCGCTCGACTCGTCG  T_GATG__C_C__C_GG_GC | Xanthomonas axonopodis |
| **Organism** | **Accession numbers** | **Homology** | **Flanking Regions** | **Homology of flanking regions** |
| *Haloarcula marismortui* | NCBI-GI: [55377038](http://www.ncbi.nih.gov/entrez/query.fcgi?cmd=Retrieve&db=Protein&list_uids=55377038&dopt=GenPept) NCBI-GeneID: [3130213](http://www.ncbi.nih.gov/entrez/query.fcgi?db=gene&cmd=Retrieve&dopt=Graphics&list_uids=3130213) UniProt: [Q5V5M0](http://www.expasy.org/uniprot/Q5V5M0) | **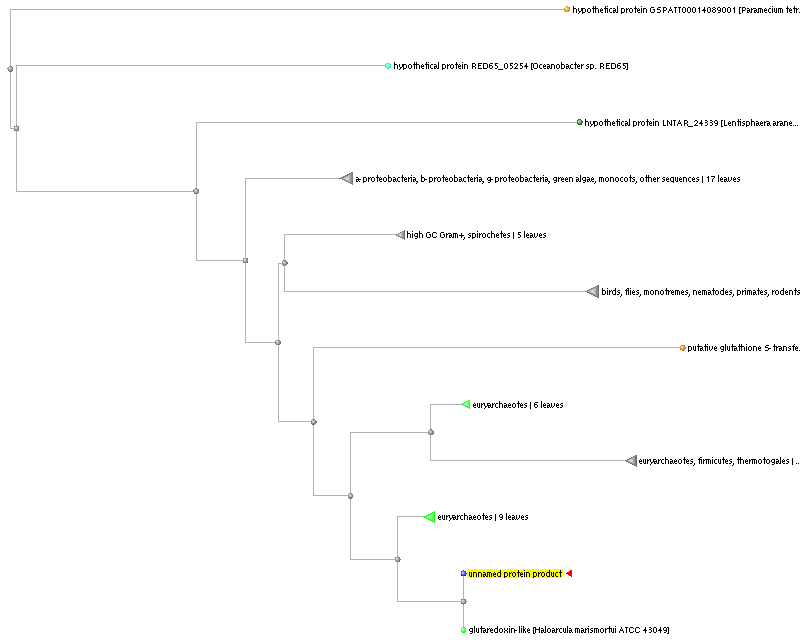** | Upstream  ACGGCGTCCGTATCGCCAGT  Downstream  AGATAGCGAGCGGCCGCGCC  Palindrome Match  ACGGCGTCCGTATCGCCAGT  _CGCGC_GGC_A_CG__AGA | No |
| **Organism** | **Accession numbers** | **Homology** | **Flanking Regions** | **Homology of Flanking regions** |
| *Haloarcula marismortui* | NCBI-GI: [55378099](http://www.ncbi.nih.gov/entrez/query.fcgi?cmd=Retrieve&db=Protein&list_uids=55378099&dopt=GenPept) NCBI-GeneID: [3127930](http://www.ncbi.nih.gov/entrez/query.fcgi?db=gene&cmd=Retrieve&dopt=Graphics&list_uids=3127930) UniProt: [Q5V2K9](http://www.expasy.org/uniprot/Q5V2K9) | **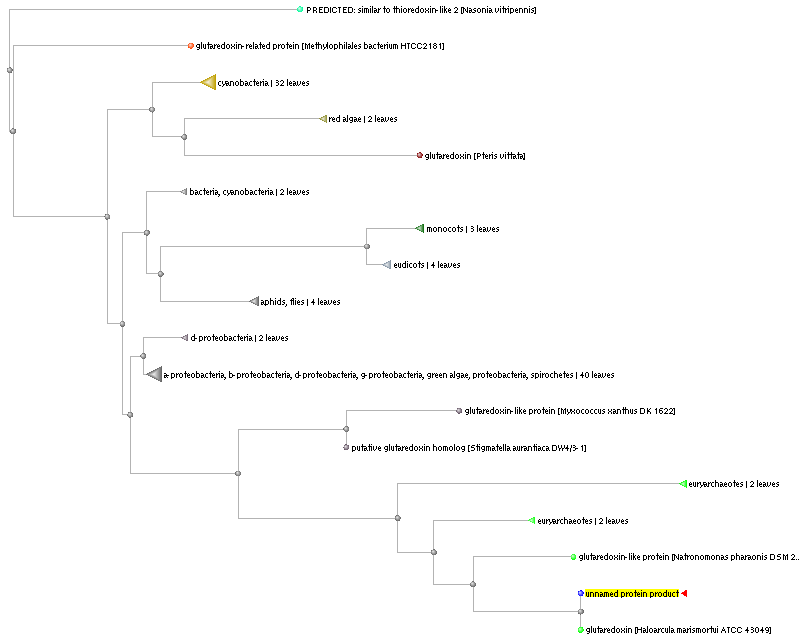** | Upstream  AGCAGAGTAGCCGGGTCTCG  Downstream  GCGCGGTCGGGAGTGGCGGC  Palindrome  AGCAGAGTAGCCGGGTCTCG  _GG_G___AGGGC_G_C_CG | Xanthomonas axonopodis |
| **Organism** | **Accession numbers** | **Homology** | **Flanking Regions** | **Homology of flanking regions** |
| Halobacterium salinarum R1 | NCBI-GI: [169235403](http://www.ncbi.nih.gov/entrez/query.fcgi?cmd=Retrieve&db=Protein&list_uids=169235403&dopt=GenPept) NCBI-GeneID: [5954172](http://www.ncbi.nih.gov/entrez/query.fcgi?db=gene&cmd=Retrieve&dopt=Graphics&list_uids=5954172) UniProt: [B0R3E0](http://www.expasy.org/uniprot/B0R3E0) | **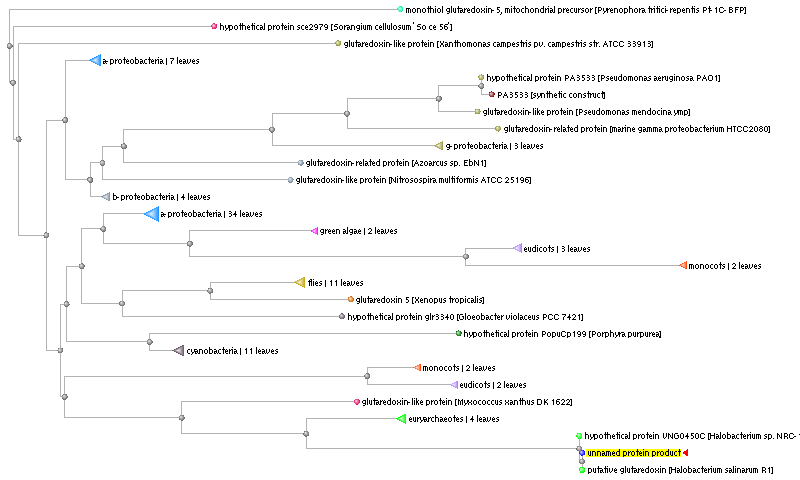** | **Upstream**  CTGCCCCCCGACGGTGGGA  **Downstream**  CGGGGCCGTCGCTAGTAGAT  **Palindrome**  CTGCCCCCCGACGGTGGGA  _AG__G__CG__GC_GGG_ | Xanthomonas axonopodis |
| **Organism** | **Accession numbers** | **Homology** | **Flanking Regions** | **Homology of Flanking regions** |
| Natronomonas pharaonis | NCBI-GI: [76801678](http://www.ncbi.nih.gov/entrez/query.fcgi?cmd=Retrieve&db=Protein&list_uids=76801678&dopt=GenPept) NCBI-GeneID: [3701340](http://www.ncbi.nih.gov/entrez/query.fcgi?db=gene&cmd=Retrieve&dopt=Graphics&list_uids=3701340) UniProt: [Q3IRW7](http://www.expasy.org/uniprot/Q3IRW7) | **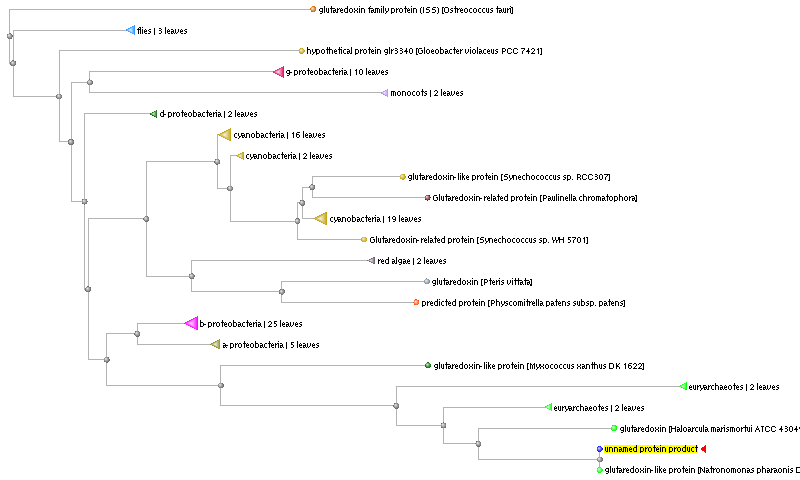** | **Upstream**  TCGATGCCGAACACGTTCCC  **Downstream**  GCCGTTCTGCGTTTCCGTAG  **Palindrome**  TCGATGCCGAACACGTTCCC  _____C_____CTC_T_CCG | No |
| **Organism** | **Accession numbers** | **Homology** | **Flanking Regions** | **Homology of Flanking regions** |
| Haloquadratum walsbyi | NCBI-GI: [110667622](http://www.ncbi.nih.gov/entrez/query.fcgi?cmd=Retrieve&db=Protein&list_uids=110667622&dopt=GenPept) NCBI-GeneID: [4192422](http://www.ncbi.nih.gov/entrez/query.fcgi?db=gene&cmd=Retrieve&dopt=Graphics&list_uids=4192422) UniProt: [Q18JL3](http://www.expasy.org/uniprot/Q18JL3) | **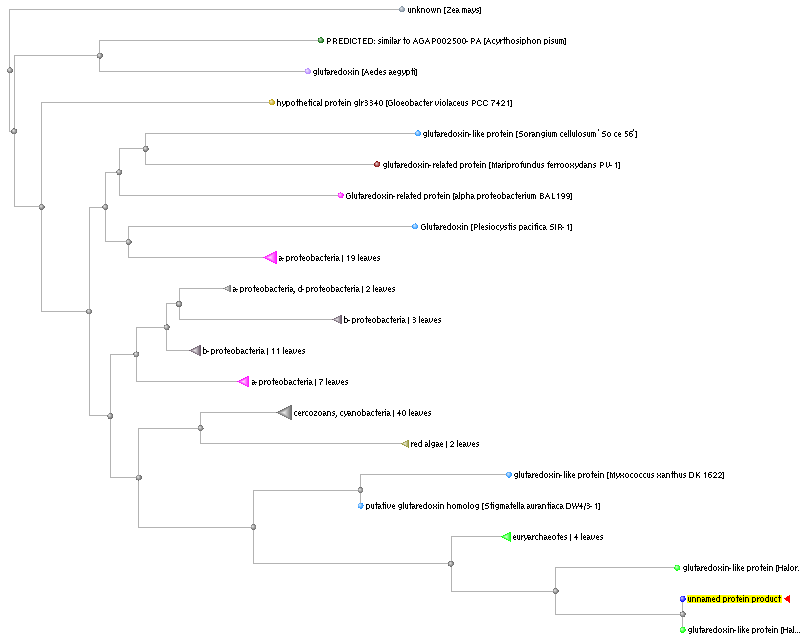** | **Upstream**  CTGAATTCATATAATAAGAT  **Downstream**  ACCCAGTCGGATGAGTATAT  **Palindrome**  CTGAATTCATATAATAAGAT  _A_AT_AGTA___ T_A_C_A | No |
